# Asymmetric synthesis of pharmaceutically relevant 1-aryl-2-heteroaryl- and 1,2-diheteroarylcyclopropane-1-carboxylates†

**DOI:** 10.1039/d1sc02474d

**Published:** 2021-07-27

**Authors:** Jack C. Sharland, Bo Wei, David J. Hardee, Timothy R. Hodges, Wei Gong, Eric A. Voight, Huw M. L. Davies

**Affiliations:** Department of Chemistry, Emory University 1515 Dickey Drive Atlanta GA 30322 USA hmdavie@emory.edu; Drug Discovery Science and Technology, AbbVie 1 North Waukegan Rd. North Chicago IL 60064 USA eric.a.voight@abbvie.com

## Abstract

This study describes general methods for the enantioselective syntheses of pharmaceutically relevant 1-aryl-2-heteroaryl- and 1,2-diheteroarylcyclopropane-1-carboxylates through dirhodium tetracarboxylate-catalysed asymmetric cyclopropanation of vinyl heterocycles with aryl- or heteroaryldiazoacetates. The reactions are highly diastereoselective and high asymmetric induction could be achieved using either (*R*)-pantolactone as a chiral auxiliary or chiral dirhodium tetracarboxylate catalysts. For *meta*- or *para*-substituted aryl- or heteroaryldiazoacetates the optimum catalyst was Rh_2_(*R-p*-Ph-TPCP)_4_. In the case of *ortho*-substituted aryl- or heteroaryldiazoacetates, the optimum catalyst was Rh_2_(*R*-TPPTTL)_4_. For a highly enantioselective reaction with the *ortho*-substituted substrates, 2-chloropyridine was required as an additive in the presence of either 4 Å molecular sieves or 1,1,1,3,3,3-hexafluoroisopropanol (HFIP). Under the optimized conditions, the cyclopropanation could be conducted in the presence of a variety of heterocycles, such as pyridines, pyrazines, quinolines, indoles, oxadiazoles, thiophenes and pyrazoles.

## Abstract 2

The dirhodium tetracarboxylate-catalysed asymmetric cyclopropanation has been applied to the enantioselective syntheses of pharmaceutically relevant 1-aryl-2-heteroaryl- and 1,2-diheteroarylcyclopropane-1-carboxylates.

## Graphical abstract

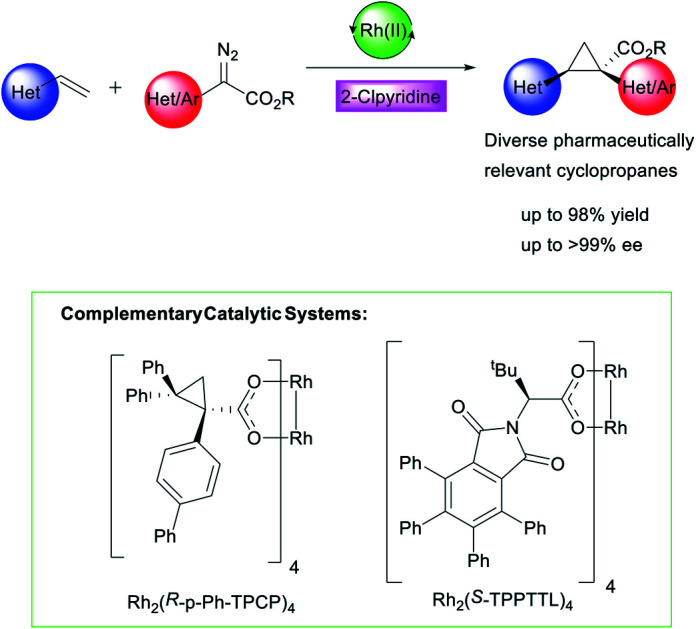

## Introduction

**Fig. 1:**
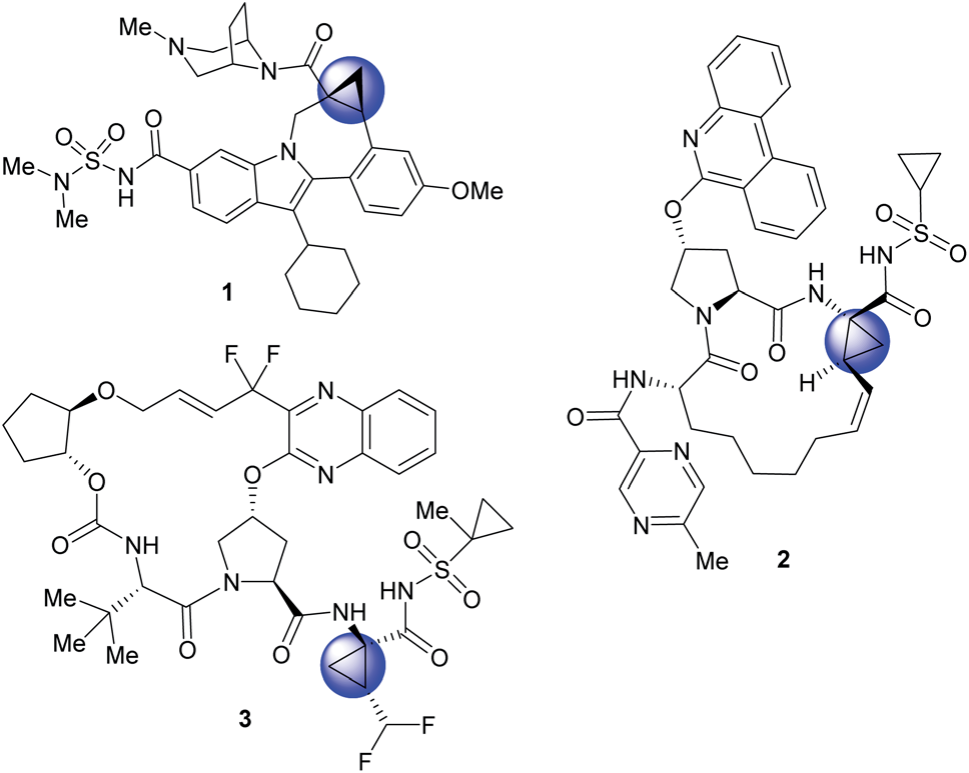
Examples of commercially available therapeutics containing highly substituted cyclopropanes. Beclabuvir (**1**), Paritaprevir (**2**), and Glecaprevir (**3**) therapeutics for the treatment of hepatitis-C (HCV).

**Scheme 1:**
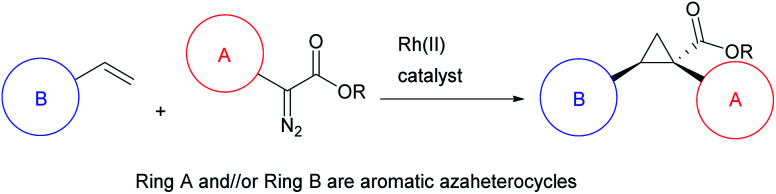
This work: determine the range of heterocycles that are compatible with enantioselective rhodium-catalysed cyclopropanation.

The cyclopropane ring is a common structural motif incorporated into many pharmaceutical agents.^1–4^ Particularly common are 1,1-disubstituted cyclopropanes.^5–10^ The two substituents are placed in a defined spacial arrangement to each other and the synthesis of such compounds is straightforward, as there is no additional chirality associated with the cyclopropane ring. In recent years more elaborate chiral cyclopropanes have been incorporated into therapeutic scaffolds, such as the trisubstituted cyclopropanes in beclabuvir (**1**),^11^ paritaprevir (**2**)^12,13^ and glecaprevir (**3**)^14–16^ (Fig. 1). In these cases, three substituents are placed in a defined orientation. The syntheses of these cyclopropanes, however, are more challenging because they contain two stereogenic centres which need to be generated in a diastereoselective and enantioselective manner. A general method for the stereoselective synthesis of tri- or tetrasubstituted cyclopropanes is the rhodium-catalysed cyclopropanation reactions of donor/acceptor carbenes.^17^ A distinctive characteristic of this cyclopropanation is its high diastereoselectivity, typically >30 : 1 d.r.^18^ Furthermore, effective methods are available to achieve asymmetric induction in the reaction by using either chiral auxiliaries^19^ or chiral catalysts.^18,20–24^ Having established the cyclopropanation chemistry, we were motivated to develop a general method to synthesize cyclopropane carboxylates with heterocyclic functionality of potential pharmaceutical interest (Scheme 1).

**Fig. 2:**
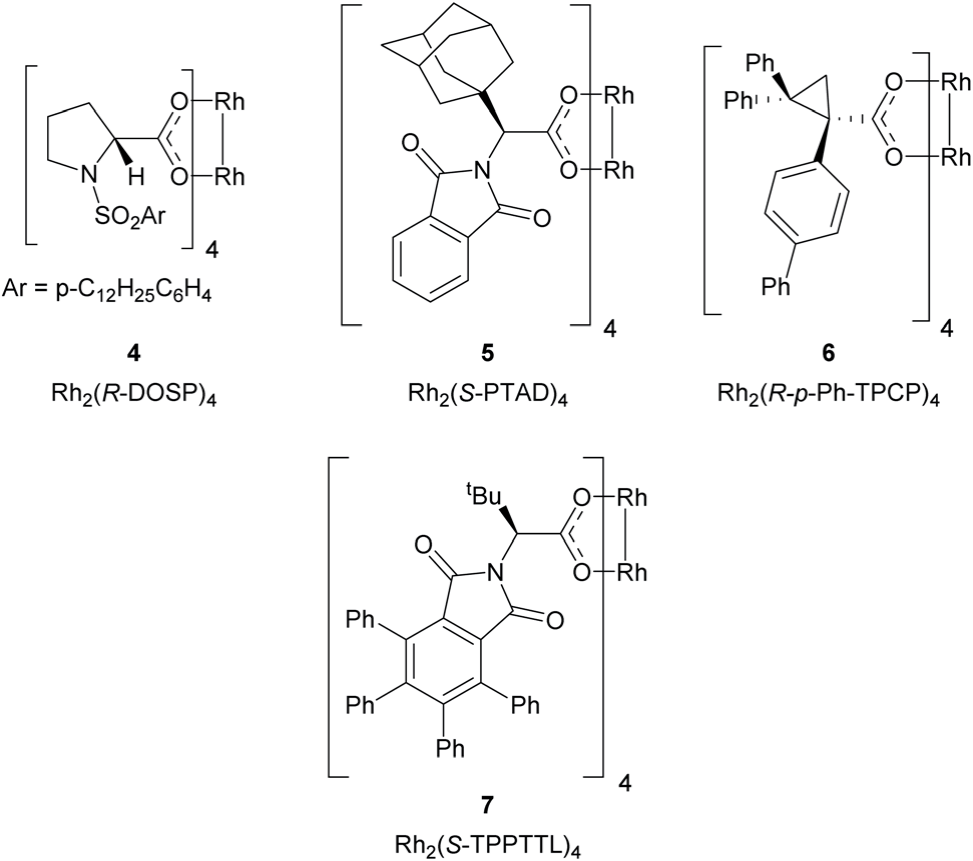
Structures of the key dirhodium catalysts used in this study.

The proposed method represents a significant challenge because the dirhodium catalysts and the rhodium–carbene intermediates are potentially susceptible to interactions with nucleophilic sites present in many heterocycles, which could interfere with the desired cyclopropanation unless carefully controlled.^25,26^ During our studies on cyclopropanation reactions with donor/acceptor carbenes, we developed two strategies for asymmetric induction. The first approach used α-hydroxyesters as chiral auxiliaries, and (*R*)-pantolactone was found to be particularly effective.^19^ Soon thereafter, we developed chiral dirhodium tetracarboxylate catalysts for asymmetric cyclopropanation,^18^ the first generally effective catalysts were *N*-sulfonylprolinate catalysts such as Rh_2_(*S*-DOSP)_4_ (**4**).^18^ Since then, a variety of other chiral dirhodium catalysts have been developed. Three of these catalysts, Rh_2_(*R*-PTAD)_4_,^20^ Rh_2_(*R-p*-Ph-TPCP)_4_ ^22^ and Rh_2_(*R*-TPPTTL)_4_ ^27^ (**5**, **6** and **7** respectively) play a significant role in the current study (Fig. 2).

Previous studies on cyclopropanation with heteroaryldiazoacetates as substrates gave mixed results.^22,28,29^ The Rh_2_(*S*-DOSP)_4_ (**4**)-catalysed cyclopropanation with methyl heteroaryldiazoacetates was generally high yielding and highly diastereoselective, but the levels of enantioselectivity were variable (23–89% ee).^29^ It was evident that nucleophilic heterocycles such as pyridine tended to poison the catalyst and forcing conditions were often required for the cyclopropanation reaction to proceed.^29^ More recently, a few trichloroethyl heteroaryldiazoacetates were shown to be capable of highly enantioselective cyclopropanation of styrene using Rh_2_(*R-p*-Ph-TPCP)_4_ (**6**) as catalyst.^22,28^ Inspired by these promising results, we decided to conduct a systematic study to determine the scope of the heterocycles that can be incorporated in both the diazo compounds and in the trapping alkenes (Scheme 1). The study described herein consists of four stages. The first stage was conducted with chiral auxiliaries to gain rapid entry to the chiral cyclopropanes and avoid the potential interference of heterocyclic substrates with chiral catalysts. The second stage explored the use of chiral catalysts to achieve cyclopropanation of vinyl heterocycles with *para*- and *meta*-substituted aryl- and heteroaryldiazoacetates, which proceeded with high yield and selectivity according to established protocols. The third stage studied *ortho*-substituted diazo compounds, which required considerable optimization, leading to the discovery of additives with unexpected influence on the enantioselectivity. Finally, studies are described to scale-up the transformation for a multi-gram synthesis and protocols for generating and using the diazo compound *in situ*.

## Results and discussion

**Scheme 2:**
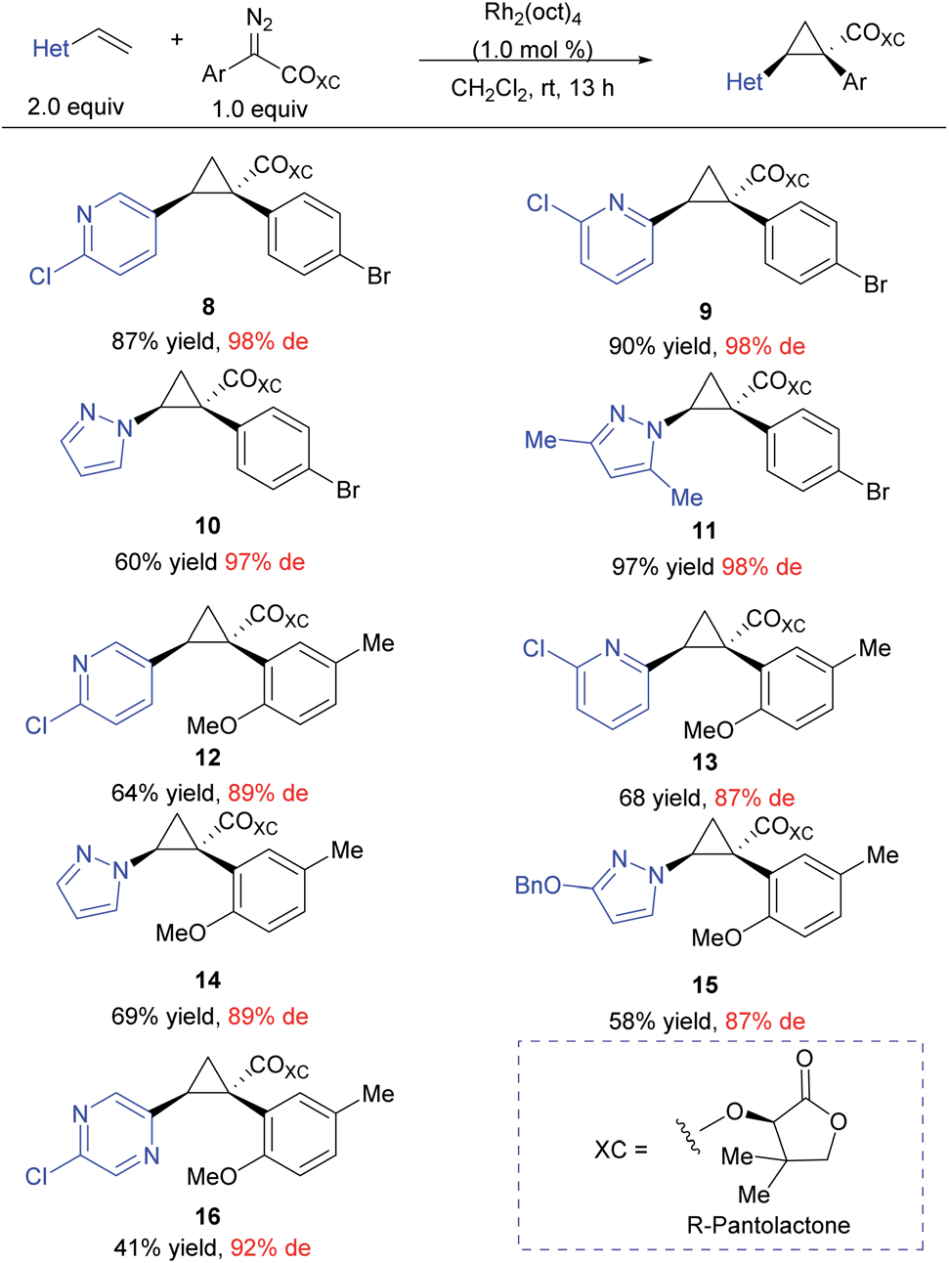
Scope of heterocycle-containing cyclopropanes including the chiral auxiliary (*R*)-pantolactone. Reaction was run on 0.20 mmol scale at room temperature using aryldiazoacetate (1.0 equiv.), vinyl-heterocycle (2.0 equiv.) and 1.0 mol% Rh_2_(oct)_4_ (0.2 μmol) with CH_2_Cl_2_ as solvent.

At the outset of this project we required rapid access to chiral 1,2-diaryl(heteroaryl)cyclopropane-1-carboxylates. We began by examining the chiral auxiliary approach using (*R*)-pantolactone.^19^ This approach is applicable to a wide range of substrates as summarized in Scheme 2. When applied towards the cyclopropanation of various vinyl heterocycles,^30,31^ the (*R*)-pantolactone-condensed-aryldiazoacetates gave routinely high asymmetric induction (87–98% de) and the process was suitable for the synthesis of a variety of heterocycle-substituted cyclopropanes. In general, reactions involving a *para*-substituted aryldiazoacetate gave slightly higher asymmetric induction than *ortho*-substituted analogues (**8–11** (97–98% de) *vs.***9–16** (87–89% de)). The absolute stereochemistry of **8–16** is tentatively assigned by analogy to the previously determined *Si* face selectivity exhibited by (*R*)-pantolactone in the reactions of donor/acceptor carbenes.^19^ While the chiral auxiliary approach proved generally effective, it does have limitations. The use of a stoichiometric amount of a chiral auxiliary is undesirable on large-scale due the cost and additional synthetic steps incurred for its installation and eventual removal. Additionally, only one of the enantiomers of pantolactone is relatively inexpensive, limiting the approach to ready accessibility to only one enantiomer of the cyclopropane product.^32^ For these reasons, while the (*R*)-pantolactone approach was useful for synthesizing a number of compounds in a short period of time, more contemporary methods using chiral catalysts were desirable.

There are few previously reported examples of highly enantioselective dirhodium-catalysed cyclopropanation involving heteroaryldiazoacetates.^22,28,29^ Even in the case of successful methodologies, the vast excess of substrate typically used in these reactions raises concerns that vinyl heterocycles, particularly pyridine derivatives, may interfere with the catalyst.^25,33,34^ In order to evaluate the influence of different heterocycles an assortment of vinyl heterocycles (2.32 equiv., see S8–S10 in the ESI† for synthetic details^30,31^) were reacted with 2,2,2-trichloroethyl 2-(4-bromophenyl)-2-diazoacetate (1.0 equiv.) (Table 1). The catalyst selected for this study was Rh_2_(*R-p*-Ph-TPCP)_4_ (**6**) (0.5 mol%), which has been shown to be the most effective chiral dirhodium tetracarboxylate catalyst for the cyclopropanation of styrenes.^22^ During the course of this previous study, considerable variability in the enantioselectivity was observed unless 10 weight equiv. of 4 Å molecular sieves was added to the reactions.^22^ Under these conditions, the reaction proved to be robust, generating a series of 1-aryl-2-heteroarylcyclopropane-1-carboxylates **17–25** with high enantioselectivity (83% to >99% ee). Either (MeO)_2_CO, the optimal solvent identified in the earlier study,^22^ or CH_2_Cl_2_, a generally effective solvent for donor/acceptor carbene transformations, could be used while maintaining high enantioselectivity. The reactions were competent with various pyridine (**17–21**) and quinoline derivatives (**22** and **23**), as well as five-membered heterocycles (**24** and **25**). The reactions of 2-chloro-5-vinyl pyridine were then conducted with a range of *para*- and *meta*-substituted methyl and trichloroethyl aryldiazoacetates and a styryldiazoacetate to generate the cyclopropanes **26–30**. Again, the reactions proceeded with high enantioselectivity (89–98% ee) except for the case of the 3,4-dimethoxy derivative, which generated the cyclopropane **30** in only 70% ee. The final series of reactions generated cyclopropanes **31–34** (83–95% ee) bearing two heteroaryl rings.

**Table 1:** Scope of vinyl heterocycles aryl-diazoacetates and heteroaryl-diazoacetates compatible with previously established high-TON cyclopropanation methodology in the presence of Rh_2_(*R-p*-Ph-TPCP)_4_ (**6**)^a^

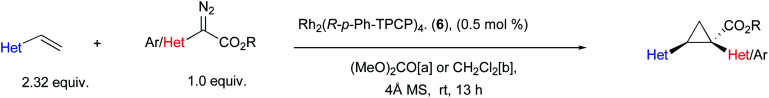
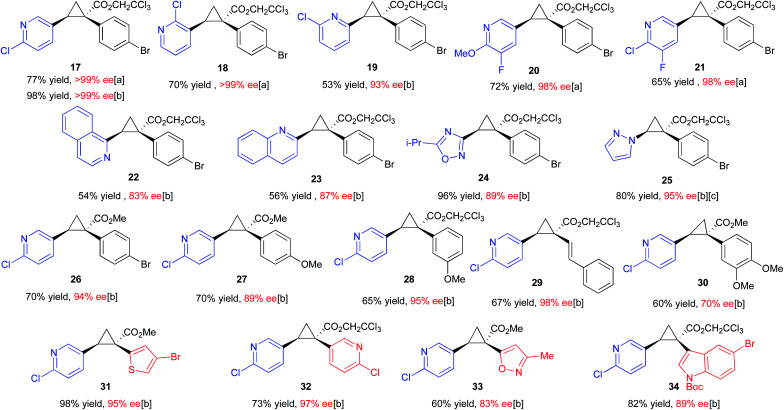

a Reactions were conducted on 0.20 mmol scale with 1.0 equiv. of diazo-compound, 2.32 equivalents of vinyl-heterocycle, 0.5 mol% catalyst loading (0.1 μmol) and either [a] (MeO)_2_CO or [b] CH_2_Cl_2_ as solvent depending on solubility and optimal enantioselectivity obtained. %Ee was determined by chiral HPLC, absolute configuration of **17** was determined by X-ray crystallography (CCDC 2071127). [c] Reaction was conducted with 1.0 mol% catalyst and run for 48 h at room temperature due to sluggish reactivity.

The absolute configuration of **17** was determined by X-ray crystallography and the absolute configurations of **18–34** are tentatively assigned by analogy.

The *ortho*-substituted aryldiazoacetates were particularly desirable substrates in this study, but unfortunately, the Rh_2_(*R-p*-Ph-TPCP)_4_ (**6**)-catalysed process, using the conditions described in Table 1, was not successful. The test cyclopropanation of 2-chloro-5-vinylpyridine (**35**) (2.5 equiv.), with the *ortho*-substituted aryldiazoacetate **36** under Rh_2_(*R-p*-Ph-TPCP)_4_ (**6**)-catalysed reaction conditions generated the product **37** in only 30% yield and 15% ee (Table 2, entry 1). While the stereoselective cyclopropanation of styrene with *ortho*-chlorophenyldiazoacetate has been reported in the presence of a second-generation dirhodium tetracarboxylate catalyst, Rh_2_(*S*-PTAD)_4_ (**5**),^20^ this transformation required pentane as solvent to ensure high asymmetric induction, which is incompatible with several of the vinyl heterocycles because of solubility issues.^29^ The Rh_2_(*R*-DOSP)_4_ (**4**)-catalysed reaction of **35** with **36** generated the cyclopropane **37** in only 22% ee (Table 2, entry 2). Similarly, the Rh_2_(*S*-PTAD)_4_ (**5**)-catalysed reaction gave low enantioselectivity (26% ee) (Table 2, entry 3).

**Table 2:** Optimization of the enantioselective cyclopropanation of a vinyl-heterocycle with an *ortho*-substituted aryldiazoacetate^a^

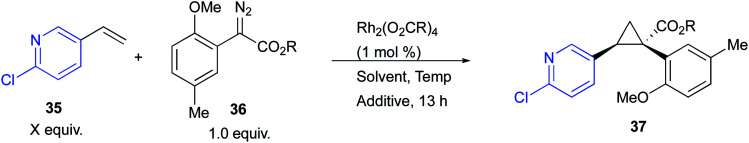								
Entry	Catalyst	Temp, °C	Additive	Solvent	Equiv. **35**	R	Yield, %	ee, %
1	Rh_2_(*R-p*-PhTPCP)_4_ (**6**)	25	4 Å Mol sieves	CH_2_Cl_2_	2.5	CH_3_	30	15
2	Rh_2_(*R*-DOSP)_4_ (**4**)	25	4 Å Mol sieves	CH_2_Cl_2_	2.5	CH_3_	68	22
3	Rh_2_(*S*-PTAD)_4_ (**5**)	25	4 Å Mol sieves	CH_2_Cl_2_	2.5	CH_3_	70	26
4	Rh_2_(*S*-TPPTTL)_4_ (**7**)	25	4 Å Mol sieves	CH_2_Cl_2_	2.5	CH_3_	88	60
5	Rh_2_(*S*-TPPTTL)_4_ (**7**)	25	4 Å Mol sieves	(MeO)_2_CO	2.5	CH_3_	74	35
6	Rh_2_(*S*-TPPTTL)_4_ (**7**)	25	4 Å Mol sieves	TFT	2.5	CH_3_	58	58
7	Rh_2_(*S*-TPPTTL)_4_ (**7**)	25	4 Å Mol sieves	CH_2_Cl_2_	2.5	CH_2_CCl_3_	47	39
8	Rh_2_(*S*-TPPTTL)_4_ (**7**)	0	4 Å Mol sieves	CH_2_Cl_2_	2.5	CH_3_	85	80
9	Rh_2_(*R*-TPPTTL)_4_ (**7**)	−50	4 Å Mol sieves	CH_2_Cl_2_	2.5	CH_3_	75	−89
10	Rh_2_(*S*-TPPTTL)_4_ (**7**)	0	4 Å Mol sieves	CH_2_Cl_2_	5.0	CH_3_	**95**	**98**
11	Rh_2_(*S*-TPPTTL)_4_ (**7**)	0	HFIP	CH_2_Cl_2_	5.0	CH_3_	**93**	**92**

a All reactions were conducted on 0.20 mmol scale using 1.0 mol% of Rh(ii) catalyst (0.2 μmol). (−) % ee denotes that the opposite enantiomer of **37** was obtained.

Due to the poor performance of the established catalysts, newer catalysts were evaluated in the reaction. Rh_2_(*S*-TPPTTL)_4_ (**7**), a recently developed catalyst with a unique selectivity profile,^27,35^ emerged as the optimal catalyst for this system, giving **37** in 88% yield and 60% ee (Table 2). Optimization of the Rh_2_(*R*-TPPTTL)_4_ (**7**)-catalysed reaction by changing solvent (entries 4–6) or changing from the methyl ester to trichloroethyl ester (entry 7), did not improve the reaction. Lowering the reaction temperature to 0 °C increased the level of enantioselectivity to 80% ee (entry 8). While further decreasing the temperature to −50 °C marginally increased the enantioselectivity, the overall yield was reduced (entry 9). The most dramatic effect, however, was to increase the amount of the 2-chloro-5-vinylpyridine (**35**) to 5 equiv., which resulted in the formation of **37** in 95% yield and 98% ee (entry 10).^26,36–40^ Even though the optimization studies resulted in a considerable improvement in the effectiveness of the reaction, we were concerned that the reaction would not be amenable to scale up with the use of 10 weight equiv. of molecular sieves to substrate. We have recently reported that 1,1,1,3,3,3-hexafluoroisopropanol (HFIP) has beneficial effects on certain rhodium-catalysed carbene reactions.^35^ Therefore, we decided to explore its effect on the optimized cyclopropanation, and we were pleased to observe that 10 equiv. of HFIP could be used in place of the 10 weight equiv. 4 Å molecular sieves and retain high enantioinduction (entry 11).

The optimized conditions developed in Table 2, were then applied to a range of substrates, but mixed results were obtained (Table 3). The Rh_2_(*S*-TPPTTL)_4_ (**7**)-catalysed reactions of aryldiazoacetate **36** with various vinyl 2-chloropyridines to form the cyclopropanes **37–39** were highly enantioselective (90–98% ee). In contrast the cyclopropanation of styrene with various *ortho*-substituted aryldiazoacetates generated the cyclopropanes **40–42** with low to moderate levels of enantioselectivity (4–64% ee). Improved enantioselectivity was obtained in the formation of cyclopropane **43** (77% ee), derived from a 2-chloropyridyldiazoacetate. The large variation in the levels of enantioselectivity was initially considered to be caused by trace impurities, but repeating the reactions with carefully purified starting materials did not change the enantioselectivity. Finally, as **37–39** and **43** all contain a 2-chloropyridyl component and are formed with high levels of enantioselectivity, it was proposed that a 2-chloropyridyl group may play a critical role in enhancing the enantioselectivity of the cyclopropanation. Such an effect would be consistent with the observed beneficial effect when using a large excess (5 equiv.) of 2-chloro-5-vinylpyridine seen in Table 2, entry 10.

**Table 3:** The substitution dependant effect of 2-chloropyridine on asymmetric cyclopropanation in the presence of Rh_2_(*S*-TPPTTL)_4_ (**7**)^a^

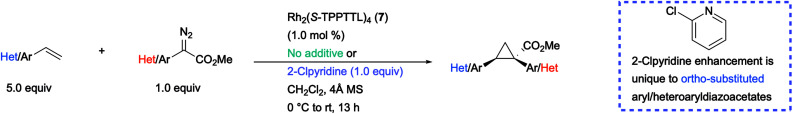
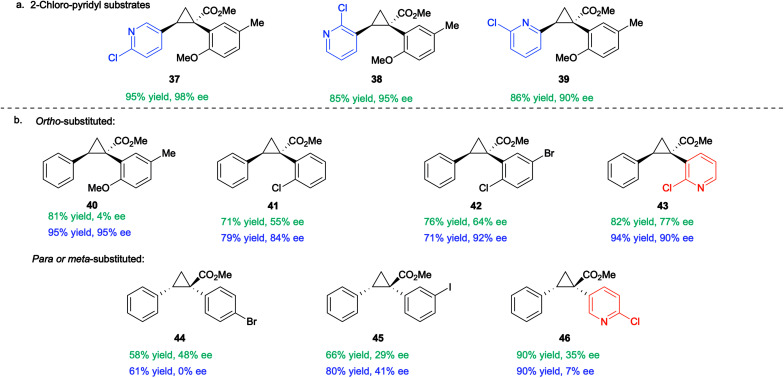

a Rh_2_(*S*-TPPTTL)_4_ (**7**)-catalysed cyclopropanation of styrene with various aryldiazoacetates [a] without additive and [b] with 1.0 equiv. of 2-Clpyridine as a coordinating additive. Reactions were conducted on 0.20 mmol scale with 1.0 mol% catalyst loading (0.2 μmol) and CH_2_Cl_2_ as solvent. The absolute configuration of **38** was determined by X-ray crystallography (CCDC 2071154). The absolute configuration of **37**, **39–43** is tentatively assigned by analogy to **38**. The absolute configuration of **44–46** is assigned by analogy to the X-ray characterization of the *para*-substituted product **17** (see Table 1). Rh_2_(*S*-TPPTTL)_4_ (**7**) affords the opposite configuration in cyclopropanations involving *ortho*-substituted aryldiazoacetates *vs. para*-substituted analogues.

A control reaction was conducted to test this hypothesis. The cyclopropanation to form **40** was repeated in the presence of 1 equiv. 2-chloropyridine as an additive. The modified conditions caused a dramatic effect on the enantioselectivity with **40** being formed in 95% ee compared to 4% ee in the absence of the additive. A systematic study was conducted with a range of pyridine and quinoline analogues (see S110–S116 in ESI† for details), which revealed that 2-chloropyridine was the optimum additive. Pyridines lacking a substituent adjacent to nitrogen tended to poison the catalyst.^29^ Quinoline and other 2-substituted pyridines, such as 2-methoxypyridine and 2-fluoropyridine, also provided considerable enhancement of enantioselectivity (70–93% ee) but none proved superior to 2-chloropyridine in this reaction. The unexpected positive influence of 2-chloropyridine prompted us to further evaluate its impact by investigating the Rh_2_(*S*-TPPTTL)_4_ (**7**)-catalysed cyclopropanation of styrene with representative aryl- and pyridyldiazoacetates (Table 3).

In the case of the cyclopropanes **41** and **42** derived from cyclopropanation of styrene with *ortho*-substituted aryldiazoacetates, the presence of 2-chloropyridine in the reaction improved the enantioselectivity from 55–77% ee to 84–92% ee. In contrast to Rh_2_(*R-p*-Ph-TPCP)_4_ (**6**), Rh_2_(*S*-TPPTTL)_4_ (**7**) is not an effective chiral catalyst for the formation of the cyclopropanes **44–46**, derived from diazo compounds lacking *ortho* substituents. The enantioselectivity is low in the absence of additive (29–48% ee) and even worse in the presence of 2-chloropyridine (0–41% ee). These studies demonstrated that while 2-chloropyridine as an additive can greatly enhance the enantioselectivity of Rh_2_(*S*-TPPTTL)_4_-catalysed cyclopropanation, the effect is unique to *ortho*-substituted aryl- and heteroaryldiazoacetates.

Having established the positive influence of 2-chloropyridine in the cyclopropanation studies using styrene, the reactions of *ortho*-substituted aryldiazoacetates was examined with a range of vinyl heterocycles as illustrated in the formation of the cyclopropanes **47–55** (Table 4). As many of the vinyl heterocycles are expensive or are not commercially available the reactions were carried out with just 1.5 equiv. of the vinyl heterocycle and 3.5 equiv. of 2-chloropyridine. During the reaction optimization (Table 2), it was found that 5.0 equiv. of coordinating additive (in this case vinyl heterocycle) was required to achieve high enantioselectivity. As a result, this ratio was preserved during elaboration of the substrate scope. The methodology proved compatible with a range of heterocycles, including pyridines, quinolines, isoquinolines, pyrazines, pyrazoles, and oxadiazoles. The reactions proceeded to form the cyclopropanes with generally very high enantioselectivity, ranging from 86% ee to >99% ee. The reaction could also be conducted with methyl 2-(2-chloropyridin-3-yl)-2-diazoacetate and in this case, 1,2-diheteroarylcycloproane carboxylates **62–65** were formed in 72–95% ee. Effective reactions could be carried out using either 4 Å molecular sieves or HFIP as co-additive. In the case of **37**, **47–49** the products were formed with high enantioselectivity using both sets of conditions.

**Table 4:** Scope of cyclopropanation with vinyl-heterocycles under the optimized *ortho*-aryldiazoacetate conditions^a^

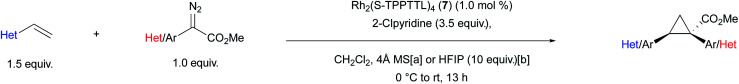
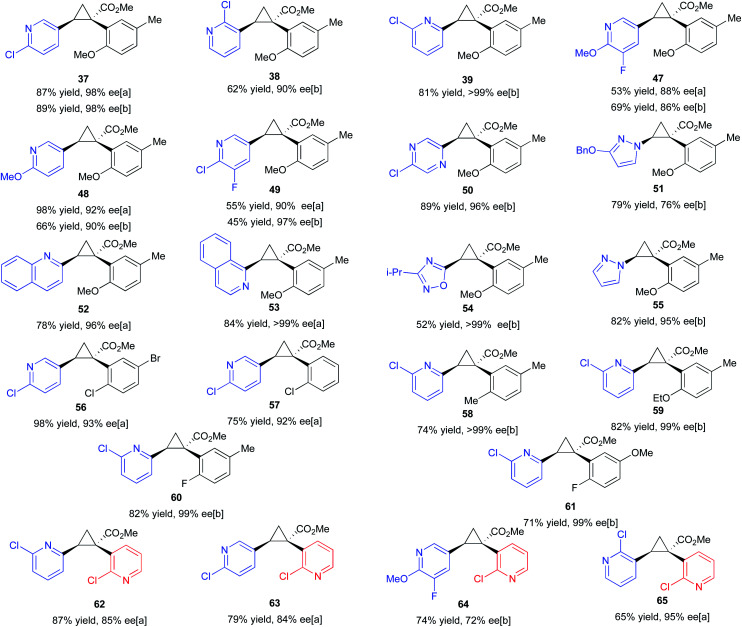

a Reactions were conducted on 0.20 mmol scale with 1.0 mol% Rh_2_(*S*-TPPTTL)_4_ (**7**) (0.2 μmol) CH_2_Cl_2_ as solvent, and a reduced loading of vinyl-heterocycle (1.5 equiv.) balanced out with 2-chloropyridine (3.5 equiv.) with [a] 10 weight equiv. 4 Å molecular sieves or [b] HFIP (10 equiv.). The absolute configuration of **37**, **39**, **47–65** is tentatively assigned by analogy to that of **38**, which was determined by X-ray crystallography (CCDC 2071154).

**Scheme 3:**
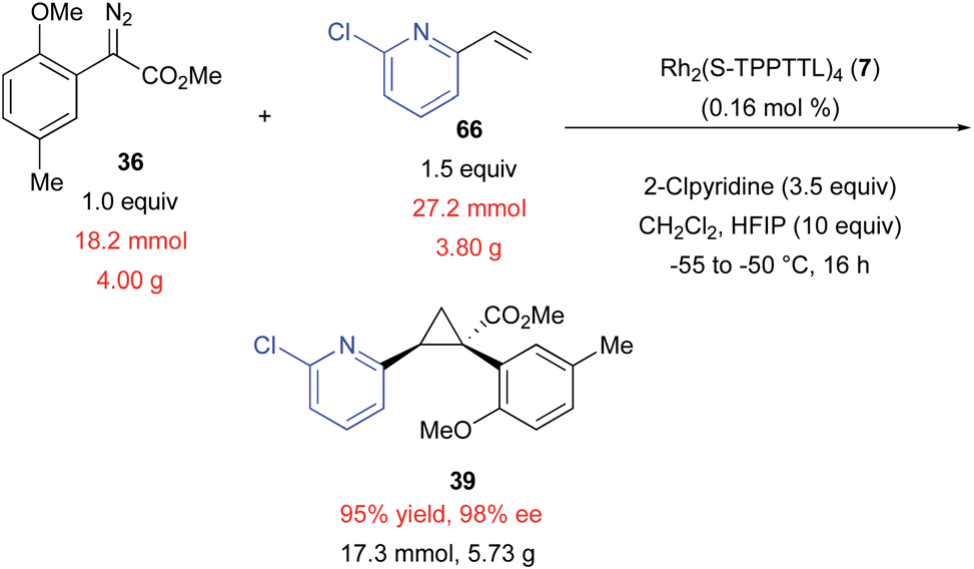
Enantioselective cyclopropanation on a multi-gram scale.

Exploratory studies were also conducted to determine whether the cyclopropanation reactions were amenable to scale-up.^11,41–44^ The replacement of molecular sieves with HFIP enabled the reaction to be performed on multi-gram scale, providing **39** in 95% yield and 98% ee (Scheme 3). Performing the reaction on large scale also enabled the use of considerably lower catalyst loading (0.16 mol% *vs.* 1.0 mol%). The reaction was conducted at lower temperature (−50 °C) to afford higher enantioselectivity, a common trend for dirhodium catalysed asymmetric cyclopropanation by donor/acceptor carbenes.^19^ In this reaction it was important to carefully control the temperature, keeping it below 0 °C throughout diazo addition. It had been previously observed that the reaction proceeds at a reasonable rate at −50 °C. The scale-up was therefore performed at this temperature to prevent the possibility of localized exotherms during diazo addition that could decrease overall reaction selectivity, resulting in product generation with high yield and stereoselectivity.

Ultimately, for the reaction to be amenable to very large scale synthesis, the diazo compound would need to be generated in flow to avoid working with large quantities of a high energy intermediate.^45^ Alternative methods are available for generating diazo-compounds from hydrazones through the use of several stoichiometric oxidants including MnO_2_, Ag_2_O, and iodamine-T.^46–48^ But in the long term a catalytic process is more desirable. We have recently developed a copper-catalysed method for the synthesis of diazo compounds from hydrazones, in which the only by-product is water.^49^ The copper catalysed-reaction is greatly accelerated with *N*,*N*′-dimethyl aminopyridine (DMAP), but DMAP, a very nucleophilic pyridine, would be expected to poison the catalyst or react with the carbene.^29,36,40^ Therefore, we have conducted exploratory studies to determine if the unpurified diazo compound from a copper-catalysed oxidation can be directly used in the rhodium-catalysed reaction. The copper-catalysed oxidation of **67** in the presence of DMAP in air generated the desired diazo compound **36** in essentially quantitative yield after stirring for 30 min. Addition of the resulting solution to another reaction flask containing the reagents for a rhodium-catalysed cyclopropanation failed to proceed unless HFIP was present.

**Scheme 4:**
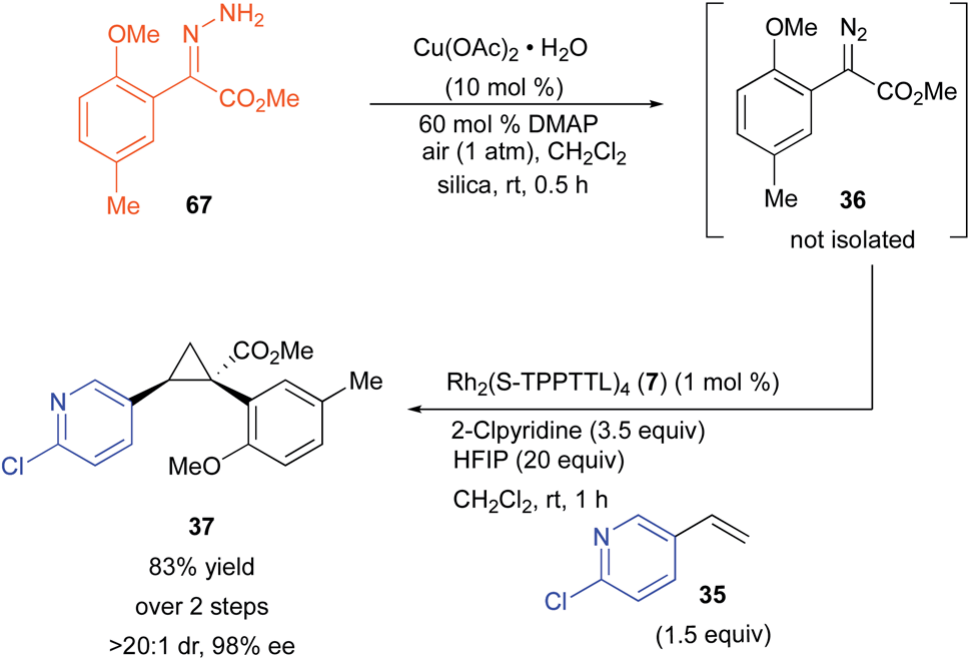
Sequential copper-catalysed diazo formation followed by a rhodium-catalysed cyclopropanation.

In the presence of HFIP (20 equiv.), the cyclopropane was formed in 83% yield and 98% ee (Scheme 4). The HFIP in this case is playing a very interesting role because it is deactivating the undesired effects of DMAP but still allowing the desirable influence of 2-chloropyridine to occur. A control experiment without HFIP resulted in recovery of unreacted diazo compound **36**. Presumably, the HFIP blocks the interference caused by the nucleophilic DMAP but does not block the positive influence of the less nucleophilic 2-chloropyridine.

One of the most intriguing features of the current study is the dramatic role of additives on the reaction with *ortho*-substituted aryl- and heteroaryldiazoacetates. Typically, the enantioselectivity of rhodium-catalysed cyclopropanation is not greatly influenced by trace moisture. Certainly, water will tend to cause a decrease in yield because it will competitively react with the carbene. In the case of *ortho*-substituted diazo compounds, trace moisture had a dramatically negative influence and a considerable excess (10 weight equiv.) of 4 Å molecular sieves was essential for reproducibly high enantioselectivity. Intriguingly, HFIP could be used in the place of molecular sieves and maintain similar levels of enantioselectivity. HFIP has been demonstrated to have a positive influence on a range of reactions,^50–57^ but the role of HFIP in rhodium-catalysed cyclopropanation is not definitively known at this stage. Presumably, it is involved in hydrogen bonding and this blocks interference from the water.^51,58,59^ The most unexpected effect was the role of 2-chloropyridine. In the absence of 2-chloropyridine, the enantioselectivity of cyclopropanations involving *ortho*-substituted diazo compounds was very poor unless the substrate itself contained a 2-chloropyridyl functionality.

**Fig. 3:**
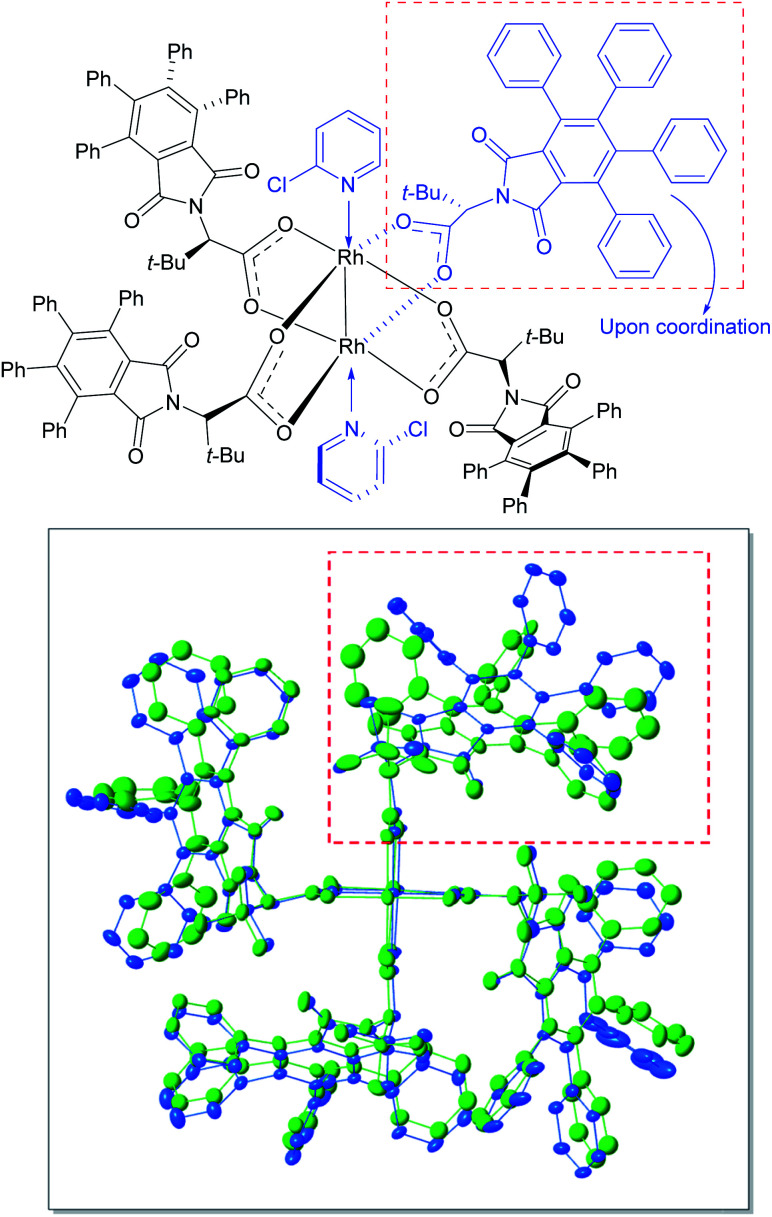
Structural perturbations in Rh_2_(*S*-TPPTTL)_4_ (**7**) enforced by the coordination of 2-chloropyridine based on X-ray analysis of a single crystal of **7** coordinated to 2-chloropyridine (CCDC 2071667†). The top-right ligand is displaced from its original position (green) upon coordination with 2-chloropyridine (blue). The axially coordinated ligands including 2-chlorpyridine ligand located inside of the bowl of the catalysts have been removed in order to give greater clarity of the overlaid structure of the catalysts.

In order to understand the influence of 2-chloropyridine better, crystals were grown of the 2-chloropyridine complex of Rh_2_(*S*-TPPTTL)_4_ (**7**). The crystal structure, contained 2-chloropyridine molecules bound to each rhodium axial site and one additional 2-chloropyridine situated within the bowl of the catalyst. An overlay of the previously reported crystal structure of the catalyst^27^ and the 2-chloropyridine-coordinated catalyst are shown in Fig. 3. The 2-chloropyridine molecules have been removed for clarity (the full structure of the complex, is shown in the ESI†). An intriguing feature of the two overlaid structures is that one of the ligands has been considerably displaced upon coordination to the 2-chloropyridine. This leads to an intriguing hypothesis that appropriate coordinating additives can alter the shape of the catalyst, which can then have a major influence on the asymmetric induction observed. Further studies need to be conducted to determine the origin of the positive influence of 2-chloropyridine as current understanding of the phenomenon is limited by the complexity of the system. Certainly, additives that would be expected to coordinate to the axial position of the dirhodium have been shown to influence the general outcome of carbene reactions, but the influence on enantioselectivity has not been extensively explored.^22,26,40,60–63^ The cyclopropanation of **35** with **36** generated *in situ* illustrates the additive effects of HFIP and 2-chloropyridine in concert. Without the presence of HFIP, the reaction cannot proceed, suggesting that DMAP acts as a poison to the rhodium catalyst, coordinating to the axial position and preventing carbene formation. However, in the presence of HFIP, the DMAP cannot coordinate, suggesting an interaction between DMAP and HFIP, possibly through protonation or hydrogen bonding.

2-Chloropyridine, however, is considerably less basic than DMAP,^64^ and apparently does not interact with HFIP in the same manner. As a result, the poisonous influence of DMAP is selectively deactivated while the beneficial coordination of 2-chloropyridine proceeds undisturbed.

## Conclusions

Complementary general methodologies for the syntheses of heterocycle-substituted cyclopropanes were developed. Use of (*R*)-pantolactone as a chiral auxiliary was identified as a direct and reliable way to synthesize stereoselectively a wide assortment of 1-aryl-2-heteroaryl- and 1,2-diheteroarylcyclopropane-1-carboxylates. Alternatively, 1-aryl-2-heteroaryl- and 1,2-diheteroarylcyclopropane-1-carboxylates could be generated with high enantioselectivity using chiral catalysts. *para* or *meta*-substituted aryldiazoacetates performed predictably and with high selectivity adapting recently reported cyclopropanation methodology using Rh_2_(*R-p*-Ph-TPCP)_4_ (**6**) as catalyst. The reaction could be extended to several heteroaryldiazoacetates, enabling access to 1,2-diheteroarylcyclopropane carboxylates. *ortho*-Substituted aryldiazoacetates, however, proved incompatible with these conditions and a different chiral catalyst, Rh_2_(*S*-TPPTTL)_4_ (**7**) was required. During these studies, additives were found to play a major role in controlling asymmetric induction. 2-Chloropyridine was discovered as a coordinating additive capable of significantly enhancing the enantioselectivity of cyclopropanation involving *ortho*-substituted aryldiazoacetates. These efforts resulted in a robust and generalizable methodology which was performed on multi-gram scale and made more process-amenable by substituting 4 Å molecular sieves for HFIP to desensitize the reaction to H_2_O. This *in situ* desensitization was further exploited to perform the reaction with aryldiazoacetate generated *in situ* from the corresponding hydrazone using copper-catalysed oxidation. These unique additive effects may have broad implications for other rhodium-catalysed carbene reactions.

## Experimental

See ESI for all experimental details. The following crystal structures have been deposited in the Cambridge Crystallographic Data Centre: **7** coordinated to 2-chloropyridine (CCDC 2071667), **17** (CCDC 2071127), and **38** (CCDC 2071154).†

## Author contributions

H. M. L. Davies is a named inventor on a patent entitled Dirhodium Catalyst Compositions and Synthetic Processes Related Thereto (US 8974428, issued 3/10/2015). D. J. Hardee, T. R. Hodges, W. Gong, and E. A. Voight are employees of AbbVie. AbbVie contributed to the design, study conduct, and financial support for this research. AbbVie participated in the interpretation of data, review, and approval of the publication.

## Conflicts of interest

The authors declare the following competing financial interests.

## Supplementary Material

SC-012-D1SC02474D-s001

SC-012-D1SC02474D-s002
