# Supplementary material for: Asymmetric synthesis of pharmaceutically relevant 1-aryl-2-heteroaryl- and 1,2-diheteroarylcyclopropane-1-carboxylates
Source: Chem Sci. 2021 Jul 27;12(33):11181–90. doi: 10.1039/d1sc02474d (PMC8386643; doi:10.1039/d1sc02474d)
Supplement: SC-012-D1SC02474D-s001 [file SC-012-D1SC02474D-s001.pdf]

Electronic Supplementary Material (ESI) for Chemical Science.
This journal is © The Royal Society of Chemistry 2021

Asymmetric Synthesis of Pharmaceutically Relevant 1-Aryl-2-heteroaryl- and 1,2-Diheteroarylcyclopropane-1-carboxylates.

Jack C. Sharland,[a] Bo Wei,[a] David J. Hardee,[b] Timothy R. Hodges, [b] Wei Gong, [b] Eric A. Voight,*[b] and Huw M.L Davies*[a]

[a.] *Department of Chemistry, Emory University, 1515 Dickey Drive, Atlanta GA 30322, United States. *hmdavie@emory.edu*

[b.] *Drug Discovery Science and Technology, AbbVie, 1 North Waukegan Rd., North Chicago IL, 60064, United States. *eric.a.voight@abbvie.com*

For full text See DOI: 10.1039/x0xx00000x

Supporting Information:

Table of Contents

1. ***General Considerations*……………………………………………………………S1**
2. ***Preparation of Substrates and Starting Materials*……………………………S2-S11**
3. ***Procedures for the Cyclopropanation of Aza-heterocyclic Substrates*……S11-S14**
4. ***Characterization of Synthesized Compounds*………………………………S14-S38**
5. ***Determination of enantioselectivity*…………………………………………S38-S116**
6. ***NMR Spectra*………………………………………………………………S117-S197**
7. ***Crystallographic Data*……………………………………………………S198-S237**
8. ***References*………………………………………………………………S237-S238**

CAUTION: Diazo compounds are high energy compounds and need to be treated with respect. Even though we experienced no energetic decomposition in this work, care should be taken in handling large quantities of diazo compounds. Large scale reactions should be conducted behind a blast shield. For a more complete analysis of the risks associated with diazo compounds see the recent review by Bull *et. al.*[1]

1. General Considerations

All experiments were carried out in oven-dried glassware under argon atmosphere unless otherwise stated. Flash column chromatography was performed on silica gel. 4Å molecular sieves were activated under vacuum at 300 °C for 4 h. After time elapsed, the flask was cooled to 60 °C under inert nitrogen atmosphere and stored in a 140 °C oven for future use. All solvents were distilled using a short-path distillation system and stored over 4Å molecular sieves under argon atmosphere. Unless otherwise noted, all other reagents were obtained from commercial sources (Sigma Aldrich, Fisher, TCI Chemicals, AK Scientific, Combi Blocks, Oakwood Chemicals) and used as received without purification. ^{1}H, ^{13}C, and ^{19}F NMR spectra were recorded at either 400 MHz (^{13}C at 100 MHz) on Bruker 400 spectrometer or 600 MHz (^{13}C at 151 MHz) on INOVA 600 or Bruker 600 spectrometer. NMR spectra were run in solutions of deuterated chloroform ($CDCl_3$) with residual chloroform taken as an internal standard (7.26 ppm for ^{1}H, and 77.16 ppm for ^{13}C), and were reported in parts per million (ppm). The abbreviations for multiplicity are as follows: s = singlet, d = doublet, t = triplet, q = quartet, p = pentet, m = multiplet, dd = doublet of doublet, etc. Coupling constants (J values) are obtained from the spectra. Thin layer chromatography was performed on aluminum-back silica gel plates with UV light and cerium aluminum molybdate (CAM) or permanganate ($KMnO_4$) stain to visualize. Mass spectra were taken on a Thermo Finnigan LTQ-FTMS spectrometer with APCI, ESI or NSI. Melting points (mp) were measured in open capillary tubes with a Mel-Temp Electrothermal melting points apparatus and are uncorrected. IR spectra were collected on a Nicolet iS10 FT-IR spectrometer from Thermo Scientific and reported in unit of cm^{-1}. Enantiomeric excess (% ee) data were obtained on a Varian Prostar chiral HPLC instrument, an Agilent 1100 HPLC, or an Agilent 1290 Infinity UHPLC, eluting the purified products using a mixed solution of HPLC-grade 2-propanol (*i*-PrOH) and *n*-hexane.

Experimental Procedures

2. Preparation of Substrates:

2.1 Preparation of known substrates.

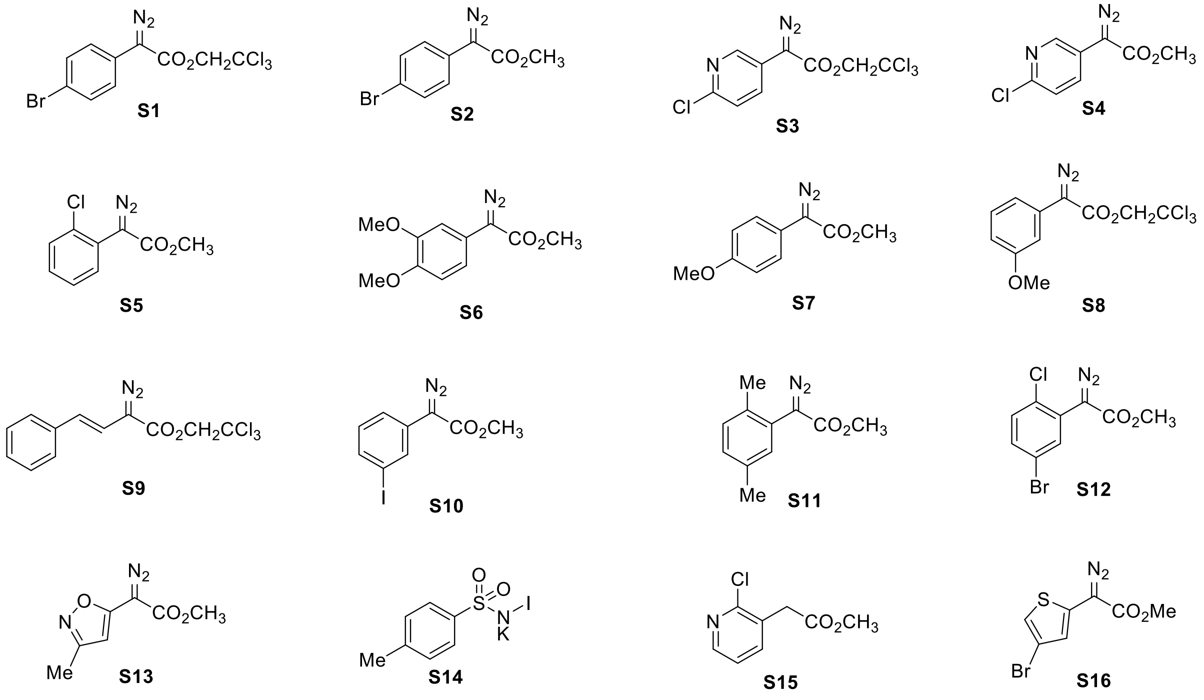

Figure S1: ***Known substrate starting materials***

Diazo compounds **s1-4** were prepared according to the established literature and matched the reported spectra.[2]
Diazo compounds **S5-7,** were prepared according to the established literature and matched the reported spectra.[3]
Diazo compounds **S8-9** were prepared according to the established literature and matched the reported spectra.[4]
Diazo compound **S10** was prepared according to the established literature and matched the reported spectra.[5]
Diazo compound **S11** was prepared according to the established literature and matched the reported spectra.[6]
Diazo compound **S12** was prepared according to the established literature and matched the reported spectra.[7]
Diazo compound **S13** was prepared according to the established literature and matched the reported spectra.[8]
Compound **s14** matched the spectra reported in the literature. [9]
Compound **s15** matched the spectra reported in the literature. [10]
Compound **s16** matched the spectra reported in the literature.[11]

2.2 Preparation of novel substrates:

2.2.1 Preparation of (*R*)-4,4-dimethyl-2-oxotetrahydrofuran-3-yl 2-(4-bromophenyl)-2-diazoacetate (S17).

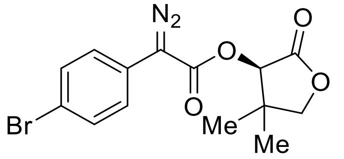

2.2.1.1 Esterification towards (*R*)-4,4-dimethyl-2-oxotetrahydrofuran-3-yl 2-(4-bromophenyl)acetate.

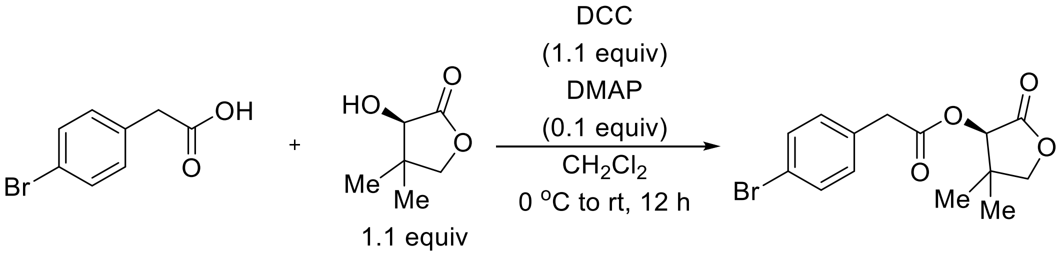

4-Bromophenylacetic acid (5.00 g, 23.3 mmol), *N,N*-dimethyl 4-aminopyridine (DMAP, 0.1 equiv, 284mg, 2.33 mmol), and (*R*)-3-hydroxy-4,4-dimethyldihydrofuran-2(*3H*)-one (*R*-pantolactone, 1.1 equiv, 3.33g, 25.6mmol) were added to a flame-dried round bottom flask. The reagents were dissolved in 100 mL CH_2Cl_2 (DCM) and the solution was cooled to 0 °C. A solution of *N,N'*-dicyclohexylcarbodiimide (DCC, 1.1 equiv, 5.28g, 25.6mmol) in CH_2Cl_2 (30 mL) was added to the reaction mixture via syringe over 5 minutes (min). The reaction mixture was removed from the ice bath allowed to stir overnight at room temperature. The reaction mixture was filtered by vacuum filtration over a pad of celite and washed with diethyl ether (Et_2O, 100 mL). The filtrate was concentrated and purified by flash column chromatography (0-5% EtOAc/hexanes over 30CV on isolera) to give pure (*R*)-4,4-dimethyl-2-oxotetrahydrofuran-3-yl 2-(4-bromophenyl)acetate as off-white needlelike crystals (70% yield, 5.3 g, 16 mmol) after aggregation and evaporation of appropriate fractions.

2.2.1.2 Diazo transfer to form (*R*)-4,4-dimethyl-2-oxotetrahydrofuran-3-yl 2-(4-bromophenyl)-2-diazoacetate.

(*R*)-4,4-dimethyl-2-oxotetrahydrofuran-3-yl 2-(4-bromophenyl)acetate (2.00 g, 6.11 mmol) and 4-acetamidobenzenesulfonyl azide (*p*-ABSA, 1.3 equiv, 1.91 g, 7.95 mmol) were added to a flame-dried round bottom flask and dissolved in acetonitrile (MeCN, 50mL) at 0 ℃ in an ice-bath. 1,8-diazabicyclo[5.4.0]undec-7-ene (DBU, 1.0 equiv, 0.921 mL) was added dropwise to the cooled stirring solution causing it to change from a clear-colorless solution to a deep orange solution. The reaction was left to stir overnight before quenching with saturated ammonium chloride solution (NH_4Cl in H_2O, 50mL). The aqueous and organic layers were separated, and the aqueous layer was extracted with Et_2O (2 X 25 mL). The combined organic layers were dried over anhydrous sodium sulfate (Na_2SO_4) before loading onto silica. The diazo -impregnated silica was then purified by flash column chromatography (100 g silica cartridge, 0% EtOAc/hexanes 3 CV, 0-20% EtOAc/Hexanes for 30 CV, 20% EtOAc/hexanes for 5 CV) to afford (*R*)-4,4-dimethyl-2-oxotetrahydrofuran-3-yl 2-(4-bromophenyl)-2-diazoacetate as a powdery bright orange solid (**S17,** 60% yield, 1.30 g, 3.68 mmol) after aggregation and evaporation of appropriate fractions.

2.2.2 Preparation of (*R*)-4,4-dimethyl-2-oxotetrahydrofuran-3-yl 2-diazo-2-(2-methoxy-5-methylphenyl)acetate (S18)

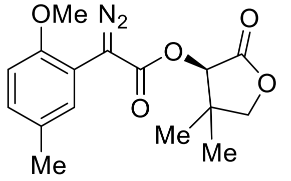

2.2.2.1 Friedel-Crafts acylation towards methyl 2-(2-methoxy-5-methylphenyl)-2-oxoacetate.

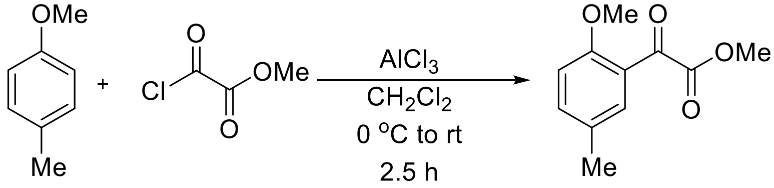

A solution of 1-methoxy-4-methylbenzene (1.0 equiv, 10.0 g, 10.3 mL, 81.9 mmol) and methyl 2-chloro-2-oxoacetate (1.5 equiv, 15.0 g, 11.3 mL, 122.8 mmol) in CH_2Cl_2 (50 mL) was added dropwise to a stirred suspension of aluminium chloride ($AlCl_3$, 1.0 equiv, 10.9 g, 81.85 mmol) in CH_2Cl_2 (150 mL) in a flame-dried round-bottom flask under an inert nitrogen atmosphere. The temperature maintained below 5 °C throughout addition. When the addition was complete, the deep purple mixture was stirred at 25 °C for 2.5 h and then poured onto 100 g of ice. The aqueous layer was washed once with CH_2Cl_2 (100 mL). The combined organic extracts were washed with 3 M HCl (200 mL), 1 M HCl (200 mL), DI water (200 mL), and

saturated NaCl (200 mL). Then the organic layer was filtered over a plug of basic alumina (25 g) and dried over sodium sulfate (Na_2SO_4) then organic solvent was concentrated to yield a yellow liquid. The crude product was then purified by flash chromatography (0-5% Et_2O/hexane over 25 CV) and the major peak containing fractions were aggregated and evaporated to yield the title product as a light yellow oil (93% yield, 15.9 g, 76.4 mmol).

2.2.2.2 Hydrolysis to afford 2-(2-methoxy-5-methylphenyl)-2-oxoacetic acid

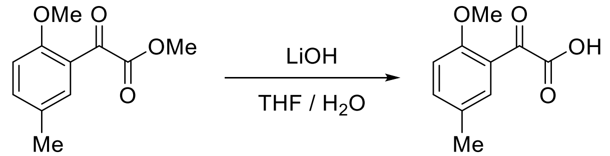

Lithium hydroxide (1.06 g, 44.3 mmol) was added to a solution of methyl 2-(2-methoxy-5-methylphenyl)-2-oxoacetate (4.61 g, 22.1 mmol) in tetrahydrofuran (30 mL) and water (15 mL) at room temperature. After stirring for 2 h the reaction was acidified with 1M HCl and extracted with EtOAc. The organic phase was washed with brine, dried with $MgSO_4$, filtered, and concentrated under reduced pressure to give the title compound (4.03 g, 20.8 mmol, 94% yield) as a gray solid without further purification. 1H NMR (600 MHz, DMSO-d6) δ 7.55 – 7.48 (m, 2H), 7.15 (d, J = 8.5 Hz, 1H), 3.81 (s, 3H), 2.29 (t, J = 0.7 Hz, 3H); MS(APCI+) m/z 195.6 (M+H)+. The material was used in the next step without further characterization.

2.2.2.3 Esterification towards (*R*)-4,4-dimethyl-2-oxotetrahydrofuran-3-yl 2-(2-methoxy-5-methylphenyl)-2-oxoacetate.

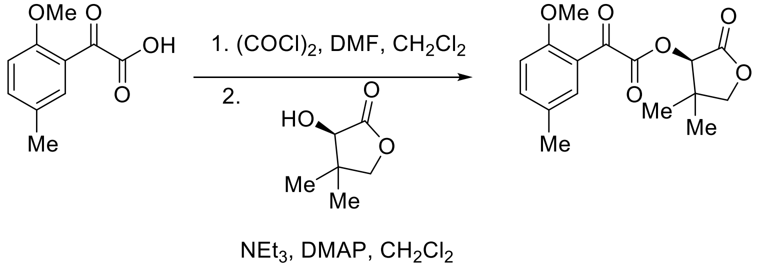

Oxalyl chloride (8.66 mL, 99 mmol) was added dropwise to a mixture of 2-(2-methoxy-5-methylphenyl)-2-oxoacetic acid (9.6 g, 49.4 mmol) and DMF (0.038 mL, 0.494 mmol) in CH_2Cl_2 (100 mL) at 0 °C. The reaction was slowly warmed to room temperature and stirred for 16 h before being concentrated under reduced pressure. The resulting residue was then taken up in CH_2Cl_2 (100 mL) and cooled in an ice bath. Triethylamine (TEA, 17.23 mL, 124 mmol), DMAP (0.060 g, 0.494 mmol), and *R*-pantolactone (9.65 g, 74.2 mmol, CombiBlocks) were then sequentially added before warming the mixture to room temperature. After stirring for 2 h the reaction was washed with 1M HCl, saturated $NaHCO_3$, and brine. The organic phase was then dried with $MgSO_4$, filtered, and concentrated under reduced pressure. The crude residue was purified by flash chromatography (ISCO Combiflash, 0-50% EtOAc/heptanes,120 g Redisep gold silica column) to give the title compound (13.8 g, 45.1 mmol, 91% yield) as a light yellow oil after aggregation and evaporation of appropriate fractions. 1H NMR (500 MHz, DMSO-d6) δ 7.62 – 7.55 (m, 2H), 7.19 (d, J = 8.5 Hz, 1H), 5.82 (s, 1H), 4.22 – 4.17 (m, 1H), 4.12 (d, J = 8.6 Hz, 1H), 3.84 (s, 3H), 2.31 (d, J = 0.8 Hz, 3H), 1.21 (s, 3H), 0.99 (s, 3H); MS(APCI+) m/z 307.3 (M+H)+. The material was used in the next step without further characterization.

2.2.2.4 One-pot diazotization of (*R*)-4,4-dimethyl-2-oxotetrahydrofuran-3-yl 2-(2-methoxy-5-methylphenyl)-2-oxoacetate.

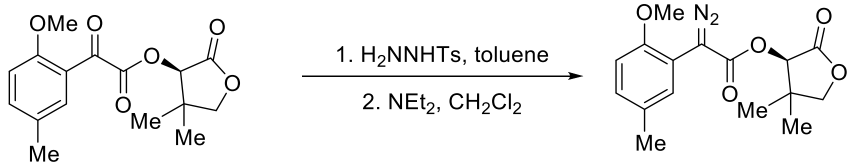

A mixture of (R)-4,4-dimethyl-2-oxotetrahydrofuran-3-yl 2-(2-methoxy-5-methylphenyl)-2-oxoacetate (8.53 g, 27.8 mmol) and 4-methylbenzenesulfonohydrazide (5.19 g, 27.8 mmol, Aldrich) in toluene (56 mL) was heated to reflux with a Dean-Stark trap. After 16 h the reaction was concentrated under reduced pressure and CH_2Cl_2 (56 mL) and TEA (5.82 mL, 41.8 mmol) were added to the resulting residue. After stirring at room temperature for 16 h the reaction was washed with saturated $NaHCO_3$ and brine, dried with $MgSO_4$, filtered, and concentrated under reduced pressure. The crude residue was purified by flash chromatography (ISCO Combiflash, 0-40% EtOAc/heptanes, 120 g Redisep gold silica column) to yield (*R*)-4,4-dimethyl-2-oxotetrahydrofuran-3-yl 2-diazo-2-(2-methoxy-5-methylphenyl)acetate (**S18,** 7.3 g, 23 mmol, 82% yield) as a yellow solid after aggregation and evaporation of appropriate fractions.

2.2.3 Preparation of methyl 2-diazo-2-(2-methoxy-5-methylphenyl)acetate (S19).

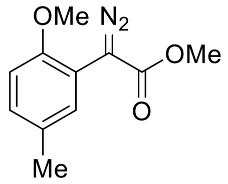

2.2.3.1 Hydrazine condensation towards methyl (*E*/*Z*)-2-hydrazineylidene-2-(2-methoxy-5-methylphenyl) acetate.

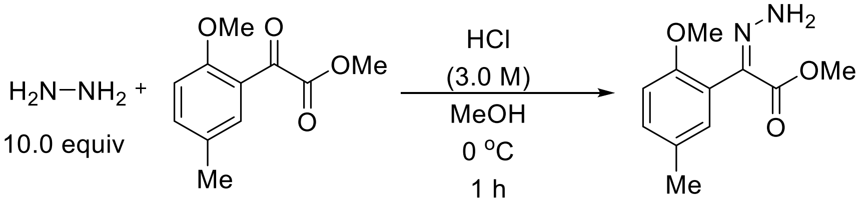

Hydrazine hydrate (50 wt% in H_2O, 10.0 equiv, 24 mL, 384 mmol) was dissolved in methanol (100 mL) in a round-bottom flask and placed into an ice bath. 3M HCl (9.0 equiv, 120 mL, 346 mmol) was added dropwise to the stirring cold solution. After the addition was complete, methyl 2-(2'-methoxy-5'-methylphenyl)-2-oxoacetate (1.0 equiv, 8.00 g, 38.4 mmol) dissolved in methanol (30 mL) was added dropwise to the stirring solution. The solution was left to stir for 1.5 h. Reaction completion was determined by the presence of only two CAM stain active spots on TLC. If the reaction is left to run too long, a yellow precipitate is generated, possibly the azine dimer. This byproduct has the same rf as the starting material by TLC and thus disappearance of the starting material cannot be used to determine reaction completion. The reaction was quenched with a saturated sodium bicarbonate ($NaHCO_3$) solution (150 mL) and left to stir and quench overnight. The reaction mixture was concentrated via rotovap to remove methanol. The solution was then extracted with EtOAc (2 X 100 mL) and the organic layer was washed with DI H_2O (100 mL) and saturated NaCl solution (100 mL) before drying over Na_2SO_4 and concentrating in vacuo. Hydrazone was purified by flash column chromatography (0-25% EtOAc/hec over 8 CV, 25% EtOAc/hex for 6 CV, 25-45% EtOAc/hexanes over 6 CV, 45% EtOAc/hex for 10 CV, then 65% EtOAC/hex- 85% EtOAc/Hex for 10 CV). Both the *E* and *Z* isomers of the hydrazone product were isolated as separate peaks CAM active peaks and combined. The *E* and *Z* isomers may be isolated separately as white powders in their pure form, either isomer or a mixture of the two is suitable for the subsequent diazotization. Product containing fractions were concentrated to afford the title compound as a clear colorless oil (89% yield, 7.56 g, 34 mmol).

2.2.3.2 Iodamine-T (TsNIK) oxidation of methyl (*E*/*Z*)-2-hydrazineylidene-2-(2-methoxy-5-methylphenyl)acetate.

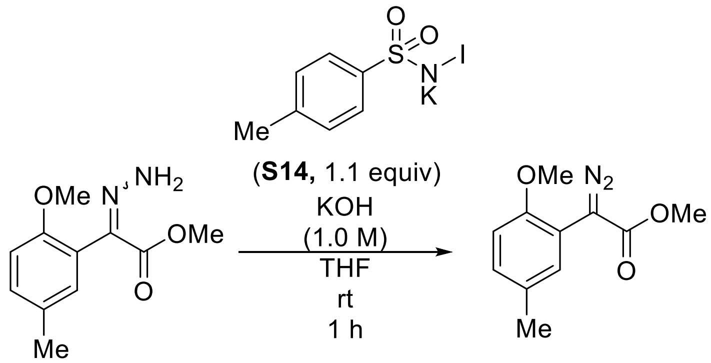

Mixture of E and Z isomers

Iodamine-T (TsNIK, **S14**, 1.1 equiv, 5.19 g, 15.47 mmol) was suspended in a solution of methyl (*E*/*Z*)-2-hydrazineylidene-2-(2-methoxy-5-methylphenyl)acetate (1.0 equiv, 3.13 g, 14.06 mmol) in THF (20 mL). Aqueous 1.0 M KOH (1.1 equiv, 15 mL, 15.47 mmol) was slowly added to the suspension, causing the solution to change in coloration from yellow to deep red. After stirring for 1 h at room temperature, the reaction solution was poured into aqueous KOH (1.0 M, 20 mL) and extracted with Et_2O (2 X 20 mL). The organic layer was washed with KOH (1.0 M, 20 mL), DI H_2O (20 mL) and saturated NaCl solution (40 mL) before being dried over Na_2SO_4. The organic layer was concentrated via rotovap and purified by flash column

chromatography (1% EtOAc/hexanes over 24 CV) to afford methyl 2-diazo-2-(2-methoxy-5-methylphenyl)acetate (**S19**) as a bright orange, crystalline powder (80% yield, 2.5 g, 11.3 mmol) after aggregation and evaporation of appropriate fractions..

2.2.4 Synthesis of methyl 2-(2-chloropyridin-3-yl)-2-diazoacetate (S20).

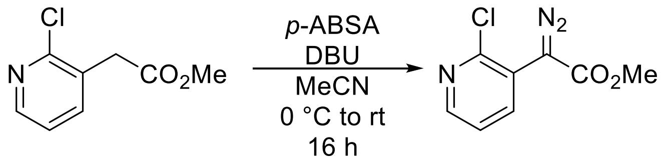

Methyl 2-(2-chloropyridin-3-yl)acetate (**S15,** 1.0 equiv, 5.39 g, 29.1 mmol) and *p*-ABSA (1.2 equiv, 8.38 g, 34.9 mmol) were added to a flame-dried 250 mL round-bottom flask under an inert nitrogen atmosphere. They were dissolved in dry acetonitrile (MeCN, 150 mL) and cooled to 0 ˚C in an ice-bath. Then DBU (1.2 equiv, 5.31 g, 5.20 mL, 10.9 mmol) was added dropwise to the stirring solution which slowly became deep yellow. The reaction was allowed to warm to room temperature over 18 h. Reaction was then quenched with saturated NH_4Cl (100 mL) and the organic layer was separated. The aqueous layer was extracted with CH_2Cl_2 (2 X 50 mL) and organic extracts were combined, dried over Na_2SO_4, and dry-loaded onto silica (5 g). The product was then purified by flash column chromatography (0-50% EtOAc/hexanes). Yellow fractions were combined and evaporated to yield methyl 2-(2-chloropyridin-3-yl)-2-diazoacetate (**S20**) as a bright yellow fluffy solid (96% yield, 5.9 g, 27.9 mmol).

2.2.5 Synthesis of methyl 2-diazo-2-(2-ethoxy-5-methylphenyl)acetate (S21).

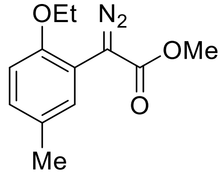

2.2.5.1 Friedel-Crafts acylation towards methyl 2-(2-ethoxy-5-methylphenyl)-2-oxoacetate.

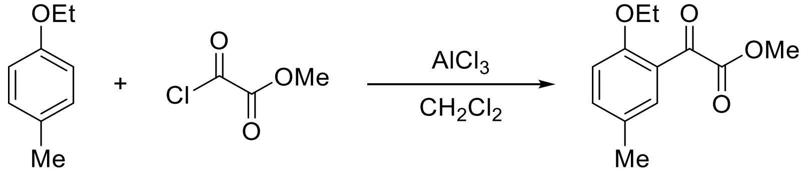

1-Ethoxy-4-methylbenzene (1.35 mL, 9.6 mmol) was added dropwise to a suspension of $AlCl_3$ (1.60 g, 12.0 mmol) in CH_2Cl_2 (38.5 mL) at 0 °C. After 5 min, methyl 2-chloro-2-oxoacetate (1.106 mL, 12.02 mmol, Aldrich) was added dropwise and the reaction was warmed to room temperature. After stirring for 16 h the reaction was poured into 250 mL of 1M HCl and extracted with CH_2Cl_2. The organic phase was washed with 1M HCl and brine, dried with $MgSO_4$ and concentrated under reduced pressure. The crude residue was then purified by flash chromatography (ISCO Combiflash, 0-30% EtOAc/heptanes, 80 g Redisep gold silica column) to yield the title compound (1.42 g, 6.39 mmol, 66.4 % yield) as a light yellow oil after aggregation and evaporation of appropriate fractions. 1H NMR (400 MHz, $CDCl_3$) δ 7.68 (dd, J = 2.4, 1.0 Hz, 1H), 7.40 7.30 (m, 1H), 6.86 (d, J = 8.5 Hz, 1H), 4.07 (q, J = 7.0 Hz, 2H), 3.91 (s, 3H), 2.32 (d, J = 0.7 Hz, 3H), 1.39 (t, J = 7.0 Hz, 3H); MS(APCI+) *m/z* 223.4 $(M+H)^+$. The material was used in the next step without further characterization.

2.2.5.2 One-pot diazotization of (methyl 2-(2-ethoxy-5-methylphenyl)-2-oxoacetate.

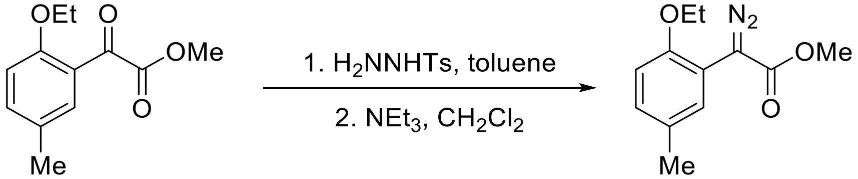

A mixture of methyl 2-(2-ethoxy-5-methylphenyl)-2-oxoacetate (1.29 g, 5.80 mmol) and 4-methylbenzenesulfonohydrazide (1.08 g, 5.80 mmol) in toluene (14.5 mL) was heated at reflux with a Dean-Stark trap for 16 h. The reaction was then concentrated under reduced pressure and the resulting residue was taken up in CH_2Cl_2 (14.5 mL) and cooled in an ice bath. TEA (1.214 mL, 8.71 mmol) was added in one portion and after 5 minthe reaction was warmed to room temperature. After stirring for 16 h the reaction was washed with sat. $NaHCO_3$ dried with $MgSO_4$, and concentrated under reduced pressure. The crude residue was then purified by flash chromatography (ISCO Combiflash, 0-20% EtOAc/heptanes, 40 g Redisep gold silica column) to yield the title compound (**S21,** 1.08 g, 4.61 mmol, 79 % yield) as an orange oil that crystallized upon standing after aggregation and evaporation of appropriate fractions..

2.2.6 Synthesis of methyl 2-diazo-2-(2-fluoro-5-methylphenyl)acetate (S22).

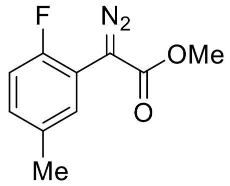

2.2.6.1 Synthesis of methyl 2-(2-fluoro-5-methylphenyl)-2-oxoacetate

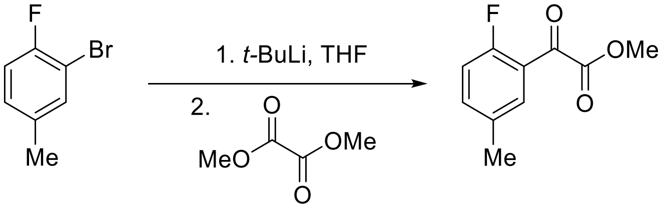

tert-Butyllithium (*t*-BuLi, 13.4 mL, 22.8 mmol, 1.7 M in pentane) was added dropwise to a solution of 2-bromo-1-fluoro-4-methylbenzene (1.43 mL, 11.4 mmol, Aldrich) in THF (57 mL) at -78 °C. After 20 mina solution of dimethyl oxalate (2.69 g, 22.75 mmol, Aldrich) in THF (3 mL) was added in one portion and the reaction was warmed to 0 °C. After stirring for 16 h the reaction was quenched with DI H_2O and extracted with EtOAc. The organic phase was washed with brine, dried with Na_2SO_4, filtered, and concentrated under reduced pressure. The crude material was purified by flash chromatography (ISCO Combiflash, 0-50% EtOAc/heptanes, 40 g Redisep gold silica column) to give the title compound (1.21 g, 6.17 mmol, 54.2 % yield) as a yellow oil after aggregation and evaporation of appropriate fractions.. ^{1}H NMR (600 MHz, $CDCl_3$) δ 7.70 (ddd, J = 6.7, 2.4, 0.9 Hz, 1H), 7.43 (dddd, J = 8.5, 5.0, 2.4, 0.7 Hz, 1H), 7.05 (dd, J = 10.5, 8.5 Hz, 1H), 3.96 (s, 3H), 2.42 – 2.33 (m, 3H); MS(APCI+) *m/z* 197.5 $(M+H)^+$. The material was used in the next step without further characterization.

2.2.6.2 Diazotization of methyl 2-(2-fluoro-5-methylphenyl)-2-oxoacetate.

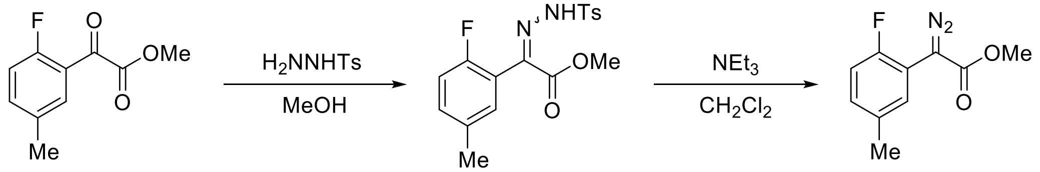

Methyl 2-(2-fluoro-5-methylphenyl)-2-oxoacetate (1.28 g, 6.52 mmol) was added to a slurry of 4-methylbenzenesulfonhydrazide (1.28 g, 6.85 mmol) in methanol (6 mL) at room temperature to give a yellow solution. After stirring for 16 h at room temperature a white precipitate formed that was collected by filtration and washed with methyl-*tert*-butyl ether (MTBE) to give 819 mg of intermediate hydrazone. The mother liquor was concentrated under reduced pressure and the resulting solid was triturated with MTBE to yield an additional 700 mg of hydrazone as a white solid. This material was used directly in the next step without further purification. CH_2Cl_2 (10 mL) and TEA (0.627 mL, 4.50 mmol) were added to the first 819 mg batch of white solid. After stirring at room temperature for 16 h the reaction was concentrated under reduced pressure. The crude material was purified by flash chromatography (ISCO Combiflash, 0-20% EtOAc / heptanes, 24 g Redisep gold silica column) to yield the title compound as a yellow solid (**S22,** 406 mg, 1.950 mmol, 87 % yield) after aggregation and evaporation of appropriate fractions.

2.2.7 Synthesis of methyl 2-diazo-2-(2-fluoro-5-methoxyphenyl)acetate (S23).

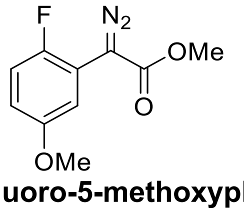

2.2.7.1 Esterification of methyl 2-(2-fluoro-5-methoxyphenyl)acetate

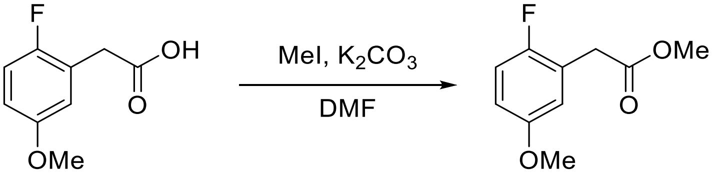

Methyl iodide (0.82 mL, 13.0 mmol) was added to a mixture of 2-(2-fluoro-5-methoxyphenyl)acetic acid (2 g, 10.86 mmol, Astatech) and potassium carbonate (K_2CO_3, 1.95 g, 14.1 mmol) in DMF (11 mL) at room temperature. After stirring for 16 h the reaction was diluted with EtOAc and washed twice with DI H_2O and once with saturated NaCl solution. The organic phase was dried with $MgSO_4$ filtered, and concentrated under reduced pressure to give methyl 2-(2-fluoro-5-methoxyphenyl)acetate (1.66 g, 8.38 mmol, 77 % yield) as a light yellow oil without further purification. ^{1}H NMR (500 MHz, $CDCl_3$) δ 7.01 – 6.95 (m, 1H), 6.80 – 6.74 (m, 2H), 3.77 (s, 3H), 3.71 (s, 3H), 3.64 (d, J = 1.3 Hz, 2H). The material was used in the next step without further characterization.

2.2.7.2 Diazo transfer to form methyl 2-diazo-2-(2-fluoro-5-methoxyphenyl)acetate.

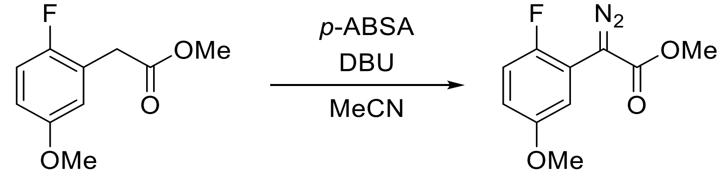

DBU (1.27 mL, 8.40 mmol) was added to a solution of methyl 2-(2-fluoro-5-methoxyphenyl)acetate (1.19 g, 6.00 mmol) and *p*-ABSA (1.73 g, 7.20 mmol, Aldrich) in MeCN (18 mL) at room temperature. After stirring for 16 h the reaction was diluted with EtOAc and washed twice with DI H_2O and once with brine. The organic phase was dried with $MgSO_4$, filtered, and concentrated under reduced pressure. The crude residue was purified by flash column chromatography (ISCO Combiflash, 0-40% EtOAc / heptanes, 40 g Redisep gold silica column) to yield the title compound (**S23,** 977 mg, 4.36 mmol, 72.6 % yield) after evaporation of appropriate fractions as a bright yellow solid.

2.2.9 Synthesis of substituted vinyl-heterocycles

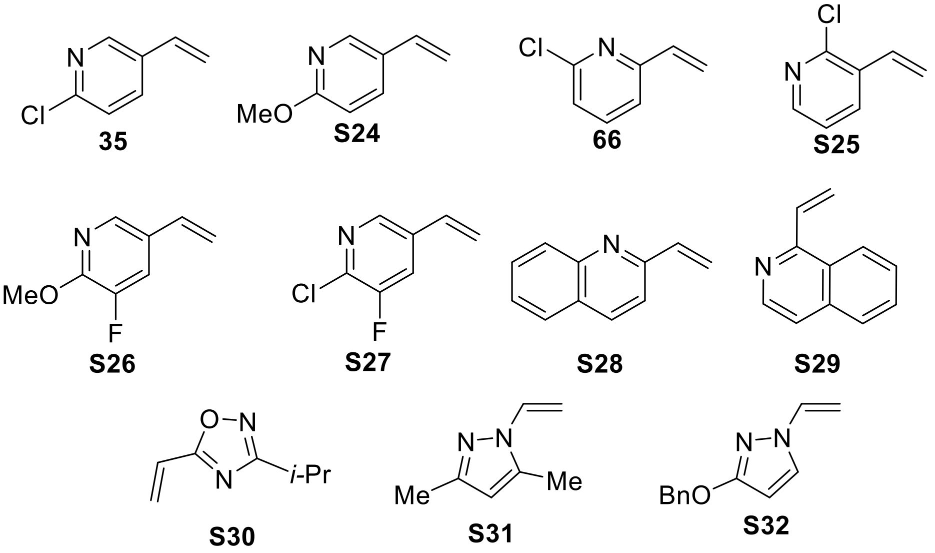

Figure S2: Vinyl-heteroaryl substrates for cyclopropanation of aza-heterocycles. Compounds **35, 66, S24-S25** were synthesized using general procedure A. Compounds **S26-S29** were synthesized using general procedure B. Compounds **S30-S32** were synthesized according to alternative procedures.

2.2.8.1 General procedure A, Wittig olefination of pyridyl-aldehydes.

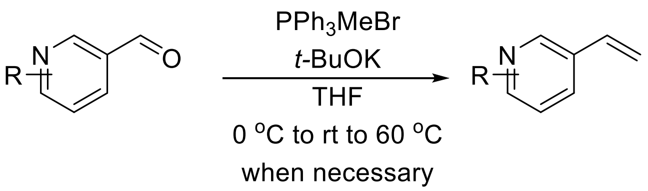

Reaction may be conducted on large scale, up to 140 mmol, although slightly lower yields may be observed. To 0 °C, stirred slurry of methyltriphenylphosphonium bromide (1.2 equiv) under an inert nitrogen atmosphere in tetrahydrofuran (150 mL) was added potassium *tert*-butoxide dissolved in 30 mL THF ((*t*-BuOK, 1.2 equiv) portionwise over a 5 minute period to produce a yellow ylide slurry. After 30 min, pyridyl aldehyde (1.0 equiv) was added slowly to produce a colored slurry (color ranges from brown to blue depending on pyridyl substitution). TLC was conducted every hour to monitor reaction progress (5% EtOAc in hexanes) warming to rt or heating to 60 ˚C if reaction was incomplete after 2 h. After the reaction had stopped the reaction mixture was treated with saturated aqueous ammonium chloride (160 mL) and a majority of the THF was

removed in vacuo (concentrated via rotovap). The resulting mixture (often a brown liquid) was washed with ethyl acetate, the combined organic layers washed with saturated aqueous brine and stirred over activated charcoal for 1hr to remove colored impurities. The mixture was then filtered over Celite, dried over Na_2SO_4 and concentrated in vacuo. The resulting semi-solid/oil was stirred overnight in pentanes (150 mL) to precipitate phosphine byproduct. Then the solution was filtered through a silica plug (~50 g) and the solids washed with an additional portion of 2:1 Et_2O/pentane (500 mL) until olefin spots (as confirmed by TLC (5% EtOAc/hexanes) developed with permanganate stain) no longer eluted from the plug. The combined filtrates were concentrated in vacuo and purified by flash column chromatography (0-5% Et_2O/hexanes over 40 CV) and product containing fractions were combined and evaporated to afford the vinyl-pyridine as a colorless or yellow oil (40-65% yield). Products **35, 66, S24,** and **S25** were synthesized via this method.

2.2.8.2 General procedure B, Suzuki-coupling towards vinyl-heterocycles.

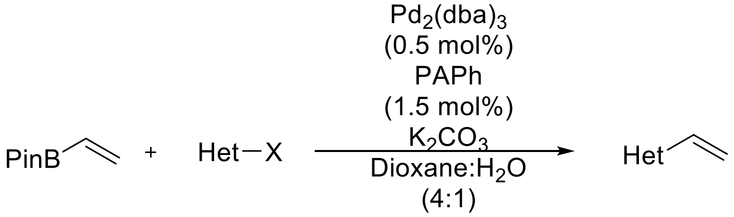

Reaction may be run on a variety of scales and is effective for heteroaryl iodides, bromides, and chlorides although higher catalyst and ligand loading (2 mol % Pd/ 5 mol % PAPh) was most effective for heteroaryl-chlorides. Heteroaryl-halide (1.0 equiv), 1,3,5,7-tetramethyl-6-phenyl-2,4,8-trioxa-6-phosphaadamantane (PAPh, 1.5 mol %), and potassium carbonate (K_3CO_3, 2.5 equiv) were combined in a round-bottom flask containing a stir bar and charged with a dioxane:water (4:1 v:v) solution. The mixture was sparged with nitrogen for 10 minbefore adding tris(dibenzylideneacetone)dipalladium(0) ($Pd_2(dba)_3$, 0.5 mol %). After the solution had turned a deep red color, neat vinylboronic acid pinacol ester (1.5 equiv) was added dropwise. The reaction mixture was then heated overnight at 80 °C. Disappearance of starting heteroaryl-halide was monitored by TLC (1% EtOAc/hexanes) to determine reaction completion. The reaction mixture was decanted into a separatory funnel and DI water (20 mL) was added. The aqueous mixture was extracted with Et_2O (2 X 20 mL). Organic extracts were combined and washed with DI H_2O (1x 50 mL) and saturated NaCl solution (1x 50 mL). The organic layer was then filtered over celite and dried over Na_2SO_4 before concentrating in vacuo. The material was then purified via flash column chromatography (0-2% EtOAc / hexanes, 18 CV) and the pure product fractions (identified by permanganate stain) were combined to afford the desired vinyl-heterocycle (**S26-S29**, 47-96% yield).

2.2.9 Synthesis of 3-isopropyl-5-vinyl-1,2,4-oxadiazole (S30)

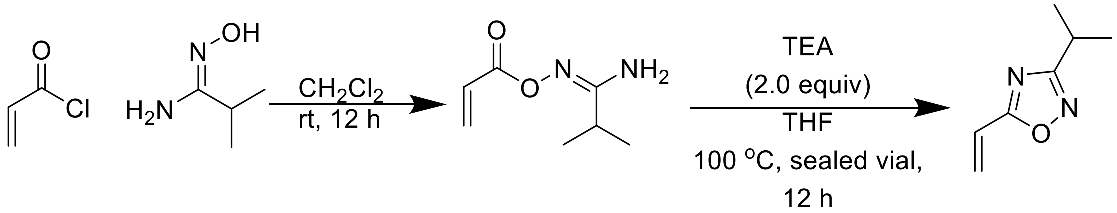

Acryloyl chloride (1.20 equiv, 1.06 g, 11.7 mmol) was added slowly to a solution of (*E*)-*N'*-hydroxyisobutyrimidamide (1.00 g, 9.79 mmol) in CH_2Cl_2 (8 mL) at rt. After 12 hr, the reaction mixture was neutralized using sat. $NaHCO_3$, extracted 3 X using CH_2Cl_2, dried over anhydrous Na_2SO_4, and concentrated to afford crude (*E*)-*N'*-(acryloyloxy)isobutyrimidamide (1.53 g, 9.78 mmol) as a white solid. Crude (*E*)-*N'*-(acryloyloxy)isobutyrimidamide (1.53 g, 9.78 mmol) and TEA (2.76 mL, 19.81 mmol) were then dissolved in THF (20 mL) in a sealed microwave vial. The reaction mixture was heated to 100 °C and stirred for 12 h and then cooled to rt. After the vial had cooled, the mixture was filtered over celite, washed with ether, and concentrated. The crude residue was purified by flash column chromatography (0% ether/hexanes for 3 CV, 0%-5% ether/hexanes over 15 CV, 5% ether/hexanes for 5 CV). The appropriate fractions (identified by permanganate stain) were concentrated via rotovap (with a bath temp of 23 °C) to afford 3-isopropyl-5-vinyl-1,2,4-oxadiazole (**S30,** 550mg, 3.98 mmol, 40% yield) as a clear colorless liquid.

2.2.10 Synthesis of 3,5-dimethyl-1-vinyl-*1H*-pyrazole (S31)

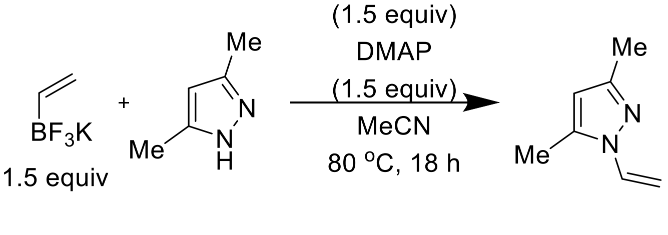

This product was synthesized via Chan-Lam coupling. $Cu(OAc)_2$ (1.5 equiv, 8.5 g, 47 mmol), DMAP (1.5 equiv, 5.7 g, 47 mmol), trifluoro(vinyl)-l4-borane potassium salt (1.5 equiv, 6.3 g, 47mmol), and 3,5-dimethyl-*1H*-pyrazole (1.0 equiv, 3.0 g, 31 mmol) were added to a 250 mL round-bottom flask and dissolved in MeCN (100 mL). The flask was sealed and heated to 80 °C under inert nitrogen atmosphere. After stirring for 18 h, the reaction was cooled to rt, diluted with Et_2O, and filtered off the solids by passing through a short silica plug. The filtrate was concentrated carefully via rotovap in order to not lose the volatile product. The resulting crude residue was purified by flash column chromatography (0-100% Et_2O/hexanes over 40 CV) and product containing fractions (identified by permanganate stain) were carefully concentrated to afford 3,5-dimethyl-1-vinyl-*1H*-pyrazole (**S31,** 1.8 g, 15 mmol, 47% yield) as a clear colorless liquid.

2.2.11 Synthesis of 3-(benzyloxy)-1-vinyl-*1H*-pyrazole (S32)

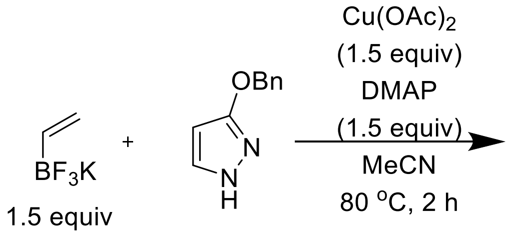

This product was synthesized via Chan-Lam coupling. Mixed DMAP (1.052 g, 8.61 mmol), $Cu(OAc)_2$ (1.564 g, 8.61 mmol), trifluoro(vinyl)-l4-borane potassium salt (1.153 g, 8.61 mmol), and 3-(benzyloxy)-*1H*-pyrazole (1 g, 5.74 mmol) in MeCN (14.4 mL). The mixture was heated at 80 °C for 2 h under ambient atmosphere. The mixture was cooled to rt, diluted with MTBE, mixed with celite, filtered, washed with additional MTBE, and loaded onto additional celite by removing the solvent under vacuum. The material was then purified by flash column chromatography (RediSep Rf Gold® Normal-Phase Silica, 24 g, 0-50% EtOAc/heptanes) and product containing fractions (identified by permanganate stain) were concentrated to afford 3-(benzyloxy)-1-vinyl-*1H*-pyrazole (**S32** 475 mg, 2.372 mmol, 41.3 % yield) as a clear colorless oil.

2.3 Catalyst preparation:

All catalysts were synthesized according to known procedures and used directly.

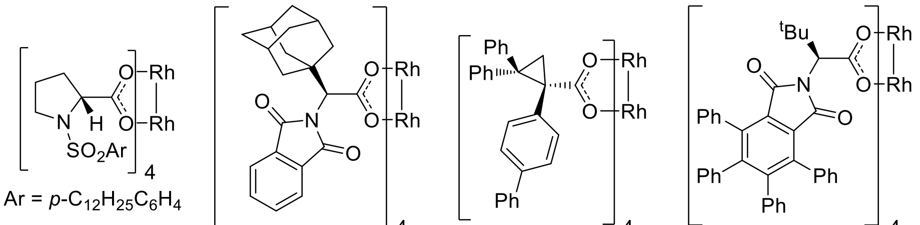

4 **Rh_2(*R*-DOSP)$_4$** · **5** **Rh_2(*S*-PTAD)$_4$** · **6** **Rh_2(*R*-p-Ph-TPCP)$_4$** · **7** **Rh_2(*S*-TPPTTL)$_4$**

Figure S3: Dirhodium tetracarboxylate catalysts used for cyclopropanation involving aza-heterocycles.
Rh_2(*R*-DOSP)$_4$ **4** was prepared using the procedure reported in the literature.[12]
Rh_2(*S*-PTAD)$_4$ **5** was prepared using the procedure reported in the literature.[13]
Rh_2(*R*-*p*-Ph-TPCP)$_4$ **6** was prepared using the procedure reported in the literature.[14]
Rh_2(*R*-TPPTTL)$_4$ and Rh_2(*S*-TPPTTL)$_4$ **7** were prepared using the procedure reported in the literature.[15]

3. Procedures for cyclopropanation involving aza-heterocycles.

3.1 General procedure for cyclopropanation of vinyl-heterocycles with aryl-diazo-(*R*)-pantolactonates.

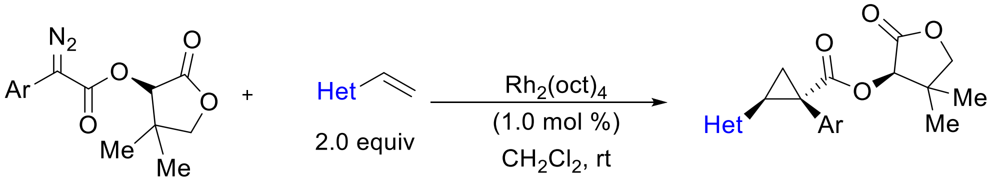

A 10 mL vial containing a stir bar was flame dried under vacuum along with a small round-bottom flask. Vessels were evacuated and purged with nitrogen 2 times to establish an inert atmosphere. Then catalytic rhodium octanoate dimer ($Rh_2(oct)_4$, 1.0 mol %, 1.6 mg, 2.0 μmol) was added to the vial. Solid aryl-diazo-(*R*)-pantolactonate (1.0 equiv, 0.20 mmol) was added to the flame dried round bottom flask. Then vacuum was reestablished on both the vial and the flask to further

remove air from the system. After 5 min, the system was flushed with nitrogen and vinyl-heterocycle (2.0 equiv, 0.40 mmol) was added to the vial via preweighed syringe along with 2 mL dry CH_2Cl_2 was added to the vial. The nitrogen line attached to the vial was then replaced by a balloon filled with argon. Diazo compound was dissolved in 3 mL dry CH_2Cl_2 under an inert atmosphere. The round bottom flask was swirled to ensure all diazo had dissolved before the solution was loaded into a syringe. The syringe was then inserted through the vial septum and the full contents were injected into the vial in one portion. The reaction was stirred at room temperature overnight under argon (at least 13 h). The reaction solution was subjected to TLC to determine reaction completion (30% EtOAc/hexanes). Crude reaction was concentrated via rotovap and asymmetric induction was determined by ^{1}H NMR of the crude reaction mixture. Purified by flash column chromatography (0% Et_2O/hexanes for 3CV, 0-100% Et_2O /hexanes over 30 CV, 100% Et_2O for 3CV). Fractions containing only product by TLC were aggregated and concentrated via rotovap. Products **8-16** were synthesized via this method.

3.2. General procedure for the additive free cyclopropanation of vinyl-heterocycles with non-*ortho*-substituted aryl/heteroaryl-diazoacetates.

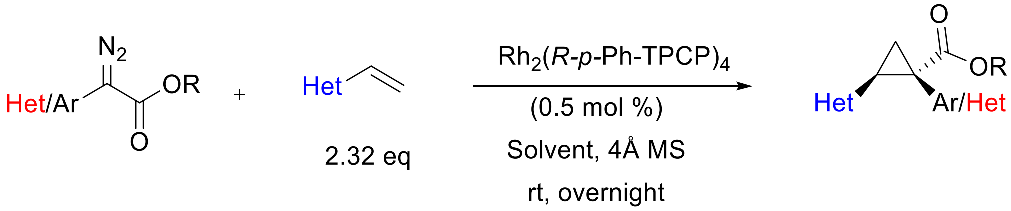

Compounds were prepared according to the established literature procedure for lab-scale cyclopropanation.[16] A 10 mL vial containing 4Å activated molecular sieves (0.5 g) and a stir bar was flame dried under vacuum along with a small round-bottom flask. Vessels were evacuated and purged with nitrogen 2 times to establish an inert atmosphere. Then catalytic Rh_2(*R*-*p*-Ph-TPCP)$_4$ (0.5 mol %, 1.8 mg, 0.0001 mmol) was added to the vial. Solid diazo compound (1.0 equiv, 0.20 mmol) was added to the flame dried round bottom flask. Then vacuum was reestablished on both the vial and the flask to further remove air from the system. After 5 min, the system was flushed with nitrogen and vinyl-heterocycle (2.32 equiv, 1.0 mmol) was added to the vial via preweighed syringe along with 2 mL dry $(MeO)_2CO$ or CH_2Cl_2. The nitrogen line attached to the vial was then replaced by a balloon filled with argon. Diazo compound was dissolved in 3 mL dry $(MeO)_2CO$ or CH_2Cl_2 under an inert atmosphere. The round bottom flask was swirled to ensure all diazo had dissolved before the solution was loaded into a syringe. The syringe was then inserted through the vial septum and the full contents were injected into the vial in one portion. The reaction was stirred overnight under argon (at least 13 h). The reaction solution was subjected to TLC to determine reaction completion (20% EtOAc/hexanes). After completion the solution was filtered through celite to remove molecular sieves before concentrating via rotovap. The crude concentrate was then directly purified by flash column chromatography (5% EtOAc/hexanes 3 CV, 5% EtOAc/hexanes to 30% EtOAc/hexanes 15 CV, 30% EtOAc/hexanes for 3-10 CV). Fractions containing only product by TLC were aggregated and concentrated via rotovap. Enantioselectivity was determined by chiral HPLC. Products **17-33** were synthesized via this method.

3.2.1 Synthesis of *Tert*-butyl 5-bromo-3-(1-diazo-2-oxo-2-(2,2,2-trichloroethoxy)ethyl)-*1H*-indole-1-carboxylate (34).

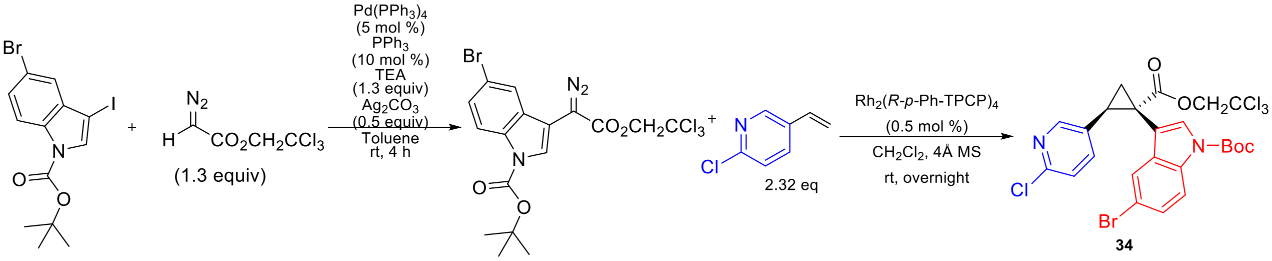

34

The diazo compound in question proved difficult to work with, but effective cyclopropanation was achieved by using the compound directly after a short column. Tetrakis(triphenylphosphine)palladium(0) (273.8 mg, 0.05 equiv, 236.9 μmol), triphenylphosphine (124.3 mg, 0.1 equiv, 473.9 μmol), *tert*-butyl 5-bromo-3-iodo-*1H*-indole-1-carboxylate (2.000 g, 1.0 equiv, 4.739 mmol), silver carbonate (653.3 mg, 0.5 equiv, 2.369 mmol) were added to a 100 mL flame-dried round bottom flask equipped with a magnetic stir-bar. The reagents were suspended in toluene (20 mL) under nitrogen, followed by addition of triethylamine (623.4 mg, 0.859 mL, 1.3 equiv, 6.160 mmol) and 2,2,2-trichloroethyl 2-diazoacetate (1.133 g, 1.1 equiv, 5.213 mmol). The resulting reaction was stirred at room temperature for 4 h and then filtered through a short path of silica gel, eluting with ethyl acetate. The volatile compounds were removed through reduced pressure and the residue was purified by column chromatography(1% -15% EtOAc in hexane) to give the desired product (2.259 g, 93% yield), ***tert*-butyl 5-bromo-3-(1-diazo-2-oxo-2-(2,2,2-trichloroethoxy)ethyl)-*1H*-indole-1-carboxylate,** as a bright orange solid after aggregation and evaporation of appropriate fractions. **^{1}H NMR** (400 MHz, $CDCl_3$) δ 8.11 (d, *J* = 88 Hz, 1H), 7.87 (s, 1H), 7.63 (d, *J* = 1.9 Hz, 1H), 7.46 (dd, *J* = 8.9, 1.9 Hz, 1H), 4.93 (s, 2H), 1.66 (s, 9H).**HRMS:** (+p APCI) Peak calculated for [$C_{17}H_{15}BrCl_3N_3O_4$+ minus $C_5H_9O_2$ minus N_2 plus 3H] 381.88095, found 381.88001. **IR**(neat): 2954, 2091, 1737, 1713, 1451, 1370, 1306, 1270, 1249, 1243, 1120, 1153, 1113, 1050, 1009, 897, 853, 841, 803, 778, 766, 717, 634, 615, 586, 573 cm^{-1}.

The product was used immediately in the subsequent cyclopropanation without further characterization. A 10 mL vial containing 4Å activated molecular sieves (0.5 g) and a stir bar was flame dried under vacuum along with a small round-bottom flask. Vessels were evacuated and purged with nitrogen 2 times to establish an inert atmosphere. Then catalytic Rh_2(*R*-*p*-Ph-TPCP)$_4$ (0.5 mol %, 1.8 mg, 0.0001 mmol) was added to the vial. Solid diazo compound (1.0 equiv, 100 mg, 0.20 mmol) was added to the flame dried round bottom flask. Then vacuum was reestablished on both the vial and the flask to further remove air from the system. After 5 min, the system was flushed with nitrogen and **35** (2.32 equiv, 0.46 mmol, 65 mg) was added to the vial via preweighed syringe along with 2 mL dry CH_2Cl_2. The nitrogen line attached to the vial was then replaced by a balloon filled with argon. Diazo compound was dissolved in 3 mL dry CH_2Cl_2 under an inert atmosphere. The round bottom flask was swirled to ensure all diazo had dissolved before the solution was loaded into a syringe. The syringe was then inserted through the vial septum and the full contents were injected into the vial in one portion. The reaction was stirred overnight under argon (at least 13 h). The reaction solution was subjected to TLC to determine reaction completion (20% EtOAc/hexanes). After completion the solution was filtered through celite to remove molecular sieves before concentrating via rotovap. The crude concentrate was then directly purified by flash column chromatography (5% EtOAc/hexanes 3 CV, 5% EtOAc/hexanes to 30% EtOAc/hexanes 15 CV, 30% EtOAc/hexanes for 3-10 CV). Fractions containing only product by TLC were aggregated and concentrated via rotovap. Product (**34**) was obtained as a brown oil in 82% yield and 89% ee (0.16 mmol, 102 mg). Enantioselectivity was determined by chiral HPLC.

3.3 General procedure for the additive free cyclopropanation of vinyl-heterocycles with *ortho*-substituted aryl/heteroaryl-diazoacetates

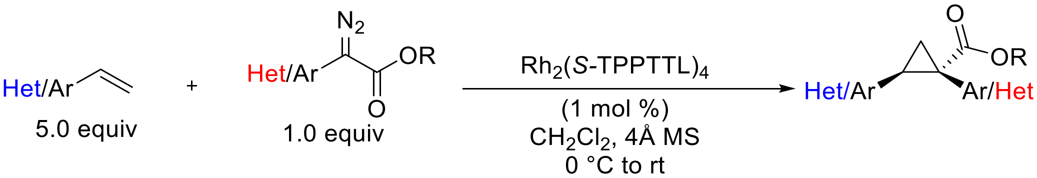

A 10 mL vial containing 4Å activated molecular sieves (0.5 g) and a stir bar was flame dried under vacuum along with a small round-bottom flask. Vessels were evacuated and purged with nitrogen 2 times to establish an inert atmosphere. Then catalyst Rh_2(*S*-TPPTTL)$_4$ (1.0 mol %, 4.9 mg, 0.002 mmol) was added to the vial. Solid diazo compound (1.0 equiv, 0.20 mmol) was added to the flame dried round bottom flask. Then vacuum was reestablished on both the vial and the flask to further remove air from the system. After 5 min, the system was flushed with nitrogen and vinyl pyridine (5.0 equiv, 1.0 mmol) was added to the vial via preweighed syringe and 2 mL distilled CH_2Cl_2 was added to the vial. The nitrogen line attached to the vial was then replaced by a balloon filled with argon and the vial was added to an ice bath to maintain the temperature at 0 ˚C and let stir. Temperature of the ice bath was monitored by thermocouple external to the reaction vessel. While the vial cooled for approximately 10 min, the diazo compound was dissolved in 3 mL distilled CH_2Cl_2 added via syringe. The round bottom flask was swirled to ensure all diazo had dissolved before the solution was loaded into the syringe. The syringe was then inserted through the vial septum and the full contents were injected into the vial in one portion maintaining the bath at 0 ˚C throughout the addition. The reaction was stirred overnight under argon in the ice bath which slowly warmed to room temperature (at least 13 h). The reaction solution was subjected to TLC to determine reaction completion (20% EtOAc/hexanes). After completion the solution was filtered through celite to remove molecular sieves before concentrating via rotovap. The crude concentrate was then directly purified by flash column chromatography (5% EtOAc/hexanes 3 CV, 5% EtOAc/hexanes to 30% EtOAc/hexanes 15 CV, 30% EtOAc/hexanes for 3-10 CV). Fractions containing only product by TLC were aggregated and concentrated via rotovap. Enantioselectivity was determined by chiral HPLC. Products **37-46** were synthesized via this method.

3.4 General procedure for the additive promoted cyclopropanation involving *ortho*-substituted aryl/heteroaryl-diazoacetates.

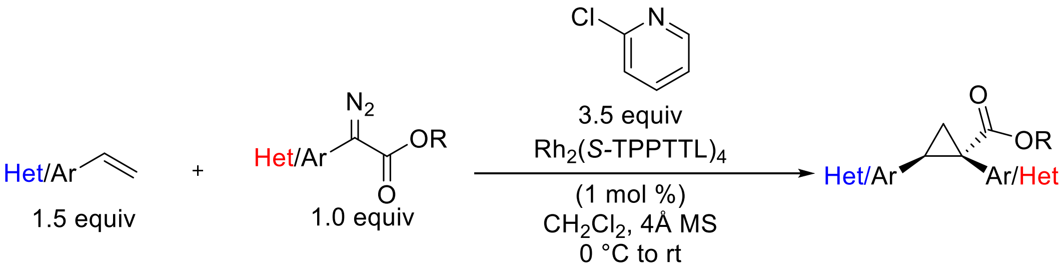

A 10 mL vial containing 4Å activated molecular sieves (0.5 g) and a stir bar was flame dried under vacuum along with a small round-bottom flask. Vessels were evacuated and purged with nitrogen 2 times to establish an inert atmosphere. Then catalytic Rh_2(*S*-TPPTTL)$_4$ (1.0 mol %, 4.9 mg, 0.002 mmol) was added to the vial. Solid diazo compound (1.0 equiv, 0.20 mmol) was added to the flame dried round bottom flask. Then vacuum was reestablished on both the vial and the flask to further remove air from the system. After 5 min, the system was flushed with nitrogen, vinyl-heterocycle (1.5 equiv, 0.30 mmol), and 2-Clpyridine (3.5 equiv, 79 mg, 66 µl, 0.70 mmol) was added to the vial via syringe along with 2 mL dry CH_2Cl_2.

The nitrogen line attached to the vial was then replaced by a balloon filled with argon and the vial was added to an ice bath to maintain the temperature at 0 ˚C and let stir. Temperature of the ice bath was monitored by thermocouple external to the reaction vessel. While the vial cooled for approximately 10 min, the diazo compound was dissolved in 3 mL dry CH_2Cl_2 added via syringe. The round bottom flask was swirled to ensure all diazo had dissolved before the solution was loaded into the syringe. The syringe was then inserted through the vial septum and the full contents were injected into the vial in one portion maintaining the bath at 0 ˚C throughout the addition. The reaction was stirred overnight under argon in the ice bath which slowly warmed to room temperature (at least 13 h). The reaction solution was subjected to TLC to determine reaction completion (20% EtOAc/hexanes). After completion the solution was filtered through celite to remove molecular sieves before concentrating via rotovap. The crude concentrate was then directly purified by flash column chromatography (5% EtOAc/hexanes 3 CV, 5% EtOAc/hexanes to 30% EtOAc/hexanes 15 CV, 30% EtOAc/hexanes for 3-10 CV). Fractions containing only product by TLC were aggregated and concentrated via rotovap. Enantioselectivity was determined by chiral HPLC. Products **37, 47-49, 52-53, 56-57, 62-65** were synthesized via this method.

3.5 General procedure for the additive promoted cyclopropanation involving *ortho*-substituted aryl/heteroaryl-diazoacetates and HFIP.

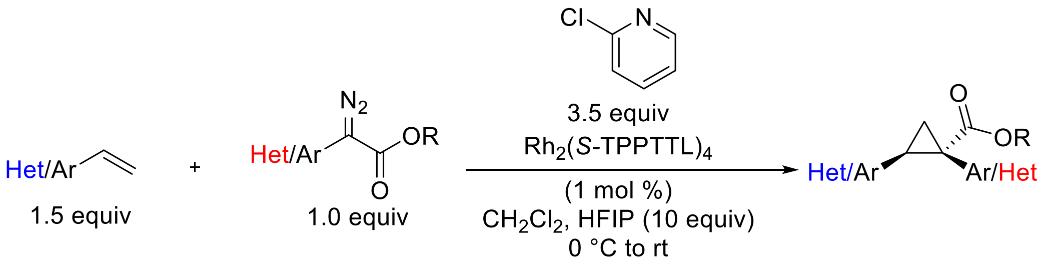

A 10 mL vial containing a stir bar was flame dried under vacuum along with a small round-bottom flask. Vessels were evacuated and purged with nitrogen 2 times to establish an inert atmosphere. Then catalytic Rh_2(*S*-TPPTTL)$_4$ (1.0 mol %, 4.9 mg, 0.002 mmol) was added to the vial. Solid diazo compound (1.0 equiv, 0.20 mmol) was added to the flame dried round bottom flask. Then vacuum was reestablished on both the vial and the flask to further remove air from the system. After 5 min, the system was flushed with nitrogen, vinyl-heterocycle (1.5 equiv, 0.30 mmol), 2-Clpyridine (3.5 equiv, 79 mg, 66 µl, 0.70 mmol), and 1,1,1-3,3,3-hexafluoroisopropanol (HFIP, 10 equiv, 340 mg, 0.21 mL, 2.0 mmol) was added to the vial via syringe along with 2 mL dry CH_2Cl_2. The nitrogen line attached to the vial was then replaced by a balloon filled with argon and the vial was added to an ice bath to maintain the temperature at 0 ˚C and let stir. Temperature of the ice bath was monitored by thermocouple external to the reaction vessel. While the vial cooled for approximately 10 min, the diazo compound was dissolved in 3 mL distilled CH_2Cl_2 added via syringe. The round bottom flask was swirled to ensure all diazo had dissolved before the solution was loaded into the syringe. The syringe was then inserted through the vial septum and the full contents were injected into the vial in one portion maintaining the bath at 0 ˚C throughout the addition. The reaction was stirred overnight under argon and allowed to warm to room temperature (at least 13 h). The reaction solution was subjected to TLC to determine reaction completion (20% EtOAc/hexanes). After completion the solution was filtered through celite to remove molecular sieves before concentrating via rotovap. The crude concentrate was then directly purified by flash column chromatography (5% EtOAc/hexanes 3 CV, 5% EtOAc/hexanes to 30% EtOAc/hexanes 15CV, 30% EtOAc/hexanes for 3-10 CV). Fractions containing product by TLC were aggregated and concentrated via rotovap. Enantioselectivity was determined by chiral HPLC. Products **37-39, 47-51, 54-55, 58-61** were synthesized via this method.

3.6 Procedure for one-Pot hydrazone oxidation/cyclopropanation

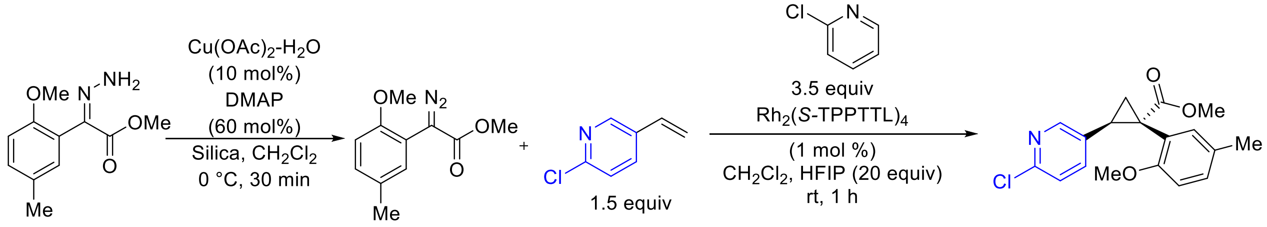

In the first step: A 20 mL scintillation vial was charged with $Cu(OAc)_2$-H_2O (3.9 mg, 0.020 mmol, 10 mol %), silica powder (44.4 mg, 100 wt%, SiliaFlash® P60, 40-63 µm), and 1.0 mL solution of 0.06 mol/L DMAP in CH_2Cl_2 The initial mixture was stirred vigorously with a stir bar (600 rpm) under air for 5 min before hydrazone was added. In a 4 mL scintillation vial, methyl (*Z*)-2-hydrazineylidene-2-(2-methoxy-5-methylphenyl)acetate (44.4 mg, 0.20 mmol, 1.0 equiv) was dissolved in 1.0 mL of the 0.06 mol/L DMAP in CH_2Cl_2 solution. The hydrazone/DMAP CH_2Cl_2 solution was then transferred by syringe in one portion to the initial mixture of $Cu(OAc)_2$-H_2O/silica/DMAP CH_2Cl_2 solution. The reaction was stirred for 0.5 h before next step to afford a crude solution of **36**.

In the second step: a 20 mL scintillation vial equipped with a stir bar was flame dried under vacuum. After cooling down, the vail was charged with Rh_2(*S*-TPPTTL)$_4$ (4.9 mg, 1.0 mol %, 0.0020 mmol), then flushed with nitrogen for 3 times and the nitrogen balloon was left on the septum. Then HFIP (672.2 mg, 0.42 mL, 20 equiv, 4.0 mmol), 2-chloropyridine (79.5 mg, 66 µL, 3.5 equiv, 0.70 mmol), 2-chloro-5-vinylpyridine (**35,** 41.9 mg, 1.5 equiv, 0.30 mmol) and 2.0 mL CH_2Cl_2 were added sequentially via syringe, the mixture was stirred at 600 rpm for 10 min before crude diazo injection. The crude diazo mixture from step **1** (~1.5 mL) was added by syringe to the 2-chloro-5-vinylpyridine/Rh_2(*S*-TPPTTL)$_4$/HFIP/2-chloropyridine solution in one portion. The reaction was then stirred 1 h under nitrogen at r.t. After completion the solution was concentrated under rotovap and purified by flash column chromatography (5 % EtOAc/hexanes 3 CV, 5 % EtOAc/hexanes to 30 % EtOAc/hexanes 15 CV, 30 % EtOAc/hexanes 10 CV). Cyclopropanation product was concentrated to give a clear colorless oil in 83% yield (**37,** 55.2 mg,0.166 mmol) and 98% ee. Repeating the same reaction without addition of the HFIP resulted in recovery of unreacted diazo compound **36**.

Results and Discussion

4. Characterization of synthesized compounds.

4.1 Characterization of novel starting materials

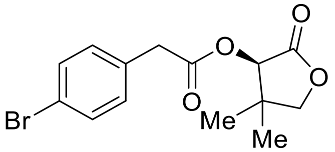

(*R*)-4,4-dimethyl-2-oxotetrahydrofuran-3-yl 2-(4-bromophenyl)acetate. This compound was prepared according to the procedure outlined in **2.2.1.1** on 23.3 mmol scale. After isolation the product was obtained as off-white crystalline solid (70% yield, 5.3 g, 16 mmol).
MP: 62-63℃
^{1}H NMR (400 MHz, $CDCl_3$) δ 7.45(d, *J* = 8.2 Hz, 2H), 7.18 (d, *J* = 8.2 Hz, 2H), 5.34 (s, 1H), 4.05 – 3.95 (m, 2H), 3.72 (s, 2H), 1.12 (s, 3H), 0.99 (s, 3H).
^{13}C NMR (151 MHz, $CDCl_3$) δ 172.1, 169.9, 132.2, 131.8, 131.1, 121.5, 76.2, 75.5, 40.2, 40.2, 22.9, 19.8.
HRMS: (+p APCI) calculated for [$C_{14}H_{16}O_4{}^{79}Br$ +] 327.0227, found 327.0227
IR(neat): 2967, 2931, 1779, 1745, 1592, 1488, 1465, 1400, 1269, 1351, 1297, 1242, 1139, 1072, 1031, 1012, 997, 914, 851, 801, 754, 735, 573, 540, 491 cm^{-1}

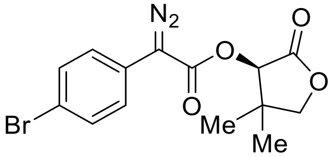

(*R*)-4,4-dimethyl-2-oxotetrahydrofuran-3-yl 2-(4-bromophenyl)-2-diazoacetate S17. This compound was prepared according to the procedure outlined **in 2.2.1.2** on 6.11 mmol scale. After isolation the product was obtained as a powdery orange solid (60% yield, 1.30 g, 3.68 mmol).
MP: 95-99℃ diazo decomposes rapidly after melting
^{1}H NMR(400 MHz, $CDCl_3$) δ 7.59 – 7.42 (m, 2H), 7.42 – 7.31 (m, 2H), 5.52 (s, 1H), 4.08 (m, 2H), 1.25 (s, 3H), 1.13 (s, 3H).
^{13}C NMR (151 MHz, $CDCl_3$) δ 172.1, 163.4, 132.2, 125.5, 123.9, 119.9, 76.2, 75.4, 40.2, 22.9, 19.8.
HRMS: (+p APCI) Compound decomposed in ESI-MS to give OH-insertion product. Peak calculated for [$C_{14}H_{14}O_4{}^{79}Br$ +] 325.007, found 325.0075
IR(neat): 2970, 2089, 1783, 1738, 1490, 1464, 1365, 1275, 1230, 1217, 1141, 1086, 1031, 1009, 996, 970, 920, 821, 781, 731, 647, 575, 543, 528, 515, 493 cm^{-1}

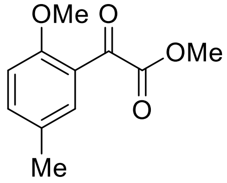

Methyl 2-(2-methoxy-5-methylphenyl)-2-oxoacetate. This compound was prepared according to the procedure outlined in **2.2.2.1** from the reaction between 4-methyl anisole and methyl-oxalyl chloride on 81.9 mmol scale. After isolation the product was obtained as a light yellow liquid (93% yield, 15.9 g, 76.4 mmol).
^{1}H NMR (600 MHz, $CDCl_3$) δ 7.67 (d, *J* = 2.5 Hz, 1H), 7.39 (dd, *J* = 8.5, 2.4 Hz, 1H), 6.88 (d, *J* = 8.5 Hz, 1H), 3.91 (s, 3H), 3.84 (s, 3H).

^{13}C NMR (151 MHz, CDCl$_3$) δ186.4, 165.7, 158.5, 137.1, 130.9, 130.6, 122.4, 112.2, 56.3, 52.3, 20.2.
HRMS: (+p APCI) calculated for [$C_{11}H_{13}O_4$+] 209.0808, found 209.0806
IR(neat): 2951, 1739, 1667, 1608, 1581, 1497, 1412, 1272, 1245, 1224, 1179, 1155, 1133, 1019, 947, 901, 864, 813, 778, 712,669, 588, 537, 489 cm^{-1}

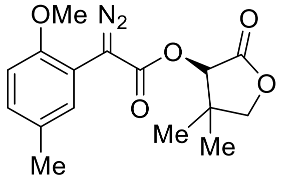

(*R*)-4,4-dimethyl-2-oxotetrahydrofuran-3-yl 2-diazo-2-(2-methoxy-5-methylphenyl)acetate S18.
This compound was prepared according to the procedure outlined in **2.2.2.4** from (*R*)-4,4-dimethyl-2-oxotetrahydrofuran-3-yl 2-(2-methoxy-5-methylphenyl)-2-oxoacetate. After isolation the product was obtained as a yellow solid (7.3 g, 23 mmol, 82% yield).
^{1}**H NMR** (500 MHz, CDCl$_3$) δ 7.37 (d, J = 2.2 Hz, 1H), 7.09 – 7.04 (m, 1H), 6.80 (d, J = 8.4 Hz, 1H), 5.50 (s, 1H), 4.10 – 4.02 (m, 2H), 3.84 (s, 3H), 2.33 – 2.29 (m, 3H), 1.25 (s, 3H), 1.12 (s, 3H);
^{13}**C NMR** (101 MHz, CDCL3) δ 172.4, 153.5, 130.6, 130.5, 129.4, 112.5, 110.9, 76.2, 75.2, 55.6, 40.2, 23.0, 20.5, 19.8
HRMS: (ESI) m/z calculated for $C_{16}H_{18}N_2O_5Na$ [M+Na]+, 475.1533; found 475.1545.

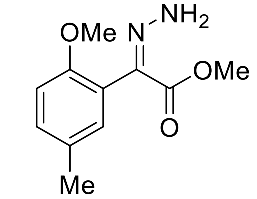

Methyl (*E*/*Z*)-2-hydrazineylidene-2-(2-methoxy-5-methylphenyl)acetate. This compound was prepared according to the procedure outlined in **2.2.3.1** between methyl 2-(2-methoxy-5-methylphenyl)-2-oxoacetate and hydrazine on 38.4 mmol scale. After isolation the product was obtained as a mixture of isomers as a clear, colorless clear colorless oil (89% yield, 7.56 g, 34 mmol). Pure *Z*-isomer appears as an off-white solid, pure *E*-isomer is obtained as a white solid.
MP: 63-65 ̊C – *Z*-isomer, 72-75℃ – *E*-isomer,
^{1}**H NMR** (400 MHz, CDCl$_3$) *Z*-Isomer: δ 8.13 (s, 2H), 7.14 – 7.12 (m, 2H), 7.10 (dt, J = 2.4, 0.8 Hz, 1H), 6.79 (d, J = 8.1 Hz, 1H), 3.76 (s, 3H), 3.73 (s, 3H), 2.30 (d, J = 0.8 Hz, 3H). *E*-isomer: δ 7.21 (ddq, J = 8.6, 2.3, 0.7 Hz, 1H), 6.97 (dt, J = 2.3, 0.6 Hz, 1H), 6.90 (d, J = 8.4 Hz, 1H), 6.11 (s, 2H), 3.83 (s, 3H), 3.77 (s, 3H).
^{13}**C NMR** (151 MHz, CDCl$_3$) *Z*-Isomer: δ163.7, 155.9, 130.7, 130.3, 130.2, 126.3, 111.1, 56.0, 51.6, 20.6. *E*-isomer: δ 165.3, 155.1, 136.1, 131.9, 130.8, 130.7, 118.3, 111.9, 56.1, 52.6, 20.7.
HRMS: (+p APCI) calculated for [$C_{11}H_{15}O_3N_2$+] 223.1077, found 223.1077 for *Z* isomer, found 223.1079 for *E* isomer.
IR(neat): *Z*-Isomer: 3454, 3293, 2948, 2836, 1695, 1575, 1498, 1463, 1435, 1295, 1266, 1245, 1186, 1150, 1130, 1037, 1025, 993, 887, 808, 730, 670, 496. *E*-Isomer: 3407,3294, 3210, 2948, 2838, 1708, 1608, 1557, 1496, 1435, 1316, 1238, 1185, 1119, 1046, 1025, 950, 872, 810, 781, 729, 468 cm^{-1}

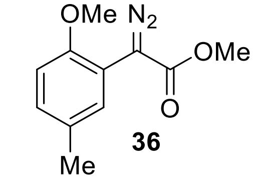

36

Methyl 2-diazo-2-(2-methoxy-5-methylphenyl)acetate (S19)
This compound was prepared according to the procedure outlined in **2.2.3.2** between methyl (*E*/*Z*)-2-hydrazineylidene-2-(2-methoxy-5-methylphenyl)acetate and TsNIK on 14.06 mmol scale. After isolation the product was obtained as a mixture of isomers as a bright orange powder (80% yield, 2.5 g, 11.3 mmol).
MP: 55-60℃
^{1}**H NMR** (600 MHz, CDCl$_3$) δ 7.36 (s, 1H), 7.06 (d, *J* = 8.41 Hz, 1H), 6.79 (d, *J* = 8.42 Hz, 1H), 3.83 (s, 3H), 3.82 (s, 3H), 2.31 (s, 3H).
^{13}**C NMR** (151 MHz, CDCl$_3$) δ 166.8, 153.5, 130.7, 130.5, 129.2, 113.3, 110.9, 55.7, 51.9, 20.6. Please note that the diazo carbon was not visible by 13C NMR.
HRMS: (+p APCI) calculated for [$C_{11}H_{13}O_3N_2$+] 221.0921, found 221.0922
IR(neat): 2090, 1693, 1503, 1434, 1339, 1291, 1248, 1186, 1138, 1048, 804, 743 cm^{-1}

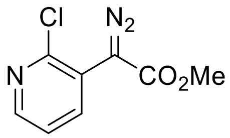

Methyl 2-(2-chloropyridin-3-yl)-2-diazoacetate (S20). This compound was prepared according to the procedure outlined in **2.2.4** between methyl 2-(2-chloropyridin-3-yl)acetate and *p*-ABSA on 29.1 mmol scale. After isolation the product was obtained as a bright yellow solid (96% yield, 5.9 g, 27.9 mmol).
MP: 71-73 ℃
^{1}H NMR (400 MHz, $CDCl_3$) δ 8.35 (dd, *J* = 4.7, 1.9 Hz, 1H), 7.95 (dd, *J* = 7.8, 1.9 Hz, 1H), 7.32 (dd, *J* = 7.8, 4.7 Hz, 1H), 3.86 (s, 3H).
^{13}C NMR (151 MHz, $CDCl_3$) δ 165.4, 149.2, 148.9, 140.5, 122.7, 121.6, 52.5. Please note that the diazo carbon was not visible by 13C NMR.
HRMS: (+p APCI) calculated for [$C_8H_7O_2N_3{}^{35}Cl$ +] 212.0221, found 212.0222
IR(neat): 2097, 1694, 1555, 1455, 1435, 1402, 1344, 1272, 1211, 1193, 1162, 1128, 1099, 1060, 1023, 1006, 798, 737, 727 cm^{-1}

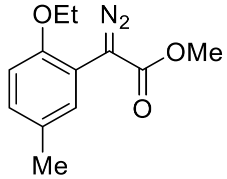

Methyl 2-diazo-2-(2-ethoxy-5-methylphenyl)acetate (S21)
This compound was prepared according to the procedure outlined in **2.2.5.2** from methyl 2-(2-ethoxy-5-methylphenyl)-2-oxoacetate on 9.6 mmol scale. After isolation the product was obtained as an orange oil which crystallized upon standing (1.08 g, 4.61 mmol, 52% yield over 2 steps)
^{1}H NMR (400 MHz, $CDCl_3$) d 7.36 (d, J = 2.3 Hz, 1H), 7.02 (ddd, J = 8.4, 2.3, 0.8 Hz, 1H), 6.77 (d, J = 8.4 Hz, 1H), 4.02 (q, J = 7.0 Hz, 2H), 3.83 (s, 3H), 2.29 (d, J = 0.7 Hz, 3H), 1.41 (t, J = 6.9 Hz, 3H);
^{13}C NMR (101 MHz, CDCL3) δ 166.9, 152.8, 130.6, 130.4, 129.0, 113.3, 111.7, 64.2, 52.0, 20.7, 14.8;
HRMS (ESI) m/z calculated for $C_{12}H_{14}N_2O_3Na$ [M+Na]+, 257.0897; found 257.0899.

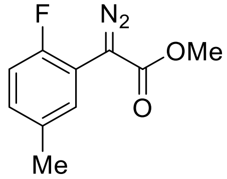

Methyl 2-diazo-2-(2-fluoro-5-methylphenyl)acetate (S22)
This compound was prepared according to the procedure outlined in **2.2.6** from 2-bromo-1-fluoro-4-methylbenzene. After isolation the product was obtained as a yellow solid (406 mg, 1.950 mmol, 47% yield over 2 steps)
^{1}H NMR (500 MHz, $CDCl_3$) δ 7.49 – 7.43 (m, 1H), 7.05 – 6.99 (m, 1H), 6.96 (dd, J = 10.8, 8.4 Hz, 1H), 3.86 (s, 3H), 2.33 (q, J = 0.8 Hz, 3H);
^{13}C NMR (101 MHz, $CDCl_3$) δ 157.9, 155.5, 134.2, 134.2, 129.7 (d, *J* = 2.0 Hz), 129.2 (d, *J* = 8.0 Hz), 115.4, 115.2, 52.1 20.8.
^{19}F NMR (376 MHz, DMSO) δ -118.92 (q, *J* = 7.9 Hz).
HRMS (ESI) m/z calculated for $C_{10}H_9FN_2O_2Na$ [M+Na]+, 231.054; found 231.0543.

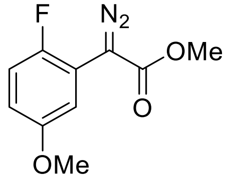

Methyl 2-diazo-2-(2-fluoro-5-methoxyphenyl)acetate (S23)
This compound was prepared according to the procedure outlined in **2.2.7** from 2-(2-fluoro-5-methoxyphenyl)acetic acid. After isolation the product was obtained as a bright yellow solid (977 mg, 4.36 mmol, 56% yield over 2 steps)
^{1}H NMR (600 MHz, $CDCl_3$) δ 7.29 – 7.22 (m, 1H), 6.98 (dd, J = 10.6, 9.0 Hz, 1H), 6.74 (ddd, J = 9.0, 3.9, 3.1 Hz, 1H), 3.86 (s, 3H), 3.79 (s, 3H);
^{13}C NMR (101 MHz, $CDCl_3$) δ 165.6, 156.0, 155.9, 153.9, 151.5, 116.2, 116.0, 113.9 (d, *J* = 8.1 Hz), 113.4 (d, *J* = 2.2 Hz), 55.8, 52.2.
^{19}F NMR (376 MHz, $CDCl_3$) δ -124.99.
HRMS (ESI) m/z calculated for $C_{10}H_9FN_2O_3Na$ [M+Na]+, 247.0489; found 247.0490

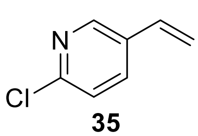

35

2-Chloro-5-vinylpyridine (35). This compound was prepared according the procedure outlined in **2.2.8.1** from the Wittig reaction between methyltriphenylphosphinium bromide (1.2 equiv, 85 mmol, 30.3 g), 6-chloronicotinaldehyde (71 mmol, 10 g), and potassium *tert*-butoxide (1.2 equiv, 85 mmol, 9.5 g). After isolation, the product was obtained as a clear colorless oil (65% yield, 6.4g, 46 mmol).
^{1}H NMR (600 MHz, $CDCl_3$) δ 8.31 (d, *J* = 2.36, 1H), 7.65 (dd, *J* = 2.64, 8.42 Hz, 1H), 7.23 (d, *J* = 8.33 Hz, 1H), 6.61 (dd, *J*= 10.97, 17.58 Hz, 1H), 5.76 (d, *J*= 17.61 Hz, 1H), 5.36 (d, *J*= 10.93 Hz, 1H).
^{13}C NMR (151 MHz, $CDCl_3$) δ 150.3, 147.9, 135.3, 132.0, 124.0, 124.0, 116.9
HRMS: (+p APCI) calculated for [$C_7H_7N^{35}Cl$ +] 140.0262, found 140.0261
IR(neat): 1633, 1592, 1558, 1458, 1422, 1360, 1142, 1099, 1019, 999, 919, 834, 804, 748, 639, 631, 622, 502, 489 cm^{-1}

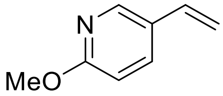

2-Methoxy-5-vinylpyridine (S24). This compound was prepared according the procedure outlined in **2.2.8.1** from the Wittig reaction between methyltriphenylphosphinium bromide (1.2 equiv, 85 mmol, 30.3 g), 6-methoxynicotinaldehyde (71 mmol, 10 g), and potassium *tert*-butoxide (1.2 equiv, 85 mmol, 9.5 g). After isolation, the product was obtained as a clear colorless oil (63% yield).
^{1}H NMR (600 MHz, $CDCl_3$) δ 8.11 (d, *J* = 2.28, 1H), 7.68 (dd, *J* = 2.53, 8.85 Hz, 1H), 6.70 (d, *J* = 8.57 Hz, 1H), 6.63 (dd, *J*= 10.81, 17.32 Hz, 1H), 5.62 (d, *J*= 17.50 Hz, 1H), 5.20 (d, *J*= 10.92 Hz, 1H), 3.92 (s, 3H).
^{13}C NMR (151 MHz, $CDCl_3$) δ 163.8, 145.5, 135.2, 133.0, 126.7, 112.9, 110.8, 53.4
HRMS: (+p APCI) calculated for [$C_8H_{10}ON$ +] 136.0757, found 136.0757
IR(neat): 1632, 1598, 1566, 1491, 1461, 1368, 1303, 1282, 1255, 1126, 1019, 987, 901, 830, 764, 729, 587, 554, 509 cm^{-1}

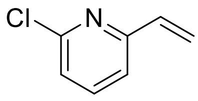

66

2-Chloro-6-vinylpyridine (66). This compound was prepared according to the procedure outlined in **2.2.8.1** from the Wittig reaction between methyltriphenylphosphinium bromide (1.2 equiv, 42.4 mmol, 15.1 g), 2-chloronicotinaldehyde (35.3 mmol, 5.0 g), and potassium *tert*-butoxide (1.2 equiv, 42.4 mmol, 4.76 g). 6-chloropicolinaldehyde. After isolation, the product was obtained as a clear yellow oil (54% yield, 19 mmol, 2.65 g).
^{1}H NMR (400 MHz, $CDCl_3$) δ 7.58 (t, *J* = 7.7 Hz, 1H), 7.21 (d, *J* = 7.6 Hz, 1H), 7.16 (d, *J* = 7.9 Hz, 1H), 6.72 (dd, *J* = 17.4, 10.8 Hz, 1H), 6.24 (d, *J* = 17.4 Hz, 1H), 5.50 (d, *J* = 10.8 Hz, 1H).
^{13}C NMR (151 MHz, $CDCl_3$) δ 156.4, 151.2, 139.0, 135.4, 122.9, 119.9, 119.6.
HRMS: (+p APCI) calculated for [$C_7H_7N^{35}Cl$ +] 140.0262, found 140.0263
IR(neat): 1580, 1551, 1441, 1411, 1396, 1161, 1135, 983, 931, 846, 803, 744, 676 cm^{-1}

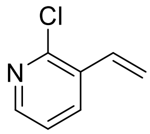

2-Chloro-3-vinylpyridine (S25). This compound was prepared according to the procedure outlined in **2.2.8.1** from the Wittig reaction between methyltriphenylphosphinium bromide (1.2 equiv, 42.4 mmol, 15.1g), 2-chloronicotinaldehyde (35.3 mmol, 5.0 g), and potassium *tert*-butoxide (1.2 equiv, 42.4 mmol, 4.76 g). After isolation, the product was obtained as a clear colorless oil (58% yield, 20.6 mmol, 2.88 g).
^{1}H NMR (400 MHz, $CDCl_3$) δ 8.18 (t, *J*=2.35 Hz, 1H), 7.76 (dd, *J*=1.96, 7.75 Hz, 1H), 7.13 (dd, *J*=4.71, 7.75 Hz, 1H), 6.91 (dd, *J*=11.01, 17.54 Hz, 1H), 5.68 (d, *J*= 17.49 Hz, 1H), 5.38 (d, *J*=10.99 Hz, 1H)
^{13}C NMR (151 MHz, $CDCl_3$) δ 149.8, 148.5, 134.9, 132.2, 131.9, 122.7, 118.6
HRMS: (+p APCI) calculated for [$C_7H_7N^{35}Cl$ +] 140.0262, found 140.0262
IR(neat): 1627, 1577, 1558, 1450, 1426, 1410, 1380, 1186, 1128, 1062, 1028, 985, 921, 804, 752, 683, 658 cm^{-1}

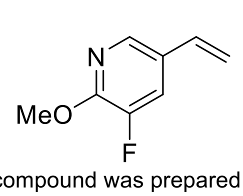

2-Methoxy-3-fluoro-5-vinylpyridine (S26). This compound was prepared according to the procedure outlined in **2.2.8.2** from the Suzuki coupling between 2-methoxy-3-fluoro-5-bromopyridine (6.65 mmol, 1.37 g) and vinylboronic acid pinacol ester (1.5 equiv, 1.54 g, 9.98 mmol) in the presence of PAPh (1.5 mol %, 0.1mmol, 29 mg), and K_2CO_3 (2.5 equiv, 16.6

mmol, 2.3 g) and $Pd_2(dba)_3$ (0.5 mol %, 0.03 mmol, 30.4 mg). After isolation, the product was obtained as a clear colorless oil (71% yield, 4.73 mmol, 725 mg).
^{1}H NMR (600 MHz, $CDCl_3$) δ 7.86 (s, 1H), 7.43 (d, *J* = 11.00 Hz, 1H), 6.62 (dd, *J* = 11.26, 17.59 Hz, 1H), 5.61 (d, *J*= 17.58 Hz, 1H), 5.26 (d, *J*= 10.96 Hz, 1H), 4.02 (s, 3H).
^{13}C NMR (151 MHz, $CDCl_3$) δ 152.9 d, *J* = 11.5 Hz), 148.6, 139.8, 132.1, 127.9, 119.1 (d, *J* = 15.5 Hz), 114.3, 53.8
^{19}F NMR (151 MHz, $CDCl_3$) δ -140.15 (d, *J* = 11.2 Hz).
HRMS: (+p APCI) calculated for [C_8H_9ONF +] 154.0663, found 154.0662
IR(neat): 1633, 1611, 1571, 1493, 1460, 1440, 1420, 1400, 1318, 1255, 1211, 1196, 1173, 1146, 1133, 1042, 1013, 986, 959, 896, 778, 750, 700, 627, 555, 525, 500, 440, 423 cm^{-1}

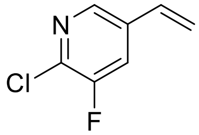

2-Chloro-3-fluoro-5-vinylpyridine (S27). This compound was prepared according to the procedure outlined in **2.2.8.2** from the Suzuki coupling between 2-methoxy-3-chloro-5-bromopyridine (12 mmol, 2.5 g) and vinylboronic acid pinacol ester (1.5 equiv, 2.7 g, 18 mmol) in the presence of PAPh (1.5 mol %, 0.18 mmol, 52 mg), and K_2CO_3 (2.5 equiv, 30 mmol, 4.1 g) and $Pd_2(dba)_3$ (0.5 mol %, 0.06 mmol, 54 mg). After isolation, the product was obtained as a clear colorless oil which crystallizes in the freezer as needlelike crystals that melt at room temperature (75% yield, 8.57 mmol, 1.35 g).
MP: 25℃
^{1}H NMR (400 MHz, $CDCl_3$) δ 8.18 (d, *J* = 2.0 Hz, 1H), 7.51 (dd, *J* = 9.0, 2.0 Hz, 1H), 6.67 (dd, *J* = 17.5, 11.0 Hz, 1H), 5.83 (d, *J* = 17.6 Hz, 1H), 5.48 (d, *J* = 11.0 Hz, 1H).
^{13}C NMR (151 MHz, $CDCl_3$) δ 155.7, 153.9, 142.9 (d, *J* = 5.0 Hz), 137.8 (d, *J* = 19.8 Hz), 134.5 (d, *J* = 2.9 Hz), 131.3, 120.6 (d, *J* = 19.0 Hz), 118.4.
^{19}F NMR (376 MHz, $CDCl_3$) δ -119.60 (d, *J*=9.0 Hz).
HRMS: (+p APCI) calculated for [$C_7H_6N^{35}ClF$ +]158.0167, found 158.0168
IR(neat): 3057, 1633, 1593, 1560, 1453, 1420, 1394, 1293, 1208, 1168, 1087, 985, 919, 896, 731, 710 690, 649, 635, 545, 471 cm^{-1}

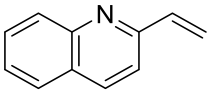

2-Vinylquinoline (S28). This compound was prepared according the procedure outlined in **2.2.8.2** from the Suzuki coupling between 2-chloroquinoline (5.0 mmol, 818 mg) and vinylboronic acid pinacol ester (1.2 equiv, 924 mg, 6.0 mmol) in the presence of PAPh (5.0 mol %, 0.25 mmol, 73.1 mg), and K_2CO_3 (2.5 equiv, 12.5 mmol, 1.73 g) and $Pd_2(dba)_3$ (2.0 mol %, 0.10 mmol, 91.6 mg). After isolation, the product was obtained as a light yellow oil (96% yield, 4.81 mmol, 746 mg). Over time product polymerizes to a dark green liquid even if stored at -20℃ in the dark. The polymer may be separated from the title compound by short silica-plug through a pipette, eluting with CH_2Cl_2 as a yellow solution while the dark colored polymer remains adhered to silica gel. Batches of the title compound either freshly columned or those that contained a high degree of polymer were equally suitable for highly enantioselective cyclopropanation.
^{1}H NMR (600 MHz, $CDCl_3$) δ 8.04 (d, *J* = 8.5 Hz, 1H), 7.97 (d, *J* = 8.6 Hz, 1H), 7.70 (d, *J* = 8.4 Hz, 1H), 7.68-7.62 (m, 1H) 7.52 (d, *J* = 8.6 Hz, 1H), 7.44 (m, 1H), 7.02 (dd, *J* = 17.6, 10.9 Hz, 1H), 6.25 (d, *J* = 17.6 Hz, 1H), 5.63 (d, *J* = 10.9 Hz, 1H).
^{13}C NMR (126 MHz, $CDCl_3$) δ 156.0, 148.0, 137.9, 136.3, 129.6, 129.4, 127.5, 127.5, 126.3, 119.8, 118.4.
HRMS: (+p APCI) calculated for [$C_{11}H_{10}N$ +] 156.0808, found 156.0808
IR(neat): 3016, 1738, 1616, 1597, 1504, 1427, 1373, 1339, 1231, 1217, 1097, 991, 926, 834, 764, 715, 699, 616, 472 cm^{-1}

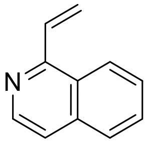

1-Vinylisoquinoline (S29). This compound was prepared according to the procedure outlined in **2.2.8.2** from the Suzuki coupling between 1-chloroisoquinoline (5.0 mmol, 818 mg) and vinylboronic acid pinacol ester (1.2 equiv, 924 mg, 6.0 mmol) in the presence of PAPh (5.0 mol %, 0.25 mmol, 73.1 mg), and K_3CO_3 (2.5 equiv, 12.5 mmol, 1.73 g) and $Pd_2(dba)_3$ (2.0 mol %, 0.10 mmol, 91.6 mg). After isolation, the product was obtained as a light yellow oil which rapidly polymerizes, changing appearance to a dark brown liquid (47% yield, 2.29 mmol, 356 mg). The polymer may be separated from the title compound by short silica-plug through a pipette, eluting with CH_2Cl_2 as a yellow solution while the dark colored polymer remains adhered to silica gel. Batches of the title compound either freshly columned or those that contained a high degree of polymer were equally suitable for highly enantioselective cyclopropanation.
^{1}H NMR (400 MHz, $CDCl_3$) δ 8.52 (d, *J* = 5.6 Hz, 1H), 8.22 (dt, *J* = 8.5, 1.1 Hz, 1H), 7.83 – 7.73 (m, 1H), 7.69 – 7.60 (m, 2H), 7.57 (ddd, *J* = 13.0, 6.1, 4.2 Hz, 3H), 6.53 (dd, *J* = 16.9, 2.0 Hz, 1H), 5.71 (dd, *J* = 10.8, 2.0 Hz, 1H).
^{13}C NMR (151 MHz, $CDCl_3$ δ 154.8, 142.4, 136.6, 132.2, 129.9, 127.2, 127.2, 126.4, 124.6, 121.7, 120.3.
HRMS: (+p APCI) calculated for [$C_{11}H_{10}N$ +] 156.0808, found 156.0809
IR(neat): 3048, 1738, 1617, 1579, 1553, 1500, 1414, 1320, 1244, 1217, 1140, 1013, 979, 936, 869, 822, 745, 710, 536 cm^{-1}

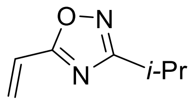

3-Isopropyl-5-vinyl-1,2,4-oxadiazole (S30). This compound was prepared according to the procedure outlined in **2.2.9** on 9.8 mmol scale. After isolation, the product was obtained as a clear colorless oil (550 mg, 3.98 mmol, 40% yield over 2 steps)
^{1}H NMR (400 MHz, $CDCl_3$) δ 6.66 (dd, *J* = 17.7, 11.1 Hz, 1H), 6.46 (d, *J* = 17.7 Hz, 1H), 5.91 (d, *J* = 11.1 Hz, 1H), 3.08 (hept, *J* = 6.9 Hz, 1H), 1.33 (d, *J* = 7.0 Hz, 6H).
^{13}C NMR (151 MHz, $CDCl_3$) δ 175.5, 174.2, 128.2, 120.7, 26.7, 20.5.
HRMS: (+p APCI) calculated for [$C_7H_{11}ON_2$ +] 139.0866, found 139.0867
IR(neat): 2973, 2934, 1651, 1571, 1547, 1505, 1465, 1415, 1387, 1353, 1310, 1261, 1215, 1164, 1096, 1066, 1024, 1015, 981, 953, 900, 878, 793, 728, 729, 710, 693 cm^{-1}

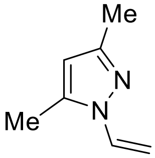

3,5-Dimethyl-1-vinyl-*1H*-pyrazole (S31). This compound was prepared according to the procedure outlined in **2.2.10** on 31 mmol scale. After isolation, the product was obtained as a clear colorless oil (1.8 g, 15 mmol, 47% yield)
^{1}H NMR (400 MHz, $CDCl_3$) δ 6.83 (dd, *J* = 15.4, 8.9 Hz, 1H), 5.82 (s, 1H), 5.55 (d, *J* = 15.4 Hz, 1H), 4.72 (d, *J* = 8.9 Hz, 1H), 2.22 (d, *J* = 1.0 Hz, 6H).
^{13}C NMR (151 MHz, $CDCl_3$) δ 149.8, 139.1, 129.2, 106.8, 99.5, 13.6, 10.9.
HRMS: (+p APCI) calculated for [$C_7H_{11}N_2$+] 123.0917, found 123.0918
IR(neat): 2924, 1720, 1645, 1561, 1446, 1427, 1372, 1344, 1283, 1240, 1119, 1044, 1014, 957, 912, 874, 793, 732, 699, 626 543 cm^{-1}

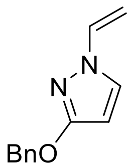

3-(benzyloxy)-1-vinyl-*1H*-pyrazole (S32)
This compound was prepared according to the procedure outlined in **2.2.11** on 5.74 mmol scale. After isolation, the product was obtained as a clear colorless oil (475 mg, 2.372 mmol, 41.3 % yield).
^{1}H NMR (400 MHz, $CDCl_3$) δ 7.49 – 7.42 (m, 2H), 7.42 – 7.27 (m, 4H), 6.81 (dd, J = 15.5, 8.8 Hz, 1H), 5.81 (d, J = 2.6 Hz, 1H), 5.39 (d, J = 15.5 Hz, 1H), 5.25 (s, 2H), 4.67 (d, J = 8.8 Hz, 1H);
^{13}C NMR (101 MHz, $CDCl_3$) δ 164.3, 136.9, 132.7, 129.5, 128.5, 128.1, 128.0, 98.1, 93.2, 71.3;
HRMS (ESI) m/z calculated for [$C_{12}H_{13}N_2O$+H+], 201.1022; found 201.1019.

4.1 Characterization of known cyclopropanation products

All products shown with absolute stereo-configuration generated with Rh$_2$(S-TPPTTL)$_4$* **(7)

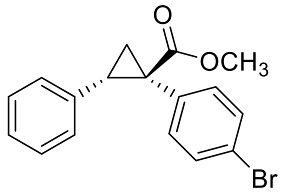

Methyl (1*R*,2*S*)-1-(4-bromophenyl)-2-phenylcyclopropane-1-carboxylate (44)
This compound was prepared according to **General procedure 3.3** and **General procedure 3.4** from the reaction between **S2** (0.20 mmol, 50 mg) and freshly columned styrene (5.0 equiv, 1.00 mmol, 100 mg). Compound prepared according to **General procedure 3.3** was isolated in 58% yield and 48% ee (0.11 mmol, 38 mg). Compound prepared according to **General procedure 3.4** was isolated in 61% yield and 0% ee (0.12 mmol, 40 mg). After isolation, product was obtained as a white solid.
^{1}H NMR (400 MHz, $CDCl_3$) δ 7.28 – 7.24 (m, 3H), 7.16 – 7.05 (m, 2H), 6.98 – 6.86 (m, 2H), 6.86 – 6.75 (m, 2H), 4.83 (d, *J* = 11.9 Hz, 1H), 4.64 (d, *J* = 11.9 Hz, 1H), 3.22 (dd, *J* = 9.4, 7.5 Hz, 1H), 2.28 (dd, *J* = 9.4, 5.2 Hz, 1H), 1.97 (dd, *J* = 7.5, 5.2 Hz, 1H).
^{13}C NMR (151 MHz, $CDCl_3$) δ 171.7, 135.4, 133.8, 133.1, 131.1, 128.2, 128.2, 127.0, 121.7, 95.1, 74.6, 36.7, 34.1, 20.3.
Chiral HPLC: (OJ-H, 30 min, 1 mL/min, 1% *i*-PrOH in *n*-hexane, UV 230 nm) RT: 10.03 min, 14.97 min.

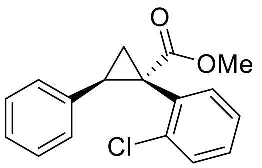

Methyl (1*S*,2*R*)-1-(2-chlorophenyl)-2-phenylcyclopropane-1-carboxylate (41)
This compound was prepared according to **General procedure 3.3** and **General procedure 3.4** from the reaction between **S5** (0.20 mmol, 42 mg) and freshly columned styrene (5.0 equiv, 1.00 mmol, 100 mg). Compound prepared according to **General procedure 3.3** was isolated in 71% yield and 55% ee (0.14 mmol, 40 mg). Compound prepared according to **General procedure 3.4** was isolated in 79% yield and 84% ee (0.16 mmol, 45 mg). After isolation, enantio-enriched product was obtained as a clear colorless oil.
^{1}H NMR (400 MHz, $CDCl_3$) δ 7.15 (broad m, 4H), 7.08 (m, 3H), 6.87 – 6.76 (dd, *J* = 6.9, 2.0 Hz 2H), 3.70 (s, 3H), 3.34 (t, *J* = 8.4 Hz, 1H), 2.13 (s, 1H), 1.94 (dd, *J* = 7.5, 5.2 Hz, 1H).
^{13}C NMR (151 MHz, $CDCl_3$) δ 173.4, 137.3, 133.3, 129.3, 128.6, 127.9, 129.4, 126.4, 126.1, 64.4, 52.7, 33.3, 25.4, 21.5
Chiral HPLC: (OJ-H, 30 min, 1 mL/min, 1% *i*-PrOH in *n*-hexane, UV 230 nm) RT: 12.80 min, 20.73 min

4.3 Characterization of novel cyclopropanation products

All products shown with absolute stereo-configuration generated with Rh_2(S-TPPTTL)$_4$* **(7) *or Rh_2(R-p-Ph-TPCP)$_4$* **(6)**

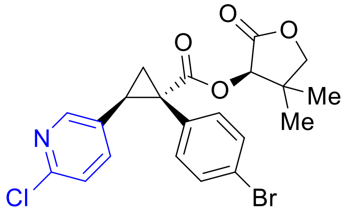

(*R*)-4,4-Dimethyl-2-oxotetrahydrofuran-3-yl-(1*S*,2*R*)-1-(4-bromophenyl)-2-(6-chloropyridin-3-yl) cyclopropane-1-carboxylate (8)
This compound was prepared according to **General procedure 3.1** from the reaction between **S17** (0.20 mmol, 71 mg) and **35** (2.0 equiv, 0.40 mmol, 56 mg). After isolation, product was obtained as a light yellow oil in 87% yield and 98% d.e as determined from the crude NMR (0.17 mmol, 81 mg).
^{1}H NMR (400 MHz, $CDCl_3$) δ 8.08 (d, *J* = 2.6 Hz, 1H), 7.40 – 7.31 (m, 2H), 7.05 (d, *J* = 8.3 Hz, 1H), 7.00 – 6.94 (m, 2H), 6.87 (dd, *J* = 8.3, 2.5 Hz, 1H), 5.33 (d, *J* = 6.9 Hz, 1H), 4.01 (s, 2H), 3.28 (dd, *J* = 9.4, 7.3 Hz, 1H), 2.31 (dd, *J* = 9.4, 5.4 Hz, 1H), 1.94 (dd, *J* = 7.3, 5.5 Hz, 1H), 1.19 (s, 3H), 0.89 (s, 3H).
^{13}C NMR (151 MHz, $CDCl_3$) δ 171.8, 171.4, 149.9, 149.7, 137.3, 133.3, 132.1, 131.6, 130.7, 123.5, 122.2, 40.1, 36.9, 29.7, 22.9, 20.8, 19.6.
HRMS: (+p APCI) calculated for $[C_{21}H_{20}O_4N^{79}Br^{35}Cl+]$ 464.0259, found 464.0258
IR(neat): 2970, 1786, 1728, 1587, 1560, 1490, 1464, 1397, 1370, 1352, 1296, 1237, 1216, 1152, 1091, 1071, 1012, 997, 976, 943, 910, 837, 730, 648, 580, 548, 525 cm^{-1}

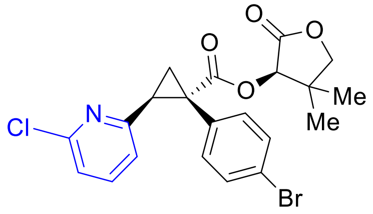

(*R*)-4,4-dimethyl-2-oxotetrahydrofuran-3-yl (1*S*,2*S*)-1-(4-bromophenyl)-2-(6-chloropyridin-2-yl)cyclopropane -1-carboxylate (9)
This compound was prepared according to **General procedure 3.1** from the reaction between **S17** (0.20 mmol, 71 mg) and **66** (2.0 equiv, 0.40 mmol, 56 mg). After isolation, product was obtained as a clear colorless oil in 90% yield and 98% d.e as determined from the crude NMR (0.18 mmol, 84 mg)
^{1}H NMR (400 MHz, $CDCl_3$) δ 7.40 (t, *J* = 7.8 Hz, 1H), 7.32 – 7.22 (m, 2H), 7.01 (dd, *J* = 7.9, 0.8 Hz, 1H), 6.99 – 6.93 (m, 2H), 6.88 (dd, *J* = 7.7, 0.8 Hz, 1H), 5.32 (d, *J* = 5.3 Hz, 1H), 3.99 (d, *J* = 1.4 Hz, 2H), 3.44 (dd, *J* = 9.0, 7.1 Hz, 1H), 2.37 (dd, *J* = 7.1, 4.9 Hz, 1H), 2.26 – 2.16 (m, 1H), 1.17 (s, 3H), 0.88 (s, 3H).
^{13}C NMR (151 MHz, $CDCl_3$) δ 171.9, 171.5, 156.3, 150.4, 138.4, 133.2, 132.8, 130.9, 122.1, 121.7, 121.5, 76.1, 75.9, 40.1, 37.4, 33.9, 22.9, 20.3, 19.6.
HRMS: (+p APCI) calculated for $[C_{21}H_{20}O_4N^{79}Br^{35}Cl +]$ 464.0259, found 464.0262
IR(neat): 2967, 1788, 1729, 1584, 1559, 1490, 1464, 1435, 1399, 1377, 1298, 1244, 1152, 1095, 1072, 1031, 1011, 996, 910, 827, 827, 798, 762, 732, 649, 532 cm^{-1}

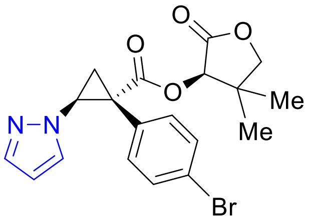

(*R*)-4,4-dimethyl-2-oxotetrahydrofuran-3-yl (1*R*,2*S*)-1-(4-bromophenyl)-2-(*1H*-pyrazol-1-yl)cyclopropane-1-carboxylate (10)
This compound was prepared according to **General procedure 3.1** from the reaction between **S17** (0.20 mmol, 71 mg) and ***N*-vinyl-pyrazole** (2.0 equiv, 0.40 mmol, 56 mg, Enamine). After isolation, product was obtained as a white waxy solid in 66% yield and 97% d.e as determined from the crude NMR (0.13 mmol, 55 mg).
MP: 142-144 ℃
^{1}H NMR (400 MHz, $CDCl_3$) δ 7.34 (t, *J* = 1.1 Hz, 1H), 7.29 – 7.19 (m, 3H), 7.17 (d, *J* = 2.4 Hz, 1H), 7.11 – 6.94 (m, 2H), 6.06 (t, *J* = 2.1 Hz, 1H), 5.31 (s, 1H), 4.79 – 4.65 (m, 1H), 3.98 (d, *J* = 1.1 Hz, 2H), 2.48 (d, *J* = 6.1 Hz, 1H), 2.41 – 2.31 (m, 1H), 1.17 (s, 3H), 0.85 (s, 3H).
^{13}C NMR (151 MHz, $CDCl_3$) δ 171.6, 170.4, 140.1, 132.5, 131.4, 131.0, 128.9, 122.1, 106.2, 76.0, 45.9, 40.0, 35.4, 22.9, 19.6, 19.4.
HRMS: (+p APCI) calculated for [$C_{19}H_{20}O_4N_2{}^{79}Br$ +] 419.0601, found 419.0600
IR(neat): 2967, 2930, 1789, 1731, 1518, 1491, 1465, 1398, 1378, 1347, 1251, 1199, 1153, 1098, 1071, 1032, 1012, 996, 859, 762, 733, 721, 647, 614, 543 cm^{-1}

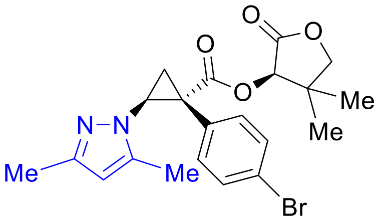

(*R*)-4,4-Dimethyl-2-oxotetrahydrofuran-3-yl (1*R*,2*S*)-1-(4-bromophenyl)-2-(3,5-dimethyl-*1H*-pyrazol-1-yl)cyclopropane-1-carboxylate (11)
This compound was prepared according to **General procedure 3.1** from the reaction between **S17** (0.20 mmol, 71 mg) and **S31** (2.0 equiv, 0.40 mmol, 49 mg). After isolation, product was obtained as a crystalline white solid in 97% yield and 98% d.e as determined from the crude NMR (0.19 mmol, 87 mg).
MP: 132-138 ℃
^{1}H NMR (400 MHz, $CDCl_3$) δ 7.31 – 7.19 (d, *J* = 8.5 Hz, 2H), 6.99 (d, *J* = 8.5 Hz, 2H), 5.68 (s, 1H), 5.37 (s, 1H), 4.43 (dd, *J* = 8.9, 5.6 Hz, 1H), 4.02 (s, 2H), 2.97 (t, *J* = 5.7 Hz, 1H), 2.41 – 2.31 (m, 3H), 2.29 (dd, *J* = 8.9, 5.9 Hz, 1H), 1.92 (s, 3H), 1.20 (s, 3H), 0.99 (s, 3H).
^{13}C NMR (151 MHz, $CDCl_3$) δ 171.9, 170.5, 147.1, 132.3, 131.3, 130.9, 121.8, 106.6, 76.1, 75.7, 44.0, 40.0, 35.2, 22.9, 19.8, 18.4, 13.1, 11.1.
HRMS: (+p APCI) calculated for [$C_{21}H_{24}O_4N_2{}^{79}Br$ +] 447.0914, found 447.0916
IR(neat): 2970, 1788, 1736, 1560, 1491, 1464, 1370, 1299, 1229, 1217, 1204, 1148, 1127, 1092, 1072, 1011, 997, 913, 851, 790, 763, 731, 719, 647, 578, 541, 528 cm^{-1}

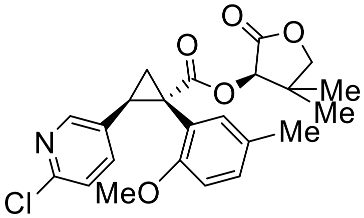

(*R*)-4,4-dimethyl-2-oxotetrahydrofuran-3-yl (*1S,2R*)-2-(6-chloropyridin-3-yl)-1-(2-methoxy-5-methylphenyl) cyclopropane-1-carboxylate (12)
This compound was prepared according to **General procedure 3.1** from the reaction between **S18** (0.20 mmol, 64 mg) and **35** (2.0 equiv, 0.40 mmol, 56 mg). After isolation the product was obtained as a white solid in 64% yield and 89% d.e as determined from the crude NMR (0.13 mmol, 86 mg)
^{1}H NMR (400 MHz, $CDCl_3$) δ 8.07 (d, J = 2.5 Hz, 1H), 7.06 (d, J = 2.3 Hz, 1H), 6.98 – 6.89 (m, 2H), 6.86 (dd, J = 8.3, 2.5 Hz, 1H), 6.41 (d, J = 8.3 Hz, 1H), 5.34 (s, 1H), 3.99 – 3.89 (m, 2H), 3.38 (s, 3H), 3.27 (dd, J = 9.3, 7.3 Hz, 1H), 2.25 (s, 3H), 2.09 (dd, J = 9.2, 5.4 Hz, 1H), 1.93 (dd, J = 7.3, 5.4 Hz, 1H), 1.13 (s, 3H), 0.74 (s, 3H);
^{13}C NMR (101 MHz, DMSO-d6) δ 172.9, 172.4, 156.1, 149.8, 148.3, 138.6, 132.4, 132.0, 129.9, 128.8, 122.8, 122.0, 110.0, 75.7, 75.6, 55.2, 40.2, 34.0, 29.0, 21.8, 20.5, 19.6, 19.1;
HRMS: (ESI) m/z calculated for $C_{23}H_{25}ClNO_5$ [M+H]+, 430.1427; found 430.1426.

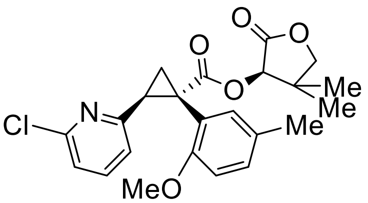

(*R*)-4,4-dimethyl-2-oxotetrahydrofuran-3-yl (1*S*,2*S*)-2-(6-chloropyridin-2-yl)-1-(2-methoxy-5-methylphenyl) cyclopropane-1-carboxylate (13)

This compound was prepared according to **General procedure 3.1** from the reaction between **S18** (0.20 mmol, 64 mg) and **66** (2.0 equiv, 0.40 mmol, 56 mg). After isolation the product was obtained as a white solid in 68% yield and 87% d.e as determined from the crude NMR (0.13 mmol, 92 mg)

^{1}H NMR (400 MHz, $CDCl_3$) δ 7.29 (d, J = 7.8 Hz, 1H), 7.10 (d, J = 2.3 Hz, 1H), 6.94 – 6.84 (m, 2H), 6.76 (dd, J = 7.8, 0.9 Hz, 1H), 6.35 (d, J = 8.3 Hz, 1H), 5.33 (s, 1H), 3.97 – 3.89 (m, 2H), 3.45 (dd, J = 9.0, 7.0 Hz, 1H), 3.37 (s, 3H), 2.34 (dd, J = 7.1, 4.9 Hz, 1H), 2.25 (s, 3H), 2.04 (dd, J = 9.0, 4.8 Hz, 1H), 1.13 (s, 3H), 0.76 (s, 3H);

^{13}C NMR (101 MHz, DMSO-d6) δ 172.8, 172.2, 157.1, 156.2, 148.8, 138.8, 132.7, 129.4, 128.4, 122.5, 122.2, 121.8, 109.8, 75.7, 75.6, 55.2, 40.3, 34.6, 33.0, 21.9, 20.5, 19.9, 19.2;

HRMS (ESI) m/z calculated for $C_{23}H_{25}ClNO_5$ [M+H]+, 430.1427; found 430.1430.

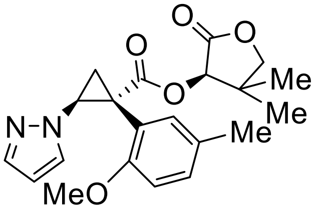

(*R*)-4,4-dimethyl-2-oxotetrahydrofuran-3-yl (1*R*,2*S*)-1-(2-methoxy-5-methylphenyl)-2-(*1H*-pyrazol-1-yl) cyclopropane-1-carboxylate (14)

This compound was prepared according to **General procedure 3.1** from the reaction between **S18** (0.20 mmol, 64 mg) and ***N*-vinyl pyrazole** (2.0 equiv, 0.40 mmol, 38 mg, Enamine). After isolation the product was obtained as a white foam in 69% yield and 89% d.e as determined from the crude NMR (0.14 mmol, 83 mg)

^{1}H NMR (400 MHz, $CDCl_3$) δ 7.24 (dd, J = 2.4, 0.7 Hz, 1H), 7.20 (dd, J = 1.8, 0.7 Hz, 1H), 6.94 – 6.90 (m, 2H), 6.54 – 6.49 (m, 1H), 6.00 (dd, J = 2.4, 1.8 Hz, 1H), 5.34 (s, 1H), 4.74 (dd, J = 8.7, 5.7 Hz, 1H), 4.01 – 3.88 (m, 2H), 3.67 (s, 3H), 2.64 (t, J = 6.0 Hz, 1H), 2.19 – 2.12 (m, 4H), 1.15 (s, 3H), 0.79 (s, 3H);

^{13}C NMR (101 MHz, $CDCl_3$) δ 172.0, 171.4, 156.3, 138.4, 132.1, 129.8, 129.6, 129.4, 120.7, 109.3, 105.5, 76.0, 75.4, 54.9, 45.2, 40.1, 32.6, 22.7, 20.4, 19.24, 19.21;

HRMS (ESI) m/z calculated for $C_{21}H_{25}N_2O_5$ [M+H]+, 385.1758; found 385.1767.

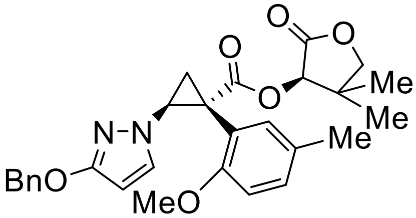

(*R*)-4,4-dimethyl-2-oxotetrahydrofuran-3-yl (1*R*,2*S*)-2-(3-(benzyloxy)-*1H*-pyrazol-1-yl)-1-(2-methoxy-5-methylphenyl)cyclopropane-1-carboxylate (15)

This compound was prepared according to **General procedure 3.1** from the reaction between **S18** (0.20 mmol, 64 mg) and **S32** (2.0 equiv, 0.40 mmol, 80 mg). After isolation the product was obtained as a clear oil in 58% yield and 87% d.e as determined from the crude NMR (0.12 mmol, 89 mg)

^{1}H NMR (400 MHz, $CDCl_3$) δ 7.38 – 7.26 (m, 5H), 7.02 (d, J = 2.5 Hz, 1H), 6.99 – 6.91 (m, 2H), 6.54 (d, J = 8.1 Hz, 1H), 5.43 (d, J = 2.5 Hz, 1H), 5.33 (s, 1H), 5.00 (d, J = 11.9 Hz, 1H), 4.89 (d, J = 12.0 Hz, 1H), 4.58 (dd, J = 8.7, 5.7 Hz, 1H), 3.94 (d, J = 2.2 Hz, 2H), 3.66 (s, 3H), 2.54 (t, J = 5.9 Hz, 1H), 2.18 (s, 3H), 2.10 (dd, J = 8.7, 6.1 Hz, 1H), 1.15 (s, 3H), 0.79 (s, 3H);

^{13}C NMR (101 MHz, $CDCl_3$) δ 172.0, 171.4, 162.0, 156.5, 137.2, 132.2, 131.2, 129.5, 129.2, 128.3, 127.8, 127.7, 121.0, 109.4, 91.4, 76.0, 75.3, 70.7, 54.9, 45.5, 40.1, 32.5, 22.7, 20.4, 19.2, 19.1;

HRMS (ESI) m/z calculated for $C_{28}H_{31}N_2O_6$ [M+H]+, 491.2177; found 491.2184.

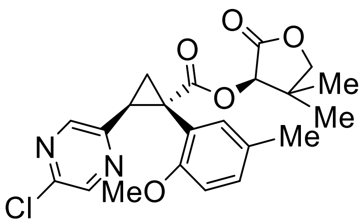

(*R*)-4,4-dimethyl-2-oxotetrahydrofuran-3-yl (1*S*,2*S*)-2-(5-chloropyrazin-2-yl)-1-(2-methoxy-5-methylphenyl) cyclopropane-1-carboxylate (16)

This compound was prepared according to **General procedure 3.1** from the reaction between **S18** (0.20 mmol, 64 mg) and **2-chloro-5-ethenyl-pyrimidine** (2.0 equiv, 0.40 mmol, 56 mg, Enamine). After isolation the product was obtained as a white foam in 41% yield and 92% d.e as determined from the crude NMR (0.08 mmol, 56 mg)

^{1}H NMR (400 MHz, $CDCl_3$) δ 8.24 (d, J = 1.4 Hz, 1H), 8.04 (d, J = 1.3 Hz, 1H), 7.10 (d, J = 2.2 Hz, 1H), 6.91 (ddd, J = 8.2, 2.3, 0.9 Hz, 1H), 6.35 (d, J = 8.3 Hz, 1H), 5.33 (s, 1H), 4.01 – 3.89 (m, 2H), 3.47 (dd, J = 8.9, 7.0 Hz, 1H), 3.41 (s, 3H), 2.43 (dd, J = 7.0, 4.7 Hz, 1H), 2.25 (s, 3H), 2.07 (dd, J = 8.9, 4.7 Hz, 1H), 1.13 (s, 3H), 0.74 (s, 3H);

^{13}C NMR (101 MHz, $CDCl_3$) δ 172.1, 172.0, 155.4, 150.7, 146.4, 144.7, 142.0, 133.0, 129.6, 129.5, 121.5, 108.9, 76.1, 75.5, 53.6, 40.2, 34.9, 30.4, 22.7, 20.5, 20.0, 19.2;

HRMS (ESI) m/z calculated for $C_{22}H_{24}ClN_2O_5$ [M+H]+, 431.1368; found 431.1377.

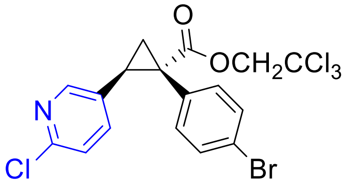

2,2,2-trichloroethyl (1*S*,2*R*)-1-(4-bromophenyl)-2-(6-chloropyridin-3-yl)cyclopropane-1-carboxylate (17)

This compound was prepared according to **General procedure 3.2** from the reaction between **s1** (0.20 mmol, 74 mg) and **35** (2.32 equiv, 0.46 mmol, 65 mg) with $(MeO)_2CO$ as solvent in 77% yield and >99% ee (0.15 mmol, 75 mg). After isolation, enantio-enriched product was obtained as an off-white greasy crystalline solid.

MP: 136-137 ℃

^{1}H NMR (600 MHz, $CDCl_3$) δ 8.05 (d, *J* = 2.5 Hz, 1H), 7.36 – 7.30 (m, 2H), 7.03 (d, *J* = 8.3 Hz, 1H), 6.96 – 6.90 (m, 2H), 6.83 (dd, *J* = 8.4, 2.5 Hz, 1H), 4.84 (d, *J* = 11.9 Hz, 1H), 4.64 (d, *J* = 11.9 Hz, 1H), 3.18 (dd, *J* = 9.4, 7.3 Hz, 1H), 2.34 (dd, *J* = 9.4, 5.5 Hz, 1H), 1.93 (dd, *J* = 7.3, 5.5 Hz, 1H).

^{13}C NMR (151 MHz, $CDCl_3$) δ 171.1, 150.2, 149.9, 137.4, 133.7, 131.9, 131.7, 130.7, 123.7, 122.5, 94.9, 74.8, 37.0, 30.3, 20.5.

HRMS: (+p APCI) calculated for [$C_{17}H_{13}O_2N^{79}Br^{35}Cl_4$+] 481.8878, found 481.8878

IR(neat): 2954, 1737, 1587, 1561, 1490, 1464, 1396, 1367, 1349, 1237, 1210, 1156, 1111, 1071, 1057, 1024, 1011, 973, 909, 836, 801, 767, 740, 717, 646, 575, 521 cm^{-1}

Chiral HPLC: (OD-H, 60 min, 1 mL/min, 1% *i*-PrOH in *n*-hexane, UV 230 nm) RT: 30.65 min, 38.71 min.

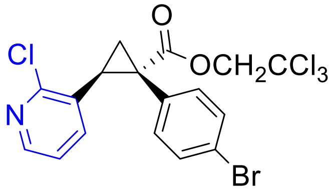

2,2,2-trichloroethyl (1*R*,2*R*)-1-(4-bromophenyl)-2-(2-chloropyridin-3-yl)cyclopropane-1-carboxylate (18)

This compound was prepared according to **General procedure 3.2** from the reaction between **s1** (0.20 mmol, 74 mg) and **S25** (2.32 equiv, 0.46 mmol, 65 mg) with $(MeO)_2CO$ as solvent in 70% yield and >99% ee (0.14 mmol, 68 mg)**.** After isolation, enantio-enriched product was obtained as a clear colorless oil.

^{1}H NMR (400 MHz, $CDCl_3$) δ 8.15 (s, 1H), 7.32 – 7.19 (m, 3H), 7.15 – 7.01 (m, 2H), 6.92 (s, 1H), 6.86 (dd, *J* = 7.7, 1.7 Hz, 1H), 4.90 (d, *J* = 11.9 Hz, 1H), 4.67 (d, *J* = 11.9 Hz, 1H), 3.47 (dd, *J* = 9.1, 7.6 Hz, 1H), 2.31 (dd, *J* = 9.2, 5.5 Hz, 1H), 2.07 (dd, *J* = 7.6, 5.5 Hz, 1H).

^{13}C NMR (151 MHz, $CDCl_3$) δ 170.9, 153.2, 147.9, 135.9, 132.8, 132.2, 131.2, 130.5, 122.0, 121.9, 94.7, 74.4, 36.0, 31.2, 18.2.

HRMS: (+p APCI) calculated for [$C_{17}H_{13}O_2N^{79}Br^{35}Cl_4$+] 481.8878, found 481.8884

IR(neat): 3016, 2770, 1737, 1564, 1490, 1440, 1409, 1367, 1234, 1193, 1156, 1131, 1091, 1071, 1011, 909, 818, 809, 767, 751, 716, 677, 575, 521 cm^{-1}

Chiral HPLC: (OD-H, 30 min, 1 mL/min, 1% *i*-PrOH in *n*-hexane, UV 230 nm) RT: 33.41 min, 39.16 min.

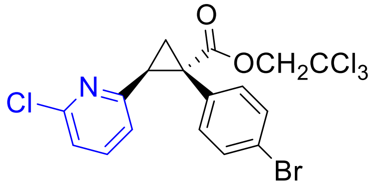

2,2,2-trichloroethyl (1*S*,2*S*)-1-(4-bromophenyl)-2-(6-chloropyridin-2-yl)cyclopropane-1-carboxylate (19)
This compound was prepared according **General procedure 3.2** from the reaction between **s1** (0.20 mmol, 74 mg) and **66** (2.32 equiv, 0.46 mmol, 65 mg) with CH_2Cl_2 as solvent in 53% yield and 93% ee (0.11 mmol, 51 mg). After isolation, enantio-enriched product was obtained as a yellow oil.
^{1}H NMR (400 MHz, $CDCl_3$) δ 7.40 (t, *J* = 7.8 Hz, 1H), 7.33 – 7.23 (m, 2H), 7.03 (d, *J* = 7.9 Hz, 1H), 6.98 (d, *J* = 6.5 Hz, 1H), 6.83 (d, *J* = 7.6 Hz, 1H), 4.85 (d, *J* = 11.9 Hz, 1H), 4.64 (d, *J* = 11.9 Hz, 1H), 3.40 (dd, *J* = 9.0, 7.1 Hz, 1H), 2.38 (dd, *J* = 7.1, 4.9 Hz, 1H), 2.28 (dd, *J* = 9.0, 4.9 Hz, 1H).
^{13}C NMR (151 MHz, $CDCl_3$) δ 170.9, 156.1, 150.5, 138.3, 133.3, 132.4, 132.0, 130.8, 129.2, 122.1, 121.6, 121.4, 94.7, 74.5, 37.3, 34.2, 19.7.
HRMS: (+p APCI) calculated for $[C_{17}H_{13}O_2N^{79}Br^{35}Cl_4+]$ 481.8878, found 481.8881
IR(neat): 2953, 1736, 1584, 1559, 1490, 1434, 1408, 1396, 1378, 1238, 1155, 1096, 1071, 1012, 990, 909, 831, 798, 766, 738, 717, 672, 574, 539 cm^{-1}
Chiral HPLC: (AD-H, 30 min, 1 mL/min, 1% *i*-PrOH in *n*-hexane, UV 230 nm) RT: 10.71 min, 14.49 min.

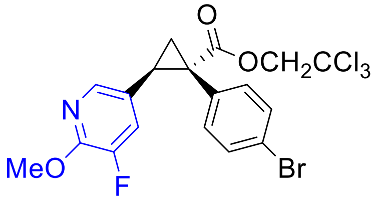

2,2,2-trichloroethyl (1*S*,2*R*)-1-(4-bromophenyl)-2-(5-fluoro-6-methoxypyridin-3-yl)cyclopropane-1-carboxylate (20)
This compound was prepared according **General procedure 3.2** from the reaction between **s1** (0.20 mmol, 74 mg) and **S26** (2.32 equiv, 0.46 mmol, 71 mg) with CH_2Cl_2 as solvent in 72% yield and 98% ee (0.14 mmol, 72 mg). After isolation, enantio-enriched product was obtained as a clear colorless oil.
^{1}H NMR (400 MHz, $CDCl_3$) δ 7.59 (d, *J* = 2.0 Hz, 1H), 7.39 – 7.28 (m, 2H), 7.04 – 6.88 (m, 2H), 6.58 (dd, *J* = 11.0, 2.1 Hz, 1H), 4.96 – 4.75 (m, 1H), 4.63 (dd, *J* = 11.9, 0.7 Hz, 1H), 3.94 (d, *J* = 0.9 Hz, 3H), 3.14 (dd, *J* = 9.5, 7.3 Hz, 1H), 2.35 – 2.22 (m, 1H), 1.87 (dd, *J* = 7.3, 5.4 Hz, 1H).
^{13}C NMR (151 MHz, $CDCl_3$) δ 171.1, 152.3 (d, *J* = 11.4 Hz), 147.6, 145.9, 140.9 (d, *J* = 5.7 Hz), 133.4, 132.1, 131.3, 125.2, 122.1, 122.0, 122.0, 94.8, 74.4, 53.7, 36.3, 30.2, 20.0.
^{19}F NMR (376 MHz, $CDCl_3$) δ -139.93 (d, *J* = 10.9 Hz).
HRMS: (+p APCI) calculated for $[C_{18}H_{15}O_3N^{79}Br^{35}Cl_3F +]$ 495.9279, found 495.9284
IR(neat): 1732, 1617, 1577, 1495, 1443, 1412, 1382, 1315, 1198, 1140, 1197, 1162, 1140, 1091, 1071, 1057, 1011, 970, 907, 850, 827, 806, 779, 766, 718, 649, 622, 573, 546, 526, 500 cm^{-1}
Chiral HPLC: (OD-H, 30 min, 1 mL/min, 1% *i*-PrOH in *n*-hexane, UV 230 nm) RT: 14.14 min, 23.37 min.

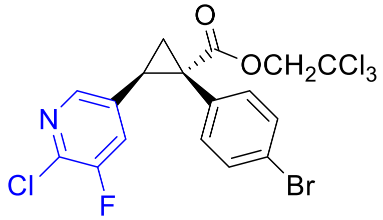

2,2,2-trichloroethyl (1*S*,2*R*)-1-(4-bromophenyl)-2-(6-chloro-5-fluoropyridin-3-yl)cyclopropane-1-carboxylate (21)
This compound was prepared according to **General procedure 3.2** from the reaction between **s1** (0.20 mmol, 74 mg) and **S27** (2.32 equiv, 0.46 mmol, 73 mg) with CH_2Cl_2 as solvent in 65% yield and 98% ee (0.13 mmol, 65 mg). After isolation, enantio-enriched product was obtained as a white solid.
MP: 109-116 ℃
^{1}H NMR (400 MHz, $CDCl_3$) δ 7.87 (d, *J* = 2.1 Hz, 1H), 7.44 – 7.30 (m, 2H), 7.04 – 6.91 (m, 2H), 6.69 (dd, *J* = 9.0, 2.1 Hz, 1H), 4.84 (d, *J* = 11.9 Hz, 1H), 4.64 (d, *J* = 11.8 Hz, 1H), 3.20 (dd, *J* = 9.4, 7.2 Hz, 1H), 2.36 (dd, *J* = 9.4, 5.5 Hz, 1H), 1.92 (dd, *J* = 7.3, 5.5 Hz, 1H).
^{13}C NMR (151 MHz, $CDCl_3$) δ 170.6, 155.0, 153.2, 144.5 (d, *J* = 5.1 Hz), 137.5, 137.4, 133.3, 131.6, 131.3, 123.1, 123.0, 122.5, 94.6, 74.6, 37.0, 29.6, 20.5.
^{19}F NMR (376 MHz, $CDCl_3$) δ -119.18 (d, *J* = 8.9 Hz).
HRMS: (+p APCI) calculated for $[C_{17}H_{12}O_2N^{79}Br^{35}Cl_4F +]$ 499.8784, found 499.8786
IR(neat): 2955, 1736, 1594, 1568, 1490, 1414, 1381, 1241, 1187, 1152, 1071, 1059, 1012, 970, 904, 828, 807, 767, 728, 717, 573, 542 cm^{-1}

Chiral HPLC: (OD-H, 60 min, 1 mL/min, 1% *i*-PrOH in *n*-hexane, UV 230 nm) RT: 25.63 min, 39.39 min.

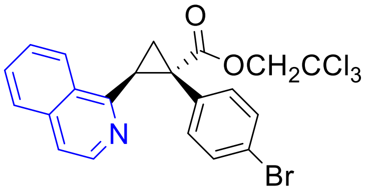

2,2,2-trichloroethyl (1*S*,2*S*)-1-(4-bromophenyl)-2-(isoquinolin-1-yl)cyclopropane-1-carboxylate (22)
This compound was prepared according to **General procedure 3.2** from the reaction between **s1** (0.20 mmol, 74 mg) and **S29** (2.32 equiv, 0.46 mmol, 72 mg) with CH_2Cl_2 as solvent in 54% yield and 83% ee (0.11 mmol, 54 mg). After isolation, enantio-enriched product was obtained as a yellow solid.
MP: 124-127 ℃
^{1}H NMR (400 MHz, $CDCl_3$) δ 8.39 (d, *J* = 8.0 Hz, 1H), 8.11 (d, *J* = 5.7 Hz, 1H), 7.79 (d, *J* = 7.5 Hz, 1H), 7.70 (dd, *J* = 8.7, 6.8 Hz, 2H), 7.47 – 7.30 (m, 1H), 7.15 – 6.96 (m, 2H), 6.88 – 6.68 (m, 2H), 4.96 (d, *J* = 11.9 Hz, 1H), 4.74 (d, *J* = 11.9 Hz, 1H), 4.03 (dd, *J* = 8.9, 7.0 Hz, 1H), 2.98 (s, 1H), 2.31 (dd, *J* = 8.9, 4.4 Hz, 1H).
^{13}C NMR (151 MHz, $CDCl_3$) δ 171.4, 153.8, 141.3, 135.9, 132.6(2 C), 132.6, 130.5(2 C), 130.1, 128.8, 127.7, 127.5, 124.4, 121.3, 119.9, 95.0, 74.3, 37.3, 31.9, 18.4.
HRMS: (+p APCI) calculated for $[C_{21}H_{16}O_2N^{79}Br^{35}Cl_3+]$ 497.9424, found 497.9415
IR(neat): 1733, 1623, 1585, 1563, 1491, 1407, 1396, 1367, 1311, 1272, 1237, 1197, 1170, 1150, 1095, 1059, 1011, 972, 906, 825, 799, 767, 730, 649, 573, 532 cm^{-1}
Chiral HPLC: (AD-H, 30 min, 1 mL/min, 1% *i*-PrOH in *n*-hexane, UV 230 nm) RT: 15.62 min, 19.25 min.

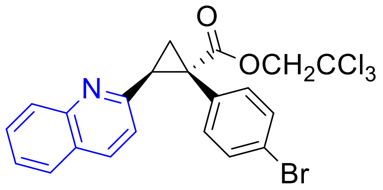

2,2,2-trichloroethyl (1*S*,2*S*)-1-(4-bromophenyl)-2-(quinolin-2-yl)cyclopropane-1-carboxylate (23)
This compound was prepared according to **General procedure 3.2** from the reaction between **s1** (0.20 mmol, 74 mg) and **S28** (2.32 equiv, 0.46 mmol, 72 mg) with CH_2Cl_2 as solvent in 56% yield and 87% ee (0.11 mmol, 56 mg). After isolation, enantio-enriched product was obtained as a light yellow oil.
^{1}H NMR (400 MHz, $CDCl_3$) δ 7.88 (dd, *J* = 8.5, 0.8 Hz, 1H), 7.83 – 7.77 (m, 1H), 7.68 (dd, *J* = 8.2, 1.4 Hz, 1H), 7.61 (ddd, *J* = 8.5, 6.9, 1.5 Hz, 1H), 7.44 (ddd, *J* = 8.1, 6.9, 1.2 Hz, 1H), 7.20 – 7.08 (m, 2H), 7.02 – 6.97 (m, 2H), 6.95 (d, *J* = 8.5 Hz, 1H), 4.85 (d, *J* = 11.9 Hz, 1H), 4.66 (d, *J* = 11.9 Hz, 1H), 3.59 (dd, *J* = 9.1, 7.2 Hz, 1H), 2.56 (dd, *J* = 7.2, 4.8 Hz, 1H), 2.35 (dd, *J* = 9.1, 4.8 Hz, 1H).
^{13}C NMR (151 MHz, $CDCl_3$) δ 171.2, 155.3, 147.5, 135.7, 133.3, 132.8, 130.8, 129.5, 128.9, 127.4, 126.6, 126.1, 121.4, 120.8, 94.8, 74.5, 37.5, 35.4, 20.1.
HRMS: (+p APCI) calculated for $[C_{21}H_{16}O_2N^{79}Br^{35}Cl_3+]$ 497.9425, found 497.9428
IR(neat): 2952, 1736, 1618, 1598, 1505, 1489, 1426, 1366, 1237, 1192, 1155, 1094, 1071, 1012, 988, 909, 834, 758, 732, 719, 575, 527 cm^{-1}
Chiral HPLC: (AD-H, 90 min, 1 mL/min, 1% *i*-PrOH in *n*-hexane, UV 230 nm) RT: 17.42 min, 54.21 min.

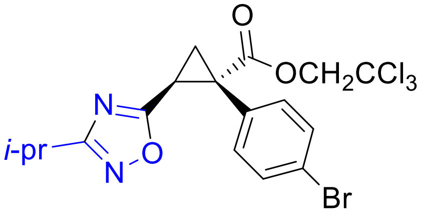

2,2,2-trichloroethyl (1*S*,2*R*)-1-(4-bromophenyl)-2-(3-Isopropyl-5-vinyl-1,2,4-oxadiazole)cyclopropane-1-carboxylate (24)
This compound was prepared according to **General procedure 3.2** from the reaction between **s1** (0.20 mmol, 74 mg) and **S30** (2.32 equiv, 0.46 mmol, 64 mg) with CH_2Cl_2 as solvent in 96% yield and 89% ee (0.19 mmol, 93 mg). After isolation, enantio-enriched product was obtained as a clear colorless oil.
^{1}H NMR (400 MHz, $CDCl_3$) δ 7.34 (d, *J* = 8.4 Hz, 2H), 7.04 (d, *J* = 8.5 Hz, 2H), 4.83 (d, *J* = 11.9 Hz, 1H), 4.64 (d, *J* = 11.9 Hz, 1H), 3.40 (dd, *J* = 9.0, 6.8 Hz, 1H), 2.87 (hept, *J* = 6.9 Hz, 1H), 2.42 – 2.30 (two overlapped signals, m, 2H), 1.10 (dd, *J* = 6.9, 2.0 Hz, 6H).
^{13}C NMR (151 MHz, $CDCl_3$) δ 174.9, 174.5, 169.8, 132.4, 131.7, 131.3, 122.4, 94.4, 74.7, 36.9, 26.4, 23.6, 20.4, 20.1, 19.9.
HRMS: (+p APCI) calculated for $[C_{17}H_{17}O_3N_2{}^{79}Br^{35}Cl_3 +]$ 480.9482, found 480.9484
IR(neat): 2970, 1739, 1588, 1489, 1435, 1366, 1230, 1217, 1158, 1094, 1070, 1012, 969, 909, 830, 809, 785, 767, 717, 574, 543, 528 cm^{-1}
Chiral HPLC: (OJ-H, 30 min, 1 mL/min, 1% *i*-PrOH in *n*-hexane, UV 230 nm) RT: 12.63 min, 15.08 min.

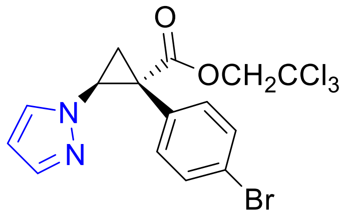

2,2,2-trichloroethyl (1*R*,2*S*)-1-(4-bromophenyl)-2-(1*H*-pyrazol-1-yl)cyclopropane-1-carboxylate (25)
This compound was prepared according to **General procedure 3.2** from the reaction between **s1** (0.20 mmol, 74 mg) and ***N*-vinyl pyrazole** (2.32 equiv, 0.46 mmol, 44 mg) purchased from Enamine with CH_2Cl_2 as solvent. The reaction had to be conducted with elevated catalyst loading (1.0 mol % Rh_2(*R*-*p*-Ph-TPCP)$_4$, 2.0 µmol, 2.6 mg) and longer reaction time (48 h) to achieve high yield and selectivity, 80% yield and 95% ee (0.16 mmol, 70 mg). After isolation, product was obtained as a white solid.
MP: 90-93℃
^{1}H NMR (400 MHz, $CDCl_3$) δ 7.36 (d, *J* = 1.8 Hz, 1H), 7.33 – 7.26 (m, 2H), 7.17 (s, 1H), 7.11 – 6.99 (m, 2H), 6.09 (t, *J* = 2.1 Hz, 1H), 4.89 – 4.78 (m, 1H), 4.72 (dd, *J* = 8.8, 5.8 Hz, 1H), 4.66 (d, *J* = 11.9 Hz, 1H), 2.53 (t, *J* = 6.0 Hz, 1H), 2.42 (dd, *J* = 8.8, 6.3 Hz, 1H).
^{13}C NMR (151 MHz, $CDCl_3$) δ 169.8, 140.1, 132.6, 131.7, 131.0, 128.8, 128.3, 122.2, 106.3, 94.6, 74.7, 74.5, 46.0, 35.5, 18.8.
HRMS: (+p APCI) calculated for $[C_{15}H_{13}O_2N_2{}^{79}Br^{35}Cl_3+]$ 436.9220, found 436.9220
IR(neat): 1735, 1593, 1517, 1489, 1448, 1395, 1330, 1242, 1156, 1097, 1071, 1011, 986, 911, 858, 827, 808, 767, 718, 667, 641, 612, 574, 491 cm^{-1}
Chiral HPLC: (OJ-H, 45 min, 1 mL/min, 1% *i*-PrOH in *n*-hexane, UV 230 nm) RT: 22.60 min, 18.87 min.

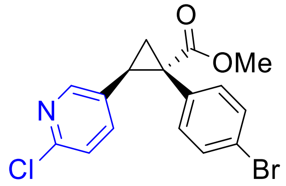

Methyl (1*S*,2*R*)-1-(4-bromophenyl)-2-(6-chloropyridin-3-yl)cyclopropane-1-carboxylate (26)
This compound was prepared according to **General procedure 3.2** from the reaction between **s2** (0.20 mmol, 51 mg) and **35** (2.32 equiv, 0.46 mmol, 65 mg) with CH_2Cl_2 as solvent in 70% yield and 94% ee (0.14 mmol, 51 mg). After isolation, enantio-enriched product was obtained as a white waxy solid.
MP: 152-156℃
^{1}H NMR (400 MHz, $CDCl_3$) δ 8.06 (d, *J* = 2.6 Hz, 1H), 7.37 – 7.31 (m, 2H), 7.03 (d, *J* = 8.4 Hz, 1H), 6.96 – 6.84 (m, 2H), 6.80 (dd, *J* = 8.3, 2.6 Hz, 1H), 3.70 (s, 3H), 3.11 (dd, *J* = 9.3, 7.2 Hz, 1H), 2.23 (dd, *J* = 9.4, 5.2 Hz, 1H), 1.82 (dd, *J* = 7.2, 5.2 Hz, 1H).
^{13}C NMR (151 MHz, $CDCl_3$) δ 173.0, 149.7, 149.6, 137.1, 133.4, 132.7, 131.4, 131.1, 123.3, 121.9, 52.9, 37.0, 29.4, 20.3.
HRMS: (+p APCI) calculated for $[C_{16}H_{14}O_2N^{79}Br^{35}Cl+]$ 365.9891, found 365.9894
IR(neat): 2951, 1716, 1587, 1560, 1489, 1464, 1434, 1395, 1349, 1258, 1211, 1193, 1163, 1142, 1111, 1080, 1025, 1011, 967, 910, 863, 834, 801, 766, 739, 647, 629, 573, 522 cm^{-1}
Chiral HPLC: (AD-H, 60 min, 1 mL/min, 1% *i*-PrOH in *n*-hexane, UV 230 nm) RT: 37.89 min, 46.52 min.

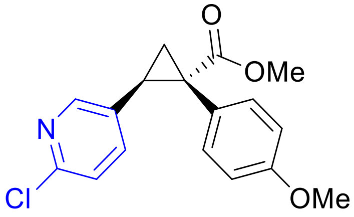

Methyl (1*S*,2*R*)-2-(6-chloropyridin-3-yl)-1-(4-methoxyphenyl)cyclopropane-1-carboxylate (27)
This compound was prepared according to **General procedure 3.2** from the reaction between **S7** (0.20 mmol, 41 mg) and **35** (2.32 equiv, 0.46 mmol, 65 mg) with CH_2Cl_2 as solvent in 70% yield and 89% ee (0.14 mmol, 45 mg). After isolation, enantio-enriched product was obtained as a clear colorless oil.
^{1}H NMR (400 MHz, $CDCl_3$) δ 8.01 (d, *J* = 2.5 Hz, 1H), 6.97 (d, *J* = 8.3 Hz, 1H), 6.95 – 6.90 (m, 2H), 6.75 (dd, *J* = 8.4, 2.6 Hz, 1H), 6.73 – 6.66 (m, 2H), 3.75 (s, 3H), 3.67 (s, 3H), 3.04 (dd, *J* = 9.3, 7.1 Hz, 1H), 2.18 (dd, *J* = 9.3, 5.1 Hz, 1H), 1.78 (dd, *J* = 7.1, 5.1 Hz, 1H), 1.57 (s, 3H).
^{13}C NMR (151 MHz, $CDCl_3$) δ 174.1, 159.0, 149.9, 149.5, 137.4, 133.0, 132.0, 125.7, 123.3, 113.9, 55.3, 53.0, 37.1, 29.7, 21.0.
HRMS: (+p APCI) calculated for $[C_{17}H_{17}O_3N^{35}Cl +]$ 318.0891, found 318.0885
IR(neat): 2952, 1717, 1612, 1584, 1560, 1516, 1463, 1435, 1349, 1264, 1247, 1211, 1176, 1163, 1109, 1031, 967, 835, 803, 756, 745, 653, 632, 604, 537, cm^{-1}

Chiral HPLC: (AD-H, 30 min, 1 mL/min, 3% *i*-PrOH in *n*-hexane, UV 230 nm) RT: 19.34 min, 21.92 min.

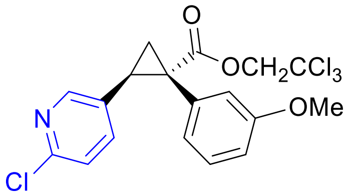

2,2,2-trichloroethyl (1*S*,2*R*)-2-(6-chloropyridin-3-yl)-1-(3-methoxyphenyl)cyclopropane-1-carboxylate (28)
This compound was prepared according to **General procedure 3.2** from the reaction between **S8** (0.20 mmol, 65 mg) and **35** (2.32 equiv, 0.46 mmol, 65 mg) with CH_2Cl_2 as solvent in 65% yield and 95% ee (0.13 mmol, 55 mg). After isolation, enantio-enriched product was obtained as a clear colorless oil.
^{1}H NMR (400 MHz, $CDCl_3$) δ 8.05 (d, *J* = 2.6 Hz, 1H), 7.10 (t, *J* = 7.9 Hz, 1H), 6.99 (d, *J* = 8.3 Hz, 1H), 6.83 (dd, *J* = 8.3, 2.6 Hz, 1H), 6.73 (ddt, *J* = 8.3, 2.6, 0.8 Hz, 1H), 6.65 (ddt, *J* = 7.7, 1.6, 0.7 Hz, 1H), 6.63 – 6.56 (m, 1H), 4.86 (dd, *J* = 12.0, 0.6 Hz, 1H), 4.63 (dd, *J* = 11.9, 0.6 Hz, 1H), 3.67 (d, *J* = 0.5 Hz, 3H), 3.16 (dd, *J* = 9.4, 7.2 Hz, 1H), 2.47 – 2.16 (m, 1H), 1.95 (dd, *J* = 7.2, 5.3 Hz, 1H).
^{13}C NMR (151 MHz, $CDCl_3$) δ 171.5, 159.5, 149.9, 149.8, 137.4, 134.1, 131.2, 129.4, 124.5, 123.4, 117.8, 113.7, 95.0, 74.6, 55.3, 37.6, 30.2, 20.7.
HRMS: (+p APCI) calculated for $[C_{18}H_{16}O_3N^{35}Cl_4+]$ 433.9878, found 433.9881
IR(neat): 2954, 1733, 1601, 1585, 1561, 1494, 1463, 1435, 1367, 1347, 1288, 1238, 1208, 1153, 1110, 1046, 978, 910, 803, 753, 736, 713, 700, 573 cm^{-1}
Chiral HPLC: (AD-H, 30 min, 1 mL/min, 5% *i*-PrOH in *n*-hexane, UV 230 nm) RT: 12.15 min, 14.45 min.

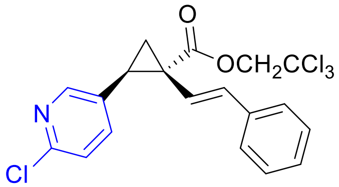

2,2,2-trichloroethyl (1*R*,2*R*)-2-(6-chloropyridin-3-yl)-1-((E)-styryl)cyclopropane-1-carboxylate (29)
This compound was prepared according to **General procedure 3.2** from the reaction between **S9** (0.20 mmol, 64 mg) and **35** (2.32 equiv, 0.46 mmol, 65 mg) with CH_2Cl_2 as solvent in 67% yield and 98% ee (0.13 mmol, 57 mg). After isolation, enantio-enriched product was obtained as a yellow oil.
^{1}H NMR (400 MHz, $CDCl_3$) δ 8.28 (s, 1H), 7.38 (dd, *J* = 8.3, 2.2 Hz, 1H), 7.33 – 7.25 (m, 3H), 7.25 – 7.11 (m, 3H), 6.45 (d, *J* = 16.0 Hz, 1H), 6.19 (d, *J* = 16.0 Hz, 1H), 4.95 – 4.77 (m, 2H), 3.09 (dd, *J* = 9.3, 7.3 Hz, 1H), 2.25 (ddd, *J* = 9.3, 5.5, 0.8 Hz, 1H), 1.94 (dd, *J* = 7.3, 5.5 Hz, 1H).
^{13}C NMR (151 MHz, $CDCl_3$) δ 171.2, 150.4, 150.1, 138.9, 136.1, 134.9, 130.1, 128.6 (2 C), 128.0, 126.3 (2 C), 123.6, 121.5, 94.9, 74.5, 33.2, 31.7, 18.4.
HRMS: (+p APCI) calculated for $[C_{19}H_{16}O_2N^{35}Cl_4+]$ 429.9929, found 429.9934
IR(neat): 3026, 2970, 1736, 2586, 1560, 1493, 1463, 1448, 1365, 1230, 1217, 1131, 1109, 1059, 966, 910, 835, 806, 783, 746, 718, 694, 646, 633, 572, 528 cm^{-1}
Chiral HPLC: (AD-H, 30 min, 1 mL/min, 3% *i*-PrOH in *n*-hexane, UV 230 nm) RT: 16.04 min, 20.66 min.

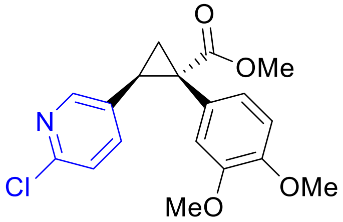

Methyl (1*S*,2*R*)-2-(6-chloropyridin-3-yl)-1-(3,4-dimethoxyphenyl)cyclopropane-1-carboxylate (30)
This compound was prepared according to **General procedure 3.2** from the reaction between **S6** (0.20 mmol, 47 mg) and **35** (2.32 equiv, 0.46 mmol, 65 mg) with CH_2Cl_2 as solvent in 60% yield and 71% ee (0.12 mmol, 41 mg). After isolation, enantio-enriched product was obtained as a clear colorless oil.
^{1}H NMR (400 MHz, $CDCl_3$) δ 8.08 (s, 1H), 6.97 (d, *J* = 8.3 Hz, 1H), 6.73 (dd, *J* = 8.1, 2.6 Hz, 1H), 6.70 (d, *J* = 8.2 Hz, 1H), 6.63 (dd, *J* = 8.3, 2.0 Hz, 1H), 6.43 (s, 1H), 3.83 (s, 3H), 3.69 (s, 3H), 3.66 (s, 3H), 3.05 (dd, *J* = 9.2, 7.1 Hz, 1H), 2.19 (dd, *J* = 9.4, 5.0 Hz, 1H), 1.79 (dd, *J* = 7.1, 5.1 Hz, 1H).
^{13}C NMR (151 MHz, $CDCl_3$) δ 173.8, 149.7, 149.4, 148.4, 148.4, 136.9, 131.8, 125.9, 124.1, 123.1, 115.0, 110.6, 55.8, 55.7, 52.8, 37.3, 29.4, 20.9.
HRMS: (+p APCI) calculated for $[C_{18}H_{19}O_4N^{35}Cl +]$ 348.0997, found 348.0996
IR(neat): 3003, 2969, 2950, 1737, 1721, 1587, 1551, 1517, 1462, 1435, 1414, 1365, 1352, 1252, 1227, 1217, 1157, 1140, 1108, 1026, 908, 742, 658, 528 cm^{-1}
Chiral HPLC: (AD-H, 60 min, 1 mL/min, 3% *i*-PrOH in *n*-hexane, UV 230 nm) RT: 32.96 min, 45.12 min.

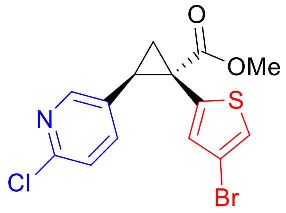

Methyl (1*R*,2*R*)-1-(4-bromothiophen-2-yl)-2-(6-chloropyridin-3-yl)cyclopropane-1-carboxylate (31)
This compound was prepared according **General procedure 3.2** from the reaction between **S16** (0.20 mmol, 52 mg) and **35** (2.32 equiv, 0.46 mmol, 65 mg) with CH_2Cl_2 as solvent in 98% yield and 95% ee (0.20 mmol, 73 mg). After isolation, enantio-enriched product was obtained as a light brown solid.
MP: 126-127 ℃
^{1}**H NMR** (400 MHz, $CDCl_3$) δ 8.11 (dt, *J* = 2.5, 0.7 Hz, 1H), 7.10 (dd, *J* = 8.3, 0.8 Hz, 1H), 7.06 (dd, *J* = 2.5, 0.5 Hz, 1H), 7.03 (d, *J* = 1.5 Hz, 1H), 6.69 (d, *J* = 1.5 Hz, 1H), 3.74 (s, 3H), 3.13 (dd, *J* = 9.3, 7.4 Hz, 1H), 2.28 (dd, *J* = 9.3, 5.3 Hz, 1H), 1.95 (dd, *J* = 7.4, 5.3 Hz, 1H).
^{13}**C NMR** (151 MHz, $CDCl_3$) δ 172.2, 150.4, 149.9, 138.8, 137.6, 132.3, 130.5, 124.2, 123.7, 108.9, 53.4, 32.1, 31.3, 22.0.
HRMS: (+p APCI) calculated for [$C_{14}H_{12}O_2N^{79}Br^{35}Cl^{32}S$+] 371.9455, found 371.9458
IR(neat): 3105, 2951, 1720, 1586, 1561, 1528, 1464, 1434, 1348, 1265, 1208, 1155, 1108, 1023, 963, 915, 881, 836, 799, 733, 678, 633, 604, 563 cm^{-1}
Chiral HPLC: (OD-H, 60 min, 1 mL/min, 1% *i*-PrOH in *n*-hexane, UV 230 nm) RT: 43.1 min, 50.5 min.

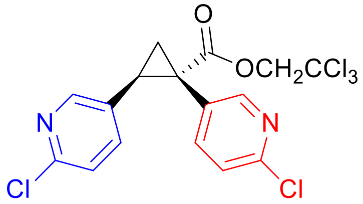

2,2,2-trichloroethyl (1*S*,2*R*)-1,2-bis(6-chloropyridin-3-yl)cyclopropane-1-carboxylate (32)
This compound was prepared according to **General procedure 3.2** from the reaction between **S3** (0.20 mmol, 66 mg) and **35** (2.32 equiv, 0.46 mmol, 65 mg) with CH_2Cl_2 as solvent in 73% yield and 97% ee (0.15 mmol, 64 mg). After isolation, enantio-enriched product was obtained as a crystalline yellow solid.
MP: 88-89 ℃
^{1}**H NMR** (400 MHz, $CDCl_3$) δ 8.19 (d, *J* = 2.5 Hz, 1H), 8.09 (d, *J* = 2.6 Hz, 1H), 7.34 (dd, *J* = 8.3, 2.5 Hz, 1H), 7.23 – 7.15 (m, 1H), 7.11 (d, *J* = 8.3 Hz, 1H), 6.94 (dd, *J* = 8.3, 2.6 Hz, 1H), 4.87 (d, *J* = 11.9 Hz, 1H), 4.67 (d, *J* = 11.9 Hz, 1H), 3.26 (dd, *J* = 9.5, 7.4 Hz, 1H), 2.44 (dd, *J* = 9.4, 5.7 Hz, 1H), 2.03 (dd, *J* = 7.4, 5.7 Hz, 1H).
^{13}**C NMR** (151 MHz, $CDCl_3$) δ 170.1, 152.2, 151.0, 150.4, 149.6, 142.3, 137.5, 129.6, 128.0, 123.9, 94.5, 74.6, 34.1, 30.0, 19.4.
HRMS: (+p APCI) calculated for [$C_{16}H_{12}O_2N_2{}^{35}Cl_5$+] 438.9335, found 438.9337
IR(neat): 3016, 2970, 1738, 1587, 1560, 1463, 1366, 1230, 1217, 1162, 1113, 1060, 1022, 912, 839, 812, 779, 749, 528 cm^{-1}
Chiral HPLC: (AD-H, 60 min, 1 mL/min, 7% *i*-PrOH in *n*-hexane, UV 230 nm) RT: 36.61 min, 44.71 min.

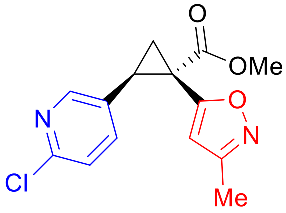

Methyl (1*S*,2*R*)-2-(6-chloropyridin-3-yl)-1-(3-methylisoxazol-5-yl)cyclopropane-1-carboxylate (33)
This compound was prepared according **General procedure 3.2** from the reaction between **S13** (0.20 mmol, 36 mg) and **35** (2.32 equiv, 0.46 mmol, 65 mg) with CH_2Cl_2 as solvent in 60% yield and 83% ee (0.12 mmol, 35 mg). After isolation, enantio-enriched product was obtained as a clear colorless oil.
^{1}**H NMR** (600 MHz, $CDCl_3$) δ 8.13 (dt, *J* = 2.6, 0.7 Hz, 1H), 7.24 (ddd, *J* = 8.3, 2.5, 0.6 Hz, 1H), 7.12 (dd, *J* = 8.4, 0.8 Hz, 1H), 5.91 (s, 1H), 3.76 (s, 3H), 3.18 (dd, *J* = 9.2, 7.8 Hz, 1H), 2.24 – 2.20 (m, 1H), 2.20 – 2.16 (m, 1H), 2.15 (s, 3H).
^{13}**C NMR** (151 MHz, $CDCl_3$) δ 170.6, 165.3, 160.1, 150.6, 150.0, 138.2, 129.9, 123.9, 106.8, 53.3, 31.2, 29.3, 19.9, 11.6
HRMS: (+p APCI) calculated for [$C_{14}H_{14}O_3N_2{}^{35}Cl$ +] 293.0687, found 293.0687
IR(neat): 2954, 1725, 1612, 1588, 1561, 1463, 1435, 1414, 1351, 1319, 1265, 1219, 1200, 1157, 1108, 1077, 1024, 1008, 986, 964, 914, 835, 799, 761, 736, 696, 645, 633, 561, 458 cm^{-1}
Chiral HPLC: (OD-H, 60 min, 1 mL/min, 3% *i*-PrOH in *n*-hexane, UV 230 nm) RT: 39.76 min, 51.41 min.

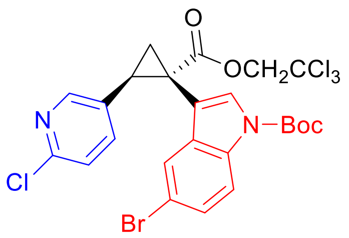

Tert-butyl 5-bromo-3-((1S,2R)-)-2-(6-chloropyridin-3-yl)-1-((2,2,2-trichloroethoxy)carbonyl)cyclopropyl)-1H-indole-1-carboxylate (34)

This compound was prepared according to the procedure outlined in **3.2.1.** After isolation, enantio-enriched product was obtained as a brown oil in 82% yield and 89% ee (0.16 mmol, 102 mg).

^{1}H NMR (400 MHz, $CDCl_3$) δ 8.04 (s, 1H), 7.87 (d, *J* = 8.8 Hz, 1H), 7.44 (s, 1H), 7.30 (dd, *J* = 8.8, 2.0 Hz, 1H), 7.25 – 7.14 (m, 1H), 7.02 (d, *J* = 8.3 Hz, 1H), 4.87 (d, *J* = 11.9 Hz, 1H), 4.65 (d, *J* = 11.9 Hz, 1H), 3.28 (dd, *J* = 9.4, 7.3 Hz, 1H), 2.36 (dd, *J* = 9.4, 5.2 Hz, 1H), 2.00 (dd, *J* = 7.4, 5.2 Hz, 1H), 1.63 (s, 9H).

^{13}C NMR (151 MHz, $CDCl_3$) δ 170.8, 150.0, 148.9, 138.2, 133.8, 131.5, 130.3, 128.1, 127.5, 123.2, 122.5, 116.6, 116.0, 113.0, 94.6, 84.6, 74.5, 29.7, 28.1 (3 C), 20.0.

HRMS: (+p APCI) calculated for [$C_{24}H_{22}O_4N_2{}^{79}Br^{35}Cl_4$+] 620.9511, found 620.9509

IR(neat): 2980, 1732, 1587, 1561, 1451, 1371, 1304, 1274, 1240, 1215, 1197, 1152, 1121, 1055, 1026, 961, 908, 839, 801, 789, 765, 731, 648, 636, 572 cm^{-1}

Chiral HPLC: (OD-H, 30 min, 1 mL/min, 3% *i*-PrOH in *n*-hexane, UV 230 nm) RT: 15.17 min, 21.87 min.

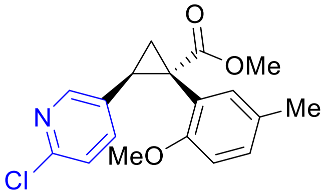

Methyl (1S,2R)-2-(6-chloropyridin-3-yl)-1-(2-methoxy-5-methylphenyl)cyclopropane-1-carboxylate (37)

This compound was prepared according to **General procedure 3.2, General procedure 3.3, General procedure 3.4, General procedure 3.5,** and **General procedure 3.6** from the reaction between **35** and **36** with varying levels of enantioselectivity depending on the method and conditions used. **General procedure 3.2: 36** (0.20 mmol, 44 mg) and **35** (2.32 equiv, 0.46 mmol, 65 mg) with CH_2Cl_2 as solvent in 22% yield and 22% ee (0.04 mmol, 15 mg). **General procedure 3.3: 36** (0.20 mmol, 44 mg) and **35** (5.0 equiv, 1.00 mmol, 160 mg) in 95% yield and 98% ee (0.19 mmol, 63 mg)**. General procedure 3.4: 36** (0.20 mmol, 44 mg) and **35** (1.5 equiv, 0.30 mmol, 42 mg) with CH_2Cl_2 as solvent in 87% yield and 98% ee (0.17 mmol, 58 mg). **General procedure 3.5: 36** (0.20 mmol, 44 mg) and **35** (1.5 equiv, 0.30 mmol, 42 mg) with CH_2Cl_2 as solvent in 89% yield and 98% ee (0.18 mmol, 59 mg). Only optimized results for each procedure are reported here, enantioselectivity data corresponding to other variants in Table 1 and Table 2 may be found in section **5.3**. **General procedure 3.6:** Reaction was performed according to the described procedure and **37** was obtained in 83% yield over two steps and 98% ee. After isolation, enantio-enriched product was obtained as a clear colorless oil, racemate is obtained as colorless orthorhombic crystals.

MP: 126-127 ℃

^{1}H NMR (600 MHz, $CDCl_3$) δ 8.00 (d, *J*= 2.53 Hz, 1H), 6.98 (s, 1H), 6.94 (d, *J*= 8.19 Hz, 1H), 6.89 (d, *J* = 8.23 Hz, 1H), 6.81 (dd, *J*= 2.51, 8.33 Hz, 1H), 6.44 (d, *J*= 8.24 Hz, 1H), 3.63 (s, 3H), 3.37 (s, 3H), 3.17 (m, 1H), 2.23 (s, 3H), 1.98 (m, 1H), 1.78 (m, 1H).

^{13}C NMR (151 MHz, $CDCl_3$) δ 173.8, 156.2, 149.6, 148.8, 136.6, 131.9, 129.7, 129.3, 122.3, 110.2, 55.0, 52.6, 34.1, 28.7, 25.3, 20.4, 20.0

HRMS: (+p APCI) calculated for [$C_{18}H_{19}O_3N^{35}Cl$ +] 332.1048, found 332.1046

IR(neat): 1716, 1562, 1501, 1461, 1434, 1348, 1262, 1241, 1158, 1140, 1107, 1029, 972, 905, 808, 732, 671, 548 cm^{-1}

Chiral HPLC: (OD-H, 30 min, 1 mL/min, 1% *i*-PrOH in *n*-hexane, UV 230 nm) RT: 19.46 min, 22.97 min.

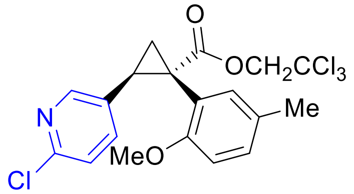

2,2,2-Trichloroethyl (1S,2R)-2-(6-chloropyridin-3-yl)-1-(2-methoxy-5-methylphenyl)cyclopropane-1-carboxylate (Table 2, **entry 7)**

This compound was prepared according to **General procedure 3.3** from the reaction between **2,2,2-Trichloroethy-1-(2-methoxy-5-methylphenyl)-1-carboxylate** (0.20 mmol, 70 mg) and **35** (5.0 equiv, 1.00 mmol, 140 mg) in in 47% yield and 39% ee (0.09 mmol, 44 mg). After isolation, enantio-enriched product was obtained as a clear colorless oil.

^{1}H NMR (400 MHz, CDCl3) δ 8.04 (d, *J* = 2.5 Hz, 1H), 7.08 – 6.82 (m, 4H), 6.44 (d, *J* = 8.3 Hz, 1H), 4.88 (d, *J* = 11.9 Hz, 1H), 4.57 (d, *J* = 11.9 Hz, 1H), 3.40 (s, 3H), 3.26 (dd, *J* = 9.3, 7.3 Hz, 1H), 2.25 (s, 3H), 2.13 (dd, *J* = 9.3, 5.4 Hz, 1H), 1.92 (dd, *J* = 7.3, 5.4 Hz, 1H).
^{13}C NMR (151 MHz, CDCl3) δ 171.5, 156.1, 149.6, 149.0, 136.8, 131.9, 131.4, 130.0, 129.2, 122.4, 121.2, 109.9, 94.9, 74.4, 54.8, 34.0, 29.2, 20.5, 20.1.
HRMS: (+p APCI) calculated for [$C_{19}H_{18}O_3N^{35}Cl_4$+] 448.0035, found 448.0033
IR(neat): 2925, 1736, 1613, 1587, 1562, 1503, 1462, 1367, 1239, 1138, 1108, 1058, 1034, 978, 911, 805, 767, 726, 572 cm^{-1}
Chiral HPLC: (AD-H, 60 min, 1 mL/min, 0.5 % *i*-PrOH in *n*-hexane, UV 230 nm) RT: 37.07 min, 40.77 min.

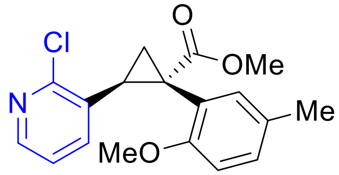

Methyl (1*S*,2*S*)-2-(2-chloropyridin-3-yl)-1-(2-methoxy-5-methylphenyl)cyclopropane-1-carboxylate (38)
This compound was prepared according to **General procedure 3.3, General procedure 3.4,** and **General procedure 3.5** from the reaction between **S25** and **36. General procedure 3.3: 36** (0.20 mmol, 44 mg) and **S25** (5.0 equiv, 1.00 mmol, 160 mg) in 85% yield and 95% ee (0.17 mmol, 56 mg) **General procedure 3.5: 36** (0.20 mmol, 44 mg) and **S25** (1.5 equiv, 0.30 mmol, 42 mg) in 62% yield and 90% ee (0.12 mmol, 41 mg). After isolation, enantio-enriched product was obtained as a white solid.
MP: 143-152℃
^{1}H NMR (600 MHz, CDCl3) δ 8.03 (dd, *J*=1.94, 4.73, 1H), 7.01 (t, *J*=3.70, 1H), 6.93 (d, *J*=8.460, 1H), 6.72 (dd, *J*=4.67, 7.66), 6.47 (d, *J*=1.94, 1H), 6.40 (d, *J*=8.15, 1H), 3.67 (s, 3H), 3.63 (m, 1H), 3.38 (s, 3H), 2.25 (s, 3H), 2.05 (m, 1H), 1.84 (m, 1H)
^{13}C NMR (151 MHz, CDCl3) δ 173.5, 156.4, 153.2, 146.3, 133.8, 132.5, 131.7, 129.5, 128.9, 122.4, 120.8, 109.8, 54.8, 52.6, 34.4, 28.5, 20.4, 19.9.
HRMS: (+p APCI) calculated for [$C_{18}H_{19}O_3N^{35}Cl$ +] 332.1048, found 332.1048
IR(neat): 2919, 1720, 1585, 1557, 1433, 1264, 1244, 1158, 1140, 1034, 910, 800, 733 cm^{-1}
Chiral HPLC: (OD-H, 30 min, 1 mL/min, 1% *i*-PrOH in *n*-hexane, UV 230 nm) RT: 12.44 min, 22.31 min.

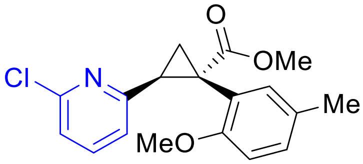

Methyl (1*S*,2*S*)-2-(6-chloropyridin-2-yl)-1-(2-methoxy-5-methylphenyl)cyclopropane-1-carboxylate (39)
This compound was prepared according to **General procedure 3.3, General procedure 3.4,** and **General procedure 3.5** from the reaction between **66** and **36. General procedure 3.3: 36** (0.20 mmol, 44 mg) and **66** (5.0 equiv, 1.00 mmol, 160 mg) in 86% yield and 90% ee (0.17 mmol, 57 mg), **General procedure 3.5: 36** (0.20 mmol, 44 mg) and **66** (1.5 equiv, 0.30 mmol, 42 mg) in 81% yield and >99% ee (0.16 mmol, 54 mg). After isolation, enantio-enriched product was obtained as a white solid.
MP: 114-116℃
^{1}H NMR (400 MHz, CDCl3) δ 7.31 – 7.16 (m, 2H), 7.02 (d, *J* = 2.3 Hz, 1H), 6.91 (ddd, *J* = 7.7, 5.6, 1.3 Hz, 2H), 6.74 (dd, *J* = 7.6, 0.8 Hz, 1H), 6.41 (d, *J* = 8.3 Hz, 1H), 3.64 (s, 3H), 3.44 – 3.31 (buried m under main s, 4H), 2.28 – 2.17 (buried m under main s, 4H), 1.96 (dd, *J* = 8.9, 4.7 Hz, 1H).
^{13}C NMR (151 MHz, CDCl3) δ 173.8, 157.9, 156.3, 149.5, 137.2, 132.6, 129.0, 128.9, 122.7, 121.0, 121.0, 109.6, 55.0, 52.5, 34.8, 32.9 29.7, 20.4
HRMS: (+p APCI) calculated for [$C_{18}H_{19}O_3N^{35}Cl$ +] 332.1048, found 332.1044
IR(neat): 2916, 2849, 1721, 1585, 1557, 1502, 1433, 1376, 1263, 1242, 1158, 1141, 1033, 910, 800, 720 cm^{-1}
Chiral HPLC: (OD-H, 60 min, 1 mL/min, 1% *i*-PrOH in *n*-hexane, UV 230 nm) RT: 22.93 min, 36.26 min.

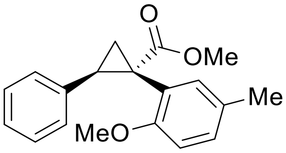

Methyl (1*S*,2*R*)-1-(2-methoxy-5-methylphenyl)-2-phenylcyclopropane-1-carboxylate (40)
This compound was prepared according to **General procedure 3.3** and **General procedure 3.4** from the reaction between **36** and freshly columned styrene. **General procedure 3.3: 36** (0.20 mmol, 44 mg) and styrene (5.0 equiv, 1.00 mmol, 100 mg) in 81% yield and 4% ee (0.16 mmol, 48 mg) **General procedure 3.4: 36** (0.20 mmol, 44 mg) and styrene (5.0 equiv, 0.30 mmol, 100 mg) in the presence of 2-chloropyridine (1.0 equiv, 0.20 mmol, 23 mg) in 95% yield and 95% ee (0.19 mmol, 56 mg). After isolation, enantio-enriched product was obtained as a clear colorless oil. Other coordinating additives gave different levels of enantioselectivity as reported in **section 5.3**.

^{1}H NMR (400 MHz, CDCl₃) δ 7.02 (dd, *J* = 5.1, 1.9 Hz, 3H), 6.96 (d, *J* = 2.3 Hz, 1H), 6.94 – 6.86 (m, 1H), 6.83 – 6.71 (m, 2H), 6.43 (d, *J* = 8.2 Hz, 1H), 3.65 (s, 3H), 3.31 (s, 3H), 3.21 (dd, *J* = 9.3, 7.4 Hz, 1H), 2.23 (s, 3H), 1.97 (dd, *J* = 9.3, 4.9 Hz, 1H), 1.82 (dd, *J* = 7.3, 5.0 Hz, 1H).
^{13}C NMR (151 MHz, CDCl₃) δ 174.5, 156.9, 136.9, 132.3, 129.0, 128.8, 127.7, 127.7, 127.0, 125.8, 123.5, 110.1, 55.0, 55.0, 52.4, 34.0, 32.4, 20.6, 20.5.
HRMS: (+p APCI) calculated for [$C_{19}H_{20}O_3$+] 296.1407, found 296.1409
IR(neat): 1716, 1502, 1460, 1436, 1410, 1354, 1263, 1244, 1186, 1159, 1144, 1067, 1031, 907, 806, 729, 683, 646, 503 cm^{-1}
Chiral HPLC: (OD-H, 30 min, 1 mL/min, 1% *i*-PrOH in *n*-hexane, UV 230 nm) RT: 10.89 min, 12.80 min. or (AD-H, 30 min, 1 mL/min, 1% *i*-PrOH in *n*-hexane, UV 230 nm) RT: 9.61 min, 11.17 min.

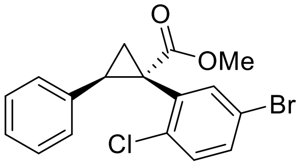

Methyl (1*S*,2*R*)-1-(5-bromo-2-chlorophenyl)-2-phenylcyclopropane-1-carboxylate (42)
This compound was prepared according to **General procedure 3.3** and **General procedure 3.4** from the reaction between **S12** and freshly columned styrene. **General procedure 3.3: S12** (0.20 mmol, 58 mg) and styrene (5.0 equiv, 1.00 mmol, 100 mg) in 76% yield and 64% ee (0.15 mmol, 56 mg) **General procedure 3.4: S12** (0.20 mmol, 58 mg) and styrene (5.0 equiv, 0.30 mmol, 100 mg) in the presence of 2-chloropyridine (1.0 equiv, 0.20 mmol, 23 mg) in 71% yield and 92% ee (0.14 mmol, 52 mg). After isolation, enantio-enriched product was obtained as a clear colorless oil. Other coordinating additives gave different levels of enantioselectivity as reported in the following section.
^{1}H NMR (600 MHz, CDCl₃) δ 7.23 (dd, *J*=2.23, 8.46 Hz, 1H), 7.10 (s, 4H), 7.02 (broad s, 1H), 6.86 (m, 2H), 3.68 (s, 3H) 3.32 (m, 1H), 2.10 (broad s, 1H), 1.90 (m, 1H)
^{13}C NMR (151 MHz, CDCl₃) δ 172.6, 136.8, 135.5, 134.0, 131.5, 130.6, 127.9, 127.6, 127.6, 119.5, 52.7, 33.3, 33.3, 21.4
HRMS: (+p APCI) calculated for [$C_{17}H_{15}O_2{}^{79}Br^{35}Cl$+] 364.9938, found 364.9932
IR(neat): 3027, 2970, 2949, 1720, 1458, 1433, 1266, 1246, 1208, 1192, 116, 1115, 1085, 1045, 969, 814, 770, 731, 695, 526 cm^{-1}
Chiral HPLC: (OJ-H, 30 min, 1 mL/min, 1% *i*-PrOH in *n*-hexane, UV 230 nm) RT: 9.07 min, 11.82 min

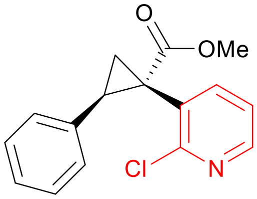

Methyl (1*S*,2*R*)-1-(2-chloropyridin-3-yl)-2-phenylcyclopropane-1-carboxylate (43)
This compound was prepared according to **General procedure 3.3** and **General procedure 3.4** from the reaction between **S20** and freshly columned styrene. **General procedure 3.3: S20** (0.20 mmol, 42 mg) and styrene (5.0 equiv, 1.00 mmol, 100 mg) in 82% yield and 77% ee (0.16 mmol, 47 mg) **General procedure 3.4: S20** (0.20 mmol, 42 mg) and styrene (5.0 equiv, 0.30 mmol, 100 mg) in the presence of 2-chloropyridine (1.0 equiv, 0.20 mmol, 23 mg) in 94% yield and 90% ee (0.19 mmol, 54 mg). After isolation, enantio-enriched product was obtained as a clear crystalline solid.
MP: 115-119℃
^{1}H NMR (600 MHz, CDCl₃) δ 8.19 (m, 1H), 7.09 (broad s, 5H), 6.85 (m, 2H), 3.68 (s, 3H), 3.34 (m, 1H), 2.18 (broad s, 1H), 1.92 (dd, *J*= 5.46, 7.59 Hz, 1H)
^{13}C NMR (151 MHz, CDCl₃) δ 172,5, 154.3, 148.1, 141.3, 134.9, 130.4, 129.3 128.0, 127.8, 126.9, 121.6, 52.7, 34.1, 33.5, 25.3, 20.0
HRMS: (+p APCI) calculated for [$C_{16}H_{15}O_2N^{35}Cl$ +] 288.0785, found 288.0786
IR(neat): 1720, 1452, 1433, 1397, 1262, 1221, 1163, 1132, 1059, 966, 908, 776, 750, 728, 697, 646, 562 cm^{-1}
Chiral HPLC: (AD-H, 30 min, 1 mL/min, 1% *i*-PrOH in *n*-hexane, UV 230 nm) RT: 20.19 min, 23.41 min.

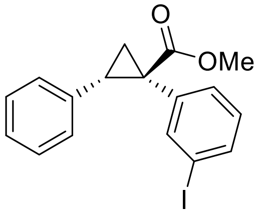

Methyl (1*R*,2*S*)-1-(3-iodophenyl)-2-phenylcyclopropane-1-carboxylate (45)
This compound was prepared according to **General procedure 3.3** and **General procedure 3.4** from the reaction between **S10** and freshly columned styrene. **General procedure 3.3: S10** (0.20 mmol, 60 mg) and styrene (5.0 equiv, 1.00 mmol, 100 mg) in 66% yield and 29% ee (0.13 mmol, 50 mg) **General procedure 3.4: S10** (0.20 mmol, 60 mg) and styrene (5.0

equiv, 0.30 mmol, 100 mg) in the presence of 2-chloropyridine (1.0 equiv, 0.20 mmol, 23 mg) in 80% yield and 41% ee (0.16 mmol, 61 mg). After isolation, product was obtained as a colorless oil.
^{1}H NMR (400 MHz, $CDCl_3$) δ 7.52 – 7.38 (m, 2H), 7.10 (dd, *J* = 5.2, 2.0 Hz, 3H), 6.98 – 6.85 (m, 1H), 6.84 (d, *J* = 7.7 Hz, 1H), 6.82 – 6.72 (m, 2H), 3.67 (s, 3H), 3.11 (dd, *J* = 9.3, 7.3 Hz, 1H), 2.13 (dd, *J* = 9.3, 5.0 Hz, 1H), 1.85 (dd, *J* = 7.4, 5.0 Hz, 1H).
^{13}C NMR (151 MHz, $CDCl_3$) δ 173.6, 140.7, 137.2, 136.1, 135.7, 131.4, 129.2, 128.0, 127.8, 126.6, 93.3, 52.7, 36.7, 33.1, 20.2.
HRMS: (+p APCI) calculated for $[C_{17}H_{16}O_2{}^{127}I +]$ 379.0189, found 379.0184
IR(neat): 1713, 1590, 1559, 1474, 1455, 1431, 1250, 1209, 1190, 1095, 1054, 995, 965, 936, 908, 884, 789, 763, 729, 696, 678, 645, 592, 565 cm^{-1}
Chiral HPLC: (OD-H, 30 min, 1 mL/min, 1% *i*-PrOH in *n*-hexane, UV 230 nm) RT: 8.23 min, 9.27 min.

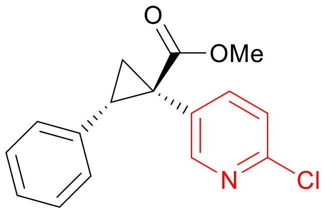

Methyl (1*R*,2*S*)-1-(6-chloropyridin-3-yl)-2-phenylcyclopropane-1-carboxylate (46)
This compound was prepared according to **General procedure 3.3** and **General procedure 3.4** from the reaction between **S4** and freshly columned styrene. **General procedure 3.3: S4** (0.20 mmol, 42 mg) and styrene (5.0 equiv, 1.00 mmol, 100 mg) in 90% yield and 35% ee (0.18 mmol, 52 mg) **General procedure 3.4: S4** (0.20 mmol, 42 mg) and styrene (5.0 equiv, 0.30 mmol, 100 mg) in the presence of 2-chloropyridine (1.0 equiv, 0.20 mmol, 23 mg) in 90% yield and 7% ee (0.18 mmol, 52 mg). After isolation, enantio-enriched product was obtained as a clear colorless oil.
^{1}H NMR (600 MHz, $CDCl_3$) δ 8.08 (dd, J = 2.6, 0.8 Hz, 1H), 7.21 (dd, J = 8.2, 2.5 Hz, 1H), 7.11 (dq, J = 4.5, 2.3 Hz, 3H), 7.05 (dd, J = 8.2, 0.7 Hz, 1H), 6.87 – 6.71 (m, 2H), 3.67 (d, J = 3.5 Hz, 3H), 3.16 (dd, J = 9.3, 7.3 Hz, 1H), 2.21 (dd, J = 9.4, 5.2 Hz, 1H), 1.96 – 1.86 (m, 1H).
^{13}C NMR (151 MHz, $CDCl_3$) δ 173.2, 152.7, 150.2, 142.4, 135.1, 130.3, 128.5, 128.2, 127.3, 123.5, 53.0, 34.3, 33.1, 19.7.
HRMS: (+p APCI) calculated for $[C_{16}H_{15}O_2N^{35}Cl+]$ 288.0785, found 288.0786
IR(neat): 2952, 1720, 1588, 1560, 1499, 1463, 1433, 1366, 1261, 1164, 1112, 1022, 965, 779, 742, 719, 696, 559, 485 cm^{-1}
Chiral HPLC: (OD-H, 30 min, 1 mL/min, 1% *i*-PrOH in *n*-hexane, UV 230 nm) RT: 22.14 min, 25.67 min.

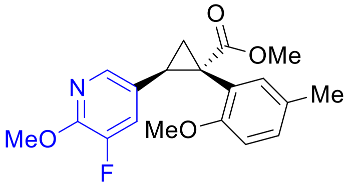

Methyl-(1*S*,2*R*)-2-(5-fluoro-6-methoxypyridin-3-yl)-1-(2-methoxy-5-methylphenyl)cyclopropane-1-carboxylate (47)
This compound was prepared according to **General procedure 3.4,** and **General procedure 3.5** from the reaction between **36** and **S26. General procedure 3.4: 36** (0.20 mmol, 44 mg) and **S26** (1.5 equiv, 0.30 mmol, 46 mg) in 53% yield and 88% ee (0.16 mmol, 37 mg).**, General procedure 3.5: 36** (0.20 mmol, 44 mg) and **S26** (1.5 equiv, 0.30 mmol, 46 mg) in 69% yield and 86% ee (0.16 mmol, 48 mg). After isolation, enantio-enriched product was obtained as a white solid.
MP: 114-116 ℃
^{1}H NMR (400 MHz, $CDCl_3$) δ 7.57 (s, 1H), 6.98 – 6.91 (m, 2H), 6.65 (dd, *J* = 11.6, 1.9 Hz, 1H), 6.53 – 6.46 (m, 1H), 3.91 (s, 3H), 3.65 (s, 3H), 3.48 (s, 3H), 3.15 (dd, *J* = 9.3, 7.2 Hz, 1H), 2.24 (s, 3H), 1.93 (dd, *J* = 9.3, 5.1 Hz, 1H), 1.74 (dd, *J* = 7.2, 5.2 Hz, 2H).
^{13}C NMR (151 MHz, $CDCl_3$) δ 174.1, 151.5 (d, *J* = 11.3 Hz), 140.6, 140.6, 131.7, 129.5, 129.3, 126.9, 122.2, 122.1, 110.2, 55.0, 53.6, 52.5, 33.5, 29.7, 28.5, 20.5, 19.8.
^{19}F NMR (151 MHz, $CDCl_3$) δ -142.02 (d, *J* = 11.6 Hz)
HRMS: (+p APCI) calculated for $[C_{19}H_{21}O_4NF +]$ 346.1449, found 346.1448
IR(neat): 2948, 2850, 1720, 1616, 1577, 1495, 1440, 1411, 1378, 1296, 1240, 1194, 1161, 1136, 1085, 1016, 965, 906, 808, 778, 726, 647, 631, 560, 513 cm^{-1}
Chiral HPLC: (OD-H, 30 min, 1 mL/min, 1% *i*-PrOH in *n*-hexane, UV 230 nm) RT: 10.90 min, 16.19 min.
.

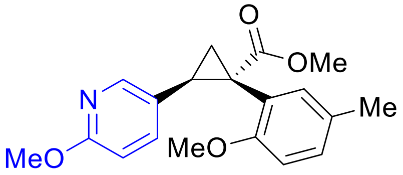

Methyl-(1*S*,2*R*)-1-(2-methoxy-5-methylphenyl)-2-(6-methoxypyridin-3-yl)cyclopropane-1-carboxylate (48)

This compound was prepared according to **General procedure 3.4** and **General procedure 3.5** from the reaction between **S24** and **36. General procedure 3.4: 36** (0.20 mmol, 44 mg) and **S24** (1.5 equiv, 30 mmol, 46 mg) in 98% yield and 92% ee (0.20 mmol, 64 mg) **General procedure 3.5: 36** (0.20 mmol, 44 mg) and **S24** (1.5 equiv, 0.30 mmol, 46 mg) in 66% yield and 90% ee (0.13 mmol, 43 mg). After isolation, enantio-enriched product was obtained as a clear colorless oil.
^{1}H NMR (400 MHz, $CDCl_3$) δ 7.78 (d, *J* = 2.5 Hz, 1H), 6.97 – 6.89 (m, 2H), 6.83 (dd, *J* = 8.6, 2.5 Hz, 1H), 6.46 (d, *J* = 8.1 Hz, 1H), 6.35 (d, *J* = 8.6 Hz, 1H), 3.81 (s, 3H), 3.64 (s, 3H), 3.42 (s, 3H), 3.14 (dd, *J* = 9.3, 7.3 Hz, 1H), 2.23 (s, 3H), 1.92 (dd, *J* = 9.3, 5.1 Hz, 1H), 1.76 (dd, *J* = 7.3, 5.1 Hz, 1H).
^{13}C NMR (151 MHz, $CDCl_3$) δ 174.3, 162.6, 156.5, 146.4, 137.3, 131.9, 129.3, 129.1, 125.3, 123.1, 110.1, 108.9, 55.0, 53.2, 52.5, 33.5, 29.1, 20.5, 19.8.
HRMS: (+p APCI) calculated for [$C_{19}H_{22}O_4N$ +] 328.1543, found 328.1537
IR(neat): 2948, 1716, 1607, 1571, 1496, 1463, 1435, 1399, 1352, 1259, 1242, 1191, 1157, 1143, 1130, 1084, 1029, 971, 907, 827, 808, 769, 726, 608, 588, 550 cm^{-1}
Chiral HPLC: (OD-H, 30 min, 1 mL/min, 1% *i*-PrOH in *n*-hexane, UV 230 nm) RT: 10.74 min, 18.24 min.

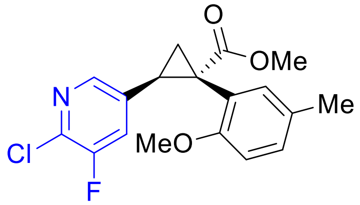

Methyl-(1*S*,2*R*)-2-(6-chloro-5-fluoropyridin-3-yl)-1-(2-methoxy-5-methylphenyl)cyclopropane-1-carboxylate (49)
This compound was prepared according to **General procedure 3.4** and **General procedure 3.5** from the reaction between **S27** and **36. General procedure 3.4: 36** (0.20 mmol, 44 mg) and **S27** (1.5 equiv, 30 mmol, 47 mg) in 55% yield and 90% ee (0.11 mmol, 38mg) **General procedure 3.5: 36** (0.20 mmol, 44 mg) and **S27** (1.5 equiv, 0.30 mmol, 47 mg) in 45% yield and 97% ee (0.09 mmol, 31 mg). After isolation, enantio-enriched product was obtained as a white solid.
MP: 124-127℃
^{1}H NMR (400 MHz, $CDCl_3$) δ 7.87 (d, *J* = 2.0 Hz, 1H), 7.06 – 6.94 (m, 2H), 6.68 (dd, *J* = 9.4, 2.1 Hz, 1H), 6.48 (d, *J* = 8.9 Hz, 1H), 3.65 (s, 3H), 3.43 (s, 3H), 3.21 (dd, *J* = 9.2, 7.1 Hz, 1H), 2.25 (s, 3H), 2.00 (dd, *J* = 9.2, 5.3 Hz, 1H), 1.78 (dd, *J* = 7.1, 5.3 Hz, 1H).
^{13}C NMR (151 MHz, $CDCl_3$) δ 173.6, 156.1, 154.4, 152.7, 144.6 (d, *J* = 5.0 Hz), 135.0 (d, *J* = 3.1 Hz), 131.7, 130.0, 129.5, 122.6 (d, *J* = 19.4 Hz), 122.0, 110.3, 55.0, 52.7, 34.3, 28.2, 20.5, 20.3.
^{19}F NMR (376 MHz, $CDCl_3$) δ -121.30 (d, *J* = 9.4 Hz).
HRMS: (+p APCI) calculated for [$C_{18}H_{18}O_3N^{35}ClF$ +] 350.0953, found 350.0951
IR(neat): 2950 1716, 1501, 1433, 1411, 1379, 1263, 1241, 1210, 1181, 1155, 1142, 1074, 1031, 964, 907, 808, 728, 703, 678, 647, 619, 559, 488 cm^{-1}
Chiral HPLC: (*R,R*-Whelk, 60 min, 1 mL/min, 1% *i*-PrOH in *n*-hexane, UV 230 nm) RT: 43.60 min, 52.05 min.

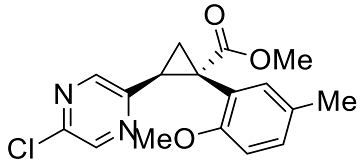

Methyl (1S,2S)-2-(5-chloropyrazin-2-yl)-1-(2-methoxy-5-methylphenyl)cyclopropane-1-carboxylate (50)
This compound was prepared according to **General procedure 3.5** from the reaction between **36** (0.20 mmol, 44 mg) and **2-chloro-5-ethenyl-pyrimidine** (1.5 equiv, 0.30 mmol, 84 mg, Enamine) in 89% yield and 96% ee (0.16 mmol, 59 mg). After isolation, enantio-enriched product was obtained as a viscous colorless oil.
^{1}H NMR (400 MHz, $CDCl_3$) δ 8.20 (d, J = 1.4 Hz, 1H), 8.03 (d, J = 1.4 Hz, 1H), 7.03 (d, J = 2.3 Hz, 1H), 6.96 – 6.91 (m, 1H), 6.39 (d, J = 8.3 Hz, 1H), 3.65 (s, 3H), 3.41 (s, 3H), 3.40 – 3.36 (m, 1H), 2.30 (dd, J = 6.9, 4.5 Hz, 1H), 2.25 (s, 3H), 1.98 (dd, J = 8.8, 4.5 Hz, 1H);
^{13}C NMR (101 MHz, $CDCl_3$) δ 173.6, 155.9, 151.1, 146.2, 144.6, 142.0, 132.7, 129.5, 129.2, 122.3, 109.5, 54.9, 52.7, 35.1, 30.1, 20.5, 19.5;
HRMS (ESI) m/z calculated for [$C_{17}H_{18}ClN_2O_3$+H]+, 333.1000; found 333.1006.
Chiral SFC: (ChiralCel-OD 3 µm column, 3 mL / minute, 5-50% MeOH / CO_2 over 5 min, RT: 0.99 min, 1.13 min.

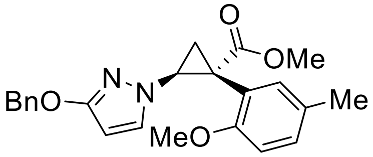

Methyl (1*R*,2*S*)-2-(3-(benzyloxy)-*1H*-pyrazol-1-yl)-1-(2-methoxy-5-methylphenyl)cyclopropane-1-carboxylate (51)
This compound was prepared according to **General procedure 3.5** from the reaction between **36** (0.20 mmol, 44 mg) and **S32** (1.5 equiv, 0.30 mmol, 60 mg) in 79% yield and 96% ee (0.16 mmol, 62mg). After isolation, enantio-enriched product was obtained as a viscous colorless oil.

^{1}H NMR (400 MHz, $CDCl_3$) δ 7.39 – 7.25 (m, 5H), 7.03 – 6.93 (m, 2H), 6.88 (d, J = 2.3 Hz, 1H), 6.60 (d, J = 8.3 Hz, 1H), 5.42 (d, J = 2.4 Hz, 1H), 5.06 – 4.88 (m, 2H), 4.49 (dd, J = 8.7, 5.6 Hz, 1H), 3.69 (s, 3H), 3.64 (s, 3H), 2.37 (t, J = 5.8 Hz, 1H), 2.17 (s, 3H), 2.04 (dd, J = 8.7, 6.0 Hz, 1H);
^{13}C NMR (101 MHz, $CDCl_3$) δ 173.0, 162.2, 156.9, 137.3, 131.9, 131.1, 129.4, 129.1, 128.4, 127.8, 127.7, 121.8, 110.0, 91.3, 70.7, 55.3, 52.5, 45.4, 32.5, 20.5, 18.7;
HRMS (ESI) m/z calculated for $[C_{23}H_{25}N_2O_4+H]+$, 393.1809; found 393.1818.
Chiral SFC: (ChiralPak-AD 3 μm column, 3 mL / minute, 5-50% MeOH / CO_2 over 5 min, RT: 1.18 min,1.39 min.

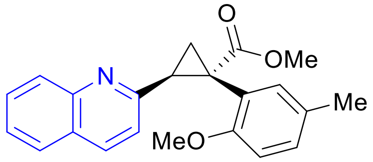

Methyl (1*S*,2*S*)-1-(2-methoxy-5-methylphenyl)-2-(quinolin-2-yl)cyclopropane-1-carboxylate (52)
This compound was prepared according to **General procedure 3.4** from the reaction between **36** (0.20 mmol, 44 mg) and **S28** (1.5 equiv, 0.30 mmol, 47 mg) in 78% yield and 96% ee (0.16 mmol, 54mg). After isolation, enantio-enriched product was obtained as a brown solid.
MP: 158-159℃
^{1}H NMR (400 MHz, $CDCl_3$) δ 7.77 (dd, *J* = 8.8, 6.4 Hz, 2H), 7.63 (dd, *J* = 8.1, 1.5 Hz, 1H), 7.54 (ddd, *J* = 8.5, 6.8, 1.5 Hz, 1H), 7.37 (ddd, *J* = 8.1, 6.8, 1.2 Hz, 1H), 7.07 (d, *J* = 2.3 Hz, 1H), 6.94 (d, *J* = 8.5 Hz, 1H), 6.86 – 6.76 (m, 1H), 6.28 (d, *J* = 8.2 Hz, 1H), 3.67 (s, 3H), 3.58 (dd, *J* = 8.9, 7.1 Hz, 1H), 3.25 (s, 3H), 2.42 (dd, *J* = 7.0, 4.5 Hz, 1H), 2.20 (s, 3H), 2.06 (dd, *J* = 8.9, 4.5 Hz, 1H).
^{13}C NMR (151 MHz, $CDCl_3$) δ 174.0, 157.1, 156.5, 134.3, 132.6, 128.9, 128.8, 128.8, 127.1, 126.5, 125.4, 123.2, 121.1, 109.6, 54.9, 52.5, 34.8, 34.2, 20.4, 20.4.
HRMS: (+p APCI) calculated for $[C_{22}H_{22}O_3N +]$ 348.1594, found 348.1594
IR(neat): 2949. 1716, 1600, 1561, 1502, 1463, 1433, 1372, 1297, 1263, 1243, 1213, 1190, 1159, 1142, 1090, 1033, 961, 911, 827, 808, 770, 732, 700, 647, 586, 503 cm^{-1}
Chiral HPLC: (AD-H, 60 min, 1 mL/min, 1% *i*-PrOH in *n*-hexane, UV 230 nm) RT: 20.15 min, 33.06 min.

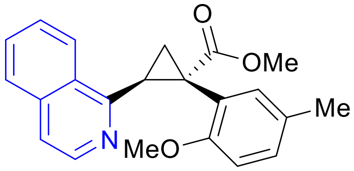

Methyl (1*S*,2*S*)-2-(isoquinolin-1-yl)-1-(2-methoxy-5-methylphenyl)cyclopropane-1-carboxylate (53)
This compound was prepared according to **General procedure 3.4** from the reaction between **36** (0.20 mmol, 44 mg) and **S29** (1.5 equiv, 30 mmol, 47 mg) in 84% yield and >99% ee (0.17 mmol, 59 mg). After isolation, enantio-enriched product was obtained as a white solid. In order to resolve enantiomers by chiral UHPLC, the product had to be reduced to the corresponding alcohol. The product (0.16 mmol 57mg) was dissolved in dry THF (1 mL) and LAH (1.5 equiv, 0.25 mmol, 0.25 mL 1.0 M solution in THF) was added dropwise to the stirring solution. The reaction was allowed to stir for 2 h before quenching with excess $Na_2SO_4 \cdot 10\ H_2O$. Quench ran for 30 min. Then the solids were filtered off and the crude residue was evaporated to dryness. The crude residue was dissolved in isopropanol, diluted with hexanes and used directly for UHPLC characterization.
MP: 118-120℃
^{1}H NMR (400 MHz, $CDCl_3$) δ 8.63 – 8.47 (m, 1H), 8.01 (d, *J* = 5.7 Hz, 1H), 7.77 – 7.69 (m, 1H), 7.65 (dq, *J* = 6.8, 4.3 Hz, 2H), 7.27 (s, 2H), 7.07 (d, *J* = 2.2 Hz, 1H), 6.78 (ddd, *J* = 8.2, 2.3, 0.8 Hz, 1H), 6.13 (d, *J* = 8.2 Hz, 1H), 4.18 (dd, *J* = 8.7, 6.9 Hz, 1H), 3.73 (s, 3H), 2.88 (broad s, 1H), 2.81 (s, 3H), 2.21 (s, 3H), 1.97 (dd, *J* = 8.7, 4.2 Hz, 1H).
^{13}C NMR (151 MHz, $CDCl_3$) δ 174.5, 156.0, 155.5, 140.6, 135.5, 132.5, 129.4, 128.8, 128.5 (2 C), 126.7, 126.4, 126.3, 122.8, 118.8, 108.7, 53.9, 52.6, 35.4, 29.9, 20.4, 18.6.
HRMS: (+p APCI) calculated for $[C_{22}H_{22}O_3N +]$ 348.1594, found 348.1593
IR(neat): 3005, 2949, 2833, 1717, 1621, 1585, 1561, 1501, 1461,1435, 1404, 1365, 1271, 1240, 1216, 1171, 1141, 1092, 1032, 992, 971, 908, 871, 822, 807, 727, 683, 647, 618, 582, 529, 505 cm^{-1}
Chiral HPLC: (AD-H, 30 min, 3 mL/min, 3% *i*-PrOH in *n*-hexane, UV 230 nm) RT: 7.98 min, 11.62 min.

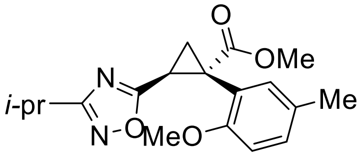

Methyl (1*S*,2*S*)-2-(3-isopropyl-1,2,4-oxadiazol-5-yl)-1-(2-methoxy-5-methylphenyl)cyclopropane-1-carboxylate (54)
This compound was prepared according to **General procedure 3.5** from the reaction between **36** (0.20 mmol, 44 mg) and **S30** (1.5 equiv, 0.30 mmol, 42 mg) in 52% yield and >99% ee (0.10 mmol, 34 mg). After isolation, enantio-enriched product was obtained as a viscous colorless oil.

^{1}H NMR (400 MHz, $CDCl_3$) δ 7.01 – 6.97 (m, 2H), 6.54 (d, J = 8.8 Hz, 1H), 3.66 (s, 3H), 3.54 (s, 3H), 3.40 (dd, J = 9.0, 6.9 Hz, 1H), 2.84 (hept, J = 6.9 Hz, 1H), 2.24 (d, J = 0.7 Hz, 3H), 2.17 (dd, J = 6.8, 4.8 Hz, 1H), 2.11 (dd, J = 9.0, 4.8 Hz, 1H), 1.07 (dd, J = 11.2, 6.9 Hz, 6H);
^{13}C NMR (101 MHz, $CDCl_3$) δ 176.4, 174.4, 172.6, 156.5, 132.2, 129.7, 129.2, 122.1, 109.8, 55.2, 52.9, 34.5, 26.4, 23.0, 21.1, 20.6, 20.4, 20.0;
HRMS (ESI) m/z calculated for $[C_{18}H_{23}N_2O_4+H]^+$, 331.1652; found 331.1659.
Chiral SFC: (ChiralPak-IB N-3 3μm column, 3 mL / minute, 5-30% *i*PrOH / CO_2 with DEA additive over 5 min, RT: 1.39 min, 1.75 min.

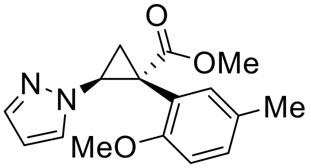

Methyl (1*R*,2*S*)-1-(2-methoxy-5-methylphenyl)-2-(*1H*-pyrazol-1-yl)cyclopropane-1-carboxylate (55)
This compound was prepared according to **General procedure 3.5** from the reaction between **36** (0.20 mmol, 44 mg) and ***N*-vinylpyrazole** (1.5 equiv, 0.30 mmol, 28 mg, Enamine) in 82% yield and 96% ee (0.16 mmol, 47 mg). After isolation, enantio-enriched product was obtained as a viscous colorless oil.
^{1}H NMR (400 MHz, $CDCl_3$) δ 7.22 (ddd, J = 3.9, 2.1, 0.7 Hz, 2H), 6.94 (dtd, J = 8.3, 1.4, 0.7 Hz, 1H), 6.85 – 6.80 (m, 1H), 6.57 (d, J = 8.3 Hz, 1H), 5.99 (dd, J = 2.4, 1.8 Hz, 1H), 4.65 (dd, J = 8.7, 5.6 Hz, 1H), 3.69 (s, 3H), 3.65 (d, J = 0.5 Hz, 3H), 2.50 (t, J = 5.8 Hz, 1H), 2.16 (t, J = 0.7 Hz, 3H), 2.08 (dd, J = 8.7, 6.0 Hz, 1H);
^{13}C NMR (101 MHz, $CDCl_3$) δ 172.9, 156.7, 138.4, 131.7, 129.9, 129.5, 129.3, 121.5, 109.9, 105.5, 55.2, 52.5, 45.1, 32.6, 20.4, 18.7;
HRMS (ESI) m/z calculated for $[C_{16}H_{19}N_2O_3 +H]^+$, 287.1390; found 287.1395.
Chiral SFC: (Lux i-Cellulose-5 (IC) column, 3 mL / minute, 5-50% MeOH / CO_2 over 5 min, RT: 1.40 min, 1.56 min.

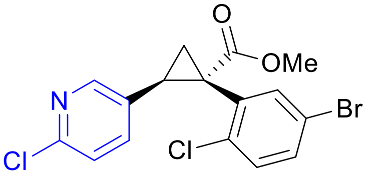

Methyl (1*S*,2*R*)-1-(5-bromo-2-chlorophenyl)-2-(6-chloropyridin-3-yl)cyclopropane-1-carboxylate (56)
This compound was prepared according to **General procedure 3.4** from the reaction between **S12** (0.20 mmol, 58 mg) and **35** (1.5 equiv, 0.30 mmol, 42 mg) in 98% yield and 93% ee (0.20 mmol, 78 mg). After isolation, enantio-enriched product was obtained as a white solid.
MP: 96-99 ℃
^{1}H NMR (400 MHz, $CDCl_3$) δ 8.07 – 7.91 (s, 1H), 7.52 (s, 1H), 7.32 (dd, *J* = 8.5, 2.4 Hz, 1H), 7.11 – 7.00 (m, 2H), 6.96 (dd, *J* = 8.3, 2.2 Hz, 1H), 3.71 (s, 3H), 3.33 (t, *J* = 7.9 Hz, 1H), 2.14 (s, 1H), 1.90 (dd, *J* = 7.3, 5.5 Hz, 1H).
^{13}C NMR (151 MHz, $CDCl_3$) δ 171.9, 149.7, 149.3, 137.0, 135.8, 135.0, 134.6, 132.4, 131.0, 130.2, 122.9, 120.2, 53.0, 36.7, 29.6, 20.8.
HRMS: (+p APCI) calculated for $[C_{16}H_{13}O_2N^{79}Br^{35}Cl_2 +]$ 399.9501 found 399.9501
IR(neat): 3016, 2951, 1722, 1586, 1560, 1465, 1434, 1365, 1350, 1270, 1248, 1208, 1166, 1141, 1114, 1045, 1024, 970, 909, 892, 815, 741, 730, 647, 634, 533 cm^{-1}
Chiral HPLC: (OD-H, 60 min, 1 mL/min, 1% *i*-PrOH in *n*-hexane, UV 230 nm) RT: 26.38 min, 29.94 min.

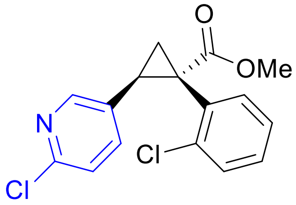

Methyl (1*S*,2*R*)-1-(2-chlorophenyl)-2-(6-chloropyridin-3-yl)cyclopropane-1-carboxylate (57)
This compound was prepared according to **General procedure 3.4** from the reaction between **S5** (0.20 mmol, 42 mg) and **35** (1.5 equiv, 0.30 mmol, 42 mg) in 75% yield and 92% ee (0.15 mmol, 49 mg). After isolation, enantio-enriched product was obtained as a clear colorless oil.
^{1}H NMR (400 MHz, $CDCl_3$) δ 7.98 (d, *J* = 2.5 Hz, 1H), 7.49 – 7.27 (m, 1H), 7.25 – 7.12 (m, 3H), 6.94 (d, *J* = 8.3 Hz, 1H), 6.87 (dd, *J* = 8.3, 2.6 Hz, 1H), 3.68 (s, 3H), 3.30 (t, *J* = 8.4 Hz, 1H), 2.11 (t, *J* = 8.2 Hz, 1H), 1.89 (dd, *J* = 7.3, 5.4 Hz, 1H).
^{13}C NMR (151 MHz, $CDCl_3$) δ 172.8, 149.6, 149.7, 137.2, 137.1, 132.5, 130.9, 129.9, 129.6, 126.9, 123.0, 53.1, 37.0, 29.7
HRMS: (+p APCI) calculated for $[C_{16}H_{14}O_2N^{35}Cl_2+]$ 322.0396, found 322.0399
IR(neat): 2923, 2852, 1720, 1586, 1561, 1464, 1434, 1397, 1348, 1269, 1250, 1208, 1165, 1143, 1110, 1034, 1023, 992, 968, 909, 885, 833, 802, 780, 741, 699, 660, 647, 586, 518, 470, 434 cm^{-1}
Chiral HPLC: (OD-H, 60 min, 1 mL/min, 1% *i*-PrOH in *n*-hexane, UV 230 nm) RT: 28.71 min, 32.79 min

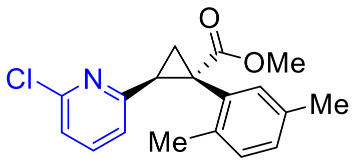

Methyl (1*S*,2*S*)-2-(6-chloropyridin-2-yl)-1-(2,5-dimethylphenyl)cyclopropane-1-carboxylate (58)
This compound was prepared according to **General procedure 3.5: S11** (0.20 mmol, 41 mg) and **66** (1.5 equiv, 0.30 mmol, 42 mg) in 74% yield and >99% ee (0.15 mmol, 47 mg). After isolation, enantio-enriched product was obtained as a clear colorless oil.
^{1}H NMR (500 MHz, $CDCl_3$) δ 7.29 – 7.05 (m, 2H), 6.97 (d, J = 7.8 Hz, 1H), 6.93 – 6.86 (m, 1H), 6.80 (s, 1H), 6.50 (d, J = 7.1 Hz, 1H), 3.66 (s, 3H), 3.46 – 3.32 (m, 1H), 2.48 – 1.82 (m, 8H);
^{13}C NMR (101 MHz, $CDCl_3$) δ 173.5, 157.6, 149.8, 137.7, 136.0, 134.6, 132.2, 129.6, 128.3, 121.4, 120.2, 52.6, 33.8, 21.7, 20.9, 18.8;
HRMS (ESI) m/z calculated for $C_{18}H_{19}ClNO_2$ [M+H]+, 316.1104; found 316.1097.
Chiral SFC: (ChiralPakIC column, 3 mL / minute, 5-30% MeOH / CO_2 over 10 min, RT: 2.15 min, 2.57 min.

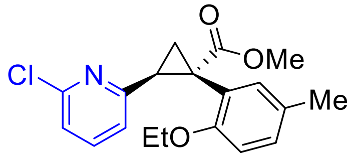

Methyl (1*S*,2*S*)-2-(6-chloropyridin-2-yl)-1-(2-ethoxy-5-methylphenyl)cyclopropane-1-carboxylate (59)
This compound was prepared according to **General procedure 3.5: S21** (0.20 mmol, 47 mg) and **66** (1.5 equiv, 0.30 mmol, 42 mg) in 82% yield and 99% ee (0.16 mmol, 56 mg). After isolation, enantio-enriched product was obtained as a clear colorless oil.
^{1}H NMR (500 MHz, $CDCl_3$) δ 7.25 (t, J = 7.8 Hz, 1H), 7.01 (d, J = 2.4 Hz, 1H), 6.92 – 6.87 (m, 2H), 6.70 (dd, J = 7.8, 0.8 Hz, 1H), 6.40 (d, J = 8.2 Hz, 1H), 3.79 (dq, J = 8.7, 7.0 Hz, 1H), 3.64 (s, 3H), 3.41 (ddd, J = 15.9, 9.1, 7.0 Hz, 2H), 2.27 – 2.20 (m, 4H), 1.94 (dd, J = 8.9, 4.8 Hz, 1H), 1.24 (t, J = 7.0 Hz, 3H);
^{13}C NMR (101 MHz, $CDCl_3$) δ 173.9, 158.0, 155.7, 149.4, 137.3, 132.5, 129.0, 128.7, 122.6, 121.0, 120.9, 110.1, 62.9, 52.4, 34.9, 32.8, 20.4, 20.1, 14.7;
HRMS (ESI) m/z calculated for $C_{19}H_{21}ClNO_3$ [M+H]+, 346.1215; found 346.1208.
Chiral SFC: (ChiralPakIC column, 3 mL / minute, 5-30% MeOH / CO_2 over 10 min, RT: 2.63 min, 2.96 min.

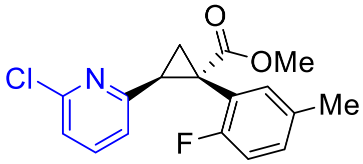

Methyl (1*S*,2*S*)-2-(6-chloropyridin-2-yl)-1-(2-fluoro-5-methylphenyl)cyclopropane-1-carboxylate (60)
This compound was prepared according to **General procedure 3.5: S22** (0.20 mmol, 42 mg) and **66** (1.5 equiv, 0.30 mmol, 42 mg) in 81% yield and >99% ee (0.16 mmol, 52 mg). After isolation, enantio-enriched product was obtained as a clear colorless oil.
^{1}H NMR (500 MHz, $CDCl_3$) δ 7.37 (t, J = 7.8 Hz, 1H), 7.02 – 6.93 (m, 3H), 6.93 – 6.88 (m, 1H), 6.62 (dd, J = 9.9, 8.3 Hz, 1H), 3.68 (s, 3H), 3.34 (dd, J = 8.9, 7.0 Hz, 1H), 2.35 (dd, J = 7.0, 4.7 Hz, 1H), 2.24 (d, J = 1.0 Hz, 3H), 2.06 (dd, J = 8.9, 4.7 Hz, 1H); **^{13}C NMR** (101 MHz, $CDCl_3$) δ 173.0, 161.5, 159.1, 156.8, 149.9, 138.0, 133.1 (d, *J* = 3.7 Hz), 129.6 (d, *J* = 8.1 Hz),121.9, 121.6, 114.3, 114.1, 52.8, 33.6, 33.1, 20.5, 19.5.
^{19}F NMR (376 MHz, $CDCl_3$) δ -119.37 (dt, *J* = 10.9, 6.0 Hz).
HRMS (ESI) m/z calculated for $C_{17}H_{16}ClFNO_2$ [M+H]+, 320.0859; found 320.0857.
Chiral SFC: ChiralPak-IB column, 3 mL / minute, 5-50% MeOH / CO_2 over 10 min, RT: 0.96 min, 1.07 min.

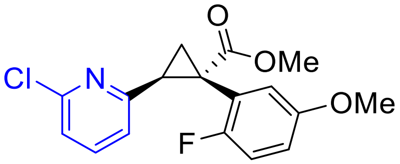

Methyl (1*S*,2*S*)-2-(6-chloropyridin-2-yl)-1-(2-fluoro-5-methoxyphenyl)cyclopropane-1-carboxylate (61)
This compound was prepared according to **General procedure 3.5: S23** (0.20 mmol, 45 mg) and **66**(1.5 equiv, 0.30 mmol, 42 mg) in 71% yield and >99% ee (0.14 mmol, 48 mg). After isolation, enantio-enriched product was obtained as a white solid.
^{1}H NMR (400 MHz, $CDCl_3$) δ 7.38 (t, J = 7.7 Hz, 1H), 7.02 6.93 (m, 2H), 6.72 – 6.59 (m, 3H), 3.70 (s, 3H), 3.69 (s, 3H), 3.34 (dd, J = 9.0, 7.0 Hz, 1H), 2.34 (dd, J = 7.0, 4.7 Hz, 1H), 2.07 (dd, J = 8.9, 4.7 Hz, 1H);
^{13}C NMR (101 MHz, $CDCl_3$) δ 172.7, 157.8, 156.7, 155.5, 155.0 (d, *J* = 2.0 Hz), 150.0, 138.0, 121.9, 121.6, 117.6 (d, *J* = 3.7 Hz), 115.2, 114.9, 114.5 (d, *J* = 8.2 Hz), 55.8, 52.7, 33.3, 19.7.

^{19}F NMR (376 MHz, $CDCl_3$) δ -124.68 (dt, *J* = 8.6, 5.5 Hz).
HRMS (ESI) m/z calculated for $[C_{17}H_{16}ClFNO_3 +H]+$, 336.0808; found 336.0808.
Chiral SFC: Lux i-Cellulose column, 3 mL / minute, 5-25% IPA / CO_2 over 10 min, RT: 2.08 min, 2.40 min.

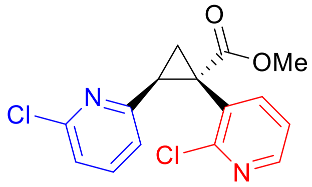

Methyl (1*S*,2*S*)-2-(6-chloropyridin-2-yl)-1-(2-chloropyridin-3-yl)cyclopropane-1-carboxylate (62)
This compound was prepared according to **General procedure 3.4** from the reaction between **S20** (0.20 mmol, 42 mg) and **66** (1.5 equiv, 30 mmol, 42 mg) in 87% yield and 85% ee (0.17 mmol, 56 mg). After isolation, enantio-enriched product was obtained as a clear colorless oil.
^{1}H NMR (400 MHz, $CDCl_3$) δ 8.21 (ddd, *J* = 10.3, 4.9, 1.9 Hz, 2H), 7.32 (broad s, 1H), 7.03 (broad s, 1H), 6.88 (broad s, 1H), 6.65 (broad s, 1H), 3.72 (s, 3H), 3.65 (broad s, 1H), 2.33 (broad s, 1H), 1.90 (dd, *J* = 7.8, 5.7 Hz, 1H).
^{13}C NMR (151 MHz, $CDCl_3$) δ 171.7, 154.1, 153.4, 148.6 (2 C), 147.8, 141.6, 129.6, 121.9 (2 C), 121.7, 53.1, 29.6, 25.3, 20.9.
HRMS: (+p APCI) calculated for $[C_{15}H_{13}O_2N_2{}^{35}Cl_2+]$ 323.0348, found 323.0340
IR(neat): 1724, 1563, 1436, 1411, 1398, 1357, 1268, 1220, 1196, 1166, 1132, 1063, 756, 732, 682cm^{-1}
Chiral HPLC: (AD-H, 30 min, 1 mL/min, 1% *i*-PrOH in *n*-hexane, UV 230 nm) RT: 10.65 min, 11.71 min.

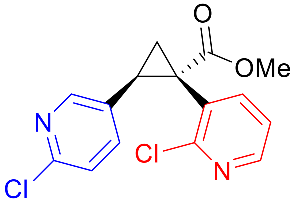

Methyl (1*S*,2*R*)-1-(2-chloropyridin-3-yl)-2-(6-chloropyridin-3-yl)cyclopropane-1-carboxylate (63)
This compound was prepared according to **General procedure 3.4** from the reaction between **S20** (0.20 mmol, 42 mg) and **35** (1.5 equiv, 30 mmol, 42 mg) in 79% yield and 84% ee (0.16 mmol, 51 mg). After isolation, enantio-enriched product was obtained as a clear colorless oil.
^{1}H NMR (600 MHz, $CDCl_3$) δ 8.27 (s, 1H), 7.98 (s, 1H), 7.19 (s, 1H), 7.03 (s, 3 H), 3.70 (s, 3H), 3.36 (m, 1 H), 2.18 (broad S, 1H), 1.69 (broad s, 1H)
^{13}C NMR (151 MHz, $CDCl_3$) δ 171.7, 153.9, 149.9, 149.1, 148.8, 137.5, 129.4, 123.1, 122.1, 53.0, 36.0, 29.6, 29.6, 25.3
HRMS: (+p APCI) calculated for $[C_{15}H_{13}O_2N_2{}^{35}Cl_2+]$ 323.0348, found 323.0349
IR(neat): 3016, 2970, 2950, 1721, 1562, 1464, 1434, 1397, 1348, 1265, 1221, 1165, 1132, 1108, 1058, 1023, 967, 911, 835, 801, 779, 754, 728, 659, 647, 633, 437 cm^{-1}
Chiral HPLC: (AD-H, 60 min, 1 mL/min, 1% *i*-PrOH in *n*-hexane, UV 230 nm) RT: 29.56 min, 34.74 min.

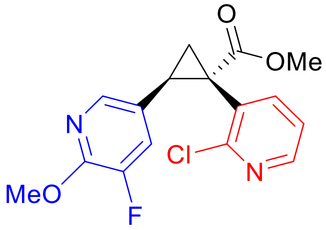

Methyl (1*S*,2*R*)-1-(2-chloropyridin-3-yl)-2-(5-fluoro-6-methoxypyridin-3-yl)cyclopropane-1-carboxylate (64)
This compound was prepared according to **General procedure 3.4** from the reaction between **S20** (0.20 mmol, 42 mg) and **S26** (1.5 equiv, 30 mmol, 46 mg) in 74% yield and 72% ee (0.15 mmol, 50 mg). After isolation, enantio-enriched product was obtained as clear colorless oil.
^{1}H NMR (400 MHz, $CDCl_3$) δ 8.25 (dd, *J* = 4.8, 1.9 Hz, 1H), 7.58 (d, *J* = 2.1 Hz, 1H), 7.48 (d, *J* = 48.6 Hz, 1H), 7.17 (s, 1H), 6.67 (dd, *J* = 11.2, 2.1 Hz, 1H), 3.92 (s, 3H), 3.69 (s, 3H), 3.31 (t, *J* = 8.4 Hz, 1H), 2.14 (s, 1H), 1.81 (dd, *J* = 7.4, 5.6 Hz, 1H).
^{13}C NMR (151 MHz, $CDCl_3$) δ 172.0, 154.0, 152.3 (d, *J* = 11.5 Hz), 148.6, 147.4, 145.7, 140.8 (d, *J* = 5.7 Hz), 129.8, 124.5, 122.1, 122.0, 121.9, 53.7, 52.9, 35.6, 29.7.
^{19}F NMR (376 MHz, $CDCl_3$) δ -140.38 (d, *J* = 11.2 Hz).
HRMS: (+p APCI) calculated for $[C_{16}H_{15}O_3N_2{}^{35}ClF +]$ 337.0749, found 337.0747
IR(neat): 2952, 1720, 1617, 1579, 1564, 1496, 1437, 1413, 1398, 1264, 1219, 1196, 1169, 1140, 1059, 959, 909, 776, 751, 731, 654, 564, 503 cm^{-1}
Chiral HPLC: (AD-H, 80 min, 1 mL/min, 1% *i*-PrOH in *n*-hexane, UV 230 nm) RT: 40.39 min, 66.59 min

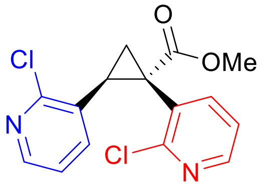

Methyl (1*S*,2*S*)-1,2-bis(2-chloropyridin-3-yl)cyclopropane-1-carboxylate (65)
This compound was prepared according to **General procedure 3.4** from the reaction between **S20** (0.20 mmol, 42 mg) and **S25** (1.5 equiv, 30 mmol, 42 mg) in 65% yield and 95% ee (0.13 mmol, 42 mg). After isolation, enantio-enriched product was obtained as a clear colorless oil.
^{1}H NMR (400 MHz, $CDCl_3$) δ 8.20 (dd, *J* = 4.8, 1.9 Hz, 1H), 7.77 (s, 1H), 7.43 (t, *J* = 7.7 Hz, 1H), 7.20 (broad s, 1H), 6.94 (d, *J* = 7.9 Hz, 1H), 3.69 (s, 3H), 3.49 – 3.40 (m, 1H), 2.44 (broad s, 1H), 2.05 (broad s, 1H).
^{13}C NMR (151 MHz, $CDCl_3$) δ 172.1, 155.7, 153.3, 149.9, 148.1, 141.8, 138.1, 130.1, 123.6, 121.8, 52.9, 37.0, 33.0, 29.7, 22.7, 20.9.
HRMS: (+p APCI) calculated for $[C_{15}H_{13}O_2N_2{}^{35}Cl_2+]$ 323.0348, found 323.0346
IR(neat): 1722, 1583, 1558, 1434, 1398, 1377, 1265, 1220, 1162, 1132, 1096, 1058, 991, 941, 910, 810, 796, 770, 752, 730, 714, 654 cm^{-1}
Chiral HPLC: (AD-H, 30 min, 1 mL/min, 1% *i*-PrOH in *n*-hexane, UV 230 nm) RT: 22.81 min, 24.16 min.

5. Determination of enantioselectivity

5.1: Diastereoselectivity of *R*-pantolactonate cyclopropanes.

Diastereoselectivity of these products was determined through analysis of the crude cyclopropanation products via ^{1}H NMR. The reaction has the potential to form 4 stereoisomers. 2 arise from the enantiocontrol of the pantolactonate, and 2 arise from the cis/trans diastereoselectivity inherent to donor-acceptor rhodium carbenes. In-order to resolve the identity of these stereoisomers, a control experiment may be performed in the presence of a chiral catalyst. One of the isomers of the chiral catalyst will oppose the enantioselectivity imparted by the pantolactonate, leading to increased production of the minor-stereoisomer that would arise from imperfect enantiocontrol by the pantolactonate. However, due to the inherent cis/trans diastereoselectivity of donor/acceptor carbenes, the ratio of cis/trans stereoisomers is not affected. By comparing distinctive peaks in the stereo-scrambled product to the crude reaction mixture of the chiral reaction the asymmetric induction of the chiral-auxiliary reaction may be determined unambiguously (**Figure s4**).

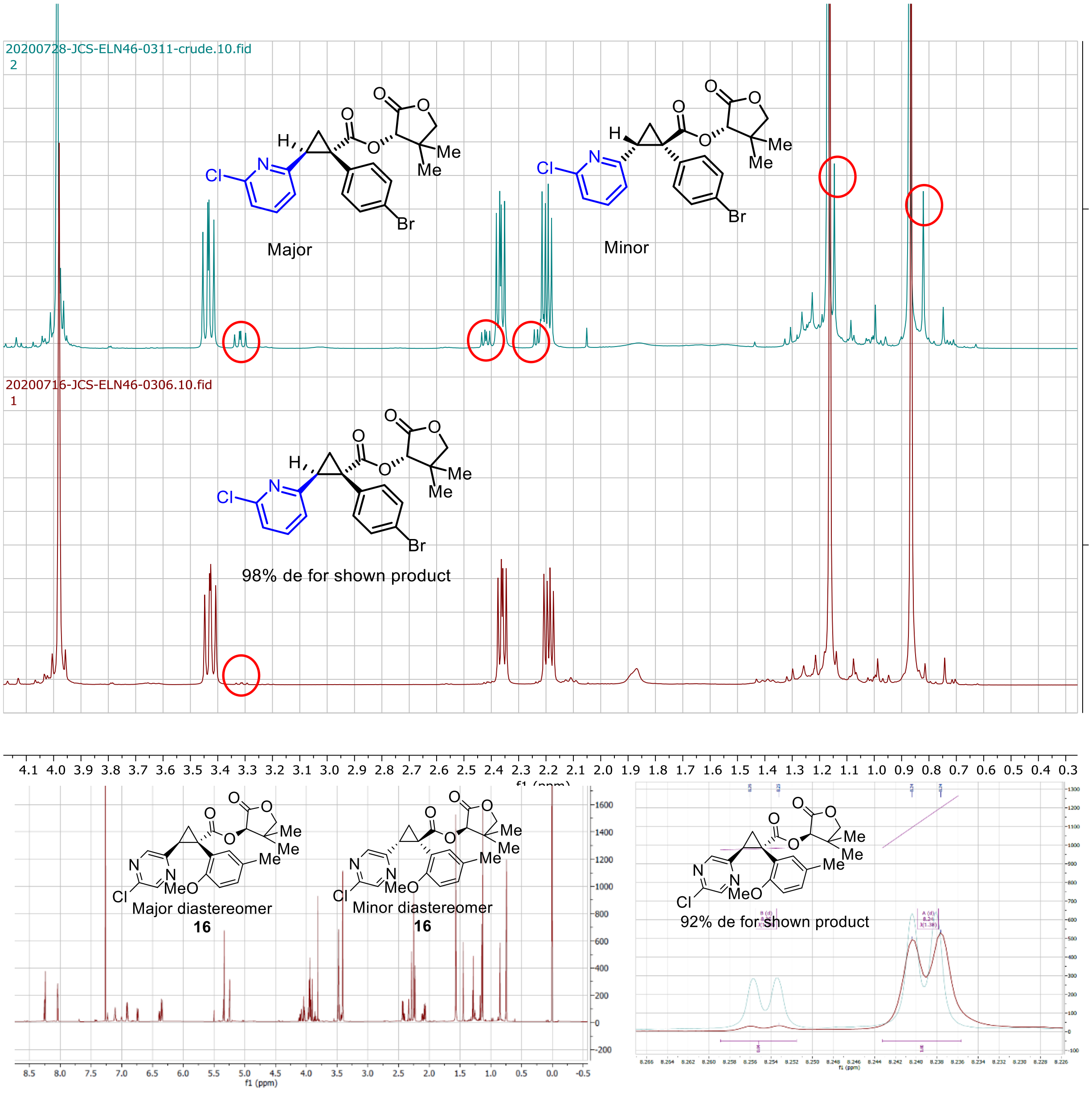

Figure S4a. Determination of diastereomeric excess of compound **9** based on identification of peaks in the reaction crude (Red) corresponding to the minor stereo-isomer due to imperfect enantiocontrol. The benzylic cyclopropyl proton gives the clearest determination of %de due to its separation from the major stereoisomer in the crude spectrum. Regardless several characteristic peaks of the minor stereoisomer are observed in the stereo-scrambled product (Blue) as circled in red. The scrambled product was obtained from the reaction between **S17** and **66** in the presence of a 1:1 mixture of Rh2(S-DOSP)4 and Rh2(R-DOSP)4 (1.0 mol % total catalyst loading), in CH_2Cl_2 at rt for 24hr. Diastereomeric excess is determined by subtracting the integration of the minor stereoisomer from the normalized integration of the major stereoisomer. **S4b.** For compounds bearing an *ortho*-substituent, comparison of protons in the aryl region corresponding to the azaheterocycle may be used for determination of diastereomeric excess due to the lack of surrounding clutter. Diastereo-scrambled product products were prepared using blue-light to generate the carbene in absence of a chiral environment, the example shown is the reaction between **S18** and **2-chloro-5-ethenyl-pyrimidine** (Enamine) in CH_2Cl_2 under blue light irradiation at rt for 24hr.

5.1.1: Crude ^{1}H NMRs of *R*-pantolactonate cyclopropanes for %d.e determination:

Compound 8

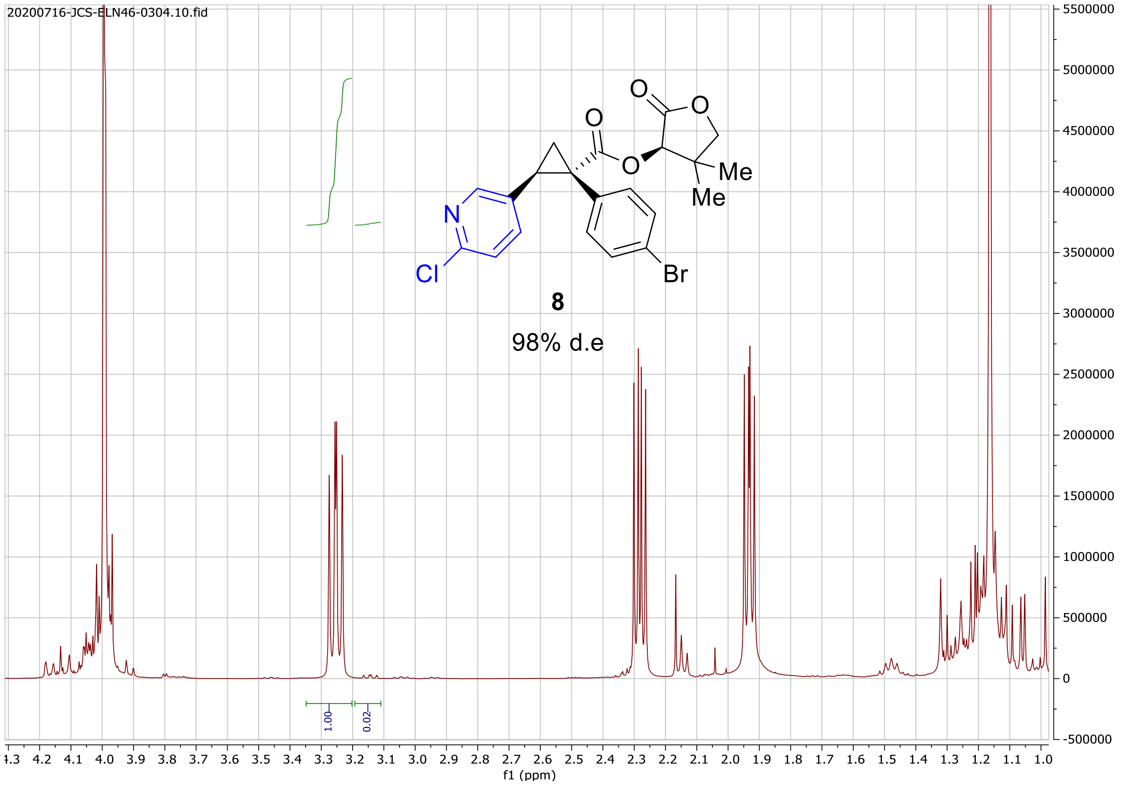

Compound 9

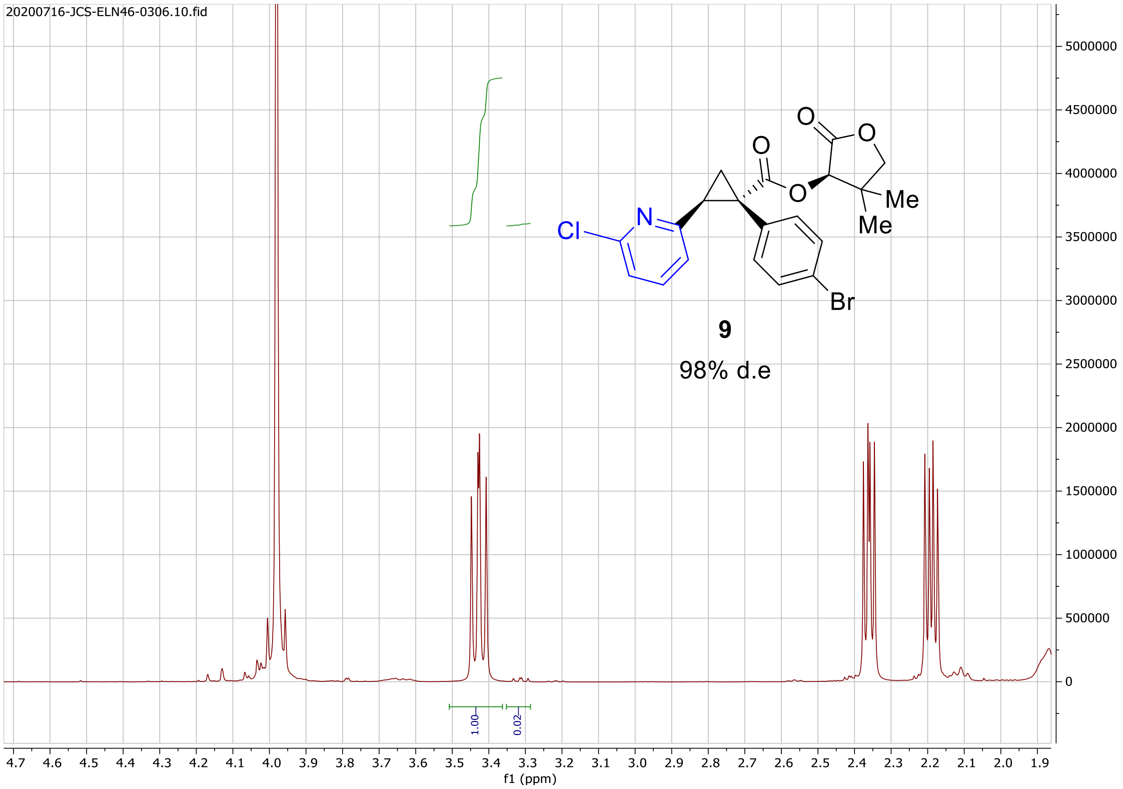

Compound 10

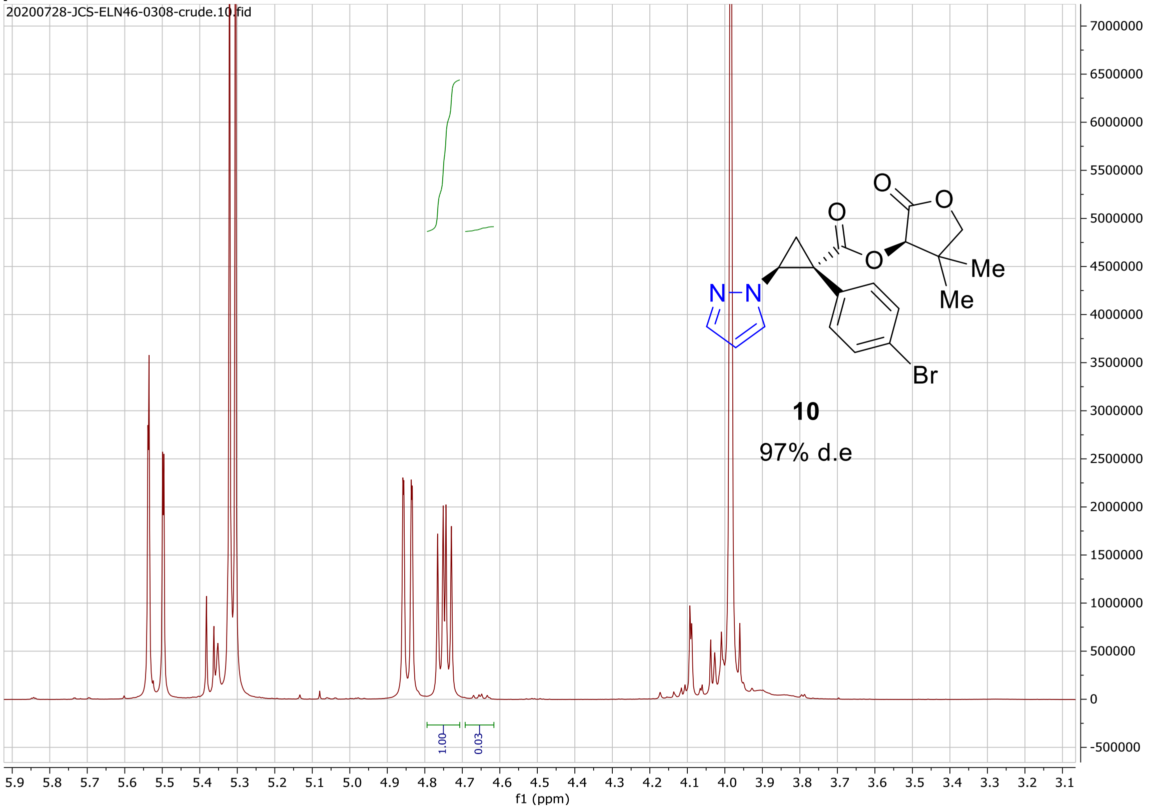

Compound 11

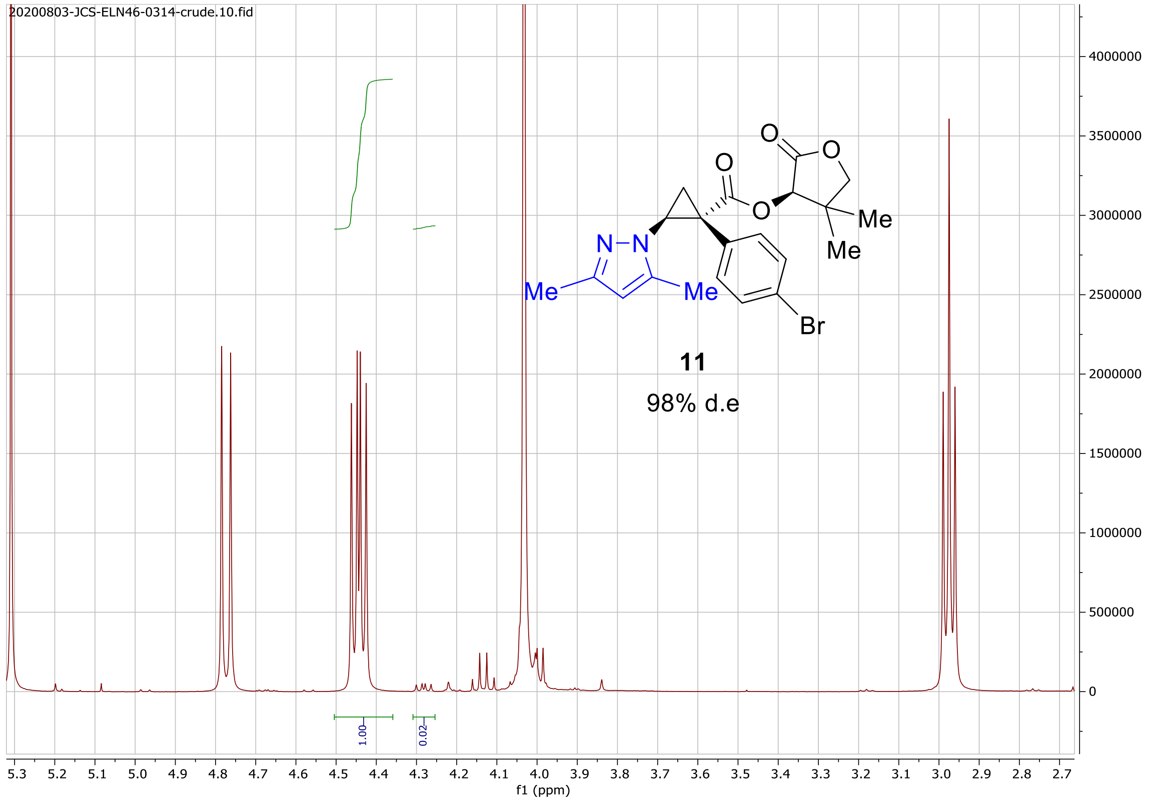

Compound 12:

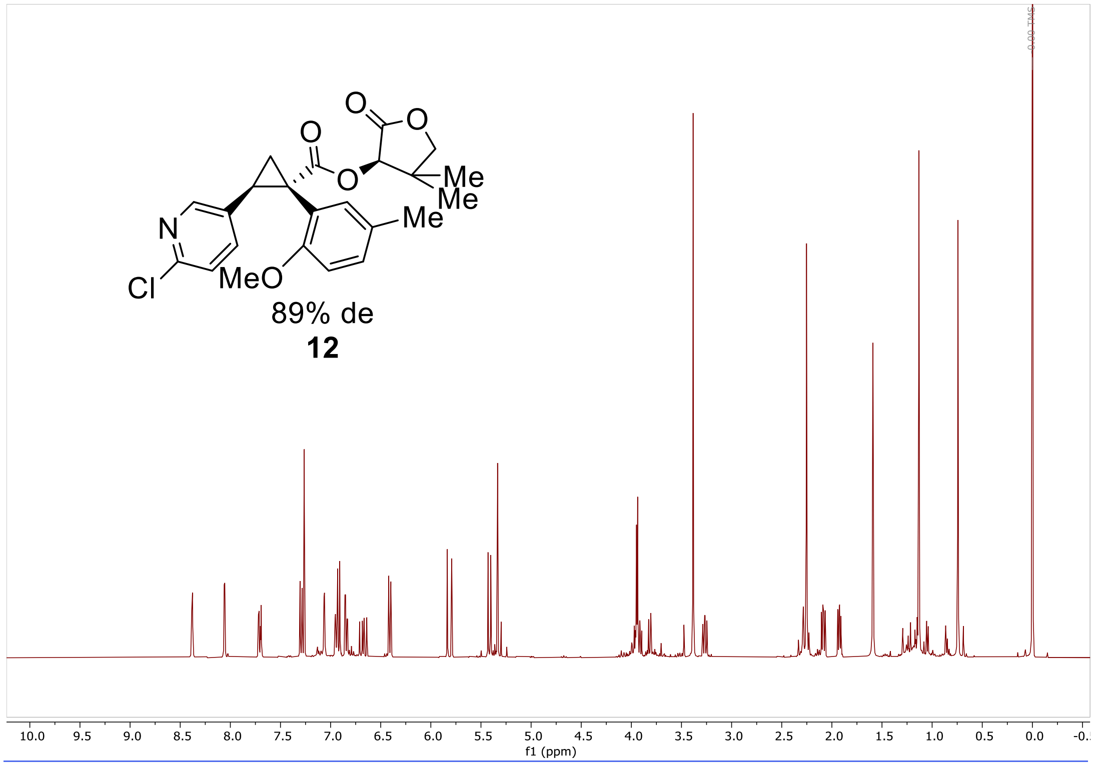

O
O
O
O
Me
Me
Me
N
Cl
MeO
89% de
12
10.0
9.5
9.0
8.5
8.0
7.5
7.0
6.5
6.0
5.5
5.0
4.5
4.0
3.5
3.0
2.5
2.0
1.5
1.0
0.5
0.0
f1 (ppm)

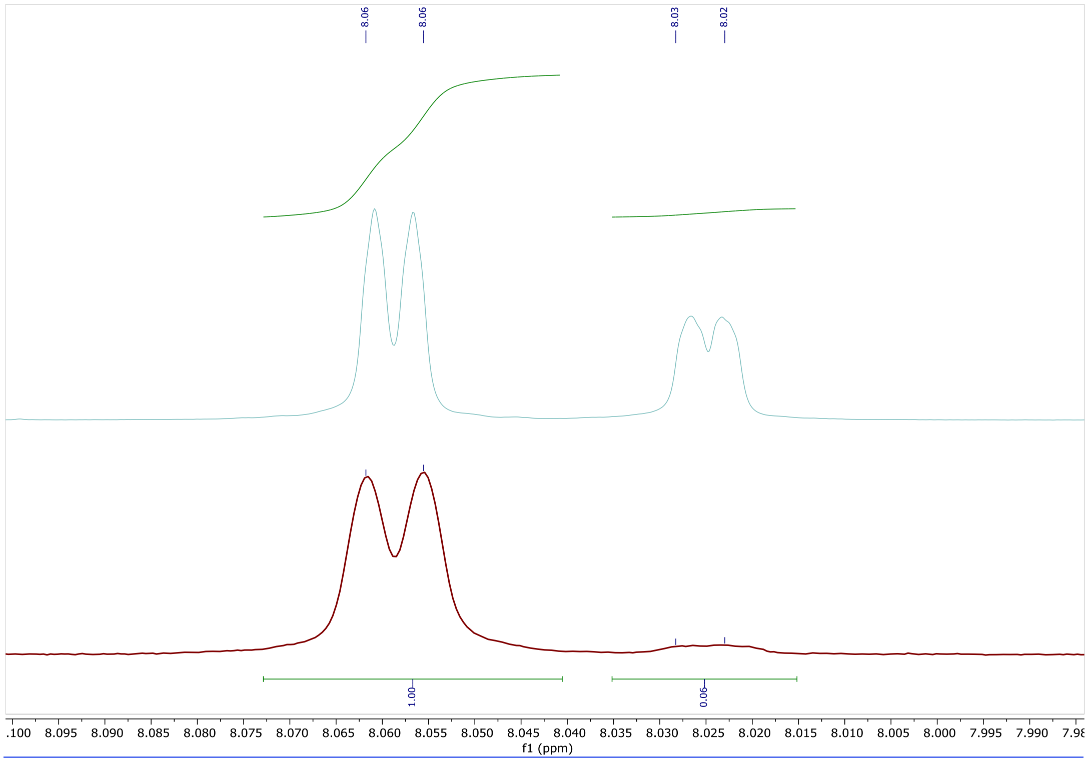

8.06
8.06
8.03
8.02
1.00
0.06
8.095
8.090
8.085
8.080
8.075
8.070
8.065
8.060
8.055
8.050
8.045
8.040
8.035
8.030
8.025
8.020
8.015
8.010
8.005
8.000
7.995
7.990
f1 (ppm)

Compound 13:

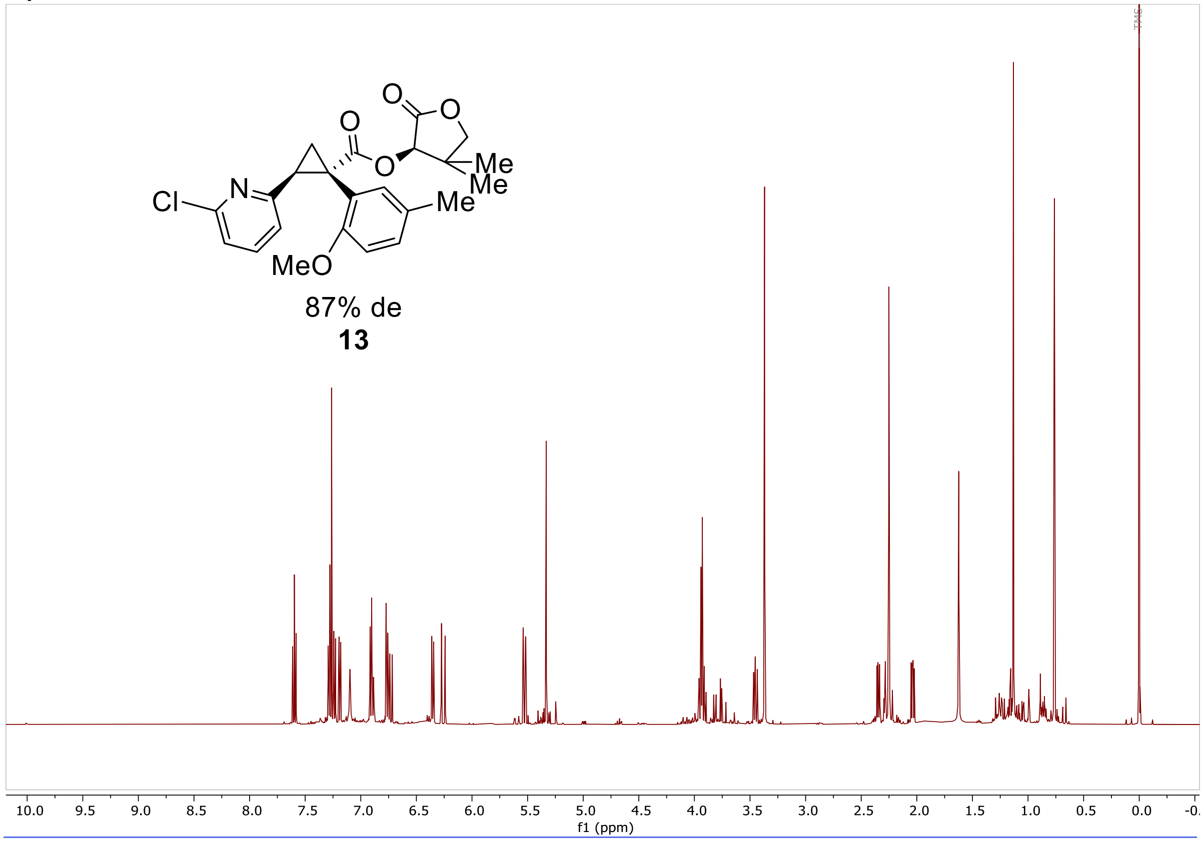

Cl
N
O
O
O
O
Me
Me
Me
MeO
87% de
13
TMS
10.0
9.5
9.0
8.5
8.0
7.5
7.0
6.5
6.0
5.5
5.0
4.5
4.0
3.5
3.0
2.5
2.0
1.5
1.0
0.5
0.0
f1 (ppm)

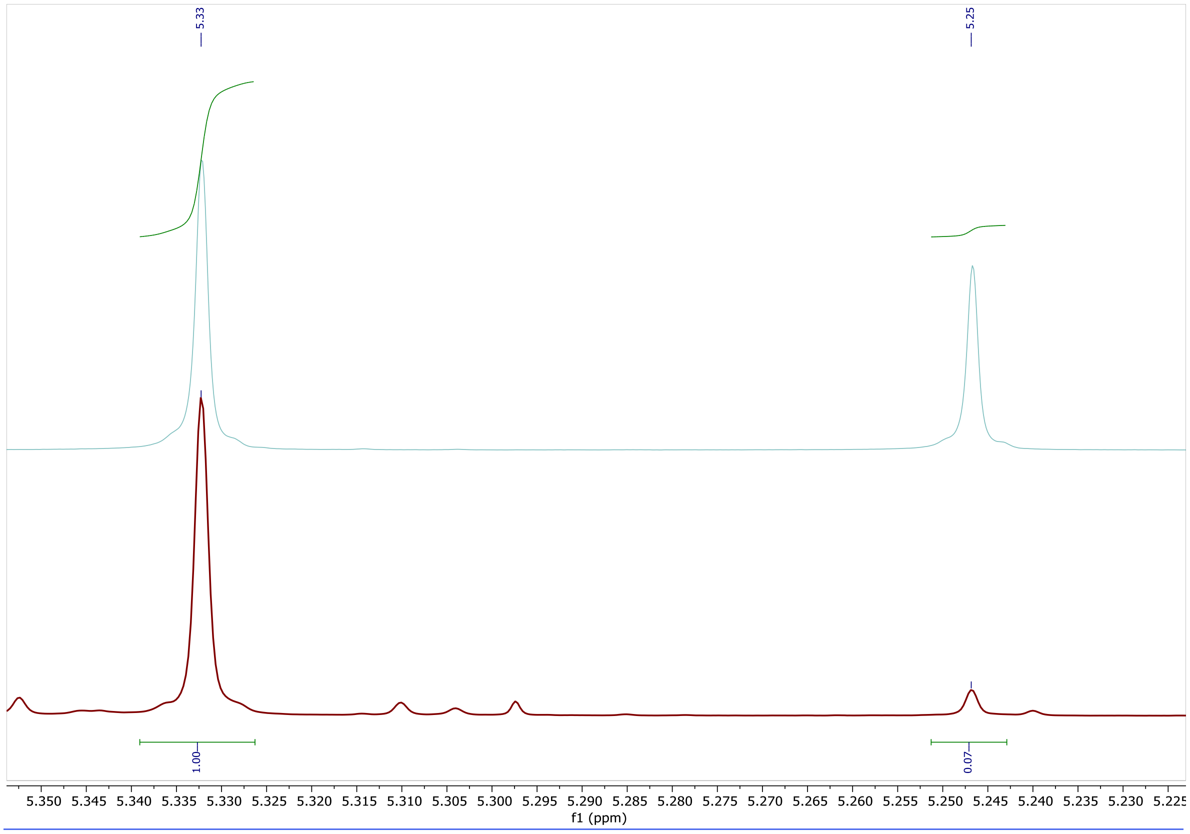

Compound 14:

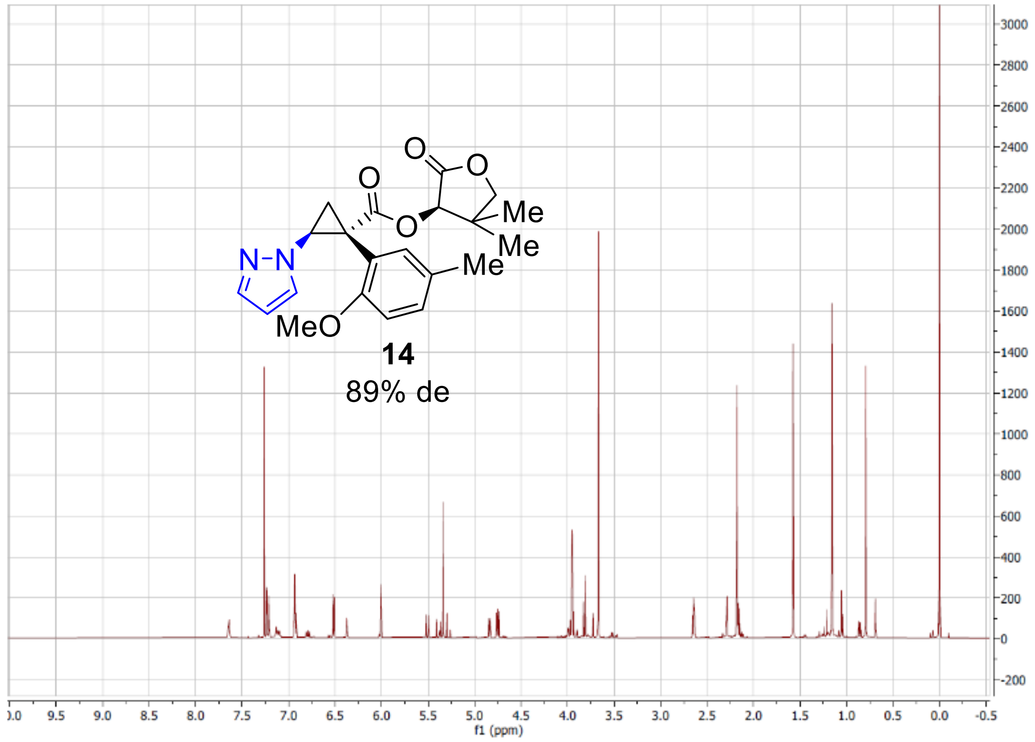

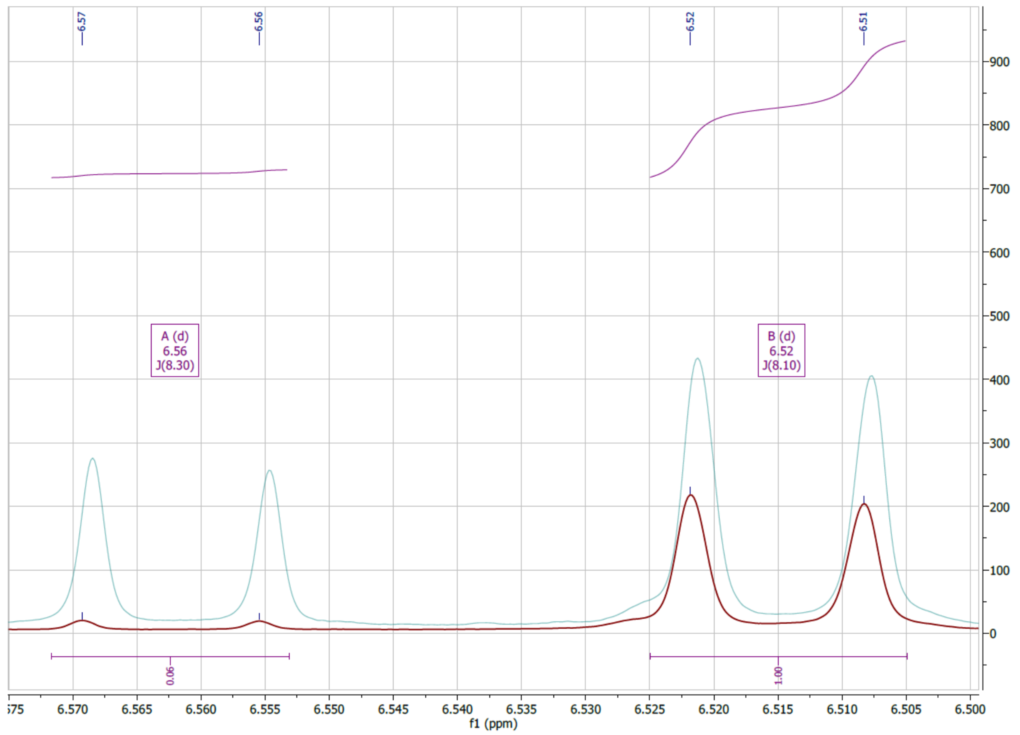

Compound 15:

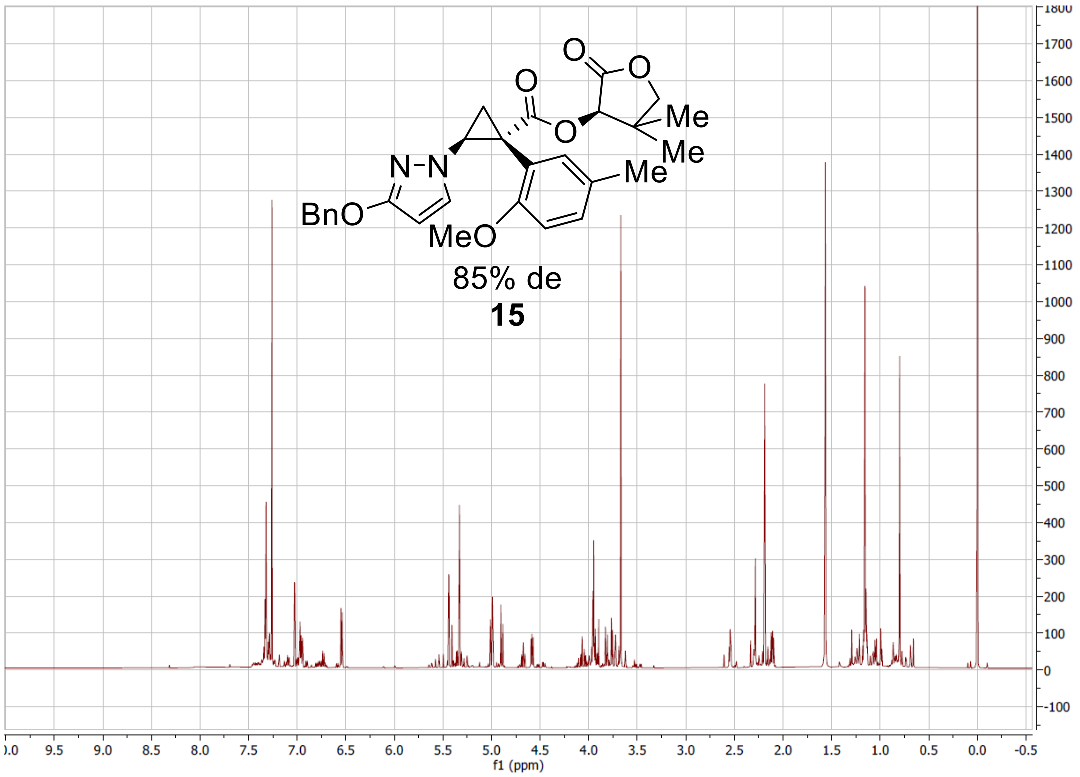
BnO
MeO
Me
Me
Me
85% de
15
f1 (ppm)

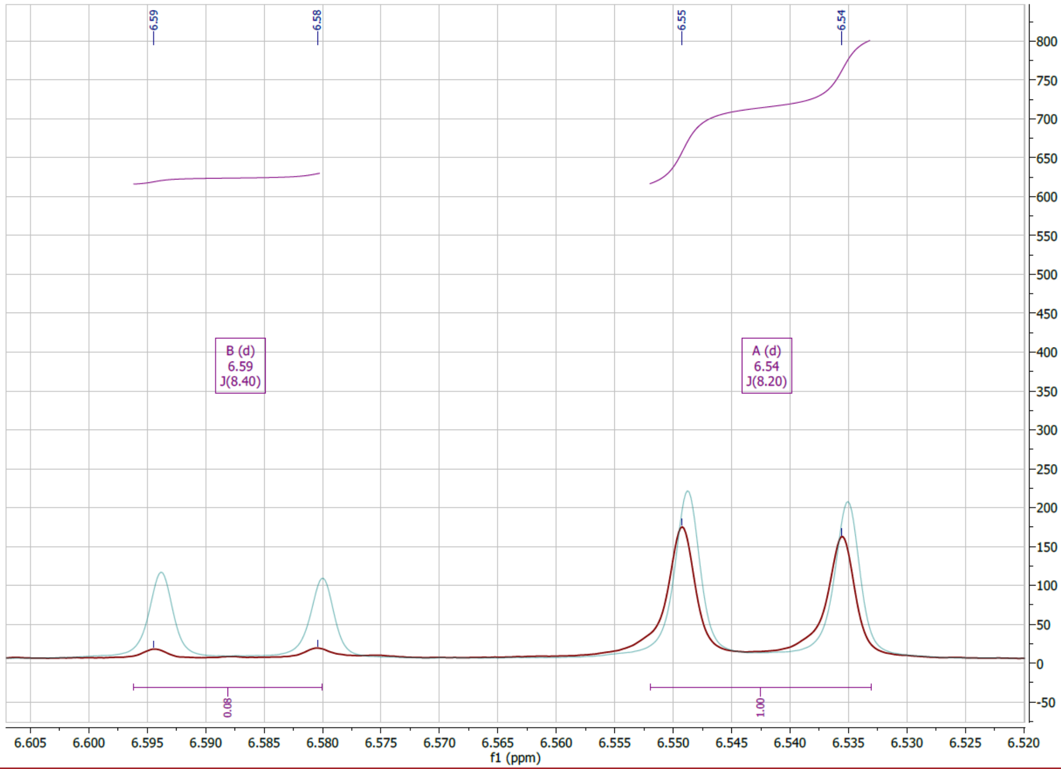
B (d)
6.59
J(8.40)
A (d)
6.54
J(8.20)
0.08
1.00
f1 (ppm)

Compound 16:

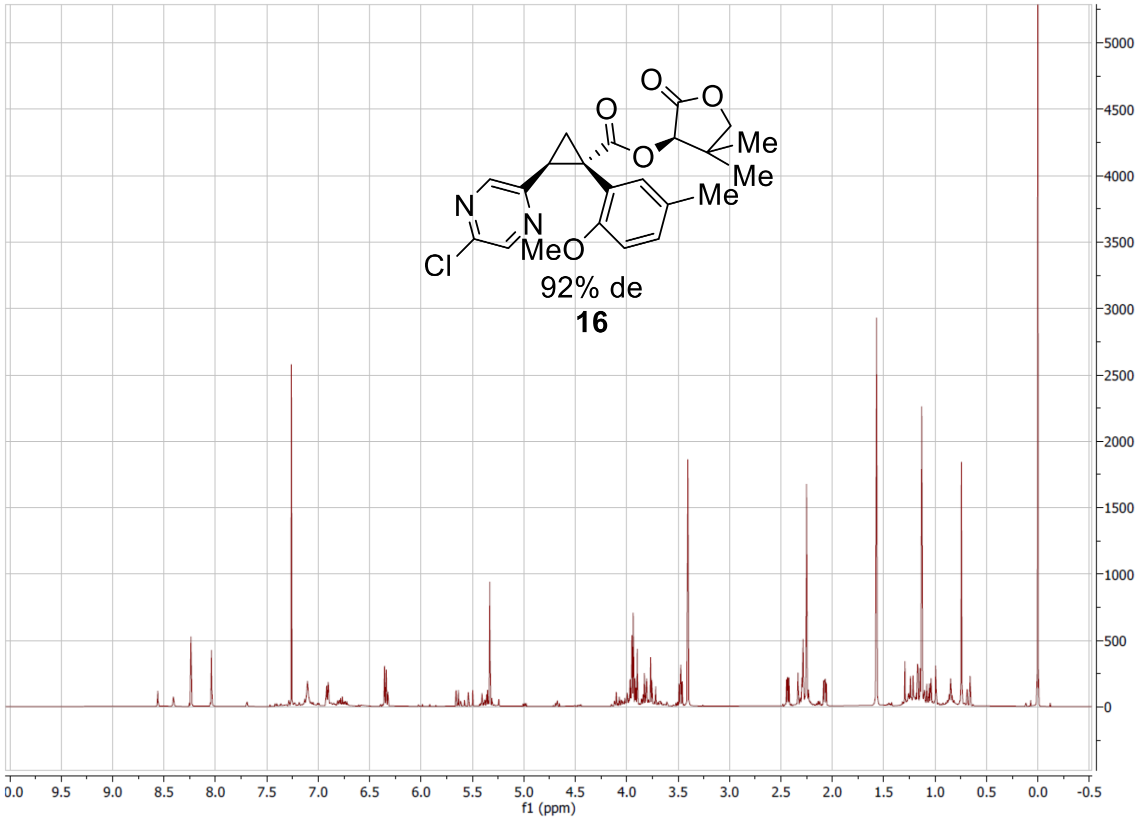

O
O
O
O
Me
Me
Me
N
N
Cl
MeO
92% de
16
f1 (ppm)

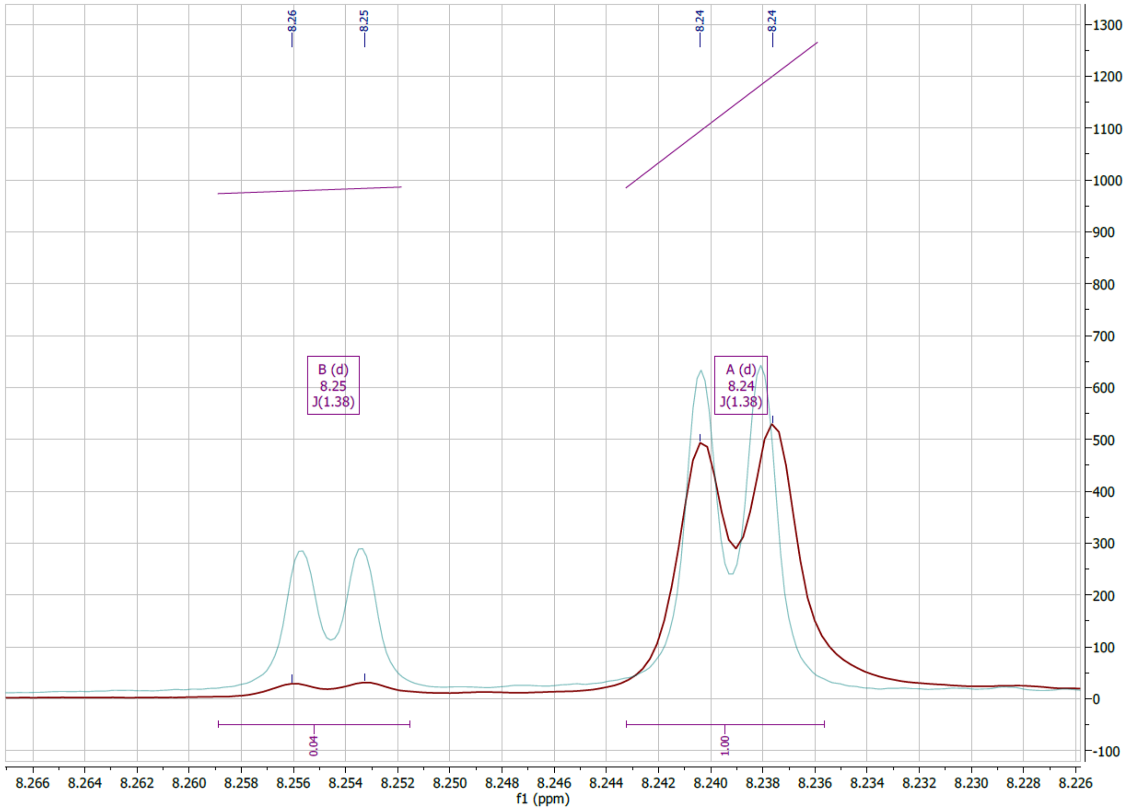

8.26
8.25
8.24
8.24
B (d)
8.25
J(1.38)
A (d)
8.24
J(1.38)
0.04
1.00
f1 (ppm)

5.2: Enantioselectivity of cyclopropanes synthesized with chiral catalysts was determined by chiral HPLC, UHPLC, of SFC.

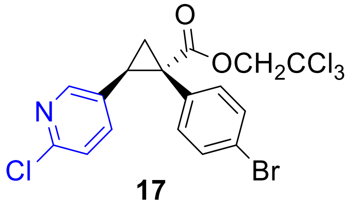

17

Racemic trace:

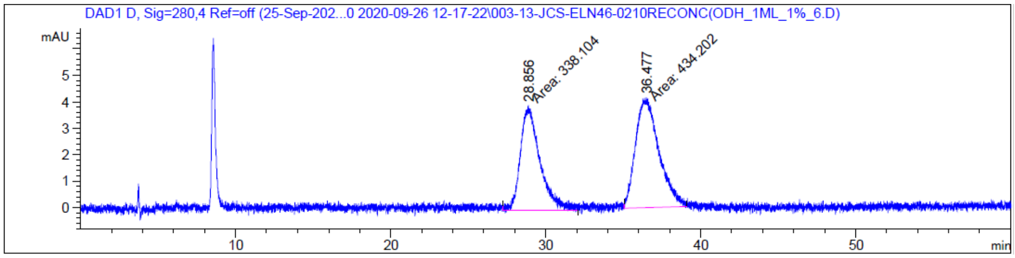

Signal 4: DAD1 D, Sig=280,4 Ref=off

Peak #	RetTime [min]	Type	Width [min]	Area [mAU*s]	Height [mAU]	Area %
1	28.856	MM	1.4272	338.10449	3.94839	43.7785
2	36.477	MM	1.7347	434.20224	4.17165	56.2215

Totals : 772.30673 8.12004

25 ℃, 0.5 mol % Rh_2(*R*-*p*-Ph-TPCP)$_4$, reaction performed according to procedure 3.2 with $(MeO)_2CO$ as solvent

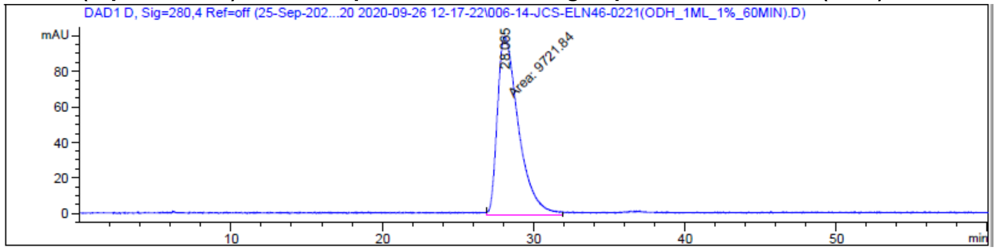

Signal 4: DAD1 D, Sig=280,4 Ref=off

Peak #	RetTime [min]	Type	Width [min]	Area [mAU*s]	Height [mAU]	Area %
1	28.065	MM	1.6141	9721.84082	100.38266	100.0000

Totals : 9721.84082 100.38266

25 ℃, 0.5 mol % Rh_2(*R*-*p*-Ph-TPCP)$_4$, reaction performed according to procedure 3.2 with CH_2Cl_2 as solvent

Chromatogram :
JCS-ELN46-0211(ODH_1ML_1%_60MIN)11_channel2

System : Prostar LC System
Method : ADH_30min_1mL_1%-230nm
User : User1

Acquired : 2/3/2020 5:14:08 PM
Processed : 2/4/2020 12:14:28 PM
Printed : 9/12/2020 12:01:47 PM

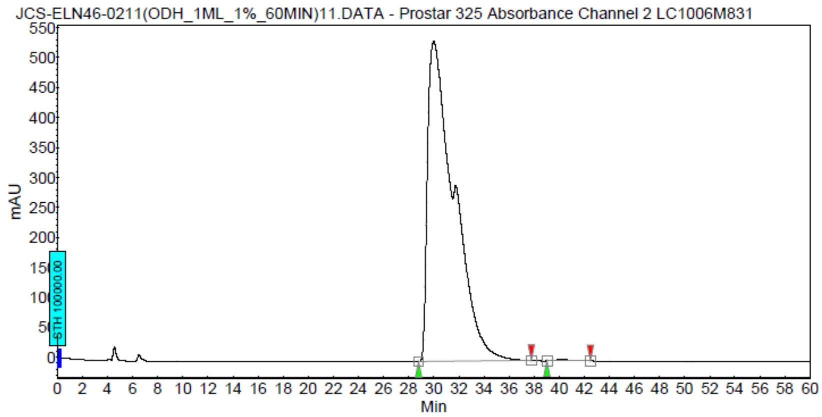

Peak results :

Index	Name	Time [Min]	Quantity [% Area]	Height [mAU]	Area [mAU.Min]	Area % [%]
1	UNKNOWN	29.99	99.72	533.4	1307.9	99.723
2	UNKNOWN	40.16	0.28	2.2	3.6	0.277
Total			100.00	535.6	1311.5	100.000

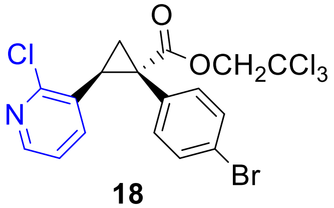

18

Racemic trace:

Chromatogram :
JCS-ELN46-0224(ADH_1ML_1%_60MIN)31_channel1

System : Prostar LC System
Method : DACHDNB_30min_1mL_1%-230nm
User : User1

Acquired : 2/7/2020 6:12:59 AM
Processed : 2/8/2020 11:36:01 AM
Printed : 9/12/2020 11:57:24 AM

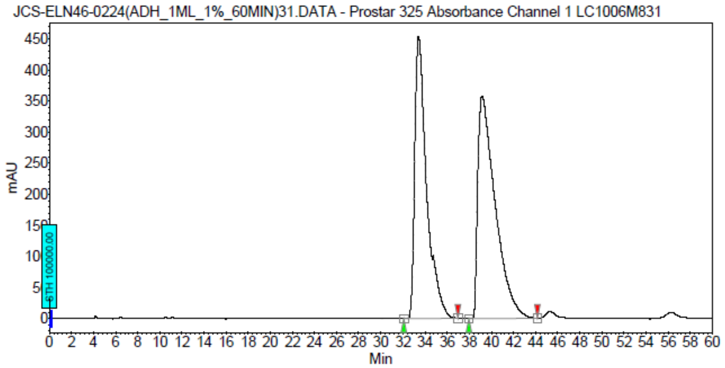

Peak results :

Index	Name	Time [Min]	Quantity [% Area]	Height [mAU]	Area [mAU.Min]	Area % [%]
1	UNKNOWN	33.41	46.30	453.4	566.0	46.298
2	UNKNOWN	39.16	53.70	358.0	656.4	53.702
Total			100.00	811.5	1222.4	100.000

25 ℃, 0.5 mol % Rh_2(*R-p*-Ph-TPCP)$_4$, reaction performed according to procedure 3.2

Chromatogram :
JCS-ELN46-0225(ADH_1ML_1%_60min)76_channel1

System : Prostar LC System
Method : DACHDNB_30min_1mL_1%-230nm
User : User1

Acquired : 2/8/2020 7:25:39 AM
Processed : 2/8/2020 11:36:31 AM
Printed : 9/12/2020 11:57:09 AM

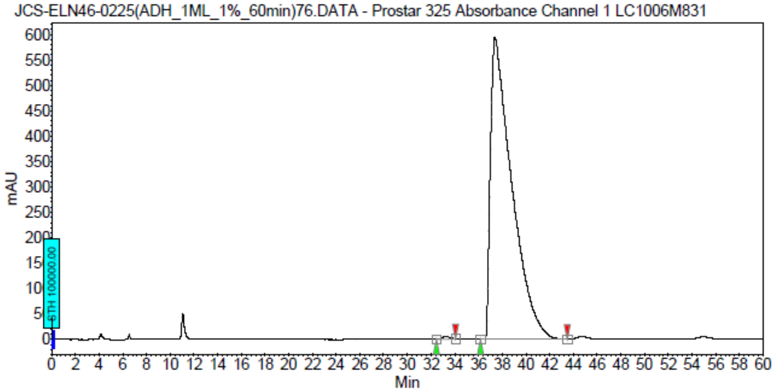

Peak results :

Index	Name	Time [Min]	Quantity [% Area]	Height [mAU]	Area [mAU.Min]	Area % [%]
1	UNKNOWN	33.25	0.31	5.4	3.9	0.314
2	UNKNOWN	37.38	99.69	594.2	1238.7	99.686
Total			100.00	599.6	1242.6	100.000

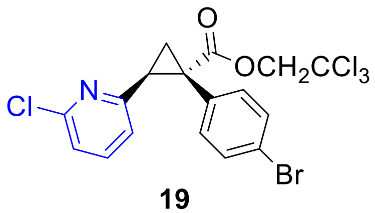

19

Racemic trace:

Chromatogram :
JCS-ELN46-0226(ADH_1ML_1%_60MIN)34_channel1

System : Prostar LC System
Method : DACHDNB_30min_1mL_1%-230nm
User : User1

Acquired : 2/7/2020 8:01:53 AM
Processed : 9/12/2020 11:56:08 AM
Printed : 9/12/2020 11:56:13 AM

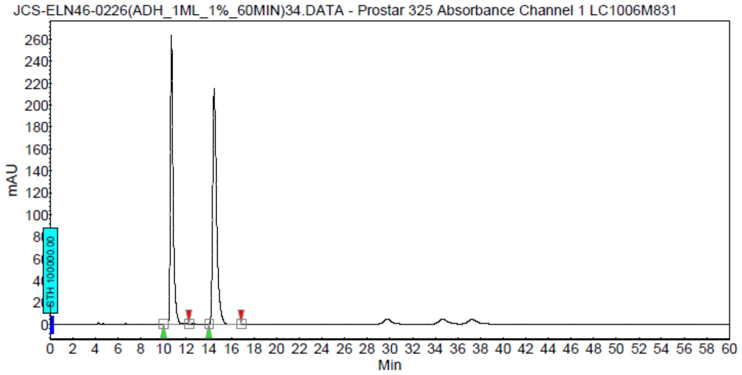

Peak results :

Index	Name	Time [Min]	Quantity [% Area]	Height [mAU]	Area [mAU.Min]	Area % [%]
1	UNKNOWN	10.71	47.51	263.2	75.0	47.514
2	UNKNOWN	14.49	52.49	214.9	82.9	52.486
Total			100.00	478.1	157.9	100.000

25 ℃, 0.5 mol % Rh_2(*R-p*-Ph-TPCP)$_4$, reaction performed according to procedure 3.2

Chromatogram :
JCS-ELN46-0227(ADH_1ML_1%_30min)79_chann el1

System : Prostar LC System
Method : DACHDNB_30min_1mL_1%-230nm
User : User1

Acquired : 2/8/2020 9:14:11 AM
Processed : 9/12/2020 11:56:41 AM
Printed : 9/12/2020 11:56:45 AM

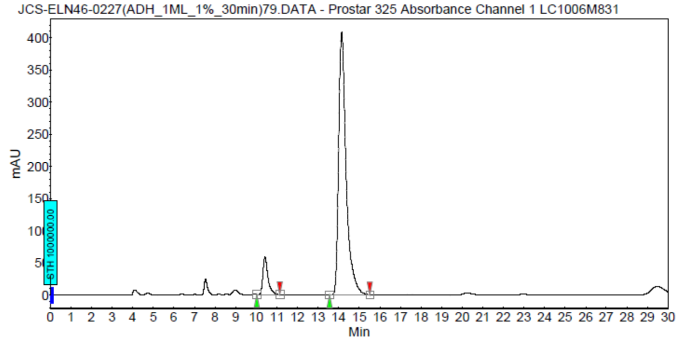

Peak results :

Index	Name	Time [Min]	Quantity [% Area]	Height [mAU]	Area [mAU.Min]	Area % [%]
2	UNKNOWN	10.42	8.92	58.8	16.7	8.916
1	UNKNOWN	14.14	91.08	408.1	170.8	91.084
Total			100.00	467.0	187.5	100.000

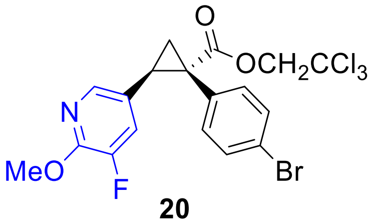

20

Racemic trace:

Chromatogram :
JCS-ELN46-0228(ODH_1ML_1%_60MIN)9_channe l1

System : Prostar LC System
Method : ADH_30min_1mL_1%-230nm
User : User1

Acquired : 2/6/2020 6:25:51 PM
Processed : 2/8/2020 11:39:19 AM
Printed : 9/12/2020 11:54:43 AM

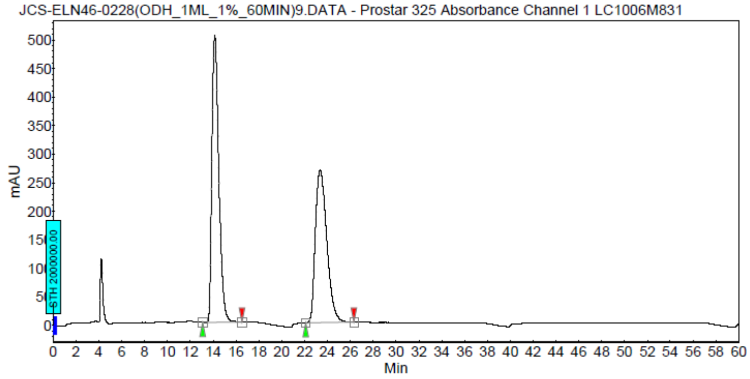

Peak results :

Index	Name	Time [Min]	Quantity [% Area]	Height [mAU]	Area [mAU.Min]	Area % [%]
1	UNKNOWN	14.14	53.42	502.8	357.7	53.415
2	UNKNOWN	23.37	46.58	268.3	312.0	46.585
Total			100.00	771.1	669.7	100.000

25 ℃, 0.5 mol % Rh_2(*R*-*p*-Ph-TPCP)$_4$, reaction performed according to procedure 3.2

Chromatogram :
JCS-ELN46-0229(ODH_1ML_30MIN_1%)54_chann
el2

System : Prostar LC System
Method : ADH_30min_1mL_1%-230nm
User : User1

Acquired : 2/7/2020 5:29:57 PM
Processed : 2/8/2020 11:40:03 AM
Printed : 9/12/2020 11:54:21 AM

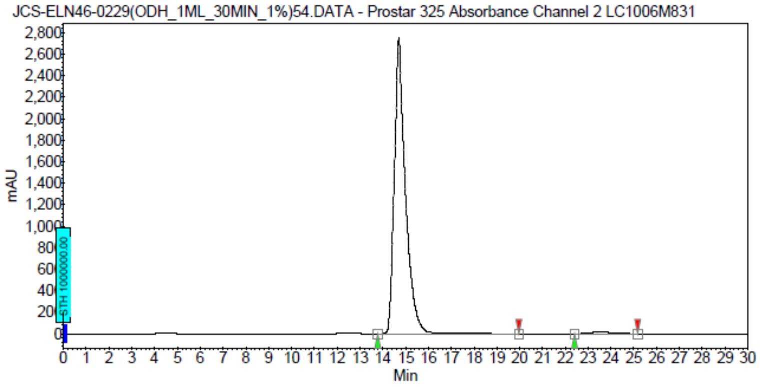

Peak results :

Index	Name	Time [Min]	Quantity [% Area]	Height [mAU]	Area [mAU.Min]	Area % [%]
2	UNKNOWN	14.70	99.01	2751.0	1541.2	99.011
1	UNKNOWN	23.54	0.99	15.0	15.4	0.989
Total			100.00	2765.9	1556.6	100.000

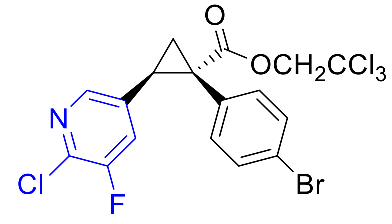

21

Racemic trace:

Chromatogram :
JCS-ELN46-0230(ODH_1ML_1%_60MIN)12_chann
el1

System : Prostar LC System
Method : ADH_30min_1mL_1%-230nm
User : User1

Acquired : 2/6/2020 8:14:14 PM
Processed : 2/8/2020 11:38:02 AM
Printed : 9/12/2020 11:54:07 AM

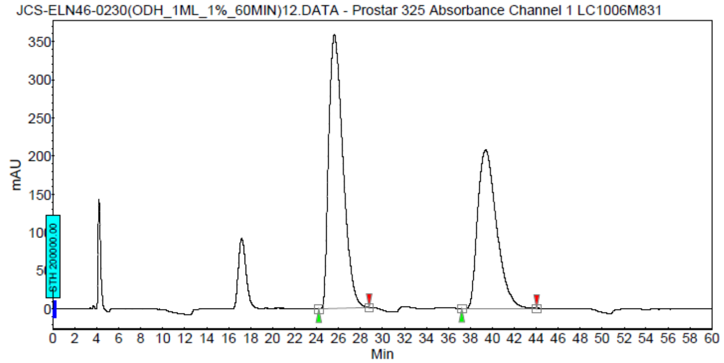

Peak results :

Index	Name	Time [Min]	Quantity [% Area]	Height [mAU]	Area [mAU.Min]	Area % [%]
1	UNKNOWN	25.63	56.49	357.9	537.0	56.492
2	UNKNOWN	39.39	43.51	207.4	413.6	43.508
Total			100.00	565.3	950.6	100.000

25 ℃, 0.5 mol % Rh_2(*R*-*p*-Ph-TPCP)$_4$, reaction performed according to procedure 3.2

Chromatogram :
JCS-ELN46-0231(ODH_1ML_60MIN_1%)57_chann
el2

System : Prostar LC System
Method : ADH_30min_1mL_1%-230nm
User : User1

Acquired : 2/7/2020 6:47:17 PM
Processed : 2/8/2020 11:38:42 AM
Printed : 9/12/2020 11:53:49 AM

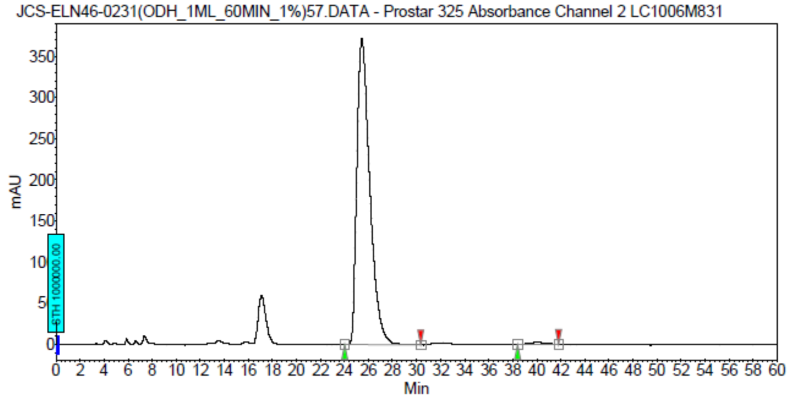

Peak results :

Index	Name	Time [Min]	Quantity [% Area]	Height [mAU]	Area [mAU.Min]	Area % [%]
2	UNKNOWN	25.44	99.08	371.7	464.2	99.076
1	UNKNOWN	40.06	0.92	2.8	4.3	0.924
Total			100.00	374.5	468.5	100.000

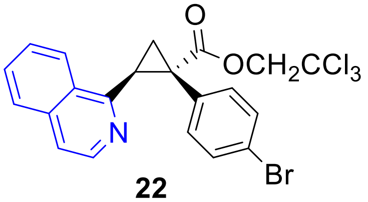

22

Racemic trace:

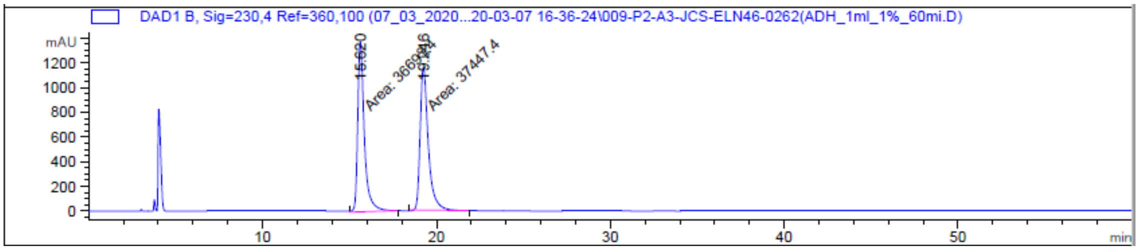


```
Signal 2: DAD1 B, Sig=230,4 Ref=360,100

Peak RetTime Type  Width     Area      Height     Area
  #   [min]        [min]   [mAU*s]     [mAU]        %
----|-------|----|-------|----------|----------|--------|
   1  15.620 MM    0.4448 3.66934e4  1375.04175  49.4915
   2  19.246 MM    0.5396 3.74474e4  1156.70520  50.5085

Totals :                  7.41407e4  2531.74695
```

25 ℃, 0.5 mol % Rh_2(*R*-*p*-Ph-TPCP)$_4$, reaction performed according to procedure 3.2

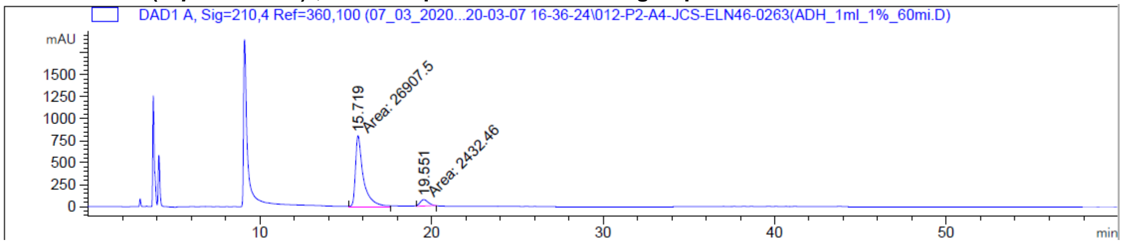

```
Signal 1: DAD1 A, Sig=210,4 Ref=360,100

Peak RetTime Type  Width     Area      Height     Area
  #   [min]        [min]   [mAU*s]     [mAU]      %
----|-------|----|-------|----------|----------|--------|
   1  15.719 MM    0.5516 2.69075e4  812.98846  91.7094
   2  19.551 MM    0.5503 2432.45801   73.66758   8.2906

Totals :                  2.93400e4  886.65604
```

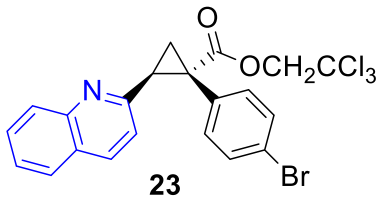

23

Racemic trace:

Chromatogram :
JCS-ELN46-0258(ADH_1ML_1%_90MIN)36_chann
el2
System : Prostar LC System
Method : OJ_30min_1mL_1%_230NM
User : User1
Acquired : 3/5/2020 4:42:38 PM
Processed : 9/12/2020 9:53:46 AM
Printed : 9/12/2020 9:54:03 AM

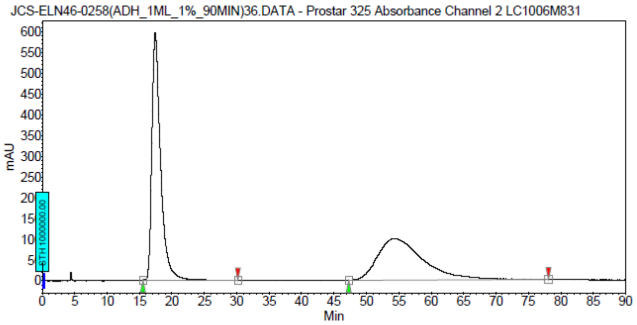

Peak results :

Index	Name	Time [Min]	Quantity [% Area]	Height [mAU]	Area [mAU.Min]	Area % [%]
1	UNKNOWN	17.42	52.95	595.3	927.8	52.950
2	UNKNOWN	54.21	47.05	99.8	824.4	47.050
Total			100.00	695.1	1752.3	100.000

25 ℃, 0.5 mol % Rh_2(*R*-*p*-Ph-TPCP)$_4$, reaction performed according to procedure 3.2

Chromatogram :
JCS-ELN46-0259RE(ADH_1ML_1%_90MIN)19_ch
annel2
System : Prostar LC System
Method : OJ_30min_1mL_1%_230NM
User : User1
Acquired : 3/10/2020 6:10:24 PM
Processed : 3/11/2020 10:29:05 AM
Printed : 9/12/2020 9:50:41 AM

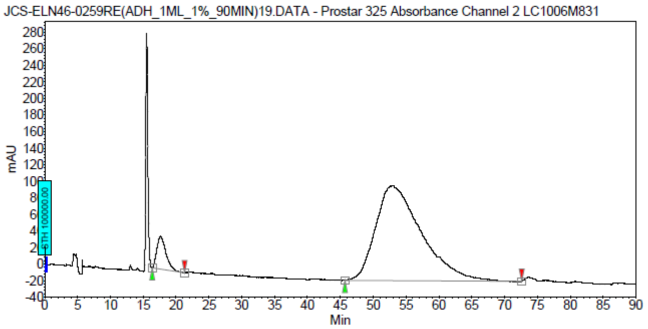

Peak results :

Index	Name	Time [Min]	Quantity [% Area]	Height [mAU]	Area [mAU.Min]	Area % [%]
2	UNKNOWN	17.62	6.50	40.0	64.3	6.502
1	UNKNOWN	52.99	93.50	114.5	925.0	93.498
Total			100.00	154.5	989.3	100.000

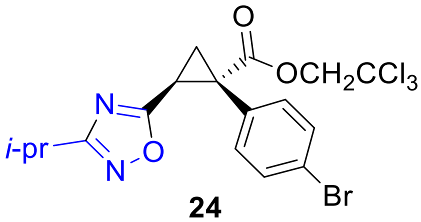

24

Racemic trace:

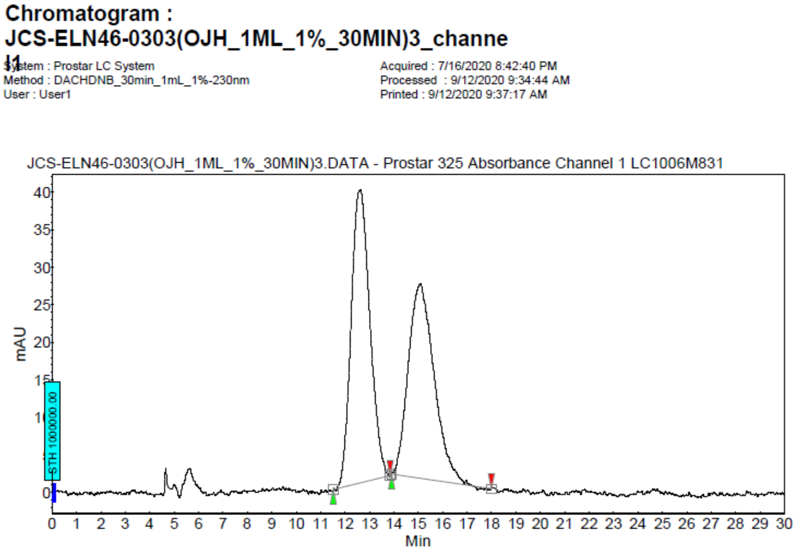

Peak results :

Index	Name	Time [Min]	Quantity [% Area]	Height [mAU]	Area [mAU.Min]	Area % [%]
1	UNKNOWN	12.63	52.26	38.9	34.5	52.261
2	UNKNOWN	15.08	47.74	25.9	31.6	47.739
Total			100.00	64.9	66.1	100.000

25 ℃, 0.5 mol % Rh_2(*R*-*p*-Ph-TPCP)$_4$, reaction performed according to procedure 3.2

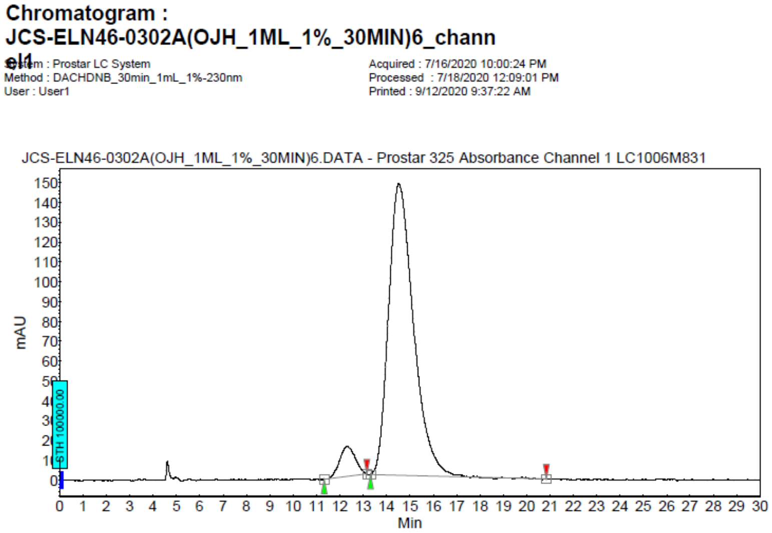

Peak results :

Index	Name	Time [Min]	Quantity [% Area]	Height [mAU]	Area [mAU.Min]	Area % [%]
1	UNKNOWN	12.33	5.99	15.0	11.6	5.990
2	UNKNOWN	14.52	94.01	146.9	182.2	94.010
Total			100.00	161.9	193.9	100.000

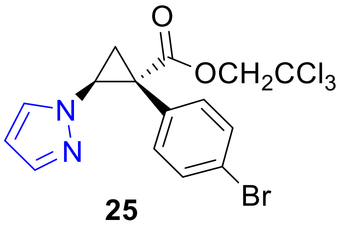

25

Racemic trace:

Chromatogram :
JCS-ELN46-0309(OJH_45min_1ml_1%)3_channel1

System : Prostar LC System
Method : DACHDNB_30min_1mL_1%-230nm
User : User1

Acquired : 7/30/2020 1:57:01 PM
Processed : 7/30/2020 5:45:01 PM
Printed : 9/12/2020 9:31:34 AM

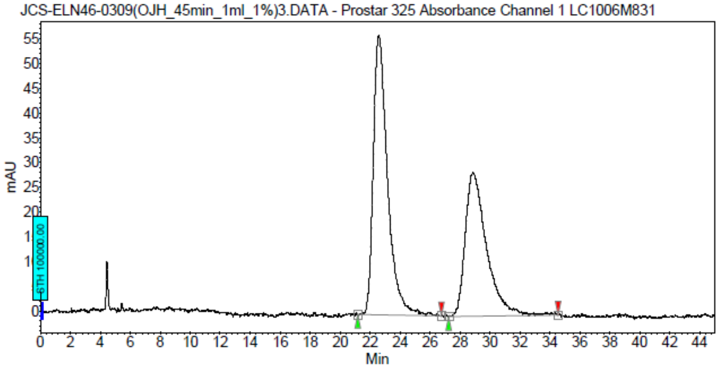

Peak results :

Index	Name	Time [Min]	Quantity [% Area]	Height [mAU]	Area [mAU.Min]	Area % [%]
1	UNKNOWN	22.60	56.44	56.5	62.2	56.439
2	UNKNOWN	28.87	43.56	29.1	48.0	43.561
Total			100.00	85.5	110.1	100.000

25 ℃, 1 mol % Rh$_2$(*R*-*p*-Ph-TPCP)$_4$, reaction performed according to procedure 3.2

Chromatogram :
JCS-ELN46-0310BRE(OJH_45min_1ml_1%)6_channel1

System : Prostar LC System
Method : DACHDNB_30min_1mL_1%-230nm
User : User1

Acquired : 8/1/2020 2:55:50 PM
Processed : 8/3/2020 1:18:21 PM
Printed : 9/12/2020 9:31:01 AM

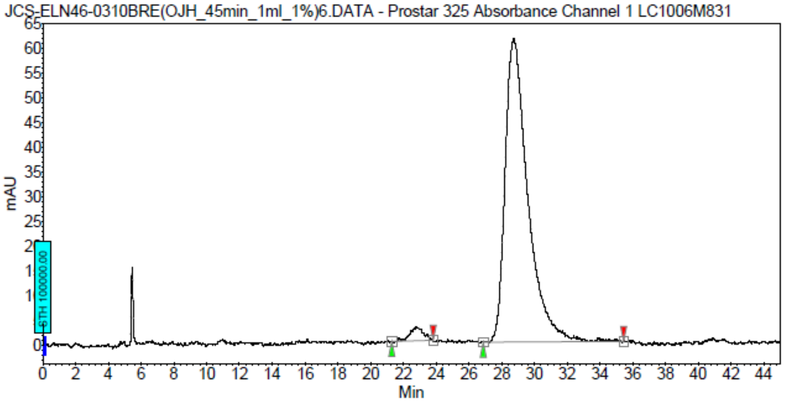

Peak results :

Index	Name	Time [Min]	Quantity [% Area]	Height [mAU]	Area [mAU.Min]	Area % [%]
1	UNKNOWN	22.84	2.62	2.8	2.7	2.622
2	UNKNOWN	28.74	97.38	61.3	98.5	97.378
Total			100.00	64.1	101.1	100.000

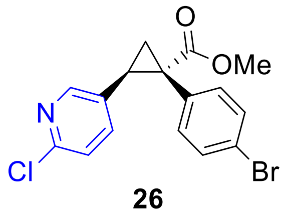

26

Racemic trace:

Chromatogram :
Bo-ELN36-0104(ADH_1ML_1%_60MIN)8_channel1

System : Prostar LC System
Method : DACHDNB_30min_1mL_1%-230nm
User : User1

Acquired : 2/4/2020 1:08:05 PM
Processed : 9/21/2020 6:17:22 PM
Printed : 9/21/2020 6:17:25 PM

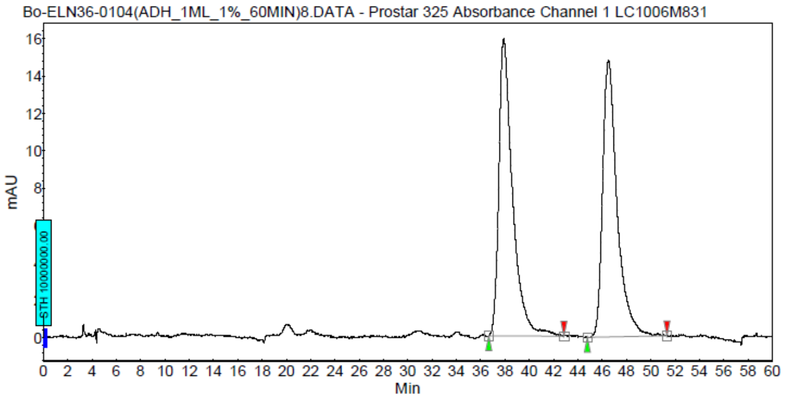

Peak results :

Index	Name	Time [Min]	Quantity [% Area]	Height [mAU]	Area [mAU.Min]	Area % [%]
1	UNKNOWN	37.89	50.44	15.9	20.8	50.437
2	UNKNOWN	46.52	49.56	14.8	20.5	49.563
Total			100.00	30.7	41.3	100.000

25 ℃, 0.5 mol % Rh_2(*R-p*-Ph-TPCP)$_4$, reaction performed according to procedure 3.2

Chromatogram :
JCS-ELN46-0215(ADH_1ML_1%_60MIN)10_chann el1

System : Prostar LC System
Method : DACHDNB_30min_1mL_1%-230nm
User : User1

Acquired : 2/4/2020 2:56:34 PM
Processed : 2/5/2020 9:28:29 AM
Printed : 9/12/2020 11:59:59 AM

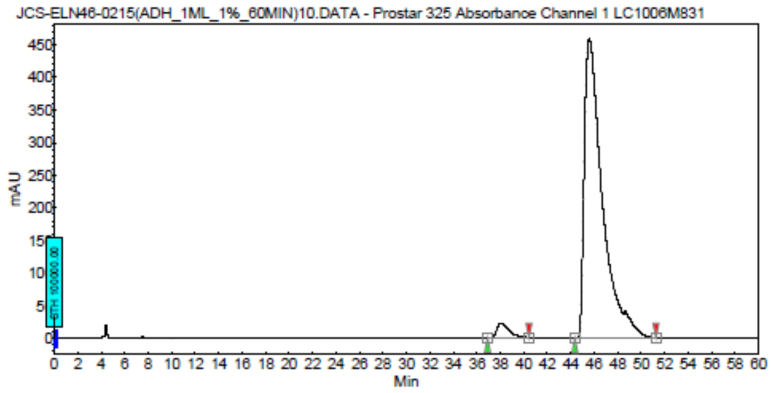

Peak results :

Index	Name	Time [Min]	Quantity [% Area]	Height [mAU]	Area [mAU.Min]	Area % [%]
1	UNKNOWN	38.06	3.20	22.9	27.0	3.197
2	UNKNOWN	45.59	96.80	458.6	818.0	96.803
Total			100.00	481.5	845.0	100.000

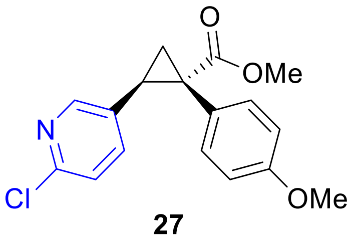

27

Racemic trace:

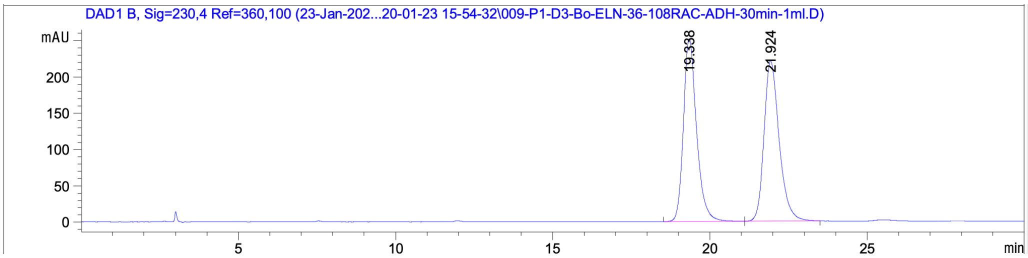

Signal 2: DAD1 B, Sig=230,4 Ref=360,100

Peak #	RetTime [min]	Type	Width [min]	Area [mAU*s]	Height [mAU]	Area %
1	19.338	BB	0.4391	7316.83301	251.73724	49.7532
2	21.924	BB	0.5079	7389.43115	221.10867	50.2468

25 ℃, 0.5 mol % Rh_2(*R*-*p*-Ph-TPCP)$_4$, reaction performed according to procedure 3.2

Chromatogram :
JCS-ELN46-0232(ADH_1ML_3%_30MIN)109_channel2

System : Prostar LC System
Method : DACHDNB_30min_1mL_3%-230nm
User : User1

Acquired : 2/8/2020 6:44:06 PM
Processed : 2/10/2020 8:45:18 AM
Printed : 9/12/2020 11:53:36 AM

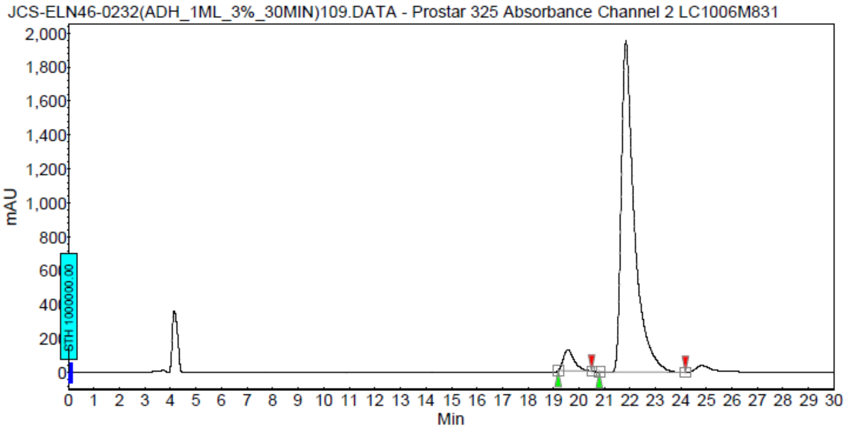

Peak results :

Index	Name	Time [Min]	Quantity [% Area]	Height [mAU]	Area [mAU.Min]	Area % [%]
1	UNKNOWN	19.57	5.01	123.8	61.0	5.010
2	UNKNOWN	21.84	94.99	1953.7	1156.0	94.990
Total			100.00	2077.5	1216.9	100.000

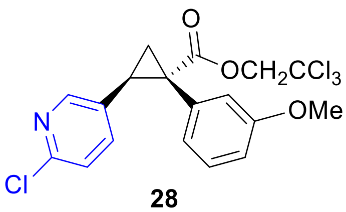

28

Racemic trace:

Chromatogram :
BO-ELN36-0102(ADH_1ML_5%_45min)89_channe l1

System : Prostar LC System
Method : DACHDNB_30min_1mL_5%_230nm
User : User1

Acquired : 2/8/2020 2:20:55 PM
Processed : 9/25/2020 4:50:39 PM
Printed : 9/25/2020 4:50:46 PM

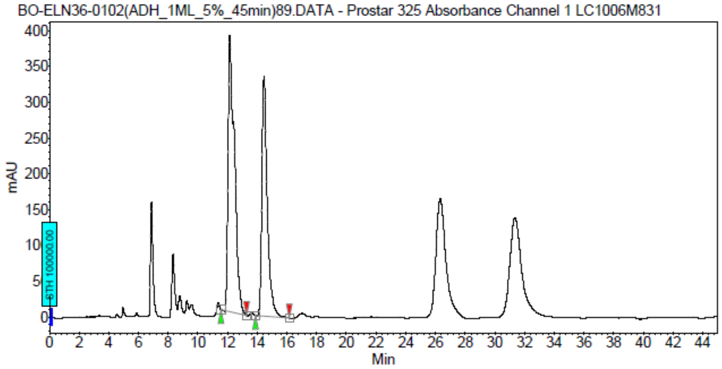

Peak results :

Index	Name	Time [Min]	Quantity [% Area]	Height [mAU]	Area [mAU.Min]	Area % [%]
1	UNKNOWN	12.15	57.51	385.2	192.0	57.512
2	UNKNOWN	14.45	42.49	334.2	141.9	42.488
Total			100.00	719.4	333.9	100.000

25 ℃, 0.5 mol % Rh_2(*R*-*p*-Ph-TPCP)$_4$, reaction performed according to procedure 3.2

Chromatogram :
JCS-ELN46-0233(ADH_1ML_5%_45min)92_chann el2

System : Prostar LC System
Method : DACHDNB_30min_1mL_5%_230nm
User : User1

Acquired : 2/8/2020 3:53:38 PM
Processed : 2/10/2020 8:43:26 AM
Printed : 9/12/2020 11:53:20 AM

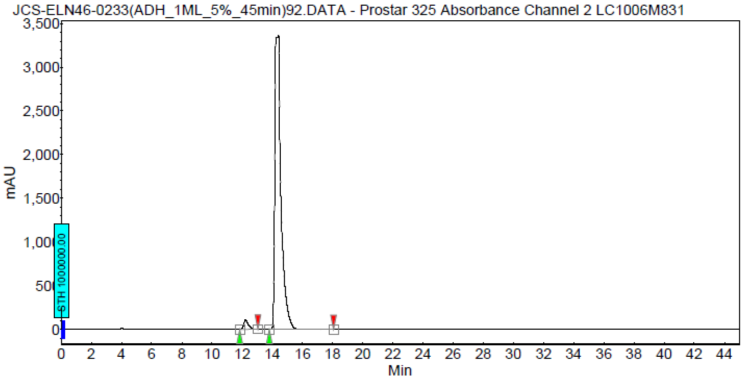

Peak results :

Index	Name	Time [Min]	Quantity [% Area]	Height [mAU]	Area [mAU.Min]	Area % [%]
1	UNKNOWN	12.23	2.40	105.1	39.8	2.404
2	UNKNOWN	14.43	97.60	3358.7	1616.3	97.596
Total			100.00	3463.9	1656.1	100.000

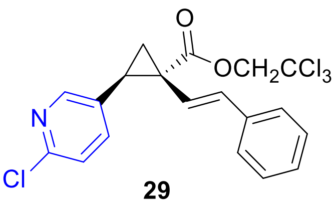

29

Racemic trace:

Chromatogram :
JCS-ELN46-0242A(ADH_1ML_3%_60MIN)15_channel2

System : Prostar LC System
Method : ADH_30min_1mL_3%-230nm
User : User1

Acquired : 2/13/2020 12:04:37 AM
Processed : 9/12/2020 11:49:31 AM
Printed : 9/12/2020 11:49:36 AM

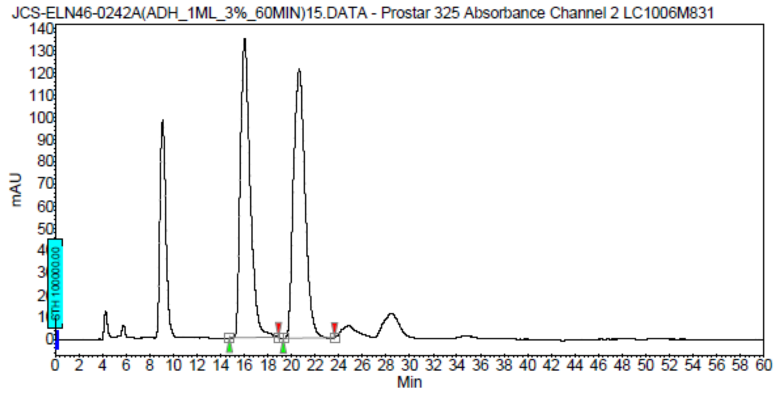

Peak results :

Index	Name	Time [Min]	Quantity [% Area]	Height [mAU]	Area [mAU.Min]	Area % [%]
1	UNKNOWN	16.04	48.67	134.3	129.6	48.668
2	UNKNOWN	20.66	51.33	120.9	136.7	51.332
Total			100.00	255.2	266.4	100.000

25 ℃, 0.5 mol % Rh_2(*R*-*p*-Ph-TPCP)$_4$, reaction performed according to procedure 3.2

Chromatogram :
JCS-ELN46-0243(ODH_1ML_3%_30MIN)18_channel1

System : Prostar LC System
Method : ADH_30min_1mL_3%-230nm
User : User1

Acquired : 2/13/2020 9:21:41 PM
Processed : 2/14/2020 12:33:29 PM
Printed : 9/12/2020 11:47:01 AM

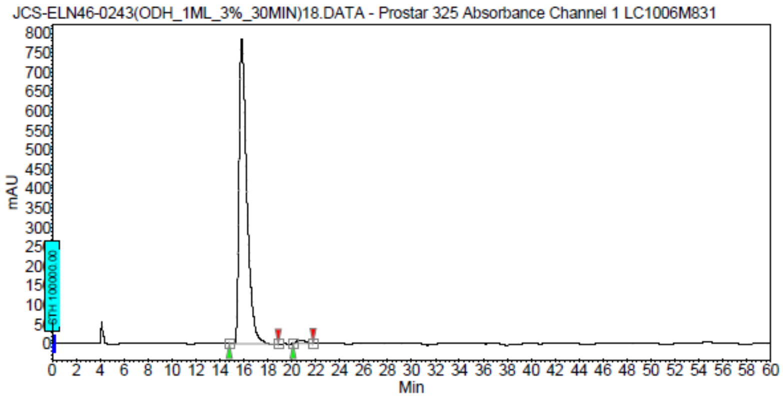

Peak results :

Index	Name	Time [Min]	Quantity [% Area]	Height [mAU]	Area [mAU.Min]	Area % [%]
1	UNKNOWN	15.83	98.96	784.2	595.5	98.955
2	UNKNOWN	20.74	1.04	8.3	6.3	1.045
Total			100.00	792.5	601.8	100.000

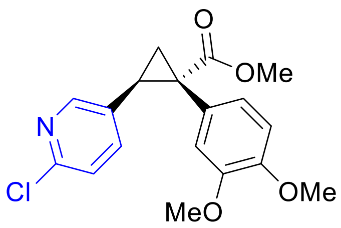

30

Racemic trace:

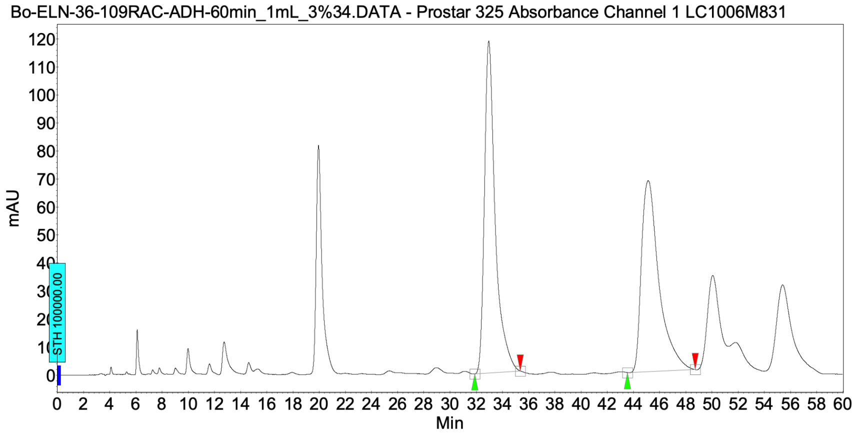

Peak results :

Index	Name	Time [Min]	Quantity [% Area]	Height [mAU]	Area [mAU.Min]	Area % [%]
1	UNKNOWN	32.96	51.67	118.6	113.3	51.674
2	UNKNOWN	45.12	48.33	68.2	106.0	48.326
Total			100.00	186.8	219.3	100.000

25 ℃, 0.5 mol % Rh_2(*R*-*p*-Ph-TPCP)$_4$, reaction performed according to procedure 3.2

Chromatogram :
JCS-ELN46-0245RE(ADH_1ML_3%_60MIN)3_channel1

System : Prostar LC System
Method : DACHDNB_30min_1mL_3%-230nm
User : User1

Acquired : 2/19/2020 11:11:37 AM
Processed : 2/19/2020 12:19:49 PM
Printed : 9/12/2020 11:43:05 AM

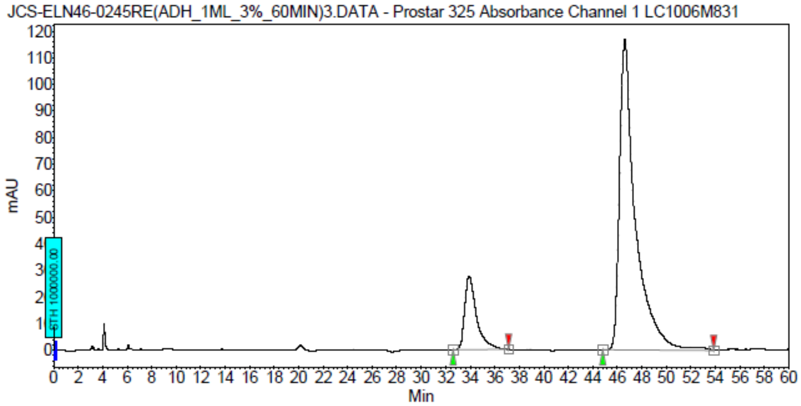

Peak results :

Index	Name	Time [Min]	Quantity [% Area]	Height [mAU]	Area [mAU.Min]	Area % [%]
1	UNKNOWN	33.90	14.93	27.4	30.8	14.930
2	UNKNOWN	46.61	85.07	116.9	175.5	85.070
Total			100.00	144.3	206.3	100.000

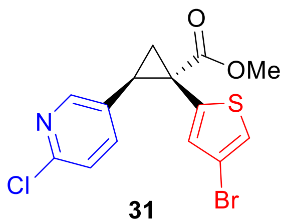

31

Racemic trace:

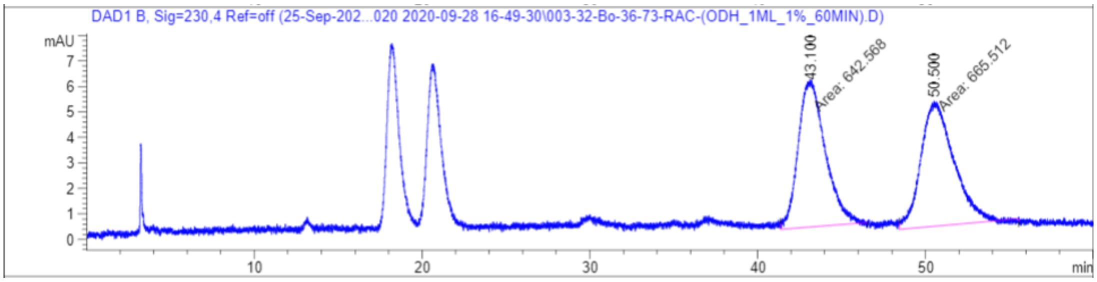

Signal 2: DAD1 B, Sig=230,4 Ref=off

Peak #	RetTime [min]	Type	Width [min]	Area [mAU*s]	Height [mAU]	Area %
1	43.100	MM	1.8564	642.56848	5.76899	49.1230
2	50.500	MM	2.2602	665.51190	4.90745	50.8770

Totals : 1308.08038 10.67644

25 ℃, 0.5 mol % Rh_2(*R*-*p*-Ph-TPCP)$_4$, reaction performed according to procedure 3.2

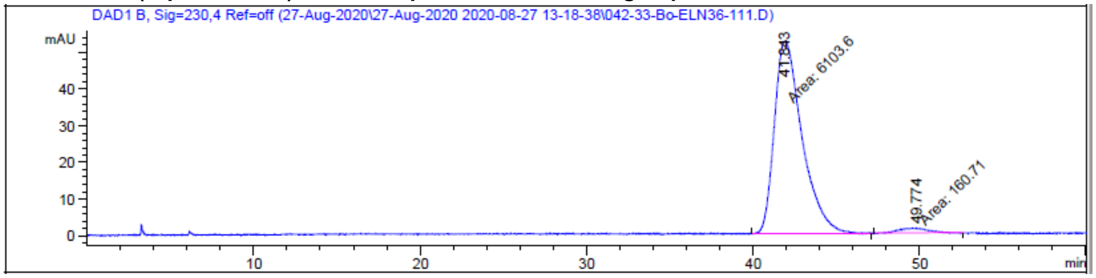

Signal 2: DAD1 B, Sig=230,4 Ref=off

Peak #	RetTime [min]	Type	Width [min]	Area [mAU*s]	Height [mAU]	Area %
1	41.843	MM	1.9352	6103.60498	52.56713	97.4345
2	49.774	MM	1.9571	160.70956	1.36859	2.5655

Totals : 6264.31454 53.93572

Signal 3: DAD1 C, Sig=254,4 Ref=off

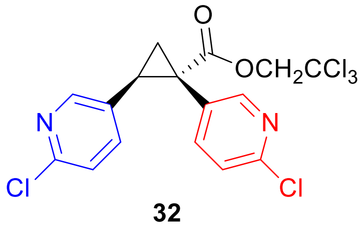

32

Racemic trace:

Chromatogram : JCS-ELN46-0234RACFINAL(ADH_1ML_7%_60MIN)6_channel1

System : [illegible] System
Method : DACHDNB_30min_1mL_7%
User : User1

Acquired : 2/19/2020 6:16:26 PM
Processed : 9/12/2020 11:43:33 AM
Printed : 9/12/2020 11:43:42 AM

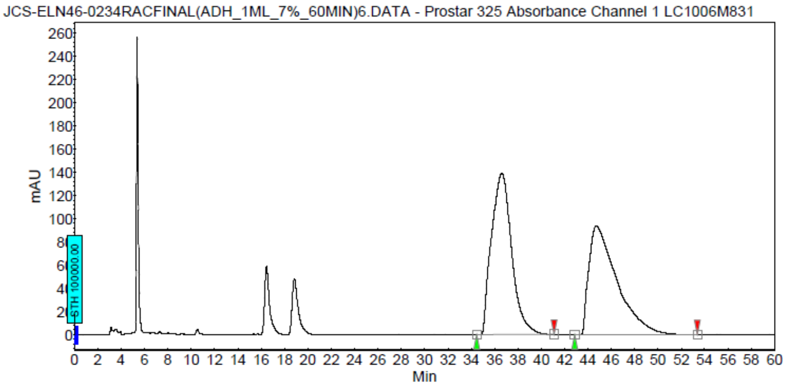

Peak results :

Index	Name	Time [Min]	Quantity [% Area]	Height [mAU]	Area [mAU.Min]	Area % [%]
1	UNKNOWN	36.61	51.67	138.8	278.8	51.672
2	UNKNOWN	44.71	48.33	93.6	260.8	48.328
Total			100.00	232.4	539.6	100.000

25 ℃, 0.5 mol % Rh_2(*R*-*p*-Ph-TPCP)$_4$, reaction performed according to procedure 3.2

Chromatogram : JCS-ELN46-0234RE(ADH_1ML_7%_60MIN)3_channel1

System : Prostar LC System
Method : DACHDNB_30min_1mL_7%
User : User1

Acquired : 2/20/2020 9:32:24 AM
Processed : 2/20/2020 11:01:57 AM
Printed : 9/12/2020 12:08:20 PM

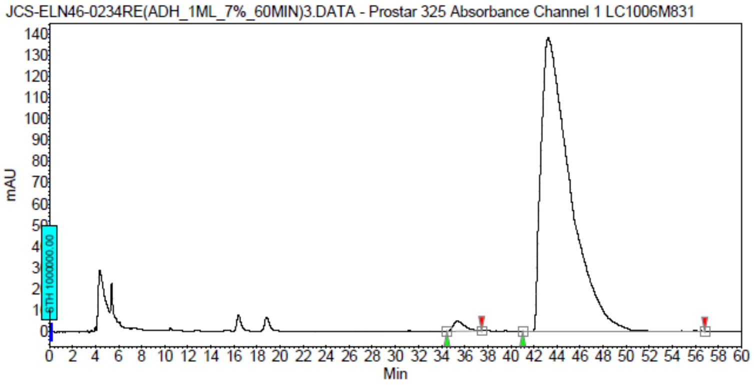

Peak results :

Index	Name	Time [Min]	Quantity [% Area]	Height [mAU]	Area [mAU.Min]	Area % [%]
1	UNKNOWN	35.37	1.42	4.8	5.8	1.415
2	UNKNOWN	43.25	98.58	138.1	402.4	98.585
Total			100.00	142.9	408.1	100.000

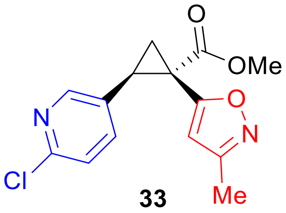

33

Racemic trace:

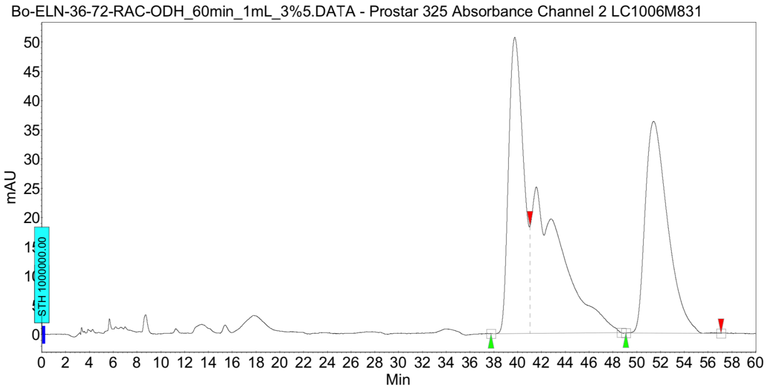

Peak results :

Index	Name	Time [Min]	Quantity [% Area]	Height [mAU]	Area [mAU.Min]	Area % [%]
2	UNKNOWN	39.76	47.92	50.7	73.0	47.918
1	UNKNOWN	51.41	52.08	36.2	79.4	52.082
Total			100.00	86.9	152.4	100.000

25 ℃, 0.5 mol % Rh_2(*R*-*p*-Ph-TPCP)$_4$, reaction performed according to procedure 3.2

Chromatogram : JCS-ELN46-0235RE(ODH_1ML_3%_60MIN)30_channel2

System : Prostar LC System
Method : ADH_30min_1mL_3%-230nm
User : User1

Acquired : 2/14/2020 4:35:50 AM
Processed : 2/14/2020 12:36:05 PM
Printed : 9/12/2020 11:46:24 AM

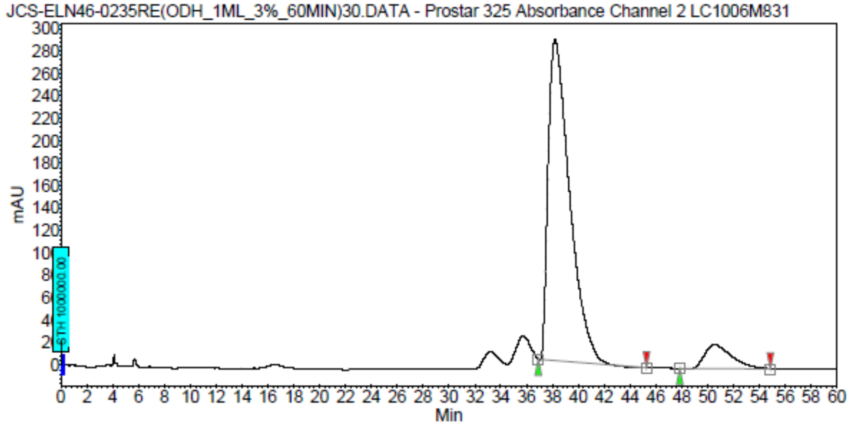

Peak results :

Index	Name	Time [Min]	Quantity [% Area]	Height [mAU]	Area [mAU.Min]	Area % [%]
1	UNKNOWN	38.21	91.41	285.8	547.1	91.409
2	UNKNOWN	50.56	8.59	21.1	51.4	8.591
Total			100.00	306.9	598.6	100.000

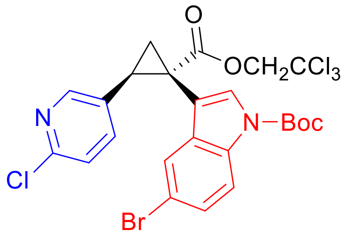

34

Racemic trace:

Chromatogram :
JCS-ELN46-0238A(ADH_1ML_3%_60MIN)3_chan nel1

System : Prostar LC System
Method : ADH_30min_1mL_3%-230nm
User : User1

Acquired : 2/12/2020 4:50:11 PM
Processed : 9/12/2020 11:51:40 AM
Printed : 9/12/2020 11:51:45 AM

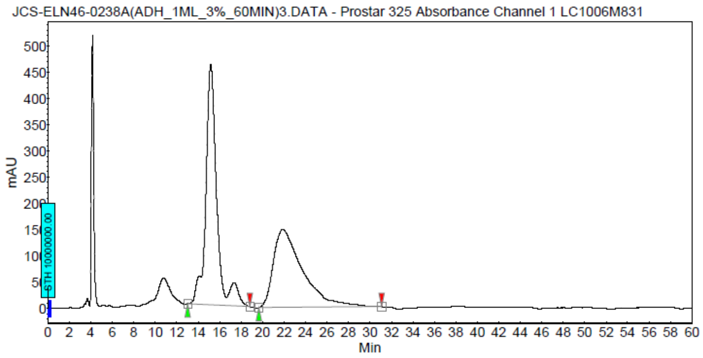

Peak results :

Index	Name	Time [Min]	Quantity [% Area]	Height [mAU]	Area [mAU.Min]	Area % [%]
1	UNKNOWN	15.17	53.91	457.7	547.2	53.912
2	UNKNOWN	21.87	46.09	147.8	467.8	46.088
Total			100.00	605.5	1015.0	100.000

25 ℃, 0.5 mol % Rh_2(*R*-*p*-Ph-TPCP)$_4$, reaction performed according to procedure 3.2

Chromatogram :
JCS-ELN46-0239(ODH_1ML_3%_30MIN)9_channe l2

System : Prostar LC System
Method : ADH_30min_1mL_3%-230nm
User : User1

Acquired : 2/13/2020 4:27:23 PM
Processed : 2/13/2020 5:37:57 PM
Printed : 9/12/2020 11:47:49 AM

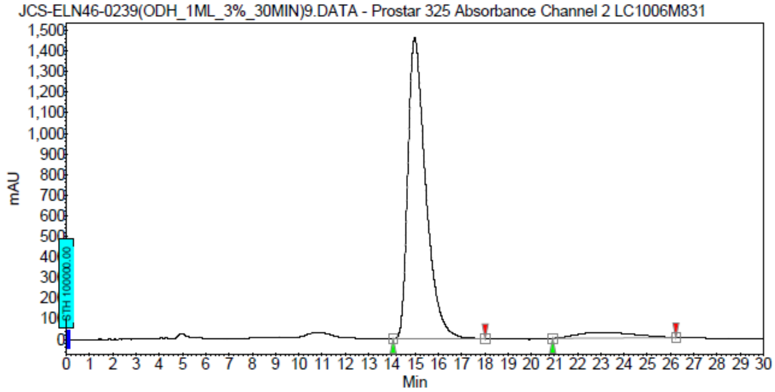

Peak results :

Index	Name	Time [Min]	Quantity [% Area]	Height [mAU]	Area [mAU.Min]	Area % [%]
1	UNKNOWN	14.99	94.21	1460.7	1300.2	94.213
2	UNKNOWN	23.00	5.79	27.2	79.9	5.787
Total			100.00	1487.9	1380.0	100.000

Racemic trace:

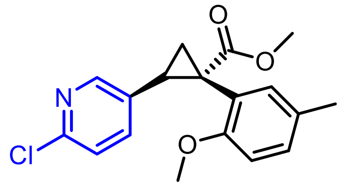

36

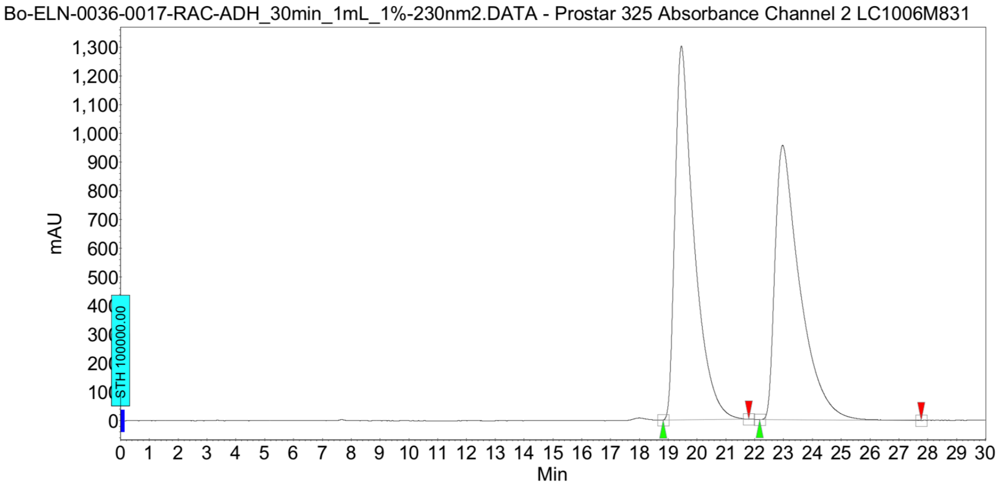

Peak results :

Index	Name	Time [Min]	Quantity [% Area]	Height [mAU]	Area [mAU.Min]	Area % [%]
2	UNKNOWN	19.46	51.18	1301.1	997.7	51.179
1	UNKNOWN	22.97	48.82	954.5	951.7	48.821
Total			100.00	2255.5	1949.4	100.000

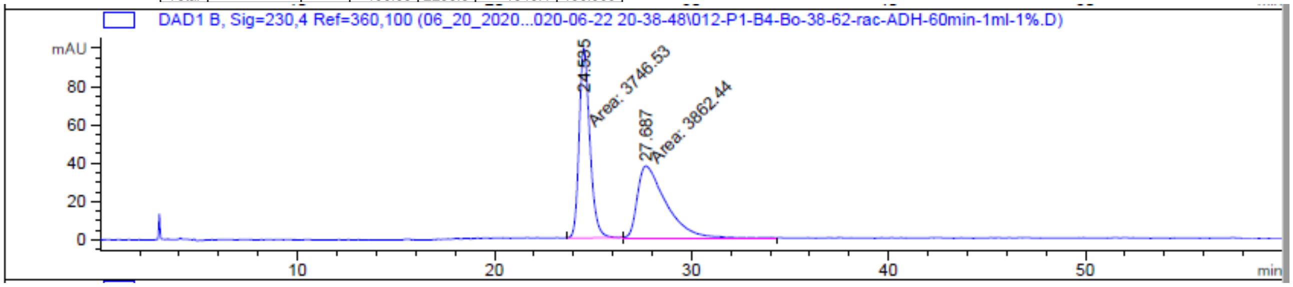


```
Signal 2: DAD1 B, Sig=230,4 Ref=360,100

Peak RetTime Type  Width     Area      Height     Area
  #   [min]        [min]   [mAU*s]     [mAU]        %
----|-------|----|-------|----------|----------|--------|
   1  24.535 MM    0.6308 3746.52588   98.99397  49.2383
   2  27.687 MM    1.7056 3862.44385   37.74244  50.7617

Totals :                  7608.96973  136.73640
```

25 ℃, 1 mol % Rh_2(*S*-Tris(*p*-^{t}BuPh)-TPCP)$_4$, 2.5 equiv of vinyl pyridine, reaction performed according to procedure 3.3

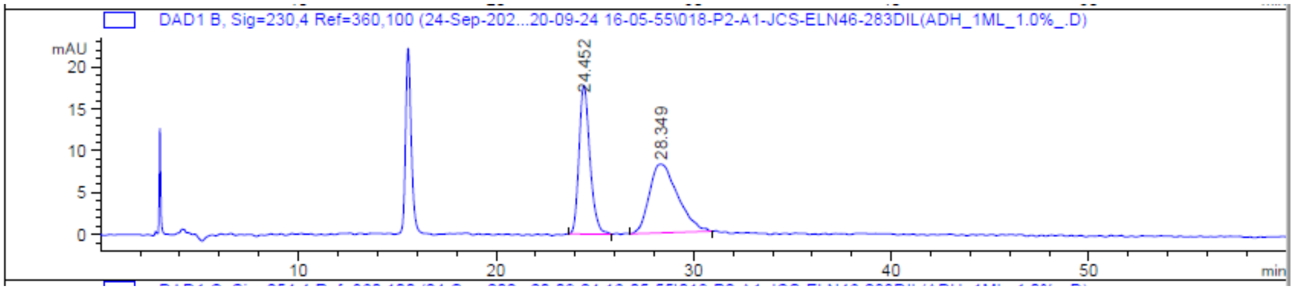

Signal 2: DAD1 B, Sig=230,4 Ref=360,100

Peak #	RetTime [min]	Type	Width [min]	Area [mAU*s]	Height [mAU]	Area %
1	24.452	BB	0.5913	682.12305	17.71050	45.3337
2	28.349	BB	1.1946	822.54871	8.19809	54.6663
Totals :				1504.67175	25.90858	

25 ℃, 1 mol % Rh_2(*R*-*p*-Ph-TPCP)$_4$, 2.5 equiv of vinyl pyridine, reaction performed according to procedure 3.3

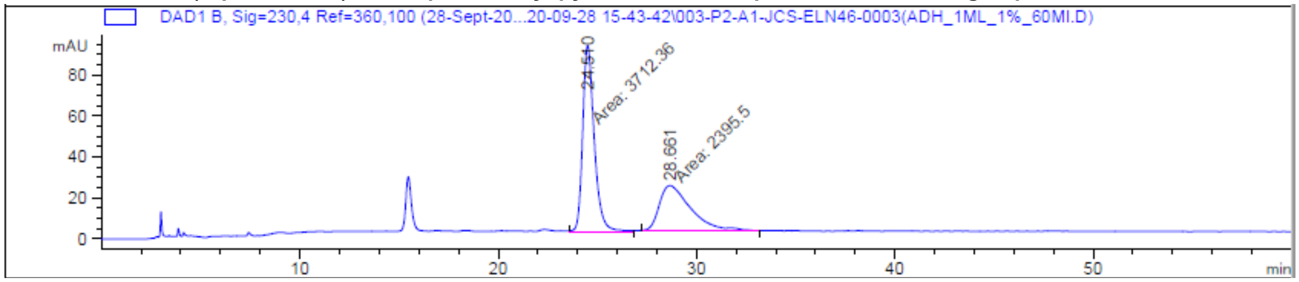

Signal 2: DAD1 B, Sig=230,4 Ref=360,100

Peak #	RetTime [min]	Type	Width [min]	Area [mAU*s]	Height [mAU]	Area %
1	24.510	MM	0.6798	3712.36304	91.01790	60.7801
2	28.661	MM	1.8180	2395.49829	21.96103	39.2199
Totals :				6107.86133	112.97893	

25 ℃, 1 mol % Rh_2(*S*-PTAD)$_4$, 2.5 equiv of vinyl pyridine, reaction performed according to procedure 3.3

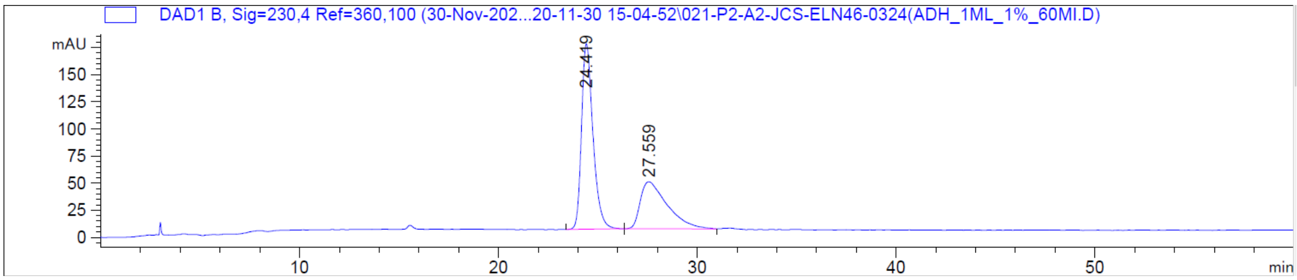

Signal 2: DAD1 B, Sig=230,4 Ref=360,100

Peak #	RetTime [min]	Type	Width [min]	Area [mAU*s]	Height [mAU]	Area %
1	24.419	BB	0.6104	6852.22852	170.68393	62.7498
2	27.559	BB	1.3235	4067.68384	43.24023	37.2502

Totals : 1.09199e4 213.92416

25 ℃, 1 mol % Rh_2(*R*-DOSP)$_4$, 2.5 equiv of vinyl pyridine, reaction performed according to procedure 3.3

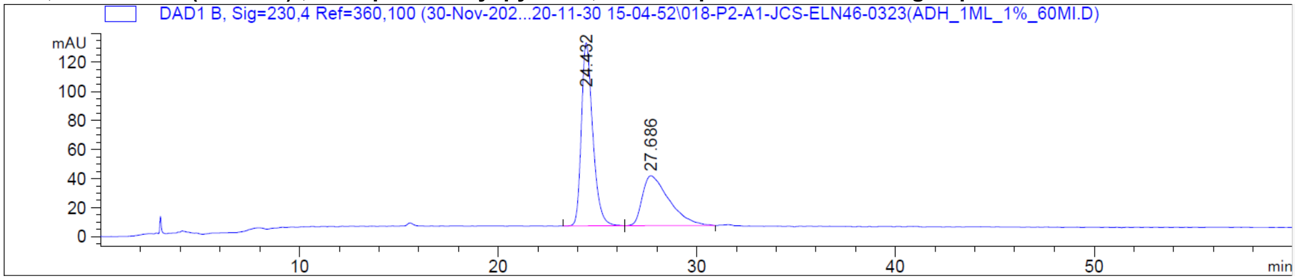

Signal 2: DAD1 B, Sig=230,4 Ref=360,100

Peak #	RetTime [min]	Type	Width [min]	Area [mAU*s]	Height [mAU]	Area %
1	24.432	BB	0.6066	5040.94482	126.03858	60.9987
2	27.686	BB	1.2961	3223.07275	34.36885	39.0013

Totals : 8264.01758 160.40743

25 ℃, 1 mol % Rh_2(*S*-TPPTTL)$_4$, 2.5 equiv of vinyl pyridine, reaction performed according to procedure 5.3

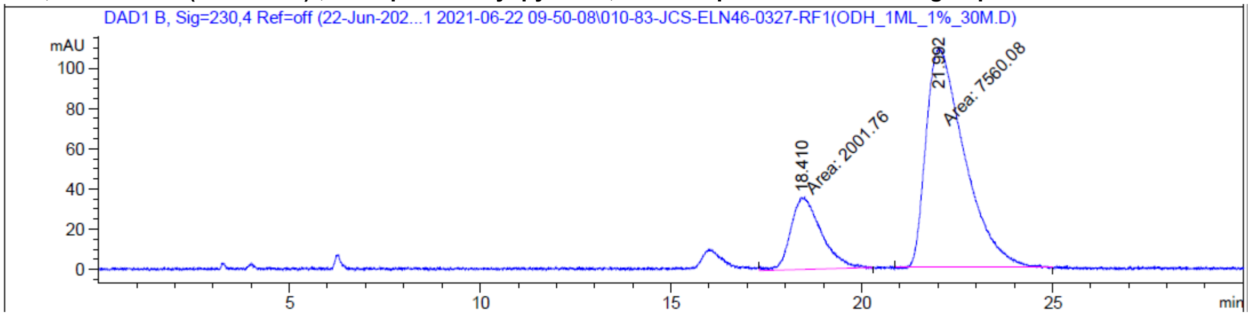

Signal 2: DAD1 B, Sig=230,4 Ref=off

Peak #	RetTime [min]	Type	Width [min]	Area [mAU*s]	Height [mAU]	Area %
1	18.410	MM	0.9206	2001.76111	36.24048	20.9349
2	21.992	MM	1.1563	7560.07959	108.97326	79.0651

Totals : 9561.84070 145.21374

25 ℃, 1 mol % Rh_2(*S*-TPPTTL)$_4$, 2.5 equiv of vinyl pyridine, trifluorotoluene as solvent, reaction performed according to procedure 3.3

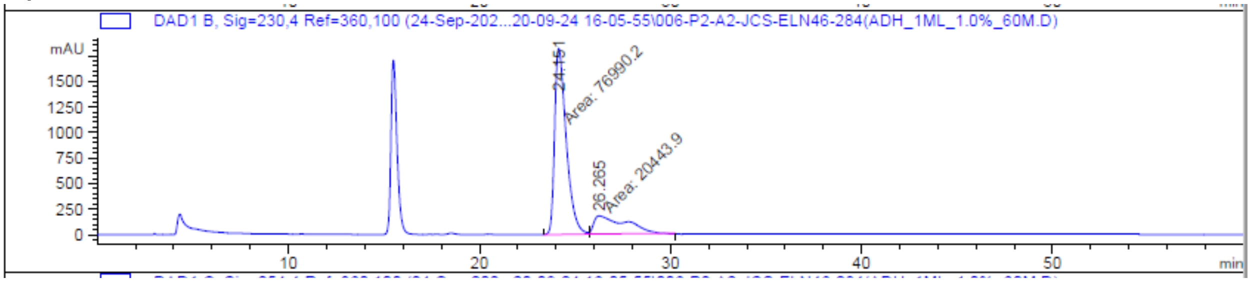

Signal 2: DAD1 B, Sig=230,4 Ref=360,100

Peak #	RetTime [min]	Type	Width [min]	Area [mAU*s]	Height [mAU]	Area %
1	24.151	MM	0.7051	7.69902e4	1819.95508	79.0177
2	26.265	MM	1.9219	2.04439e4	177.29097	20.9823

Totals : 9.74341e4 1997.24605

25 ℃, 1.0 mol % Rh_2(*S*-TPPTTL)$_4$, 2.5 equiv vinyl heterocycle, $(MeO)_2CO$ as solvent, reaction performed according to procedure 3.3

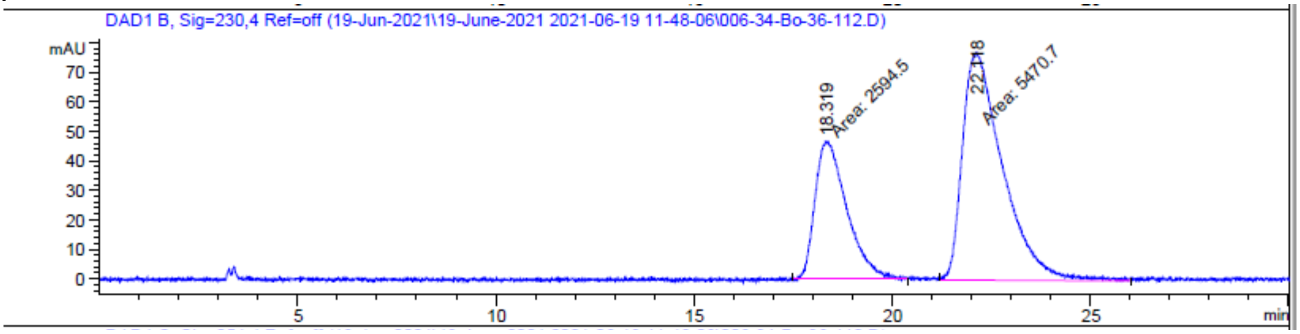

Signal 2: DAD1 B, Sig=230,4 Ref=off

Peak #	RetTime [min]	Type	Width [min]	Area [mAU*s]	Height [mAU]	Area %
1	18.319	MM	0.9232	2594.49976	46.84001	32.1691
2	22.118	MM	1.1747	5470.69922	77.62168	67.8309

Totals : 8065.19897 124.46169

0 ℃, 1.0 mol % Rh_2(*S*-TPPTTL)$_4$, 2.5 equiv vinyl heterocycle, CH_2Cl_2 as solvent, reaction performed according to procedure 3.3

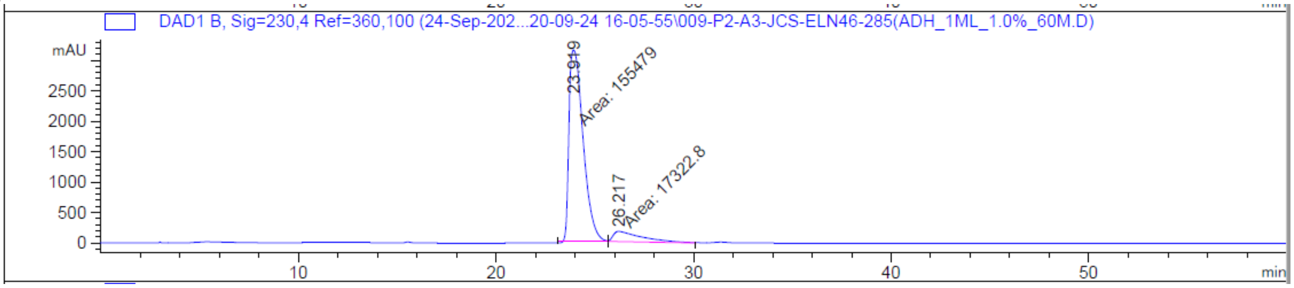

Signal 2: DAD1 B, Sig=230,4 Ref=360,100

Peak #	RetTime [min]	Type	Width [min]	Area [mAU*s]	Height [mAU]	Area %
1	23.919	MM	0.8237	1.55479e5	3145.96094	89.9753
2	26.217	MM	1.7319	1.73228e4	166.70610	10.0247

Totals : 1.72802e5 3312.66704

–50 ℃, 1.0 mol % Rh_2(*R*-TPPTTL)$_4$, 2.5 equiv vinyl heterocycle, CH_2Cl_2 as solvent, reaction performed according to procedure 3.3

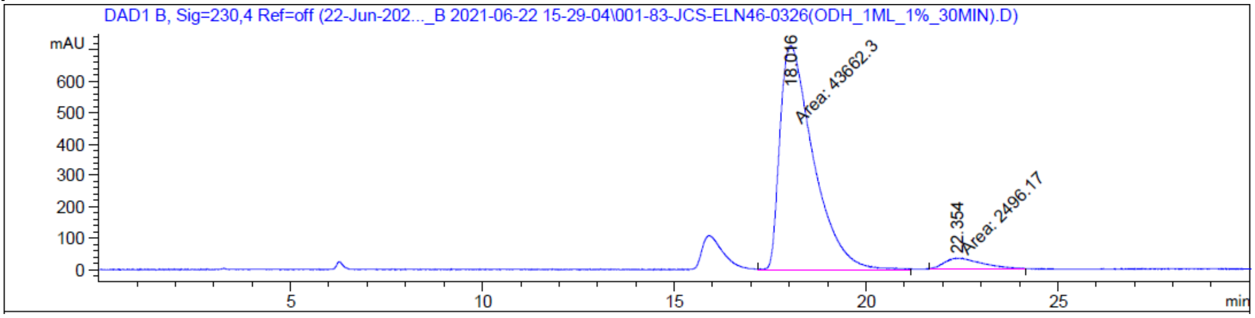

Signal 2: DAD1 B, Sig=230,4 Ref=off

Peak #	RetTime [min]	Type	Width [min]	Area [mAU*s]	Height [mAU]	Area %
1	18.016	MM	1.0163	4.36623e4	716.00336	94.5922
2	22.354	MM	1.1754	2496.16895	35.39403	5.4078
Totals :				4.61585e4	751.39739	

0 ℃, 1.0 mol % Rh_2(*S*-TPPTTL)$_4$, 5.0 equiv vinyl heterocycle, CH_2Cl_2 as solvent, reaction performed according to procedure 3.3

Chromatogram :
JCS-ELN46-0088(OD-H_30min_1ml_1%)23_chann
el2

System : Prostar LC System
Method : ADH_30min_1mL_1%-230nm
User : User1

Acquired : 10/10/2019 4:30:32 PM
Processed : 10/10/2019 5:01:43 PM
Printed : 9/30/2020 3:25:52 PM

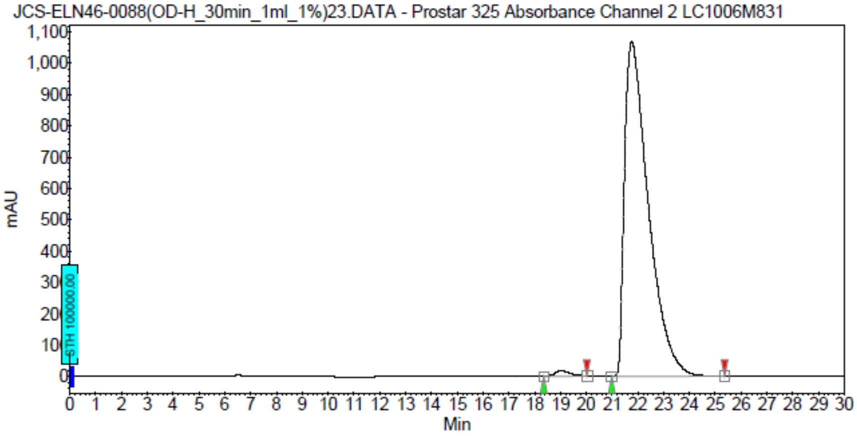

Peak results :

Index	Name	Time [Min]	Quantity [% Area]	Height [mAU]	Area [mAU.Min]	Area % [%]
1	UNKNOWN	19.04	1.08	17.1	12.5	1.084
2	UNKNOWN	21.76	98.92	1068.8	1142.0	98.916
Total			100.00	1085.9	1154.6	100.000

0 ℃, 1.0 mol % Rh_2(*S*-TPPTTL)$_4$, 10.0 equiv vinyl heterocycle, CH_2Cl_2 as solvent, reaction performed according to procedure 3.3

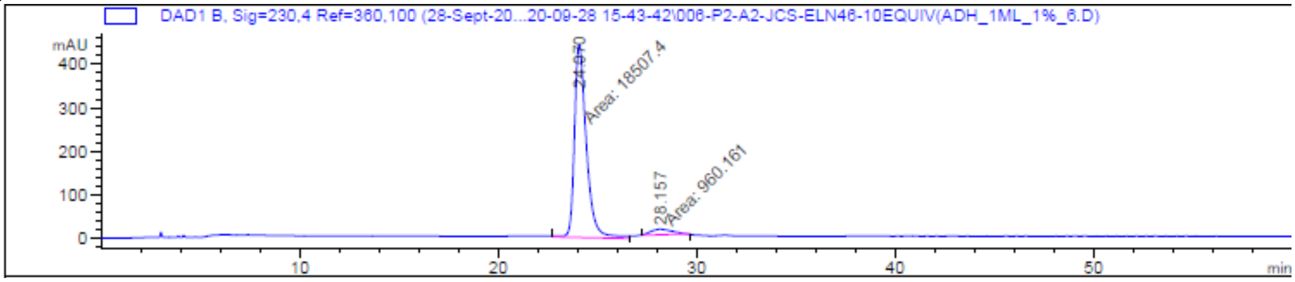


```
Signal 2: DAD1 B, Sig=230,4 Ref=360,100

Peak RetTime Type  Width     Area      Height     Area
  #   [min]        [min]   [mAU*s]     [mAU]       %
----|-------|----|-------|----------|----------|--------|
   1  24.070 MM    0.6933 1.85074e4  444.89807  95.0679
   2  28.157 MM    1.2559  960.16119  12.74203   4.9321

Totals :                  1.94676e4  457.64010
```

0 ℃, 1.0 mol % Rh_2(*S*-TPPTTL)$_4$, 1.5 equiv vinyl heterocycle, 3.5 equiv 2-chloropyridine, CH_2Cl_2 as solvent, reaction performed according to procedure 3.4

Chromatogram : JCS-ELN46-0085(OD-H_30min_1ml_1%)20_channel2

System : Prostar LC System
Method : ADH_30min_1mL_1%-230nm
User : User1

Acquired : 10/10/2019 3:13:03 PM
Processed : 10/10/2019 3:52:13 PM
Printed : 9/30/2020 3:25:44 PM

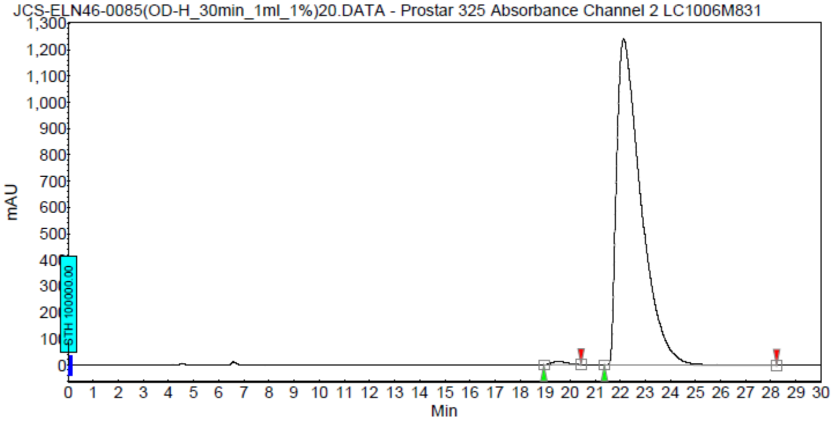

Peak results :

Index	Name	Time [Min]	Quantity [% Area]	Height [mAU]	Area [mAU.Min]	Area % [%]
1	UNKNOWN	19.54	0.67	13.0	9.4	0.670
2	UNKNOWN	22.14	99.33	1239.6	1387.0	99.330
Total			100.00	1252.6	1396.4	100.000

0 ℃, 1.0 mol % Rh_2(*S*-TPPTTL)$_4$, 1.5 equiv vinyl heterocycle, 2.5 equiv 2-chloropyridine, CH_2Cl_2 as solvent, reaction performed according to procedure 3.4

Chromatogram : JCS-ELN46-2.5P(ODH_30MIN_1ML_1%)9_channe l2

System : Prostar LC System
Method : ADH_30min_1mL_1%-230nm
User : User1

Acquired : 12/10/2019 4:34:30 PM
Processed : 12/11/2019 11:15:42 AM
Printed : 12/31/2019 12:04:53 PM

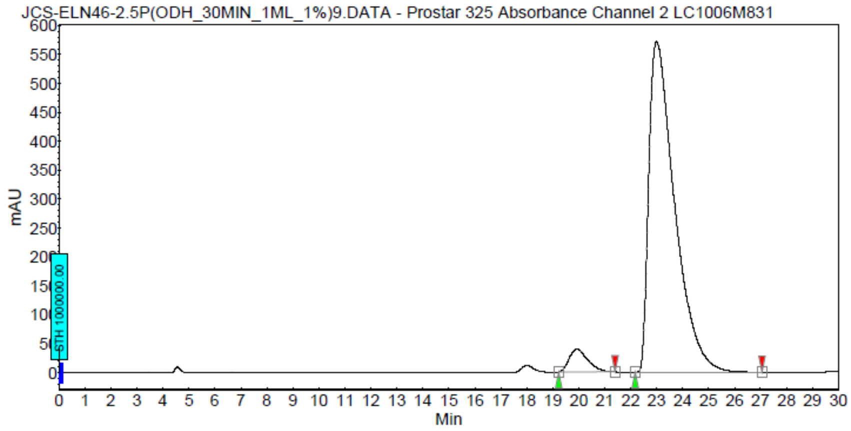

Peak results :

Index	Name	Time [Min]	Quantity [% Area]	Height [mAU]	Area [mAU.Min]	Area % [%]
1	UNKNOWN	19.92	4.75	39.3	32.8	4.753
2	UNKNOWN	23.00	95.25	571.8	657.6	95.247
Total			100.00	611.1	690.4	100.000

0 ℃, 1.0 mol % Rh_2(*S*-TPPTTL)$_4$, 1.5 equiv vinyl heterocycle, 1.5 equiv 2-chloropyridine, CH_2Cl_2 as solvent, reaction performed according to procedure 3.4

Chromatogram : JCS-ELN46-1.5P(ODH_30MIN_1ML_1%)6_channe l2

System : Prostar LC System
Method : ADH_30min_1mL_1%-230nm
User : User1

Acquired : 12/10/2019 3:17:21 PM
Processed : 12/11/2019 11:15:02 AM
Printed : 12/31/2019 12:04:31 PM

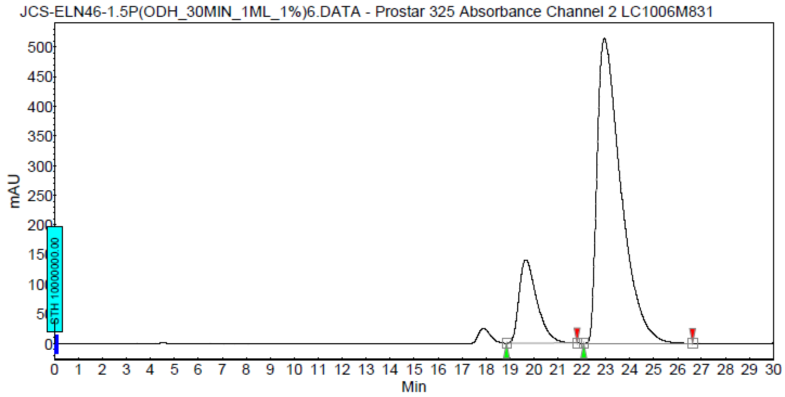

Peak results :

Index	Name	Time [Min]	Quantity [% Area]	Height [mAU]	Area [mAU.Min]	Area % [%]
1	UNKNOWN	19.66	17.07	140.3	121.7	17.068
2	UNKNOWN	22.94	82.93	513.6	591.4	82.932
Total			100.00	653.9	713.1	100.000

0 ℃, 1.0 mol % Rh_2(*S*-TPPTTL)$_4$, 1.5 equiv vinyl heterocycle, 3.5 equiv 2-chloropyridine, CH_2Cl_2 as solvent, no drying additive present, reaction performed according to procedure 3.4

Chromatogram : JCS-ELN46-0046re(ADH_1ml_1%_60min)2_channel1

System : Prostar LC System
Method : DACHDNB_30min_1mL_1%-230nm
User : User1

Acquired : 8/28/2019 5:38:18 PM
Processed : 8/29/2019 11:36:36 AM
Printed : 9/30/2020 3:29:25 PM

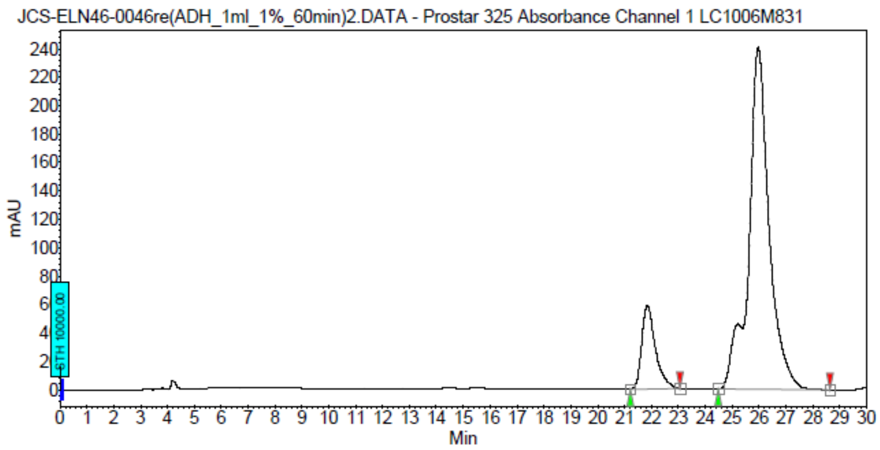

Peak results :

Index	Name	Time [Min]	Quantity [% Area]	Height [mAU]	Area [mAU.Min]	Area % [%]
1	UNKNOWN	21.84	14.11	59.4	34.9	14.110
2	UNKNOWN	25.97	85.89	241.1	212.6	85.890
Total			100.00	300.5	247.5	100.000

0 ℃, 1.0 mol % Rh_2(*S*-TPPTTL)$_4$, 1.5 equiv vinyl heterocycle, 3.5 equiv 2-chloropyridine, CH_2Cl_2 as solvent, 10 equiv HFIP, reaction performed according to procedure 3.5

Chromatogram : JCS-ELN46-0185(ODH_1ML_1%_30MIN)67_channel2

System : Prostar LC System
Method : ADH_30min_1mL_1%-230nm
User : User1

Acquired : 12/11/2019 10:42:37 PM
Processed : 12/12/2019 9:45:36 AM
Printed : 9/12/2020 12:15:49 PM

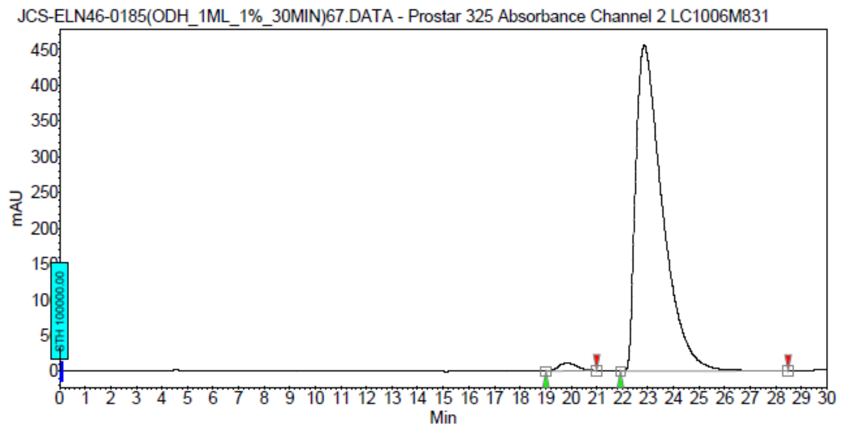

Peak results :

Index	Name	Time [Min]	Quantity [% Area]	Height [mAU]	Area [mAU.Min]	Area % [%]
1	UNKNOWN	19.82	1.71	11.3	9.6	1.714
2	UNKNOWN	22.86	98.29	455.9	550.6	98.286
Total			100.00	467.2	560.2	100.000

0 ℃, 1.0 mol % Rh_2(*S*-TPPTTL)$_4$, 1.5 equiv vinyl heterocycle, 3.5 equiv 2-chloropyridine, CH_2Cl_2 as solvent, 5 equiv HFIP, reaction performed according to procedure 3.5

Chromatogram :
JCS-ELN46-0144(ODSH_1ml_1%_30min)3_channel2

System : Prostar LC System
Method : ADH_30min_1mL_1%-230nm
User : User1

Acquired : 11/21/2019 11:16:20 AM
Processed : 11/21/2019 11:57:48 AM
Printed : 12/31/2019 12:18:07 PM

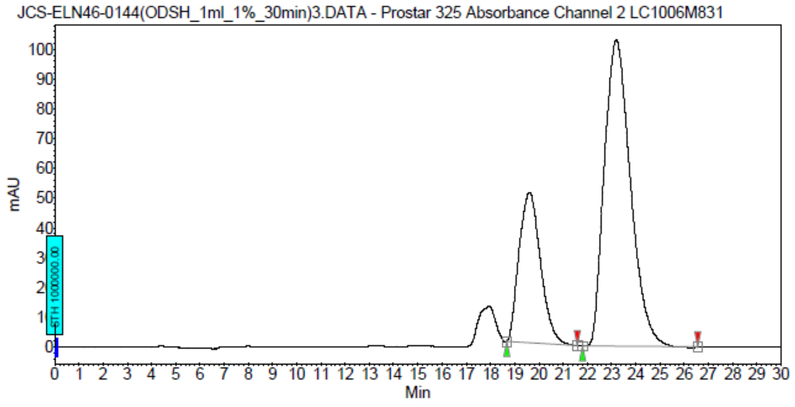

Peak results :

Index	Name	Time [Min]	Quantity [% Area]	Height [mAU]	Area [mAU.Min]	Area % [%]
1	UNKNOWN	19.61	28.83	50.4	51.9	28.830
2	UNKNOWN	23.20	71.17	102.7	128.0	71.170
Total			100.00	153.1	179.9	100.000

0 ℃, 1.0 mol % Rh_2(*S*-TPPTTL)$_4$, 1.5 equiv vinyl heterocycle, 3.5 equiv 2-chloropyridine, CH_2Cl_2 as solvent, 1.0 equiv HFIP, reaction performed according to procedure 3.5

Chromatogram :
JCS-ELN46-0146(ODSH_1ml_1%_30min)9_channel2

System : Prostar LC System
Method : ADH_30min_1mL_1%-230nm
User : User1

Acquired : 11/21/2019 1:54:03 PM
Processed : 11/25/2019 3:27:35 PM
Printed : 12/31/2019 12:18:17 PM

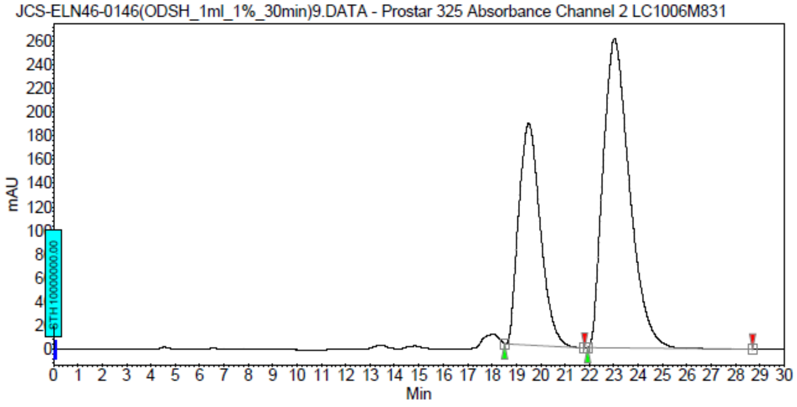

Peak results :

Index	Name	Time [Min]	Quantity [% Area]	Height [mAU]	Area [mAU.Min]	Area % [%]
1	UNKNOWN	19.51	37.00	187.6	192.8	36.999
2	UNKNOWN	23.03	63.00	260.8	328.3	63.001
Total			100.00	448.4	521.1	100.000

25 ℃, 1.0 mol % Rh_2(*S*-TPPTTL)$_4$, 1.5 equiv vinyl heterocycle, 3.5 equiv 2-chloropyridine, CH_2Cl_2 as solvent 20 equiv HFIP, reaction performed according to procedure 3.6

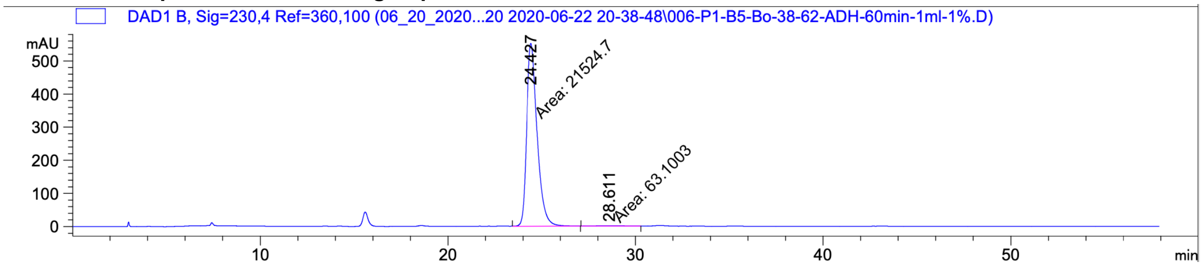

Signal 2: DAD1 B, Sig=230,4 Ref=360,100

Peak #	RetTime [min]	Type	Width [min]	Area [mAU*s]	Height [mAU]	Area %
1	24.427	MM	0.6497	2.15247e4	552.13348	99.7077
2	28.611	MM	1.2945	63.10027	8.12402e-1	0.2923
Totals :				2.15878e4	552.94589	

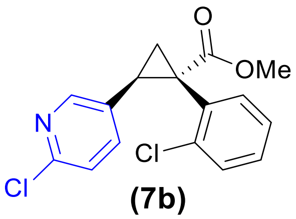

(7b)

Racemic Trace:

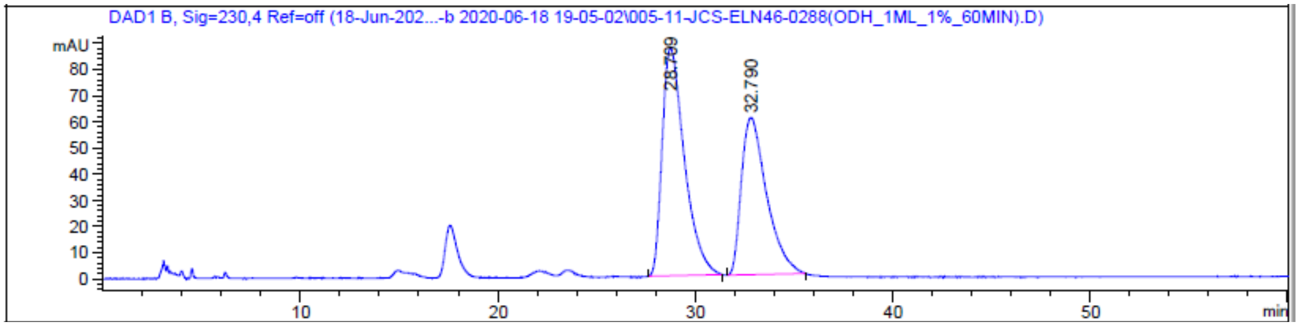

Signal 2: DAD1 B, Sig=230,4 Ref=off

Peak #	RetTime [min]	Type	Width [min]	Area [mAU*s]	Height [mAU]	Area %
1	28.709	VV R	0.9181	6819.27051	86.98462	56.0344
2	32.790	VV R	1.0426	5350.51172	60.01482	43.9656
Totals :				1.21698e4	146.99944	

0 ℃, 1.0 mol % Rh_2(*S*-TPPTTL)$_4$, 1.5 equiv vinyl heterocycle, 3.5 equiv 2-chloropyridine, CH_2Cl_2 as solvent, reaction performed according to procedure 3.4

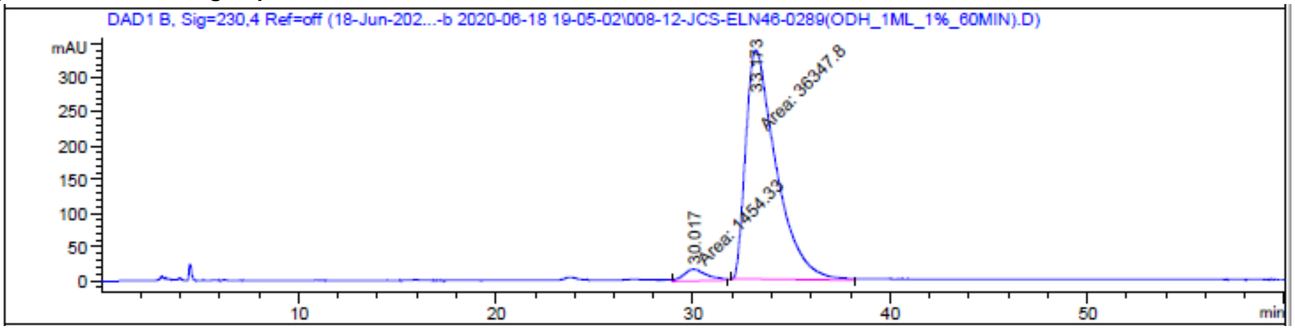


```
Signal 2: DAD1 B, Sig=230,4 Ref=off

Peak RetTime Type  Width     Area      Height     Area
  #   [min]        [min]   [mAU*s]     [mAU]        %
----|-------|----|-------|----------|----------|--------|
   1  30.017 MM     1.3712 1454.32605   17.67680   3.8472
   2  33.173 MM     1.7882 3.63478e4   338.76584  96.1528

Totals :                   3.78022e4   356.44263
```

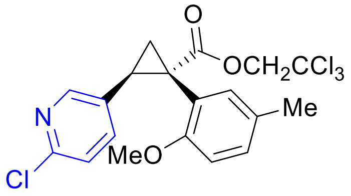

Table 2, Entry 7

Racemic Trace:

Chromatogram :
JCS-ELN-46-0018conc(ADH_30min_1mL_0.5%-230nm6_channel2

[illegible]
Method : ADH_30min_1mL_0.5%-230nm
User : User1

Acquired : 7/17/2019 9:11:27 AM
Processed : 9/21/2020 5:15:00 PM
Printed : 9/21/2020 5:15:08 PM

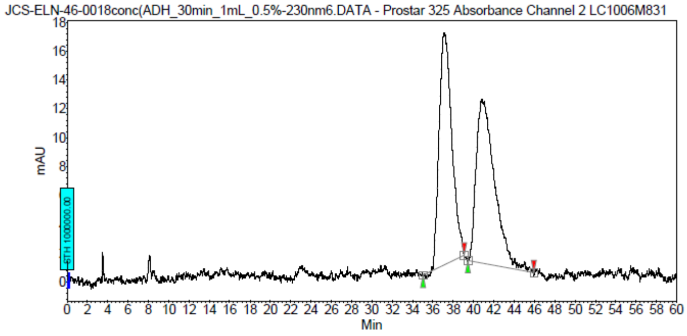

Peak results :

Index	Name	Time [Min]	Quantity [% Area]	Height [mAU]	Area [mAU.Min]	Area % [%]
1	UNKNOWN	37.07	49.19	16.1	23.1	49.195
2	UNKNOWN	40.77	50.81	11.4	23.9	50.805
Total			100.00	27.4	47.0	100.000

25 ℃, 1.0 mol % Rh_2(*R*-TPPTTL)$_4$, 2.5 equiv vinyl heterocycle, CH_2Cl_2 as solvent, reaction performed according to procedure 3.3

Chromatogram :
JCS-ELN-46-0019conc(ADH_30min_1mL_0.5%-2
30nm16_channel2

Method : ADH_30min_1mL_0.5%-230nm
User : User1

Acquired : 7/17/2019 10:59:49 AM
Processed : 12/31/2019 12:36:29 PM
Printed : 9/21/2020 5:14:37 PM

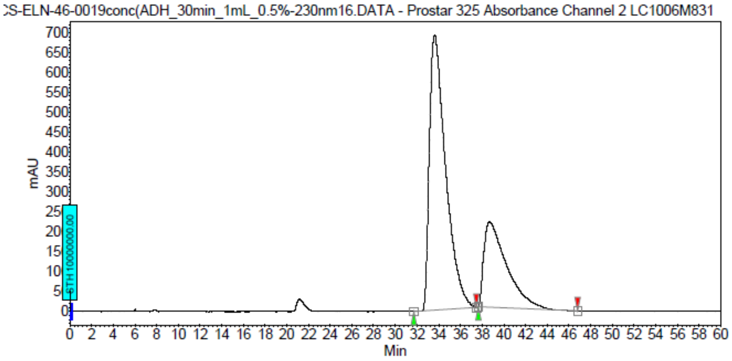

Peak results :

Index	Name	Time [Min]	Quantity [% Area]	Height [mAU]	Area [mAU.Min]	Area % [%]
1	UNKNOWN	33.63	69.60	690.8	1200.9	69.601
2	UNKNOWN	38.71	30.40	215.1	524.5	30.399
Total			100.00	905.9	1725.4	100.000

Racemic Trace:

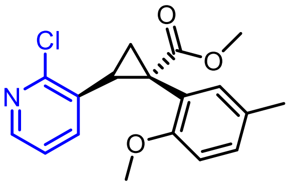

38

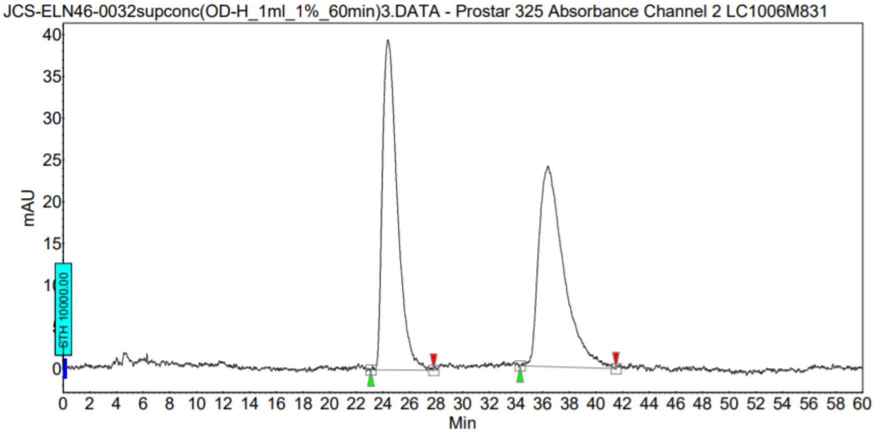

Peak results :

Index	Name	Time [Min]	Quantity [% Area]	Height [mAU]	Area [mAU.Min]	Area % [%]
1	UNKNOWN	24.36	49.68	39.5	51.3	49.675
2	UNKNOWN	36.40	50.32	24.0	52.0	50.325
Total			100.00	63.6	103.2	100.000

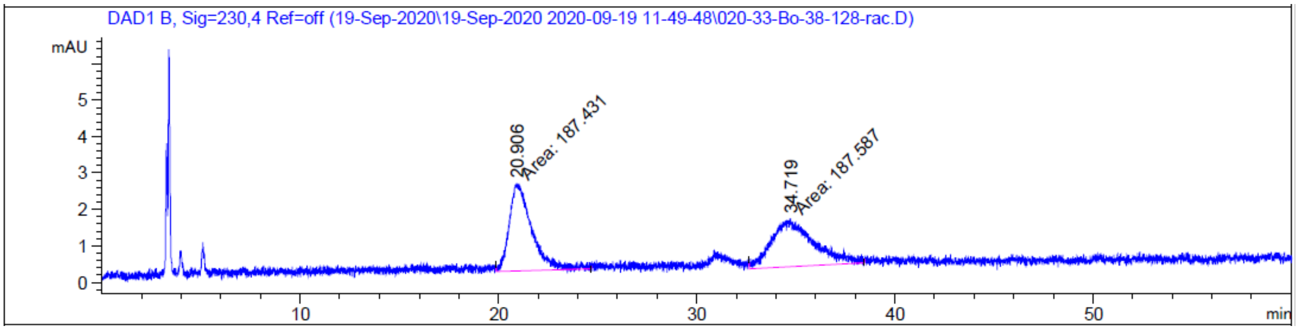


```
Signal 2: DAD1 B, Sig=230,4 Ref=off

Peak RetTime Type  Width     Area      Height     Area
  #   [min]        [min]   [mAU*s]     [mAU]       %
----|-------|----|-------|----------|----------|--------|
   1  20.906 MM     1.2927  187.43066    2.41649  49.9792
   2  34.719 MM     2.3695  187.58693    1.31948  50.0208

Totals :                    375.01759    3.73597
```

0 ℃, 1.0 mol % Rh_2(*R*-TPPTTL)$_4$ 5.0 equiv vinyl heterocycle, CH_2Cl_2 as solvent, reaction performed according to procedure 3.3

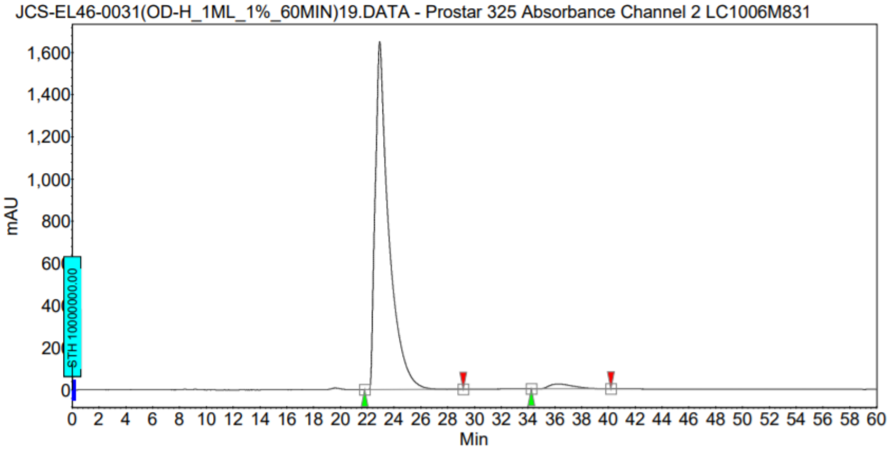

Peak results :

Index	Name	Time [Min]	Quantity [% Area]	Height [mAU]	Area [mAU.Min]	Area % [%]
1	UNKNOWN	22.93	97.43	1649.8	1929.1	97.427
2	UNKNOWN	36.26	2.57	23.4	50.9	2.573
Total			100.00	1673.3	1980.0	100.000

0 ℃, 1.0 mol % Rh_2(*S*-TPPTTL)$_4$, 1.5 equiv vinyl heterocycle, 3.5 equiv 2-chloropyridine, CH_2Cl_2 as solvent, 10 equiv HFIP, reaction performed according to procedure 3.5

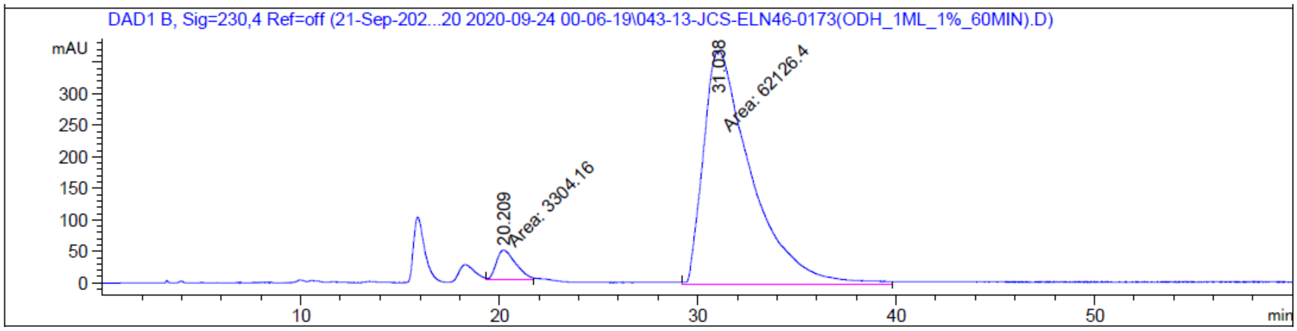

Signal 2: DAD1 B, Sig=230,4 Ref=off

Peak #	RetTime [min]	Type	Width [min]	Area [mAU*s]	Height [mAU]	Area %
1	20.209	MM	1.1572	3304.15601	47.58920	5.0499
2	31.038	MM	2.7909	6.21264e4	371.00339	94.9501

Totals : 6.54305e4 418.59259

Racemic Trace:

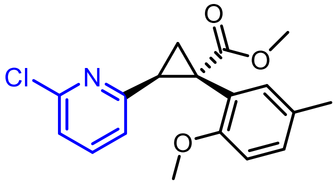

39

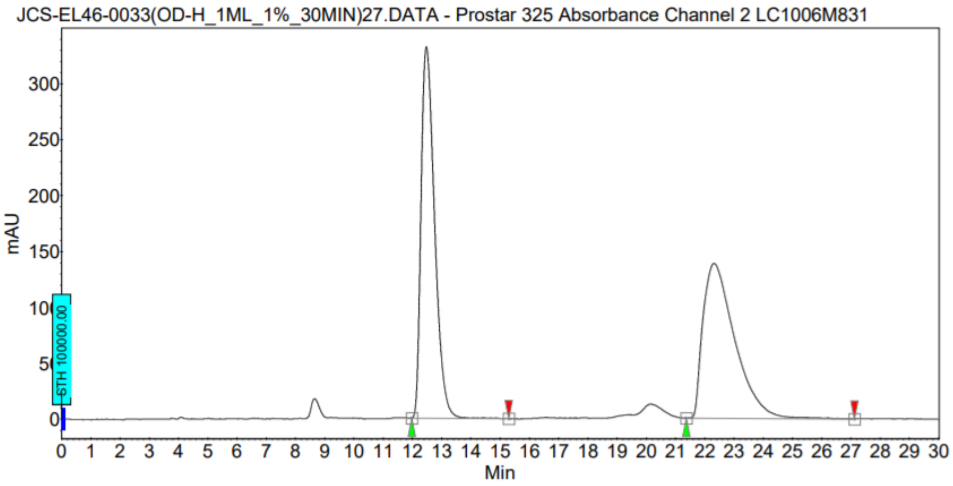

Peak results :

Index	Name	Time [Min]	Quantity [% Area]	Height [mAU]	Area [mAU.Min]	Area % [%]
1	UNKNOWN	12.48	50.48	332.2	178.2	50.479
2	UNKNOWN	22.31	49.52	138.6	174.9	49.521
Total			100.00	470.8	353.1	100.000

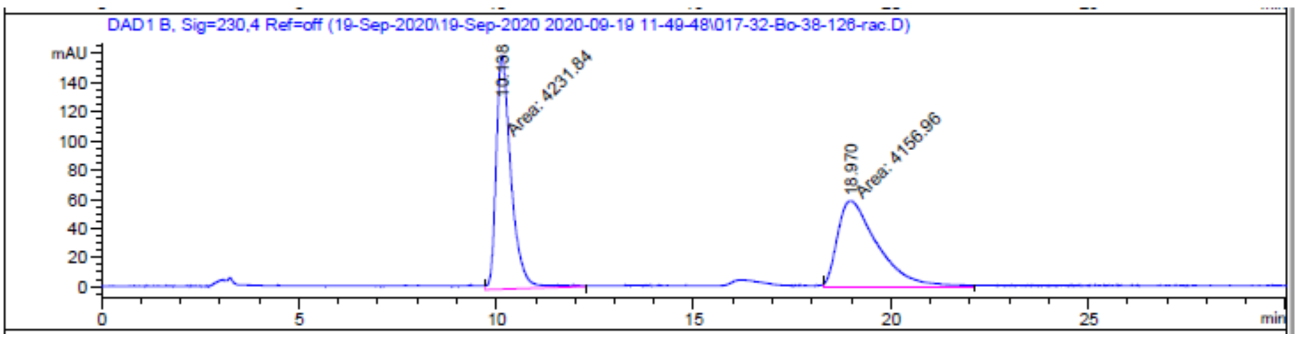

Signal 2: DAD1 B, Sig=230,4 Ref=off

Peak #	RetTime [min]	Type	Width [min]	Area [mAU*s]	Height [mAU]	Area %
1	10.138	MM	0.4400	4231.83789	160.27992	50.4463
2	18.970	MM	1.1751	4156.95947	58.95683	49.5537
Totals :				8388.79736	219.23675	

0 ℃, 1.0 mol % Rh_2(*R*-TPPTTL)$_4$ 5.0 equiv vinyl heterocycle, CH_2Cl_2 as solvent, reaction performed according to procedure 3.3

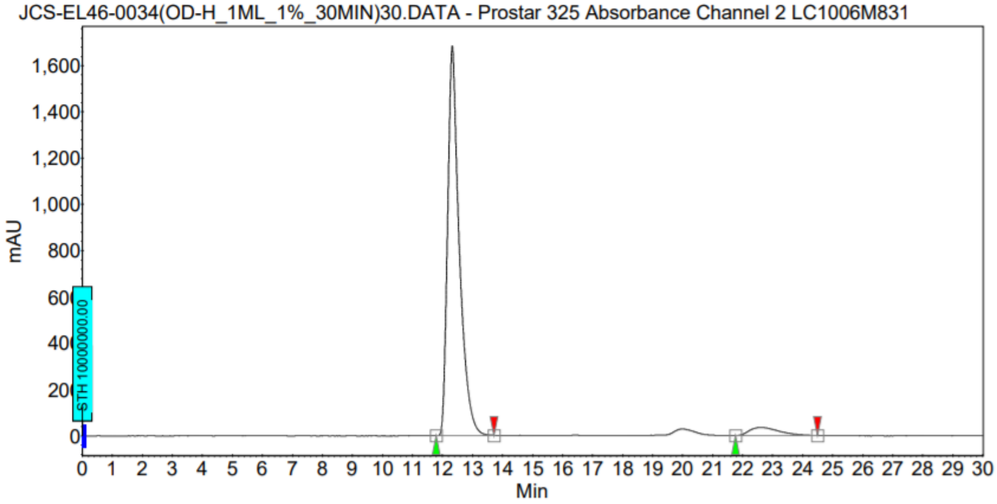

Peak results :

Index	Name	Time [Min]	Quantity [% Area]	Height [mAU]	Area [mAU.Min]	Area % [%]
1	UNKNOWN	12.32	94.97	1683.9	756.0	94.972
2	UNKNOWN	22.59	5.03	35.0	40.0	5.028
Total			100.00	1718.9	796.0	100.000

0 ℃, 1.0 mol % Rh_2(*S*-TPPTTL)$_4$, 1.5 equiv vinyl heterocycle, 3.5 equiv 2-chloropyridine, CH_2Cl_2 as solvent, 10 equiv HFIP, reaction performed according to procedure 3.5

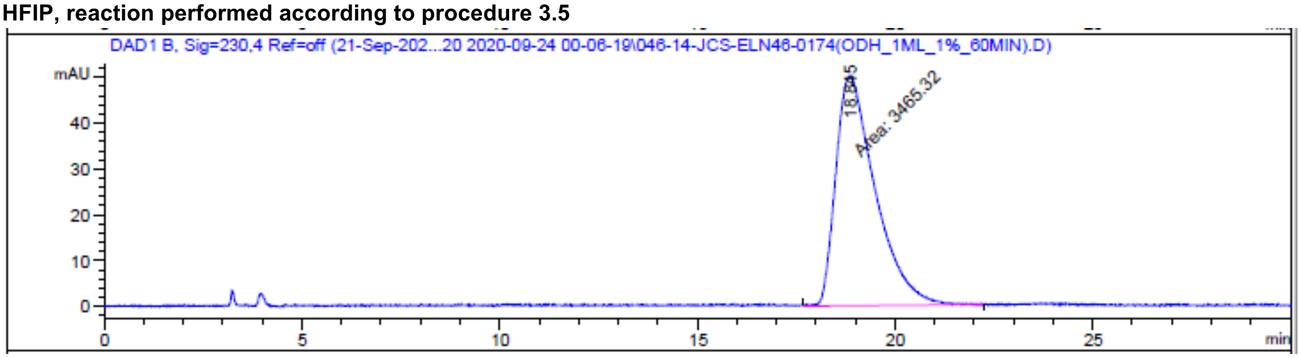

Signal 2: DAD1 B, Sig=230,4 Ref=off

Peak #	RetTime [min]	Type	Width [min]	Area [mAU*s]	Height [mAU]	Area %
1	18.845	MM	1.1559	3465.31787	49.96665	100.0000
Totals :				3465.31787	49.96665	

Racemic trace:

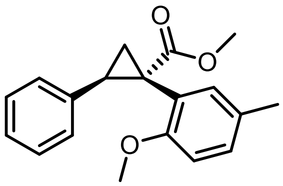

40

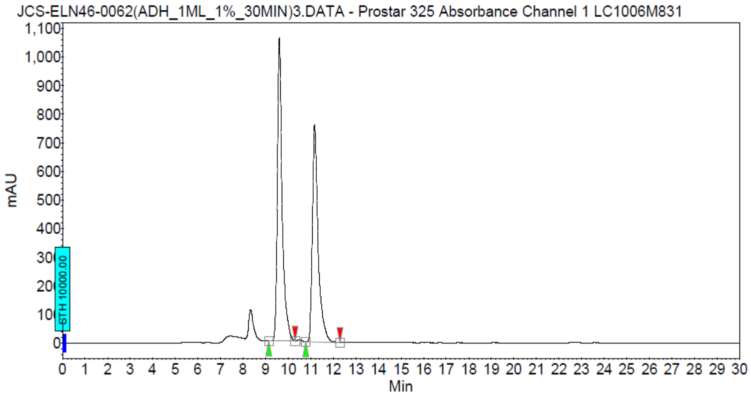

Peak results :

Index	Name	Time [Min]	Quantity [% Area]	Height [mAU]	Area [mAU.Min]	Area % [%]
1	UNKNOWN	9.61	53.67	1060.1	260.5	53.666
2	UNKNOWN	11.17	46.33	760.0	224.9	46.334
Total			100.00	1820.2	485.3	100.000

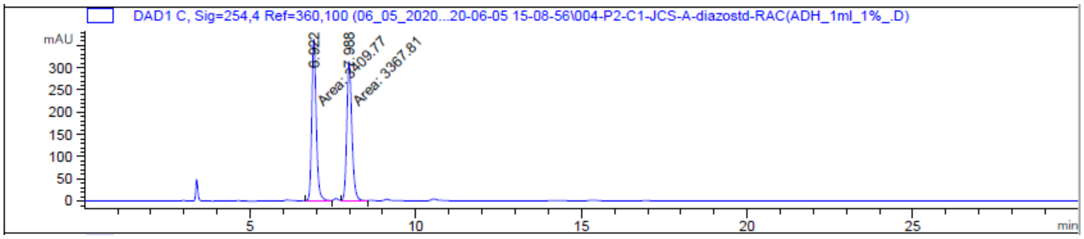

```
Signal 3: DAD1 C, Sig=254,4 Ref=360,100

Peak RetTime Type  Width     Area      Height     Area
  #   [min]        [min]   [mAU*s]     [mAU]       %
----|-------|----|-------|----------|----------|--------|
   1   6.922 MM    0.1564 3409.76978  363.26288  50.3095
   2   7.988 MM    0.1792 3367.81470  313.22736  49.6905

Totals :                  6777.58447  676.49023
```

0 ℃, 1.0 mol % Rh_2(*S*-TPPTTL)$_4$ 5.0 equiv styrene, CH_2Cl_2 as solvent, reaction performed according to procedure 3.3

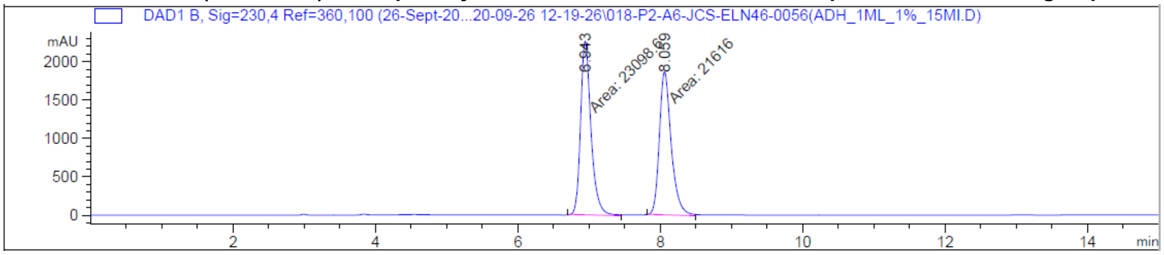


```
Signal 2: DAD1 B, Sig=230,4 Ref=360,100

Peak RetTime Type  Width     Area      Height     Area
  #   [min]        [min]   [mAU*s]     [mAU]       %
----|-------|----|-------|----------|----------|--------|
   1   6.943 MM    0.1701 2.30986e4 2263.13354  51.6578
   2   8.059 MM    0.1933 2.16160e4 1863.70142  48.3422

Totals :                  4.47146e4 4126.83496
```

0 ℃, 1.0 mol % Rh_2(*S*-TPPTTL)$_4$ 5.0 equiv styrene, 1.0 equiv 2-chloropyridine, CH_2Cl_2 as solvent, reaction performed according to procedure 3.4

Chromatogram : JCS-ELN46-0095DIL(ADH_30MIN_1ml_1%)3_channel1

System : Prostar LC System
Method : DACHDNB_30min_1mL_1%-230nm
User : User1

Acquired : 10/17/2019 9:55:29 AM
Processed : 10/17/2019 10:44:45 AM
Printed : 12/31/2019 12:32:50 PM

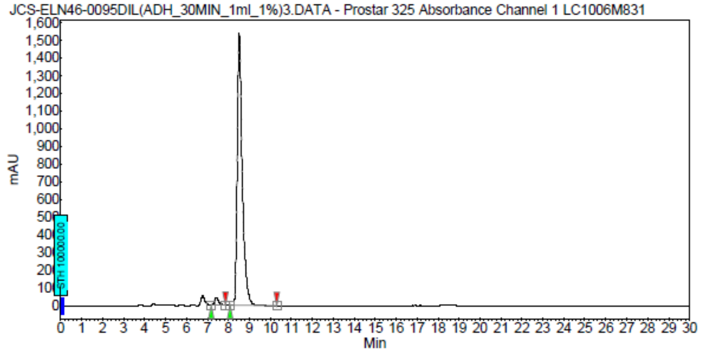

Peak results :

Index	Name	Time [Min]	Quantity [% Area]	Height [mAU]	Area [mAU.Min]	Area % [%]
2	UNKNOWN	7.41	2.41	44.3	10.6	2.413
1	UNKNOWN	8.52	97.59	1536.4	429.5	97.587
Total			100.00	1580.6	440.1	100.000

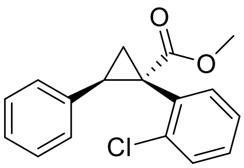

41

Racemic trace:

Chromatogram :
JCS-ELN46-0070(OJH_1ml_1%_30min)6_channe
l2
System : Prostar LC System
Method : OD_30min_1mL_1%_230nm
User : User1
Acquired : 10/2/2019 2:34:15 PM
Processed : 12/31/2019 12:46:22 PM
Printed : 12/31/2019 12:46:27 PM

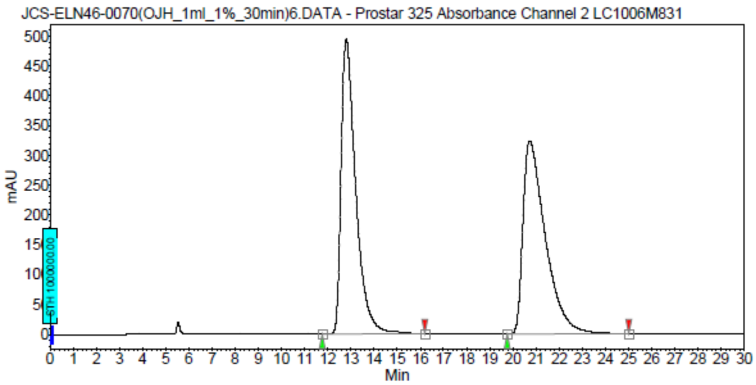

Peak results :

Index	Name	Time [Min]	Quantity [% Area]	Height [mAU]	Area [mAU.Min]	Area % [%]
1	UNKNOWN	12.80	49.85	493.8	360.6	49.854
2	UNKNOWN	20.73	50.15	323.5	362.7	50.146
Total			100.00	817.3	723.4	100.000

0 ℃, 1.0 mol % Rh_2(*S*-TPPTTL)$_4$ 5.0 equiv styrene, CH_2Cl_2 as solvent, reaction performed according to procedure 3.3

Chromatogram :
JCS-ELN46-0071(OJH_1ml_1%_30min)9_channe
l2
System : Prostar LC System
Method : OD_30min_1mL_1%_230nm
User : User1
Acquired : 10/2/2019 3:51:39 PM
Processed : 12/31/2019 12:46:44 PM
Printed : 12/31/2019 12:46:49 PM

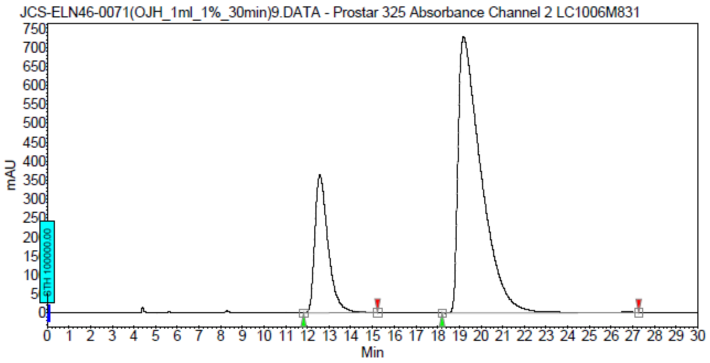

Peak results :

Index	Name	Time [Min]	Quantity [% Area]	Height [mAU]	Area [mAU.Min]	Area % [%]
1	UNKNOWN	12.57	22.27	363.9	257.5	22.266
2	UNKNOWN	19.20	77.73	728.1	898.8	77.734
Total			100.00	1092.0	1156.2	100.000

0 ℃, 1.0 mol % Rh_2(*S*-TPPTTL)$_4$ 5.0 equiv styrene, 1.0 equiv 2-chloropyridine, CH_2Cl_2 as solvent, reaction performed according to procedure 3.4

Chromatogram :
JCS-ELN46-0072F7-8RERUNOJH_1ML_1%_30MIN)58_channel2

System : Prostar LC System
Method : OD_30min_1mL_1%_230nm
User : User1

Acquired : 10/4/2019 5:19:31 PM
Processed : 12/31/2019 12:45:42 PM
Printed : 12/31/2019 12:45:46 PM

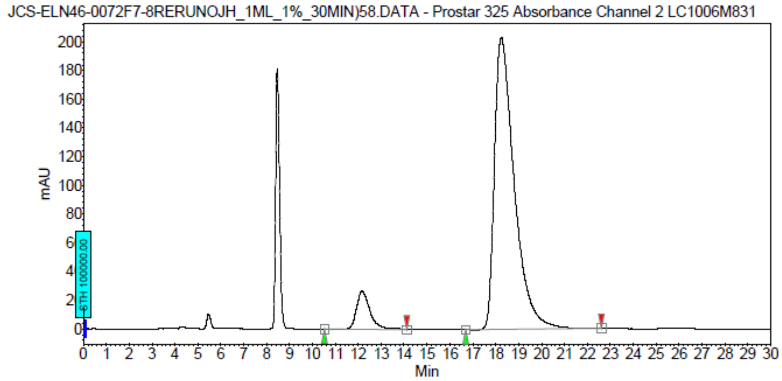

Peak results :

Index	Name	Time [Min]	Quantity [% Area]	Height [mAU]	Area [mAU.Min]	Area % [%]
1	UNKNOWN	12.17	8.19	26.5	18.2	8.194
2	UNKNOWN	18.24	91.81	203.3	204.2	91.806
Total			100.00	229.9	222.4	100.000

Racemic trace:

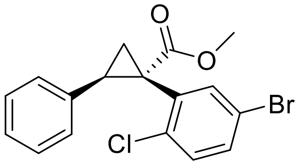

42

Chromatogram :
JCS-ELN46-0080(OJH_1ML_1%_30MIN)11_channel2

System : Prostar LC System
Method : OD_30min_1mL_1%_210_230nm
User : User1

Acquired : 10/8/2019 4:32:30 PM
Processed : 10/8/2019 5:19:04 PM
Printed : 12/31/2019 12:44:41 PM

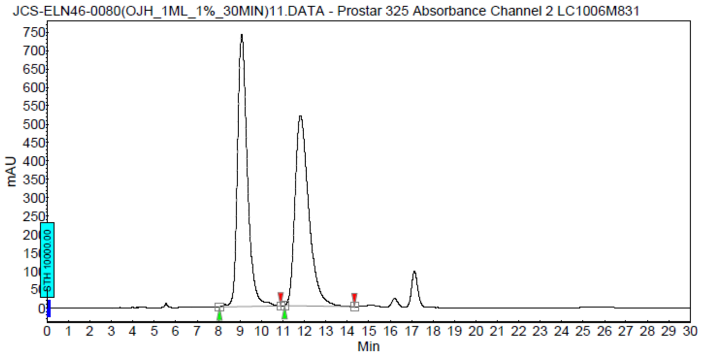

Peak results :

Index	Name	Time [Min]	Quantity [% Area]	Height [mAU]	Area [mAU.Min]	Area % [%]
1	UNKNOWN	9.07	50.51	739.1	394.0	50.510
2	UNKNOWN	11.82	49.49	516.8	386.0	49.490
Total			100.00	1255.9	780.0	100.000

0 ℃, 1.0 mol % Rh_2(*S*-TPPTTL)$_4$ 5.0 equiv styrene, CH_2Cl_2 as solvent, reaction performed according to procedure 3.3

Chromatogram : JCS-ELN46-0078(OJH_1ML_1%_30MIN)14_channel2

System : Prostar LC System
Method : OD_30min_1mL_1%_210_230nm
User : User1

Acquired : 10/8/2019 5:49:55 PM
Processed : 10/9/2019 8:51:34 AM
Printed : 12/31/2019 12:44:37 PM

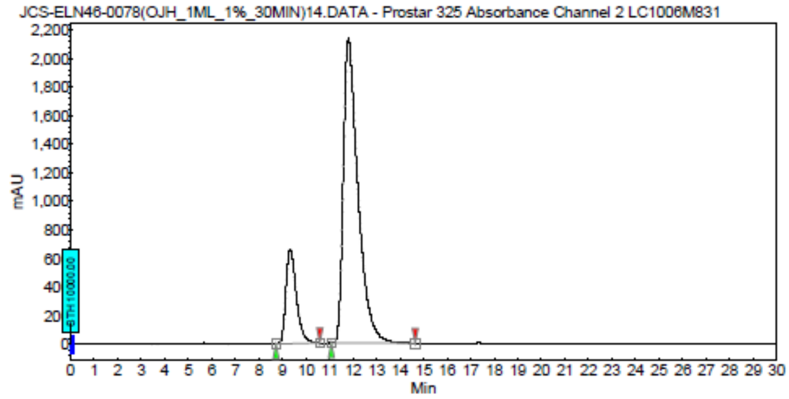

Peak results :

Index	Name	Time [Min]	Quantity [% Area]	Height [mAU]	Area [mAU.Min]	Area % [%]
1	UNKNOWN	9.33	18.01	664.7	338.8	18.006
2	UNKNOWN	11.81	81.99	2130.8	1542.8	81.994
Total			100.00	2795.5	1881.6	100.000

0 ℃, 1.0 mol % Rh_2(*S*-TPPTTL)$_4$ 5.0 equiv styrene, 1.0 equiv 2-chloropyridine, CH_2Cl_2 as solvent, reaction performed according to procedure 3.4

Chromatogram : JCS-ELN46-0079DIL(OJH_30MIN_1ML_1%)3_channel2

System : Prostar LC System
Method : OD_30min_1mL_1%_230nm
User : User1

Acquired : 10/9/2019 9:44:19 AM
Processed : 12/31/2019 12:43:26 PM
Printed : 12/31/2019 12:43:28 PM

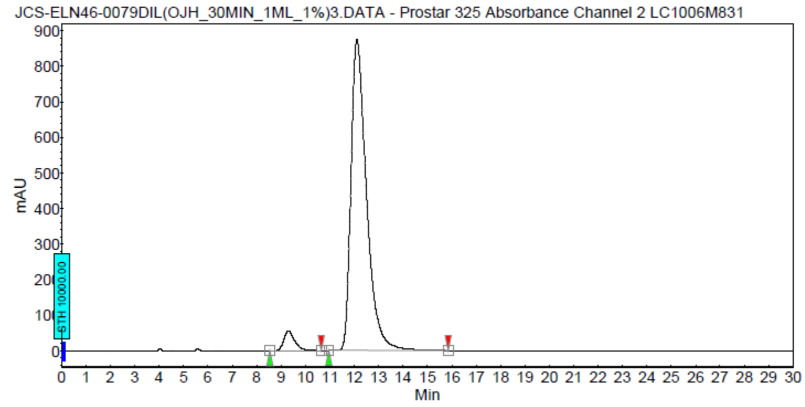

Peak results :

Index	Name	Time [Min]	Quantity [% Area]	Height [mAU]	Area [mAU.Min]	Area % [%]
1	UNKNOWN	9.30	4.30	55.7	29.0	4.303
2	UNKNOWN	12.11	95.70	872.6	643.9	95.697
Total			100.00	928.3	672.8	100.000

Racemic trace:

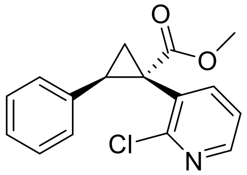

43

Chromatogram : JCS-ELN46-0040(ADH_1ml_1%_60min)2_channel2

System : Prostar LC System
Method : DACHDNB_30min_1mL_1%-230nm
User : User1

Acquired : 8/23/2019 10:20:38 AM
Processed : 8/26/2019 10:23:30 AM
Printed : 9/30/2020 3:27:58 PM

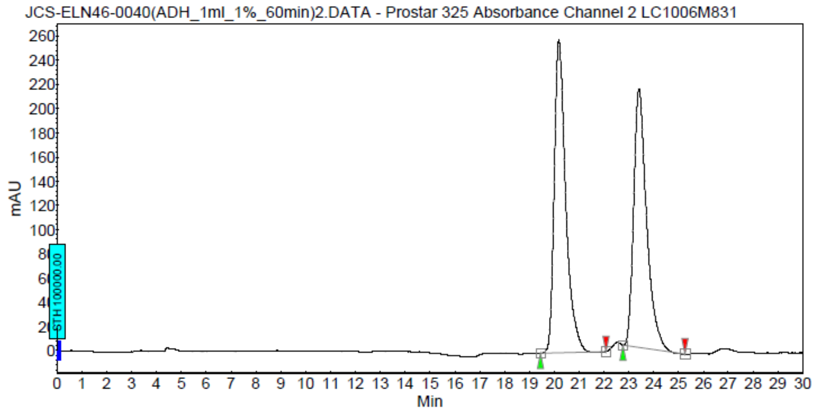

Peak results :

Index	Name	Time [Min]	Quantity [% Area]	Height [mAU]	Area [mAU.Min]	Area % [%]
1	UNKNOWN	20.19	51.66	258.3	138.2	51.657
2	UNKNOWN	23.41	48.34	214.0	129.4	48.343
Total			100.00	472.3	267.6	100.000

0 ℃, 1.0 mol % Rh_2(*R*-TPPTTL)$_4$ 5.0 equiv styrene, CH_2Cl_2 as solvent, reaction performed according to procedure 3.3

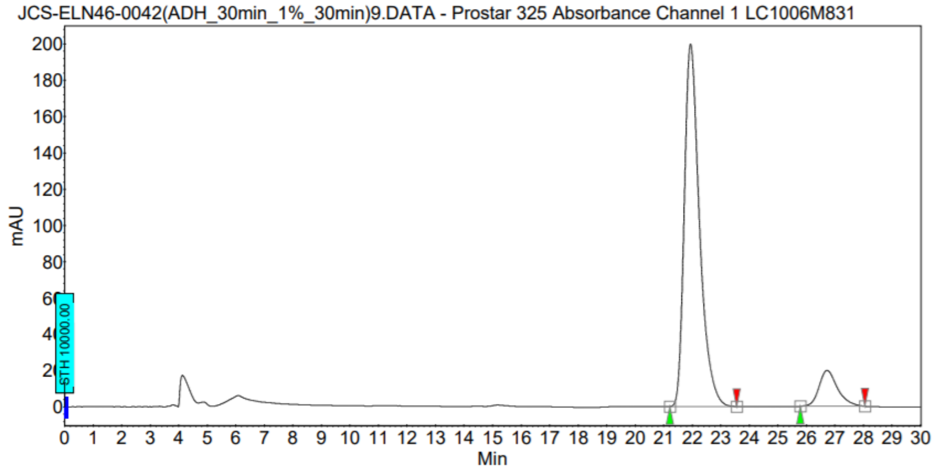

Peak results :

Index	Name	Time [Min]	Quantity [% Area]	Height [mAU]	Area [mAU.Min]	Area % [%]
1	UNKNOWN	21.94	89.24	199.8	123.6	89.238
2	UNKNOWN	26.72	10.76	19.8	14.9	10.762
Total			100.00	219.6	138.5	100.000

0 ℃, 1.0 mol % Rh_2(*S*-TPPTTL)$_4$ 5.0 equiv styrene, 1.0 equiv 2-chloropyridine, CH_2Cl_2 as solvent, reaction performed according to procedure 3.4

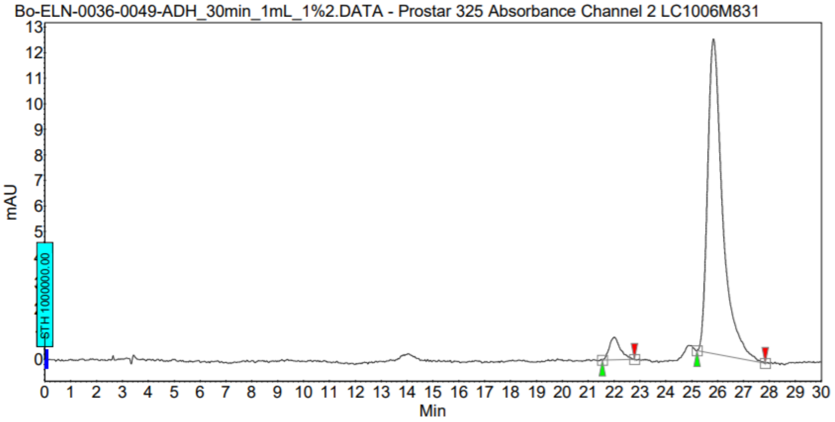

Peak results :

Index	Name	Time [Min]	Quantity [% Area]	Height [mAU]	Area [mAU.Min]	Area % [%]
2	UNKNOWN	22.01	5.01	0.9	0.4	5.010
1	UNKNOWN	25.84	94.99	12.3	8.3	94.990
Total			100.00	13.2	8.7	100.000

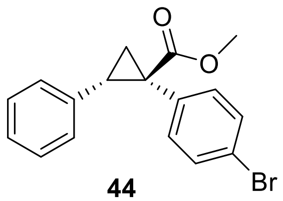

44

Racemic trace:

Chromatogram : JCS-ELN46-0109(OJH_1ml_1%_30min)3_channel1

System : Prostar LC System
Method : S4900_30min_1mL_1%_230nm
User : User1

Acquired : 10/29/2019 5:28:42 PM
Processed : 10/30/2019 9:10:55 AM
Printed : 12/31/2019 12:29:24 PM

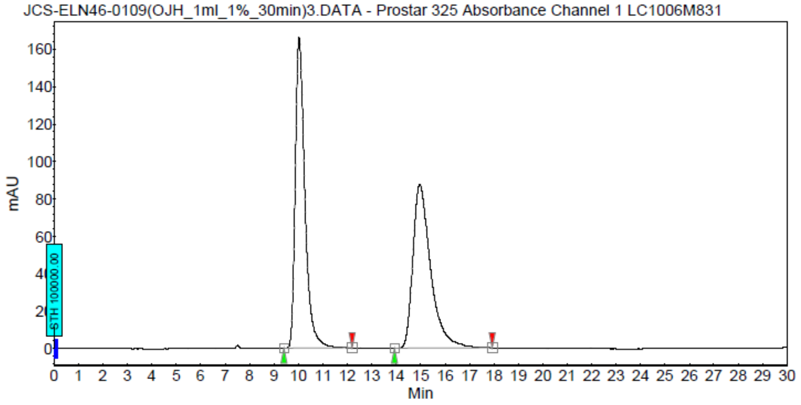

Peak results :

Index	Name	Time [Min]	Quantity [% Area]	Height [mAU]	Area [mAU.Min]	Area % [%]
1	UNKNOWN	10.03	50.57	165.9	75.6	50.571
2	UNKNOWN	14.97	49.43	87.5	73.9	49.429
Total			100.00	253.4	149.5	100.000

0 ℃ , 1.0 mol % Rh_2(*S*-TPPTTL)$_4$ 5.0 equiv styrene, CH_2Cl_2 as solvent, reaction performed according to procedure 3.3

Chromatogram :
JCS-ELN46-0110(OJH_1ml_1%_30min)6_channe l1

System : Prostar LC System
Method : S4900_30min_1mL_1%_230nm
User : User1

Acquired : 10/29/2019 6:46:05 PM
Processed : 10/30/2019 9:11:19 AM
Printed : 12/31/2019 12:29:32 PM

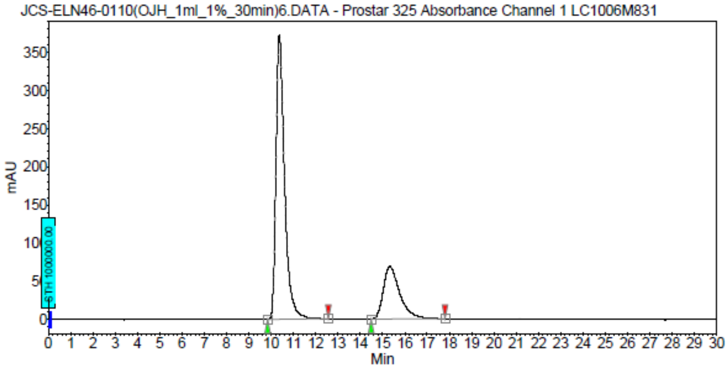

Peak results :

Index	Name	Time [Min]	Quantity [% Area]	Height [mAU]	Area [mAU.Min]	Area % [%]
1	UNKNOWN	10.37	73.78	371.9	166.1	73.781
2	UNKNOWN	15.33	26.22	68.7	59.0	26.219
Total			100.00	440.6	225.1	100.000

0 ℃, 1.0 mol % Rh_2(*S*-TPPTTL)$_4$ 5.0 equiv styrene, 1.0 equiv 2-chloropyridine, CH_2Cl_2 as solvent, reaction performed according to procedure 3.4

Chromatogram :
JCS-ELN46-0111(OJH_1ml_1%_30min)9_channe l1

System : Prostar LC System
Method : S4900_30min_1mL_1%_230nm
User : User1

Acquired : 10/29/2019 8:03:22 PM
Processed : 10/30/2019 9:11:45 AM
Printed : 12/31/2019 12:29:28 PM

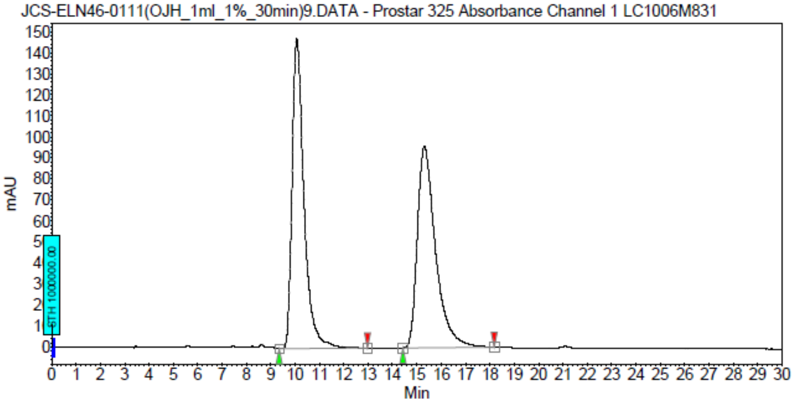

Peak results :

Index	Name	Time [Min]	Quantity [% Area]	Height [mAU]	Area [mAU.Min]	Area % [%]
1	UNKNOWN	10.07	50.07	147.5	83.6	50.070
2	UNKNOWN	15.31	49.93	95.7	83.4	49.930
Total			100.00	243.3	167.0	100.000

Racemic trace:

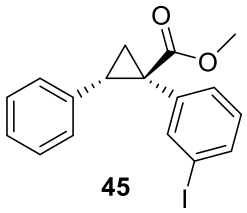

45

Chromatogram :
JCS-ELN46-0181(ODH_30MIN_1ML_1%)48_channel2

System : Prostar LC System
Method : ADH_30min_1mL_1%-230nm
User : User1

Acquired : 12/11/2019 4:01:46 PM
Processed : 12/12/2019 9:38:09 AM
Printed : 12/31/2019 12:05:13 PM

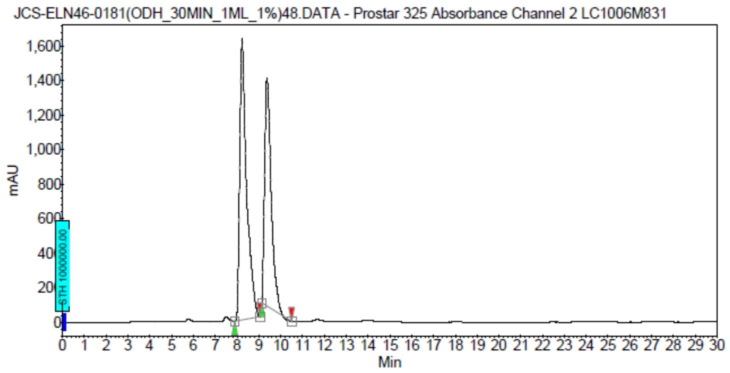

Peak results :

Index	Name	Time [Min]	Quantity [% Area]	Height [mAU]	Area [mAU.Min]	Area % [%]
1	UNKNOWN	8.23	56.24	1631.2	592.4	56.236
2	UNKNOWN	9.37	43.76	1320.4	461.0	43.764
Total			100.00	2951.6	1053.3	100.000

0 ℃, 1.0 mol % Rh_2(*S*-TPPTTL)$_4$ 5.0 equiv styrene, CH_2Cl_2 as solvent, reaction performed according to procedure 3.3

Chromatogram :
JCS-ELN46-0182(ODH_30MIN_1ML_1%)51_channel2

System : Prostar LC System
Method : ADH_30min_1mL_1%-230nm
User : User1

Acquired : 12/11/2019 5:19:44 PM
Processed : 12/12/2019 9:38:35 AM
Printed : 12/31/2019 12:05:08 PM

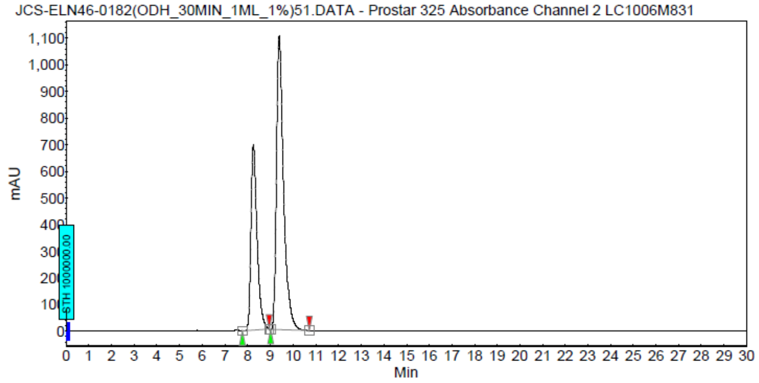

Peak results :

Index	Name	Time [Min]	Quantity [% Area]	Height [mAU]	Area [mAU.Min]	Area % [%]
1	UNKNOWN	8.25	35.34	699.4	221.1	35.336
2	UNKNOWN	9.39	64.66	1102.8	404.6	64.664
Total			100.00	1802.3	625.7	100.000

0 ℃, 1.0 mol % Rh_2(*S*-TPPTTL)$_4$ 5.0 equiv styrene, 1.0 equiv 2-chloropyridine, CH_2Cl_2 as solvent, reaction performed according to procedure 3.4

Chromatogram : JCS-ELN46-0183P1(ODH_30MIN_1ML_1%)54_channel2

System : Prostar LC System
Method : ADH_30min_1mL_1%-230nm
User : User1

Acquired : 12/11/2019 6:37:10 PM
Processed : 12/12/2019 9:39:14 AM
Printed : 12/31/2019 12:04:57 PM

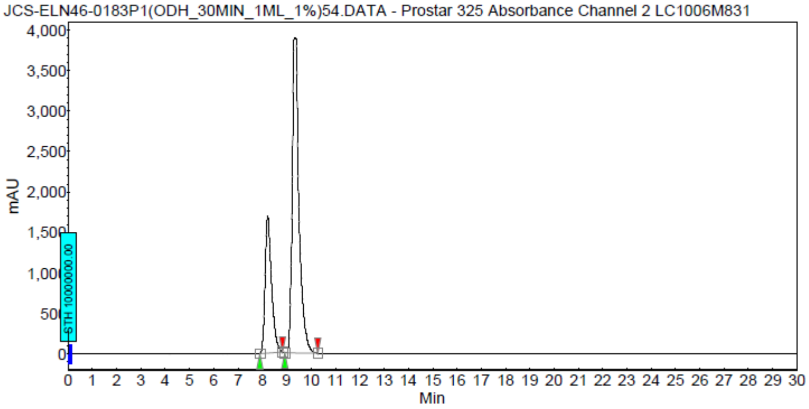

Peak results :

Index	Name	Time [Min]	Quantity [% Area]	Height [mAU]	Area [mAU.Min]	Area % [%]
1	UNKNOWN	8.21	29.17	1691.5	523.5	29.169
2	UNKNOWN	9.36	70.83	3884.5	1271.2	70.831
Total			100.00	5576.1	1794.8	100.000

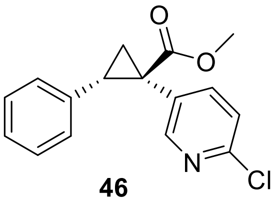

46

Racemic trace:

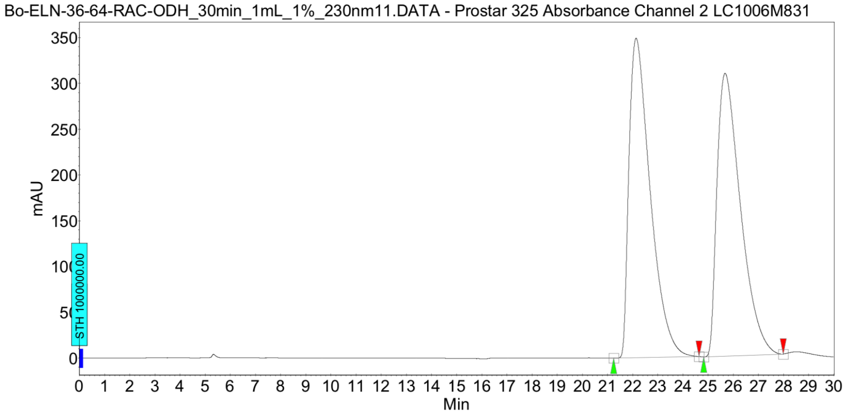

Peak results :

Index	Name	Time [Min]	Quantity [% Area]	Height [mAU]	Area [mAU.Min]	Area % [%]
1	UNKNOWN	22.14	50.51	349.2	341.9	50.507
2	UNKNOWN	25.67	49.49	309.1	335.0	49.493
Total			100.00	658.2	676.9	100.000

0 ℃, 1.0 mol % Rh_2(*S*-TPPTTL)$_4$ 5.0 equiv styrene, CH_2Cl_2 as solvent, reaction performed according to procedure 3.3

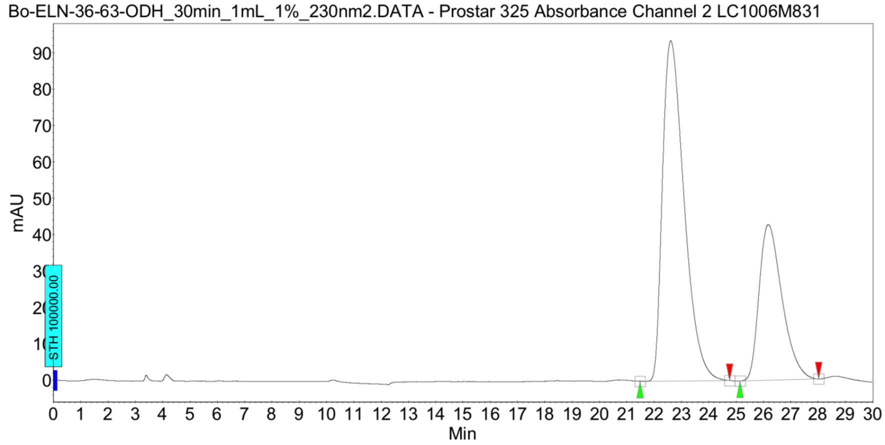

Peak results :

Index	Name	Time [Min]	Quantity [% Area]	Height [mAU]	Area [mAU.Min]	Area % [%]
1	UNKNOWN	22.61	67.37	93.5	86.0	67.366
2	UNKNOWN	26.18	32.63	42.8	41.6	32.634
Total			100.00	136.3	127.6	100.000

0 ℃, 1.0 mol % Rh_2(*S*-TPPTTL)$_4$ 5.0 equiv styrene, 1.0 equiv 2-chloropyridine, CH_2Cl_2 as solvent, reaction performed according to procedure 3.4

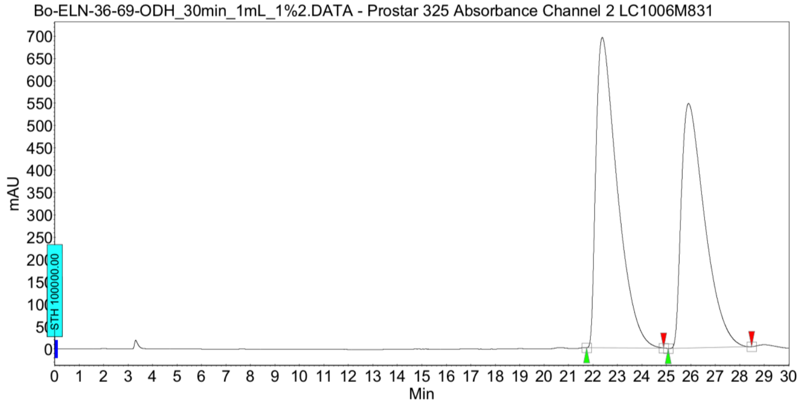

Peak results :

Index	Name	Time [Min]	Quantity [% Area]	Height [mAU]	Area [mAU.Min]	Area % [%]
1	UNKNOWN	22.38	53.63	694.8	701.8	53.635
2	UNKNOWN	25.91	46.37	547.3	606.6	46.365
Total			100.00	1242.1	1308.4	100.000

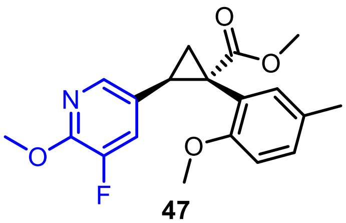

47

Racemic trace:

Chromatogram : JCS-ELN46-0160(ODH_30min_1ml_1%)6_channel2

System : Prostar LC System
Method : ADH_30min_1mL_1%-230nm
User : User1

Acquired : 11/26/2019 12:28:10 PM
Processed : 11/30/2019 1:57:41 PM
Printed : 12/31/2019 12:16:50 PM

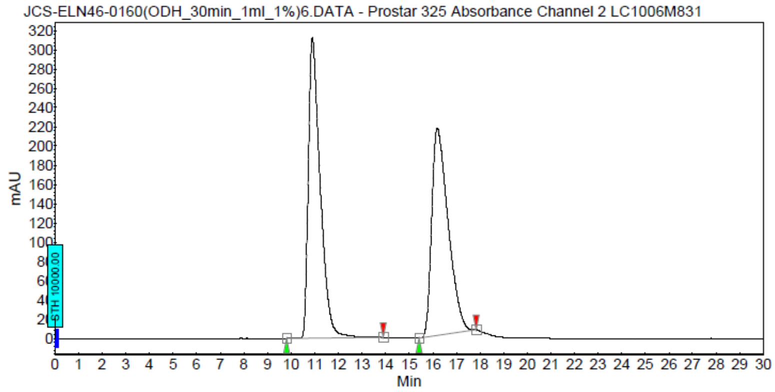

Peak results :

Index	Name	Time [Min]	Quantity [% Area]	Height [mAU]	Area [mAU.Min]	Area % [%]
1	UNKNOWN	10.90	51.23	312.1	182.2	51.230
2	UNKNOWN	16.19	48.77	215.7	173.4	48.770
Total			100.00	527.8	355.6	100.000

0 ℃, 1.0 mol % Rh_2(*S*-TPPTTL)$_4$ 5.0 equiv vinyl heterocycle, CH_2Cl_2 as solvent, reaction performed according to procedure 3.3

Chromatogram : JCS-ELN46-0161(ODH_30min_1ml_1%)18_channel2

System : Prostar LC System
Method : ADH_30min_1mL_1%-230nm
User : User1

Acquired : 11/26/2019 5:37:29 PM
Processed : 11/27/2019 9:48:33 AM
Printed : 11/30/2019 1:58:04 PM

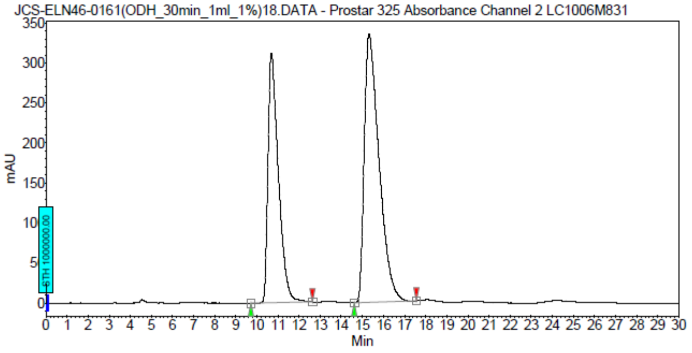

Peak results :

Index	Name	Time [Min]	Quantity [% Area]	Height [mAU]	Area [mAU.Min]	Area % [%]
2	UNKNOWN	10.69	39.64	312.2	178.6	39.641
1	UNKNOWN	15.32	60.36	335.3	272.0	60.359
Total			100.00	647.6	450.6	100.000

0 ℃, 1.0 mol % Rh_2(*S*-TPPTTL)$_4$ 1.5 equiv vinyl heterocycle, 3.5 equiv 2-chloropyridine, CH_2Cl_2 as solvent, reaction performed according to procedure 3.4

Chromatogram :
JCS-ELN46-0162(ODH_30min_1ml_1%)21_chann el2

System : Prostar LC System
Method : ADH_30min_1mL_1%-230nm
User : User1

Acquired : 11/26/2019 6:54:56 PM
Processed : 11/30/2019 1:58:24 PM
Printed : 12/31/2019 12:16:37 PM

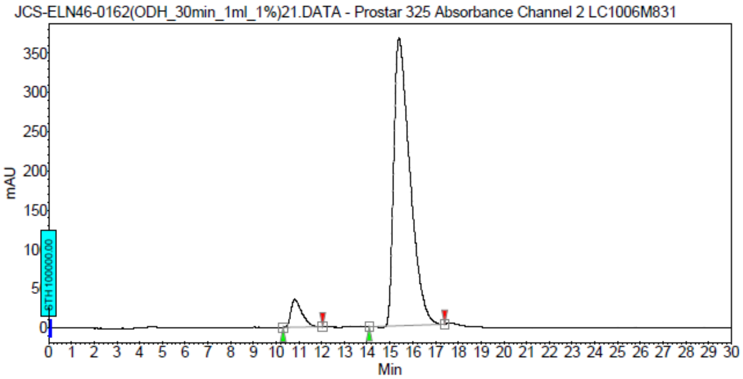

Peak results :

Index	Name	Time [Min]	Quantity [% Area]	Height [mAU]	Area [mAU.Min]	Area % [%]
1	UNKNOWN	10.82	6.24	35.7	20.1	6.239
2	UNKNOWN	15.40	93.76	367.4	301.3	93.761
Total			100.00	403.1	321.4	100.000

0 ℃, 1.0 mol % Rh_2(*S*-TPPTTL)$_4$ 1.5 equiv vinyl heterocycle, 3.5 equiv 2-chloropyridine, CH_2Cl_2 as solvent, 10 equiv HFIP, reaction performed according to procedure 3.5

Chromatogram :
JCS-ELN46-0186(ODH_1ML_1%_30MIN)70_chann el2

System : Prostar LC System
Method : ADH_30min_1mL_1%-230nm
User : User1

Acquired : 12/11/2019 11:59:57 PM
Processed : 12/12/2019 9:45:57 AM
Printed : 12/31/2019 12:04:34 PM

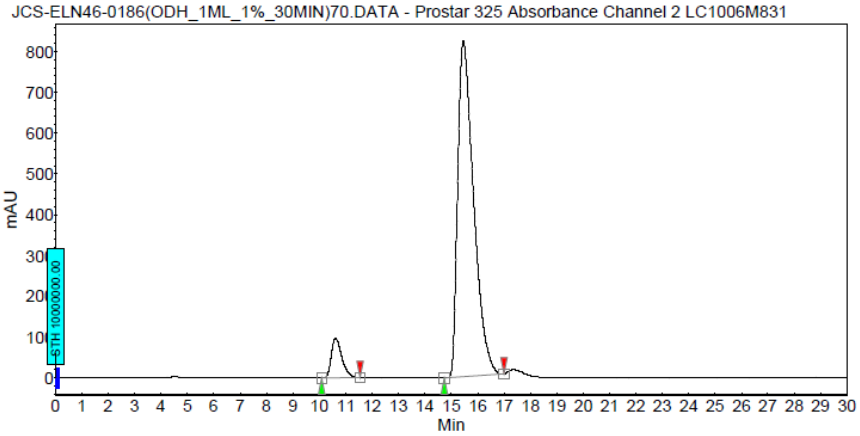

Peak results :

Index	Name	Time [Min]	Quantity [% Area]	Height [mAU]	Area [mAU.Min]	Area % [%]
1	UNKNOWN	10.61	7.21	97.1	44.4	7.209
2	UNKNOWN	15.46	92.79	821.4	571.9	92.791
Total			100.00	918.5	616.3	100.000

Racemic Trace:

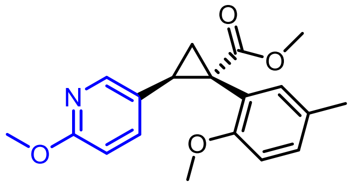

48

Chromatogram : JCS-ELN46-0103(ODH_30MIN_1ML_1%)16_channel2

System : Prostar LC System
Method : ADH_30min_1mL_1%-230nm
User : User1

Acquired : 10/17/2019 6:04:06 PM
Processed : 10/18/2019 8:04:05 AM
Printed : 10/23/2019 4:33:17 PM

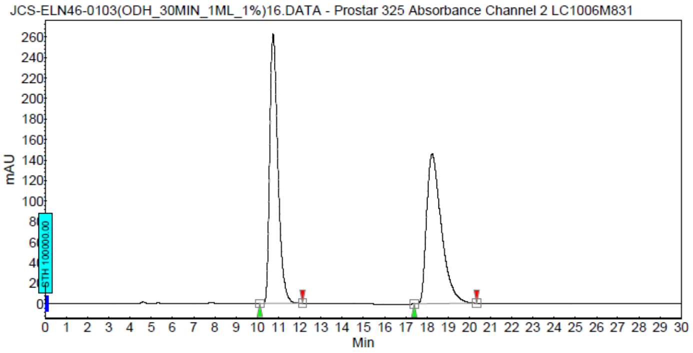

Peak results :

Index	Name	Time [Min]	Quantity [% Area]	Height [mAU]	Area [mAU.Min]	Area % [%]
1	UNKNOWN	10.74	49.79	262.2	117.0	49.792
2	UNKNOWN	18.24	50.21	145.9	118.0	50.208
Total			100.00	408.0	234.9	100.000

0 ℃, 1.0 mol % Rh_2(*S*-TPPTTL)$_4$, 1.5 equiv vinyl heterocycle, 3.5 equiv 2-chloropyridine, CH_2Cl_2 as solvent, reaction performed according to procedure 3.4

Chromatogram : JCS-ELN46-0106(ODH_30MIN_1ML_1%)25_channel2

System : Prostar LC System
Method : ADH_30min_1mL_1%-230nm
User : User1

Acquired : 10/17/2019 9:56:08 PM
Processed : 10/18/2019 8:05:47 AM
Printed : 12/31/2019 12:32:45 PM

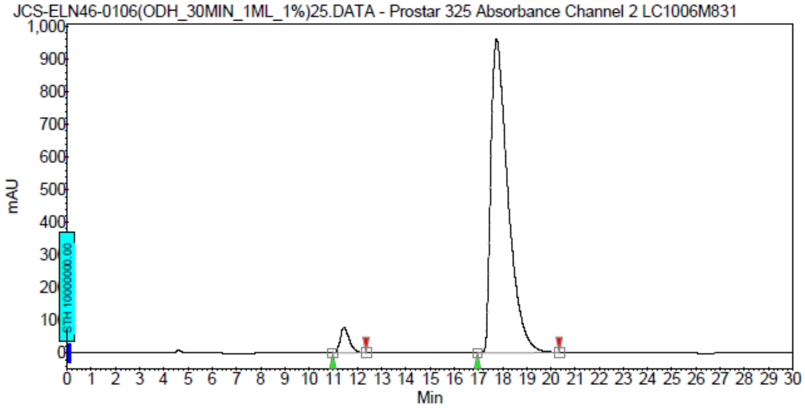

Peak results :

Index	Name	Time [Min]	Quantity [% Area]	Height [mAU]	Area [mAU.Min]	Area % [%]
1	UNKNOWN	11.45	4.11	77.2	33.7	4.106
2	UNKNOWN	17.75	95.89	961.0	787.3	95.894
Total			100.00	1038.2	821.0	100.000

0 ℃, 1.0 mol % Rh_2(*S*-TPPTTL)$_4$, 1.5 equiv vinyl heterocycle, 3.5 equiv 2-chloropyridine, CH_2Cl_2 as solvent, 10 equiv HFIP, reaction performed according to procedure 3.5

Chromatogram :
JCS-ELN46-0172(ODH_30MIN_1ML_1%)12_channel2

System : Prostar LC System
Method : ADH_30min_1mL_1%-230nm
User : User1

Acquired : 11/30/2019 6:11:24 PM
Processed : 12/2/2019 8:41:54 AM
Printed : 12/31/2019 12:15:28 PM

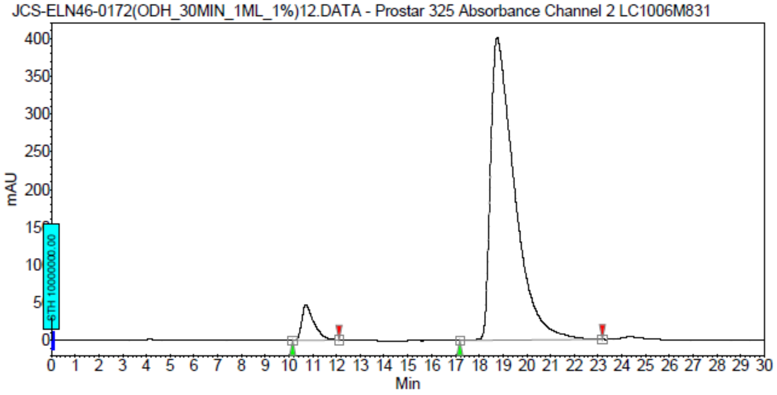

Peak results :

Index	Name	Time [Min]	Quantity [% Area]	Height [mAU]	Area [mAU.Min]	Area % [%]
1	UNKNOWN	10.71	5.61	47.2	27.8	5.609
2	UNKNOWN	18.76	94.39	400.5	468.1	94.391
Total			100.00	447.7	495.9	100.000

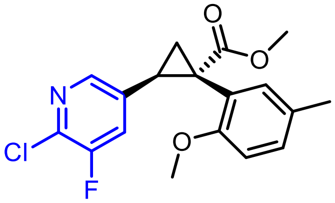

49

Racemic trace:

Chromatogram :
JCS-ELN46-0189RE(R,R-WHELK_60MIN_1ML_1%)3_channel2

System : Prostar LC System
Method : OD_30min_1mL_1%_230nm
User : User1

Acquired : 12/16/2019 9:41:58 AM
Processed : 12/16/2019 3:26:28 PM
Printed : 12/31/2019 12:59:07 PM

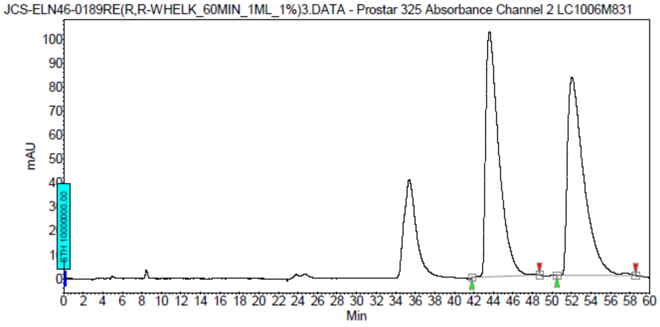

Peak results :

Index	Name	Time [Min]	Quantity [% Area]	Height [mAU]	Area [mAU.Min]	Area % [%]
1	UNKNOWN	43.60	49.93	102.4	160.2	49.933
2	UNKNOWN	52.05	50.07	82.8	160.7	50.067
Total			100.00	185.1	320.9	100.000

0 ℃, 1.0 mol % Rh_2(*S*-TPPTTL)$_4$ 1.5 equiv vinyl heterocycle, 3.5 equiv 2-chloropyridine, CH_2Cl_2 as solvent, reaction performed according to procedure 3.4

Chromatogram :
JCS-ELN46-0192(WHELK_60MIN_1ML_1%)16_channel2

[illegible]star LC System
Method : OD_30min_1mL_1%_230nm
User : User1

Acquired : 12/16/2019 5:09:25 PM
Processed : 12/16/2019 8:55:34 PM
Printed : 12/31/2019 12:59:13 PM

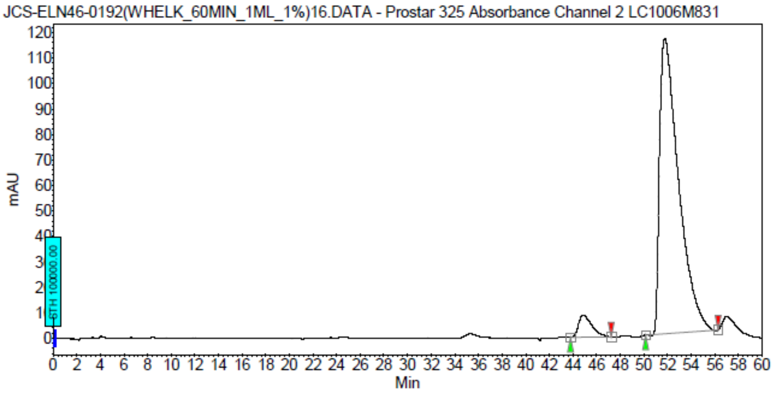

Peak results :

Index	Name	Time [Min]	Quantity [% Area]	Height [mAU]	Area [mAU.Min]	Area % [%]
1	UNKNOWN	44.88	5.04	8.7	11.5	5.037
2	UNKNOWN	51.77	94.96	115.9	216.7	94.963
Total			100.00	124.6	228.2	100.000

0 ℃, 1.0 mol % Rh_2(*S*-TPPTTL)$_4$ 1.5 equiv vinyl heterocycle, 3.5 equiv 2-chloropyridine, CH_2Cl_2 as solvent, 10 equiv HFIP, reaction performed according to procedure 3.5

Chromatogram :
JCS-ELN46-0198(WHELK_60MIN_1ML_1%)3_channel2

[illegible]rostar LC System
Method : OD_30min_1mL_1%_230nm
User : User1

Acquired : 12/20/2019 11:37:43 AM
Processed : 9/23/2020 5:58:12 PM
Printed : 9/23/2020 5:58:19 PM

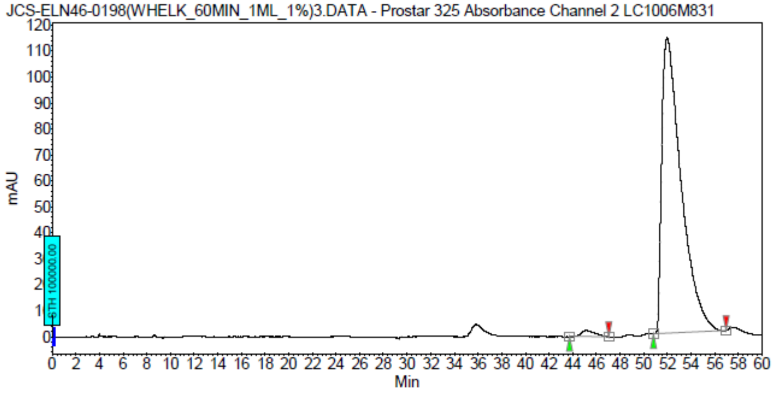

Peak results :

Index	Name	Time [Min]	Quantity [% Area]	Height [mAU]	Area [mAU.Min]	Area % [%]
1	UNKNOWN	45.17	1.43	2.4	3.1	1.434
2	UNKNOWN	51.97	98.57	114.0	216.4	98.566
Total			100.00	116.3	219.6	100.000

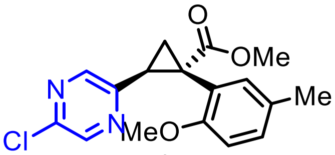

50

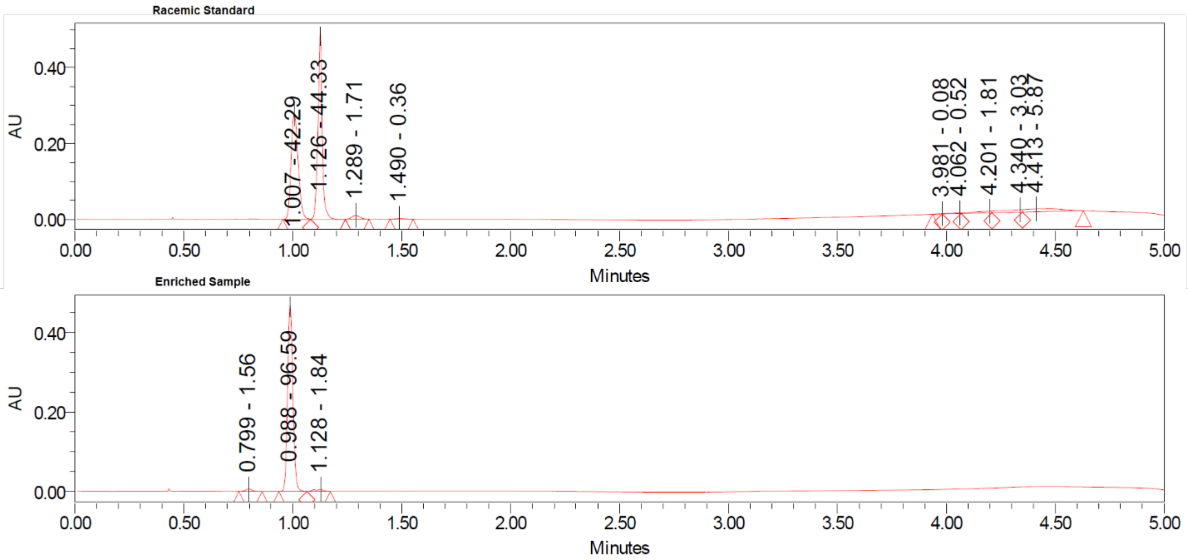

Racemic Standard	Enriched Sample
RT = 1.007 min<br>Area = 42.29	RT_{major} = 0.988 min<br>Area = 96.59
RT = 1.126 min<br>Area = 44.33	RT_{minor} = 1.128 min<br>Area = 1.84

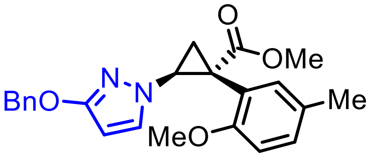

51

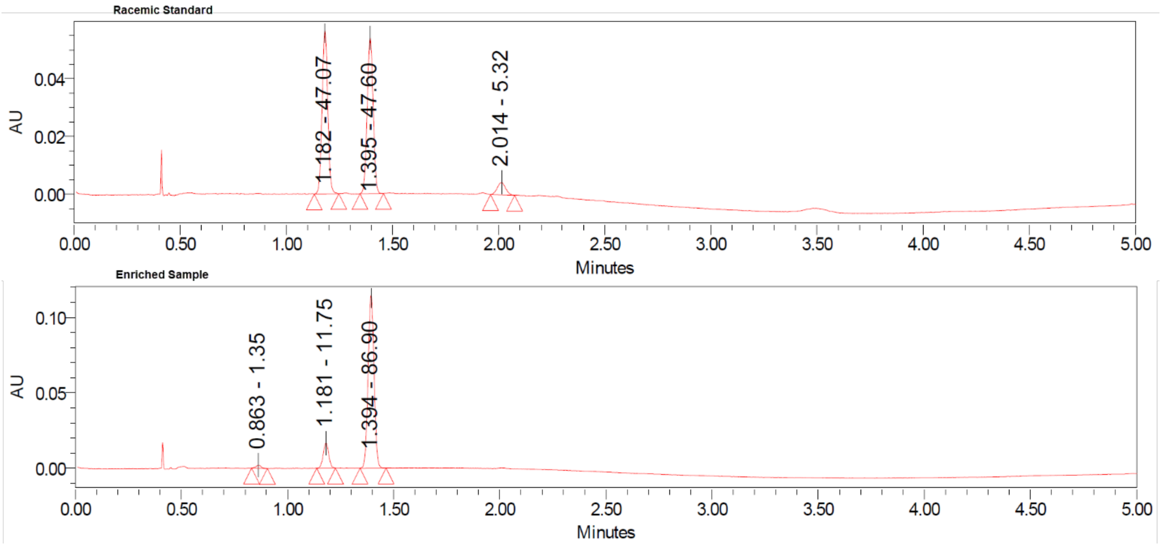

Racemic Standard	Enriched Sample
RT = 1.182 min<br>Area = 47.07	RT_{minor} = 1.181 min<br>Area = 11.75
RT = 1.395 min<br>Area = 47.60	RT_{major} = 1.394 min<br>Area = 86.90

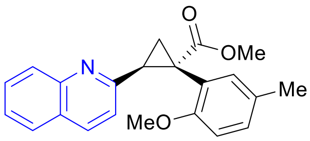

52

Racemic trace:

Chromatogram :
JCS-ELN46-0277conc(ADH_1ML_1%_60MIN)44_c
hannel2

[illegible]ar LC System
Method : OJ_30min_1mL_1%_230NM
User : User1

Acquired : 6/7/2020 4:59:26 AM
Processed : 9/12/2020 9:43:51 AM
Printed : 9/12/2020 9:43:58 AM

JCS-ELN46-0277conc(ADH_1ML_1%_60MIN)44.DATA - Prostar 325 Absorbance Channel 2 LC1006M831

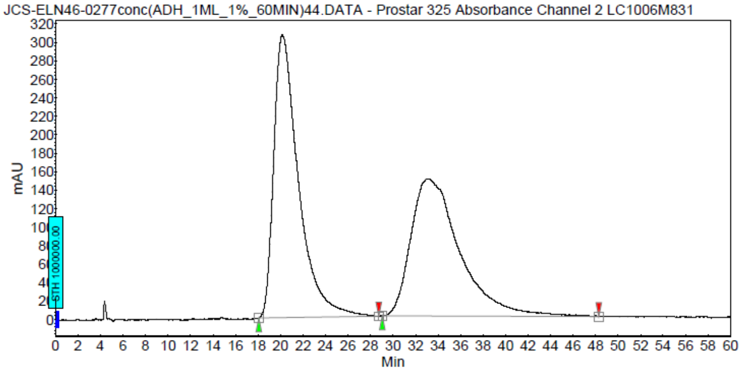

Peak results :

Index	Name	Time [Min]	Quantity [% Area]	Height [mAU]	Area [mAU.Min]	Area % [%]
1	UNKNOWN	20.15	51.09	306.0	780.3	51.090
2	UNKNOWN	33.06	48.91	148.7	747.0	48.910
Total			100.00	454.7	1527.2	100.000

0 ℃, 1.0 mol % Rh_2(*S*-TPPTTL)$_4$ 1.5 equiv vinyl heterocycle, 3.5 equiv 2-chloropyridine, CH_2Cl_2 as solvent, reaction performed according to procedure 3.4

Chromatogram :
JCS-ELN46-0278conc(ADH_1ml_1%_60min)5_channel2

[illegible]star LC System
Method : OJ_30min_1mL_1%_230NM
User : User1

Acquired : 6/9/2020 6:17:52 PM
Processed : 6/10/2020 4:48:38 PM
Printed : 9/12/2020 9:40:49 AM

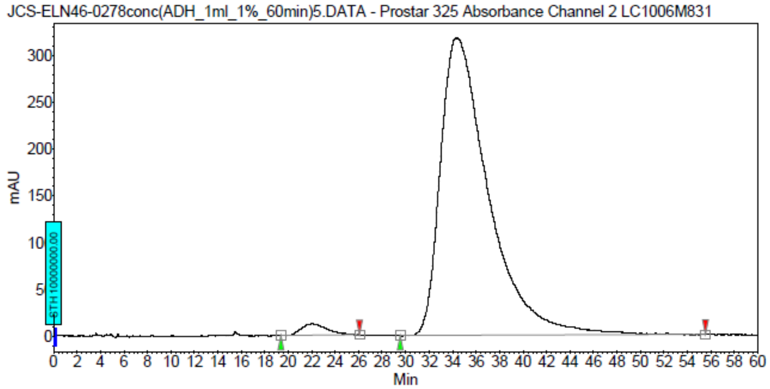

Peak results :

Index	Name	Time [Min]	Quantity [% Area]	Height [mAU]	Area [mAU.Min]	Area % [%]
1	UNKNOWN	22.04	2.03	12.5	31.3	2.034
2	UNKNOWN	34.36	97.97	317.8	1508.3	97.966
Total			100.00	330.3	1539.6	100.000

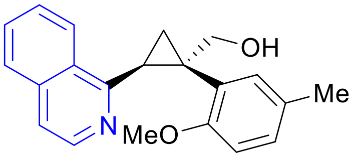

(53-reduced)

LAH reduction of **53** was required to achieve separation of the enantiomers by chiral UHPLC. See section 4.3 for experimental details.

Racemic trace of reduced product:

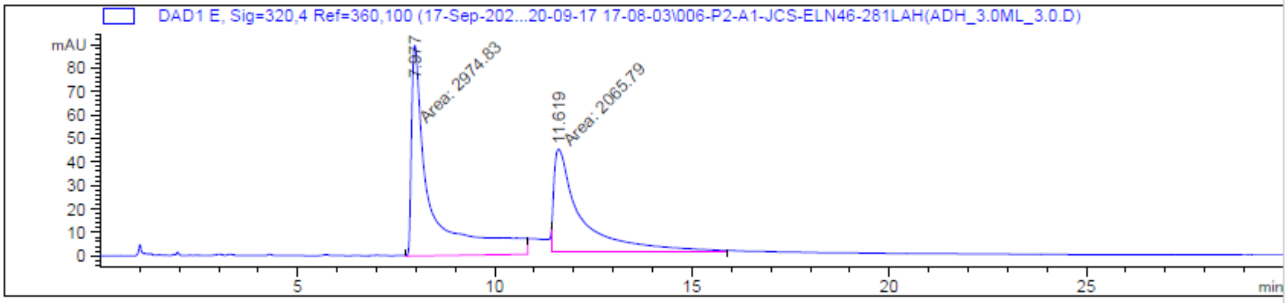


```
Signal 5: DAD1 E, Sig=320,4 Ref=360,100

Peak RetTime Type  Width     Area      Height     Area
  #   [min]        [min]   [mAU*s]     [mAU]        %
----|-------|----|-------|----------|----------|--------|
   1   7.977 MM    0.5532 2974.82690   89.62110  59.0172
   2  11.619 MM    0.7913 2065.78540   43.50774  40.9828

Totals :                  5040.61230  133.12885
```

0 ℃, 1.0 mol % Rh_2(*S*-TPPTTL)$_4$ 1.5 equiv vinyl heterocycle, 3.5 equiv 2-chloropyridine, CH_2Cl_2 as solvent, reaction performed according to procedure 3.4 prior to LAH reduction.

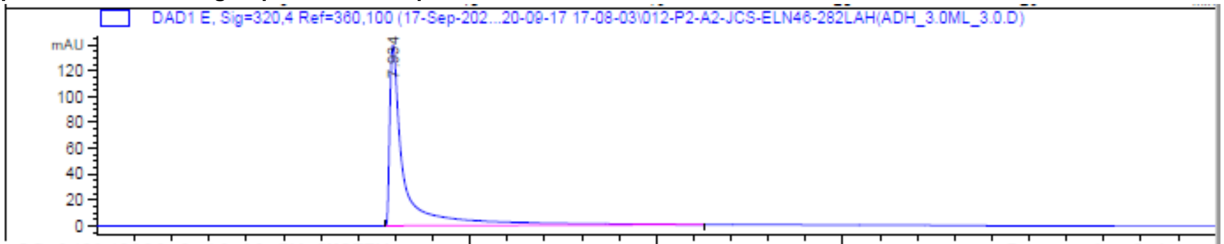

LC 9/21/2020 2:46:08 PM SYSTEM Page 1 of 4

Signal 5: DAD1 E, Sig=320,4 Ref=360,100

Peak #	RetTime [min]	Type	Width [min]	Area [mAU*s]	Height [mAU]	Area %
1	7.934	BB	0.3805	3865.99292	139.85376	100.0000

Totals : 3865.99292 139.85376

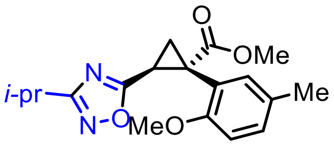

54

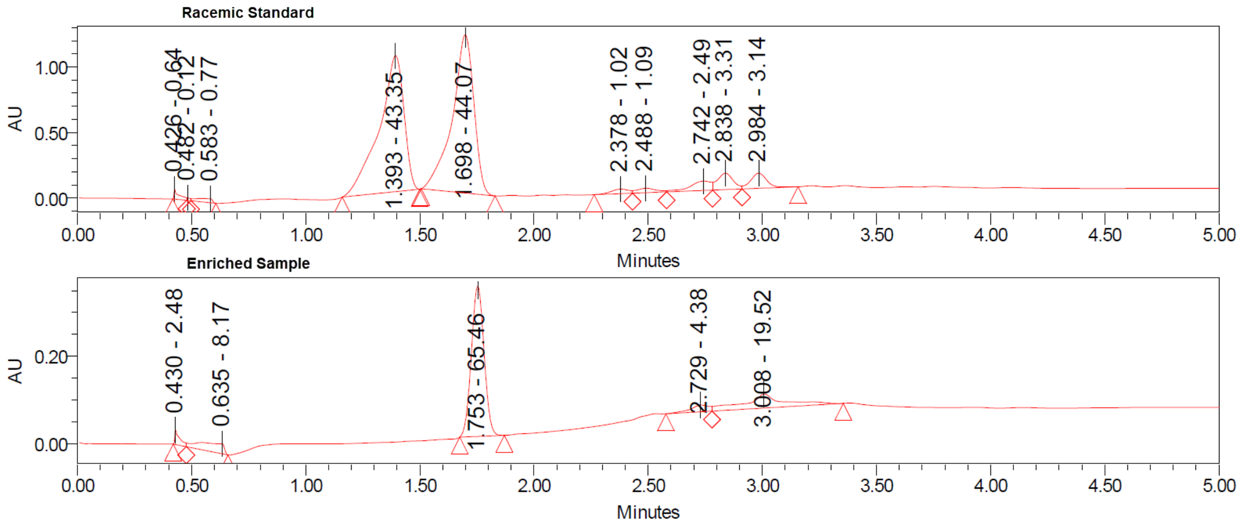

Racemic Standard	Enriched Sample
RT = 1.393 min<br>Area = 43.35	RT_{minor} = n.d.<br>Area = n.d.
RT = 1.698 min<br>Area = 44.07	RT_{major} = 1.753 min<br>Area = 65.46

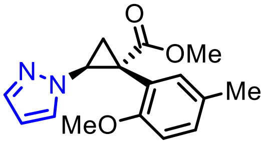

55

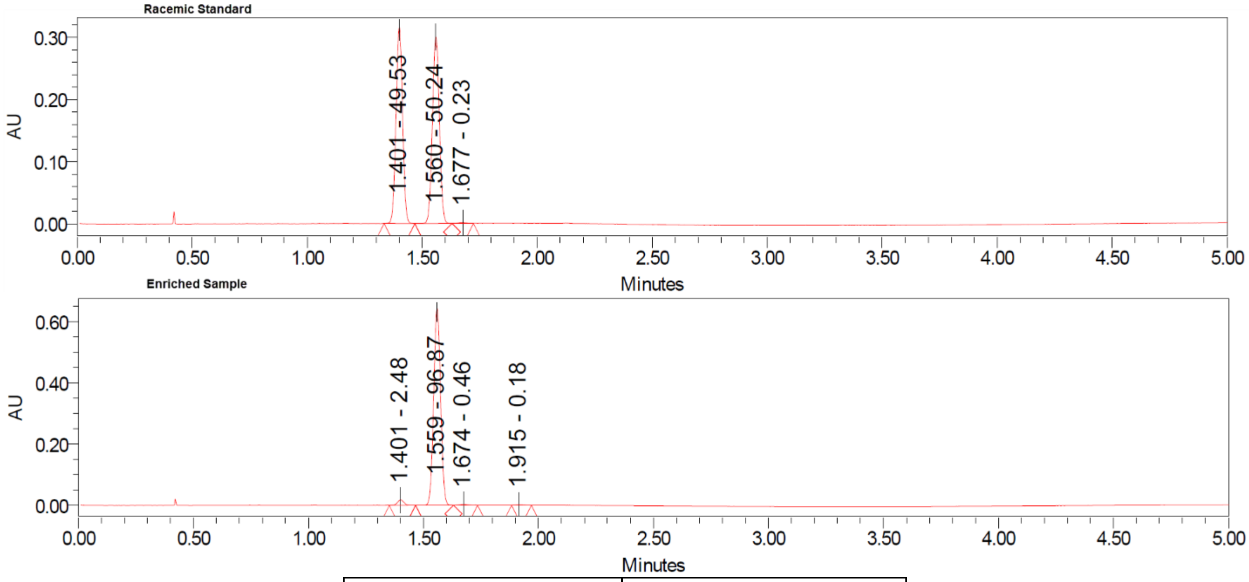

Racemic Standard	Enriched Sample
RT = 1.401 min<br>Area = 49.53	RT_{minor} = 1.401 min<br>Area = 2.48
RT = 1.560 min<br>Area = 50.24	RT_{major} = 1.559 min<br>Area = 96.87

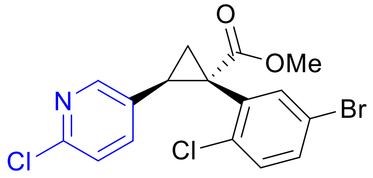

56

Racemic trace:

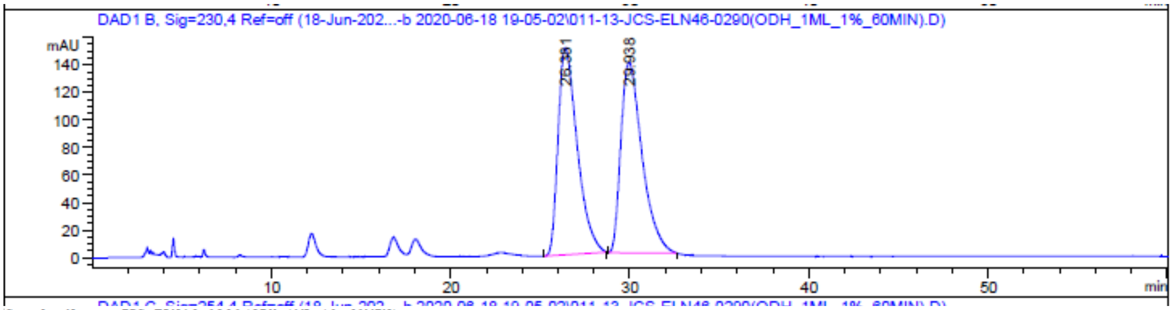

Sample Name: JCS-ELN46-0290(ODH_1ML_1%_60MIN)

Peak #	RetTime [min]	Type	Width [min]	Area [mAU*s]	Height [mAU]	Area %
1	26.381	VV R	0.8758	1.12299e4	150.62157	49.6462
2	29.938	VV R	0.9626	1.13900e4	138.47125	50.3538

Totals : 2.26198e4 289.09282

0 ℃, 1.0 mol % Rh_2(*S*-TPPTTL)$_4$ 1.5 equiv vinyl heterocycle, 3.5 equiv 2-chloropyridine, CH_2Cl_2 as solvent, reaction performed according to procedure 3.4

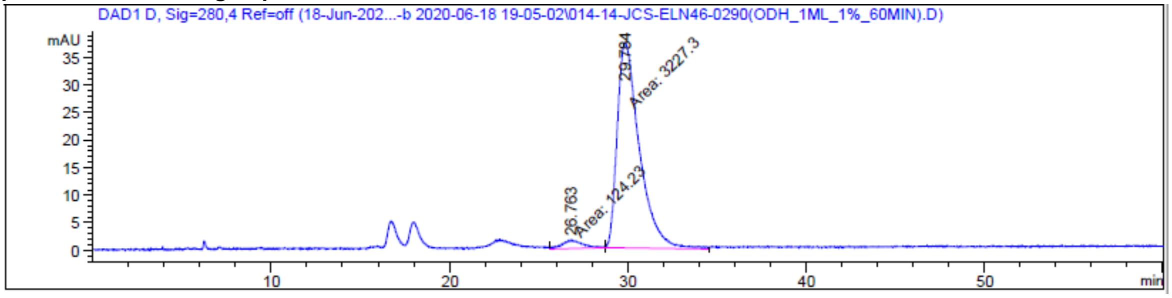

Signal 4: DAD1 D, Sig=280,4 Ref=off

Peak #	RetTime [min]	Type	Width [min]	Area [mAU*s]	Height [mAU]	Area %
1	26.763	MM	1.3216	124.23034	1.56664	3.7067
2	29.784	MM	1.4409	3227.30420	37.33059	96.2933
Totals :				3351.53454	38.89723	

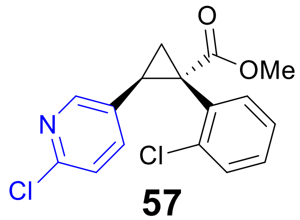

57

Racemic Trace:

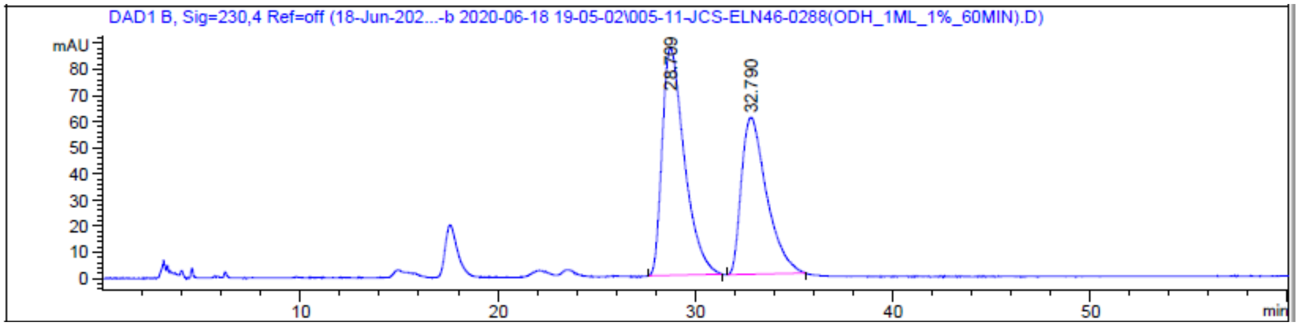

Signal 2: DAD1 B, Sig=230,4 Ref=off

Peak #	RetTime [min]	Type	Width [min]	Area [mAU*s]	Height [mAU]	Area %
1	28.709	VV R	0.9181	6819.27051	86.98462	56.0344
2	32.790	VV R	1.0426	5350.51172	60.01482	43.9656
Totals :				1.21698e4	146.99944	

0 ℃, 1.0 mol % Rh_2(*S*-TPPTTL)$_4$, 1.5 equiv vinyl heterocycle, 3.5 equiv 2-chloropyridine, CH_2Cl_2 as solvent, reaction performed according to procedure 3.4

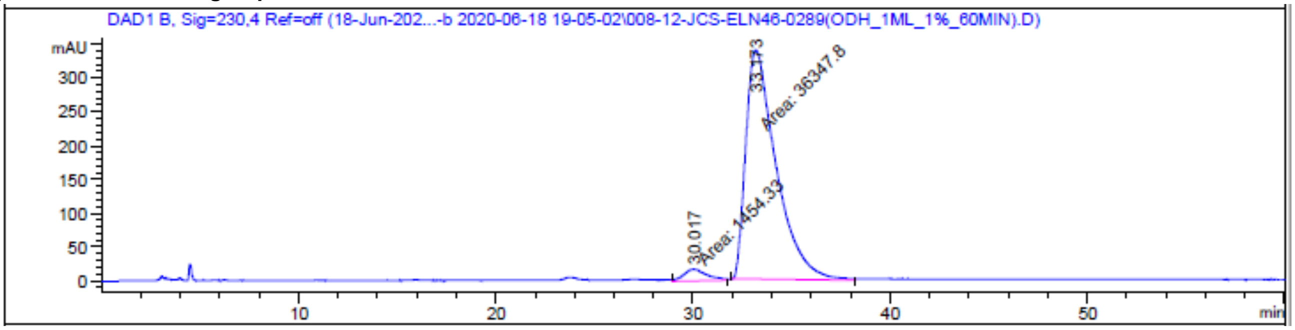


```
Signal 2: DAD1 B, Sig=230,4 Ref=off

Peak RetTime Type  Width     Area      Height     Area
  #   [min]        [min]   [mAU*s]     [mAU]       %
----|-------|----|-------|----------|----------|--------|
   1  30.017 MM    1.3712 1454.32605   17.67680   3.8472
   2  33.173 MM    1.7882 3.63478e4   338.76584  96.1528

Totals :                  3.78022e4   356.44263
```

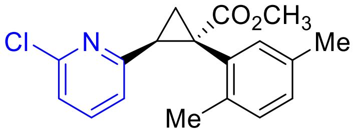

58

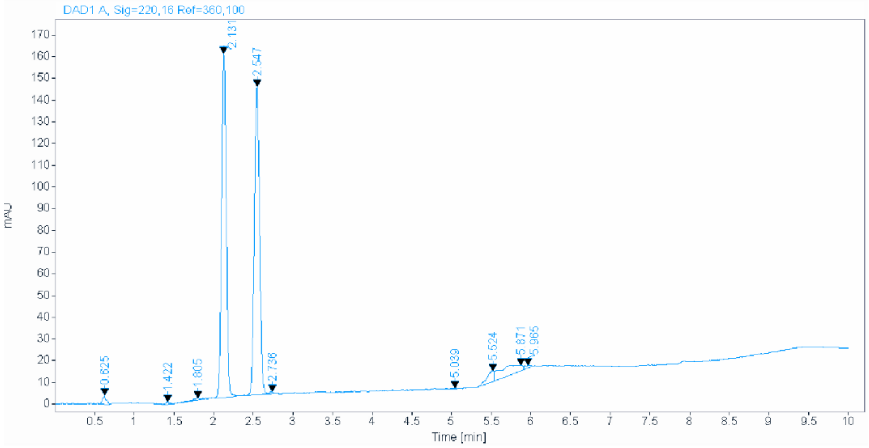

Racemic Standard
RT = 2.13 min<br>Area = 647.6
RT = 2.55 min<br>Area = 646.9

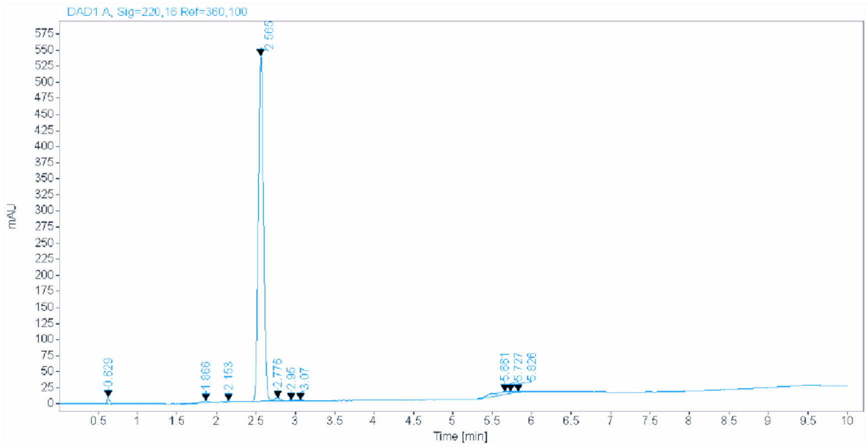

Enriched Sample
RT_{minor} = 2.15 min<br>Area = 5.4
RT_{major} = 2.57 min<br>Area = 2451.9

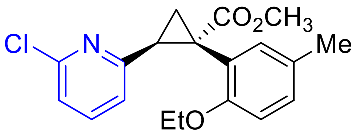

59

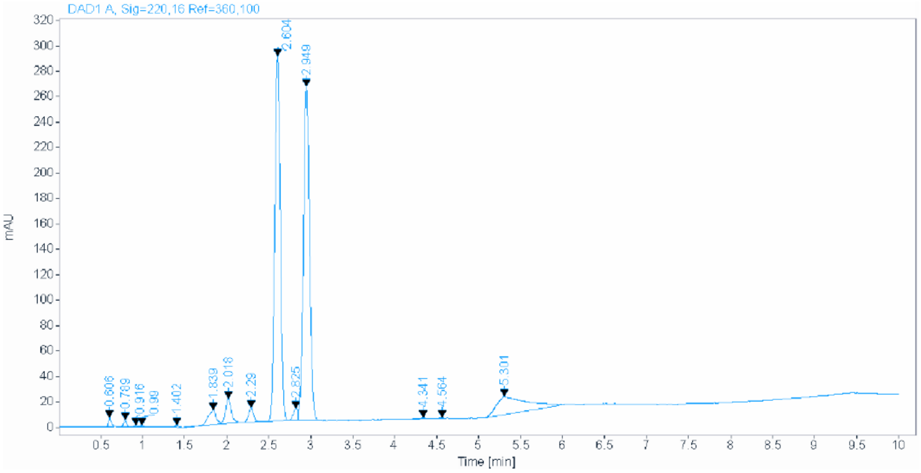

Racemic Standard
RT = 2.60 min
Area = 1318.7
RT = 2.95 min
Area = 1334.2

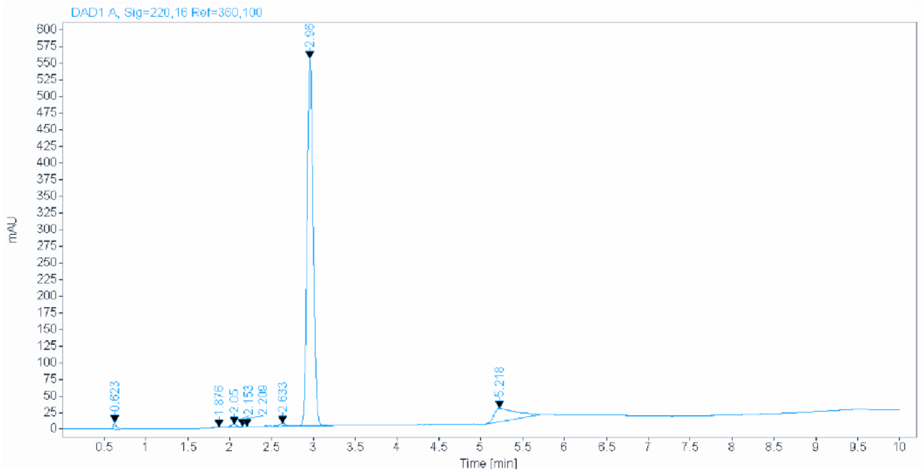

Enriched Sample
RT_{minor} = 2.63 min
Area = 19.7
RT_{major} = 2.96 min
Area = 2782.0

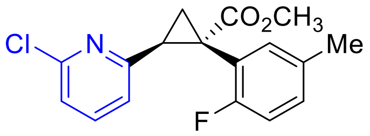

60

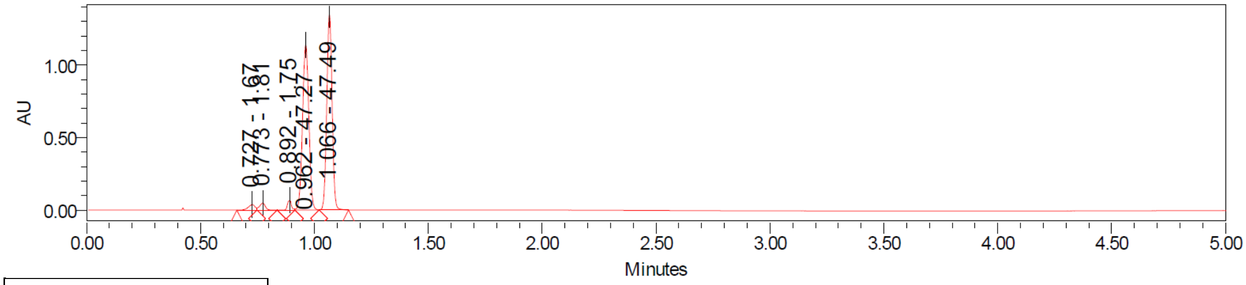

Racemic Standard
RT = 0.96 min<br>Area = 47.27
RT = 1.07 min<br>Area = 47.49

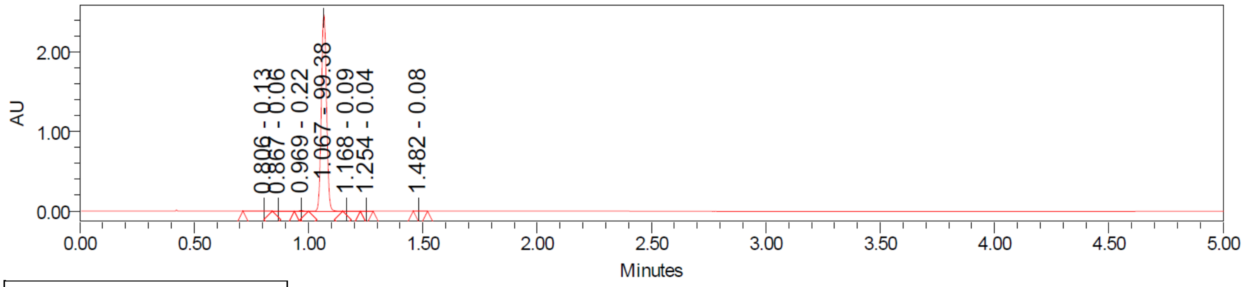

Enriched Sample
RT_{minor} = 0.97 min<br>Area = 0.22
RT_{major} = 1.07 min<br>Area = 99.38

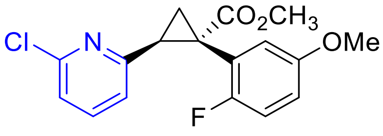

61

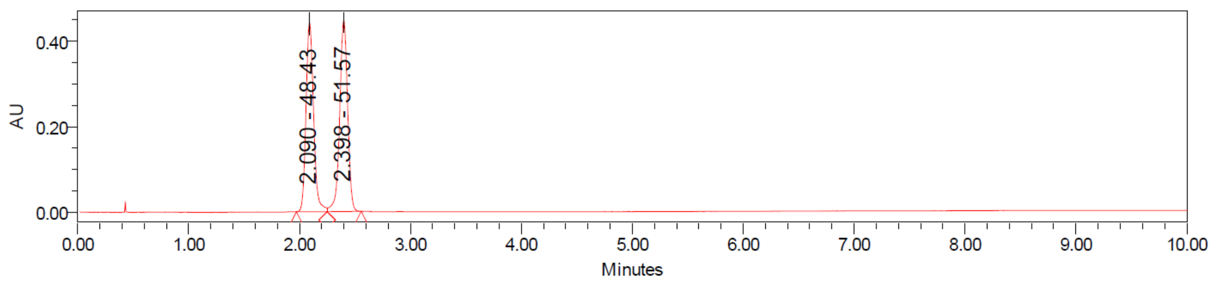

Racemic Standard
RT = 2.09 min<br>Area = 48.43
RT = 2.40 min<br>Area = 51.57

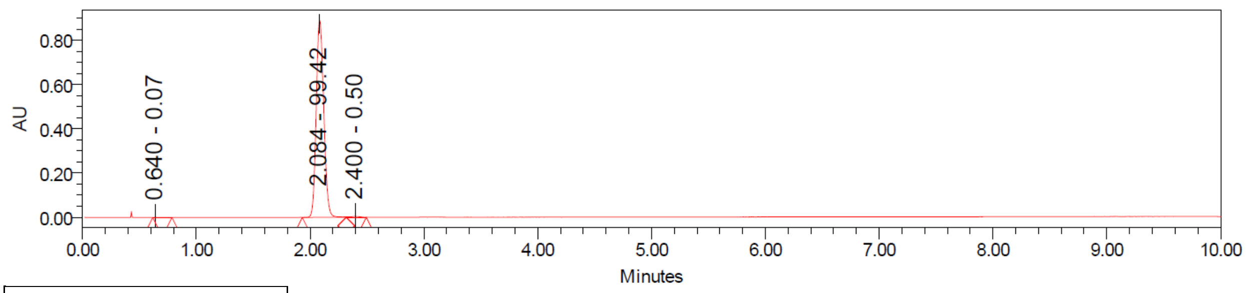

Enriched Sample
RT_{major} = 2.08 min<br>Area = 99.42
RT_{minor} = 2.40 min<br>Area = 0.50

Racemic trace:

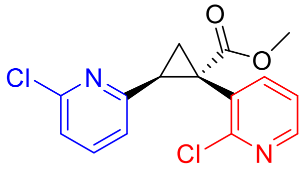

62

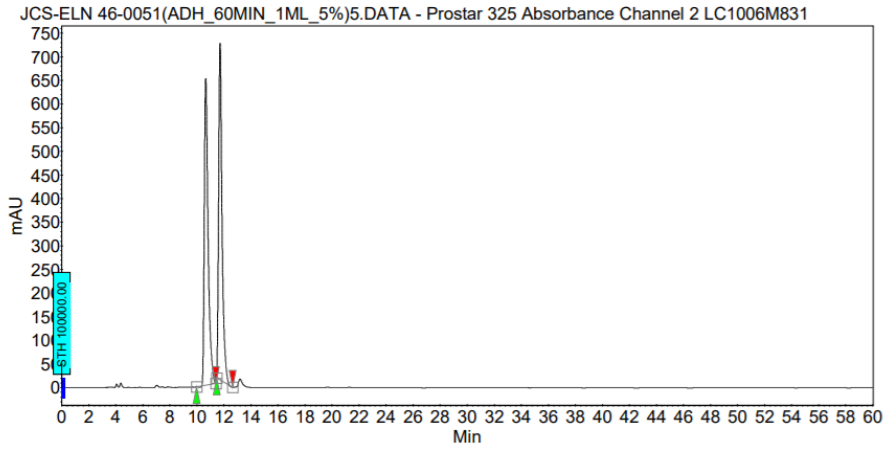

Peak results :

Index	Name	Time [Min]	Quantity [% Area]	Height [mAU]	Area [mAU.Min]	Area % [%]
1	UNKNOWN	10.65	50.25	649.3	210.6	50.254
2	UNKNOWN	11.71	49.75	712.3	208.5	49.746
Total			100.00	1361.7	419.1	100.000

0 ℃, 1.0 mol % Rh_2(*S*-TPPTTL)$_4$ 1.5 equiv vinyl heterocycle, 3.5 equiv 2-chloropyridine, CH_2Cl_2 as solvent, reaction performed according to procedure 3.4

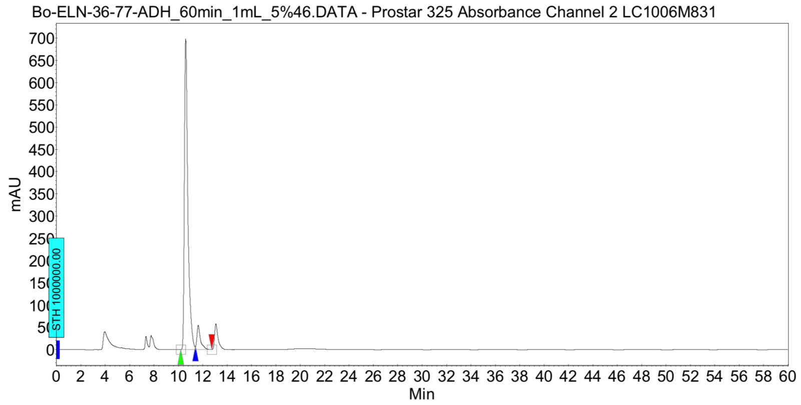

Peak results :

Index	Name	Time [Min]	Quantity [% Area]	Height [mAU]	Area [mAU.Min]	Area % [%]
1	UNKNOWN	10.60	92.62	698.2	230.9	92.622
2	UNKNOWN	11.63	7.38	54.6	18.4	7.378
Total			100.00	752.7	249.3	100.000

Racemic trace:

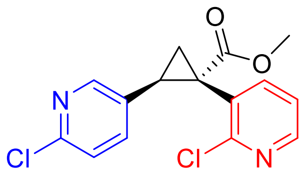

63

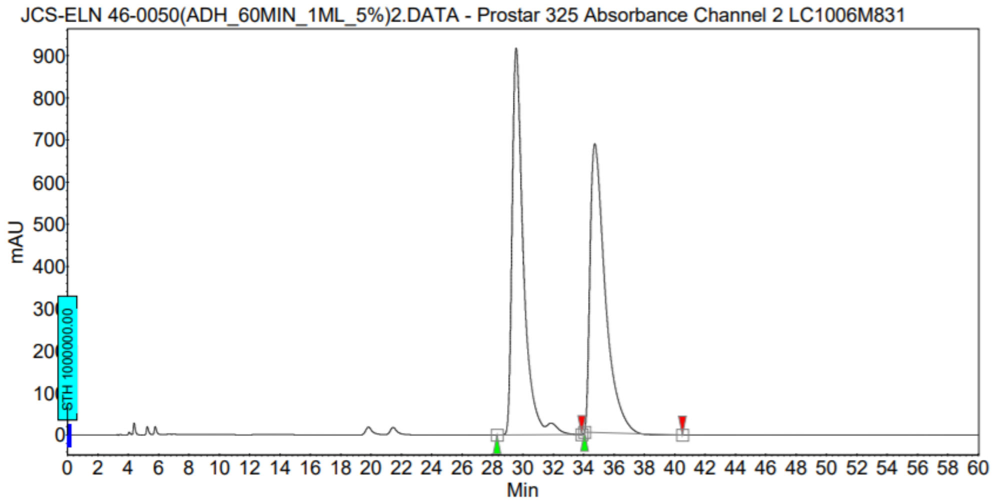

Peak results :

Index	Name	Time [Min]	Quantity [% Area]	Height [mAU]	Area [mAU.Min]	Area % [%]
1	UNKNOWN	29.56	50.43	917.3	801.0	50.427
2	UNKNOWN	34.74	49.57	684.2	787.4	49.573
Total			100.00	1601.4	1588.4	100.000

0 ℃, 1.0 mol % Rh_2(*S*-TPPTTL)$_4$1.5 equiv vinyl heterocycle, 3.5 equiv 2-chloropyridine, CH_2Cl_2 as solvent, reaction performed according to procedure 3.4

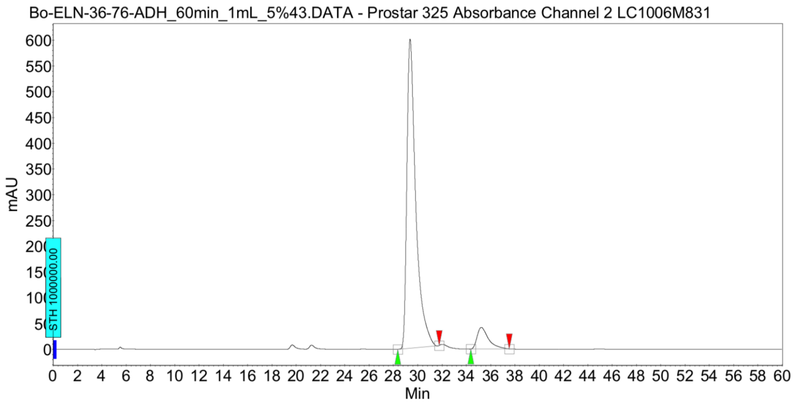

Peak results :

Index	Name	Time [Min]	Quantity [% Area]	Height [mAU]	Area [mAU.Min]	Area % [%]
2	UNKNOWN	29.37	91.81	599.4	508.8	91.813
1	UNKNOWN	35.23	8.19	41.9	45.4	8.187
Total			100.00	641.3	554.2	100.000

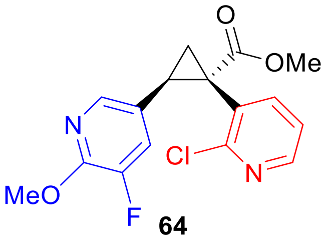

64

Racemic trace:

Chromatogram :
JCS-ELN46-202(ADH_80MIN_1ML_1%)3_channel1

System : Prostar LC System
Method : DACHDNB_30min_1mL_1%-230nm
User : User1

Acquired : 1/27/2020 9:42:16 AM
Processed : 1/30/2020 9:32:53 AM
Printed : 9/12/2020 12:07:06 PM

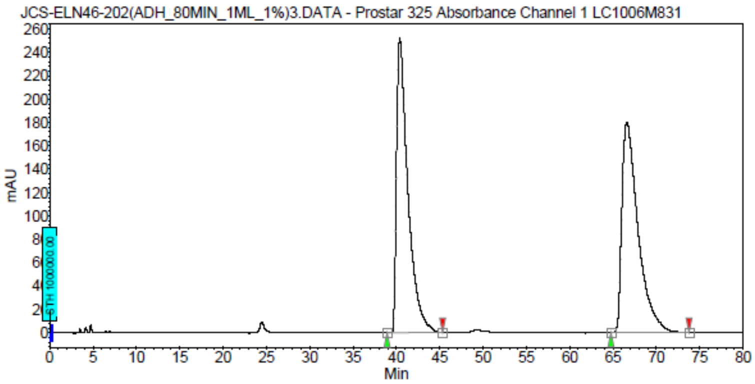

Peak results :

Index	Name	Time [Min]	Quantity [% Area]	Height [mAU]	Area [mAU.Min]	Area % [%]
1	UNKNOWN	40.39	49.63	252.0	384.1	49.626
2	UNKNOWN	66.59	50.37	180.0	389.9	50.374
Total			100.00	432.0	774.0	100.000

0 ℃, 1.0 mol % Rh_2(*S*-TPPTTL)$_4$ 1.5 equiv vinyl heterocycle, 3.5 equiv 2-chloropyridine, CH_2Cl_2 as solvent, reaction performed according to procedure 3.4

Chromatogram :
JCS-ELN46-203(ADH_80MIN_1ML_1%)6_channel1

System : Prostar LC System
Method : DACHDNB_30min_1mL_1%-230nm
User : User1

Acquired : 1/27/2020 11:52:55 AM
Processed : 1/27/2020 1:22:55 PM
Printed : 9/12/2020 12:06:16 PM

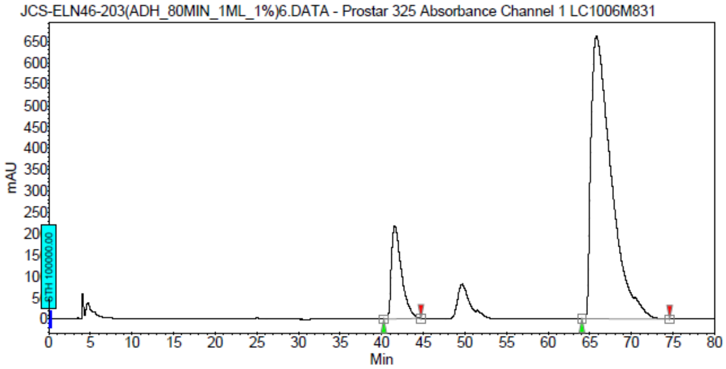

Peak results :

Index	Name	Time [Min]	Quantity [% Area]	Height [mAU]	Area [mAU.Min]	Area % [%]
1	UNKNOWN	41.57	14.09	218.8	306.3	14.092
2	UNKNOWN	65.80	85.91	661.4	1867.1	85.908
Total			100.00	880.2	2173.4	100.000

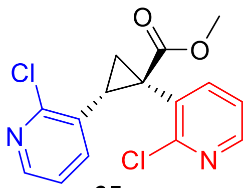

65

Racemic trace:

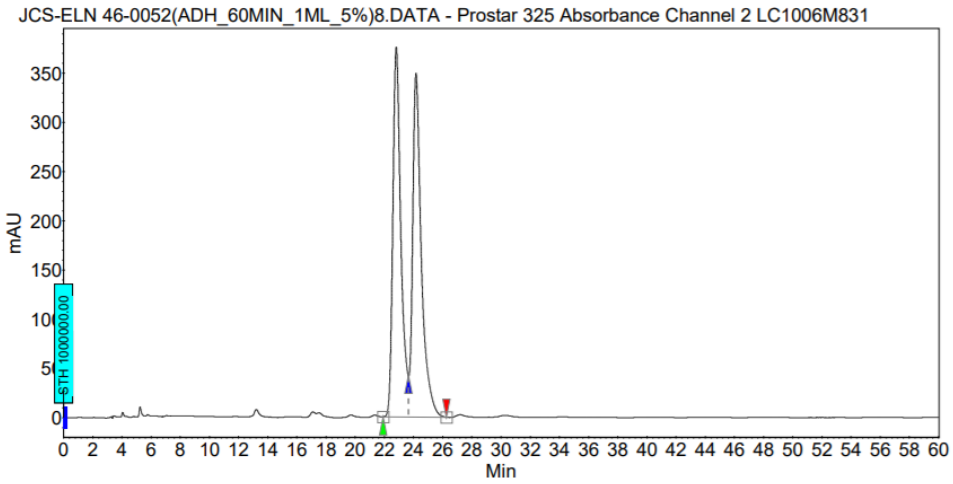

Peak results :

Index	Name	Time [Min]	Quantity [% Area]	Height [mAU]	Area [mAU.Min]	Area % [%]
1	UNKNOWN	22.81	49.64	375.9	239.1	49.641
2	UNKNOWN	24.16	50.36	349.3	242.6	50.359
Total			100.00	725.2	481.8	100.000

0 ℃, 1.0 mol % Rh_2(*S*-TPPTTL)$_4$ 1.5 equiv vinyl heterocycle, 3.5 equiv 2-chloropyridine, CH_2Cl_2 as solvent, reaction performed according to procedure 3.4

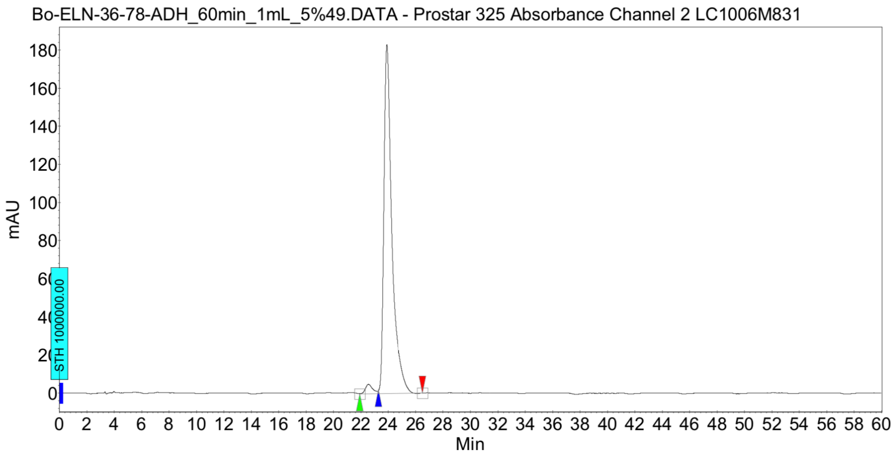

Peak results :

Index	Name	Time [Min]	Quantity [% Area]	Height [mAU]	Area [mAU.Min]	Area % [%]
1	UNKNOWN	22.54	2.40	4.9	3.1	2.396
2	UNKNOWN	23.89	97.60	183.1	124.9	97.604
Total			100.00	188.0	127.9	100.000

5.3: Screening of different coordinating additives for optimal %ee enhancement in cyclopropanation with *ortho*-substituted aryl/heteroaryl diazoacetates.

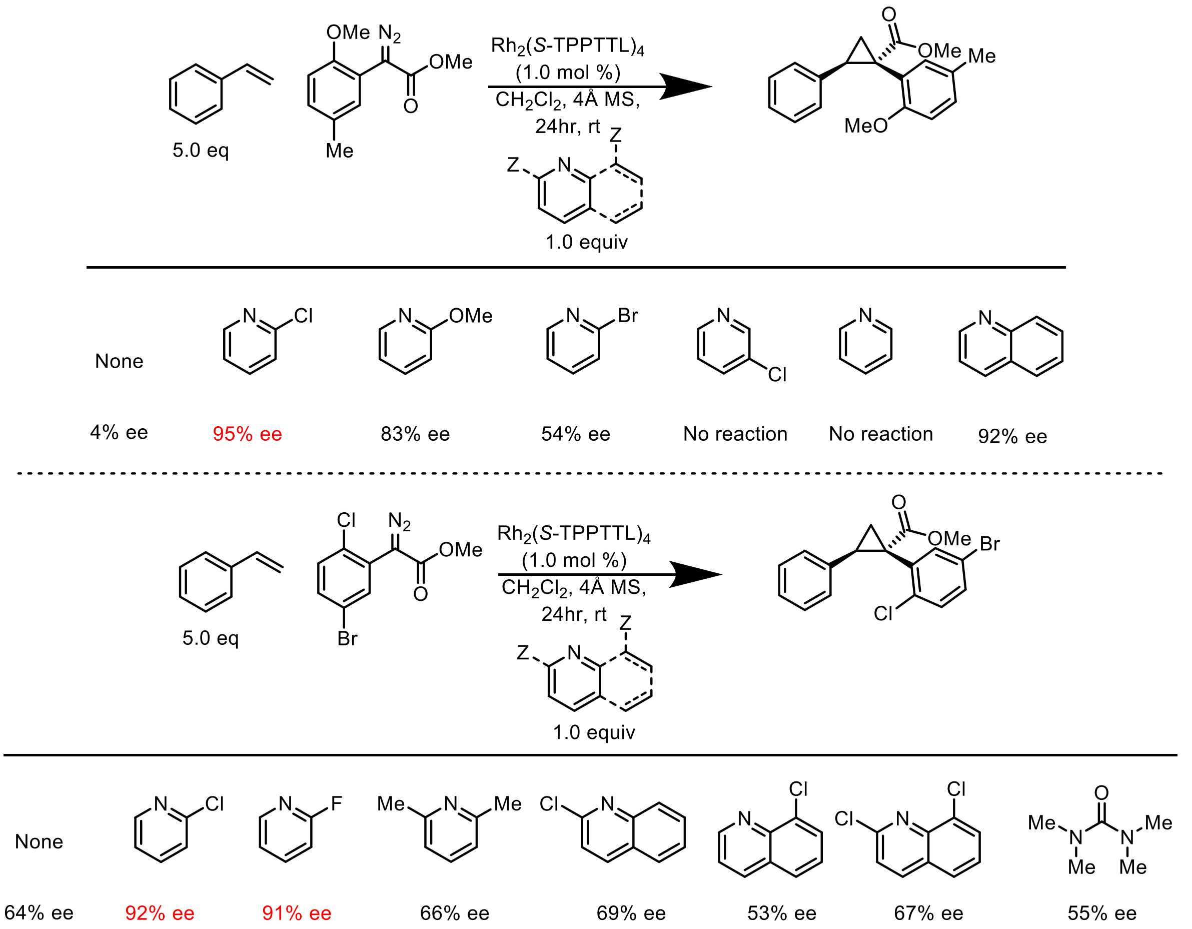

Figure S5. Examination of various coordinating additives to determine the optimal compound to enhance the enantioselectivity of cyclopropanation involving *ortho*-substituted aryl diazo acetates. Two aryldiazoacetates were examined to ensure generalizability. Reactions were conducted on 0.20 mmol scale with 5.0 equiv styrene, 1.0 equiv coordinating additive, 1.0 mol % catalyst loading, and CH_2Cl_2 as solvent. The reaction was conducted at room temperature and run for at least 13 hr. Of the additives tested, 2-chloropyridine and 2-fluoropyridine (red) gave the best levels of enantio-enhancement. Other additives seemed to hamper the enantioselectivity of the reaction, in particular substituted quinolines and tetra-methyl urea. 3-Chloropyridine and pyridine served to poison the reaction, no rhodium-carbene was generated and the diazo-starting material was recovered.

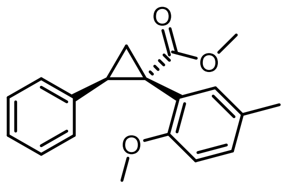

40

Racemic trace, AD-H column:

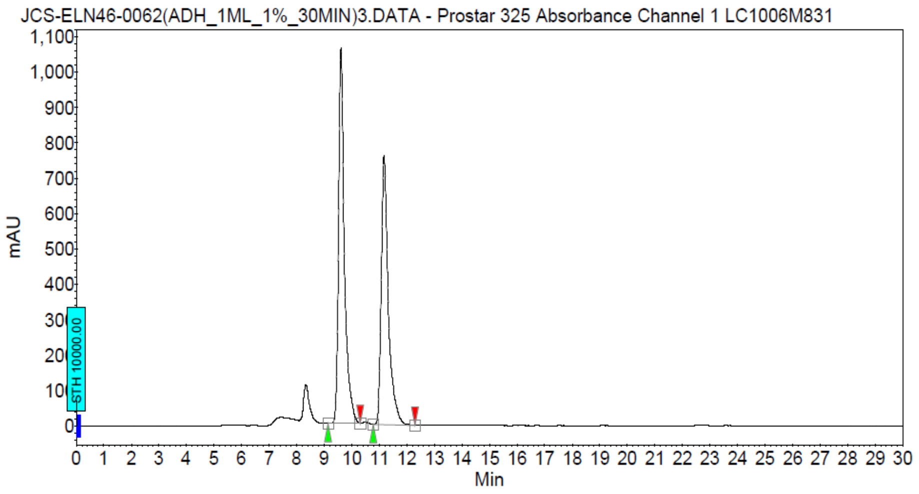

Peak results :

Index	Name	Time [Min]	Quantity [% Area]	Height [mAU]	Area [mAU.Min]	Area % [%]
1	UNKNOWN	9.61	53.67	1060.1	260.5	53.666
2	UNKNOWN	11.17	46.33	760.0	224.9	46.334
Total			100.00	1820.2	485.3	100.000

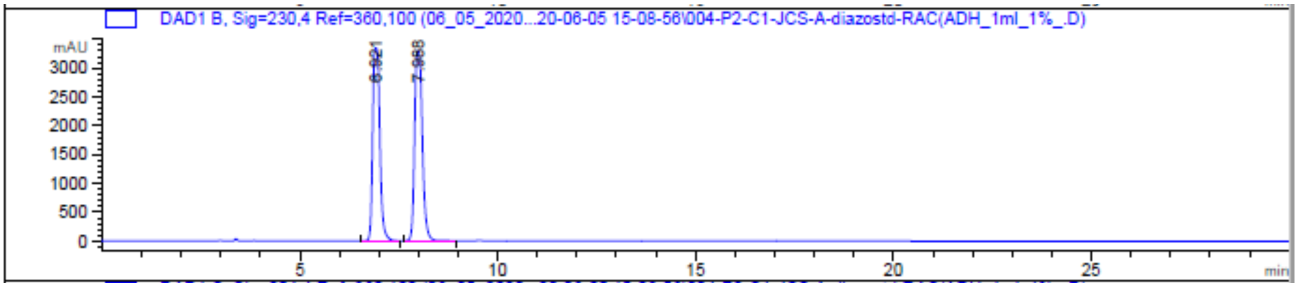

Signal 2: DAD1 B, Sig=230,4 Ref=360,100

Peak #	RetTime [min]	Type	Width [min]	Area [mAU*s]	Height [mAU]	Area %
1	6.921	BB	0.1986	4.20731e4	3348.93286	48.2033
2	7.988	BV R	0.2130	4.52095e4	3305.63135	51.7967

Totals : 8.72826e4 6654.56421

0 ℃, 1.0 mol % Rh_2(*S*-TPPTTL)$_4$, 5.0 equiv styrene, 1.0 equiv 2-chloropyridine, CH_2Cl_2 as solvent, reaction performed according to procedure 3.4

Chromatogram : JCS-ELN46-0095DIL(ADH_30MIN_1ml_1%)3_channel1

System : Prostar LC System
Method : DACHDNB_30min_1mL_1%-230nm
User : User1

Acquired : 10/17/2019 9:55:29 AM
Processed : 10/17/2019 10:44:45 AM
Printed : 12/31/2019 12:32:50 PM

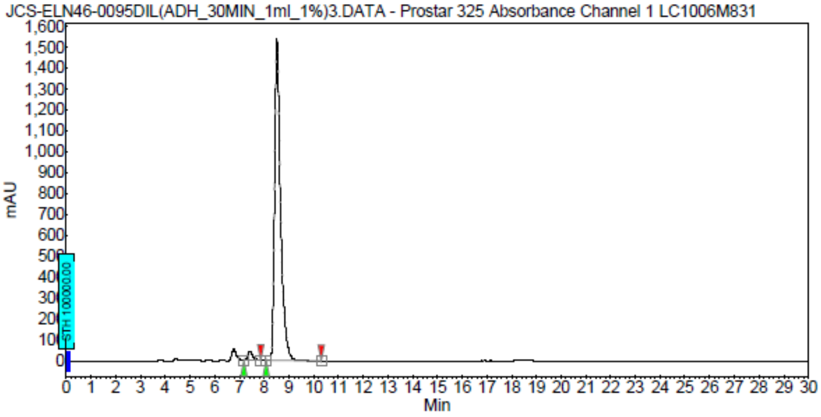

Peak results :

Index	Name	Time [Min]	Quantity [% Area]	Height [mAU]	Area [mAU.Min]	Area % [%]
2	UNKNOWN	7.41	2.41	44.3	10.6	2.413
1	UNKNOWN	8.52	97.59	1536.4	429.5	97.587
Total			100.00	1580.6	440.1	100.000

0 ℃, 1.0 mol % Rh_2(*S*-TPPTTL)$_4$, 5.0 equiv styrene, 1.0 equiv 2-MeOpyridine, CH_2Cl_2 as solvent, reaction performed according to procedure 3.4

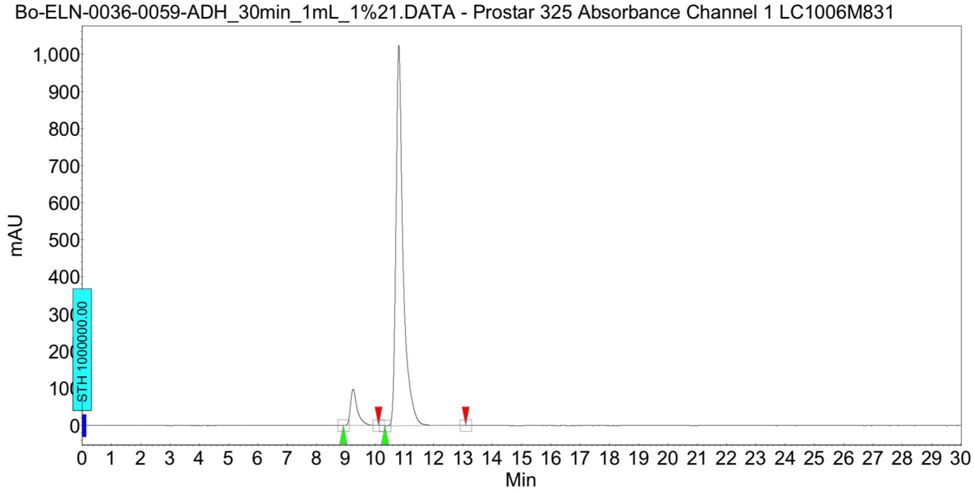

Peak results :

Index	Name	Time [Min]	Quantity [% Area]	Height [mAU]	Area [mAU.Min]	Area % [%]
2	UNKNOWN	9.25	8.27	97.7	26.3	8.274
1	UNKNOWN	10.81	91.73	1025.7	291.2	91.726
Total			100.00	1123.4	317.5	100.000

0 ℃, 1.0 mol % Rh_2(*S*-TPPTTL)$_4$, 5.0 equiv styrene, 1.0 equiv 2-Brpyridine, CH_2Cl_2 as solvent, reaction performed according to procedure 3.4

Chromatogram :
JCS-ELN46-0100(ADH_30MIN_1ml_1%)8_channel1

System : Prostar LC System
Method : DACHDNB_30min_1mL_1%-230nm
User : User1

Acquired : 10/16/2019 7:22:07 PM
Processed : 10/17/2019 8:59:27 AM
Printed : 12/31/2019 12:34:44 PM

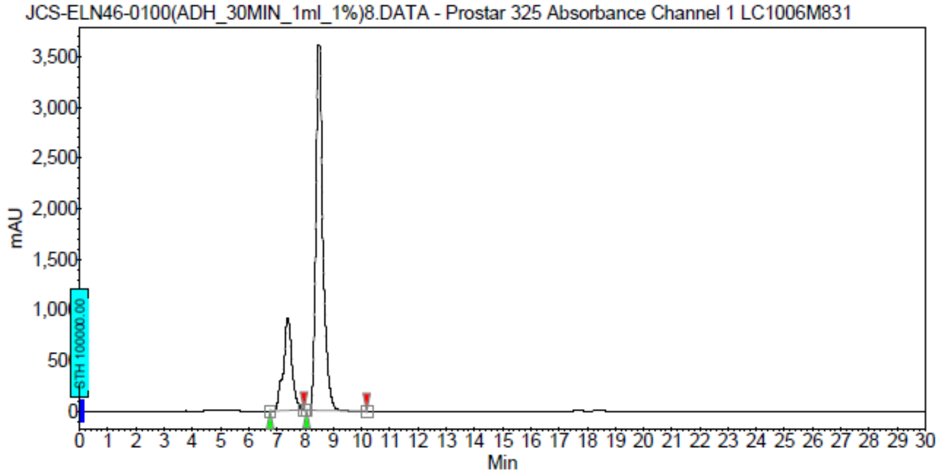

Peak results :

Index	Name	Time [Min]	Quantity [% Area]	Height [mAU]	Area [mAU.Min]	Area % [%]
2	UNKNOWN	7.38	23.23	916.7	323.5	23.230
1	UNKNOWN	8.50	76.77	3610.0	1069.1	76.770
Total			100.00	4526.7	1392.6	100.000

0 ℃, 1.0 mol % Rh_2(*S*-TPPTTL)$_4$, 5.0 equiv styrene, 1.0 equiv quinoline, CH_2Cl_2 as solvent, reaction performed according to procedure 3.4

Chromatogram :
JCS-ELN46-0102(ADH_30MIN_1ml_1%)11_channel1

System : Prostar LC System
Method : DACHDNB_30min_1mL_1%-230nm
User : User1

Acquired : 10/16/2019 8:39:23 PM
Processed : 10/17/2019 8:58:20 AM
Printed : 1/3/2020 11:11:40 AM

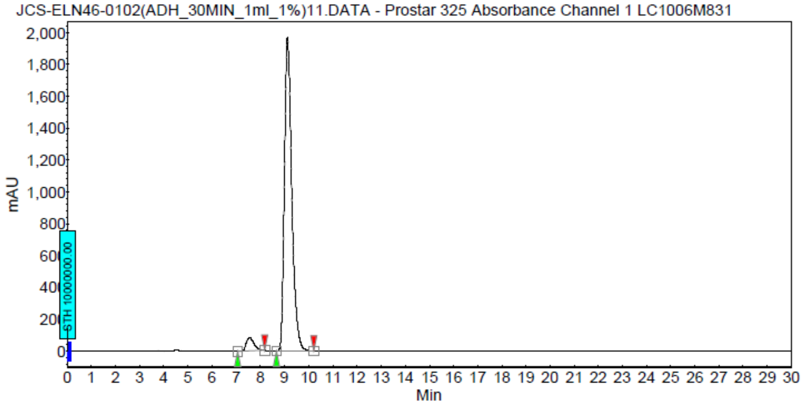

Peak results :

Index	Name	Time [Min]	Quantity [% Area]	Height [mAU]	Area [mAU.Min]	Area % [%]
1	UNKNOWN	7.54	4.24	81.7	30.5	4.245
2	UNKNOWN	9.12	95.76	1966.1	687.8	95.755
Total			100.00	2047.8	718.3	100.000

Racemic trace:

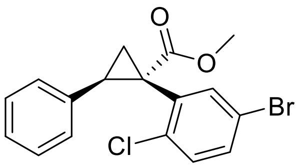

42

Chromatogram :
JCS-ELN46-0080(OJH_1ML_1%_30MIN)11_chann
el2

[illegible]m : Prostar LC System
Method : OD_30min_1mL_1%_210_230nm
User : User1

Acquired : 10/8/2019 4:32:30 PM
Processed : 10/8/2019 5:19:04 PM
Printed : 12/31/2019 12:44:41 PM

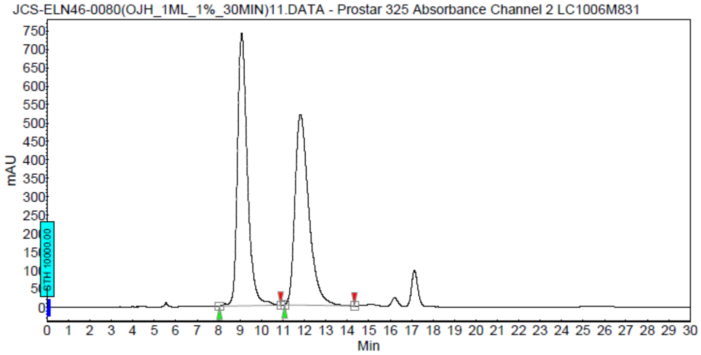

Peak results :

Index	Name	Time [Min]	Quantity [% Area]	Height [mAU]	Area [mAU.Min]	Area % [%]
1	UNKNOWN	9.07	50.51	739.1	394.0	50.510
2	UNKNOWN	11.82	49.49	516.8	386.0	49.490
Total			100.00	1255.9	780.0	100.000

0 ℃, 1.0 mol % Rh_2(*S*-TPPTTL)$_4$, 5.0 equiv styrene, 1.0 equiv 2-Clpyridine, CH_2Cl_2 as solvent, reaction performed according to procedure 3.4

Chromatogram :
JCS-ELN46-0079DIL(OJH_30MIN_1ML_1%)3_cha
nnel2

[illegible]rostar LC System
Method : OD_30min_1mL_1%_230nm
User : User1

Acquired : 10/9/2019 9:44:19 AM
Processed : 12/31/2019 12:43:26 PM
Printed : 12/31/2019 12:43:28 PM

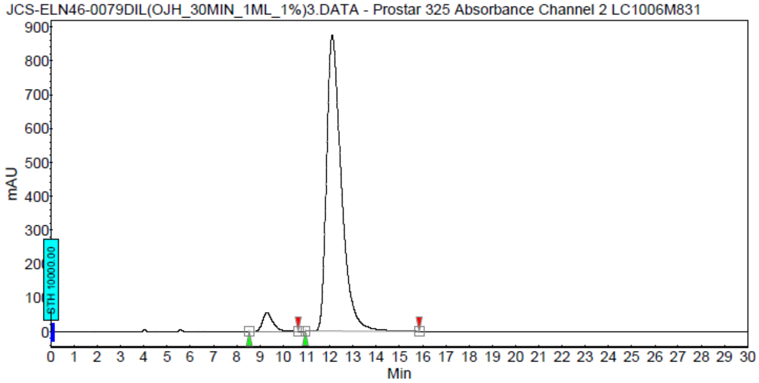

Peak results :

Index	Name	Time [Min]	Quantity [% Area]	Height [mAU]	Area [mAU.Min]	Area % [%]
1	UNKNOWN	9.30	4.30	55.7	29.0	4.303
2	UNKNOWN	12.11	95.70	872.6	643.9	95.697
Total			100.00	928.3	672.8	100.000

0 ℃, 1.0 mol % Rh_2(*S*-TPPTTL)$_4$, 5.0 equiv styrene, 1.0 equiv 2-Clquinoline, CH_2Cl_2 as solvent, reaction performed according to procedure 3.4

Chromatogram : JCS-ELN46-0115RE(OJH_30MIN_1ML_1%)23_channel2

System : Prostar LC System
Method : S4900_30min_1mL_1%_230nm
User : User1

Acquired : 11/10/2019 12:14:54 AM
Processed : 9/29/2020 4:40:17 PM
Printed : 9/29/2020 4:40:29 PM

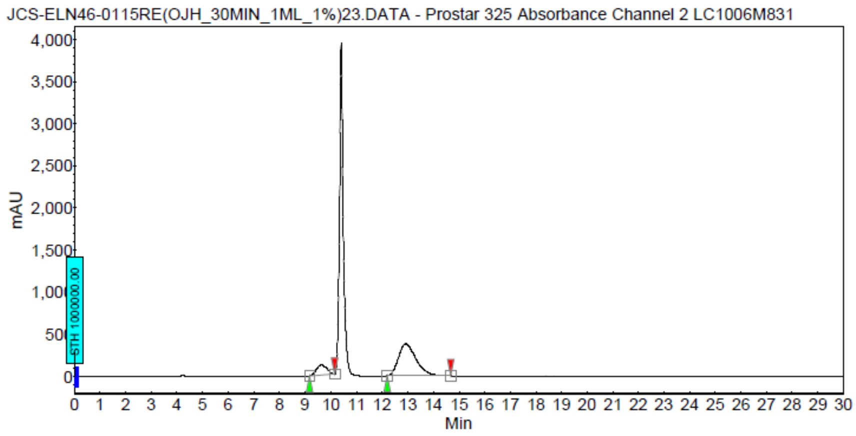

Peak results :

Index	Name	Time [Min]	Quantity [% Area]	Height [mAU]	Area [mAU.Min]	Area % [%]
1	UNKNOWN	9.62	15.41	120.3	55.1	15.411
2	UNKNOWN	12.92	84.59	379.7	302.3	84.589
Total			100.00	500.0	357.4	100.000

0 ℃, 1.0 mol % Rh_2(*S*-TPPTTL)$_4$, 5.0 equiv styrene, 1.0 equiv 2,8-dichloroquinoline, CH_2Cl_2 as solvent, reaction performed according to procedure 3.4

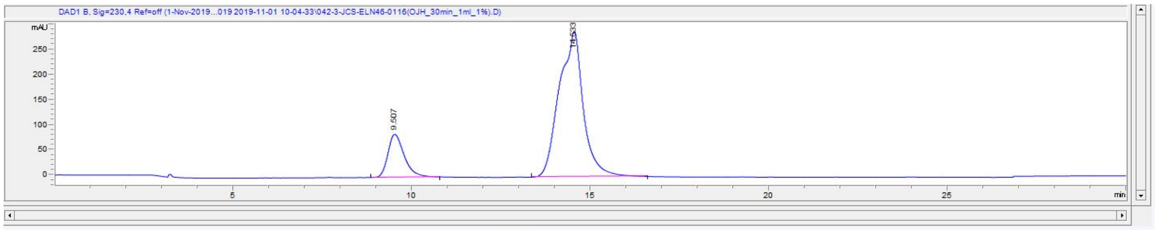

File Information

LC-File	042-3-JCS-ELN46-0116(OJH_30min_1ml_1%).D
File Path	C:\Chem32\1\Data\1-Nov-2019\31-OCT-2019 2019-11-01 10-04-3:
Date	02-Nov-19, 02:40:13
Sample	JCS-ELN46-0116(OJH_30min_1ml_1%)
Sample Info	
Barcode	
Operator	SYSTEM

#	Time	Type	Area	Height	Width	Area%	Symmetry
1	9.507	BB	2743.8	86.2	0.3758	16.745	0.698
2	14.533	BV R	13642.1	291.1	0.6067	83.255	1.512

0 ℃, 1.0 mol % Rh_2(*S*-TPPTTL)$_4$, 5.0 equiv styrene, 1.0 equiv 8-chloroquinoline, CH_2Cl_2 as solvent, reaction performed according to procedure 3.4

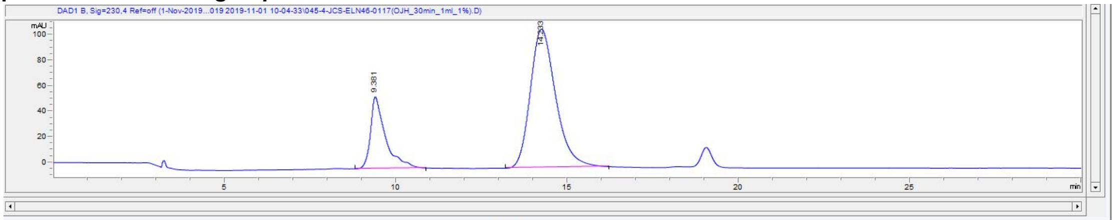

File Information

LC-File	045-4-JCS-ELN46-0117(OJH_30min_1ml_1%).D
File Path	C:\Chem32\1\Data\1-Nov-2019\31-OCT-2019 2019-11-01 10-04-3:
Date	02-Nov-19, 03:53:19
Sample	JCS-ELN46-0117(OJH_30min_1ml_1%)
Sample Info	
Barcode	
Operator	SYSTEM

#	Time	Type	Area	Height	Width	Area%	Symmetry
1	9.381	BB	1715.3	56.5	0.3951	23.400	0.453
2	14.233	BB	5615	108.9	0.6141	76.600	0.764

0 ℃, 1.0 mol % Rh_2(*S*-TPPTTL)$_4$, 5.0 equiv styrene, 1.0 equiv 2-Fpyridine, CH_2Cl_2 as solvent, reaction performed according to procedure 3.4

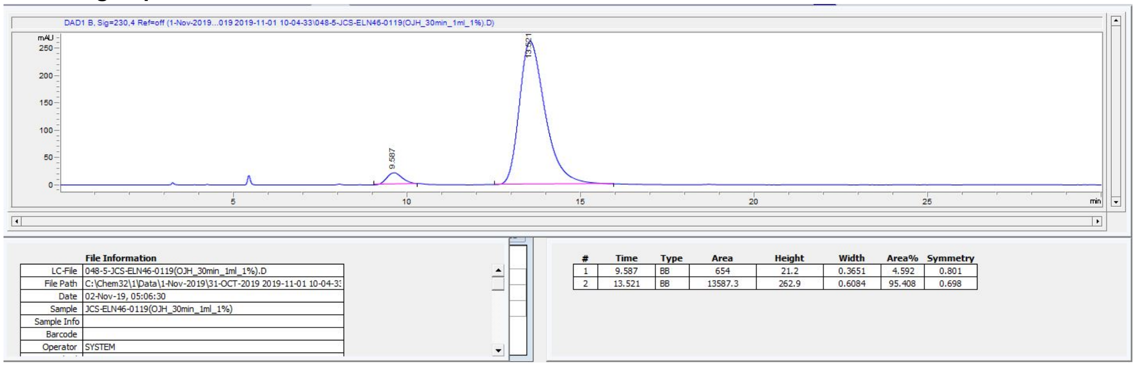

#	Time	Type	Area	Height	Width	Area%	Symmetry
1	9.587	BB	654	21.2	0.3651	4.592	0.801
2	13.521	BB	13587.3	262.9	0.6084	95.408	0.698

0 ℃, 1.0 mol % Rh_2(*S*-TPPTTL)$_4$, 5.0 equiv styrene, 1.0 equiv 2,6-lutidine, CH_2Cl_2 as solvent, reaction performed according to procedure 3.4

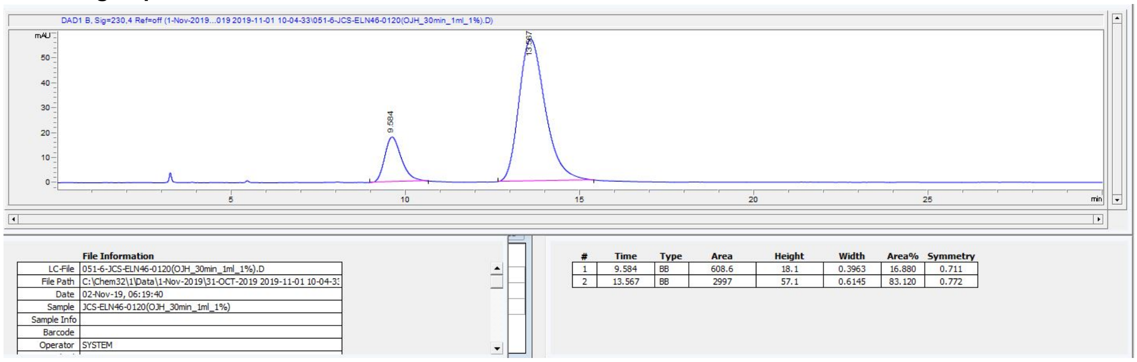

#	Time	Type	Area	Height	Width	Area%	Symmetry
1	9.584	BB	608.6	18.1	0.3963	16.880	0.711
2	13.567	BB	2997	57.1	0.6145	83.120	0.772

0 ℃, 1.0 mol % Rh_2(*S*-TPPTTL)$_4$, 5.0 equiv styrene, 1.0 equiv *N,N,N'N'*- tetramethyl urea, CH_2Cl_2 as solvent, reaction performed according to procedure 3.4

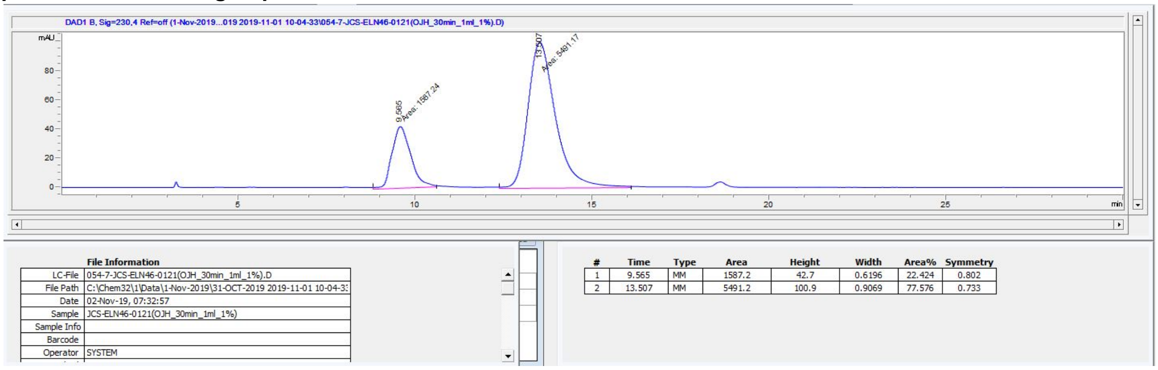

#	Time	Type	Area	Height	Width	Area%	Symmetry
1	9.565	MM	1587.2	42.7	0.6196	22.424	0.802
2	13.507	MM	5491.2	100.9	0.9069	77.576	0.733

6. NMR Spectra:

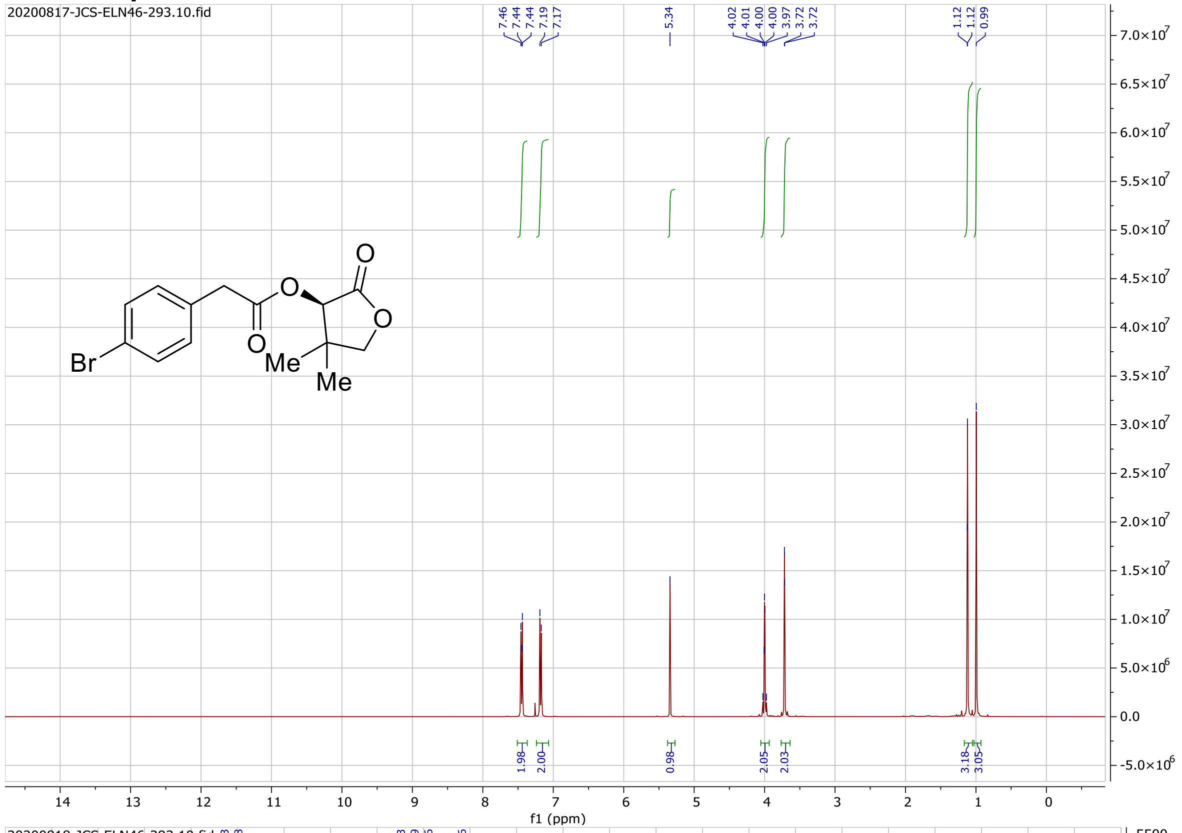

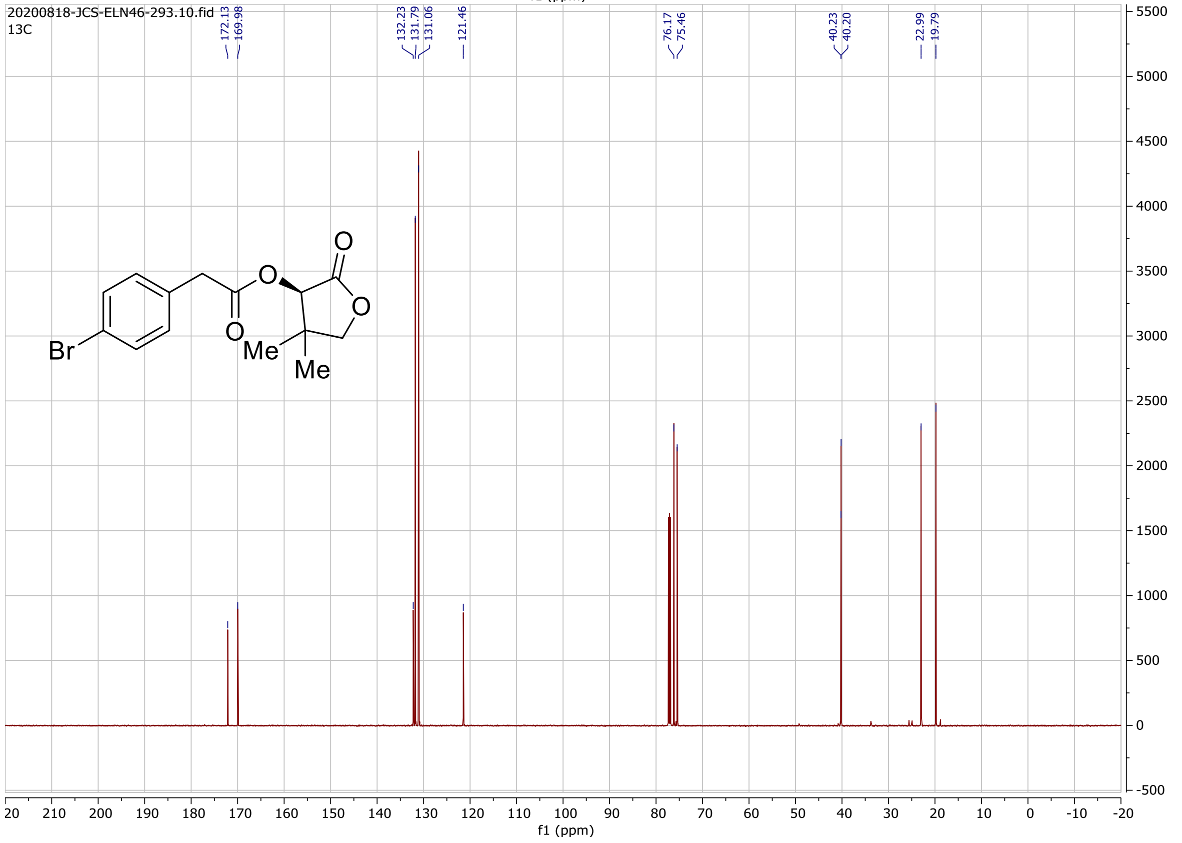

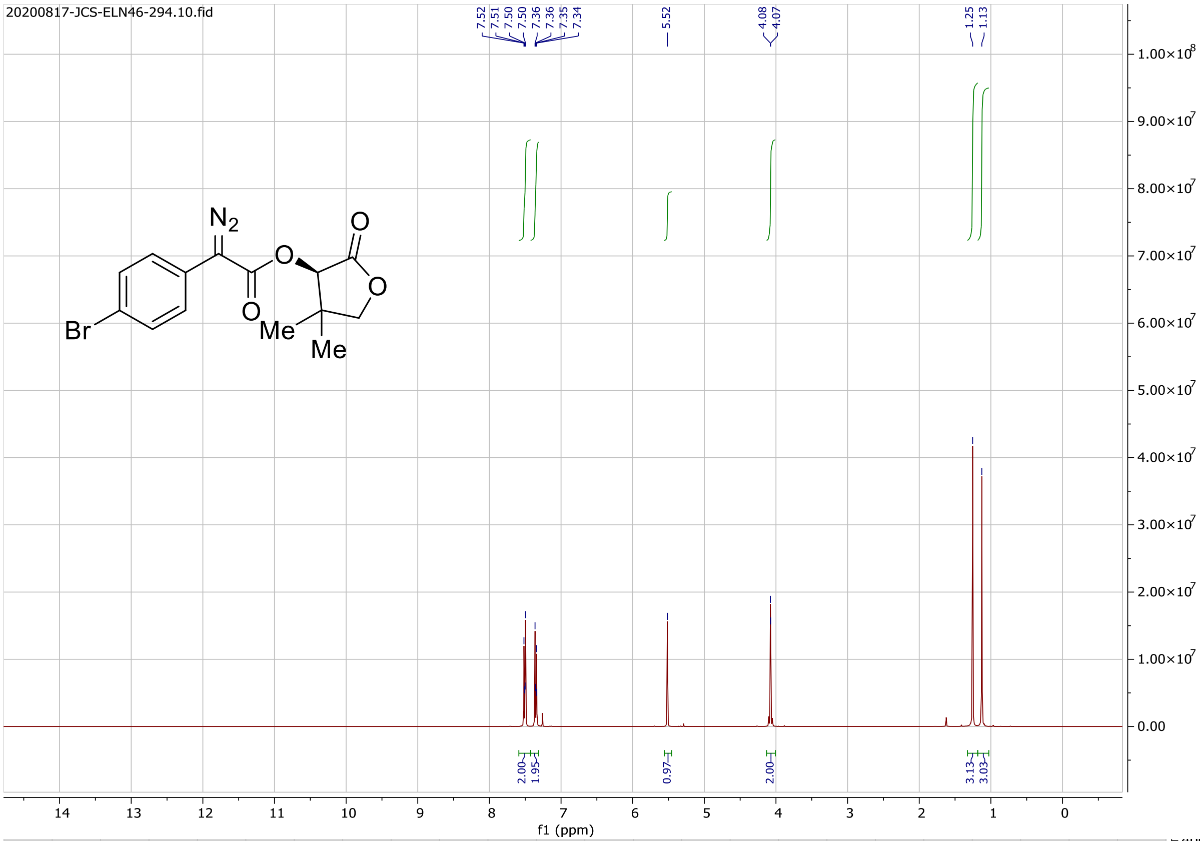
20200817-JCS-ELN46-294.10.fid
7.52
7.51
7.50
7.50
7.36
7.36
7.35
7.34
5.52
4.08
4.07
1.25
1.13
N2
O
O
O
O
Br
Me
Me
2.00
1.95
0.97
2.00
3.13
3.03
f1 (ppm)

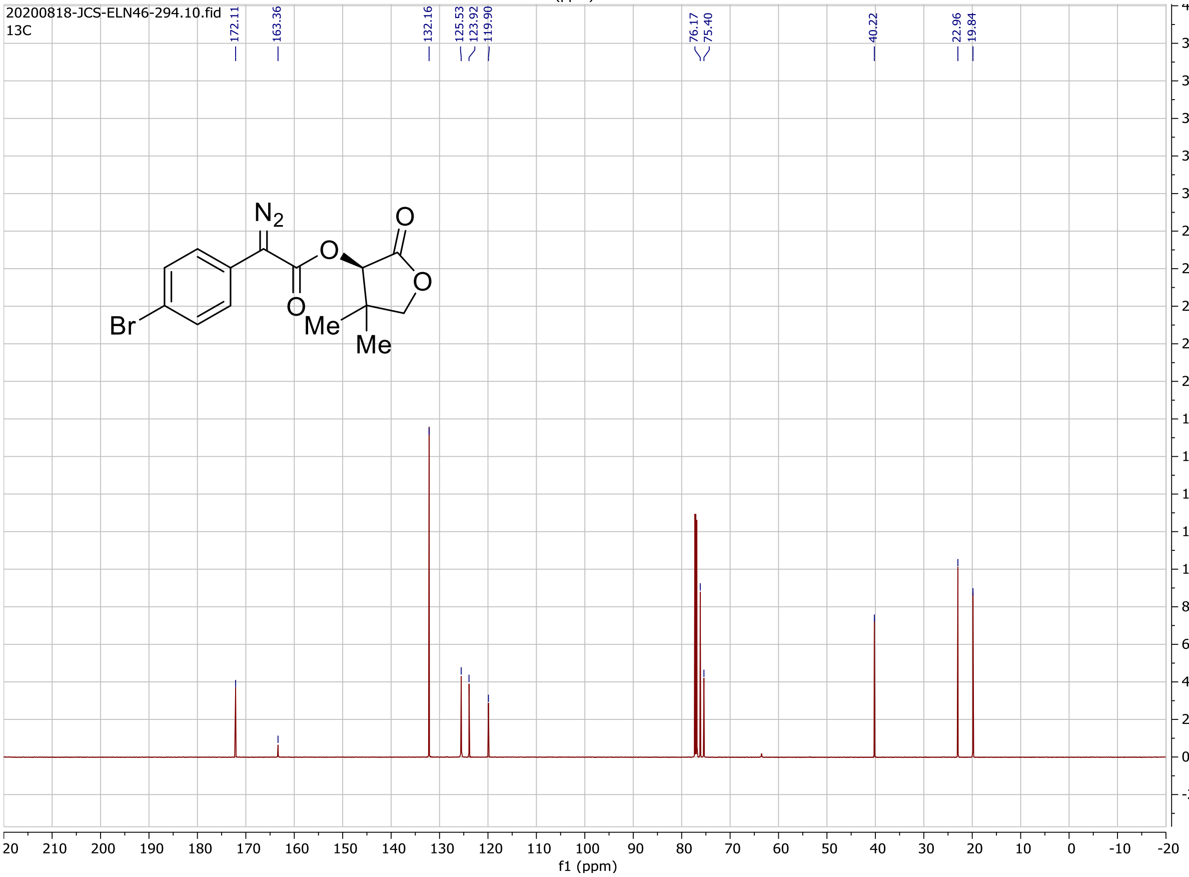
20200818-JCS-ELN46-294.10.fid
13C
172.11
163.36
132.16
125.53
123.92
119.90
76.17
75.40
40.22
22.96
19.84
N2
O
O
O
O
Br
Me
Me
f1 (ppm)

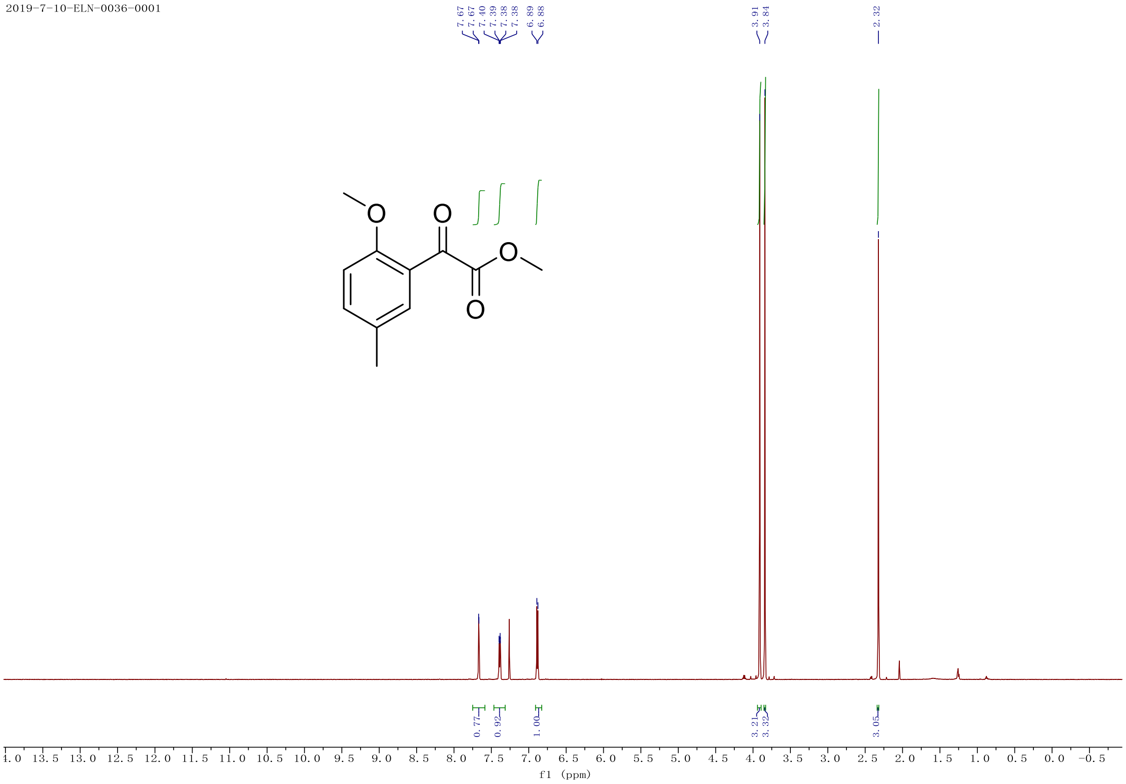
2019-7-10-ELN-0036-0001
7.67
7.67
7.40
7.39
7.38
7.38
6.89
6.88
3.91
3.84
2.32
0.77
0.92
1.00
3.21
3.32
3.05
f1 (ppm)

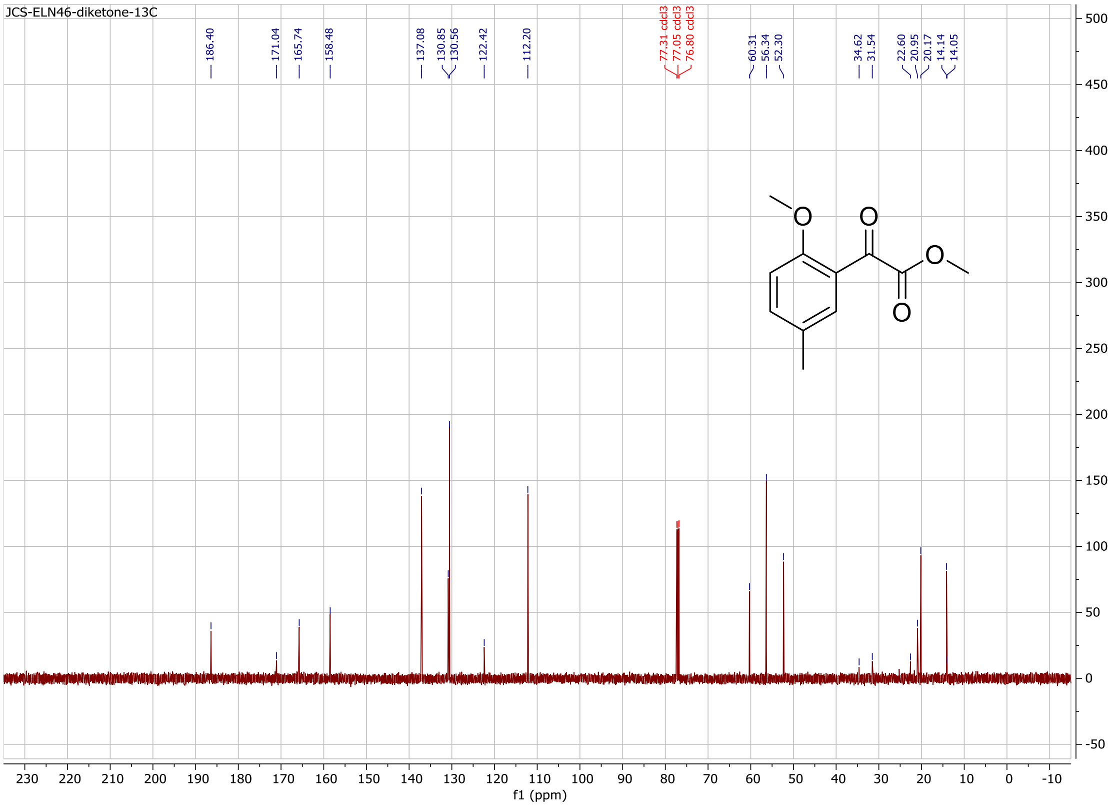
JCS-ELN46-diketone-13C
186.40
171.04
165.74
158.48
137.08
130.85
130.56
122.42
112.20
77.31 cdcl3
77.05 cdcl3
76.80 cdcl3
60.31
56.34
52.30
34.62
31.54
22.60
20.95
20.17
14.14
14.05
f1 (ppm)

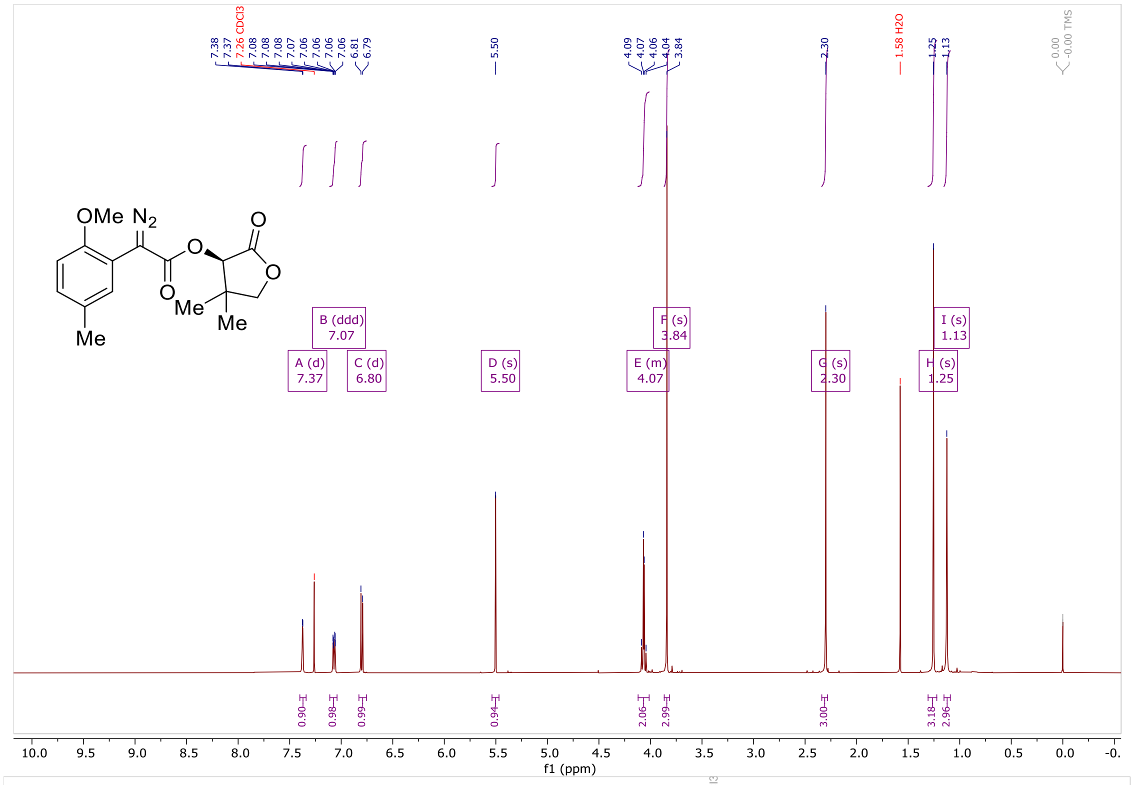

7.38
7.37
7.26 CDCl3
7.08
7.08
7.08
7.08
7.07
7.06
7.06
7.06
7.06
6.81
6.79
5.50
4.09
4.07
4.06
4.04
3.84
2.30
1.58 H2O
1.25
1.13
0.00
-0.00 TMS
OMe N2
O
O
O
O
Me
Me
Me
A (d) 7.37
B (ddd) 7.07
C (d) 6.80
D (s) 5.50
E (m) 4.07
F (s) 3.84
G (s) 2.30
H (s) 1.25
I (s) 1.13
0.90
0.98
0.99
0.94
2.06
2.99
3.00
3.18
2.96
10.0 9.5 9.0 8.5 8.0 7.5 7.0 6.5 6.0 5.5 5.0 4.5 4.0 3.5 3.0 2.5 2.0 1.5 1.0 0.5 0.0
f1 (ppm)

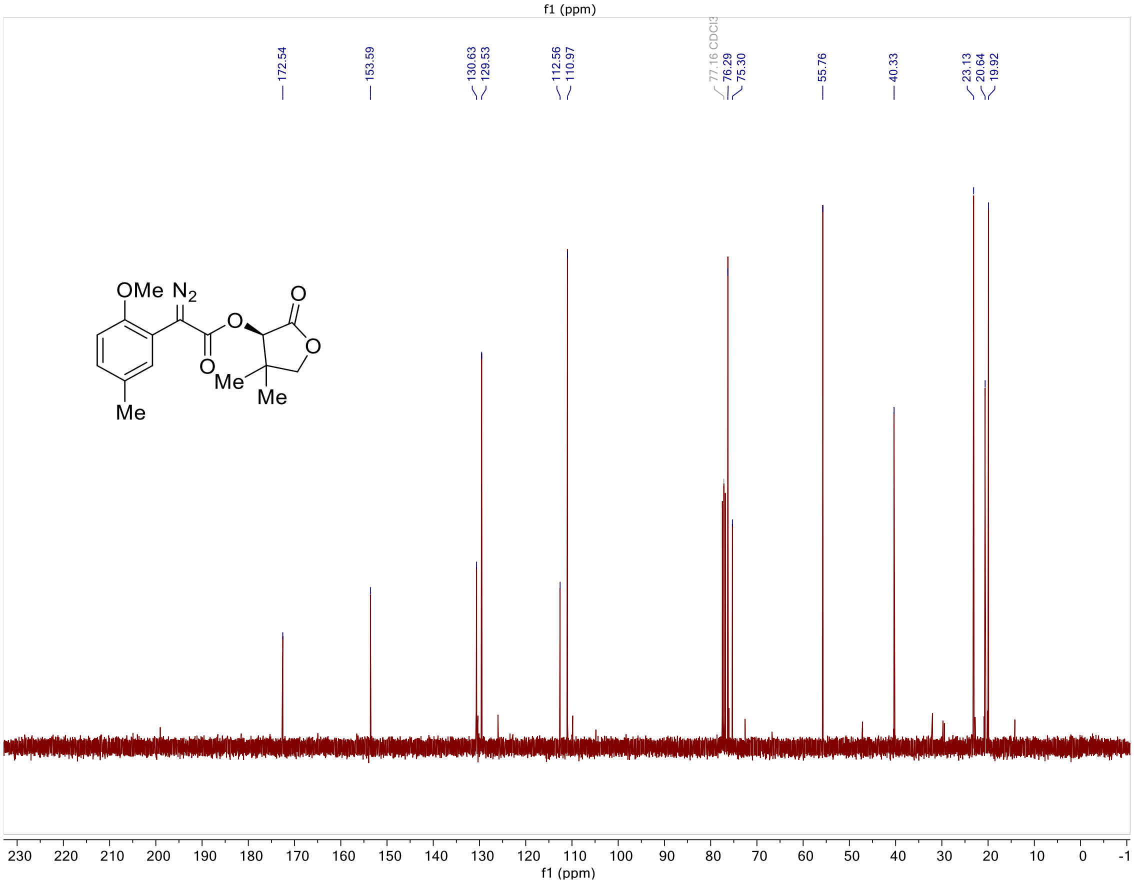

172.54
153.59
130.63
129.53
112.56
110.97
77.16 CDCl3
76.29
75.30
55.76
40.33
23.13
20.64
19.92
OMe N2
O
O
O
O
Me
Me
Me
230 220 210 200 190 180 170 160 150 140 130 120 110 100 90 80 70 60 50 40 30 20 10 0 -1
f1 (ppm)

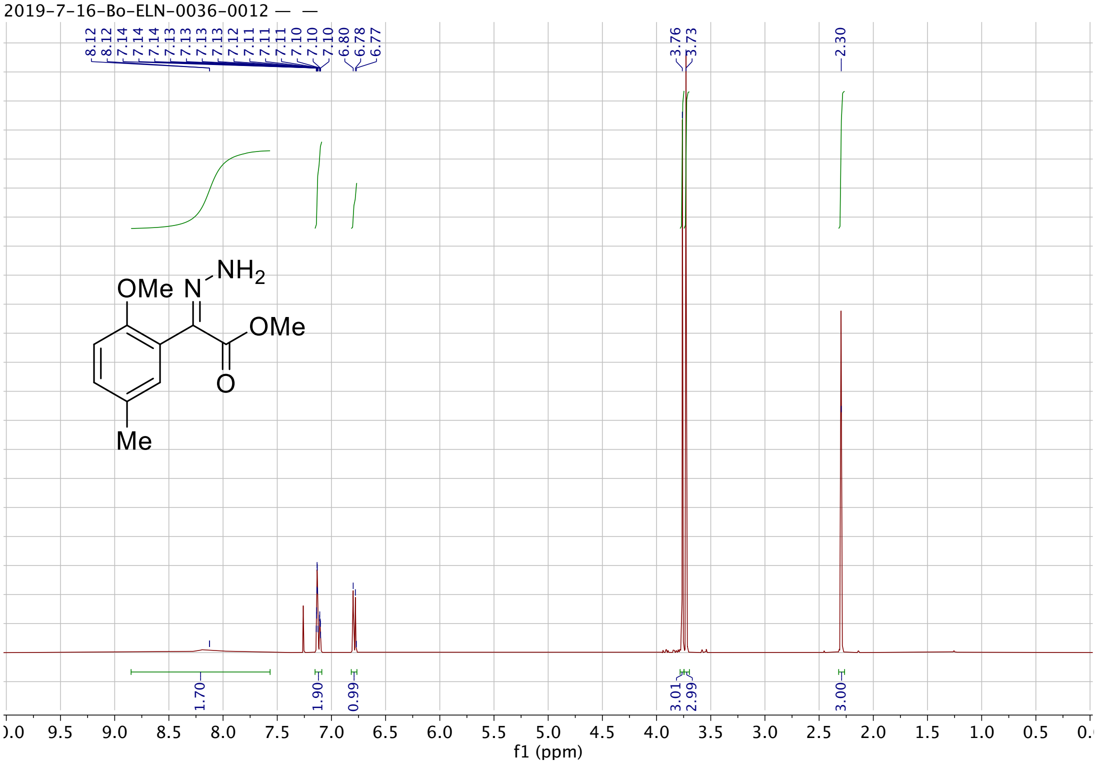
2019-7-16-Bo-ELN-0036-0012 — —
8.12
8.12
7.14
7.14
7.13
7.13
7.13
7.13
7.12
7.11
7.11
7.11
7.10
7.10
7.10
6.80
6.78
6.77
3.76
3.73
2.30
OMe
N
NH2
OMe
O
Me
1.70
1.90
0.99
3.01
2.99
3.00
f1 (ppm)

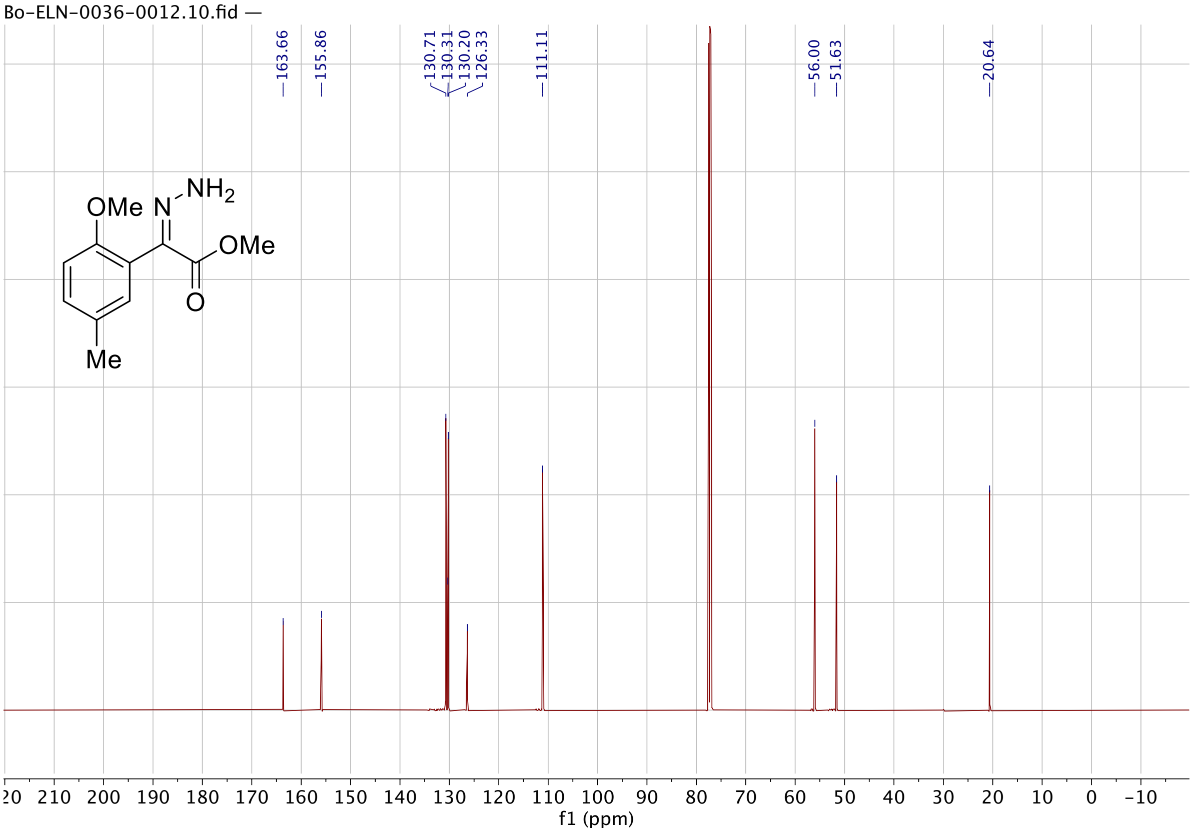
Bo-ELN-0036-0012.10.fid —
163.66
155.86
130.71
130.31
130.20
126.33
111.11
56.00
51.63
20.64
OMe
N
NH2
OMe
O
Me
f1 (ppm)

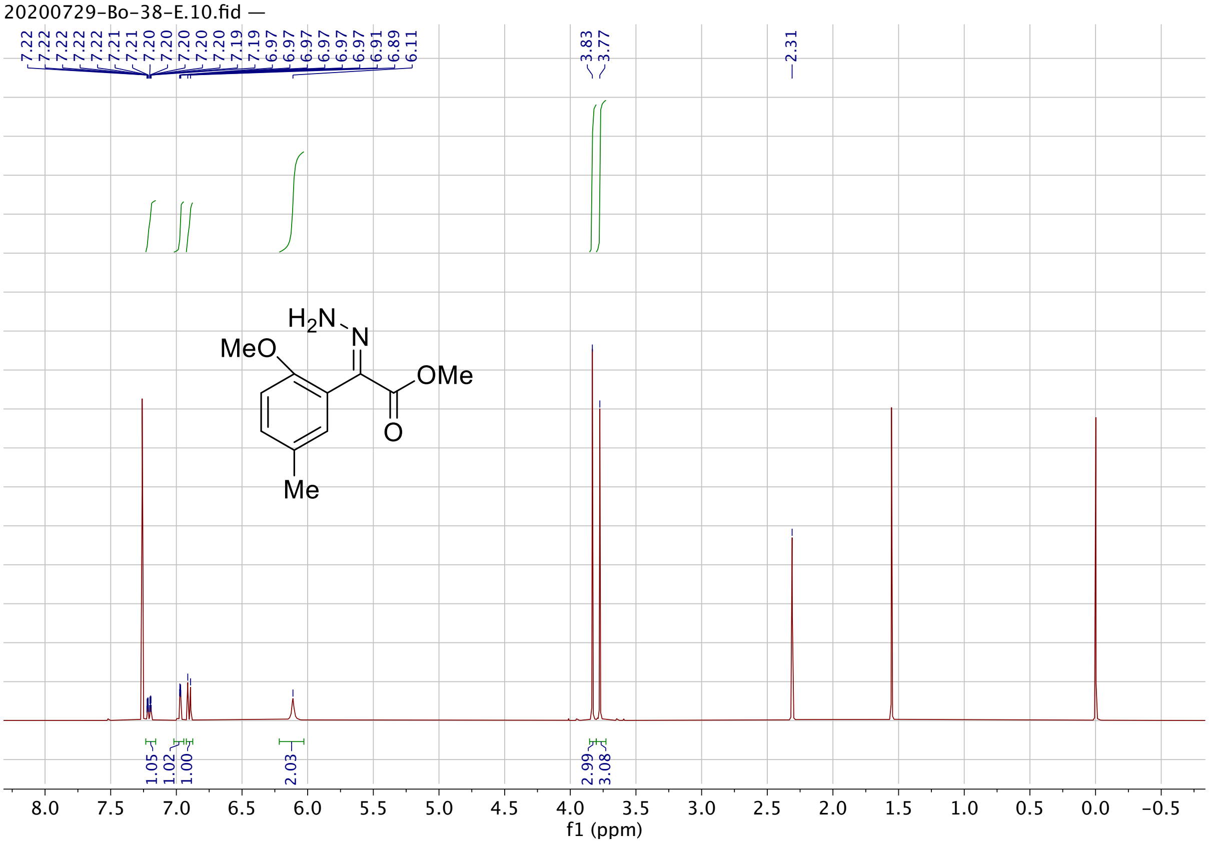
20200729-Bo-38-E.10.fid —
7.22
7.22
7.22
7.22
7.22
7.21
7.21
7.20
7.20
7.20
7.20
7.20
7.19
7.19
6.97
6.97
6.97
6.97
6.97
6.97
6.91
6.89
6.11
3.83
3.77
2.31
H2N
N
MeO
OMe
O
Me
1.05
1.02
1.00
2.03
2.99
3.08
f1 (ppm)

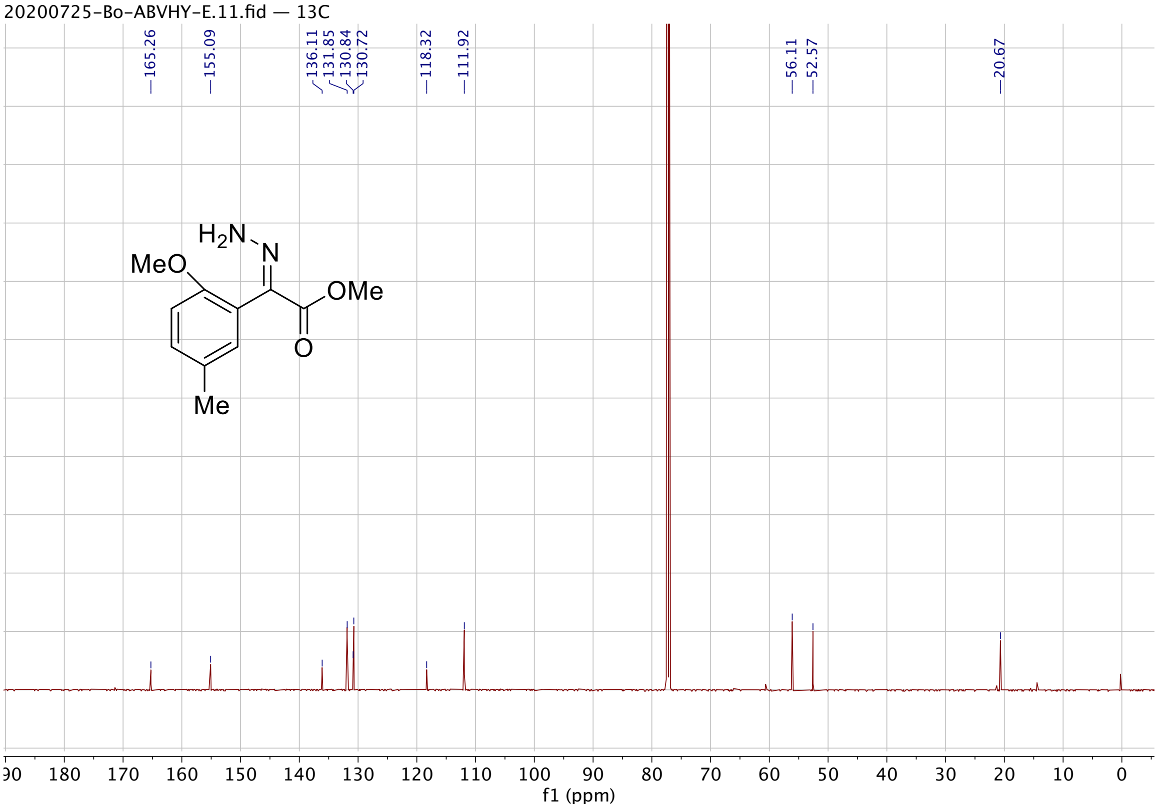
20200725-Bo-ABVHY-E.11.fid — 13C
165.26
155.09
136.11
131.85
130.84
130.72
118.32
111.92
56.11
52.57
20.67
H2N
N
MeO
OMe
O
Me
f1 (ppm)

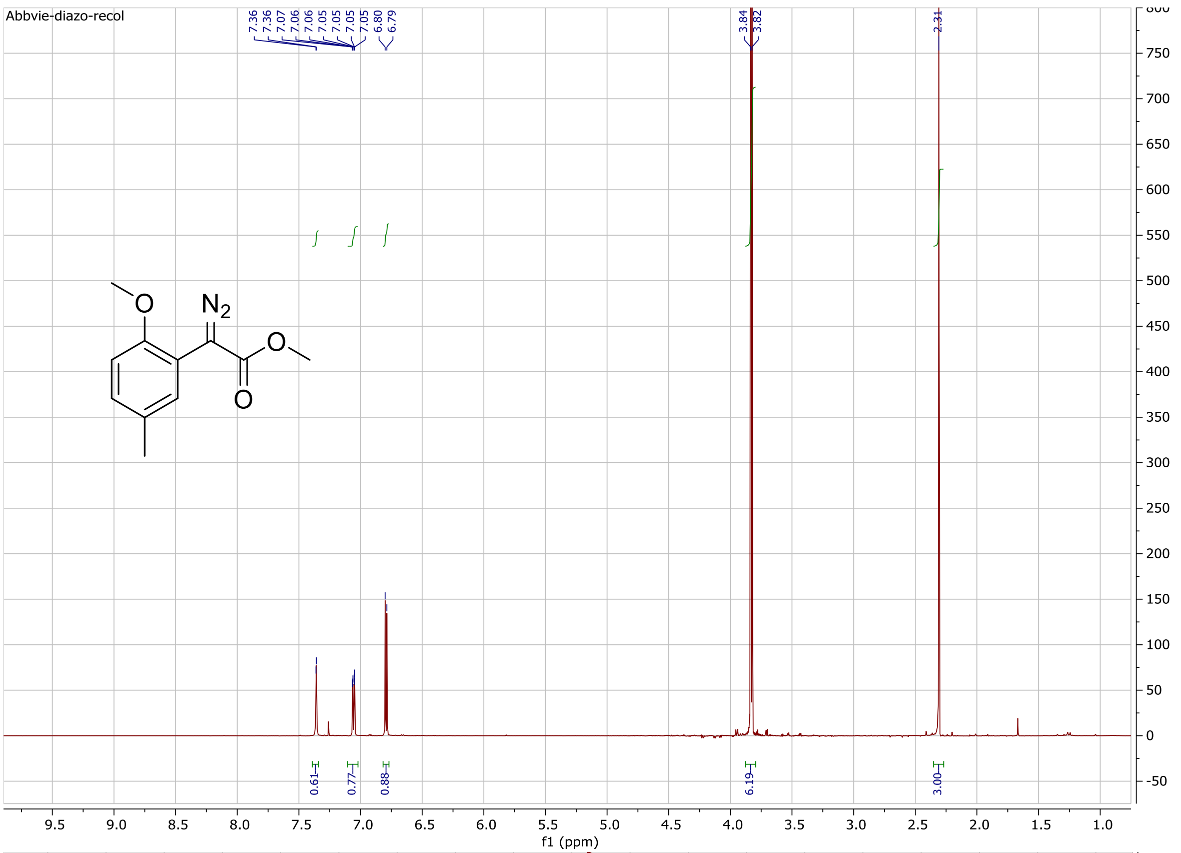
Abbvie-diazo-recol
7.36
7.36
7.07
7.06
7.06
7.05
7.05
7.05
7.05
6.80
6.79
3.84
3.82
2.31
0.61
0.77
0.88
6.19
3.00
N2
O
O
O
f1 (ppm)

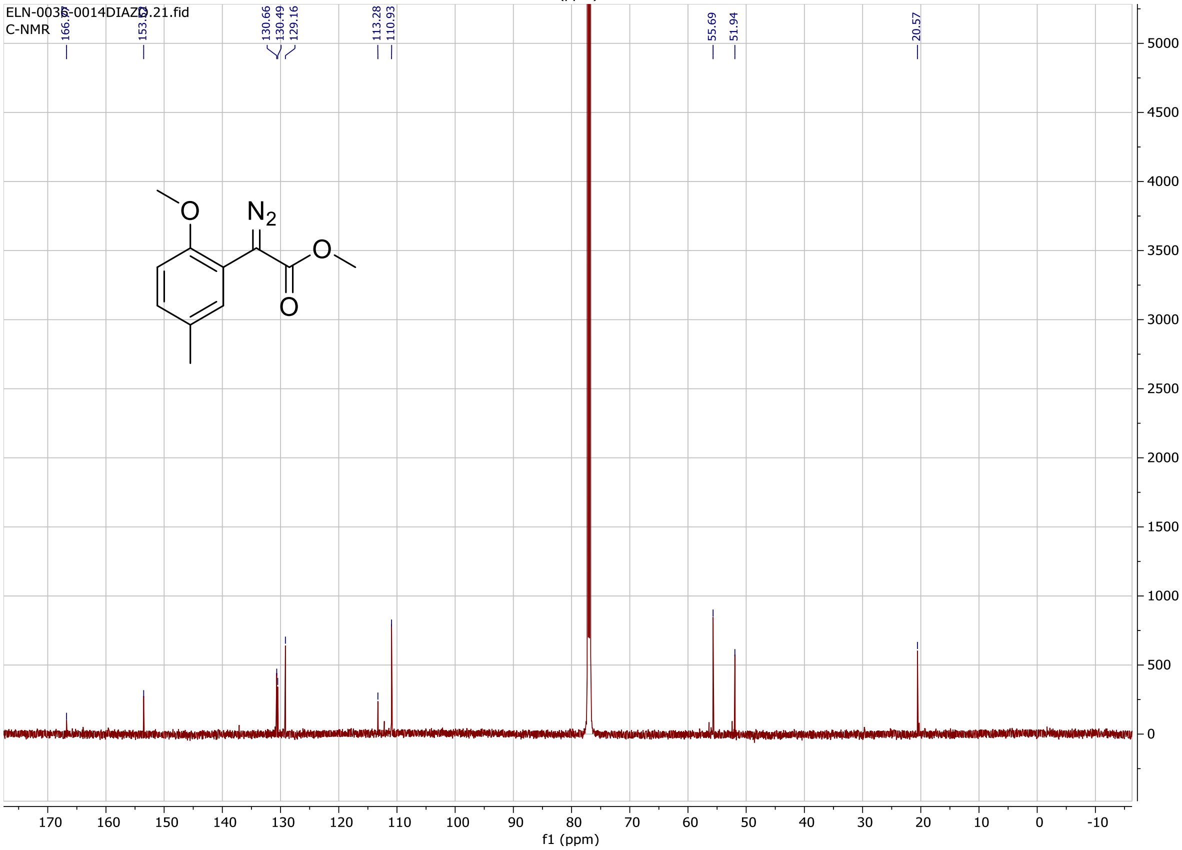
C-NMR
130.66
130.49
129.16
113.28
110.93
55.69
51.94
20.57
N2
O
O
O
f1 (ppm)

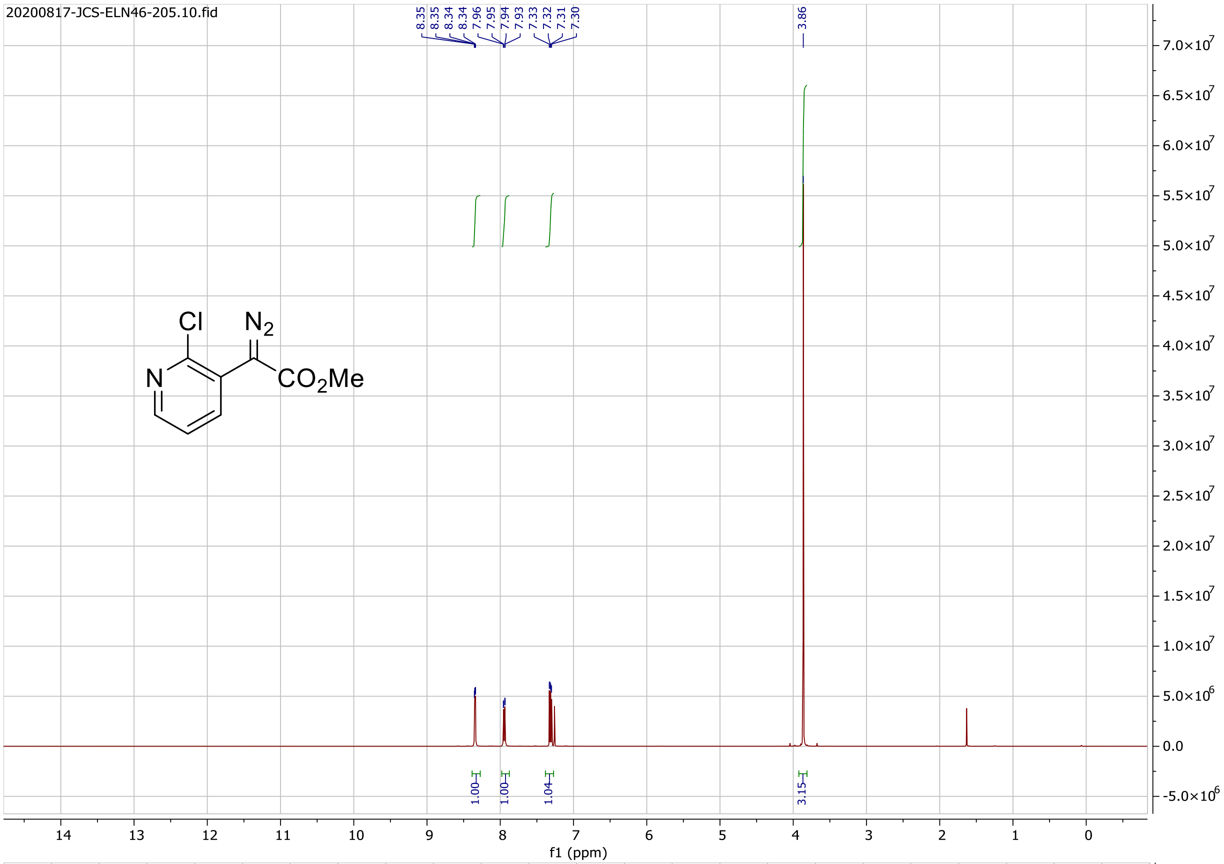
20200817-JCS-ELN46-205.10.fid
8.35
8.35
8.34
8.34
7.96
7.95
7.94
7.93
7.33
7.32
7.31
7.30
3.86
Cl
N2
N
CO2Me
1.00
1.00
1.04
3.15
f1 (ppm)

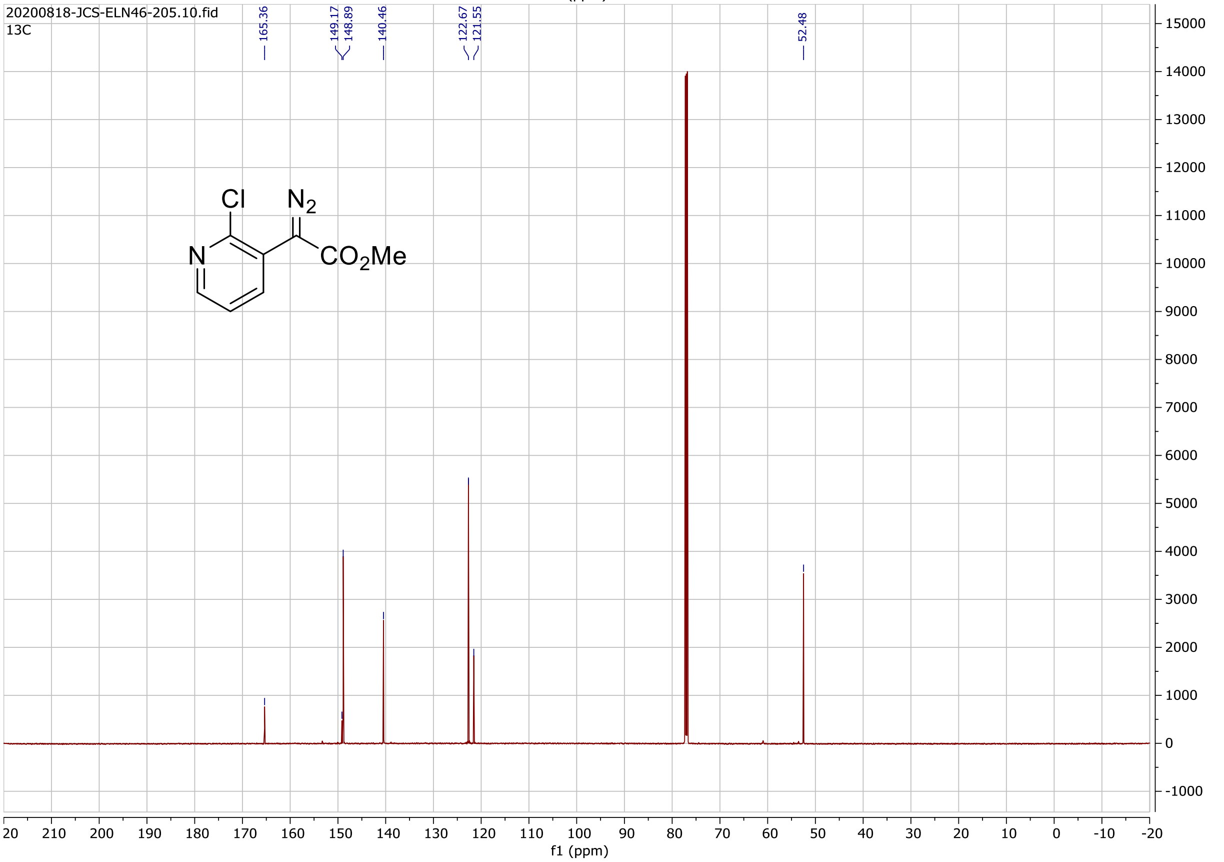
20200818-JCS-ELN46-205.10.fid
13C
165.36
149.17
148.89
140.46
122.67
121.55
52.48
Cl
N2
N
CO2Me
f1 (ppm)

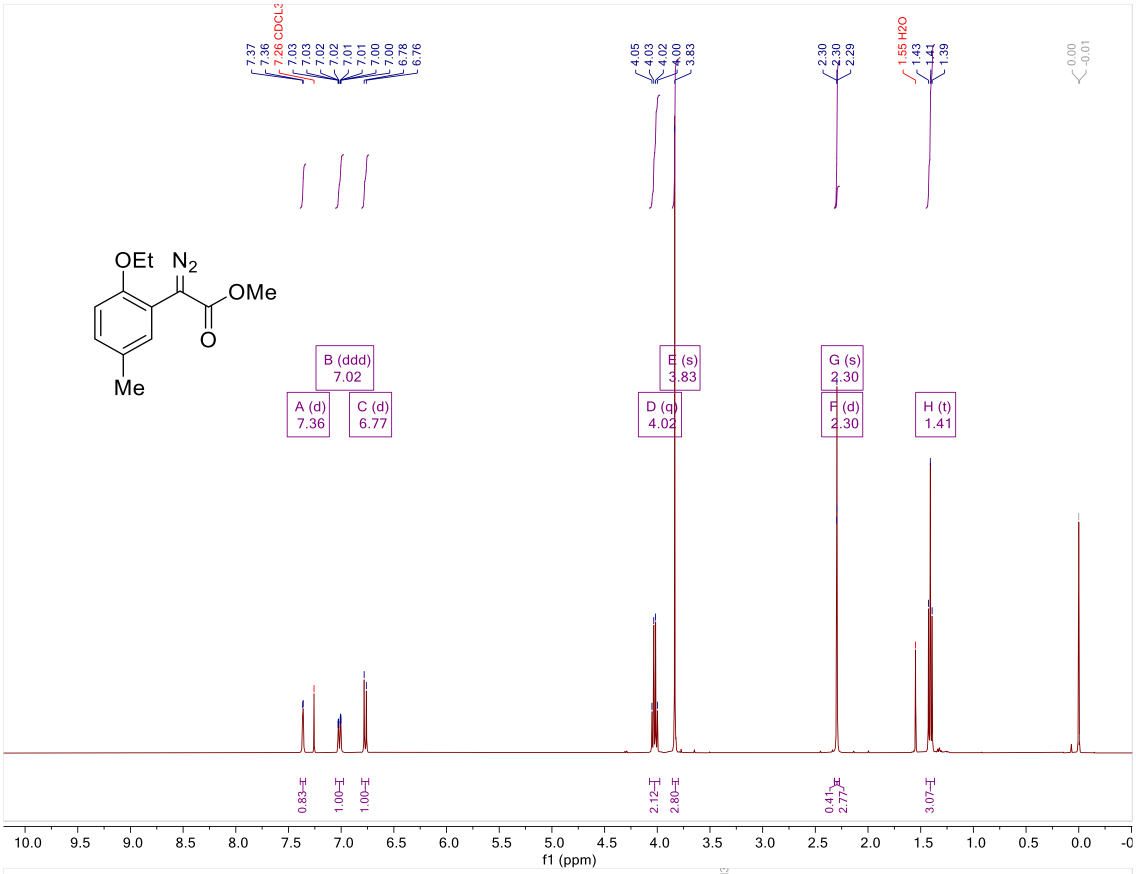

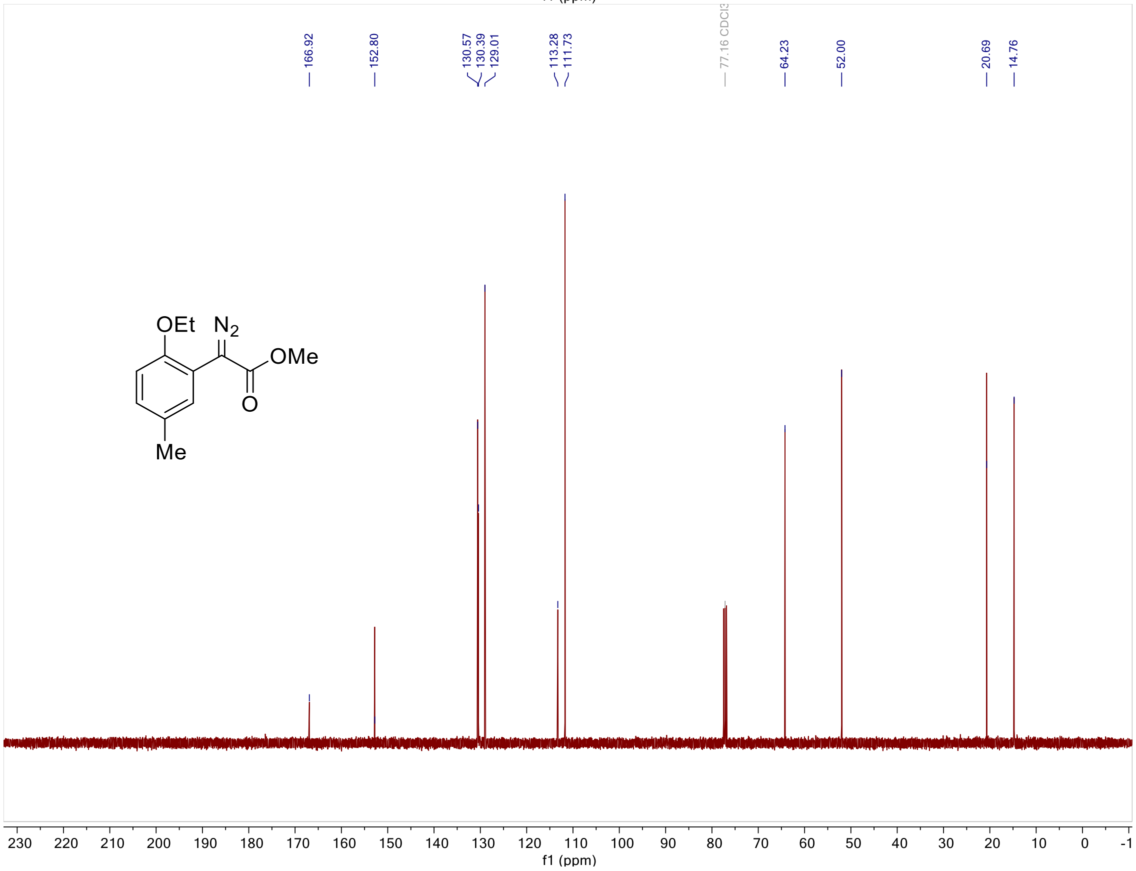

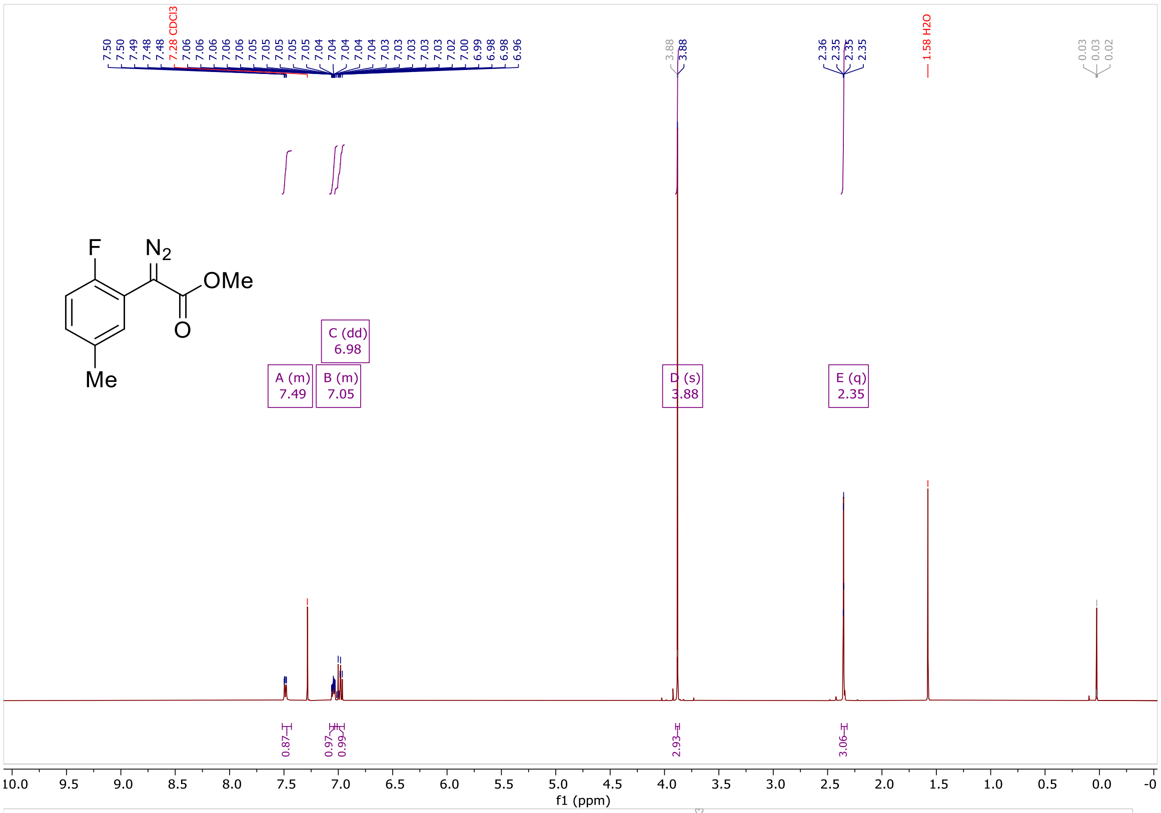

7.50
7.50
7.49
7.48
7.48
7.28 CDCl3
7.06
7.06
7.06
7.06
7.05
7.05
7.05
7.05
7.05
7.04
7.04
7.04
7.04
7.04
7.04
7.03
7.03
7.03
7.03
7.03
7.02
7.00
6.99
6.98
6.98
6.96
3.88
3.88
2.36
2.35
2.35
2.35
1.58 H2O
0.03
0.03
0.02
F
N2
OMe
O
Me
C (dd)
6.98
A (m)
7.49
B (m)
7.05
D (s)
3.88
E (q)
2.35
0.87
0.97
0.99
2.93
3.06
10.0
9.5
9.0
8.5
8.0
7.5
7.0
6.5
6.0
5.5
5.0
4.5
4.0
3.5
3.0
2.5
2.0
1.5
1.0
0.5
0.0
-0
f1 (ppm)

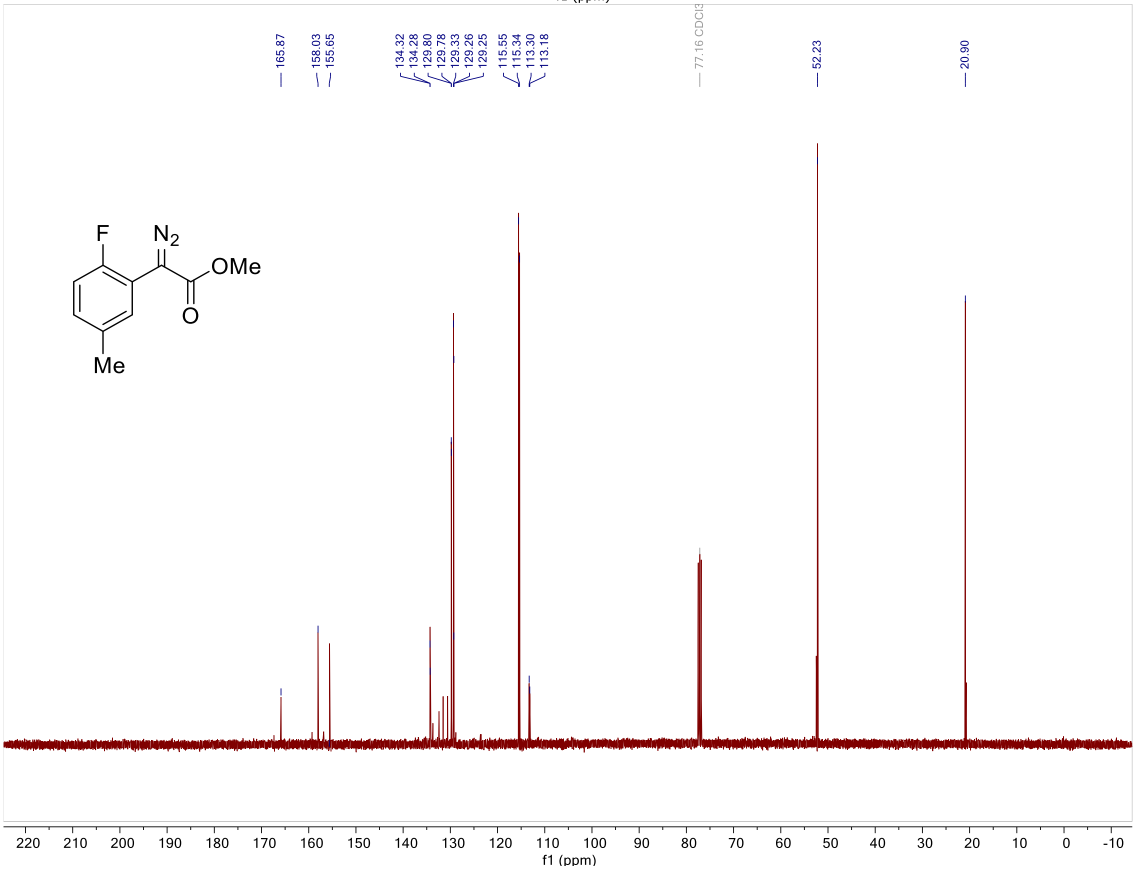

165.87
158.03
155.65
134.32
134.28
129.80
129.78
129.33
129.26
129.25
115.55
115.34
113.30
113.18
77.16 CDCl3
52.23
20.90
F
N2
OMe
O
Me
220
210
200
190
180
170
160
150
140
130
120
110
100
90
80
70
60
50
40
30
20
10
0
-10
f1 (ppm)

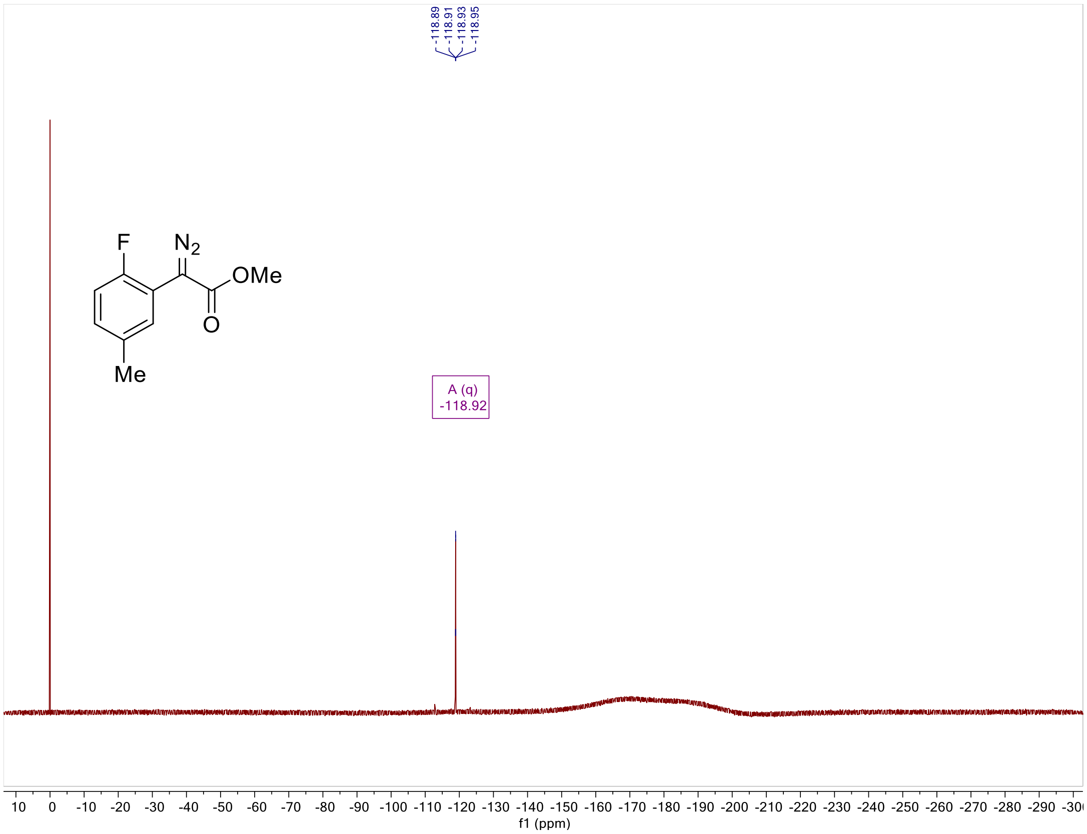
-118.89
-118.91
-118.93
-118.95
F
N2
OMe
O
Me
A (q)
-118.92
10 0 -10 -20 -30 -40 -50 -60 -70 -80 -90 -100 -110 -120 -130 -140 -150 -160 -170 -180 -190 -200 -210 -220 -230 -240 -250 -260 -270 -280 -290
f1 (ppm)

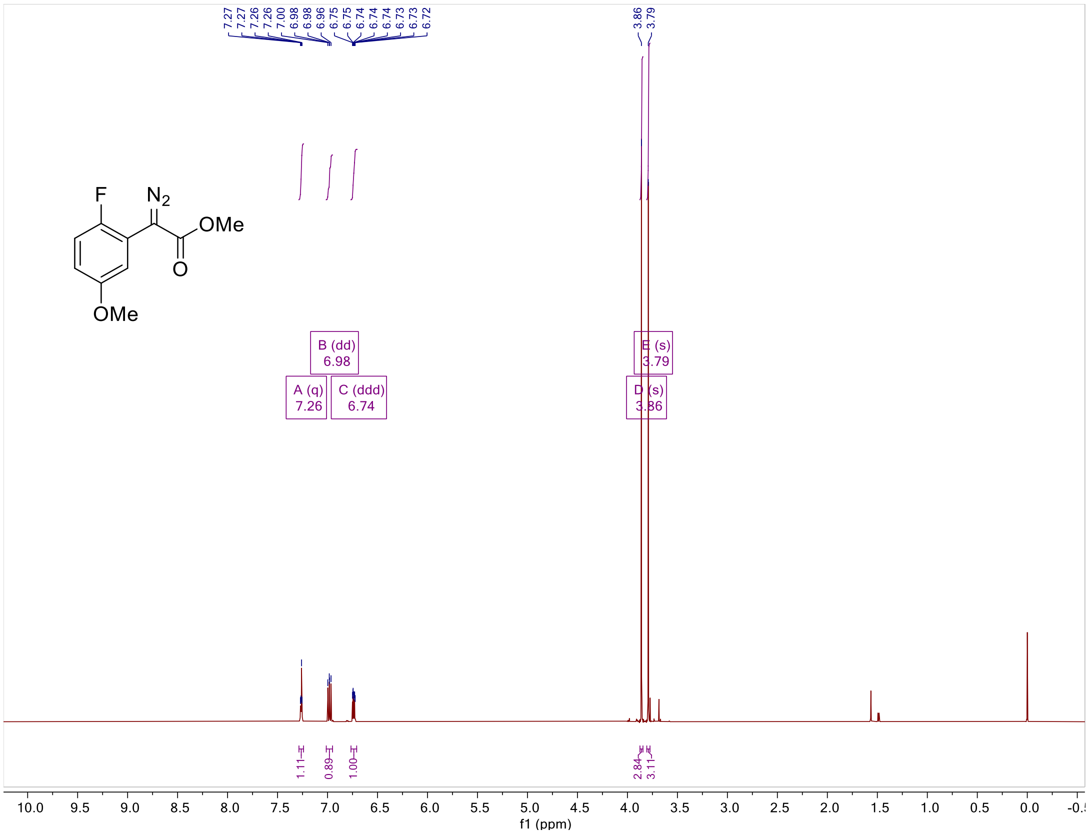
7.27
7.27
7.26
7.26
7.00
6.98
6.98
6.96
6.75
6.75
6.74
6.74
6.74
6.74
6.73
6.72
3.86
3.79
F
N2
OMe
O
OMe
B (dd)
6.98
A (q)
7.26
C (ddd)
6.74
E (s)
3.79
D (s)
3.86
1.11
0.89
1.00
2.84
3.11
10.0 9.5 9.0 8.5 8.0 7.5 7.0 6.5 6.0 5.5 5.0 4.5 4.0 3.5 3.0 2.5 2.0 1.5 1.0 0.5 0.0
f1 (ppm)

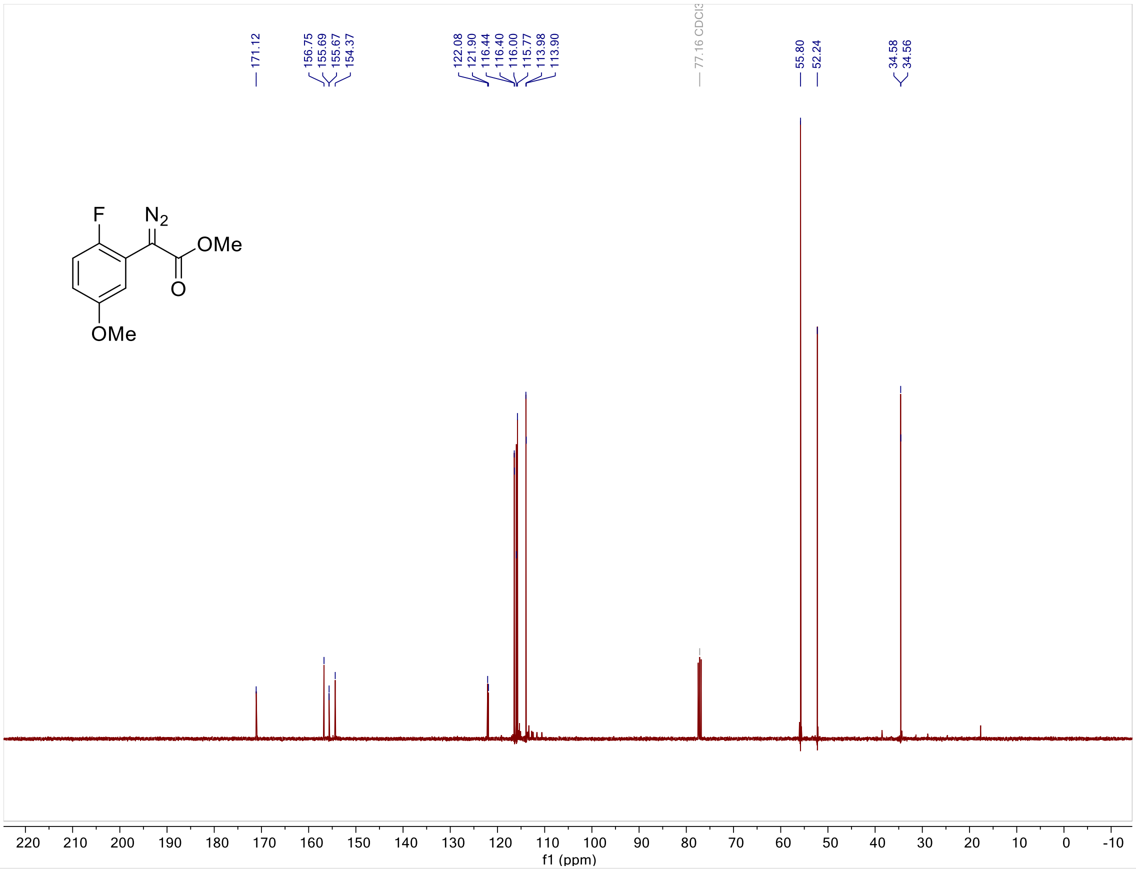
171.12
156.75
155.69
155.67
154.37
122.08
121.90
116.44
116.40
116.00
115.77
113.98
113.90
77.16 CDCl3
55.80
52.24
34.58
34.56
F
N2
OMe
O
OMe
220 210 200 190 180 170 160 150 140 130 120 110 100 90 80 70 60 50 40 30 20 10 0 -10
f1 (ppm)

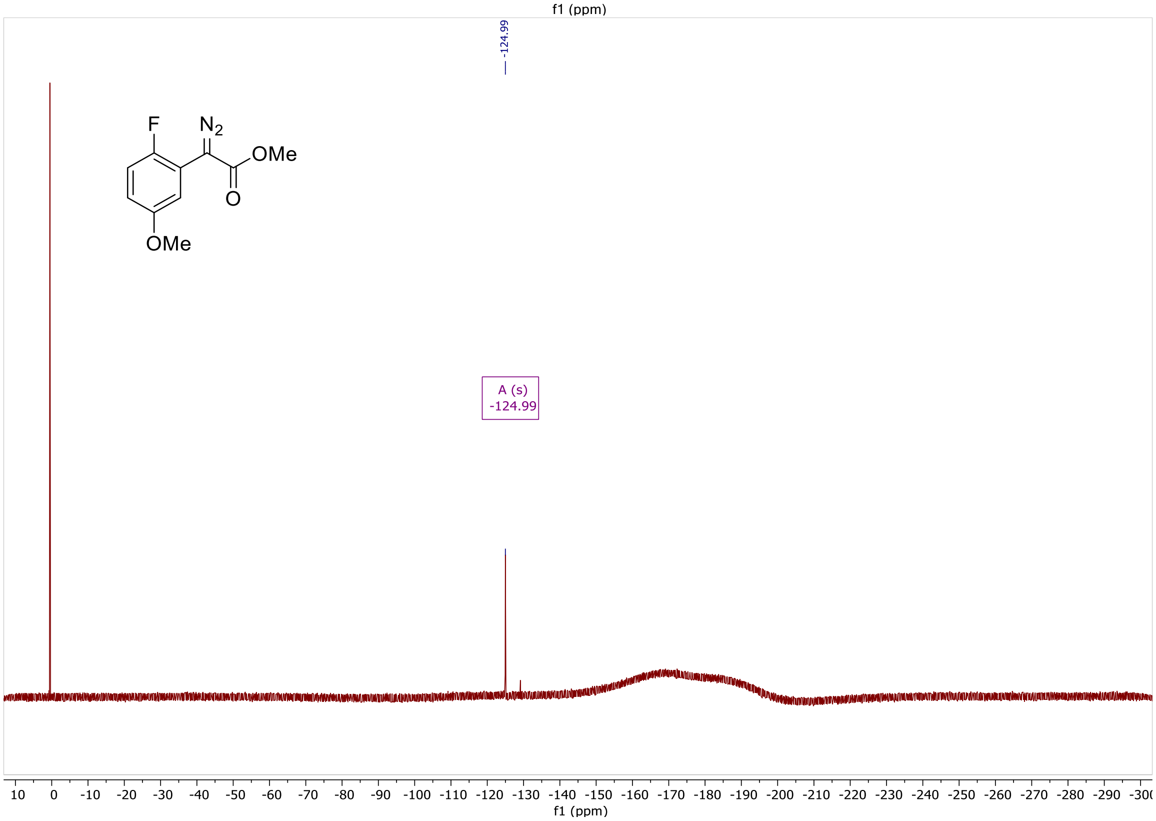
-124.99
F
N2
OMe
O
OMe
A (s)
-124.99
10 0 -10 -20 -30 -40 -50 -60 -70 -80 -90 -100 -110 -120 -130 -140 -150 -160 -170 -180 -190 -200 -210 -220 -230 -240 -250 -260 -270 -280 -290 -300
f1 (ppm)

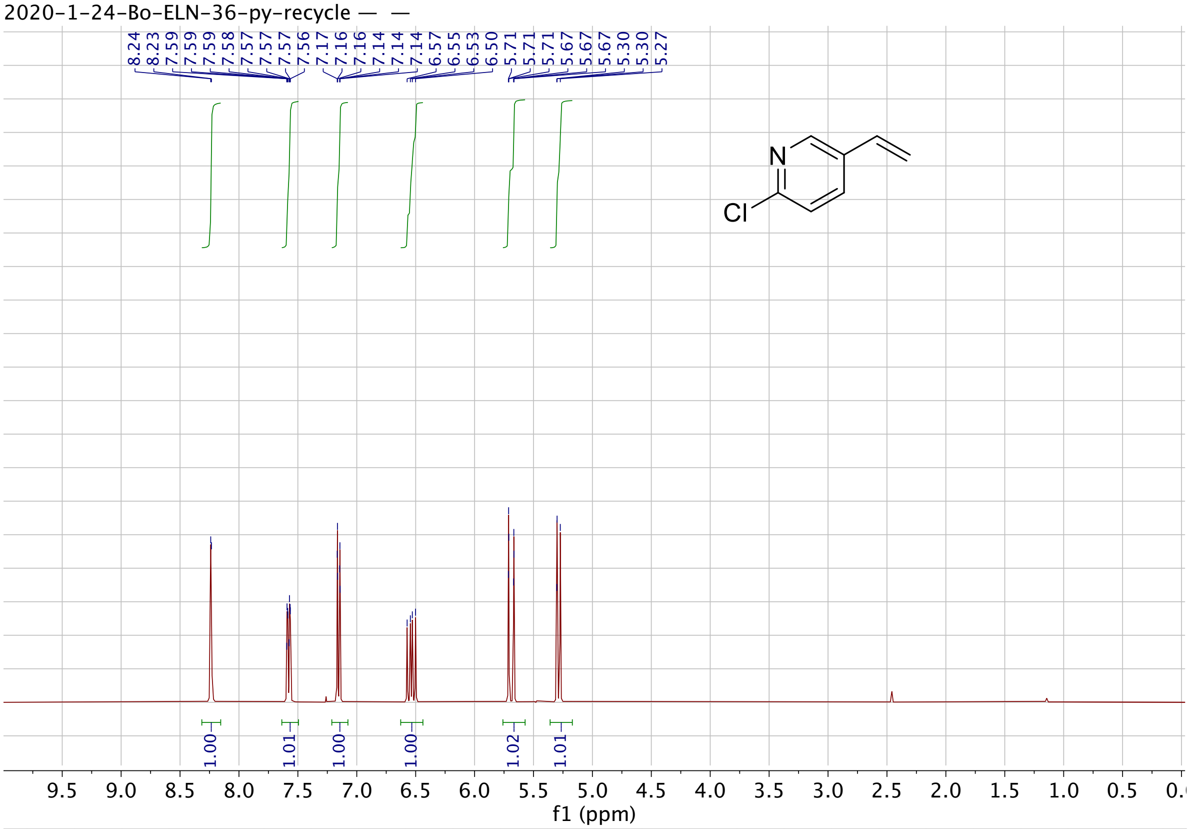
2020-1-24-Bo-ELN-36-py-recycle — —
8.24
8.23
7.59
7.59
7.58
7.57
7.57
7.57
7.56
7.17
7.16
7.14
7.14
6.57
6.55
6.53
6.50
5.71
5.71
5.71
5.67
5.67
5.30
5.27
N
Cl
1.00
1.01
1.00
1.00
1.02
1.01
f1 (ppm)

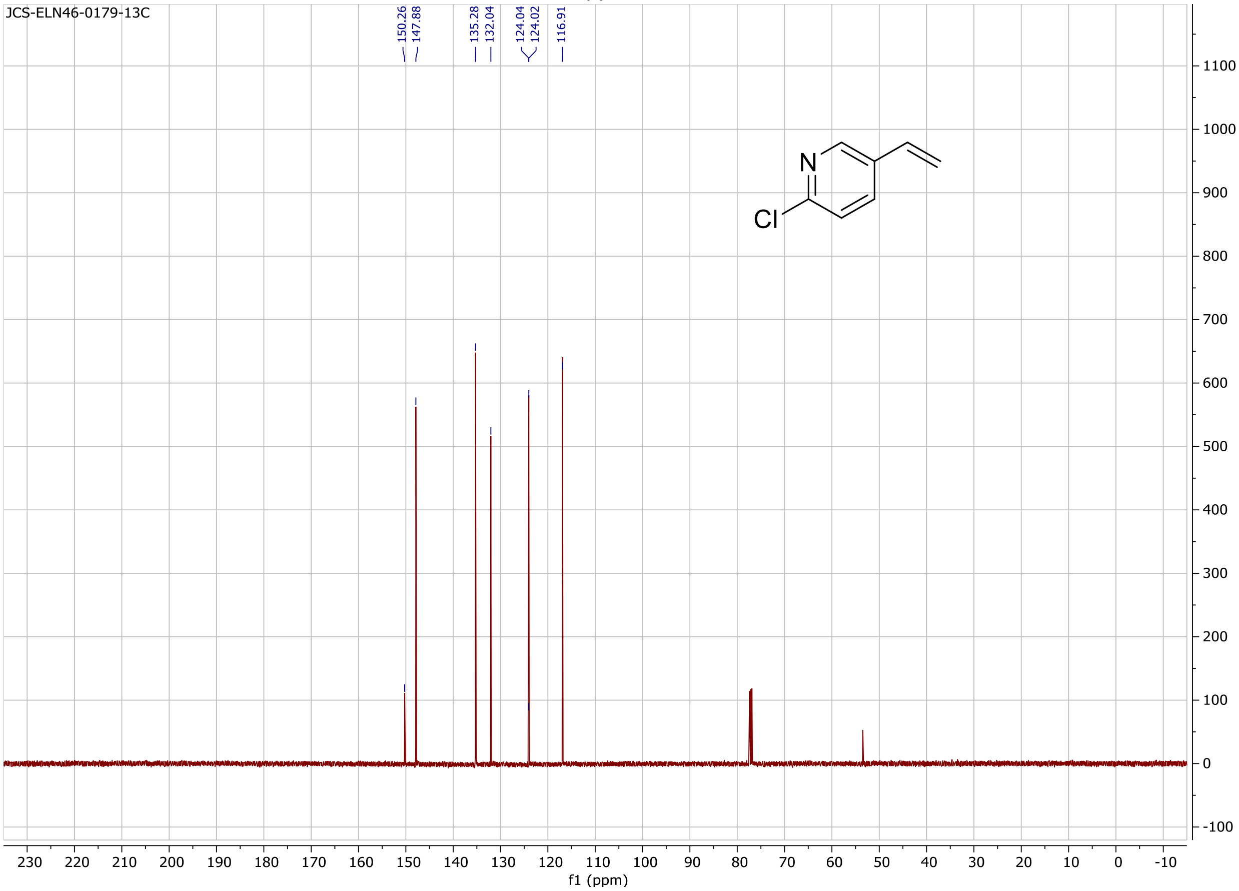
JCS-ELN46-0179-13C
150.26
147.88
135.28
132.04
124.04
124.02
116.91
N
Cl
f1 (ppm)

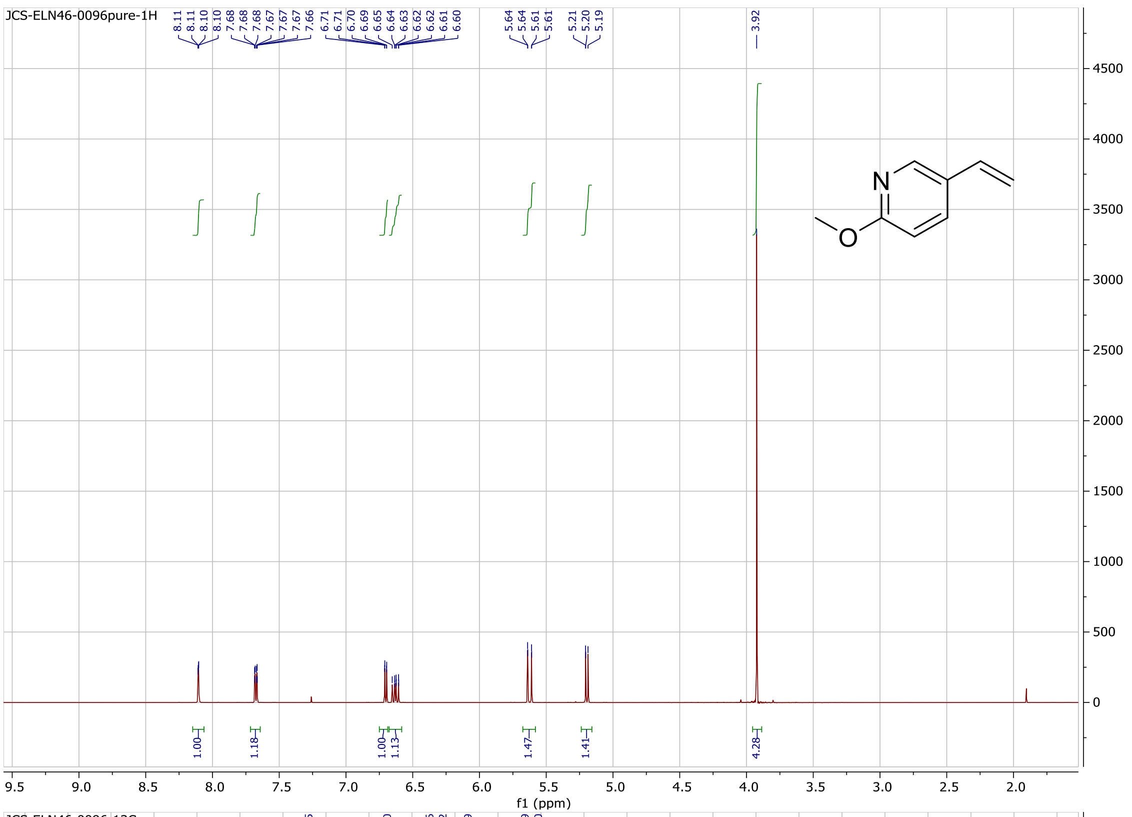
JCS-ELN46-0096pure-1H
8.11
8.11
8.10
8.10
7.68
7.68
7.67
7.67
7.67
7.66
6.71
6.71
6.70
6.69
6.65
6.64
6.63
6.62
6.62
6.61
6.60
5.64
5.64
5.61
5.61
5.21
5.20
5.19
3.92
1.00
1.18
1.00
1.13
1.47
1.41
4.28
N
O
f1 (ppm)

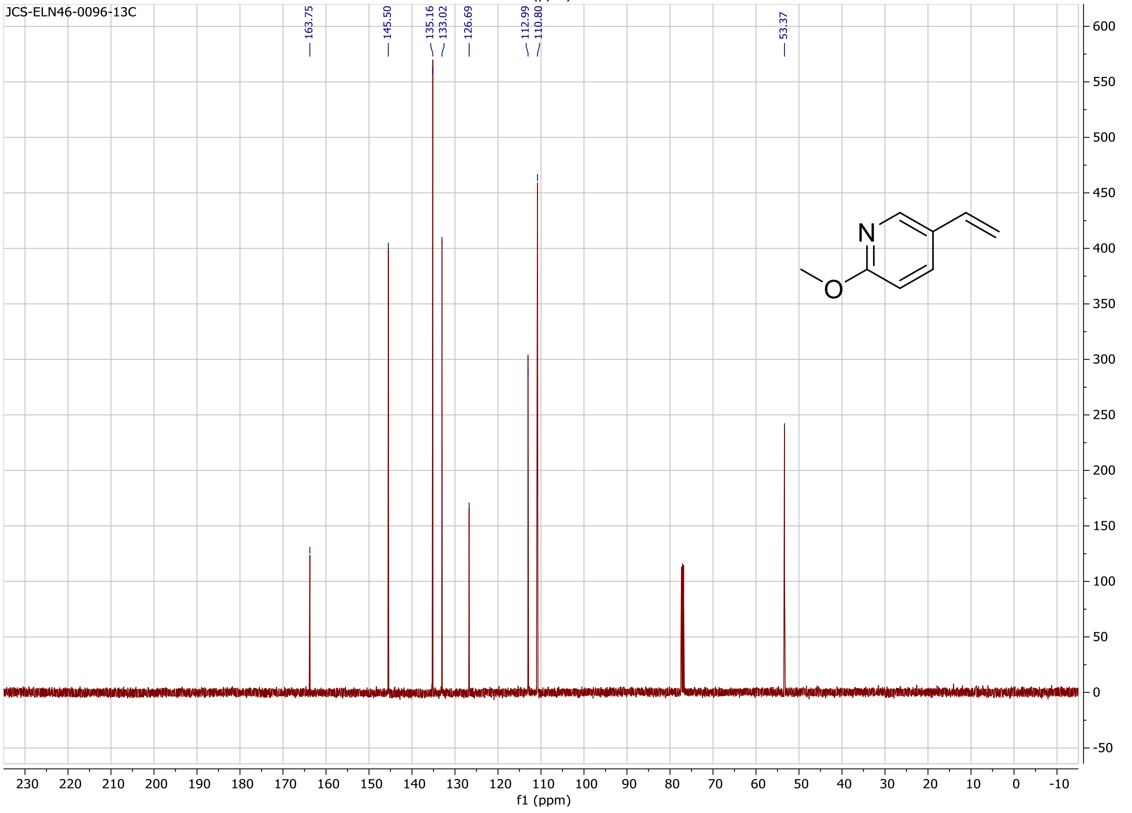
JCS-ELN46-0096-13C
163.75
145.50
135.16
133.02
126.69
112.99
110.80
53.37
N
O
f1 (ppm)

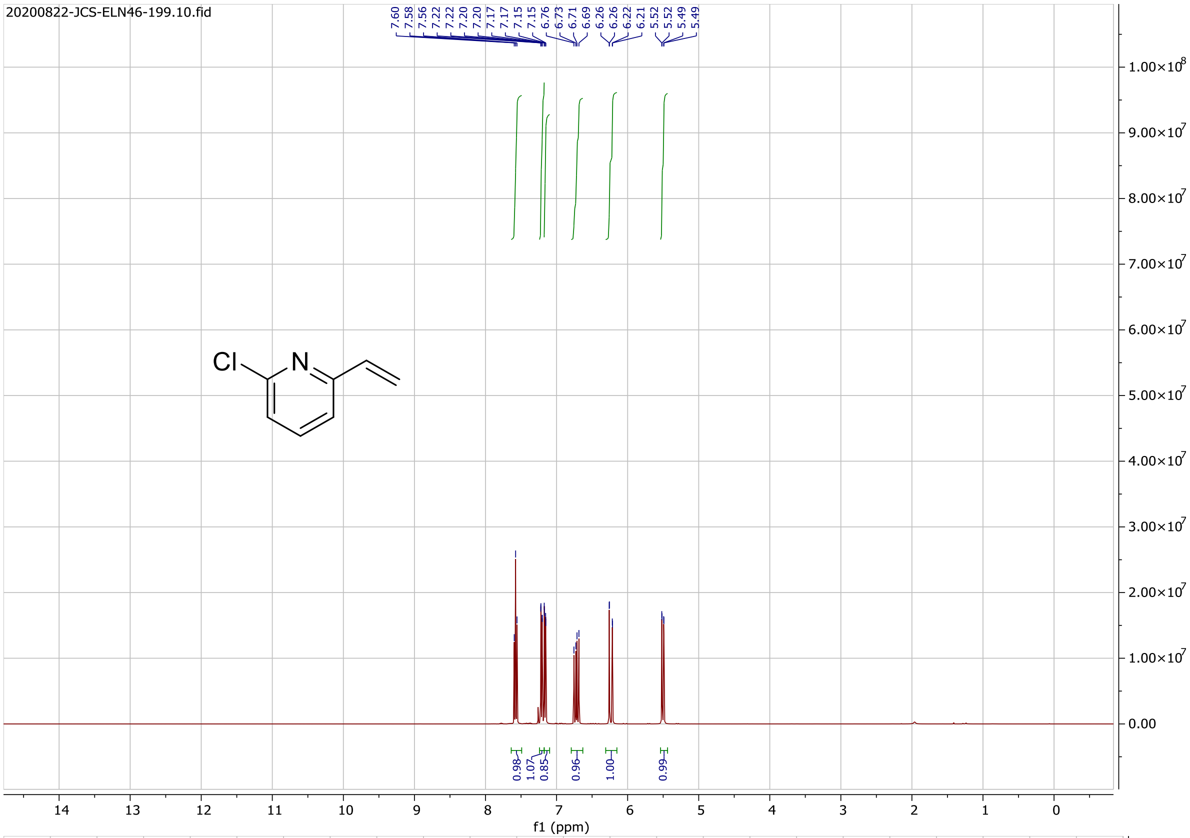
20200822-JCS-ELN46-199.10.fid
7.60
7.58
7.56
7.22
7.22
7.20
7.20
7.17
7.17
7.15
7.15
6.76
6.73
6.71
6.69
6.26
6.26
6.22
6.21
5.52
5.52
5.49
5.49
Cl
N
0.98
1.07
0.85
0.96
1.00
0.99
f1 (ppm)

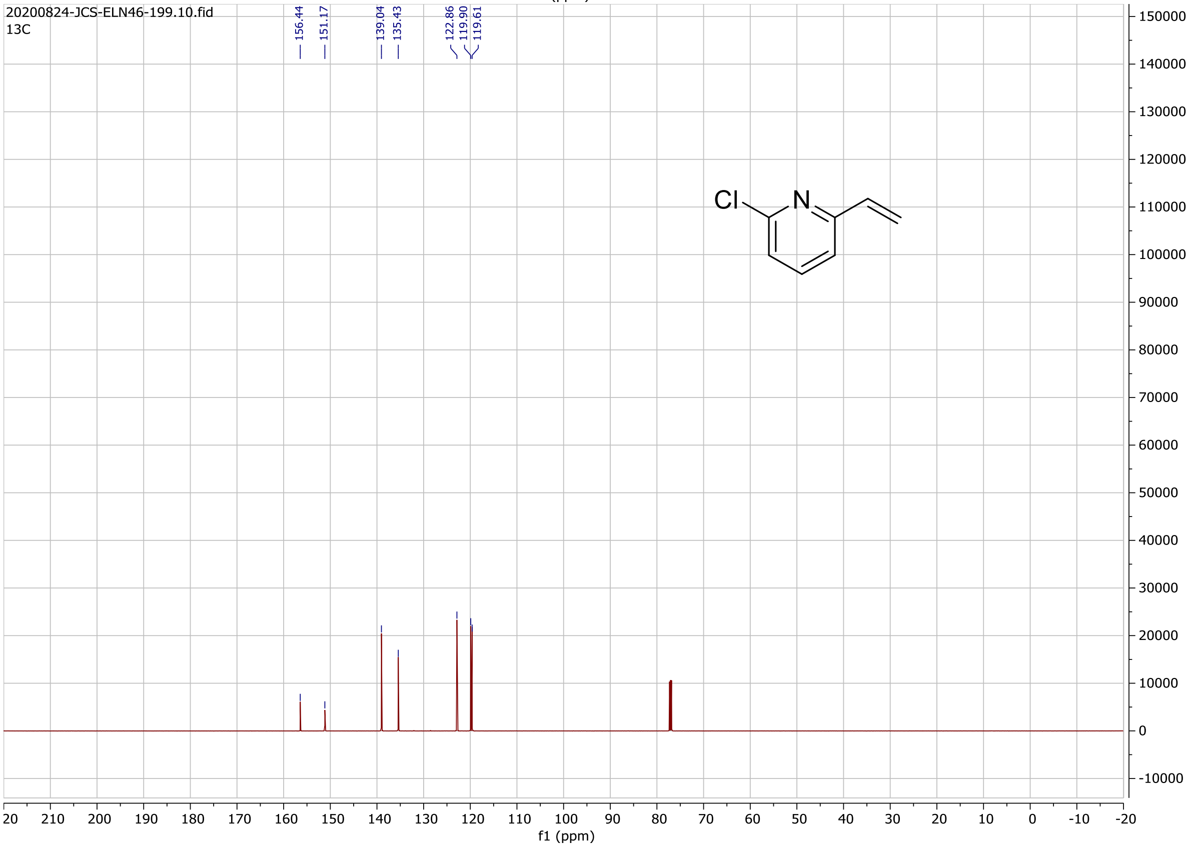
20200824-JCS-ELN46-199.10.fid
13C
156.44
151.17
139.04
135.43
122.86
119.90
119.61
Cl
N
f1 (ppm)

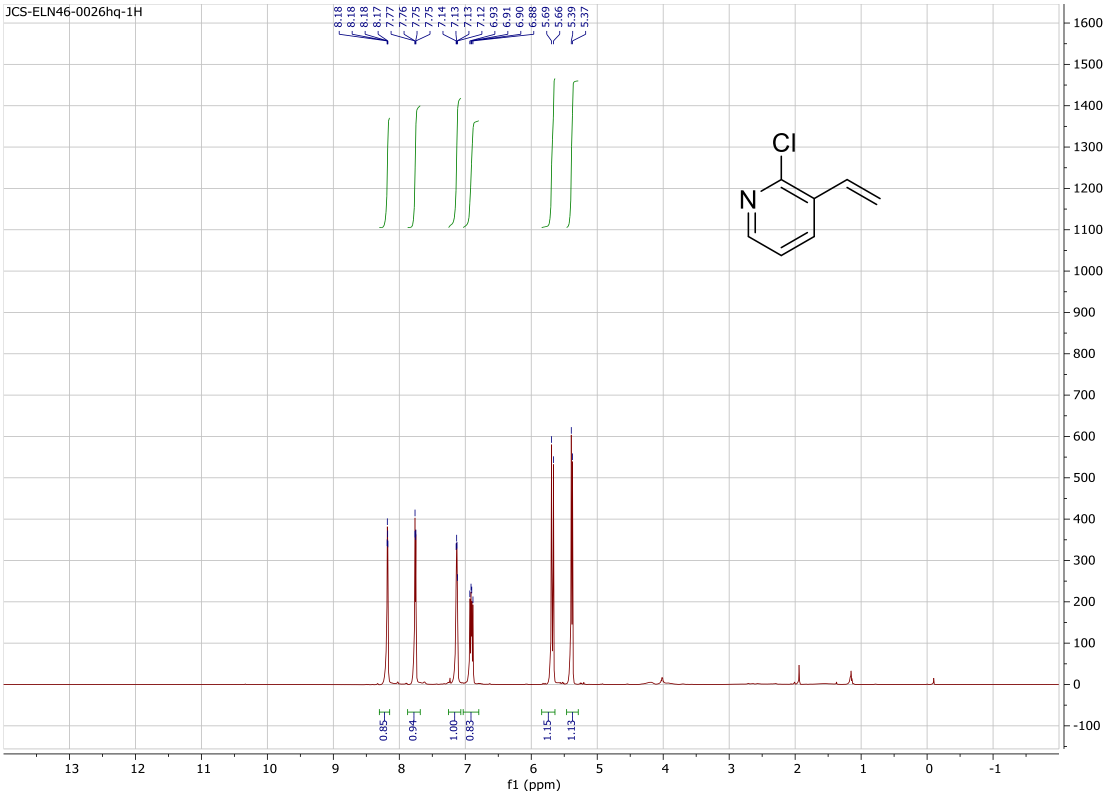
JCS-ELN46-0026hq-1H
8.18
8.18
8.18
8.17
7.77
7.76
7.75
7.75
7.14
7.13
7.13
7.12
6.93
6.91
6.90
6.88
5.69
5.66
5.39
5.37
Cl
N
0.85
0.94
1.00
0.83
1.15
1.13
f1 (ppm)

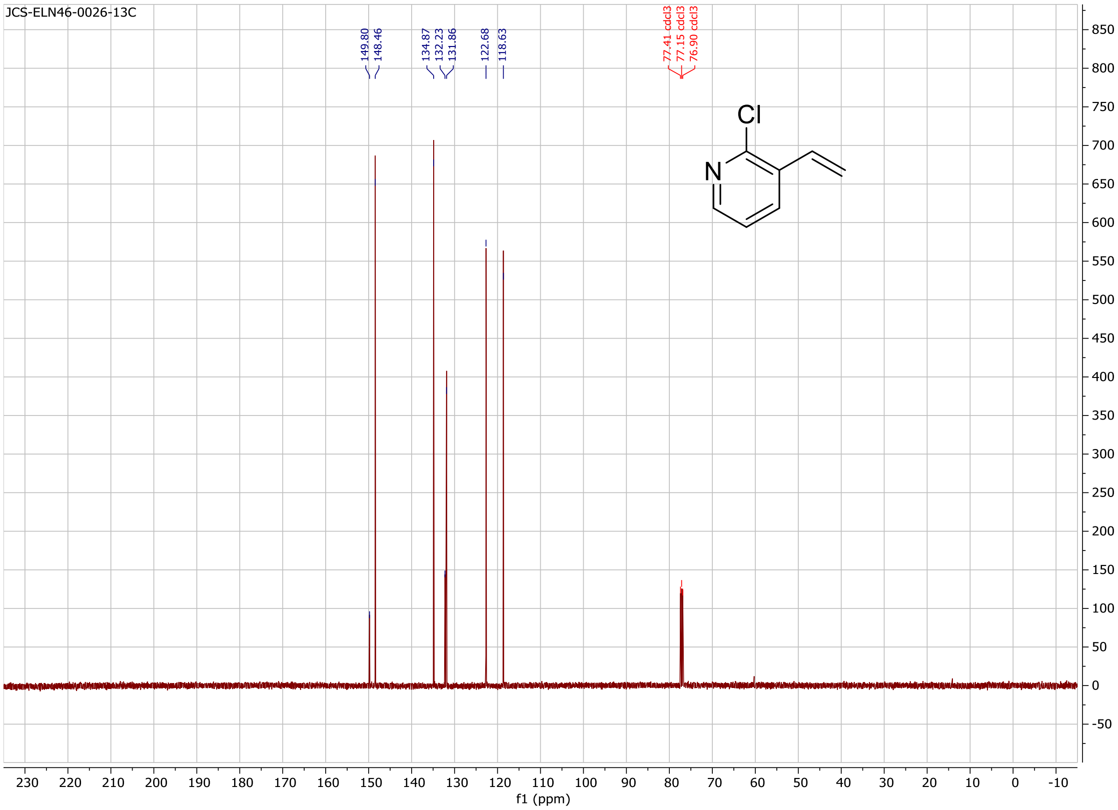
JCS-ELN46-0026-13C
149.80
148.46
134.87
132.23
131.86
122.68
118.63
77.41 cdcl3
77.15 cdcl3
76.90 cdcl3
Cl
N
f1 (ppm)

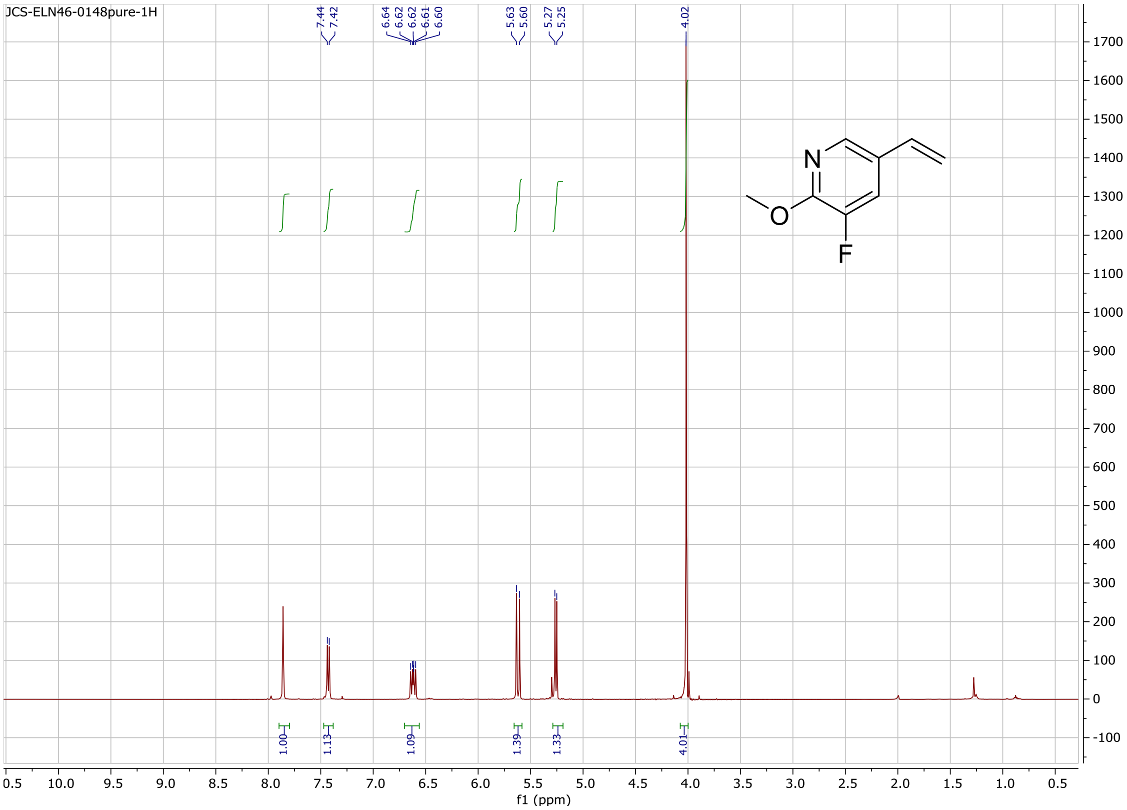
JCS-ELN46-0148pure-1H
7.44
7.42
6.64
6.62
6.62
6.61
6.60
5.63
5.60
5.27
5.25
4.02
1.00
1.13
1.09
1.39
1.33
4.01
N
O
F
f1 (ppm)

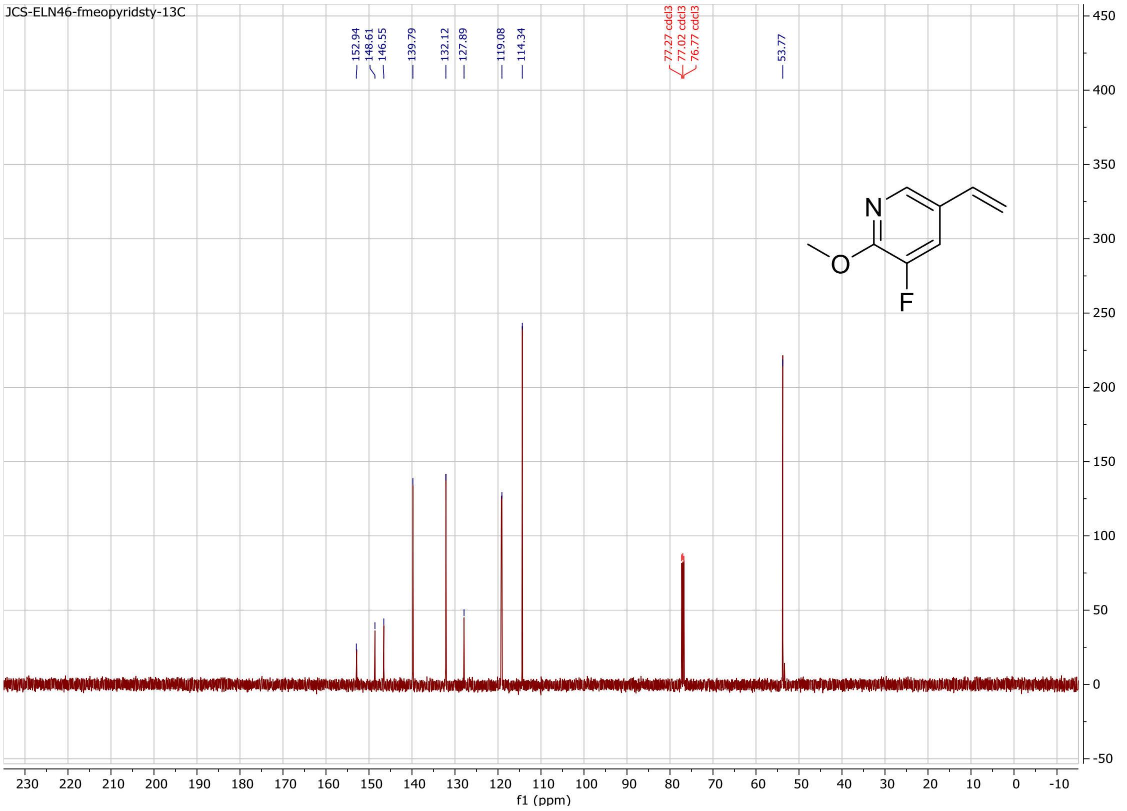
JCS-ELN46-fmeopyridsty-13C
152.94
148.61
146.55
139.79
132.12
127.89
119.08
114.34
77.27 cdcl3
77.02 cdcl3
76.77 cdcl3
53.77
N
O
F
f1 (ppm)

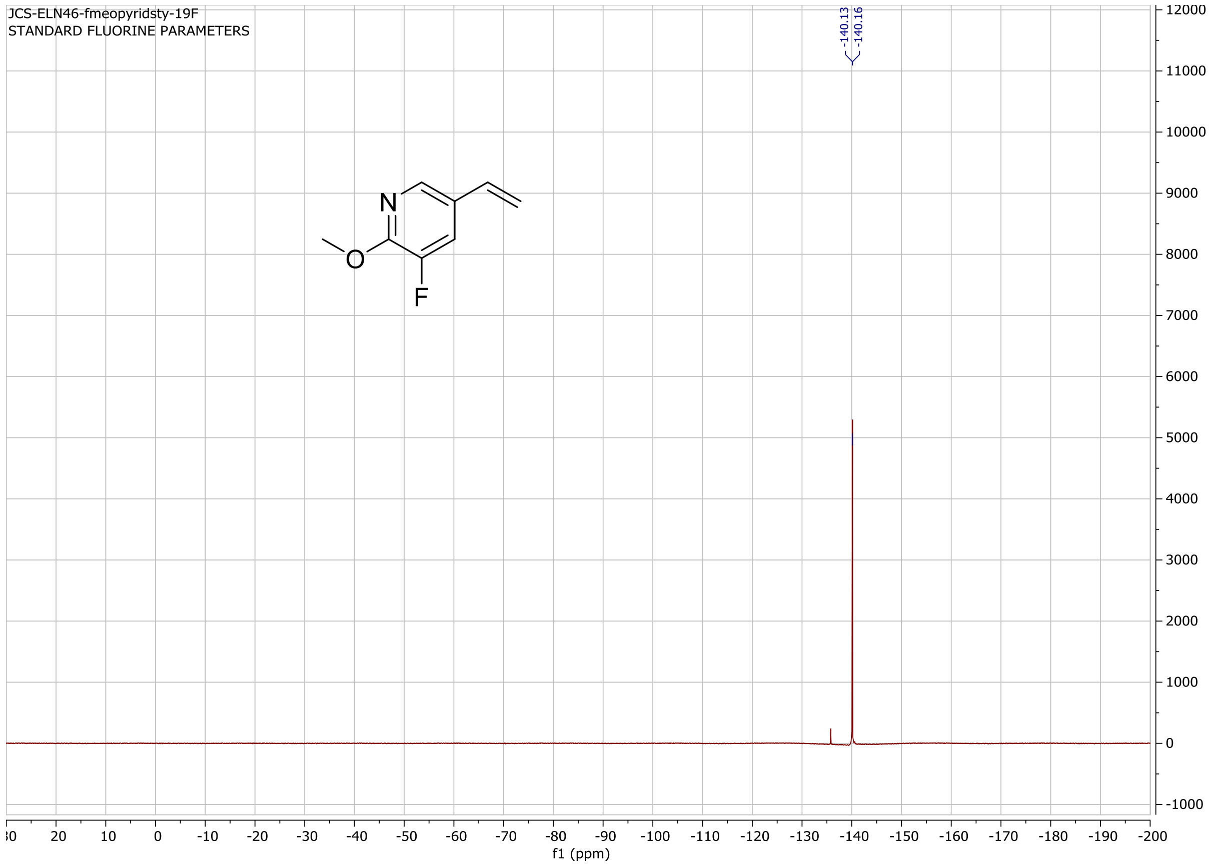
JCS-ELN46-fmeopyridsty-19F
STANDARD FLUORINE PARAMETERS
-140.13
-140.16
N
O
F
f1 (ppm)

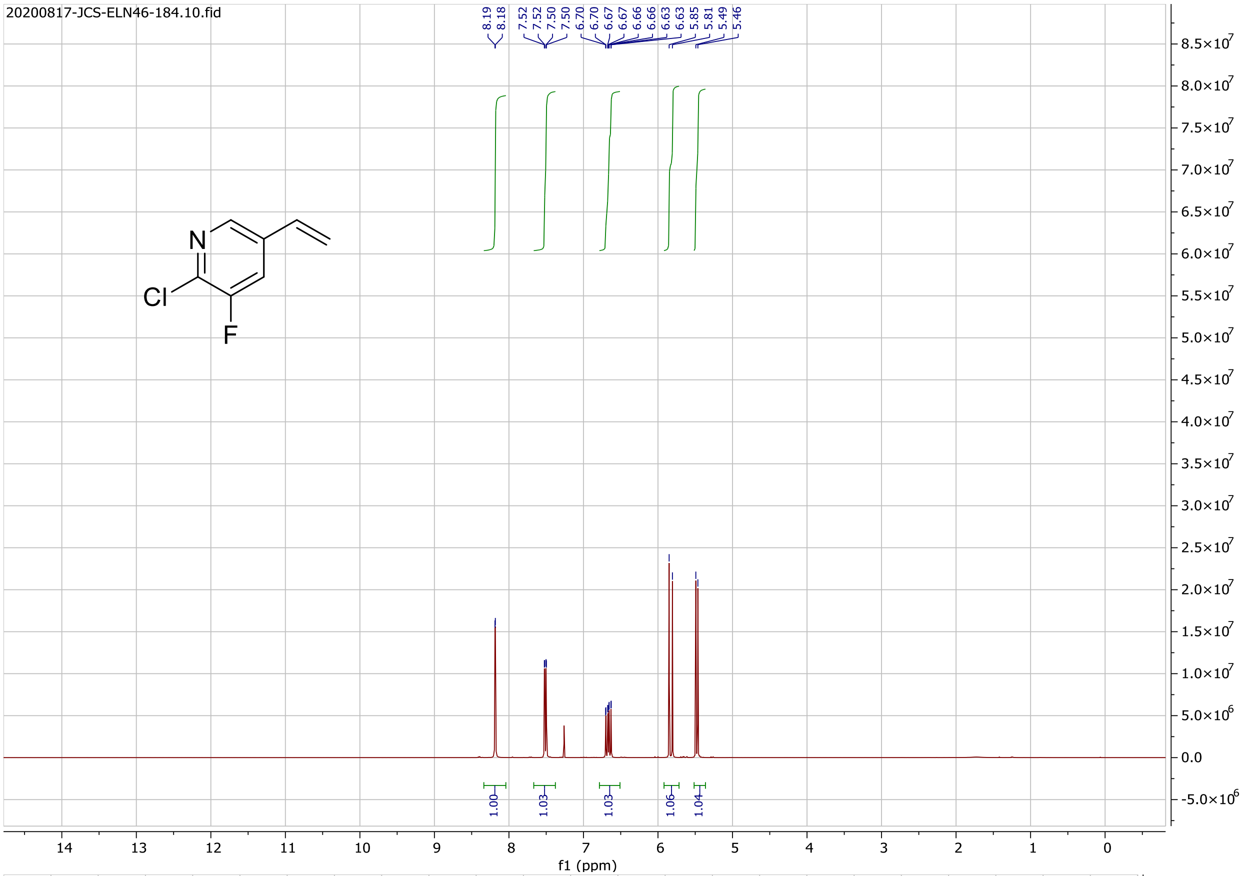
20200817-JCS-ELN46-184.10.fid
8.19
8.18
7.52
7.52
7.50
7.50
6.70
6.70
6.67
6.67
6.66
6.66
6.63
6.63
5.85
5.81
5.49
5.46
N
Cl
F
1.00
1.03
1.03
1.06
1.04
f1 (ppm)

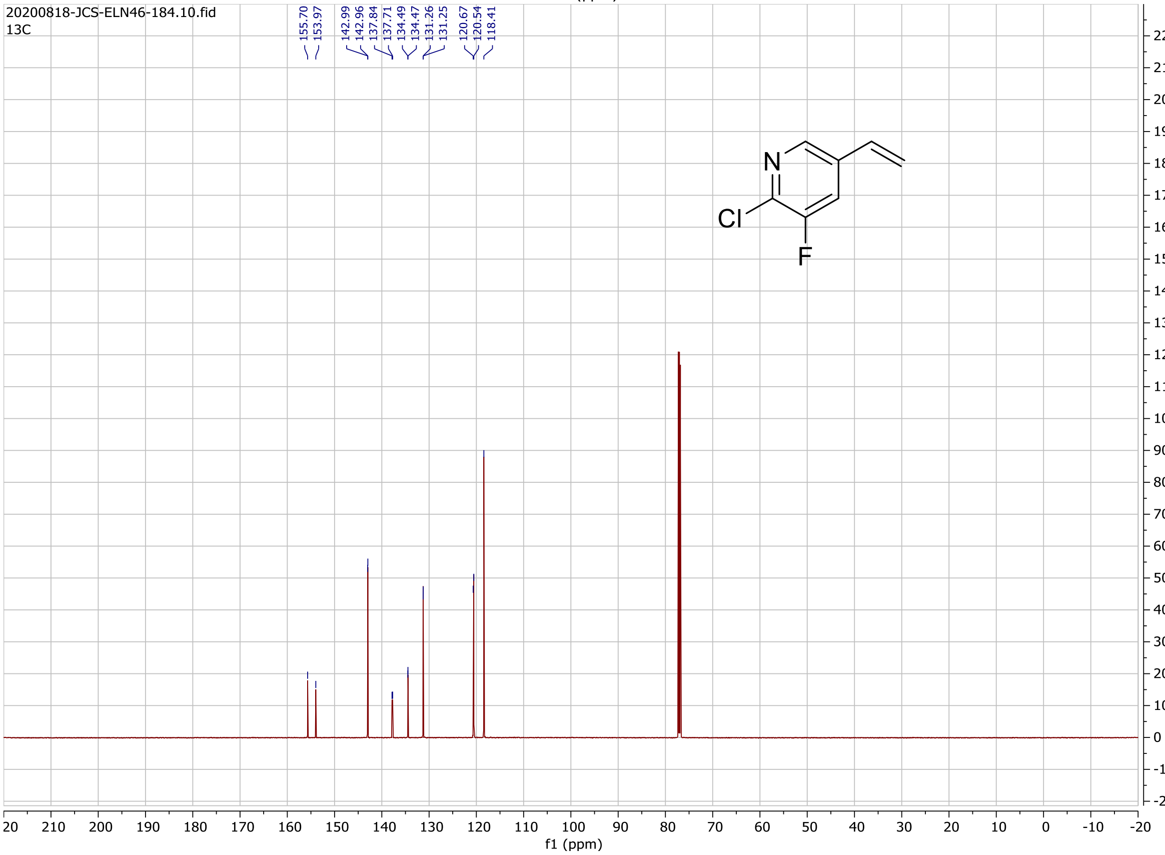
20200818-JCS-ELN46-184.10.fid
13C
155.70
153.97
142.99
142.96
137.84
137.71
134.49
134.47
131.26
131.25
120.67
120.54
118.41
N
Cl
F
f1 (ppm)

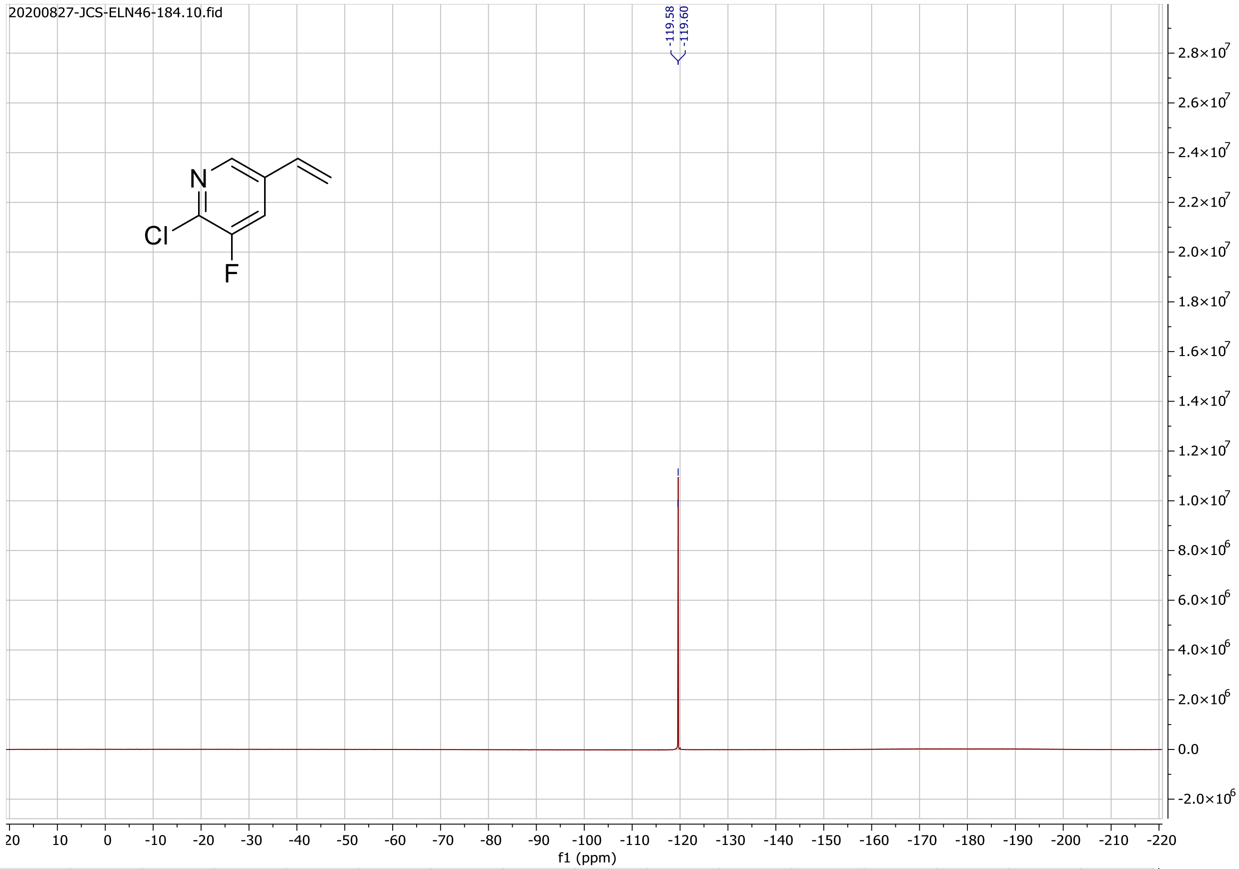
20200827-JCS-ELN46-184.10.fid
-119.58
-119.60
N
Cl
F
f1 (ppm)

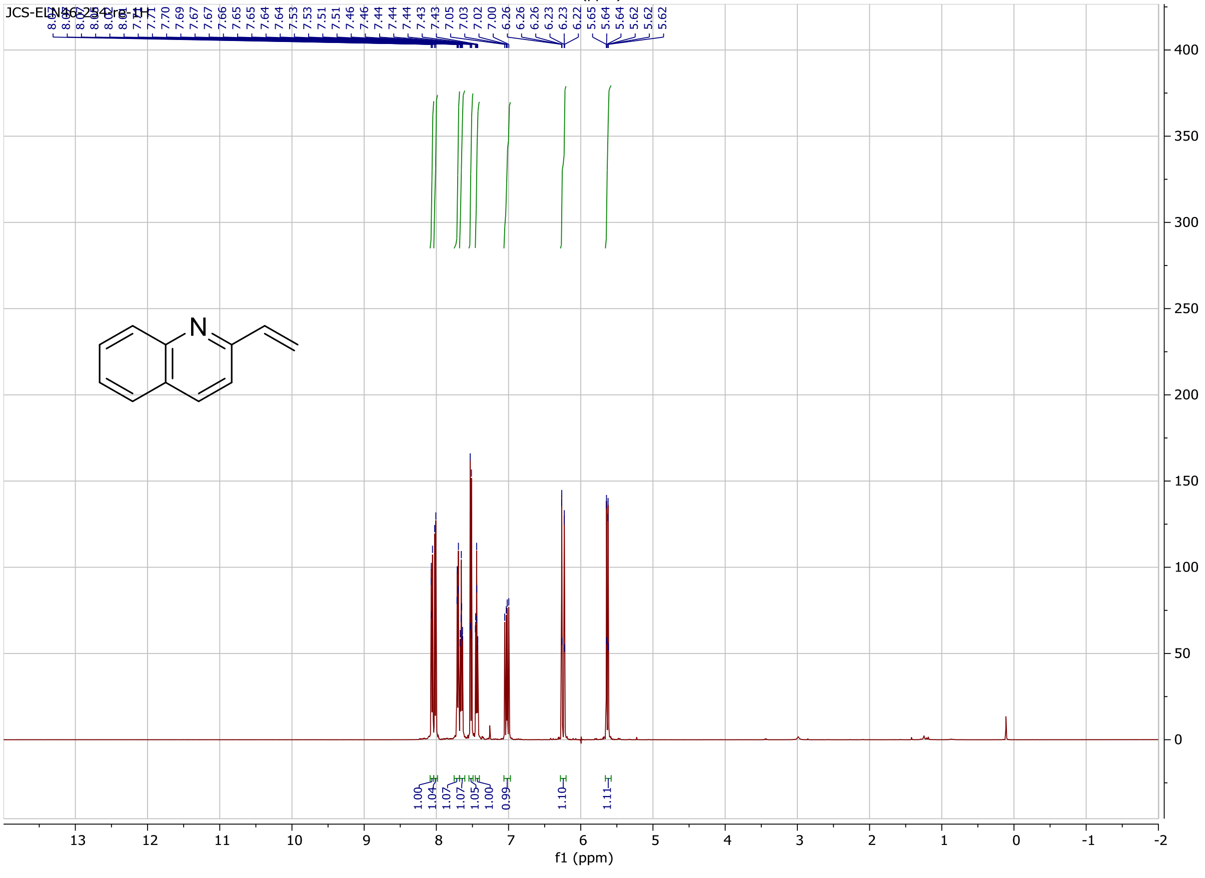
JCS-ELN46-254.r-1H
N
f1 (ppm)

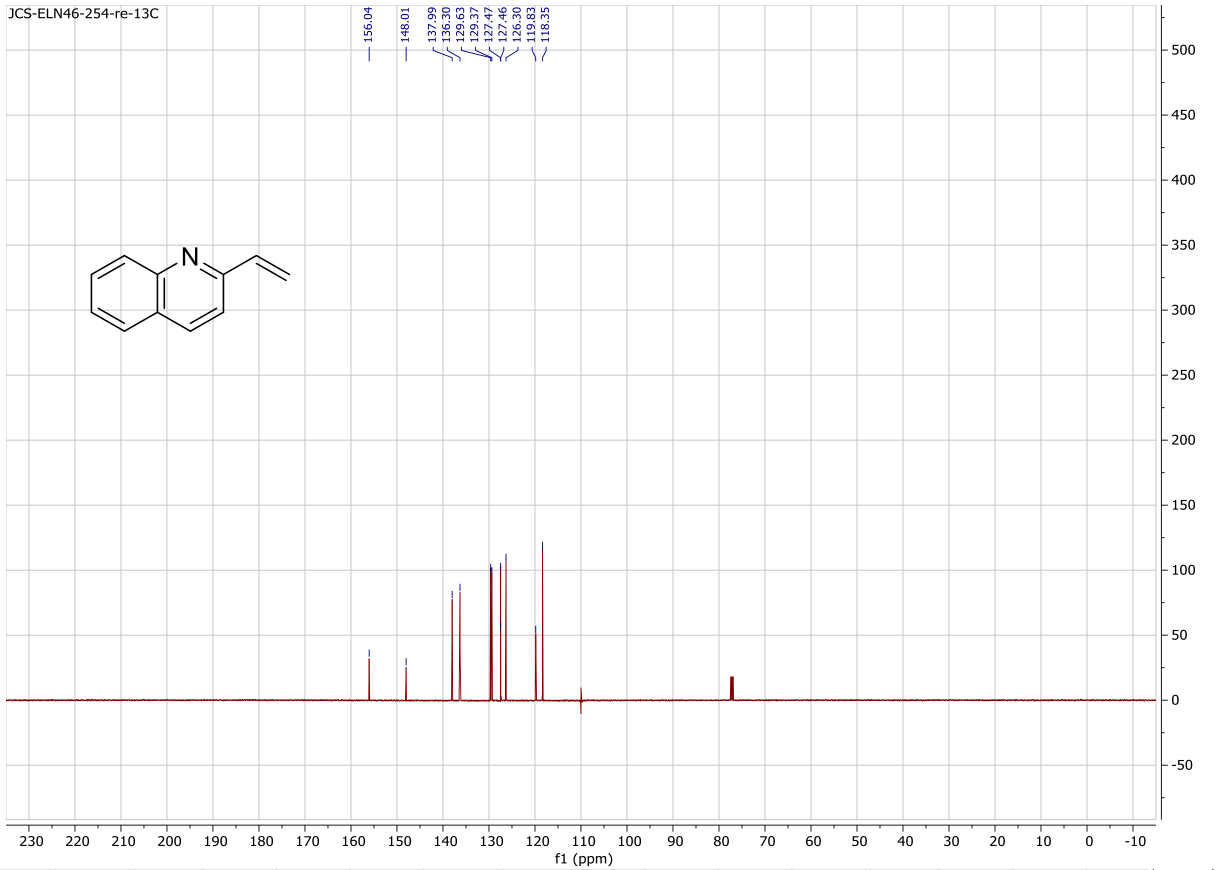

JCS-ELN46-254-re-13C
156.04
148.01
137.99
136.30
129.63
129.37
127.47
127.46
126.30
119.83
118.35
N
f1 (ppm)

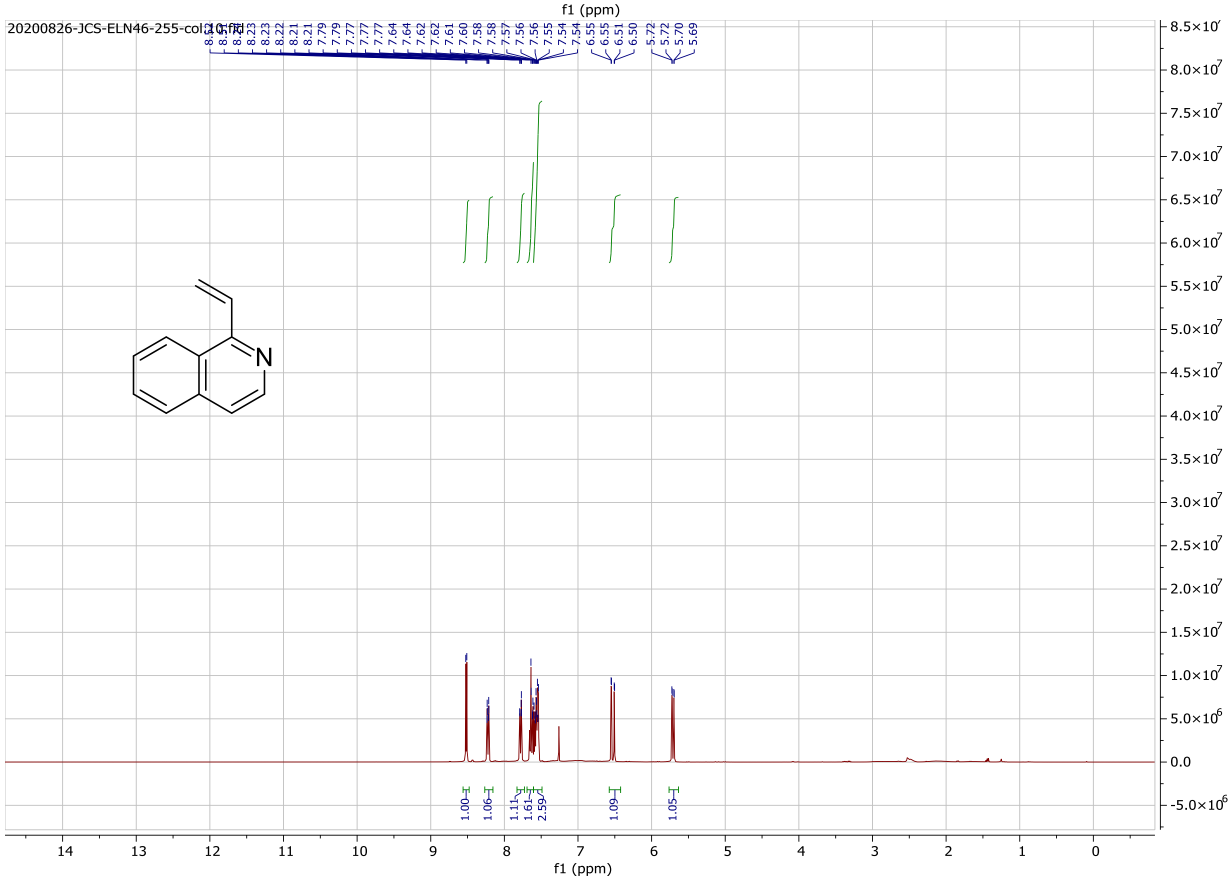

20200826-JCS-ELN46-255-col10.fid
8.23
8.23
8.22
8.21
7.79
7.79
7.77
7.77
7.64
7.62
7.61
7.60
7.58
7.57
7.56
7.55
7.54
6.55
6.55
6.51
6.50
5.72
5.72
5.70
5.69
N
1.00
1.06
1.11
1.61
2.59
1.09
1.05
f1 (ppm)

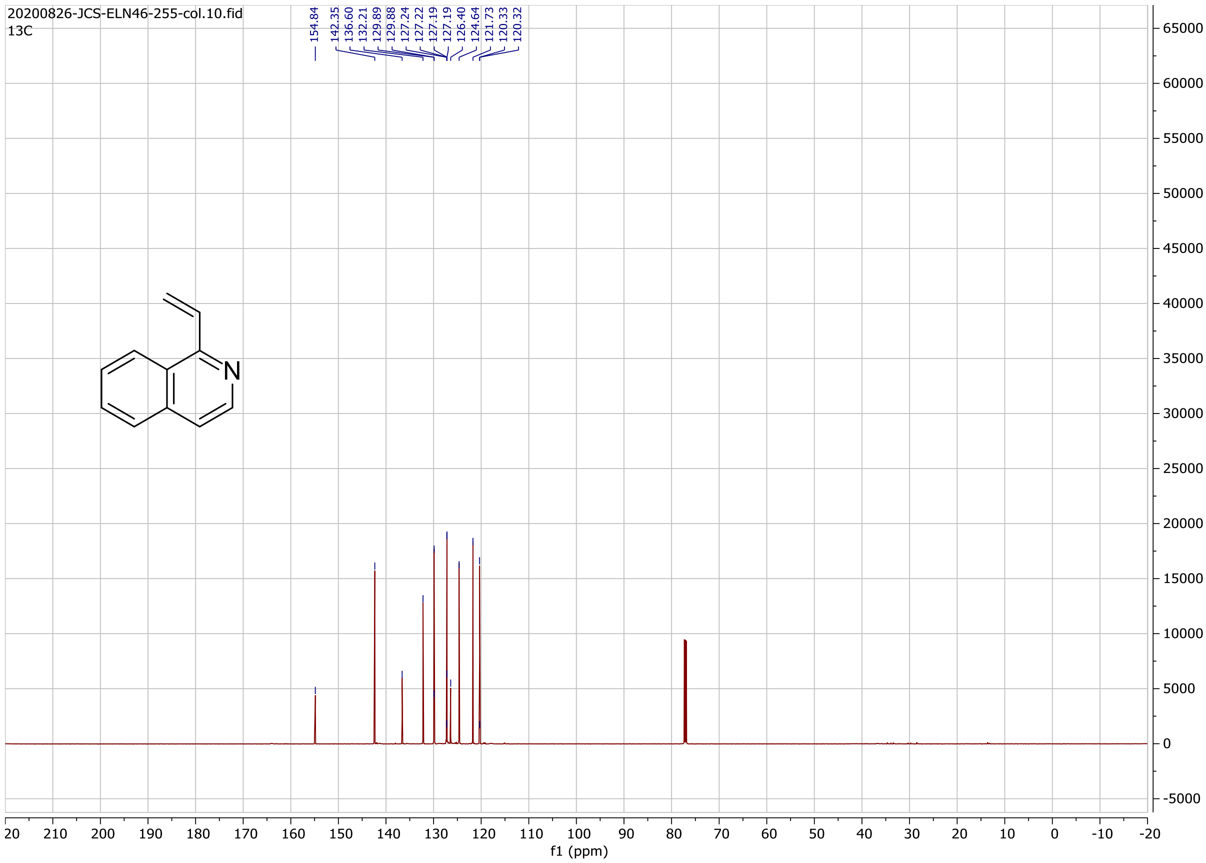
20200826-JCS-ELN46-255-col.10.fid
13C
154.84
142.35
136.60
132.21
129.89
129.88
127.24
127.22
127.19
127.19
126.40
124.64
121.73
120.33
120.32
N
f1 (ppm)

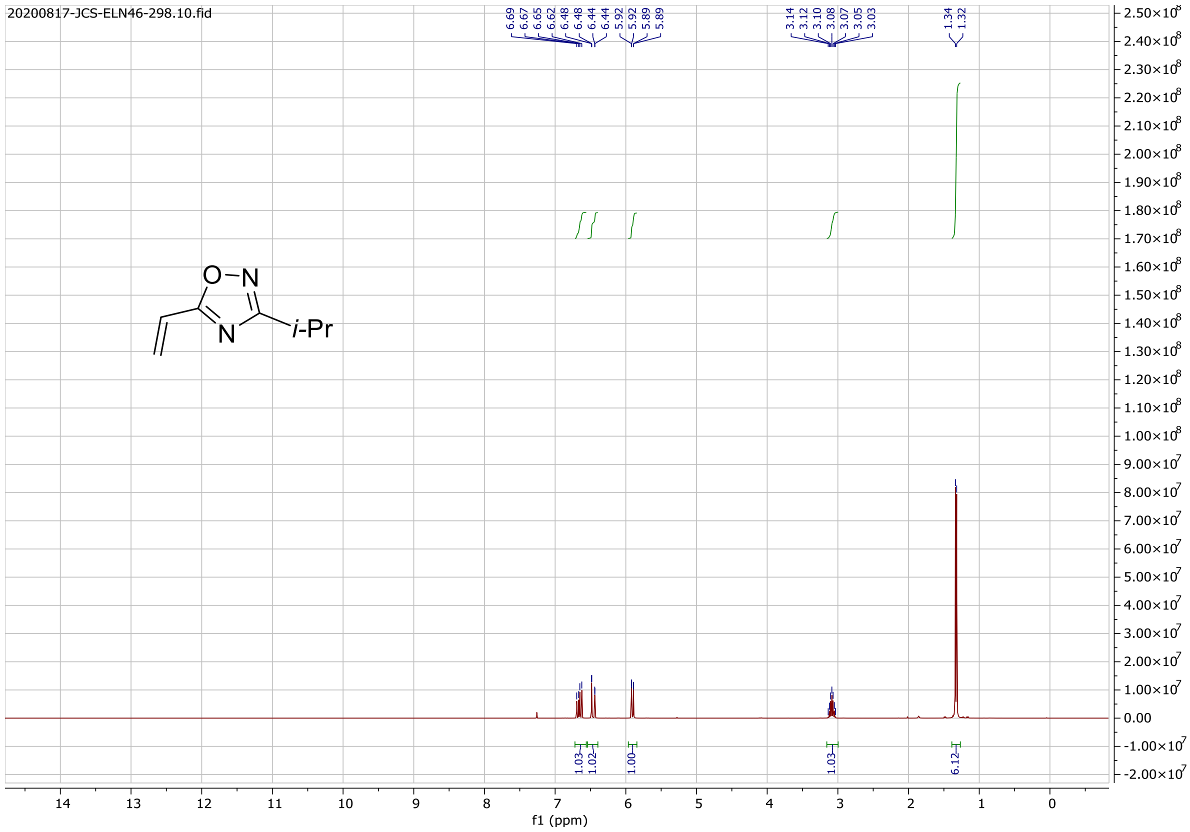
20200817-JCS-ELN46-298.10.fid
6.69
6.67
6.65
6.62
6.48
6.44
6.44
5.92
5.89
5.89
3.14
3.12
3.10
3.08
3.07
3.05
3.03
1.34
1.32
O
N
N
i-Pr
1.03
1.02
1.00
1.03
6.12
f1 (ppm)

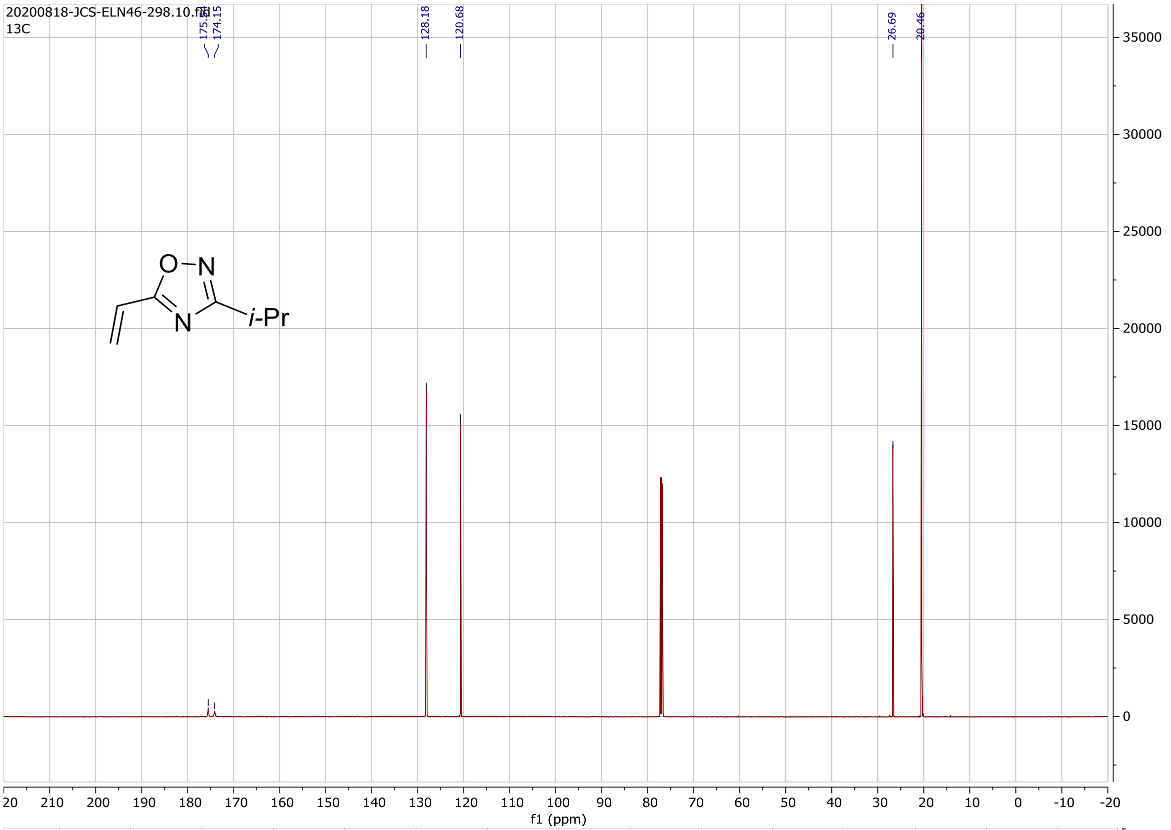
20200818-JCS-ELN46-298.10.fid
13C
175.61
174.15
128.18
120.68
26.69
20.46
O
N
N
i-Pr
f1 (ppm)

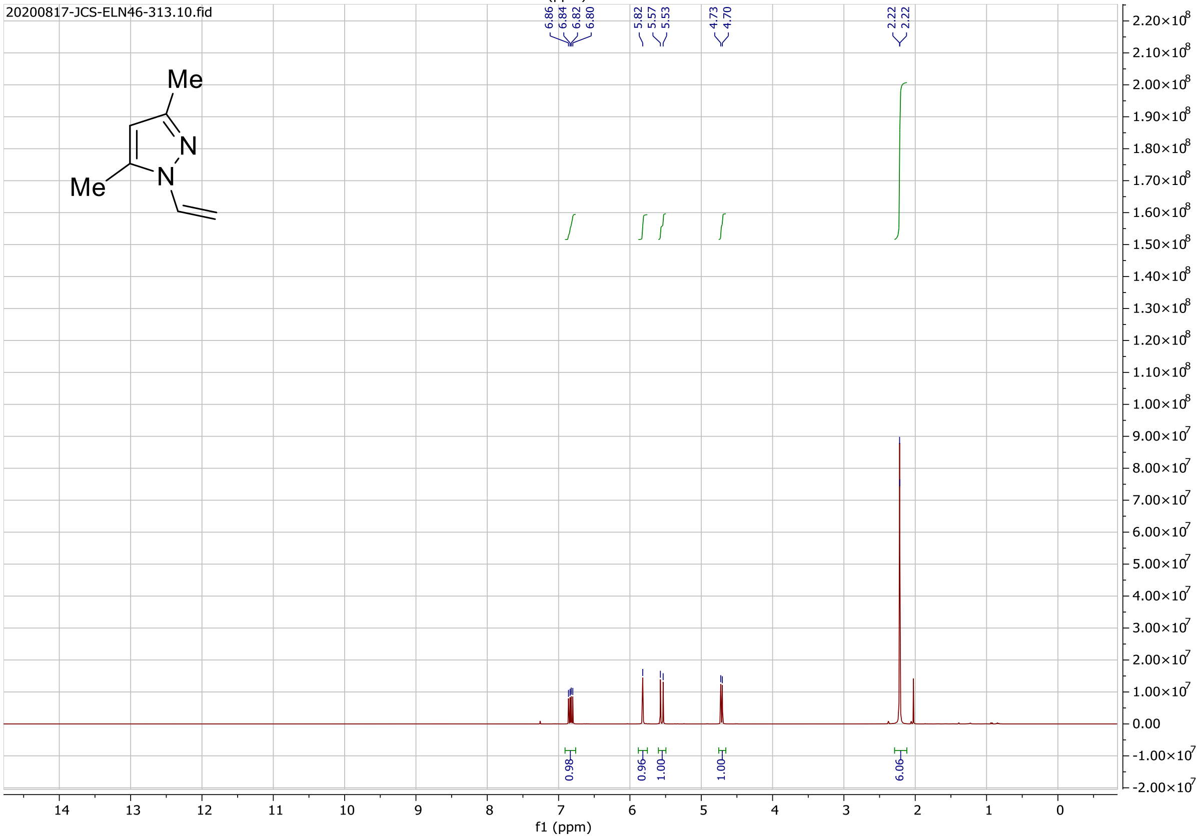
20200817-JCS-ELN46-313.10.fid
6.86
6.84
6.82
6.80
5.82
5.57
5.53
4.73
4.70
2.22
2.22
Me
N
N
Me
0.98
0.96
1.00
1.00
6.06
f1 (ppm)

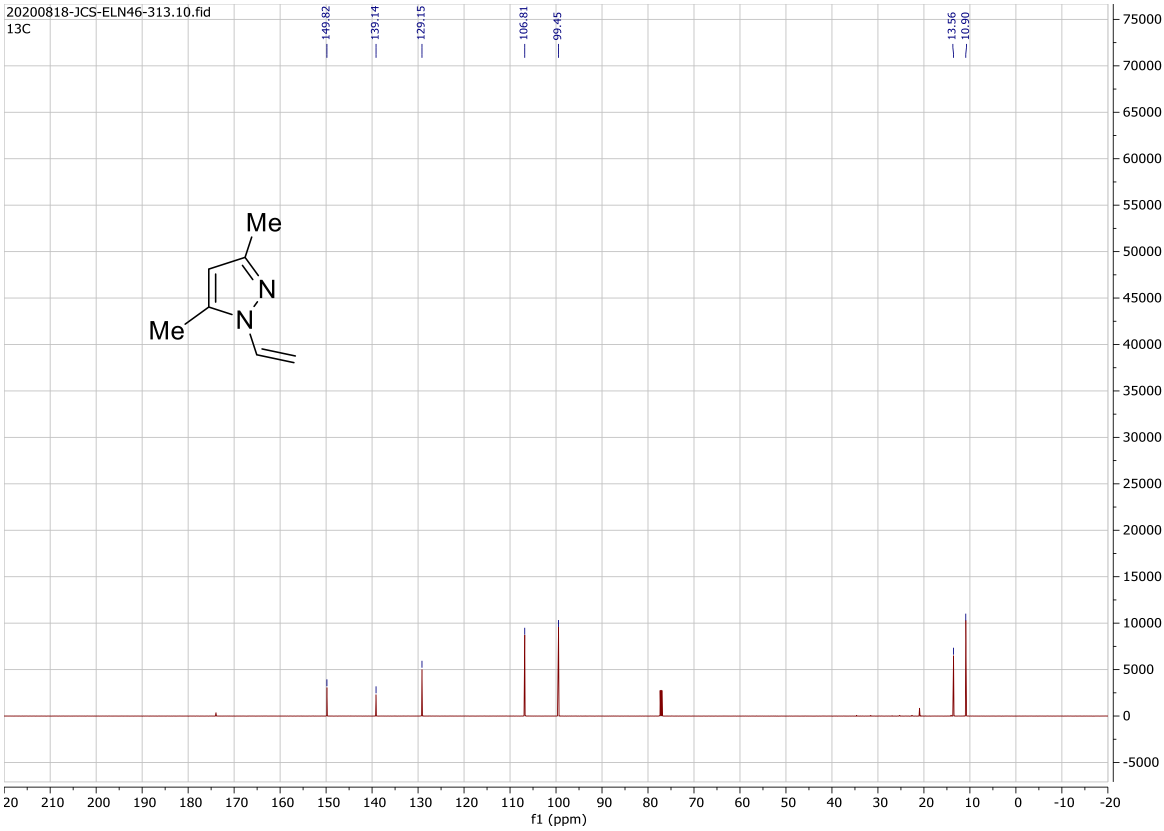

20200818-JCS-ELN46-313.10.fid
13C
149.82
139.14
129.15
106.81
99.45
13.56
10.90
Me
N
N
Me
f1 (ppm)

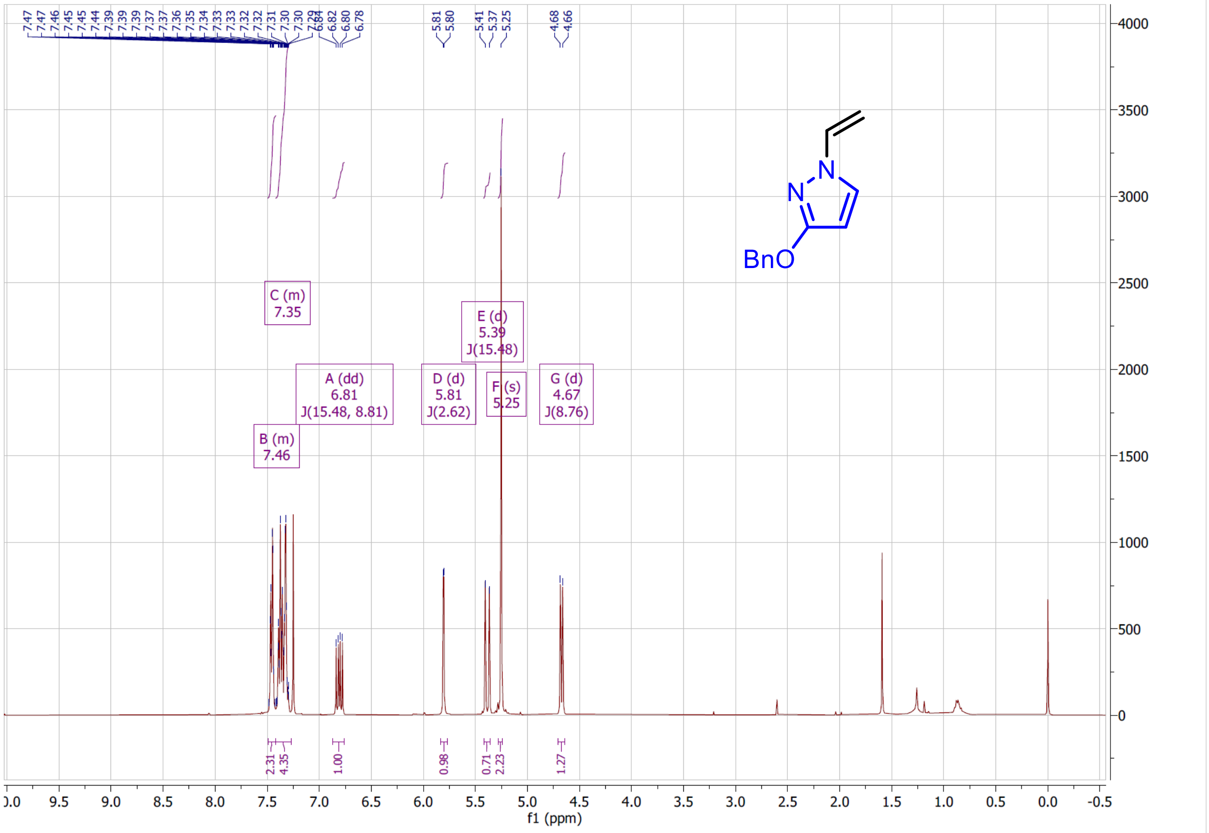

BnO
C (m)
7.35
E (d)
5.39
J(15.48)
A (dd)
6.81
J(15.48, 8.81)
D (d)
5.81
J(2.62)
F (s)
5.25
G (d)
4.67
J(8.76)
B (m)
7.46
f1 (ppm)

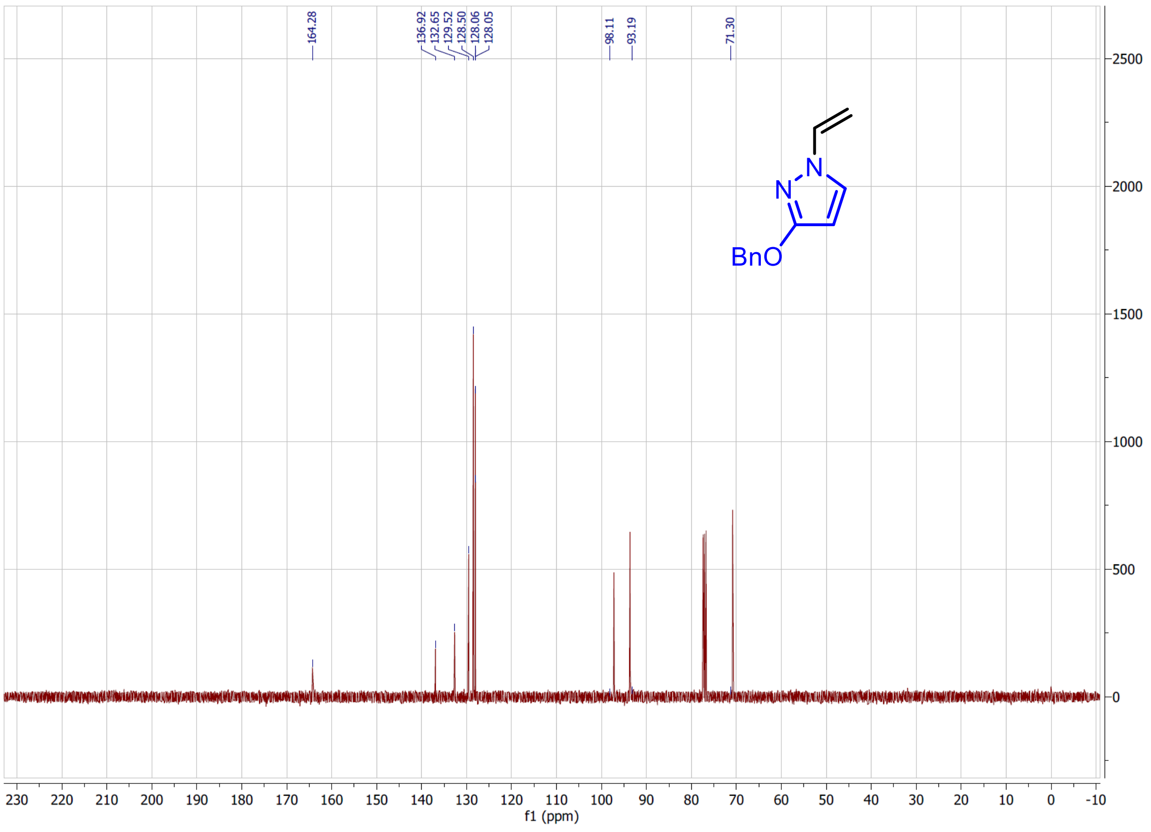

164.28
136.92
132.65
129.52
128.50
128.06
128.05
98.11
93.19
71.30
BnO
f1 (ppm)

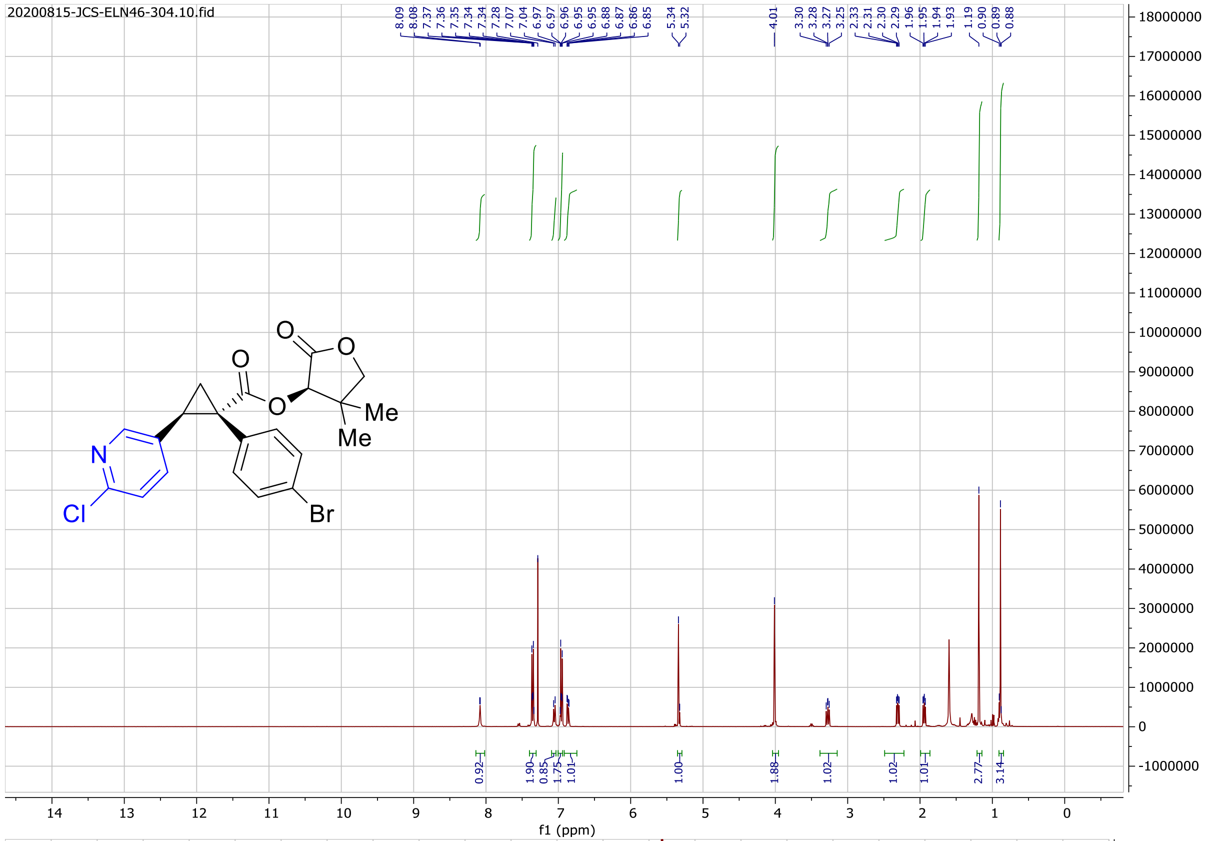
20200815-JCS-ELN46-304.10.fid
8.09
8.08
7.37
7.36
7.35
7.34
7.34
7.28
7.07
7.04
6.97
6.97
6.96
6.95
6.95
6.88
6.87
6.86
6.85
5.34
5.32
4.01
3.30
3.28
3.27
3.25
2.33
2.31
2.30
2.29
1.96
1.95
1.94
1.93
1.19
0.90
0.89
0.88
0.92
1.90
0.85
1.75
1.01
1.00
1.88
1.02
1.02
1.01
2.77
3.14
O
O
O
O
Me
Me
N
Cl
Br
f1 (ppm)

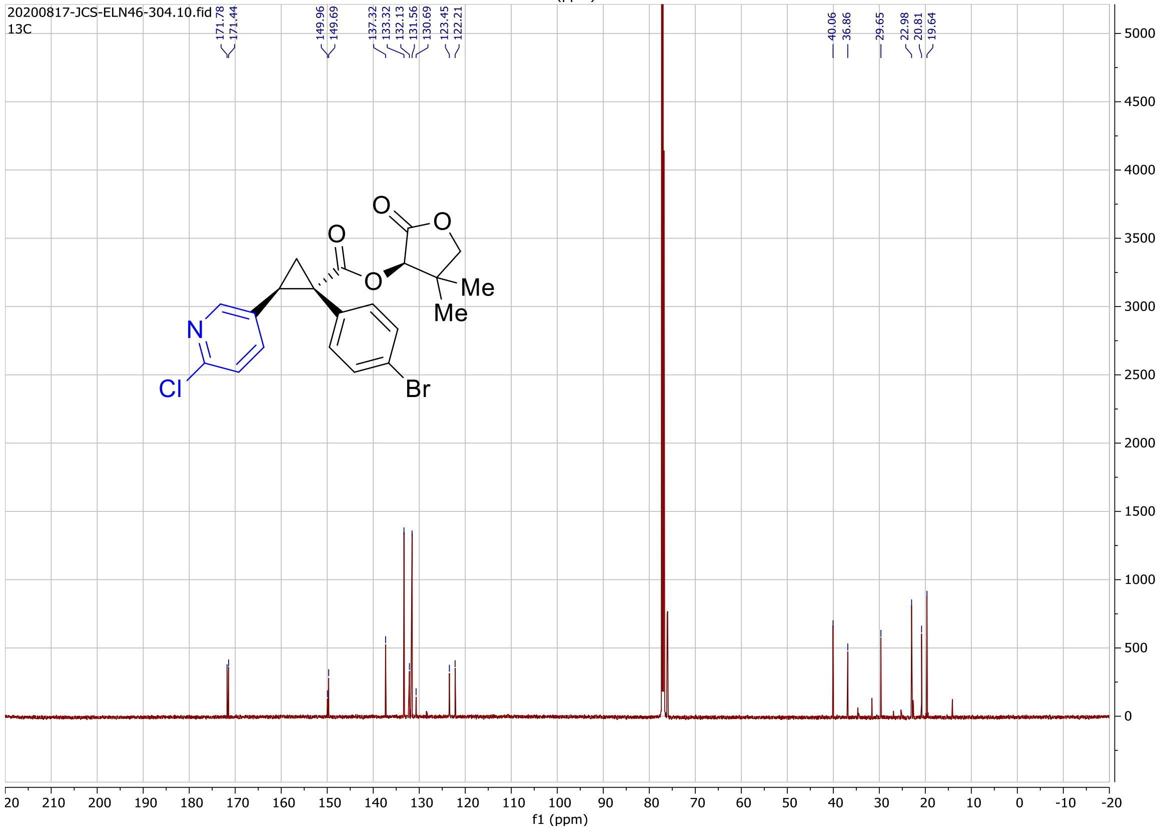
20200817-JCS-ELN46-304.10.fid
13C
171.78
171.44
149.96
149.69
137.32
133.32
132.13
131.56
130.69
123.45
122.21
40.06
36.86
29.65
22.98
20.81
19.64
O
O
O
O
Me
Me
N
Cl
Br
f1 (ppm)

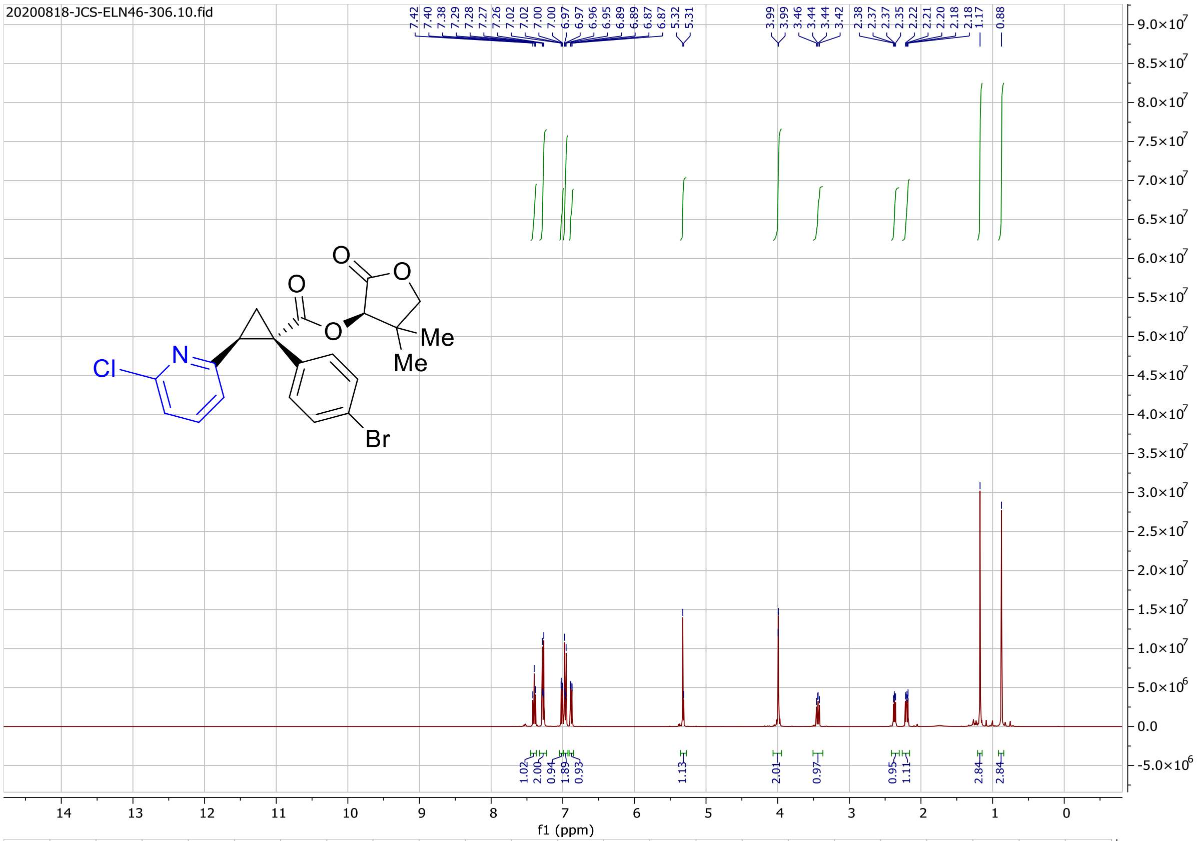
20200818-JCS-ELN46-306.10.fid
7.42
7.40
7.38
7.29
7.28
7.27
7.26
7.02
7.02
7.00
7.00
6.97
6.97
6.96
6.95
6.89
6.89
6.87
6.87
5.32
5.31
3.99
3.99
3.46
3.44
3.44
3.42
2.38
2.37
2.37
2.35
2.22
2.21
2.20
2.18
1.17
0.88
1.02
2.00
0.94
1.89
0.93
1.13
2.01
0.97
0.95
1.11
2.84
2.84
O
O
O
O
Cl
N
Me
Me
Br
f1 (ppm)

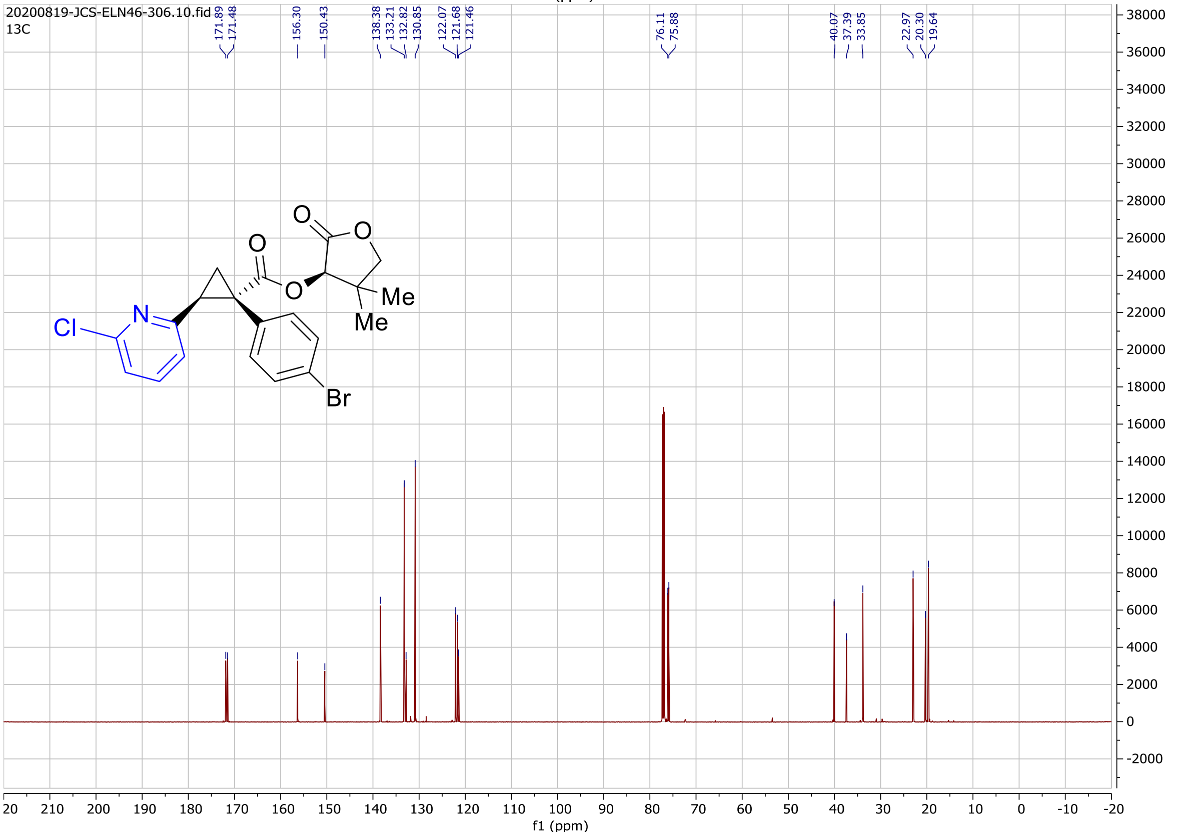
20200819-JCS-ELN46-306.10.fid
13C
171.89
171.48
156.30
150.43
138.38
133.21
132.82
130.85
122.07
121.68
121.46
76.11
75.88
40.07
37.39
33.85
22.97
20.30
19.64
O
O
O
O
Cl
N
Me
Me
Br
f1 (ppm)

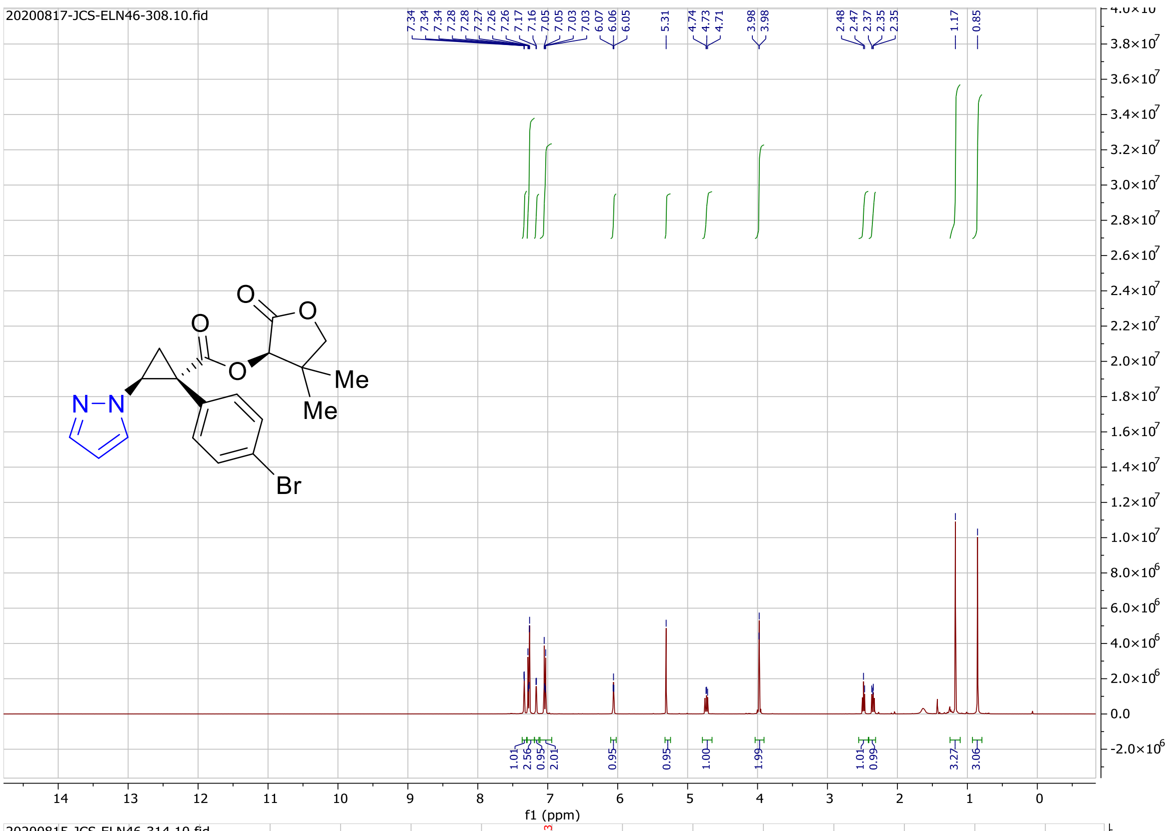

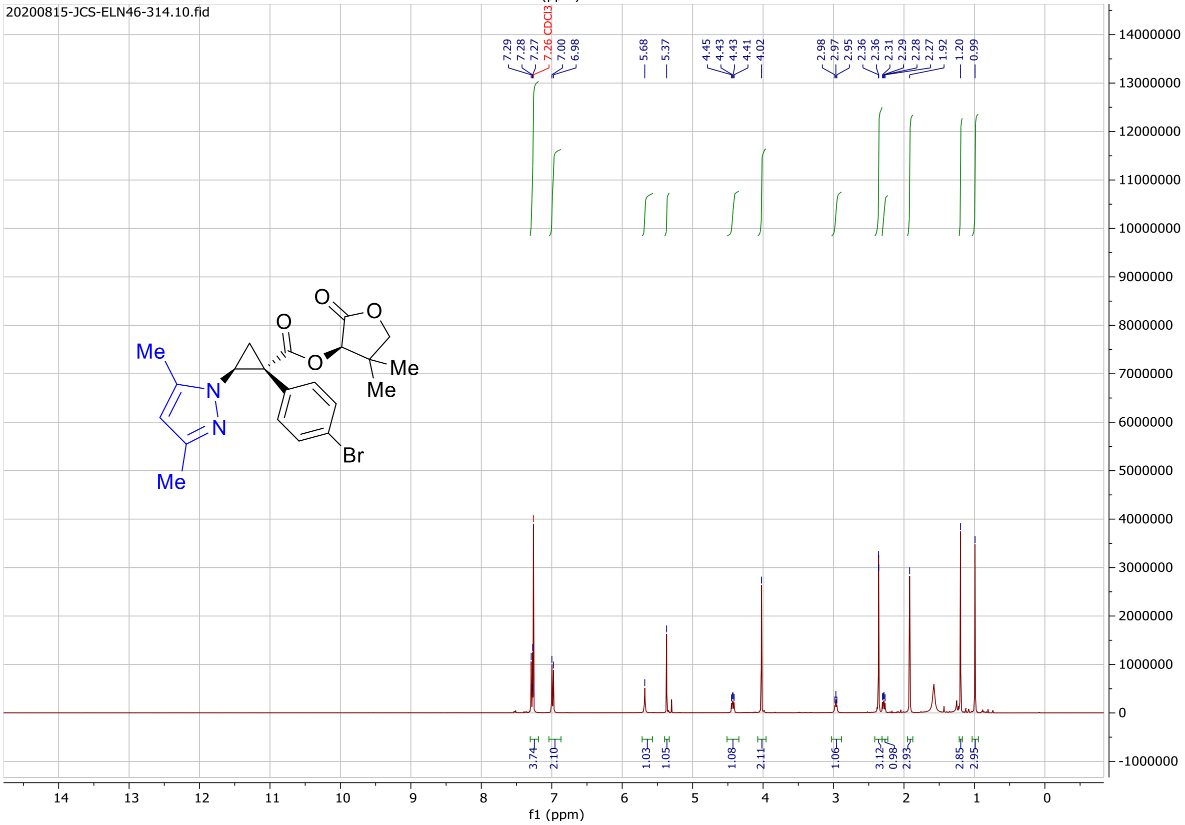

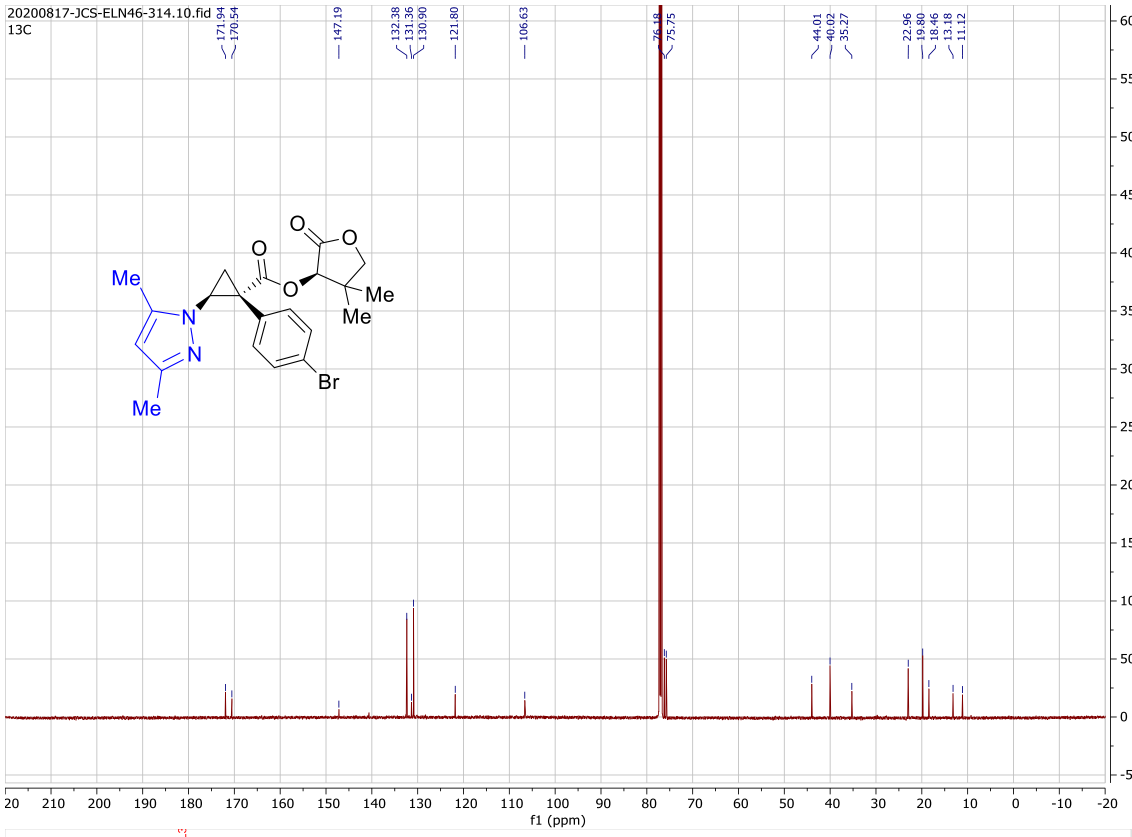
20200817-JCS-ELN46-314.10.fid
13C
f1 (ppm)

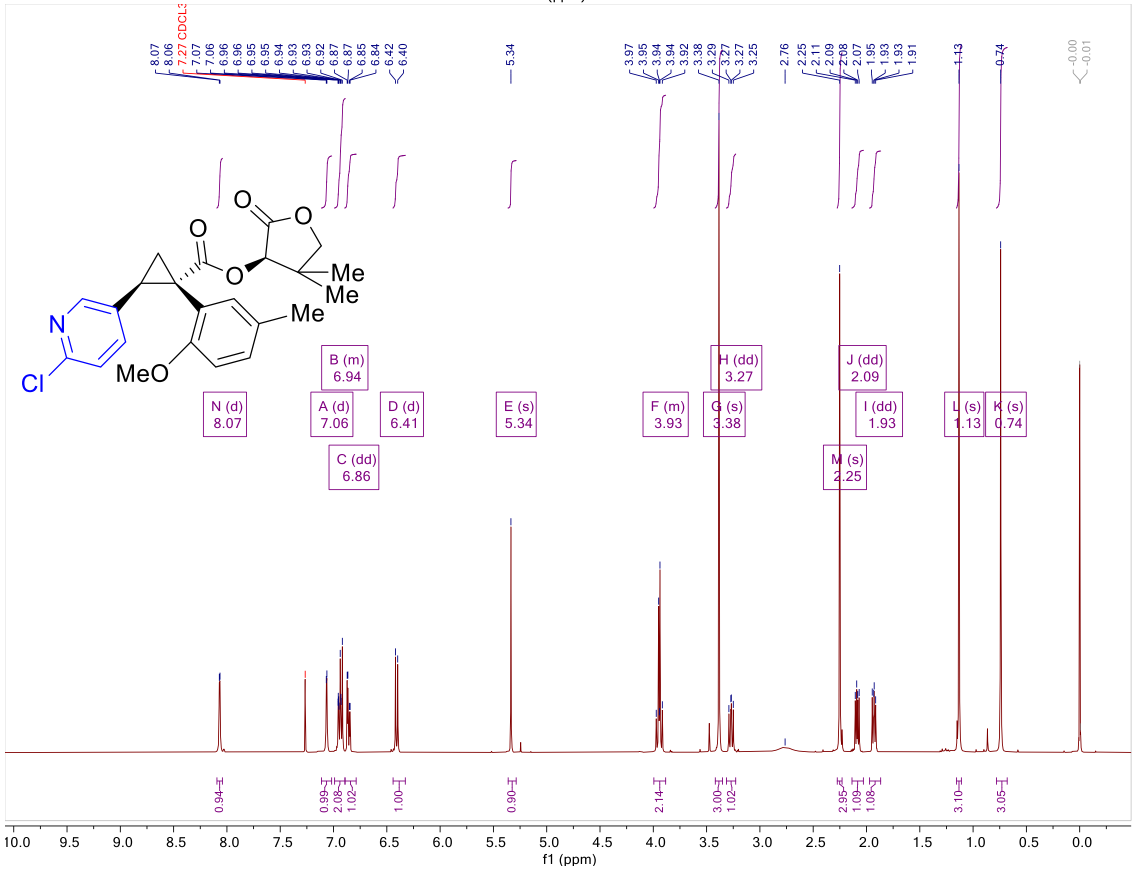
f1 (ppm)

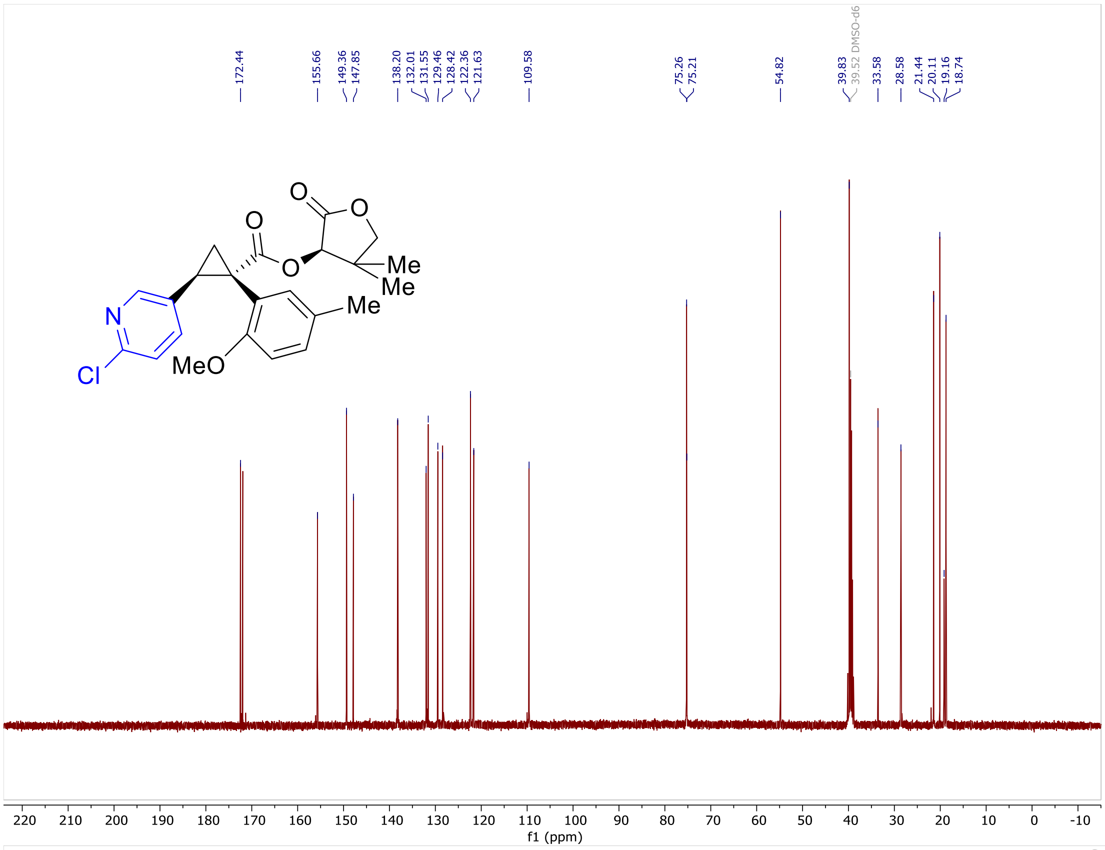

172.44
155.66
149.36
147.85
138.20
132.01
131.55
129.46
128.42
122.36
121.63
109.58
75.26
75.21
54.82
39.83
39.52 DMSO-d6
33.58
28.58
21.44
20.11
19.16
18.74
O
O
O
O
N
Cl
MeO
Me
Me
Me
f1 (ppm)

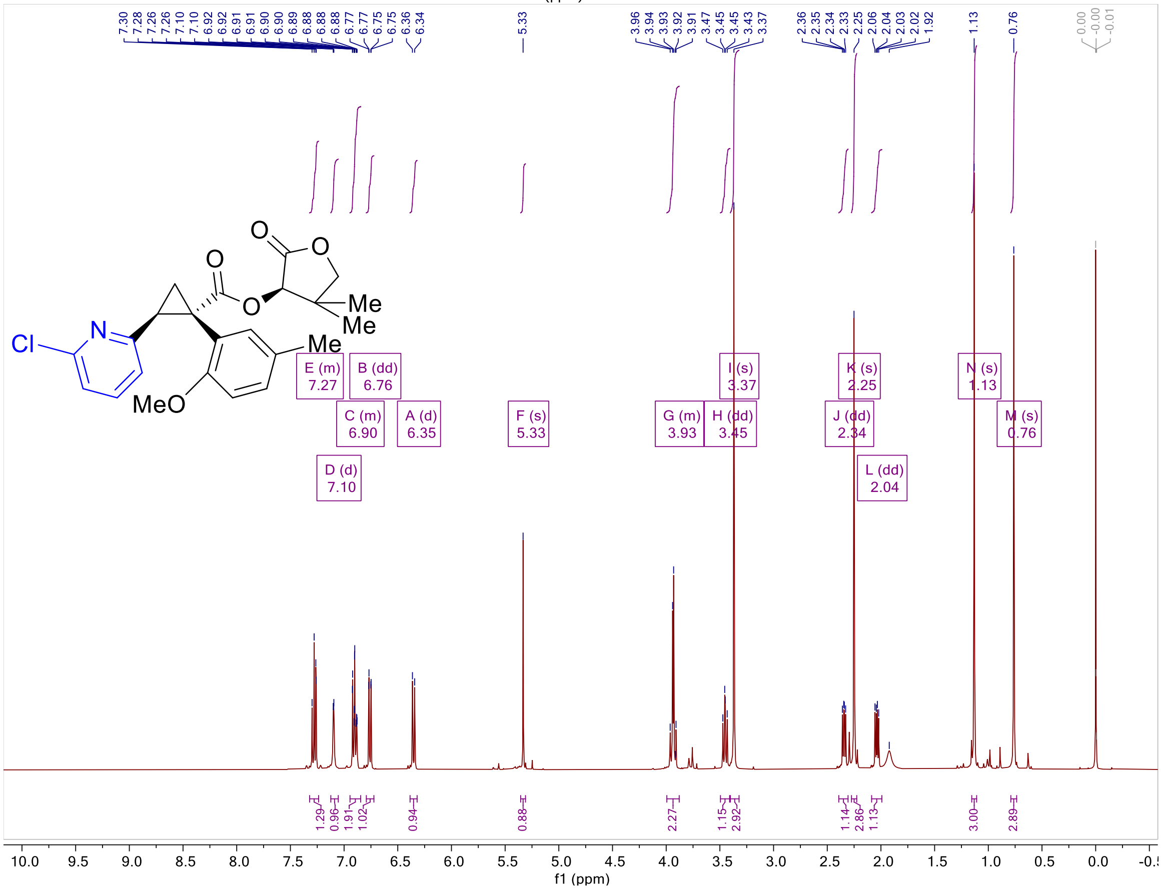

7.30
7.28
7.26
7.26
7.10
7.10
6.92
6.92
6.91
6.91
6.90
6.90
6.89
6.88
6.88
6.88
6.77
6.77
6.75
6.75
6.36
6.34
5.33
3.96
3.94
3.93
3.92
3.91
3.47
3.45
3.45
3.43
3.37
2.36
2.35
2.34
2.33
2.25
2.06
2.04
2.03
2.02
1.92
1.13
0.76
0.00
-0.00
-0.01
E (m) 7.27
B (dd) 6.76
C (m) 6.90
A (d) 6.35
D (d) 7.10
F (s) 5.33
G (m) 3.93
H (dd) 3.45
I (s) 3.37
K (s) 2.25
J (dd) 2.34
L (dd) 2.04
N (s) 1.13
M (s) 0.76
1.29
0.96
1.91
1.02
0.94
0.88
2.27
1.15
2.92
1.14
2.86
1.13
3.00
2.89
Cl
N
MeO
Me
Me
Me
O
O
O
O
f1 (ppm)

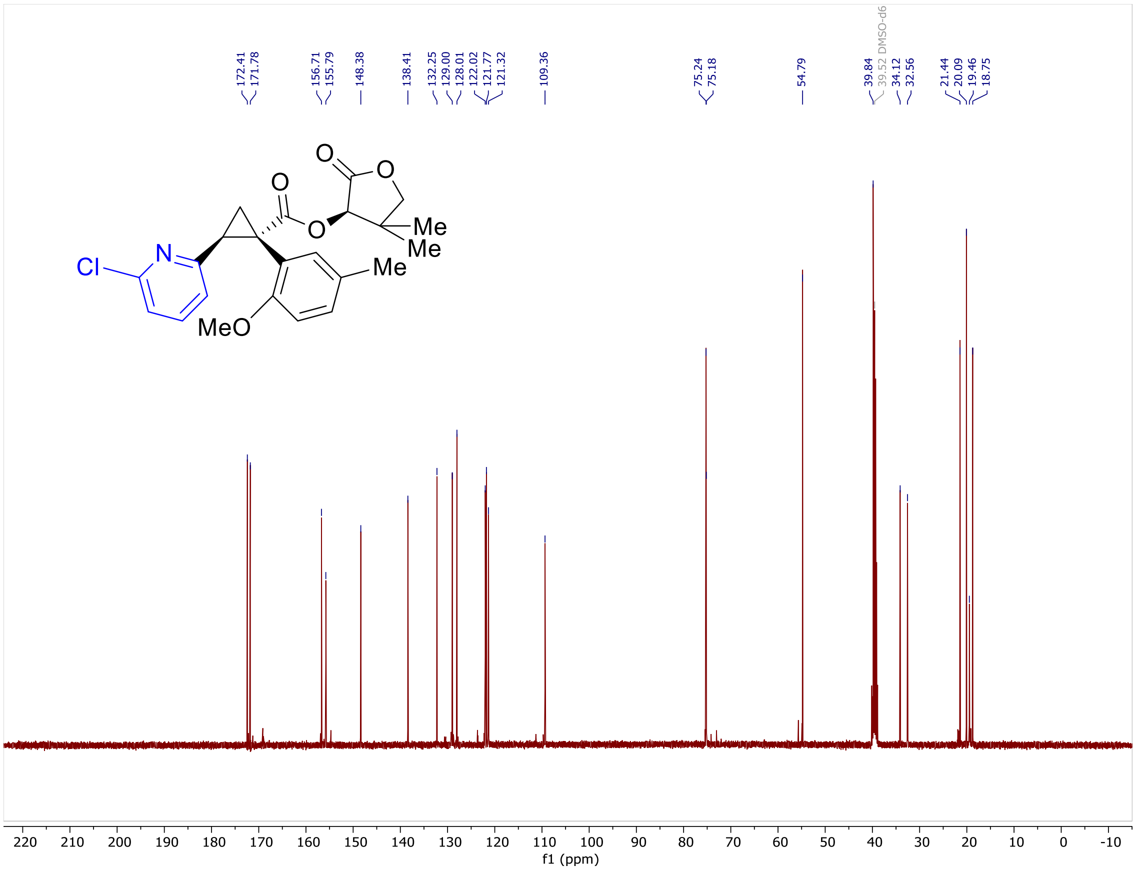
172.41
171.78
156.71
155.79
148.38
138.41
132.25
129.00
128.01
122.02
121.77
121.32
109.36
75.24
75.18
54.79
39.84
39.52 DMSO-d6
34.12
32.56
21.44
20.09
19.46
18.75
Cl
N
O
O
O
O
Me
Me
Me
MeO
f1 (ppm)

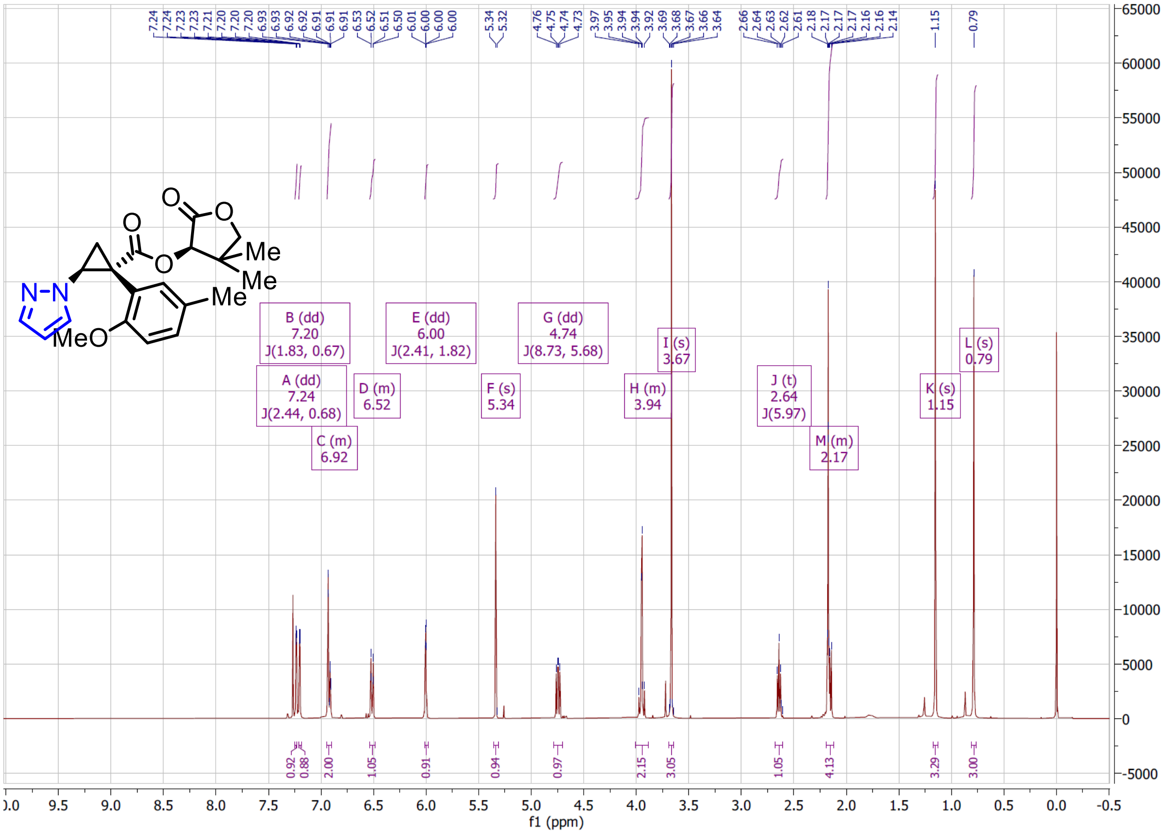
B (dd)
7.20
J(1.83, 0.67)
A (dd)
7.24
J(2.44, 0.68)
C (m)
6.92
D (m)
6.52
E (dd)
6.00
J(2.41, 1.82)
F (s)
5.34
G (dd)
4.74
J(8.73, 5.68)
H (m)
3.94
I (s)
3.67
J (t)
2.64
J(5.97)
M (m)
2.17
K (s)
1.15
L (s)
0.79
0.92
0.88
2.00
1.05
0.91
0.94
0.97
2.15
3.05
1.05
4.13
3.29
3.00
N–N
MeO
Me
Me
Me
O
O
O
O
f1 (ppm)

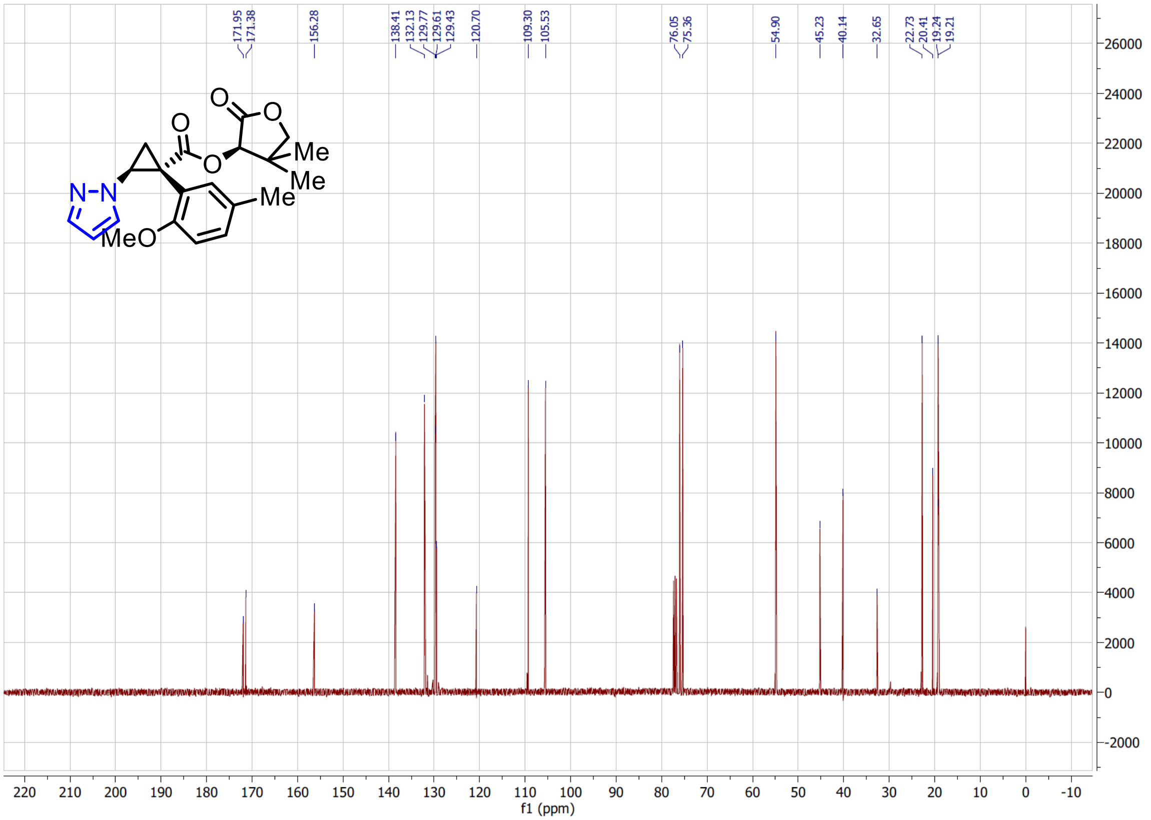
171.95
171.38
156.28
138.41
132.13
129.77
129.61
129.43
120.70
109.30
105.53
76.05
75.36
54.90
45.23
40.14
32.65
22.73
20.41
19.24
19.21
MeO
Me
f1 (ppm)

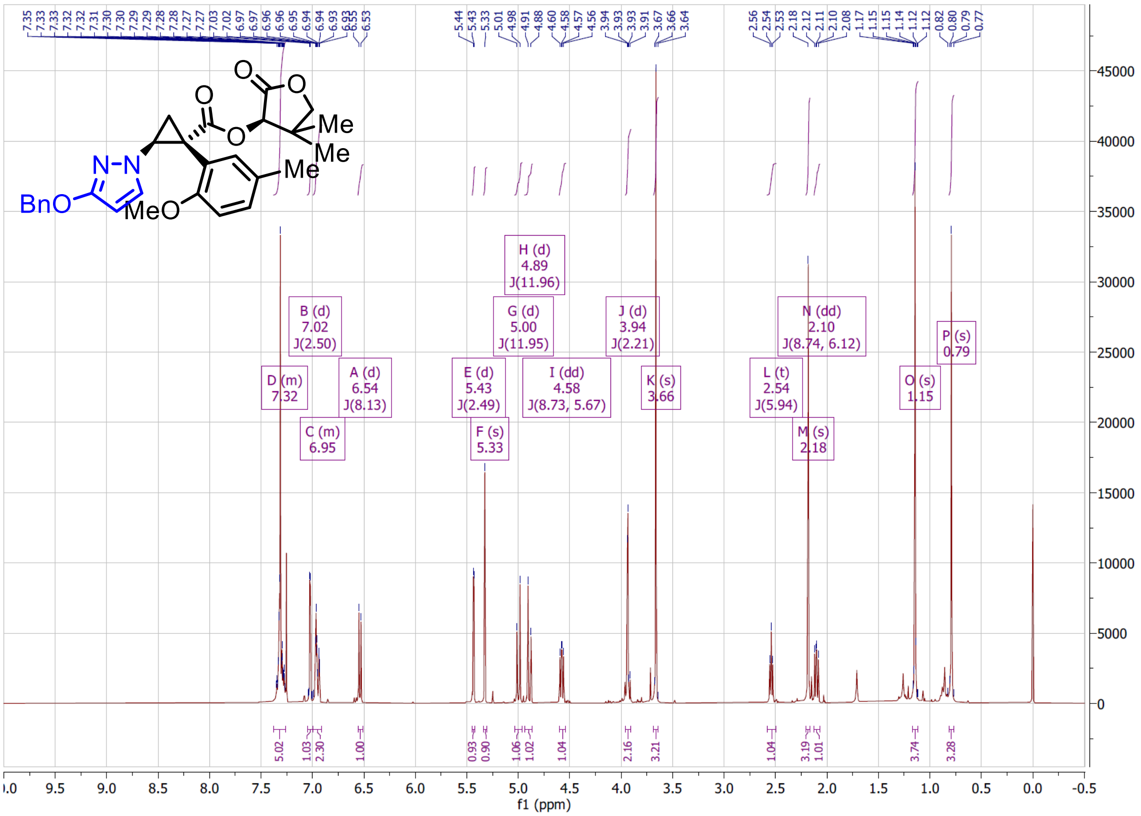
BnO
MeO
Me
D (m) 7.32
B (d) 7.02 J(2.50)
C (m) 6.95
A (d) 6.54 J(8.13)
E (d) 5.43 J(2.49)
F (s) 5.33
G (d) 5.00 J(11.95)
H (d) 4.89 J(11.96)
I (dd) 4.58 J(8.73, 5.67)
J (d) 3.94 J(2.21)
K (s) 3.66
L (t) 2.54 J(5.94)
M (s) 2.18
N (dd) 2.10 J(8.74, 6.12)
O (s) 1.15
P (s) 0.79
5.02
1.03
2.30
1.00
0.93
0.90
1.06
1.02
1.04
2.16
3.21
1.04
3.19
1.01
3.74
3.28
f1 (ppm)

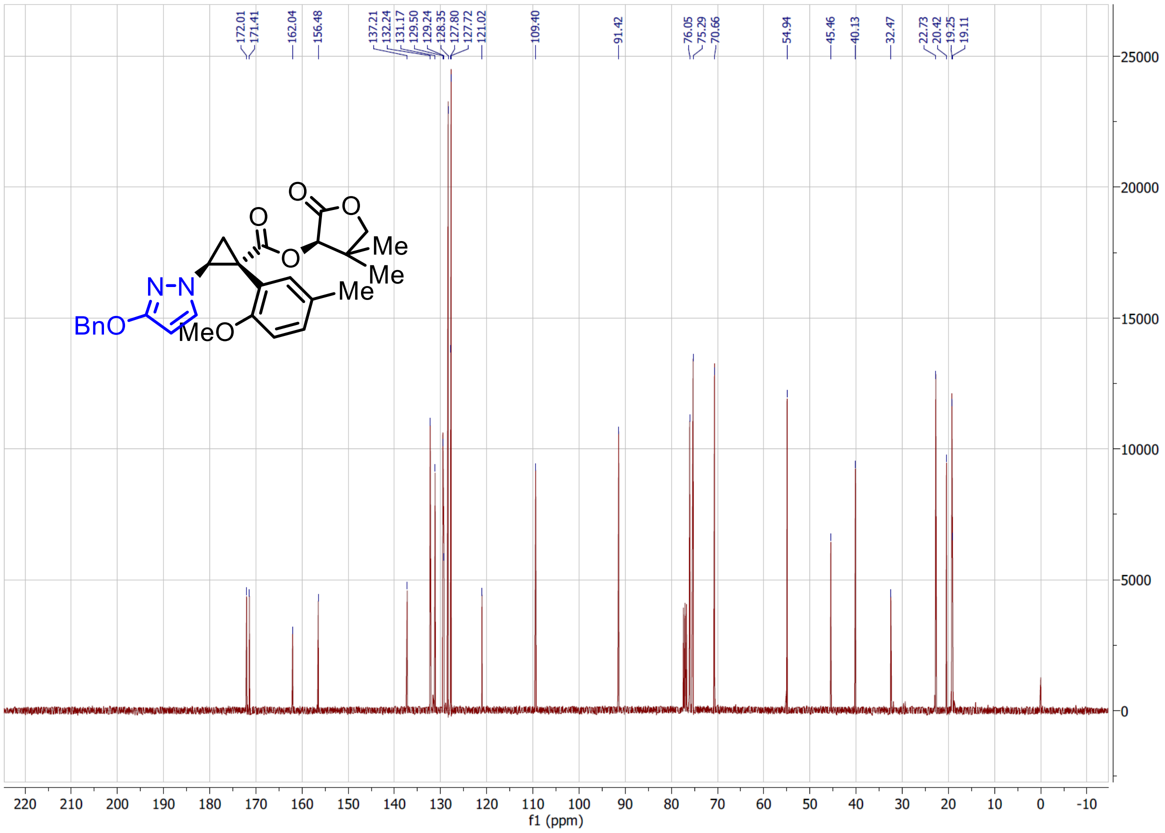

172.01
171.41
162.04
156.48
137.21
132.24
131.17
129.50
129.24
128.35
127.80
127.72
121.02
109.40
91.42
76.05
75.29
70.66
54.94
45.46
40.13
32.47
22.73
20.42
19.25
19.11
BnO
MeO
Me
f1 (ppm)

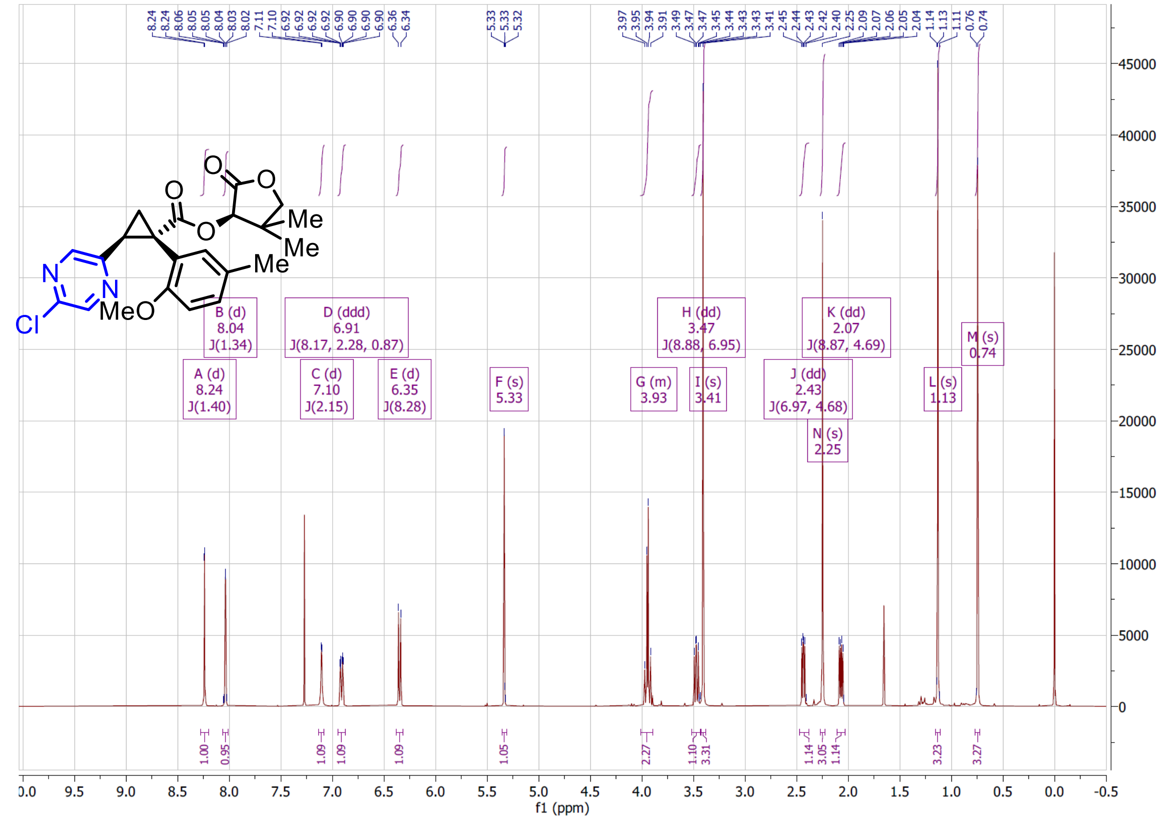

A (d) 8.24 J(1.40)
B (d) 8.04 J(1.34)
C (d) 7.10 J(2.15)
D (ddd) 6.91 J(8.17, 2.28, 0.87)
E (d) 6.35 J(8.28)
F (s) 5.33
G (m) 3.93
H (dd) 3.47 J(8.88, 6.95)
I (s) 3.41
J (dd) 2.43 J(6.97, 4.68)
K (dd) 2.07 J(8.87, 4.69)
N (s) 2.25
L (s) 1.13
M (s) 0.74
Cl
MeO
Me
f1 (ppm)

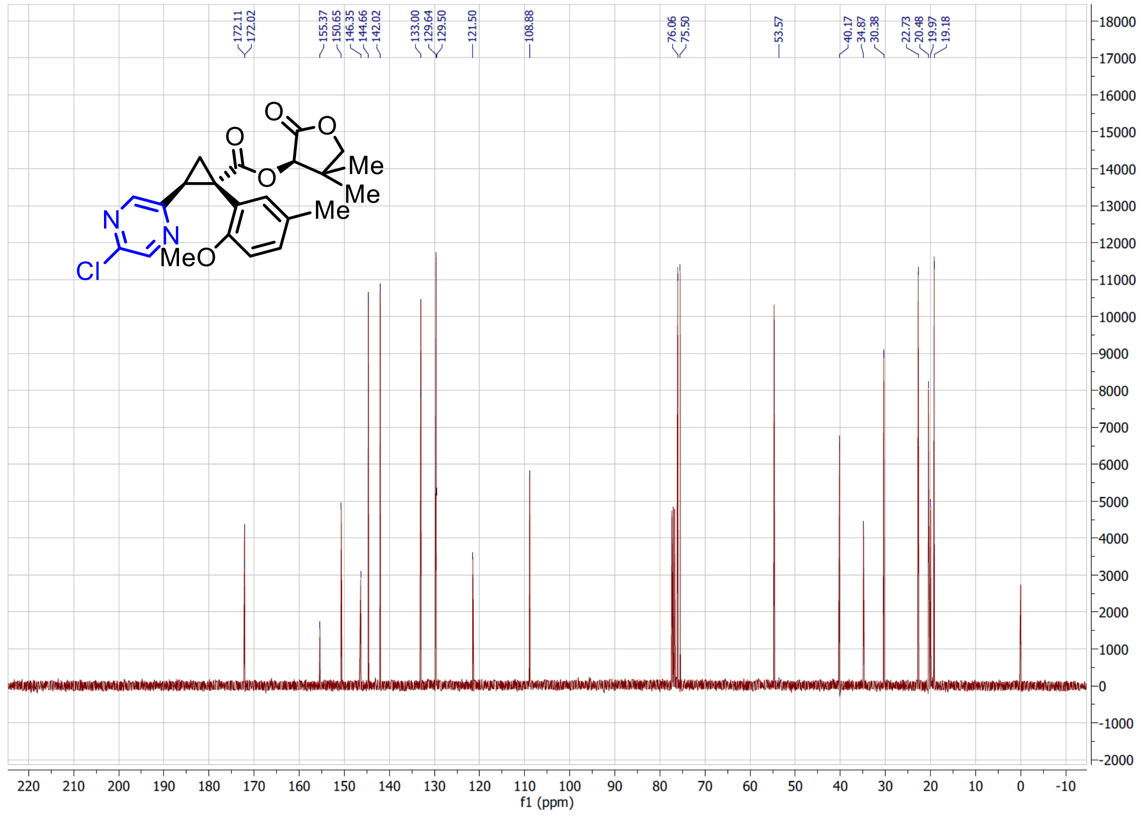

172.11
172.02
155.37
150.65
146.35
144.66
142.02
133.00
129.64
129.50
121.50
108.88
76.06
75.50
53.57
40.17
34.87
30.38
22.73
20.48
19.97
19.18
O
O
O
O
O
Me
Me
Me
N
N
Cl
MeO
f1 (ppm)

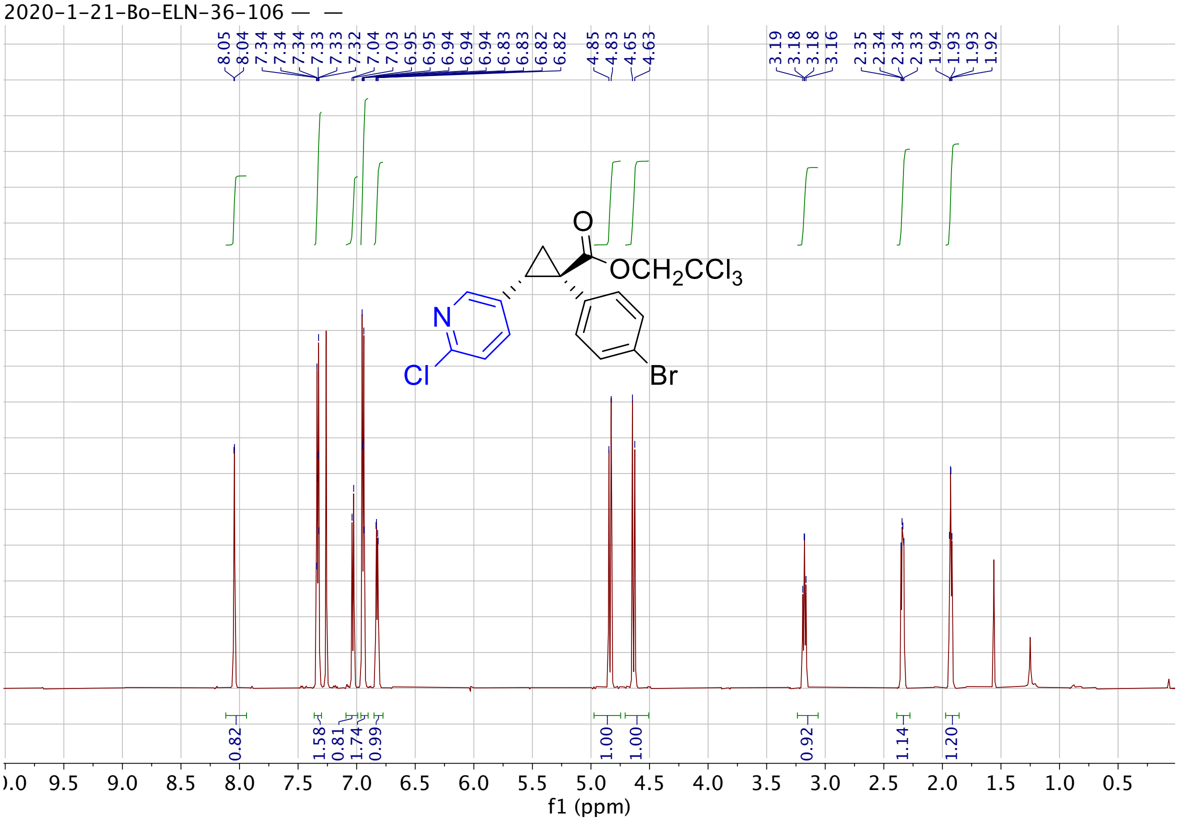

2020-1-21-Bo-ELN-36-106 — —
8.05
8.04
7.34
7.34
7.33
7.33
7.32
7.04
7.03
6.95
6.95
6.94
6.94
6.83
6.83
6.82
6.82
4.85
4.83
4.65
4.63
3.19
3.18
3.18
3.16
2.35
2.34
2.34
2.33
1.94
1.93
1.93
1.92
O
OCH_2CCl_3
N
Cl
Br
0.82
1.58
0.81
1.74
0.99
1.00
1.00
0.92
1.14
1.20
f1 (ppm)

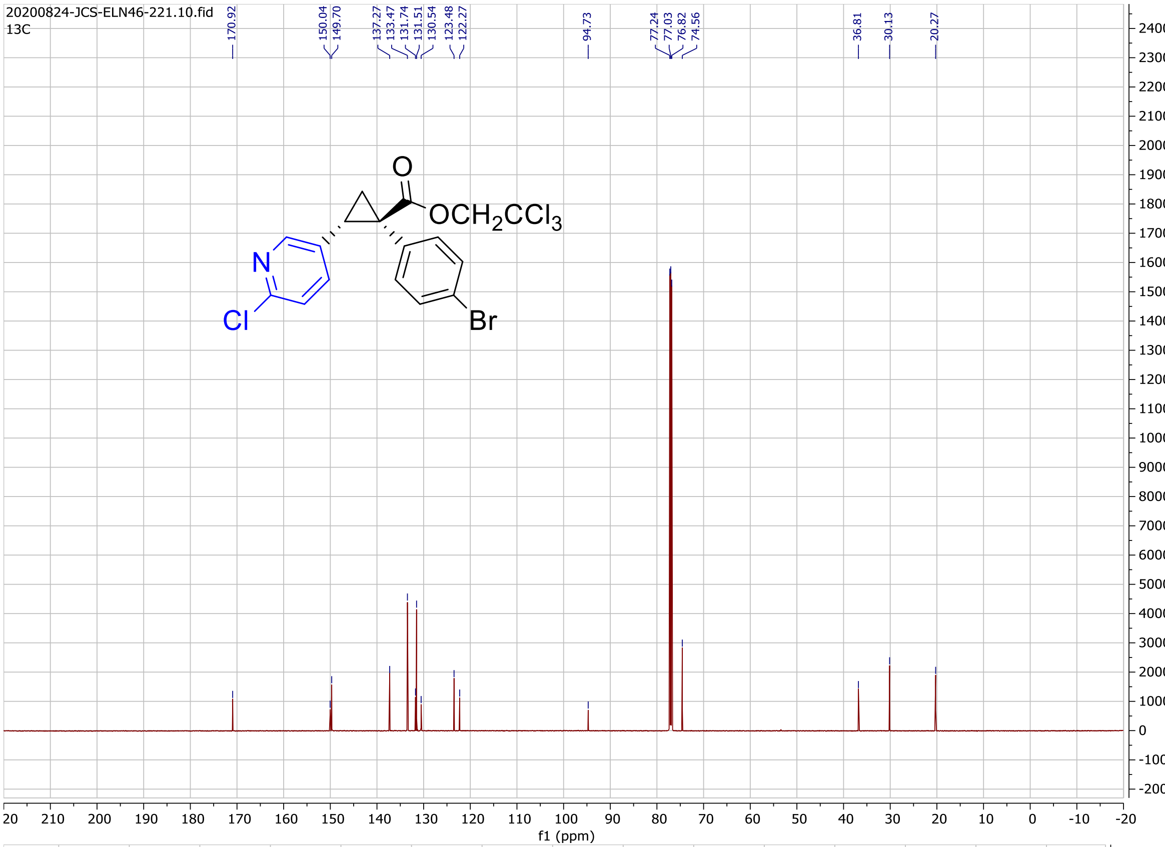
20200824-JCS-ELN46-221.10.fid
13C
170.92
150.04
149.70
137.27
133.47
131.74
131.51
130.54
123.48
122.27
94.73
77.24
77.03
76.82
74.56
36.81
30.13
20.27
OCH2CCl3
N
Cl
Br
O
f1 (ppm)

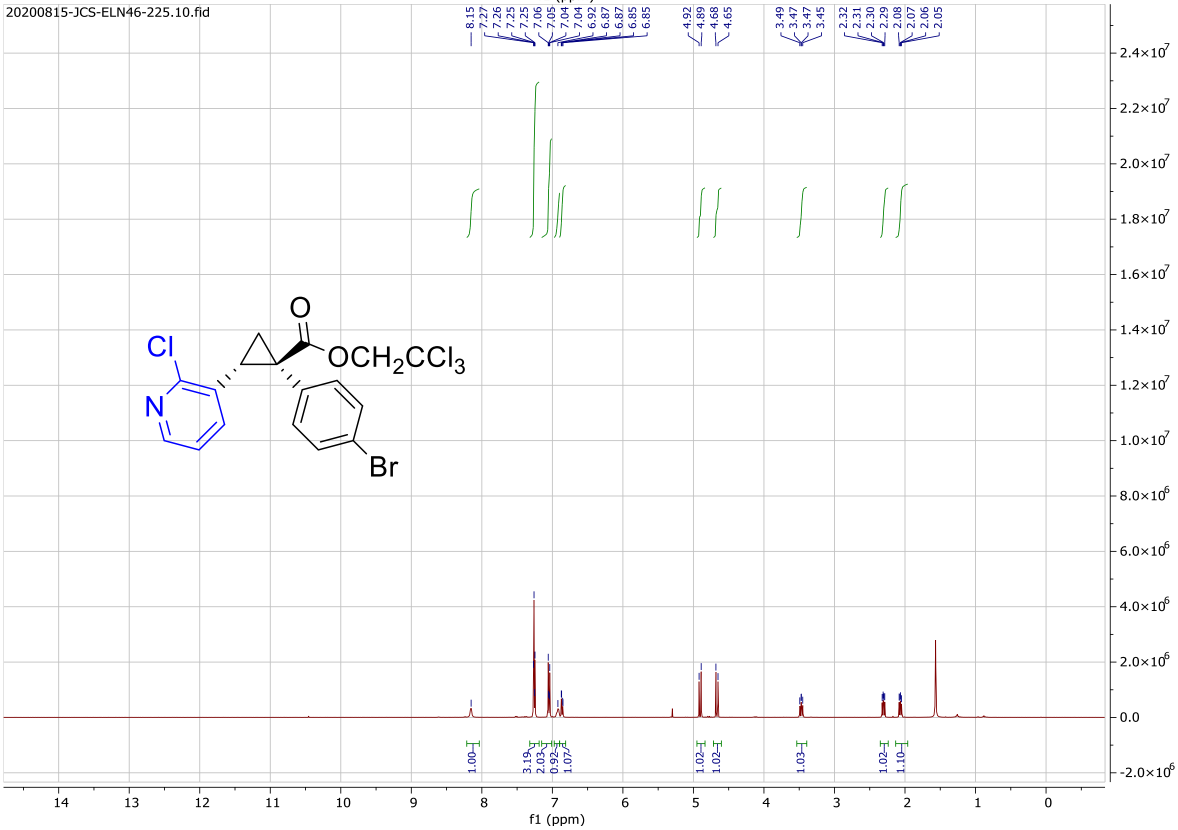
20200815-JCS-ELN46-225.10.fid
8.15
7.27
7.26
7.25
7.25
7.06
7.05
7.04
6.92
6.87
6.87
6.85
6.85
4.92
4.89
4.68
4.65
3.49
3.47
3.47
3.45
2.32
2.31
2.30
2.29
2.08
2.07
2.06
2.05
Cl
N
O
OCH2CCl3
Br
1.00
3.19
2.03
0.92
1.07
1.02
1.02
1.03
1.02
1.10
f1 (ppm)

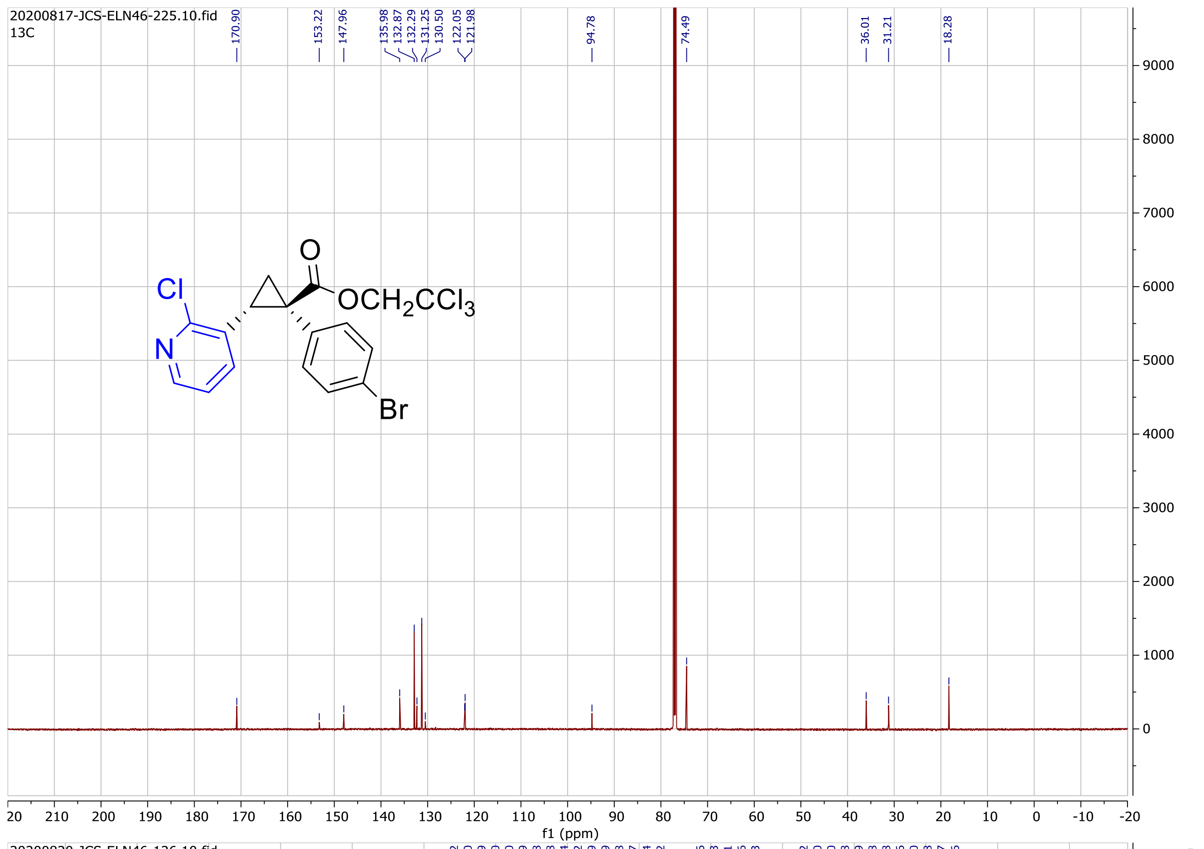
20200817-JCS-ELN46-225.10.fid
13C
170.90
153.22
147.96
135.98
132.87
132.29
131.25
130.50
122.05
121.98
94.78
74.49
36.01
31.21
18.28
O
Cl
N
OCH2CCl3
Br
f1 (ppm)

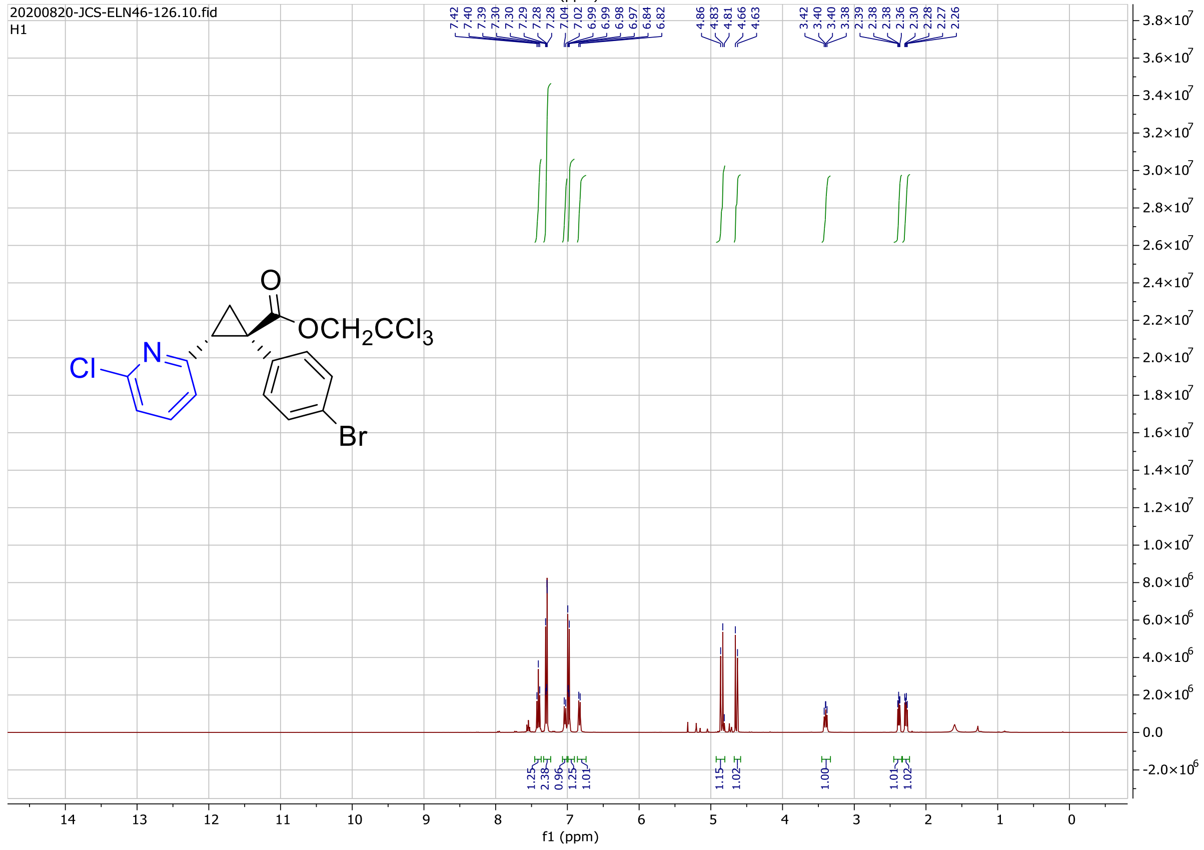
20200820-JCS-ELN46-126.10.fid
H1
7.42
7.40
7.39
7.30
7.30
7.29
7.28
7.28
7.04
7.02
6.99
6.99
6.98
6.97
6.84
6.82
4.86
4.83
4.81
4.66
4.63
3.42
3.40
3.40
3.38
2.39
2.38
2.38
2.36
2.30
2.28
2.27
2.26
O
Cl
N
OCH2CCl3
Br
1.25
2.38
0.96
1.25
1.01
1.15
1.02
1.00
1.01
1.02
f1 (ppm)

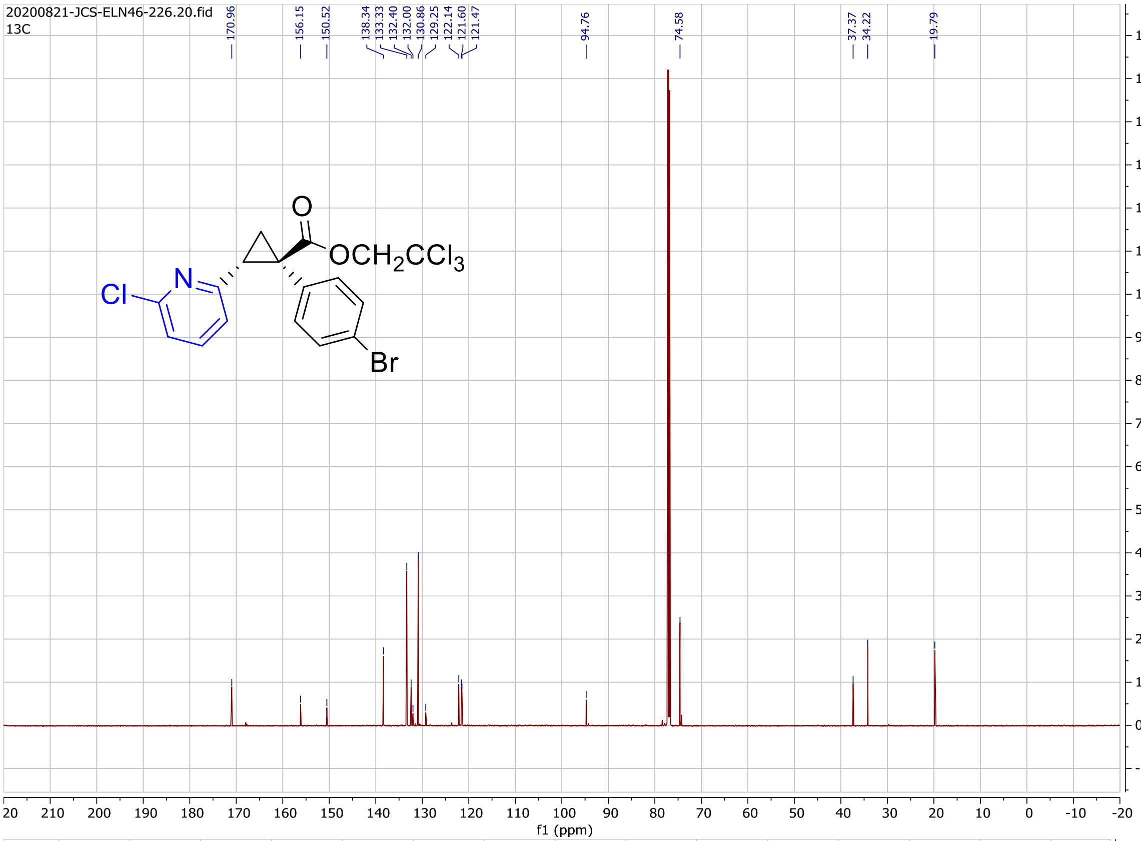

20200821-JCS-ELN46-226.20.fid
13C
170.96
156.15
150.52
138.34
133.33
132.40
132.00
130.86
129.25
122.14
121.60
121.47
94.76
74.58
37.37
34.22
19.79
Cl
N
O
OCH2CCl3
Br
f1 (ppm)

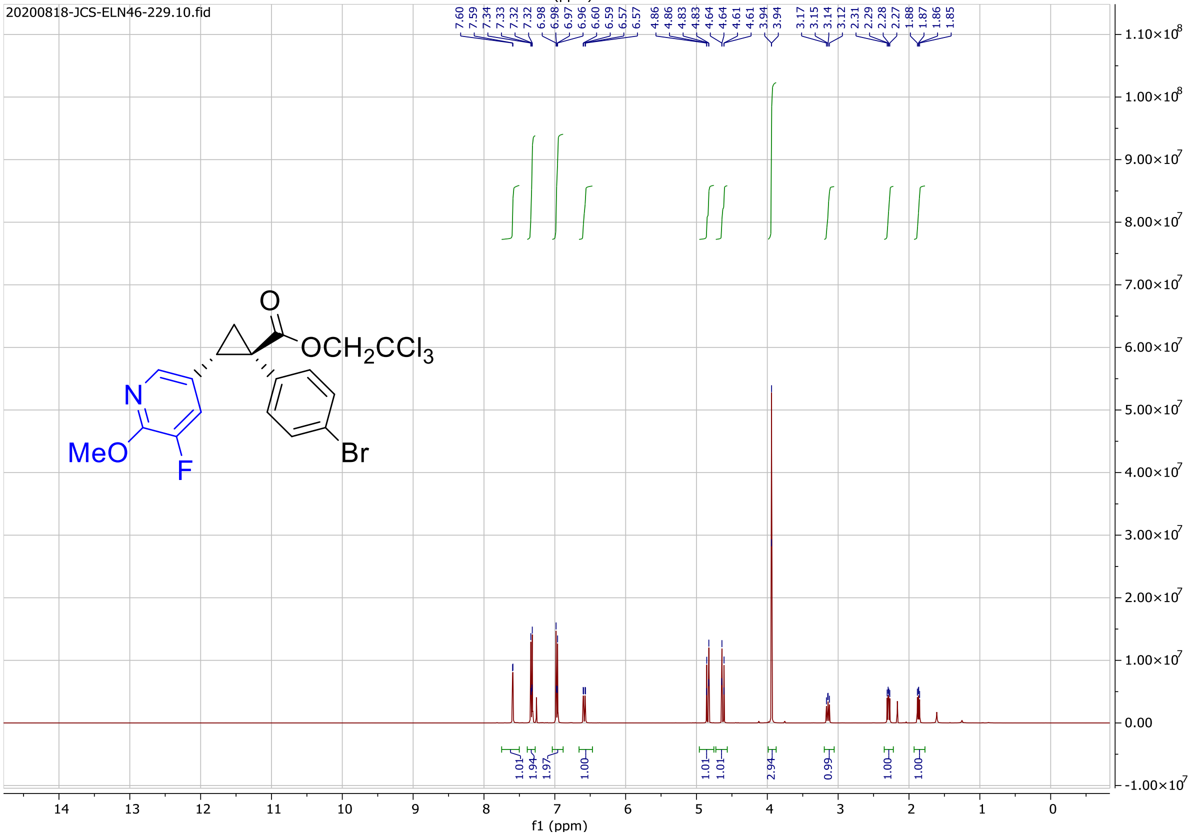

20200818-JCS-ELN46-229.10.fid
7.60
7.59
7.34
7.33
7.32
7.32
6.98
6.98
6.97
6.96
6.60
6.59
6.57
6.57
4.86
4.86
4.83
4.83
4.64
4.64
4.61
4.61
3.94
3.94
3.17
3.15
3.14
3.12
2.31
2.29
2.28
2.27
1.88
1.87
1.86
1.85
N
MeO
F
O
OCH2CCl3
Br
1.01
1.94
1.97
1.00
1.01
1.01
2.94
0.99
1.00
1.00
f1 (ppm)

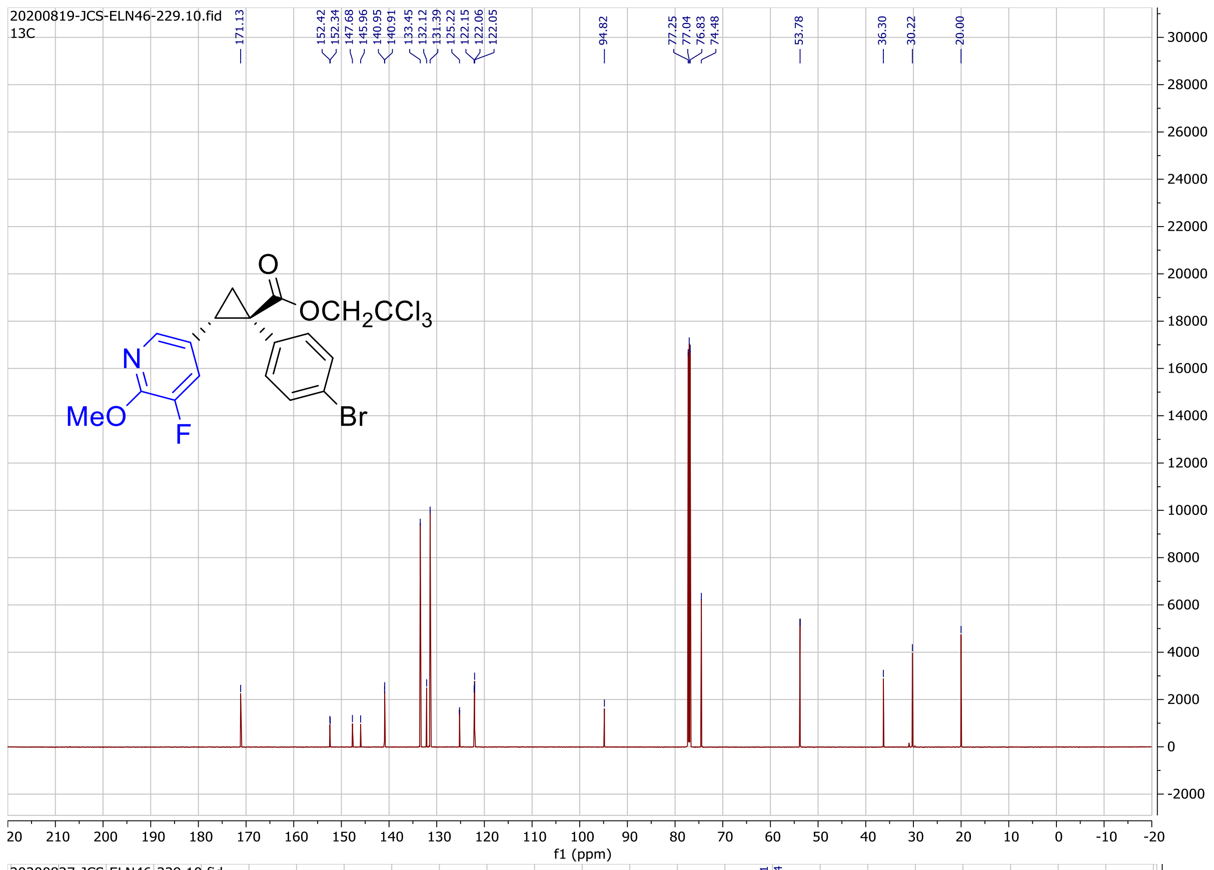

20200819-JCS-ELN46-229.10.fid
13C
171.13
152.42
152.34
147.68
145.96
140.95
140.91
133.45
132.12
131.39
125.22
122.15
122.06
122.05
94.82
77.25
77.04
76.83
74.48
53.78
36.30
30.22
20.00
O
OCH2CCl3
N
MeO
F
Br
f1 (ppm)

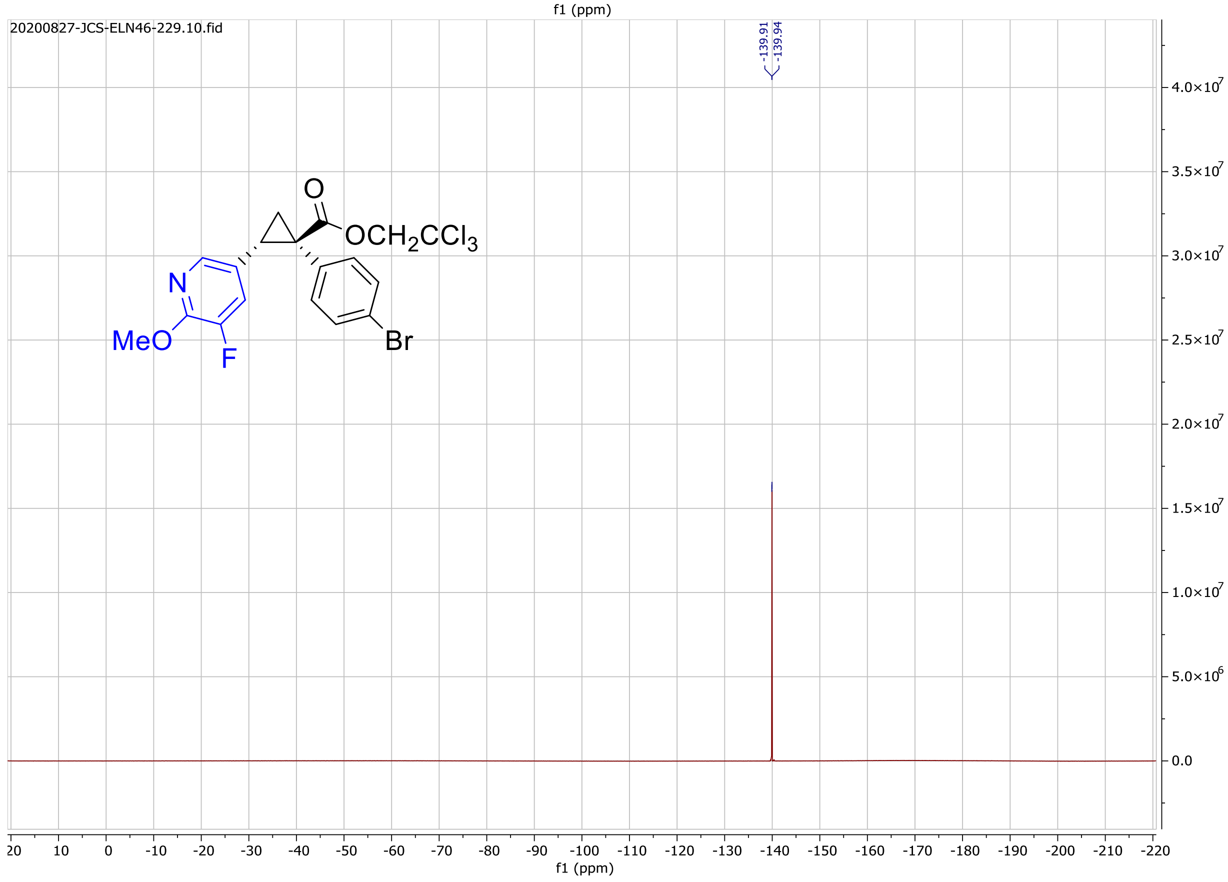

20200827-JCS-ELN46-229.10.fid
-139.91
-139.94
O
OCH2CCl3
N
MeO
F
Br
f1 (ppm)

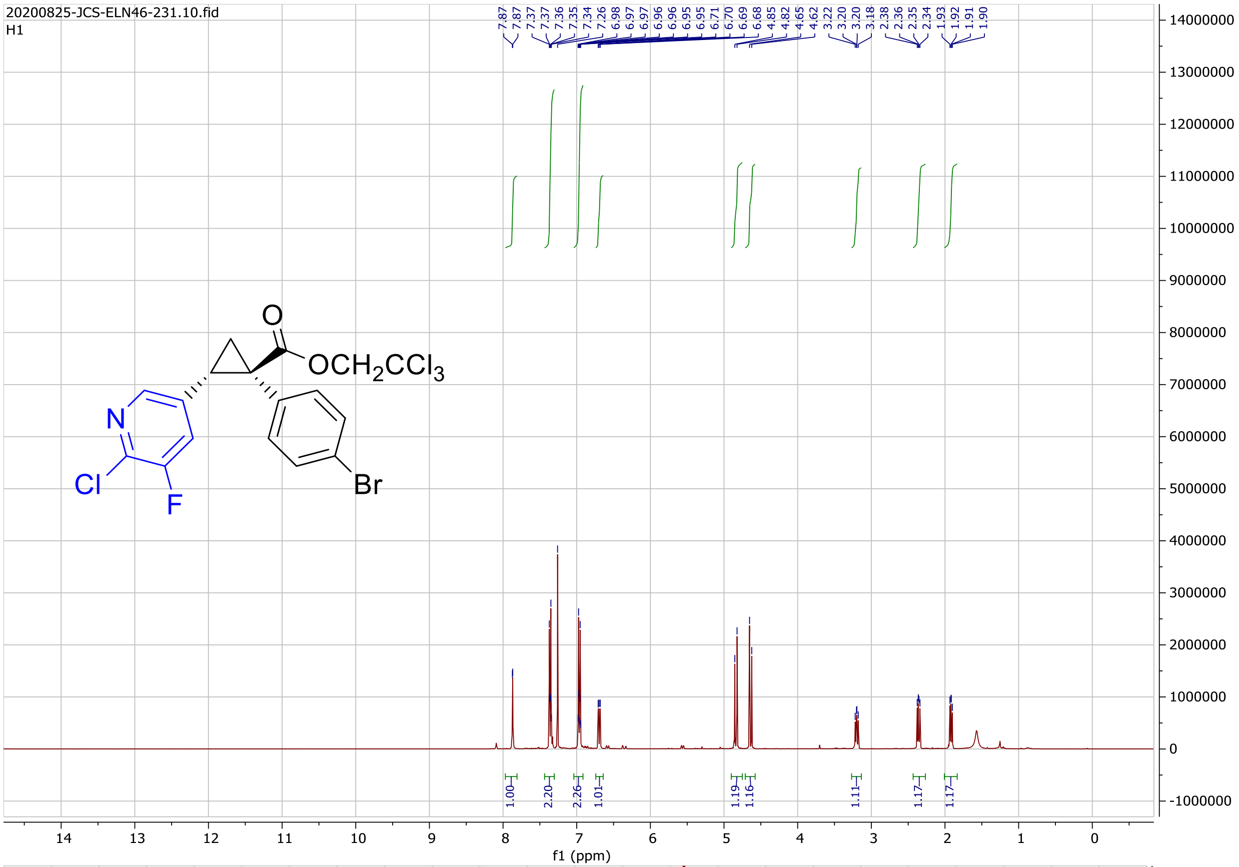

20200825-JCS-ELN46-231.10.fid
H1
7.87
7.87
7.37
7.36
7.35
7.34
7.26
6.98
6.97
6.97
6.96
6.96
6.95
6.95
6.71
6.70
6.69
6.68
4.85
4.82
4.65
4.62
3.22
3.20
3.20
3.18
2.38
2.36
2.35
2.34
1.93
1.92
1.91
1.90
O
OCH2CCl3
N
Cl
F
Br
1.00
2.20
2.26
1.01
1.19
1.16
1.11
1.17
1.17
f1 (ppm)

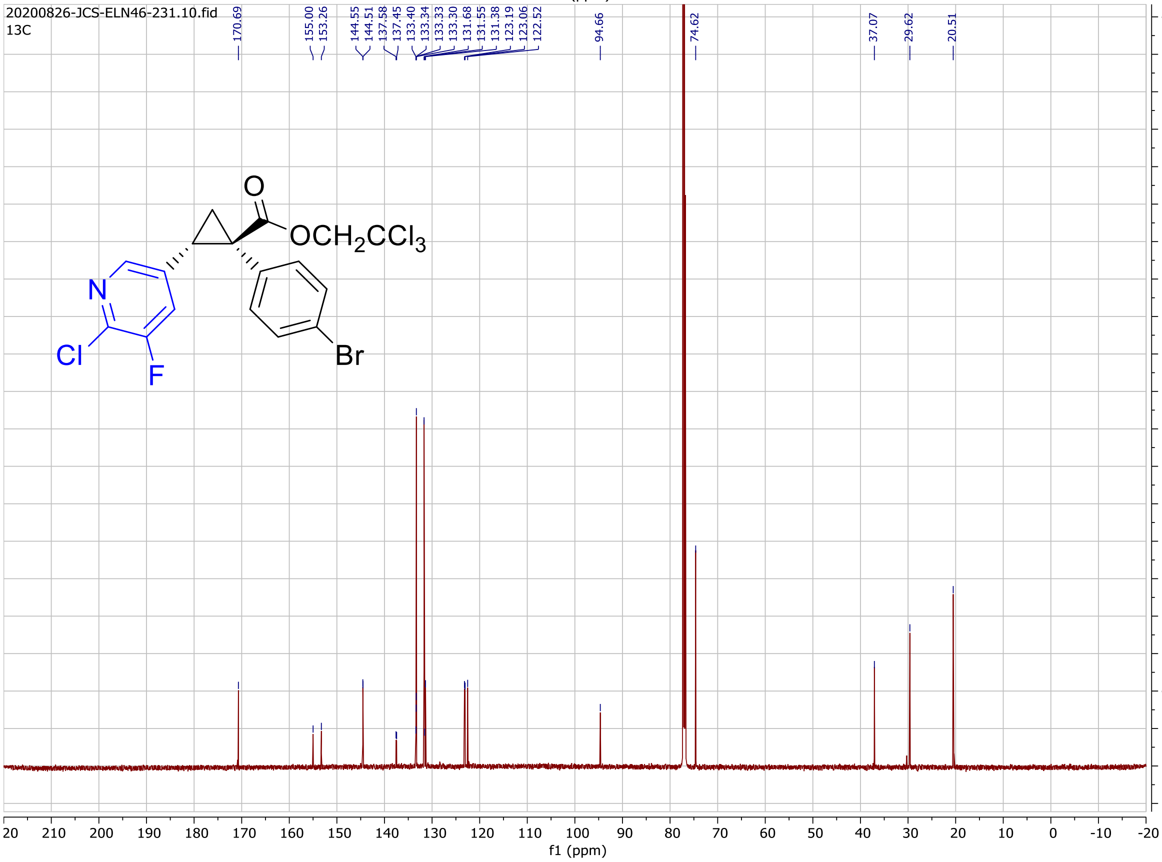

20200826-JCS-ELN46-231.10.fid
13C
170.69
155.00
153.26
144.55
144.51
137.58
137.45
133.40
133.34
133.33
133.30
131.68
131.55
131.38
123.19
123.06
122.52
94.66
74.62
37.07
29.62
20.51
O
OCH2CCl3
N
Cl
F
Br
f1 (ppm)

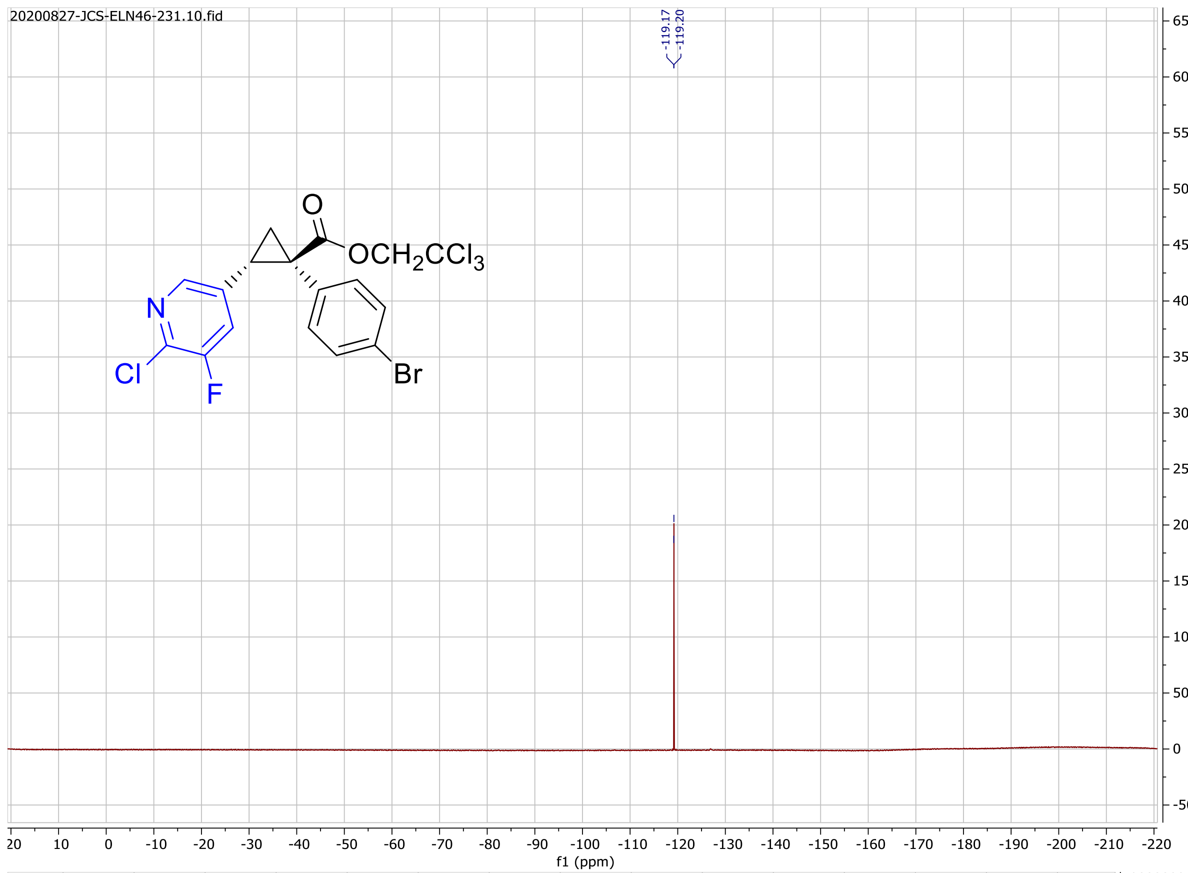
20200827-JCS-ELN46-231.10.fid
-119.17
-119.20
O
OCH2CCl3
N
Cl
F
Br
f1 (ppm)

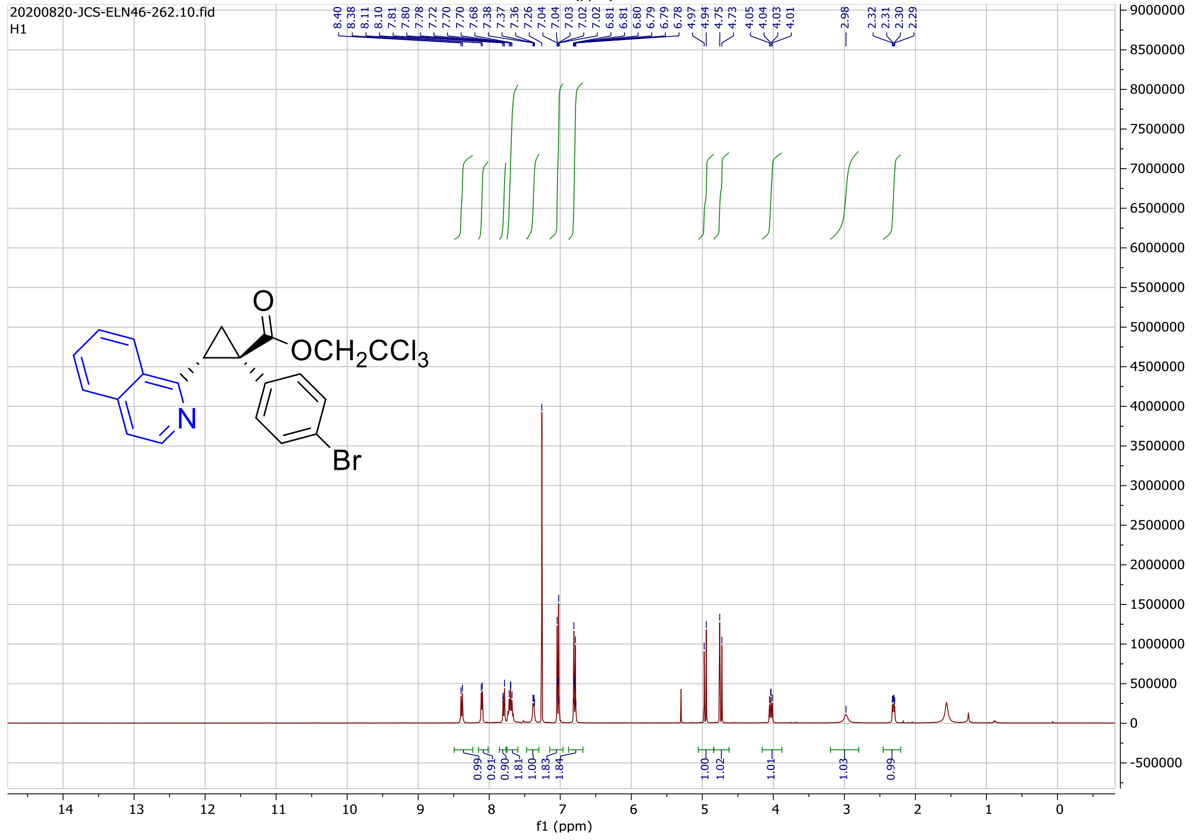
20200820-JCS-ELN46-262.10.fid
H1
8.40
8.38
8.11
8.10
7.81
7.80
7.78
7.72
7.70
7.70
7.68
7.38
7.37
7.36
7.26
7.04
7.04
7.03
7.02
7.02
6.81
6.81
6.80
6.79
6.79
6.78
4.97
4.94
4.75
4.73
4.05
4.04
4.03
4.01
2.98
2.32
2.31
2.30
2.29
O
OCH2CCl3
N
Br
0.99
0.91
0.90
1.81
1.00
1.83
1.84
1.00
1.02
1.01
1.03
0.99
f1 (ppm)

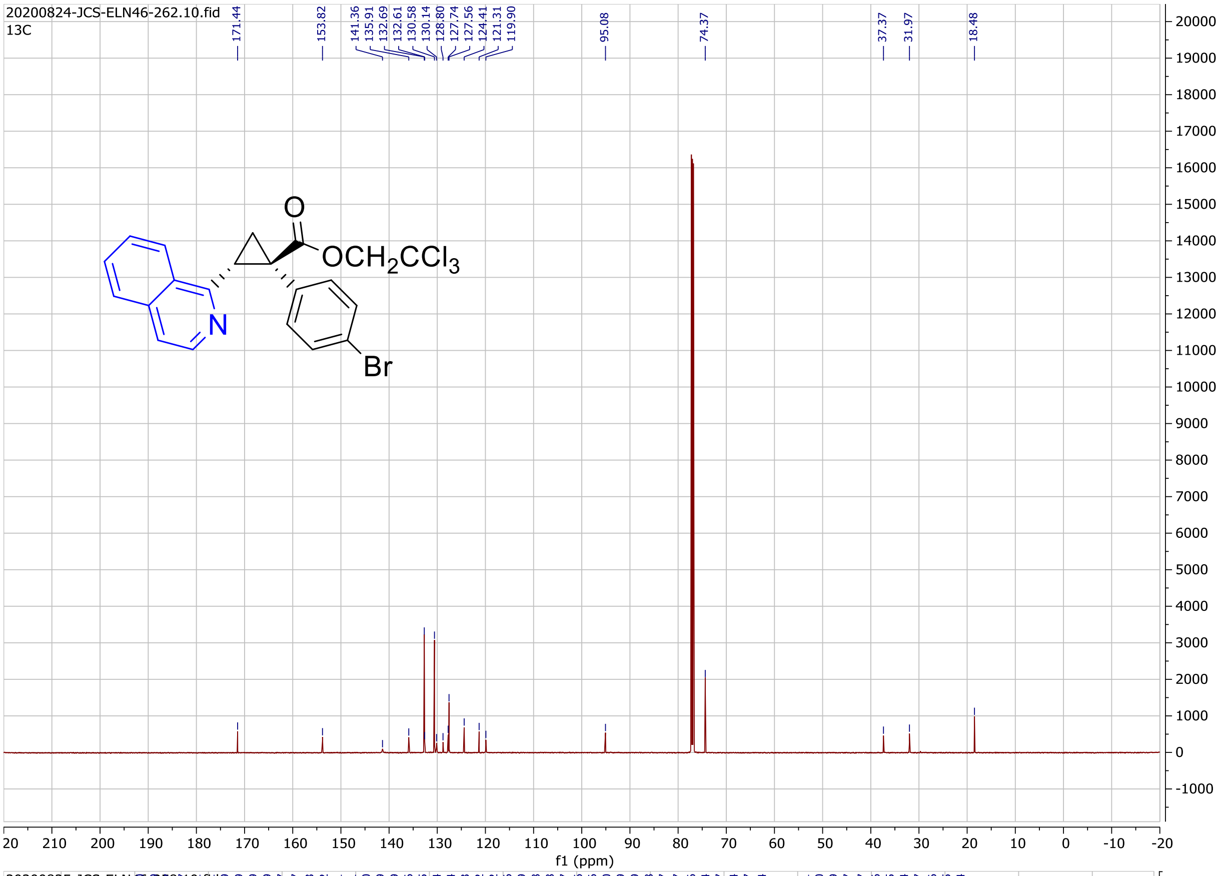
20200824-JCS-ELN46-262.10.fid
13C
171.44
153.82
141.36
135.91
132.69
132.61
130.58
130.14
128.80
127.74
127.56
124.41
121.31
119.90
95.08
74.37
37.37
31.97
18.48
OCH2CCl3
O
N
Br
f1 (ppm)

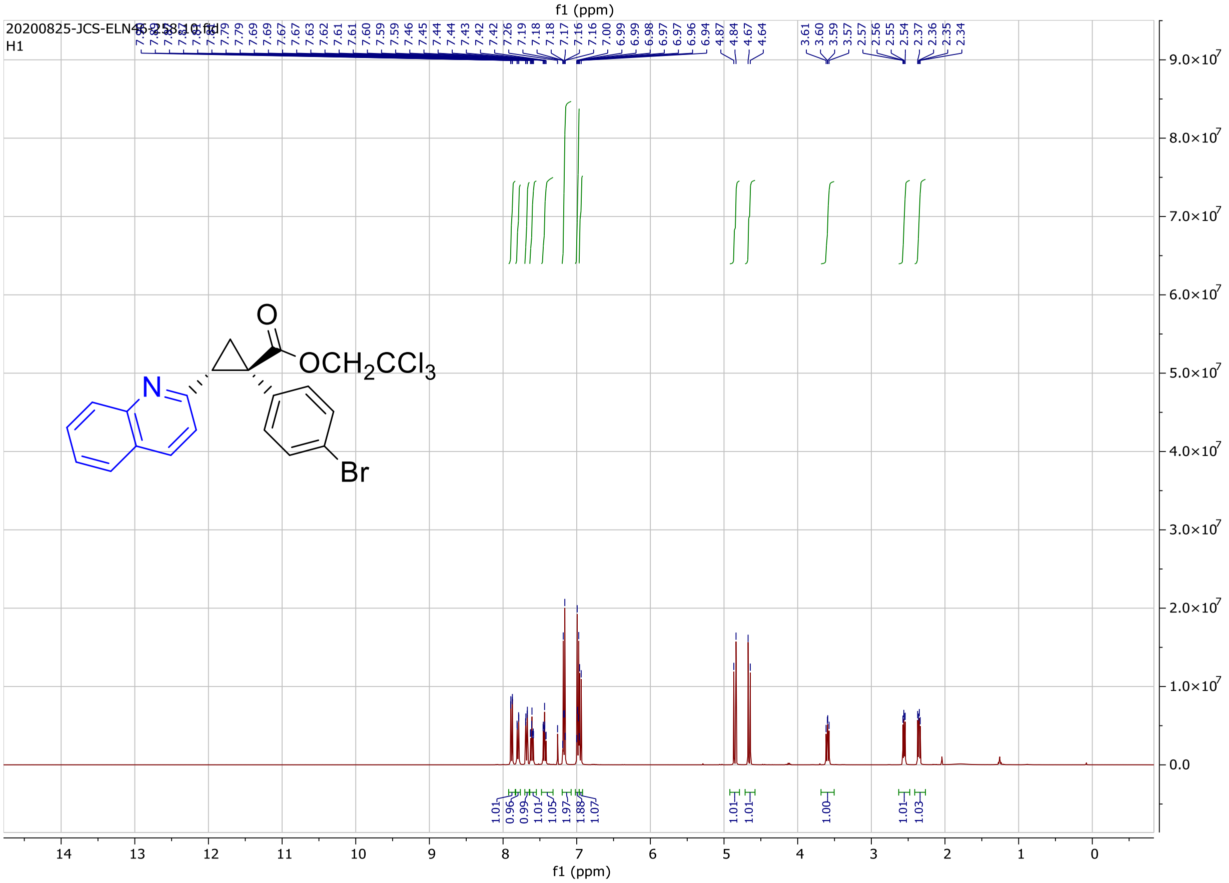
H1
7.79
7.79
7.69
7.69
7.67
7.67
7.63
7.62
7.61
7.61
7.60
7.59
7.59
7.46
7.45
7.44
7.44
7.43
7.42
7.42
7.26
7.19
7.18
7.17
7.16
7.16
7.00
6.99
6.99
6.98
6.97
6.97
6.96
6.94
4.87
4.84
4.67
4.64
3.61
3.60
3.59
3.57
2.57
2.56
2.55
2.54
2.37
2.36
2.35
2.34
1.01
0.96
0.99
1.01
1.05
1.97
1.88
1.07
1.01
1.01
1.00
1.01
1.03
OCH2CCl3
O
N
Br
f1 (ppm)

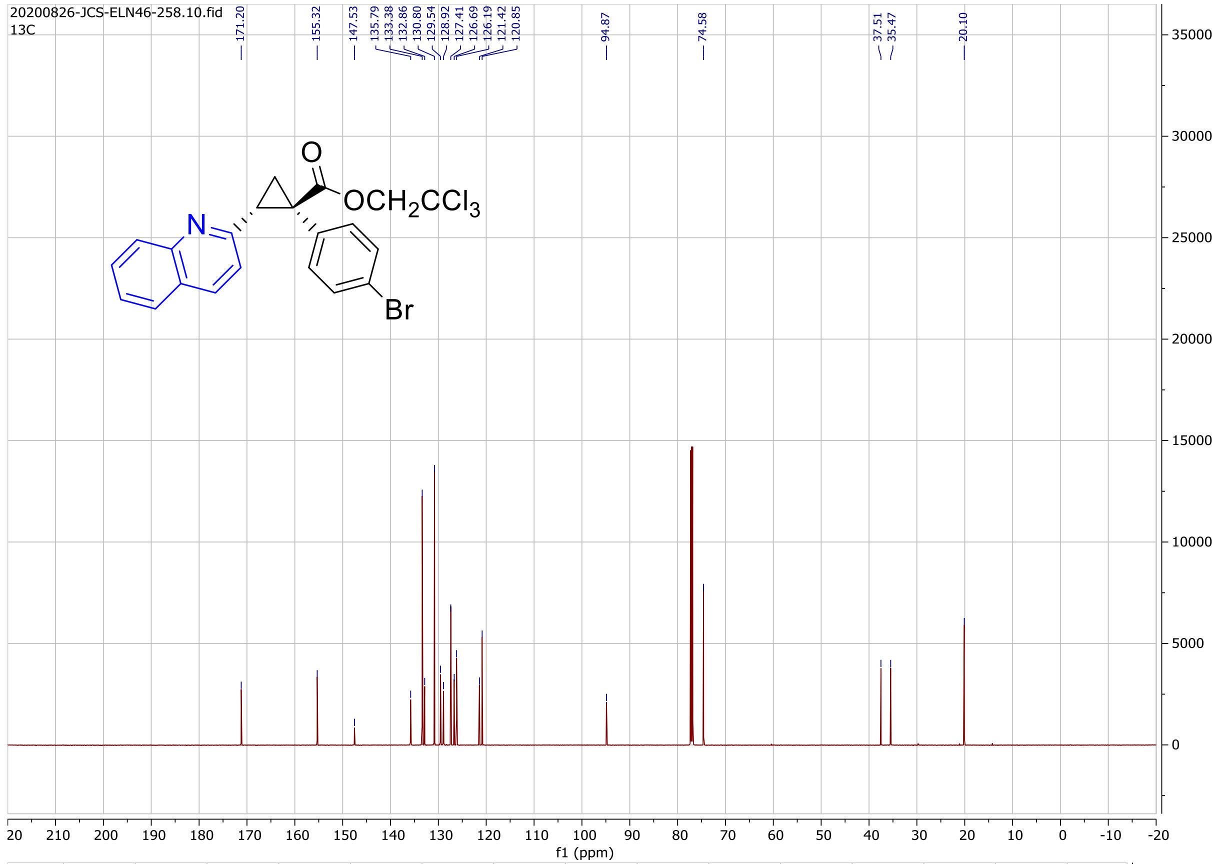
20200826-JCS-ELN46-258.10.fid
13C
171.20
155.32
147.53
135.79
133.38
132.86
130.80
129.54
128.92
127.41
126.69
126.19
121.42
120.85
94.87
74.58
37.51
35.47
20.10
O
OCH2CCl3
N
Br
f1 (ppm)

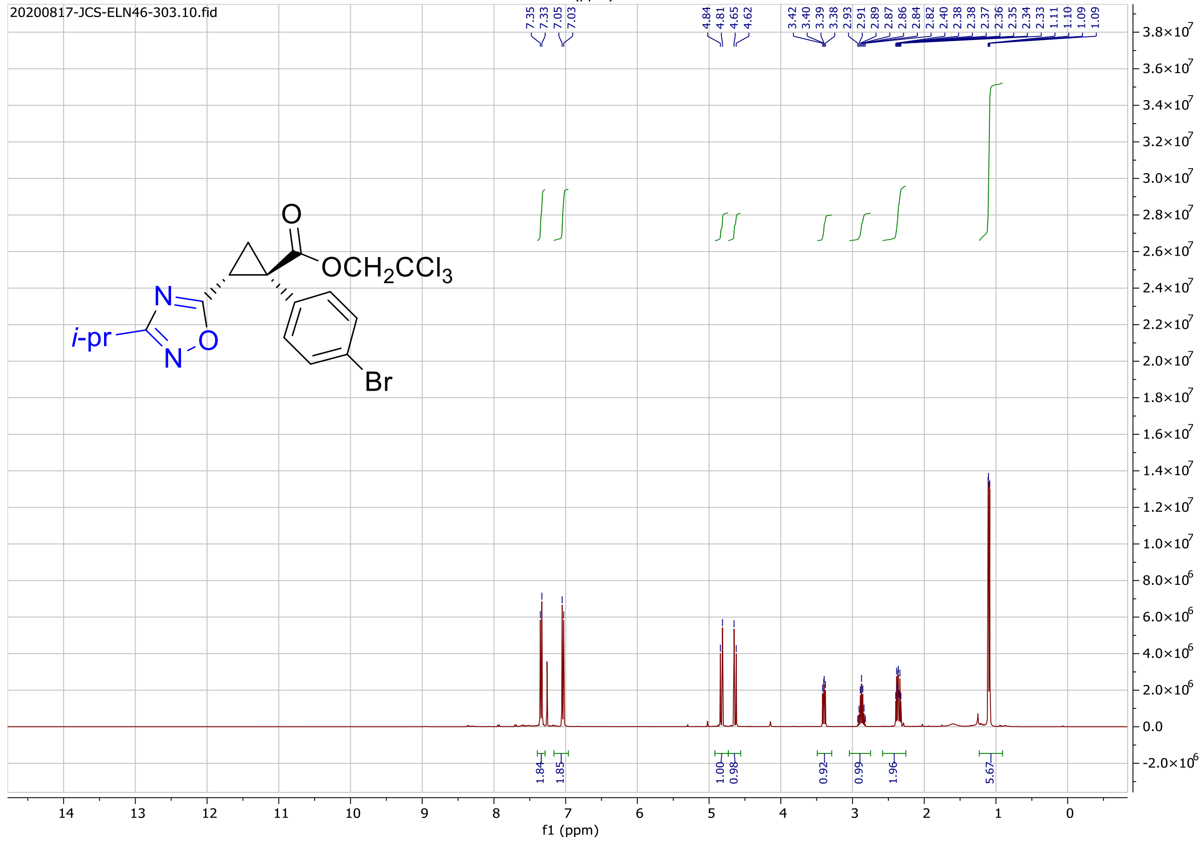
20200817-JCS-ELN46-303.10.fid
7.35
7.33
7.05
7.03
4.84
4.81
4.65
4.62
3.42
3.40
3.39
3.38
2.93
2.91
2.89
2.87
2.86
2.84
2.82
2.40
2.38
2.38
2.37
2.36
2.35
2.34
2.33
1.11
1.10
1.09
1.09
O
OCH2CCl3
N
i-pr
N
O
Br
1.84
1.85
1.00
0.98
0.92
0.99
1.96
5.67
f1 (ppm)

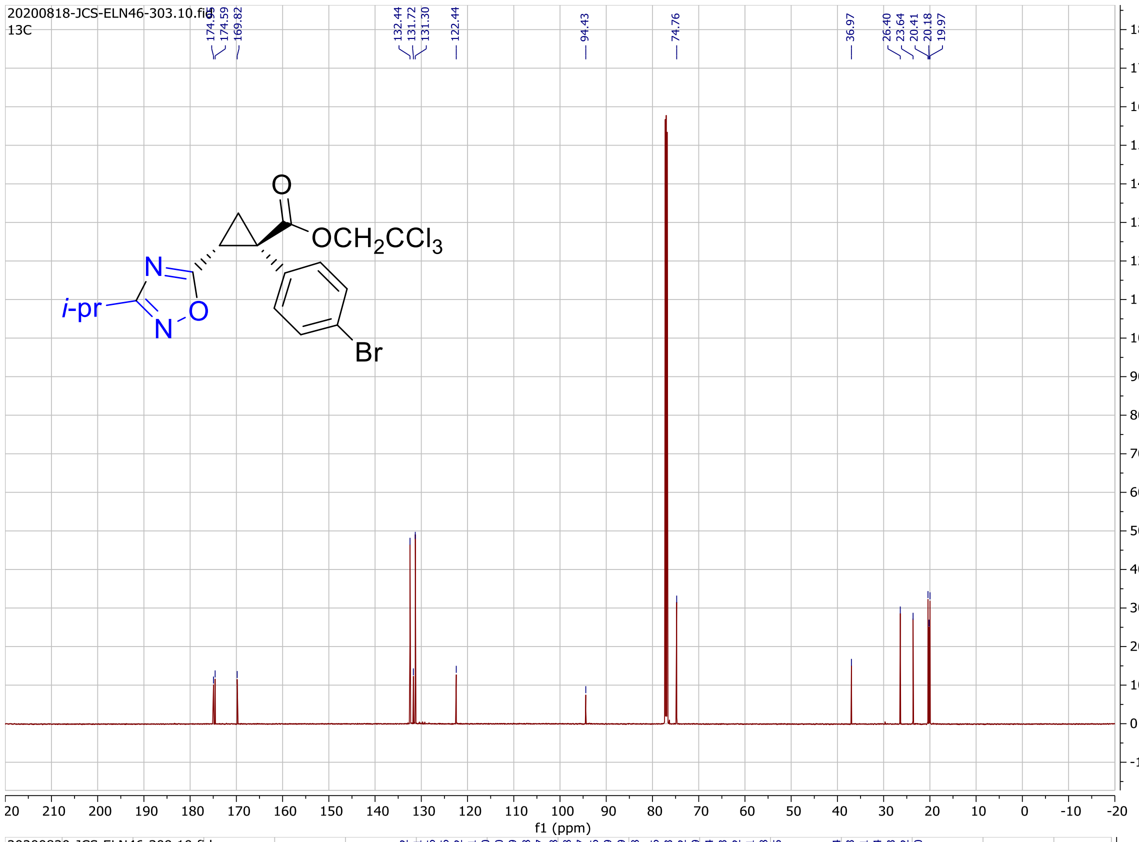
20200818-JCS-ELN46-303.10.fid
13C
174.65
174.59
169.82
132.44
131.72
131.30
122.44
94.43
74.76
36.97
26.40
23.64
20.41
20.18
19.97
O
OCH2CCl3
N
i-pr
N-O
Br
f1 (ppm)

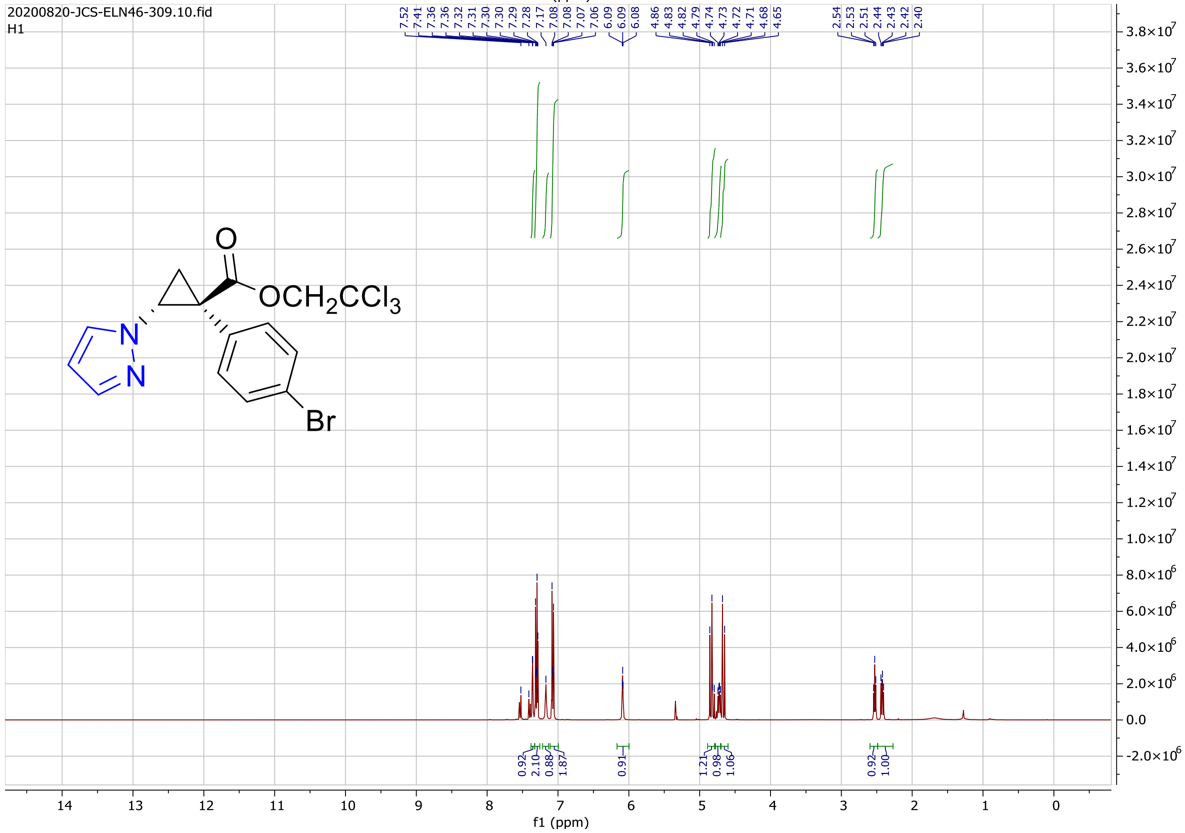
20200820-JCS-ELN46-309.10.fid
H1
7.52
7.41
7.36
7.36
7.32
7.31
7.30
7.30
7.29
7.28
7.17
7.08
7.08
7.07
7.06
6.09
6.09
6.08
4.86
4.83
4.82
4.79
4.74
4.73
4.72
4.71
4.68
4.65
2.54
2.53
2.51
2.44
2.43
2.42
2.40
0.92
2.10
0.88
1.87
0.91
1.21
0.98
1.06
0.92
1.00
O
OCH2CCl3
N
N
Br
f1 (ppm)

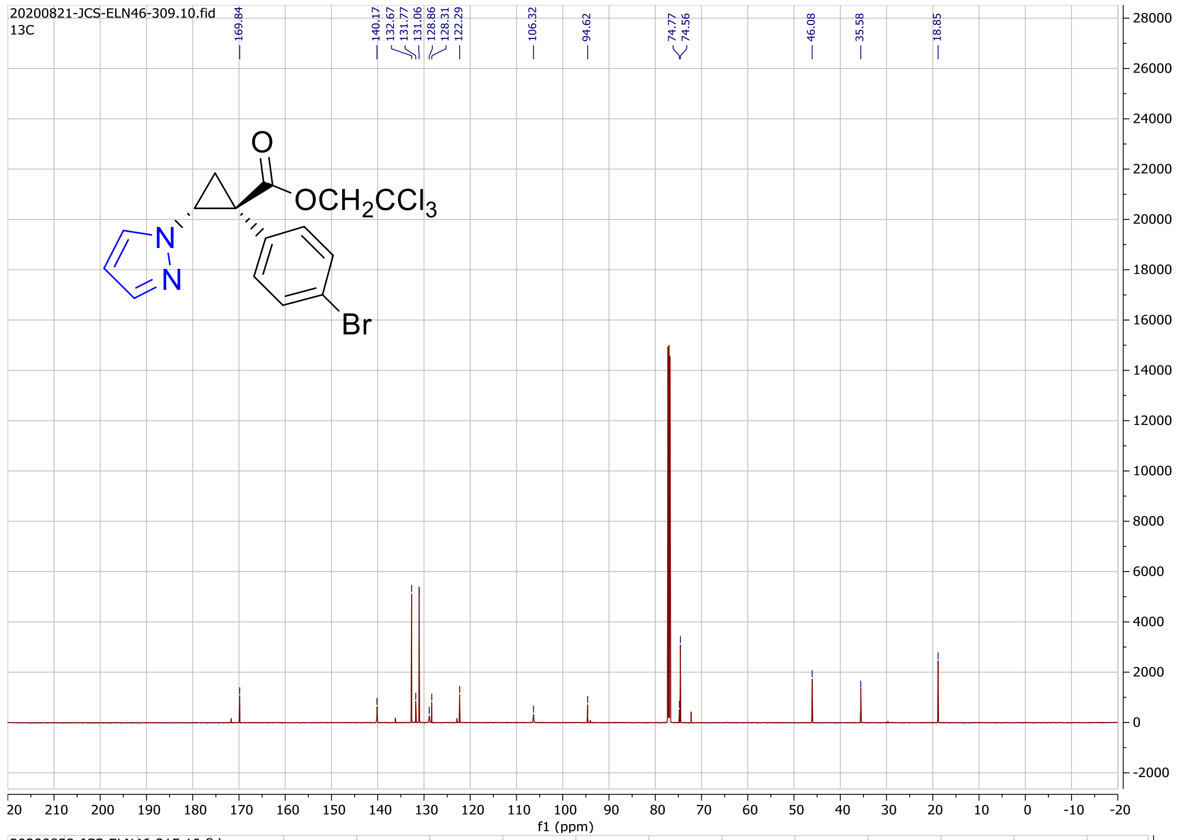

20200821-JCS-ELN46-309.10.fid
13C
169.84
140.17
132.67
131.77
131.06
128.86
128.31
122.29
106.32
94.62
74.77
74.56
46.08
35.58
18.85
O
OCH2CCl3
N
N
Br
f1 (ppm)

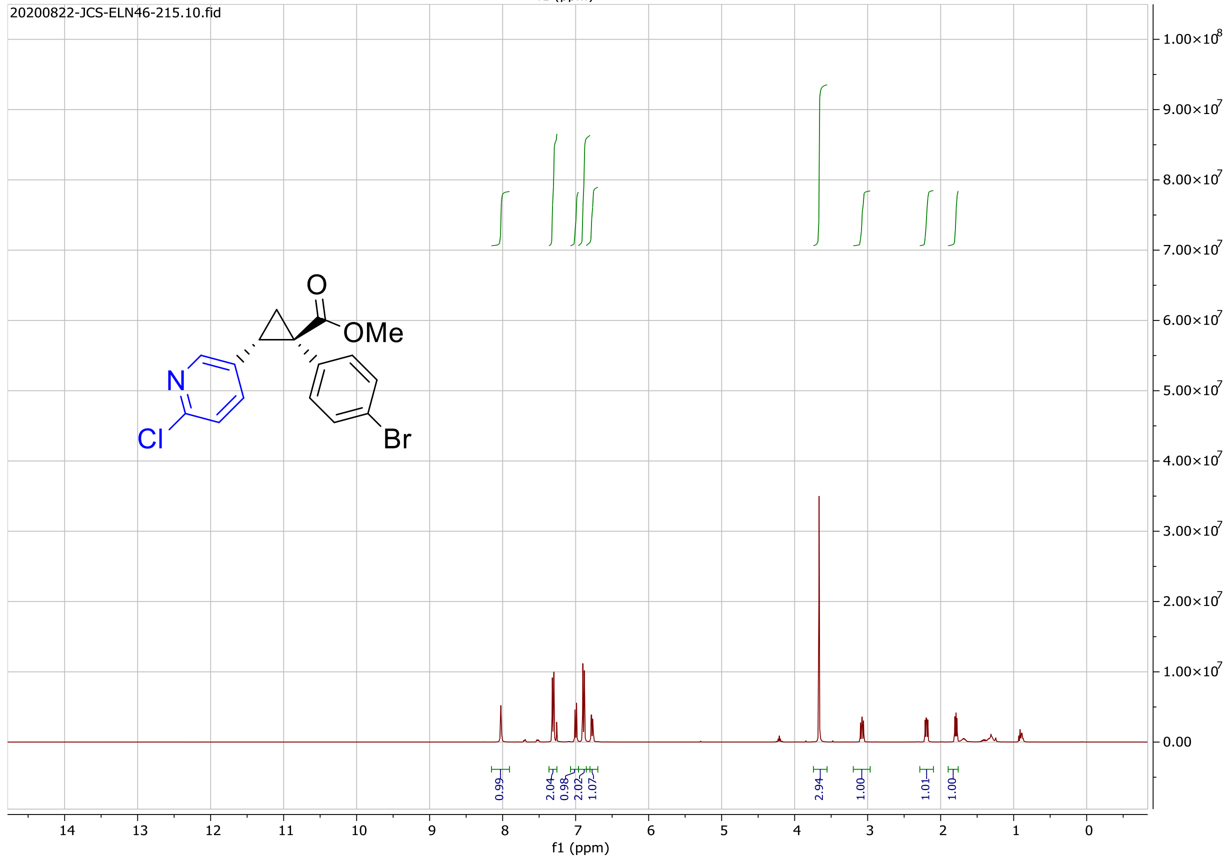

20200822-JCS-ELN46-215.10.fid
O
OMe
N
Cl
Br
0.99
2.04
0.98
2.02
1.07
2.94
1.00
1.01
1.00
f1 (ppm)

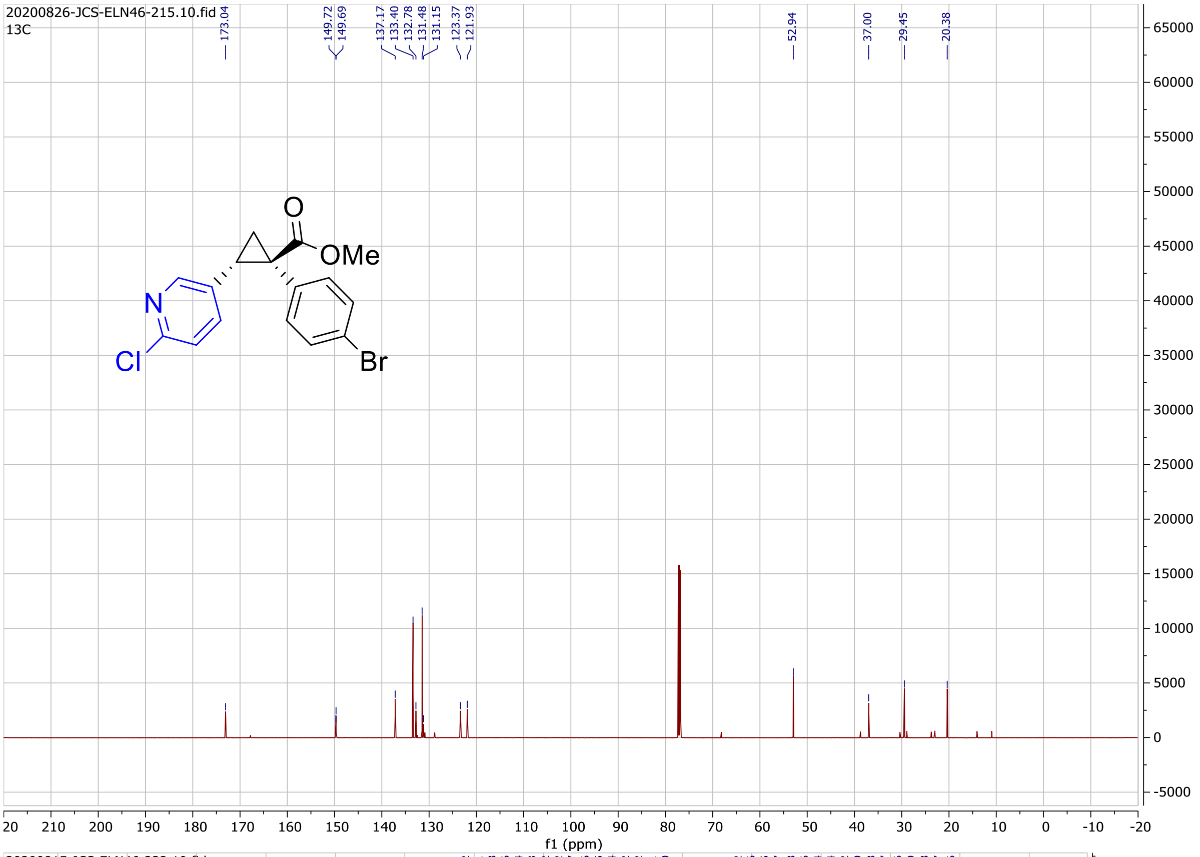
20200826-JCS-ELN46-215.10.fid
13C
173.04
149.72
149.69
137.17
133.40
132.78
131.48
131.15
123.37
121.93
52.94
37.00
29.45
20.38
O
OMe
N
Cl
Br
f1 (ppm)

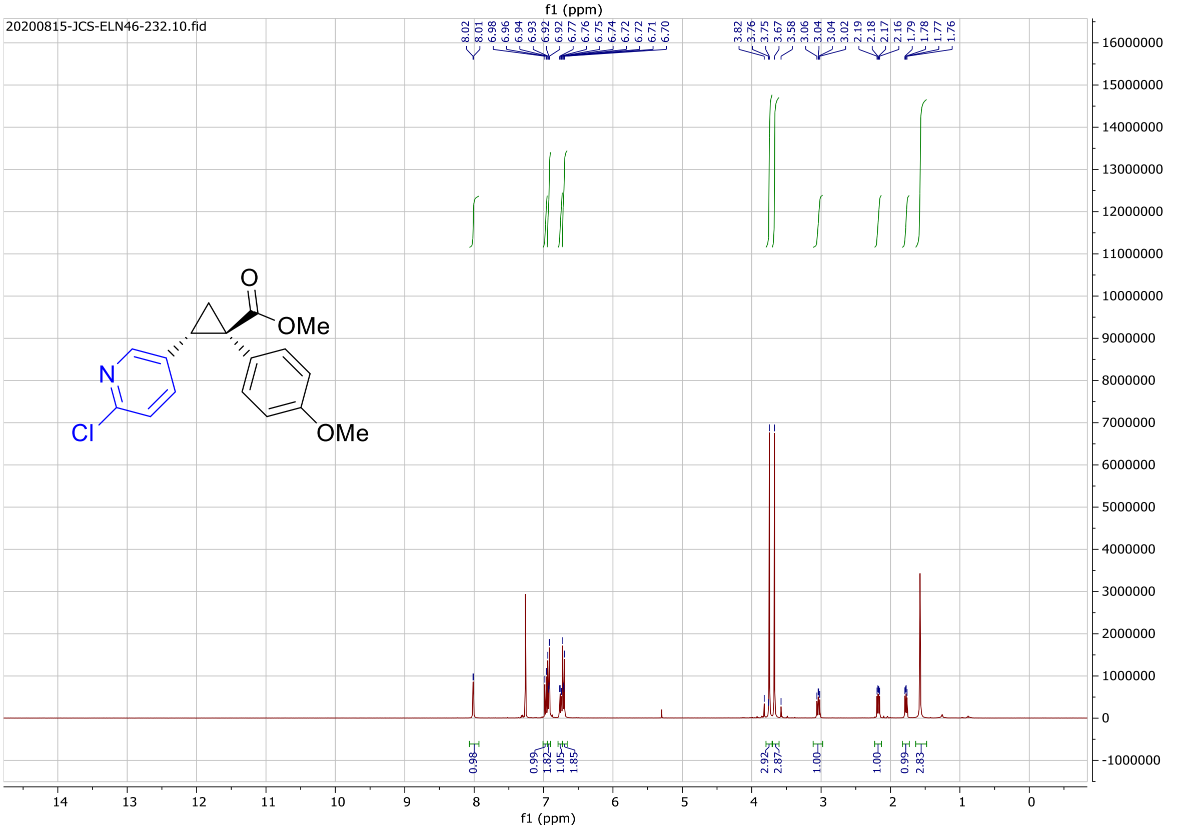
20200815-JCS-ELN46-232.10.fid
8.02
8.01
6.98
6.96
6.94
6.93
6.92
6.92
6.77
6.76
6.75
6.74
6.72
6.72
6.71
6.70
3.82
3.76
3.75
3.67
3.58
3.06
3.04
3.04
3.02
2.19
2.18
2.17
2.16
1.79
1.78
1.77
1.76
O
OMe
N
Cl
OMe
0.98
0.99
1.82
1.05
1.85
2.92
2.87
1.00
1.00
0.99
2.83
f1 (ppm)

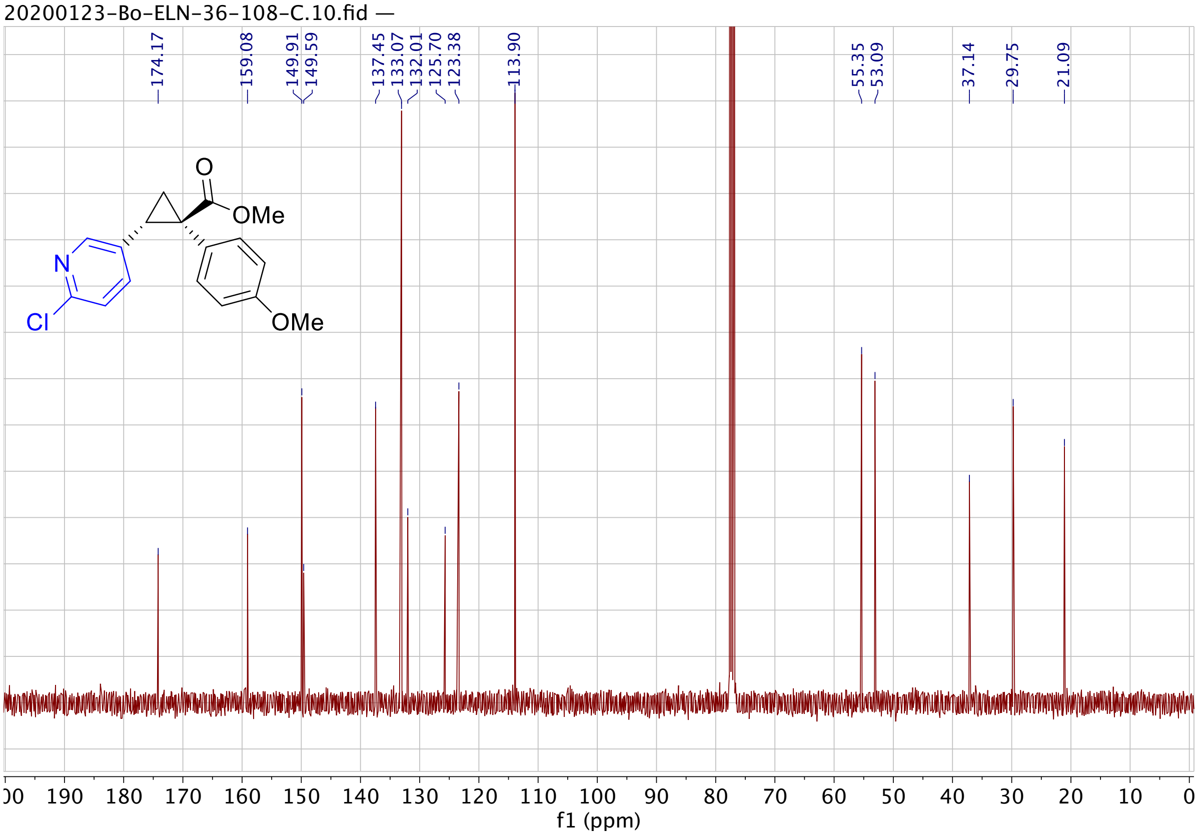
20200123-Bo-ELN-36-108-C.10.fid —
174.17
159.08
149.91
149.59
137.45
133.07
132.01
125.70
123.38
113.90
55.35
53.09
37.14
29.75
21.09
O
OMe
N
Cl
OMe
00 190 180 170 160 150 140 130 120 110 100 90 80 70 60 50 40 30 20 10 0
f1 (ppm)

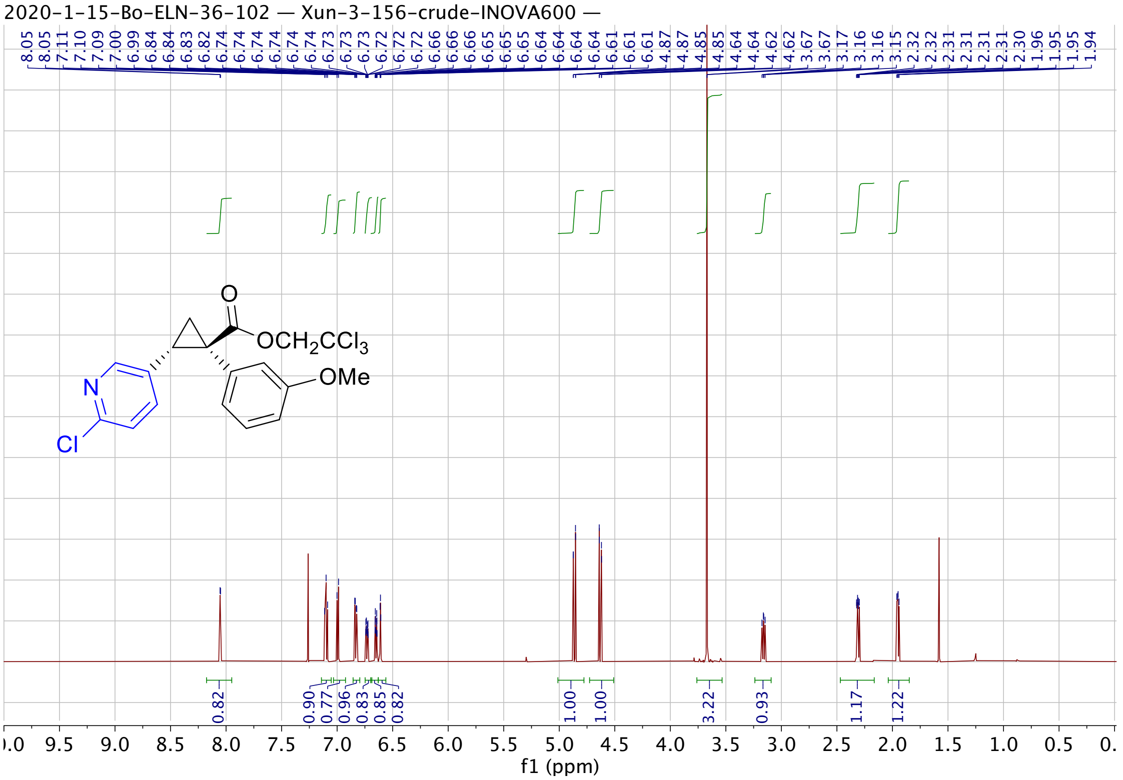
2020-1-15-Bo-ELN-36-102 — Xun-3-156-crude-INOVA600 —
8.05 8.05 7.11 7.10 7.09 7.00 6.99 6.84 6.84 6.83 6.82 6.74 6.74 6.74 6.74 6.74 6.73 6.73 6.73 6.72 6.72 6.72 6.66 6.66 6.65 6.65 6.64 6.64 6.64 6.64 6.61 6.61 6.61 4.87 4.87 4.85 4.64 4.64 4.62 3.67 3.17 3.16 3.16 3.15 2.32 2.32 2.31 2.31 2.31 2.30 1.96 1.95 1.95 1.94
O
OCH_2CCl_3
OMe
N
Cl
0.82
0.90
0.77
0.96
0.83
0.85
0.82
1.00
1.00
3.22
0.93
1.17
1.22
0.0 9.5 9.0 8.5 8.0 7.5 7.0 6.5 6.0 5.5 5.0 4.5 4.0 3.5 3.0 2.5 2.0 1.5 1.0 0.5 0.
f1 (ppm)

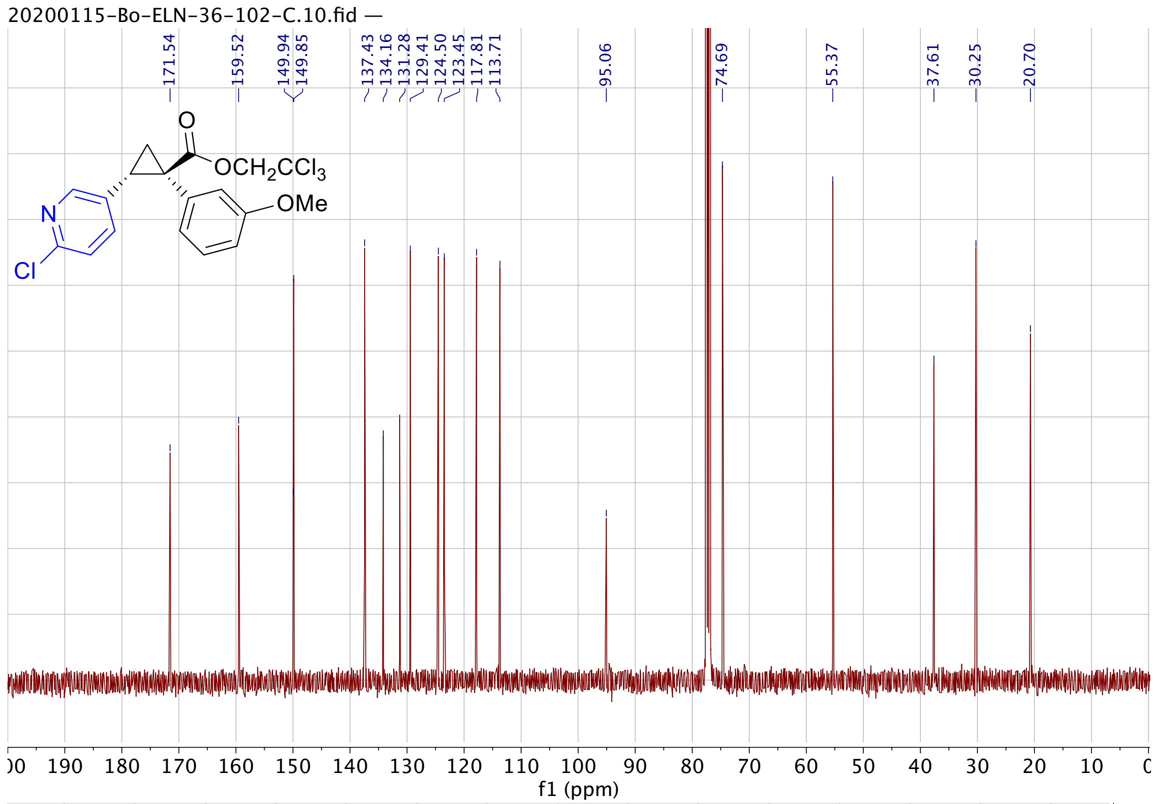
20200115-Bo-ELN-36-102-C.10.fid
171.54
159.52
149.94
149.85
137.43
134.16
131.28
129.41
124.50
123.45
117.81
113.71
95.06
74.69
55.37
37.61
30.25
20.70
O
OCH_2CCl_3
OMe
N
Cl
f1 (ppm)

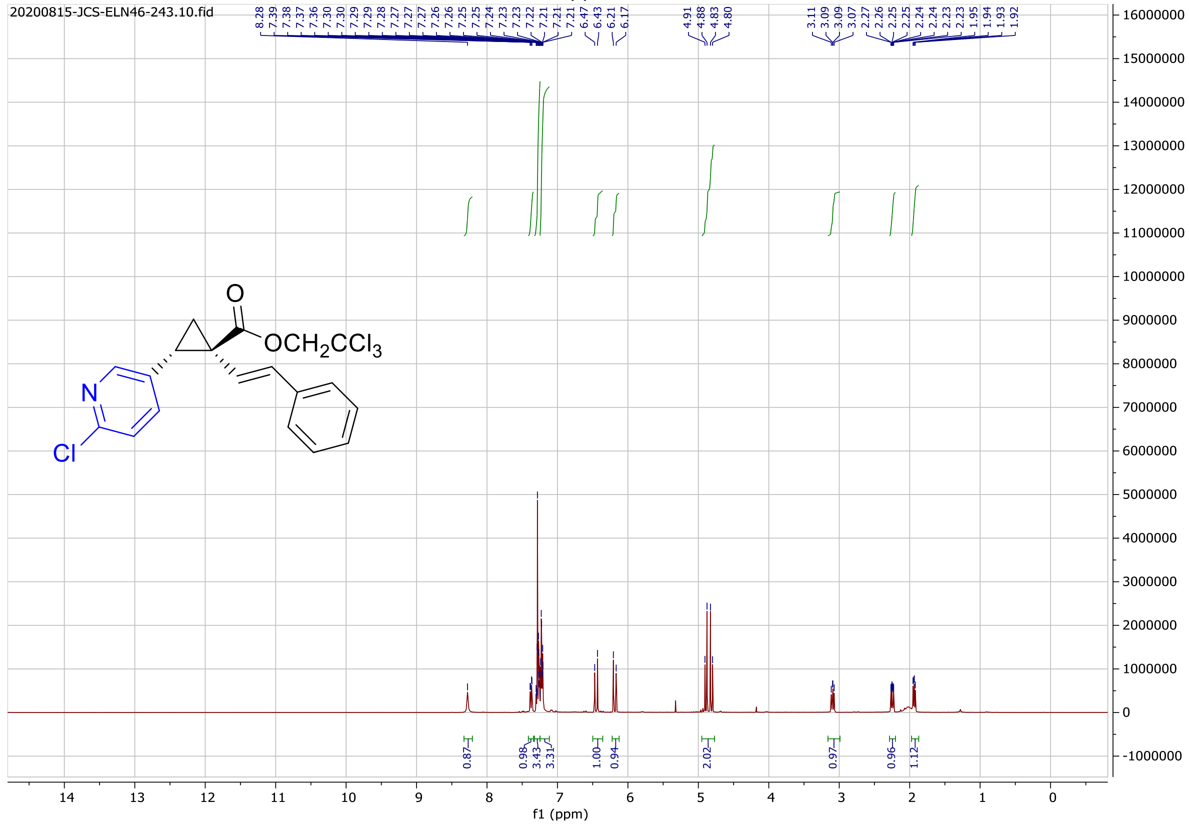
20200815-JCS-ELN46-243.10.fid
8.28
7.39
7.38
7.37
7.36
7.30
7.30
7.29
7.29
7.28
7.27
7.27
7.27
7.26
7.26
7.25
7.25
7.24
7.23
7.23
7.22
7.22
7.21
7.21
7.21
6.47
6.43
6.21
6.17
4.91
4.88
4.83
4.80
3.11
3.09
3.09
3.07
2.27
2.26
2.25
2.25
2.24
2.24
2.23
2.23
1.95
1.94
1.93
1.92
O
OCH_2CCl_3
N
Cl
0.87
0.98
3.43
3.31
1.00
0.94
2.02
0.97
0.96
1.12
f1 (ppm)

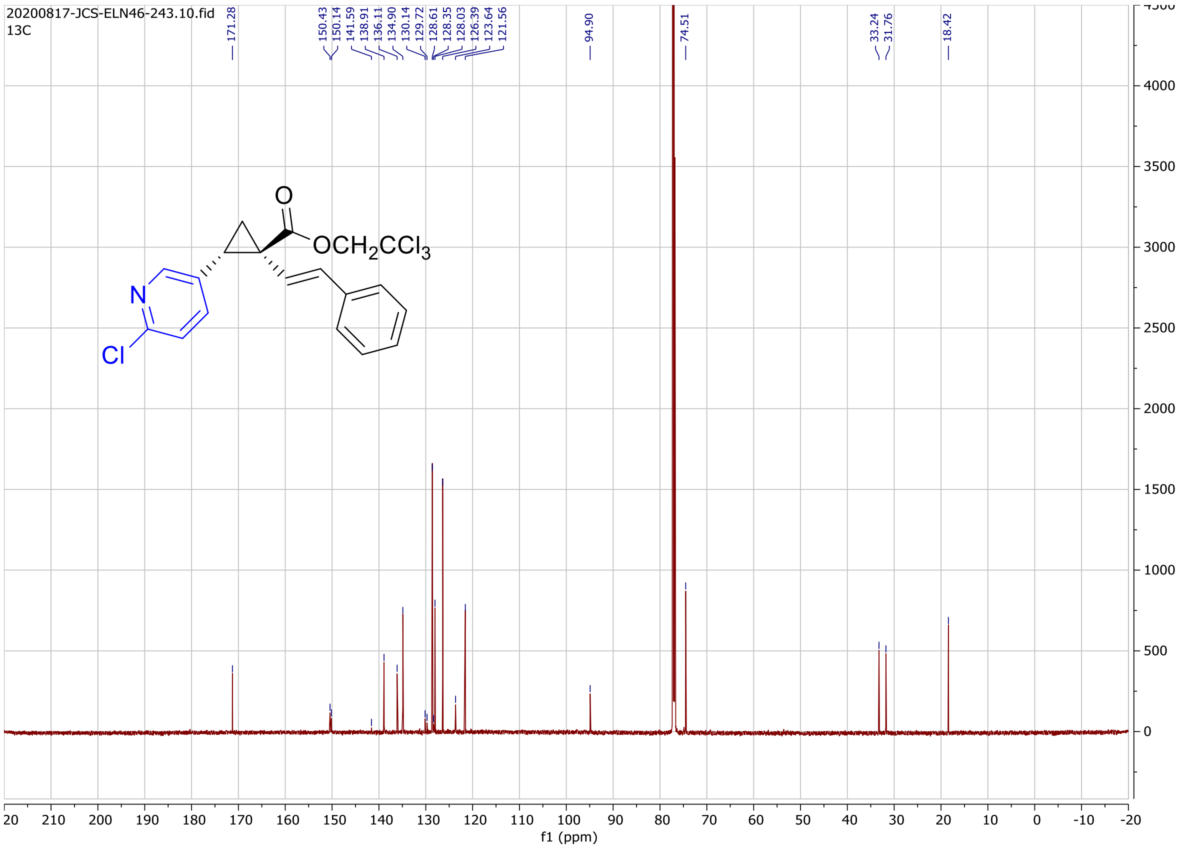
20200817-JCS-ELN46-243.10.fid
13C
OCH2CCl3
N
Cl
O
f1 (ppm)

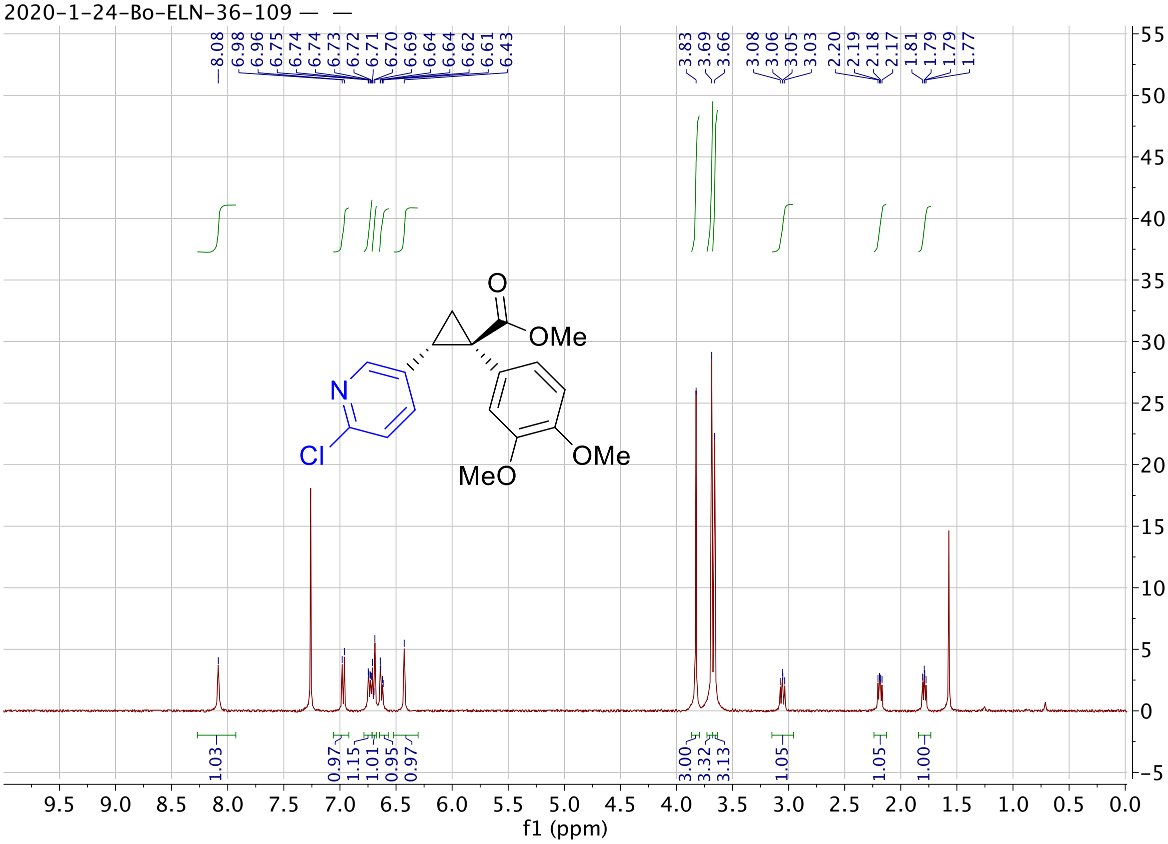
2020-1-24-Bo-ELN-36-109 — —
OMe
N
Cl
MeO
OMe
O
f1 (ppm)

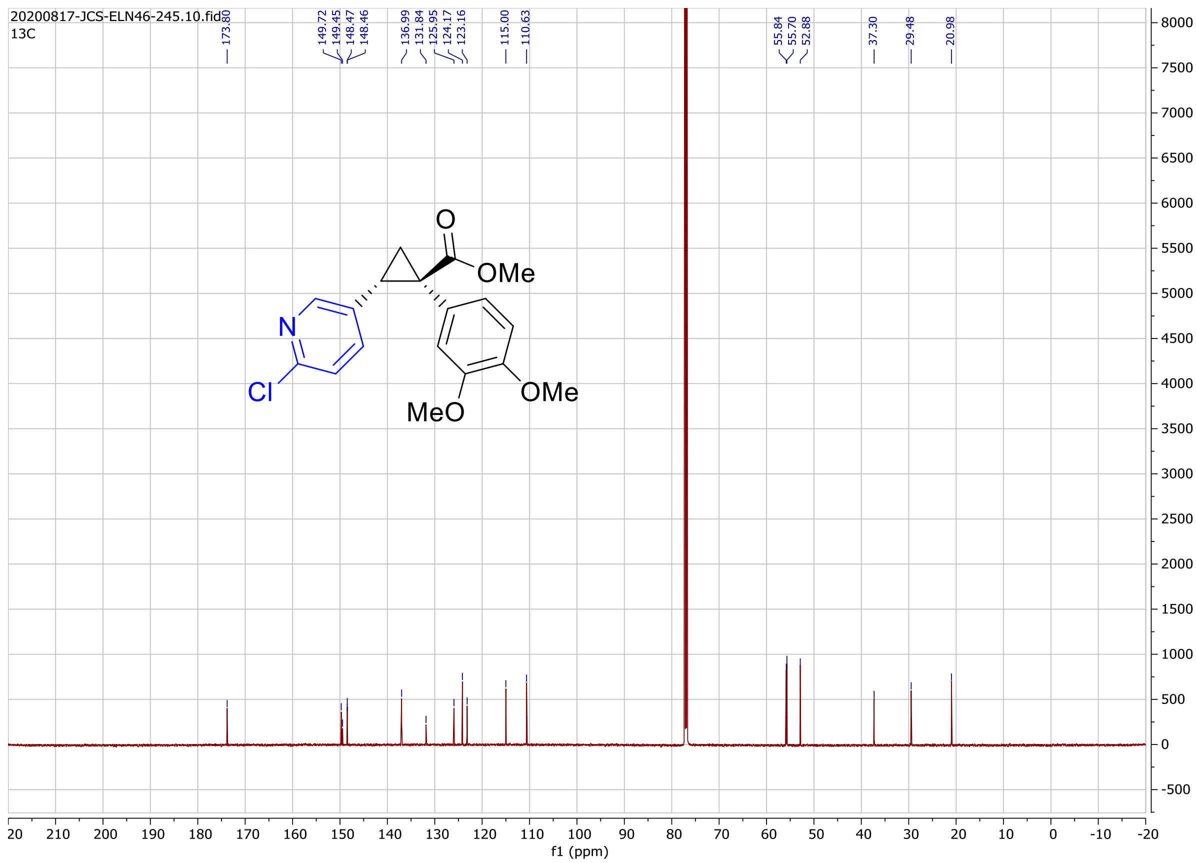
20200817-JCS-ELN46-245.10.fid
13C
173.80
149.72
149.45
148.47
148.46
136.99
131.84
125.95
124.17
123.16
115.00
110.63
55.84
55.70
52.88
37.30
29.48
20.98
O
OMe
N
Cl
MeO
OMe
f1 (ppm)

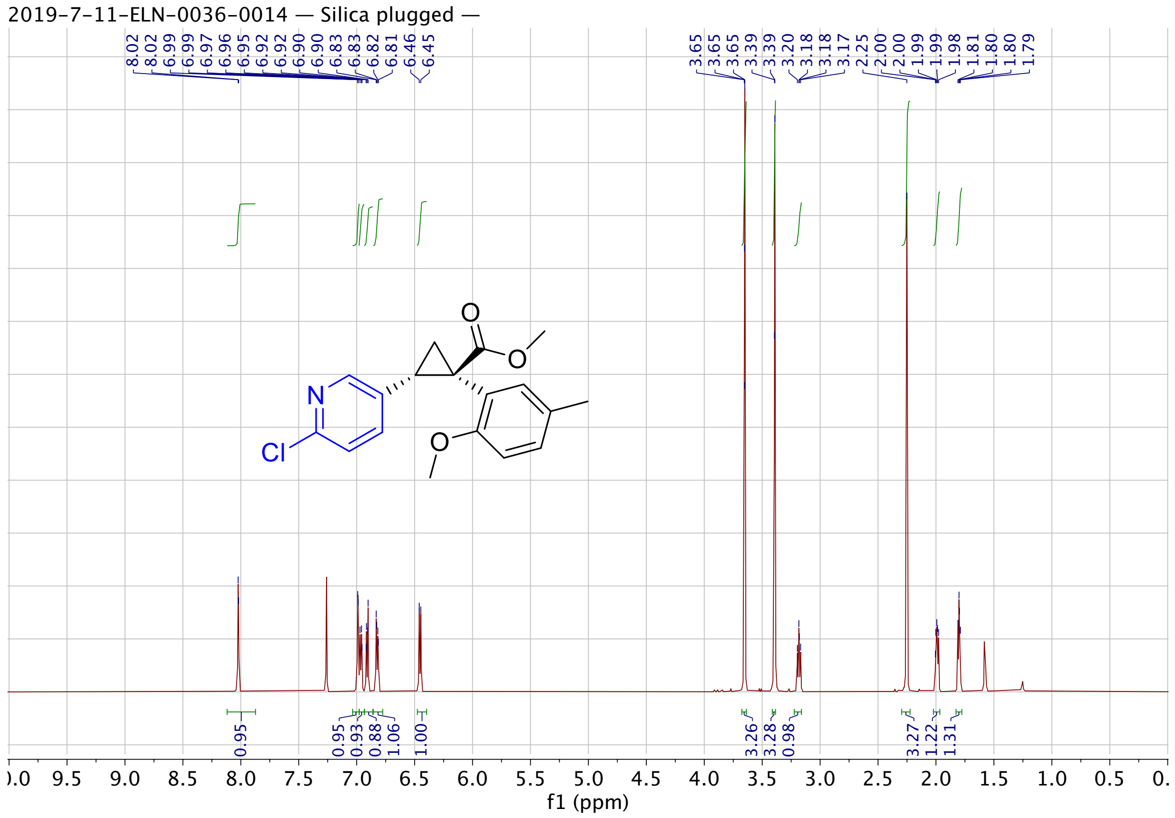
2019-7-11-ELN-0036-0014 — Silica plugged —
8.02
8.02
6.99
6.99
6.97
6.96
6.95
6.92
6.92
6.90
6.90
6.83
6.83
6.82
6.81
6.46
6.45
3.65
3.65
3.65
3.39
3.39
3.20
3.18
3.18
3.17
2.25
2.00
2.00
1.99
1.99
1.98
1.81
1.80
1.80
1.79
O
O
N
Cl
O
0.95
0.95
0.93
0.88
1.06
1.00
3.26
3.28
0.98
3.27
1.22
1.31
f1 (ppm)

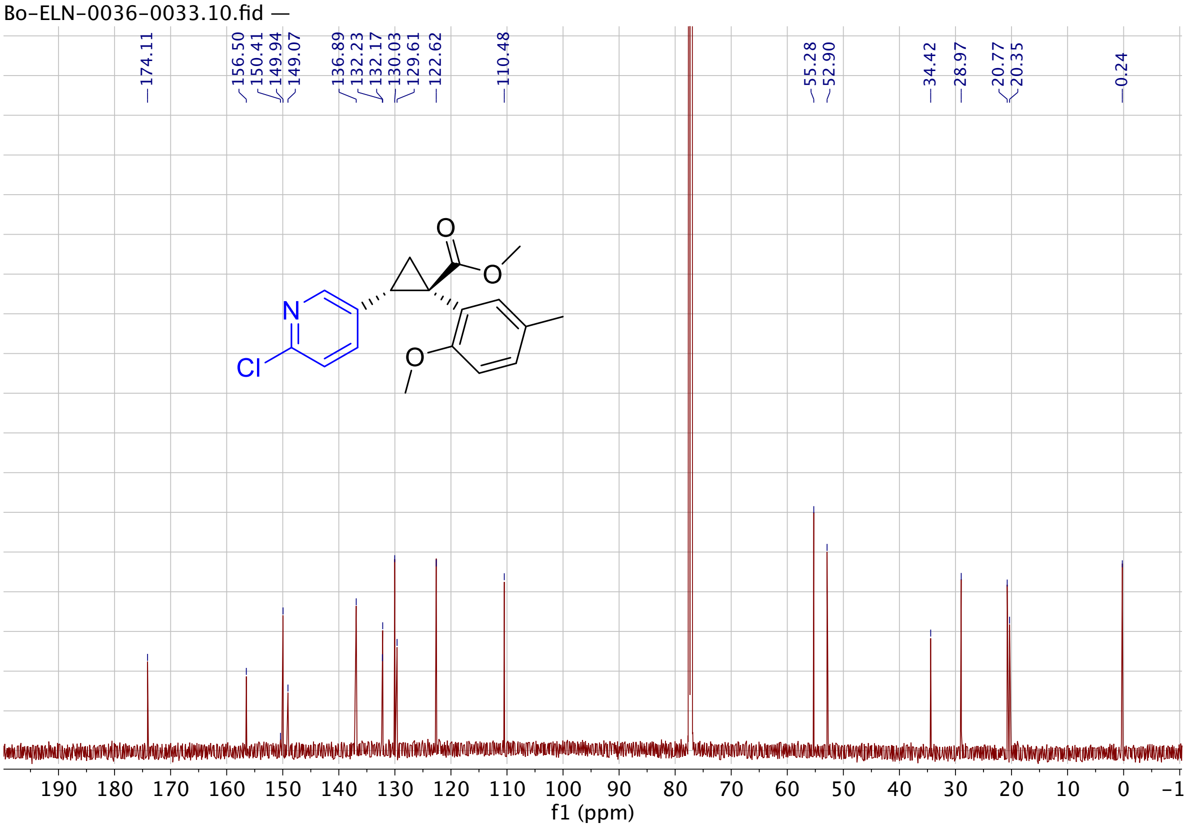
Bo-ELN-0036-0033.10.fid —
174.11
156.50
150.41
149.94
149.07
136.89
132.23
132.17
130.03
129.61
122.62
110.48
55.28
52.90
34.42
28.97
20.77
20.35
0.24
O
O
N
Cl
O
190 180 170 160 150 140 130 120 110 100 90 80 70 60 50 40 30 20 10 0 -1
f1 (ppm)

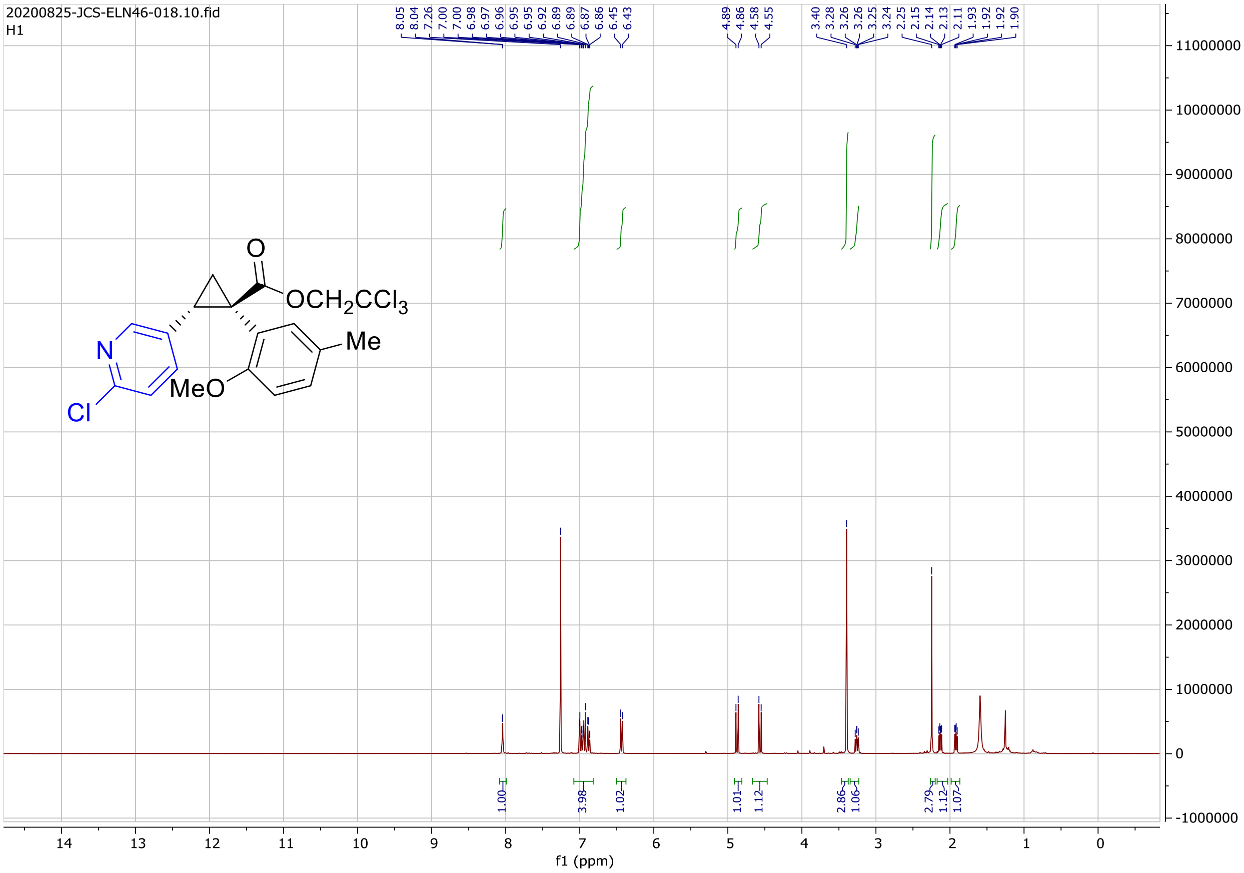
20200825-JCS-ELN46-018.10.fid
H1
8.05
8.04
7.26
7.00
7.00
6.98
6.97
6.96
6.96
6.95
6.95
6.92
6.89
6.87
6.86
6.45
6.43
4.89
4.86
4.58
4.55
3.40
3.28
3.26
3.26
3.25
3.24
2.25
2.15
2.14
2.13
2.11
1.93
1.92
1.92
1.90
O
OCH2CCl3
N
Me
Cl
MeO
1.00
3.98
1.02
1.01
1.12
2.86
1.06
2.79
1.12
1.07
11000000
10000000
9000000
8000000
7000000
6000000
5000000
4000000
3000000
2000000
1000000
0
-1000000
14 13 12 11 10 9 8 7 6 5 4 3 2 1 0
f1 (ppm)

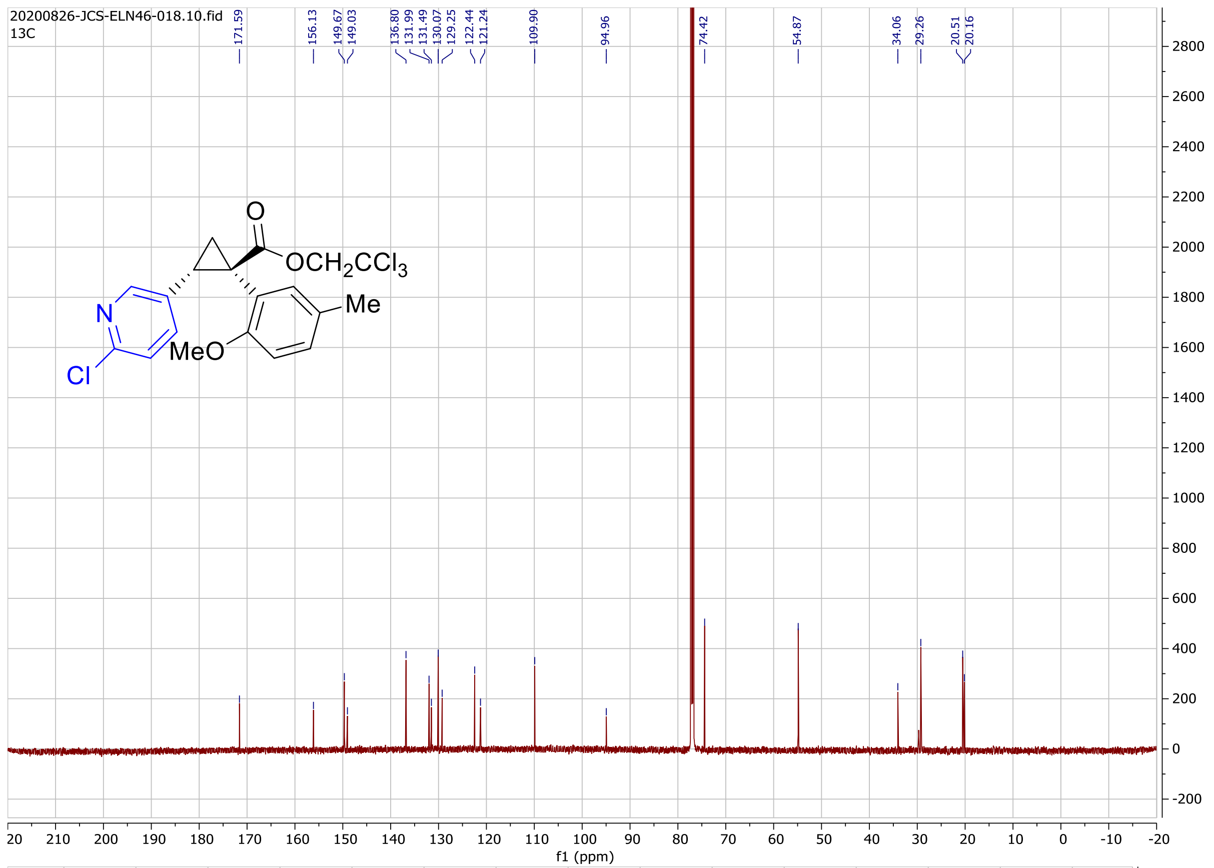

20200826-JCS-ELN46-018.10.fid
13C
171.59
156.13
149.67
149.03
136.80
131.99
131.49
130.07
129.25
122.44
121.24
109.90
94.96
74.42
54.87
34.06
29.26
20.51
20.16
OCH2CCl3
Me
MeO
N
Cl
O
f1 (ppm)

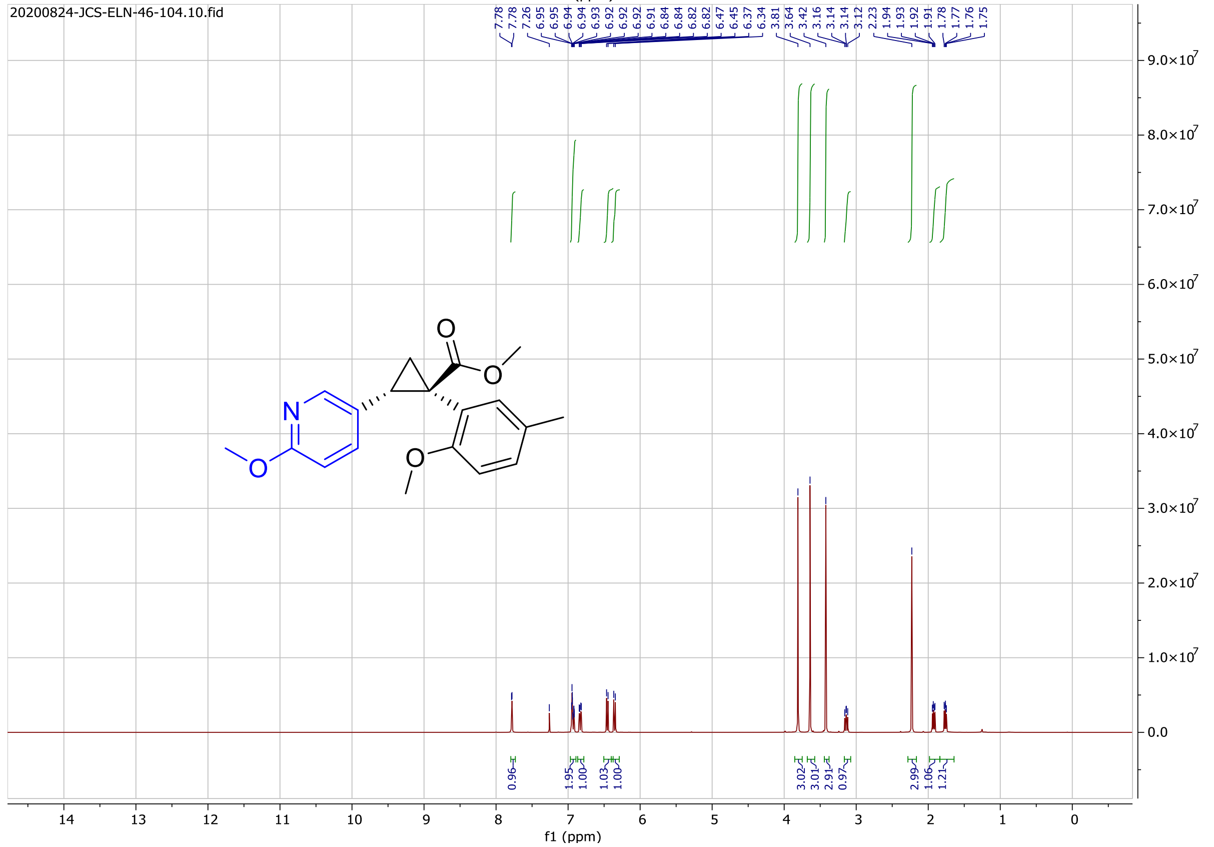

20200824-JCS-ELN-46-104.10.fid
7.78
7.78
7.26
6.95
6.94
6.93
6.92
6.92
6.92
6.91
6.84
6.84
6.82
6.82
6.47
6.45
6.37
6.34
3.81
3.64
3.42
3.16
3.14
3.14
3.12
2.23
1.94
1.93
1.92
1.91
1.78
1.77
1.76
1.75
0.96
1.95
1.00
1.03
1.00
3.02
3.01
2.91
0.97
2.99
1.06
1.21
N
O
f1 (ppm)

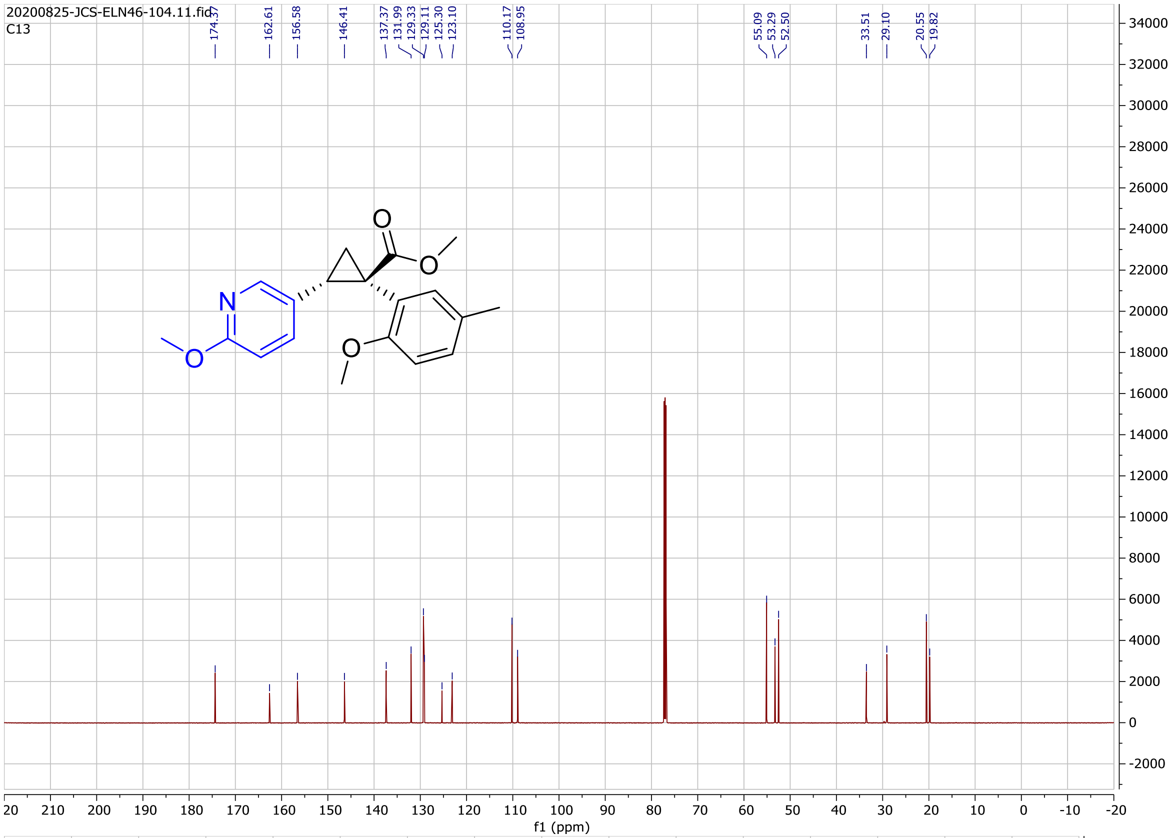

20200825-JCS-ELN46-104.11.fid
C13
174.37
162.61
156.58
146.41
137.37
131.99
129.33
129.11
125.30
123.10
110.17
108.95
55.09
53.29
52.50
33.51
29.10
20.55
19.82
N
O
O
O
O
f1 (ppm)

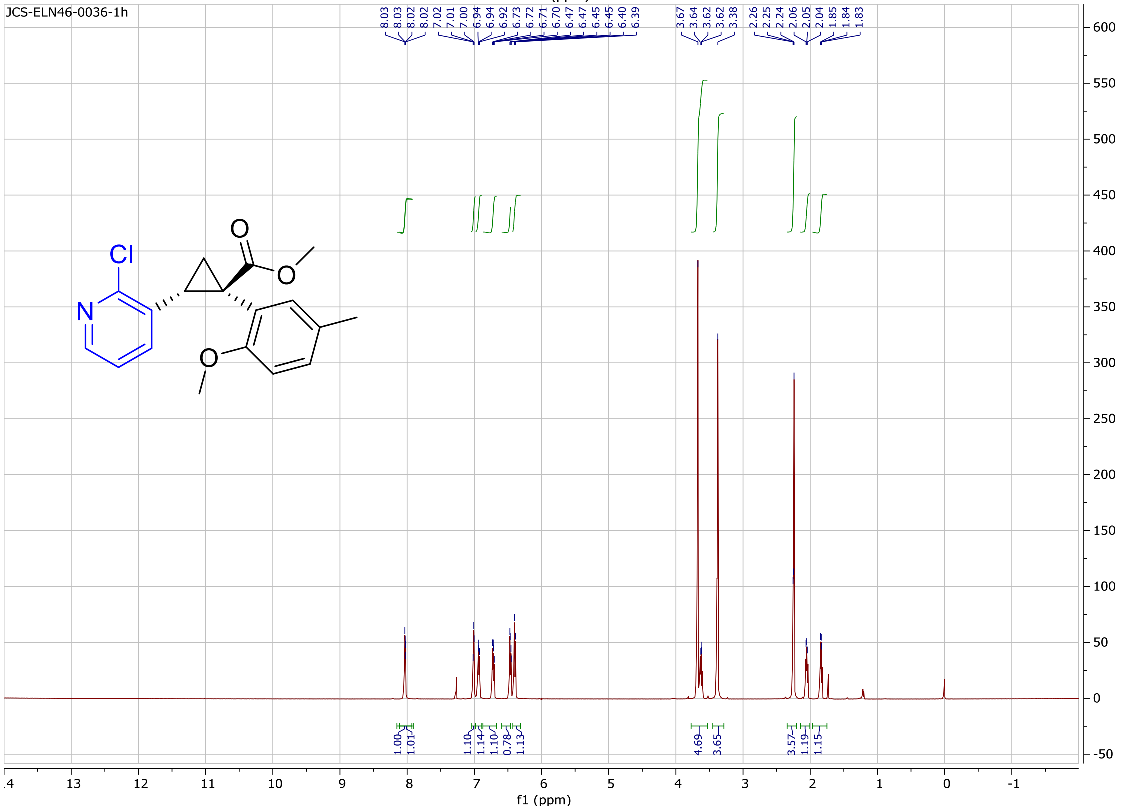

JCS-ELN46-0036-1h
8.03
8.03
8.02
8.02
7.02
7.01
7.00
6.94
6.92
6.73
6.72
6.71
6.70
6.47
6.47
6.45
6.45
6.40
6.39
3.67
3.64
3.62
3.62
3.38
2.26
2.25
2.24
2.06
2.05
2.04
1.85
1.84
1.83
Cl
N
O
O
O
1.00
1.01
1.10
1.14
1.10
0.78
1.13
4.69
3.65
3.57
1.19
1.15
f1 (ppm)

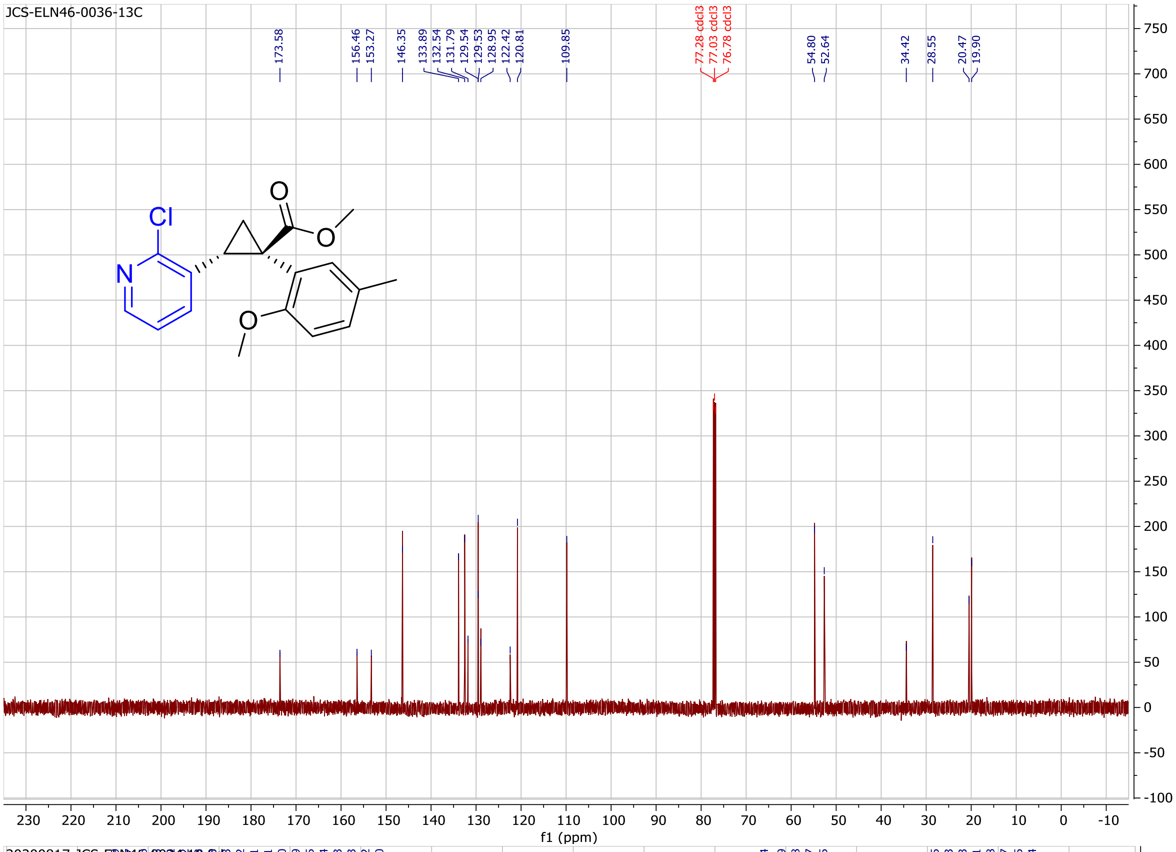
JCS-ELN46-0036-13C
173.58
156.46
153.27
146.35
133.89
132.54
131.79
129.54
129.53
128.95
122.42
120.81
109.85
77.28 cdcl3
77.03 cdcl3
76.78 cdcl3
54.80
52.64
34.42
28.55
20.47
19.90
Cl
N
O
O
O
f1 (ppm)

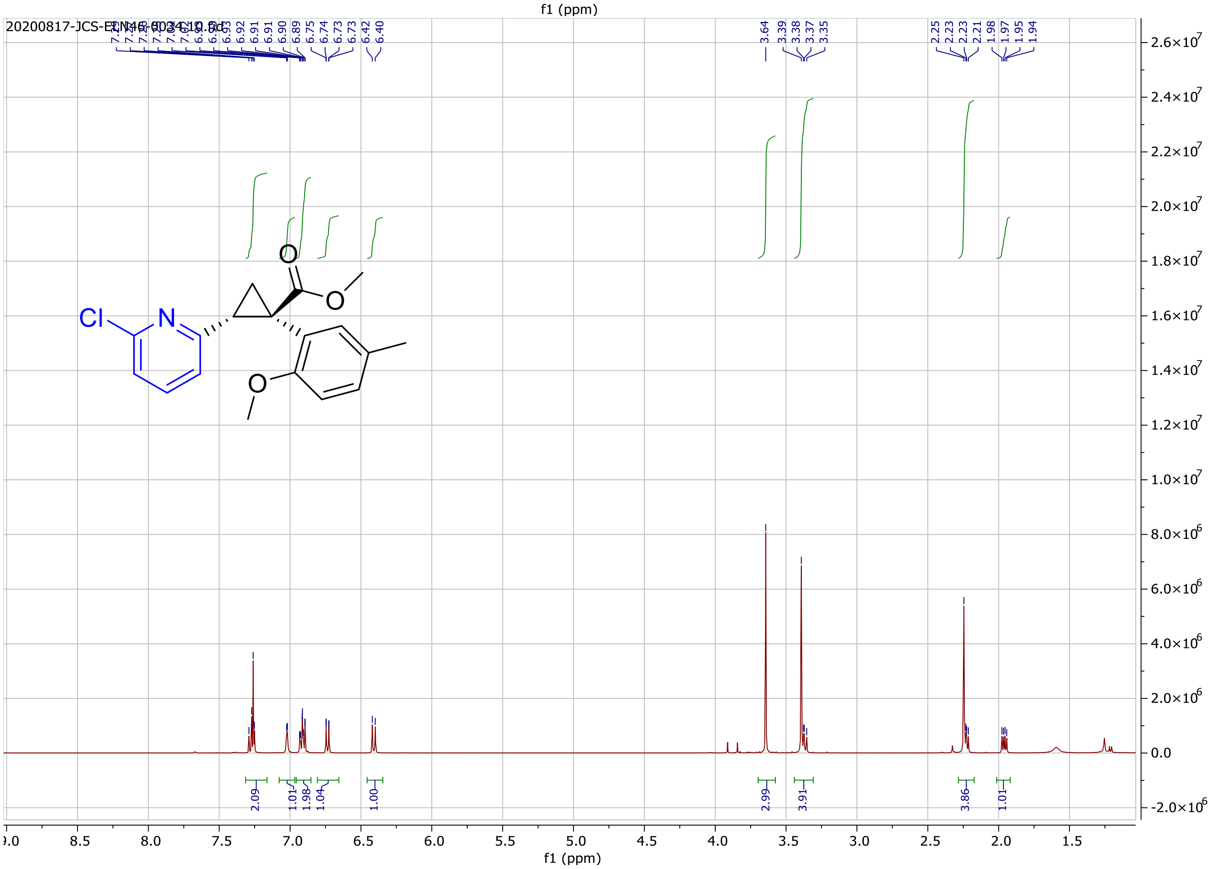
6.92
6.91
6.91
6.90
6.89
6.75
6.74
6.73
6.73
6.42
6.40
3.64
3.39
3.38
3.37
3.35
2.25
2.23
2.23
2.21
1.98
1.97
1.95
1.94
Cl
N
O
O
O
2.09
1.01
1.98
1.04
1.00
2.99
3.91
3.86
1.01
f1 (ppm)

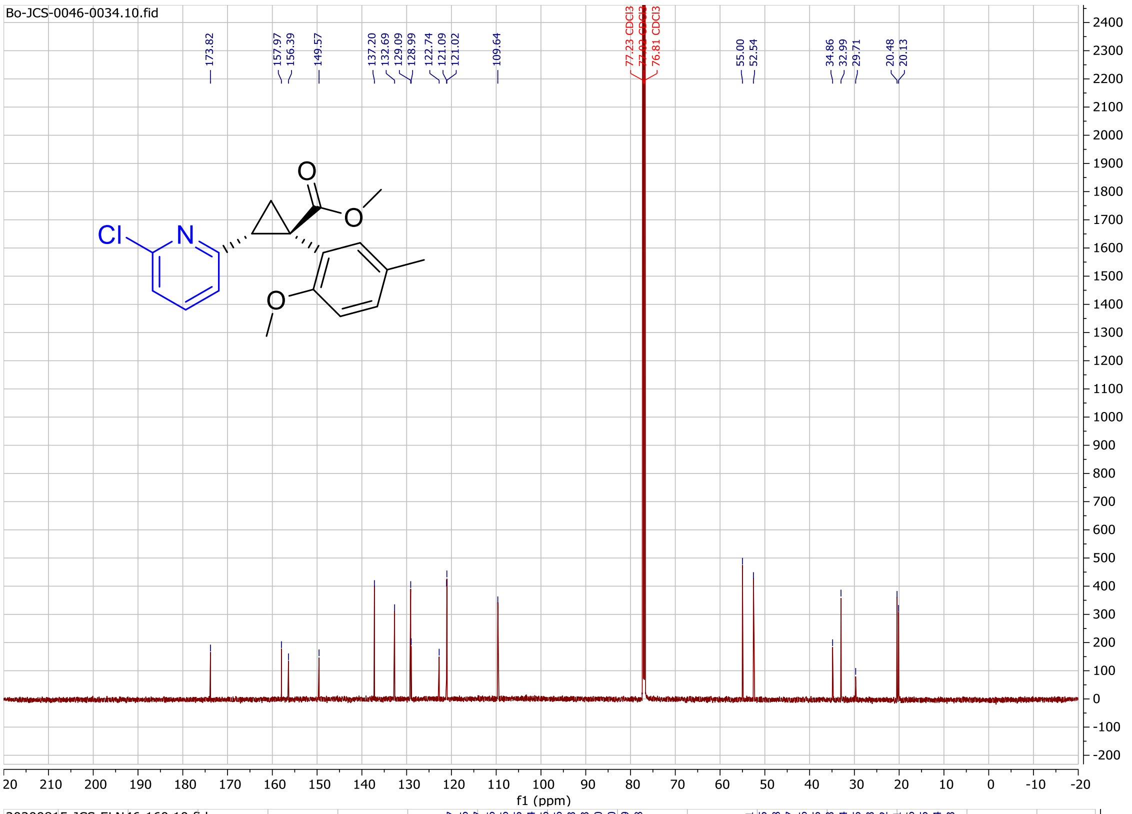
Bo-JCS-0046-0034.10.fid
173.82
157.97
156.39
149.57
137.20
132.69
129.09
128.99
122.74
121.09
121.02
109.64
77.23 CDCl3
76.81 CDCl3
55.00
52.54
34.86
32.99
29.71
20.48
20.13
Cl
N
O
f1 (ppm)

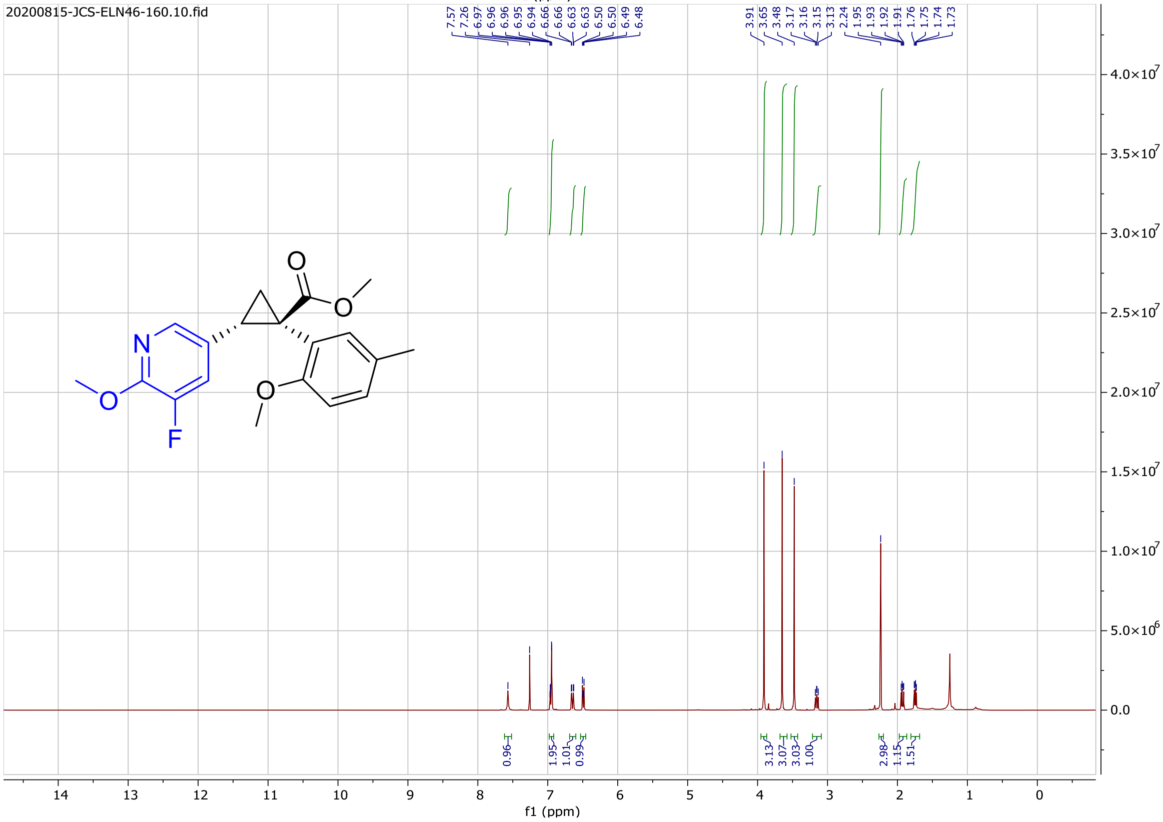
20200815-JCS-ELN46-160.10.fid
7.57
7.26
6.97
6.96
6.96
6.95
6.94
6.66
6.66
6.63
6.63
6.50
6.50
6.49
6.48
3.91
3.65
3.48
3.17
3.16
3.15
3.13
2.24
1.95
1.93
1.92
1.91
1.76
1.75
1.74
1.73
0.96
1.95
1.01
0.99
3.13
3.07
3.03
1.00
2.98
1.15
1.51
N
O
F
f1 (ppm)

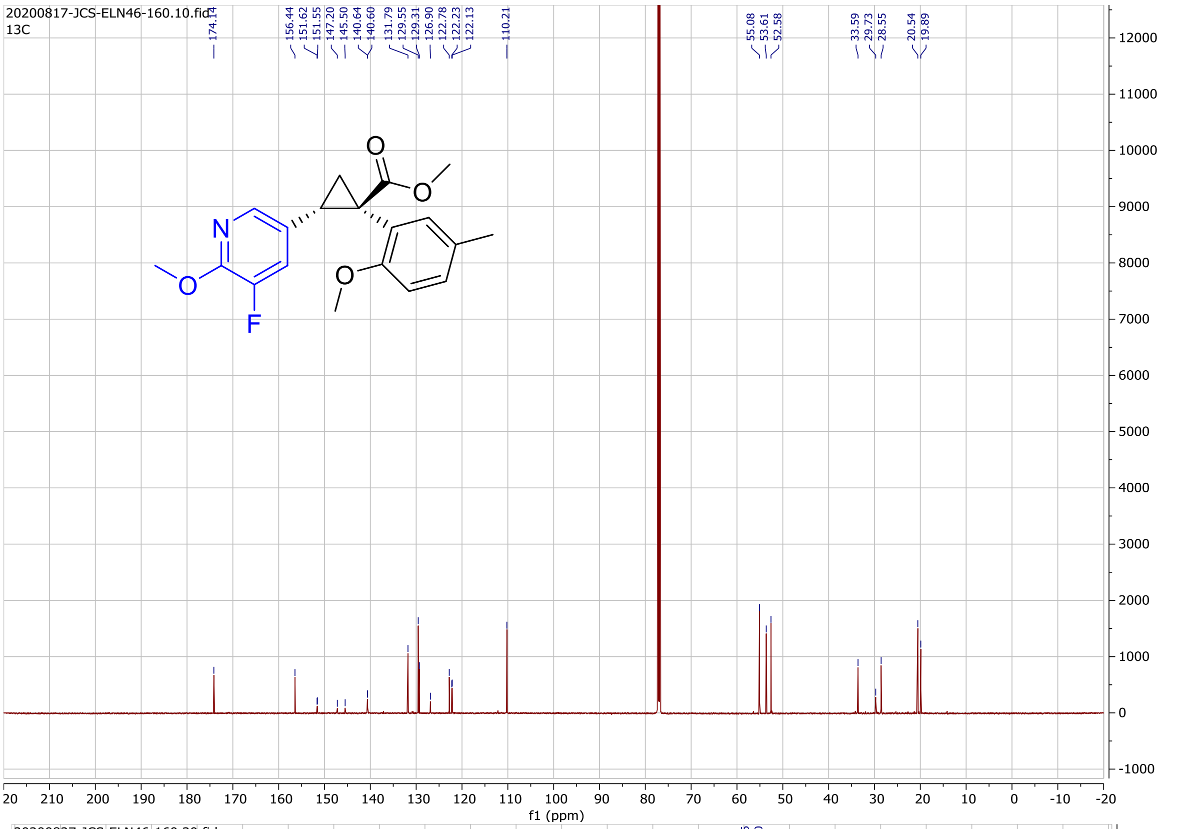
20200817-JCS-ELN46-160.10.fid
13C
174.14
156.44
151.62
151.55
147.20
145.50
140.64
140.60
131.79
129.55
129.31
126.90
122.78
122.23
122.13
110.21
55.08
53.61
52.58
33.59
29.73
28.55
20.54
19.89
f1 (ppm)

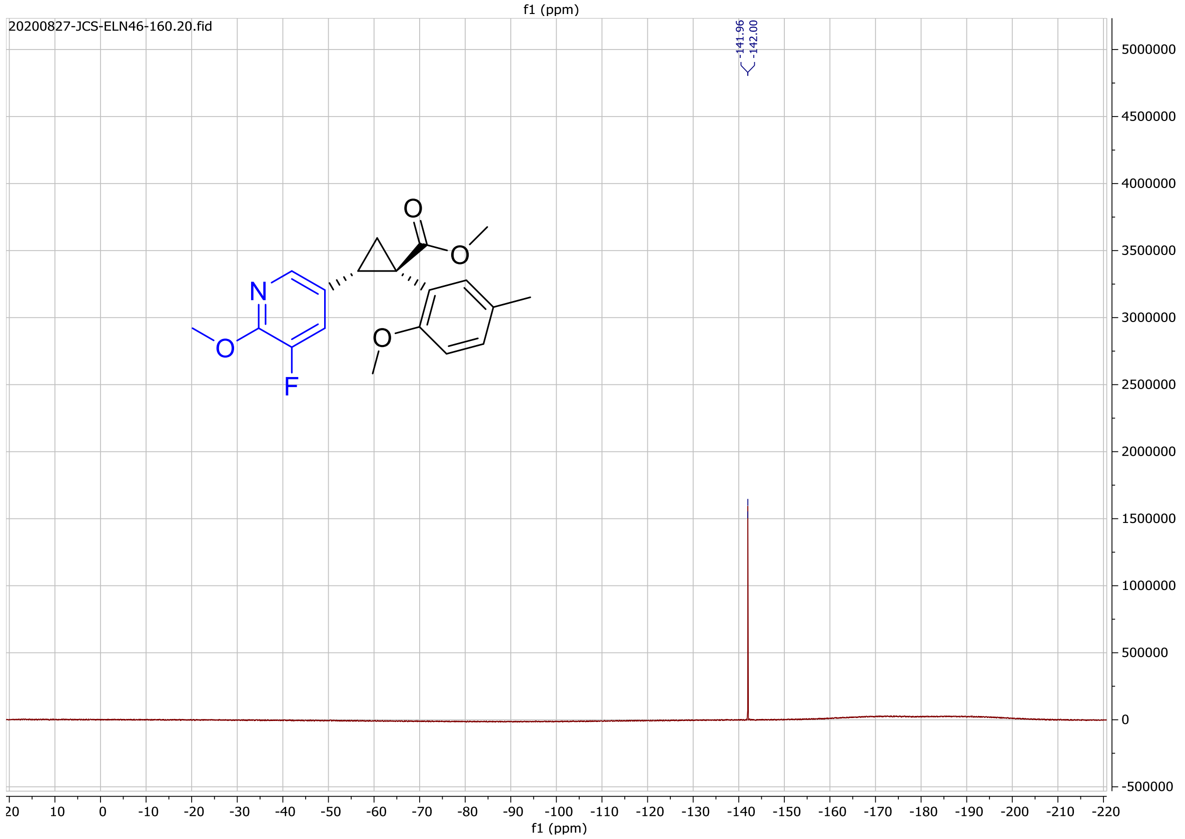
20200827-JCS-ELN46-160.20.fid
-141.96
-142.00
f1 (ppm)

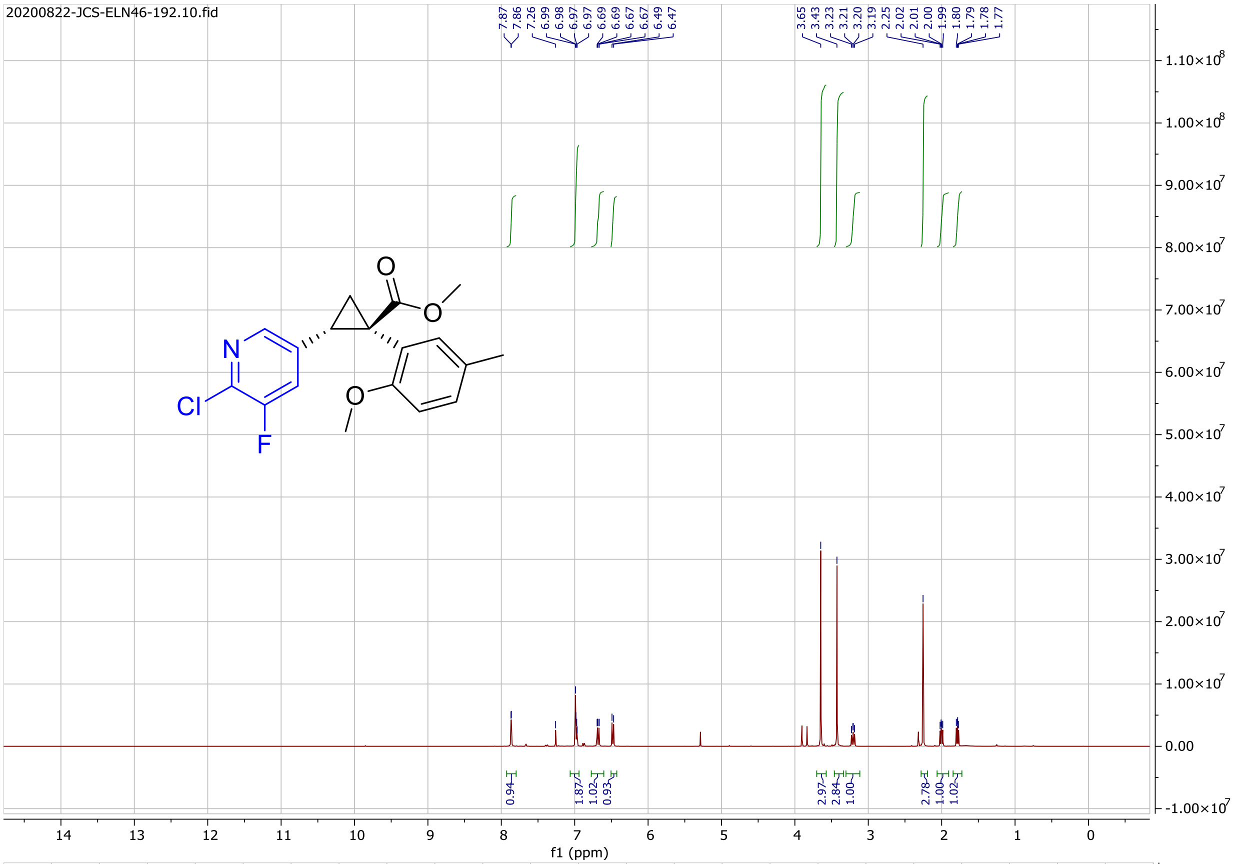
20200822-JCS-ELN46-192.10.fid
7.87
7.86
7.26
6.99
6.98
6.97
6.97
6.69
6.69
6.67
6.67
6.49
6.47
3.65
3.43
3.23
3.21
3.20
3.19
2.25
2.02
2.01
2.00
1.99
1.80
1.79
1.78
1.77
N
Cl
F
O
O
O
0.94
1.87
1.02
0.93
2.97
2.84
1.00
2.78
1.00
1.02
f1 (ppm)

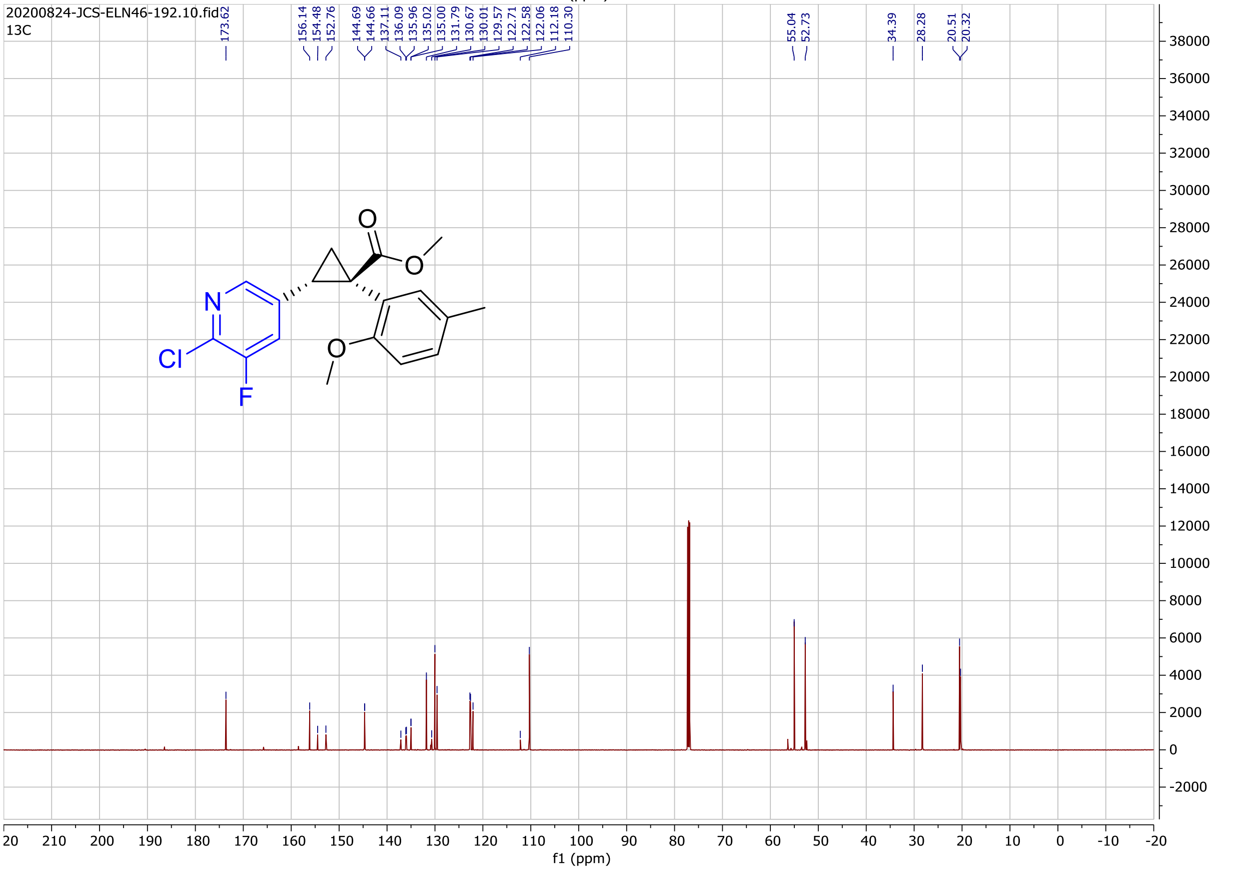
20200824-JCS-ELN46-192.10.fid
13C
173.62
156.14
154.48
152.76
144.69
144.66
137.11
136.09
135.96
135.02
135.00
131.79
130.67
130.01
129.57
122.71
122.58
122.06
112.18
110.30
55.04
52.73
34.39
28.28
20.51
20.32
N
Cl
F
O
O
O
f1 (ppm)

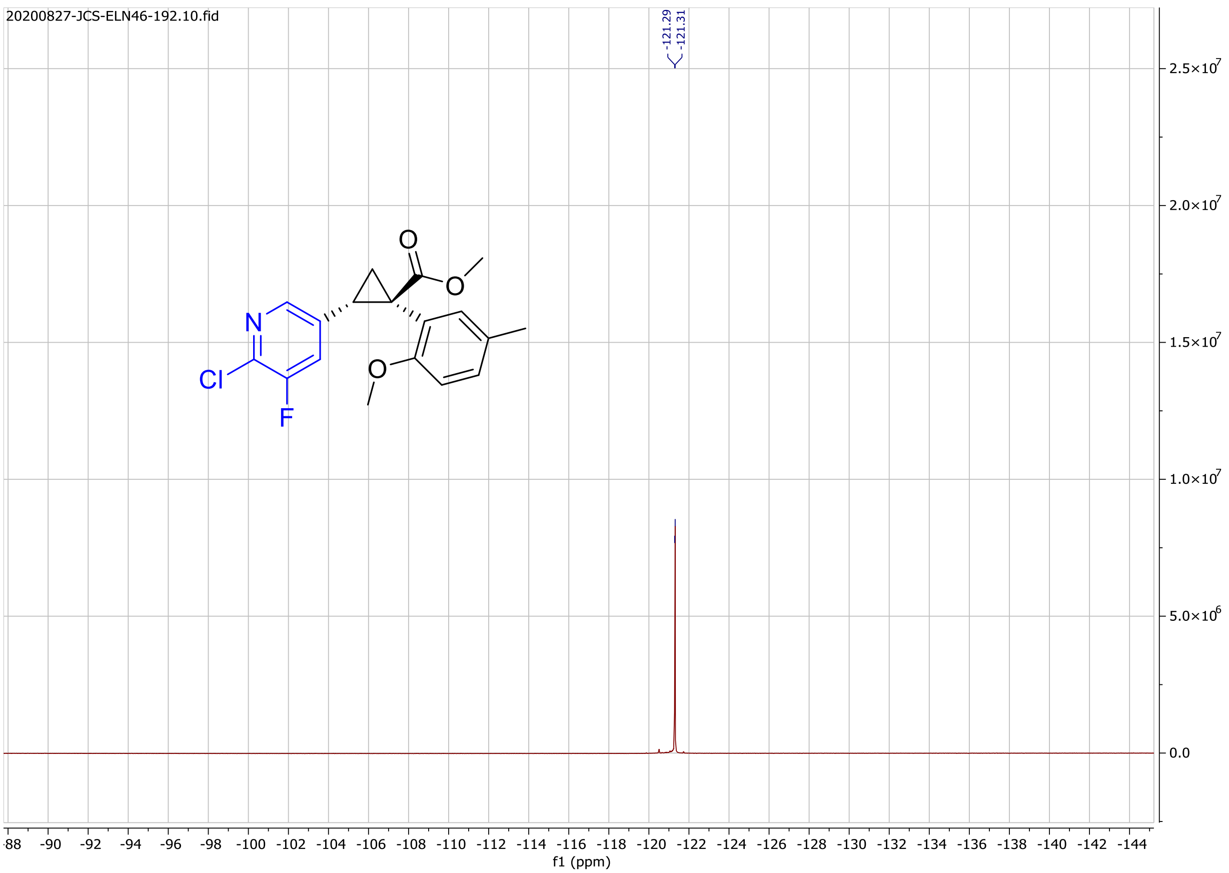

20200827-JCS-ELN46-192.10.fid
-121.29
-121.31
O
O
N
Cl
F
O
f1 (ppm)

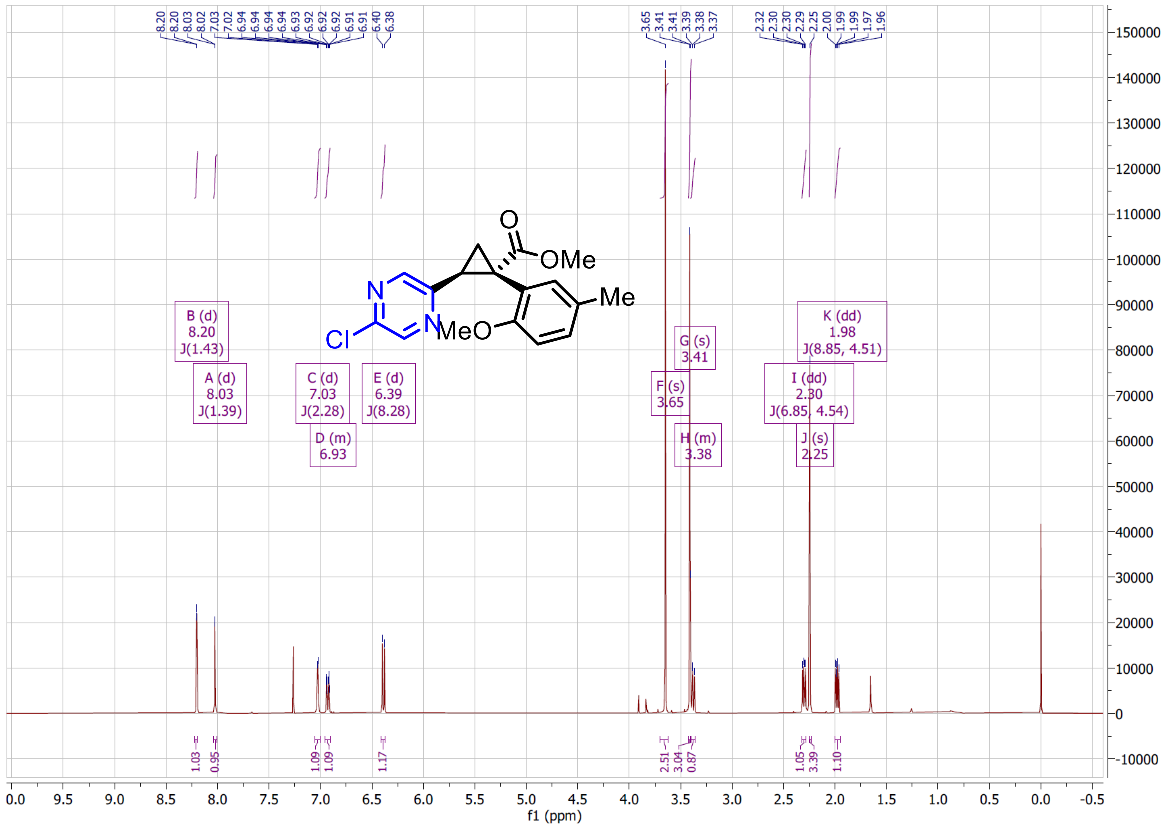

O
OMe
N
Cl
N
MeO
Me
B (d)
8.20
J(1.43)
A (d)
8.03
J(1.39)
C (d)
7.03
J(2.28)
D (m)
6.93
E (d)
6.39
J(8.28)
G (s)
3.41
F (s)
3.65
H (m)
3.38
K (dd)
1.98
J(8.85, 4.51)
I (dd)
2.30
J(6.85, 4.54)
J (s)
2.25
f1 (ppm)

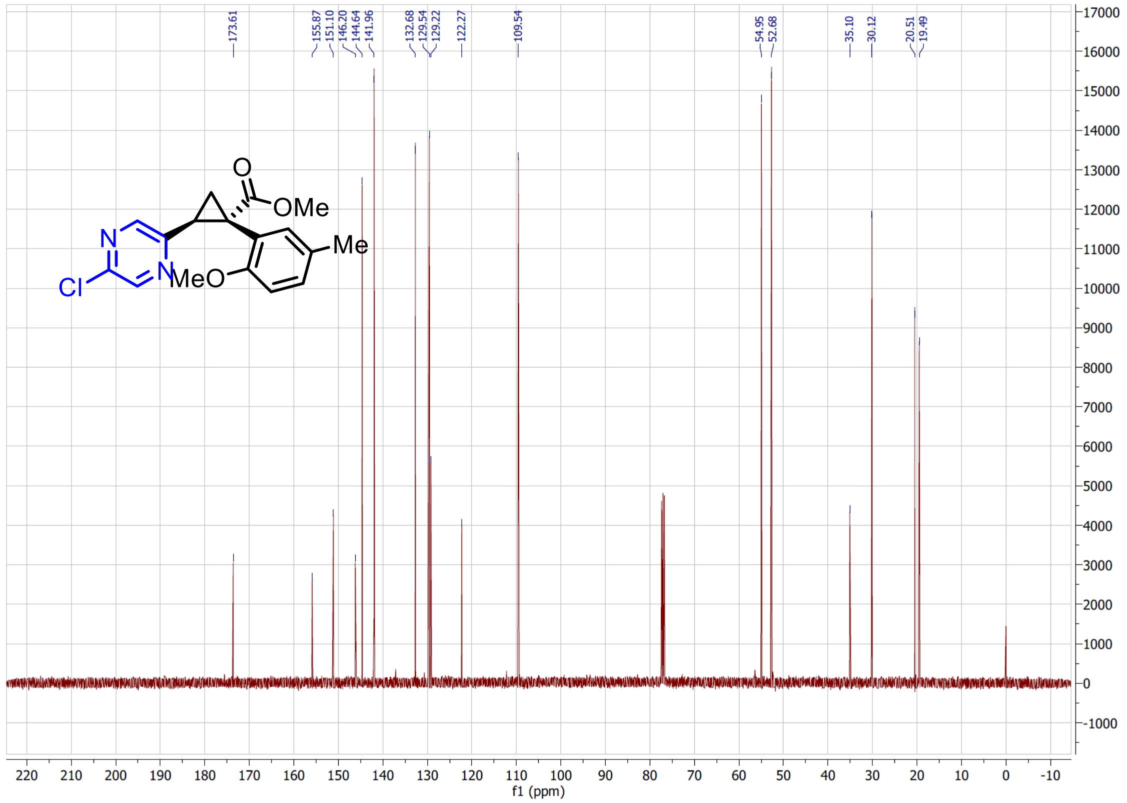

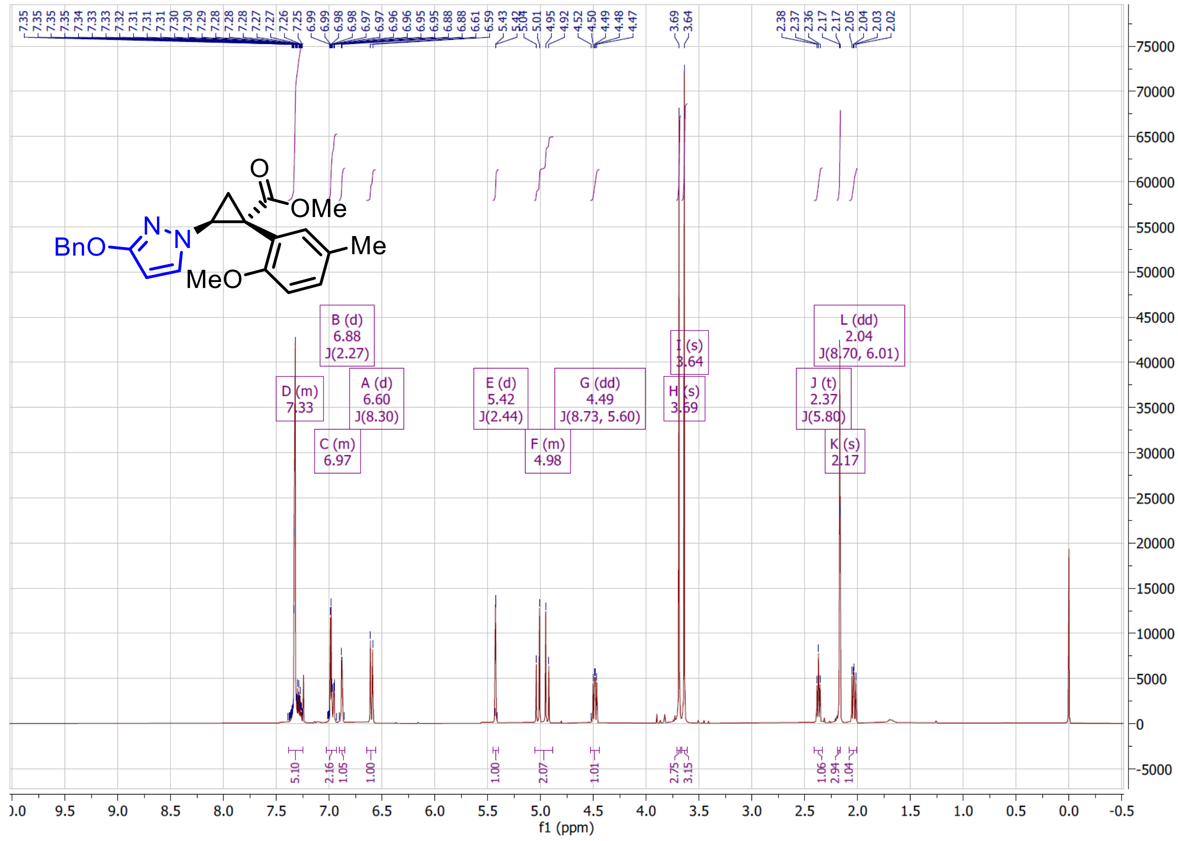

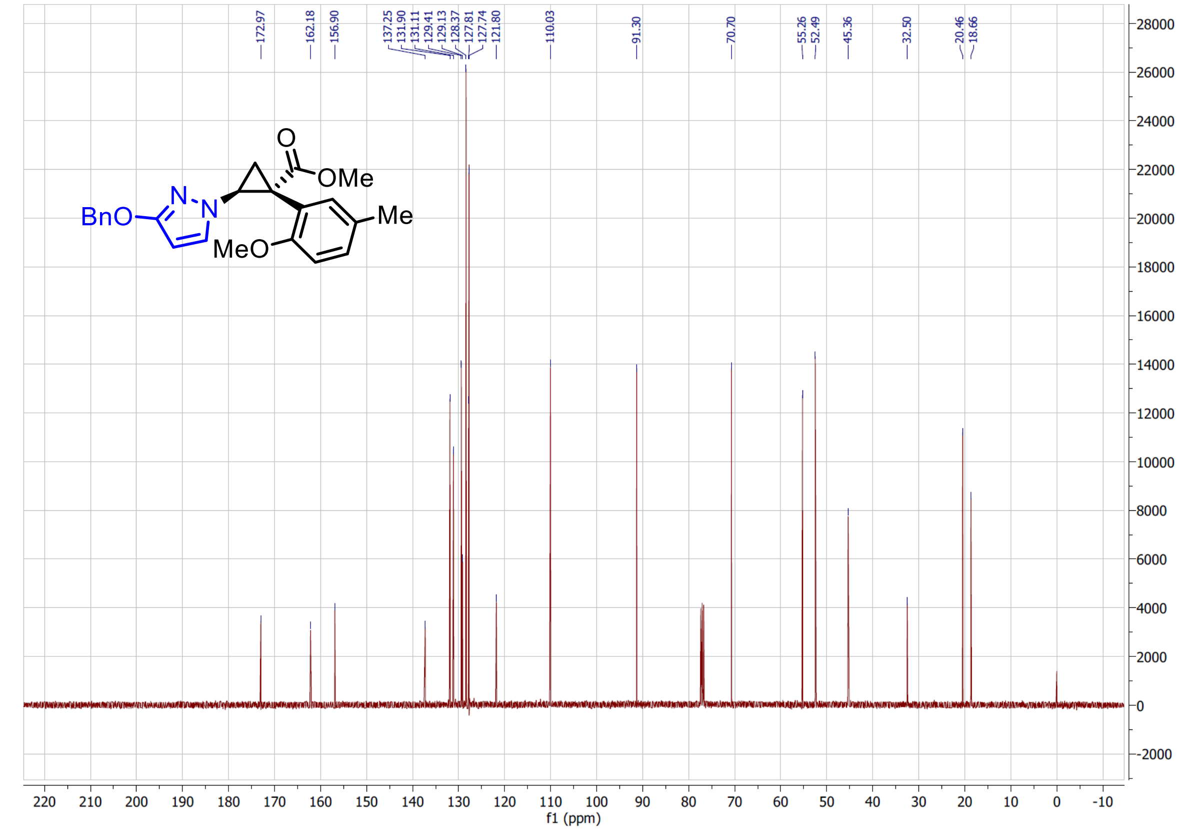
172.97
162.18
156.90
137.25
131.90
131.11
129.41
129.13
128.37
127.81
127.74
121.80
110.03
91.30
70.70
55.26
52.49
45.36
32.50
20.46
18.66
O
OMe
N
N
BnO
Me
MeO
f1 (ppm)

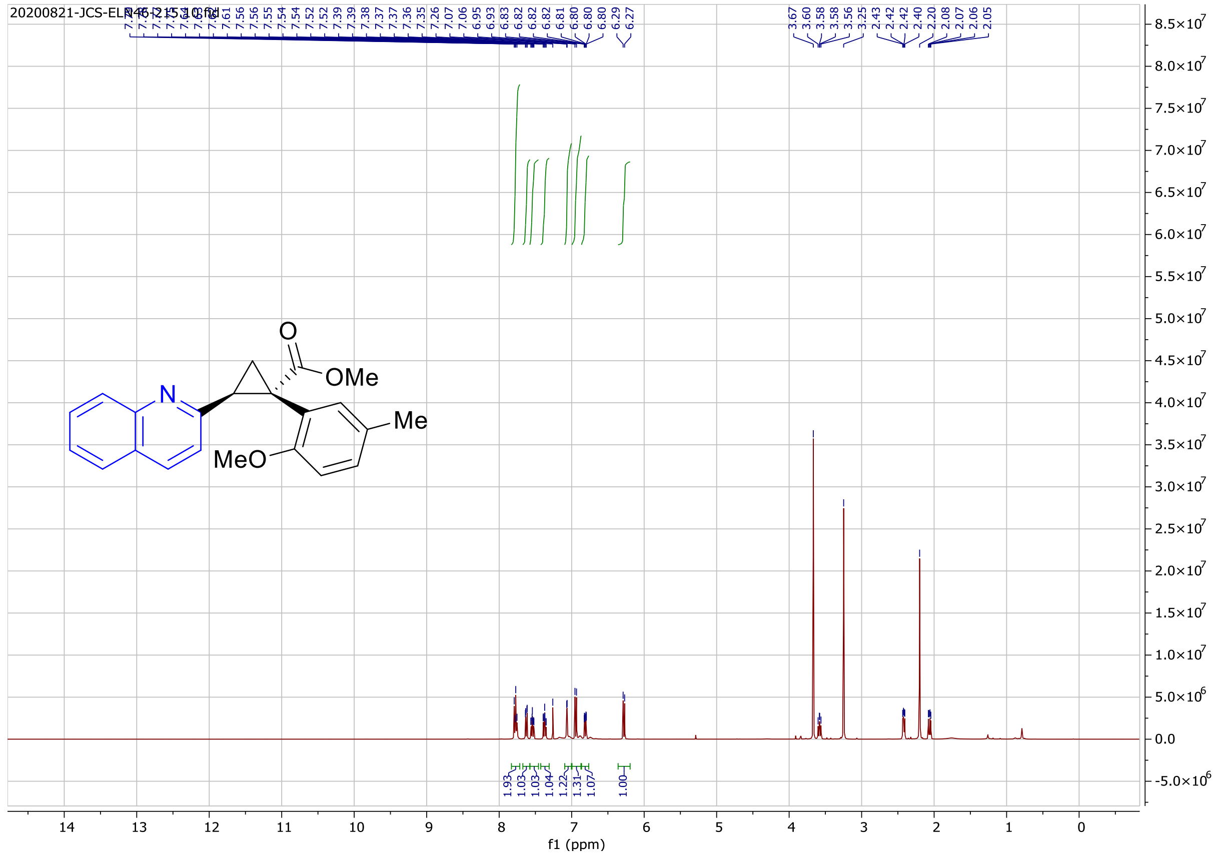
20200821-JCS-ELN46-215
7.61
7.56
7.55
7.54
7.54
7.52
7.52
7.39
7.39
7.38
7.37
7.37
7.36
7.35
7.26
7.07
7.06
6.95
6.93
6.83
6.82
6.82
6.82
6.81
6.80
6.80
6.80
6.29
6.27
3.67
3.60
3.58
3.56
3.25
2.43
2.42
2.40
2.20
2.08
2.07
2.06
2.05
1.93
1.03
1.03
1.04
1.22
1.31
1.07
1.00
O
OMe
N
Me
MeO
f1 (ppm)

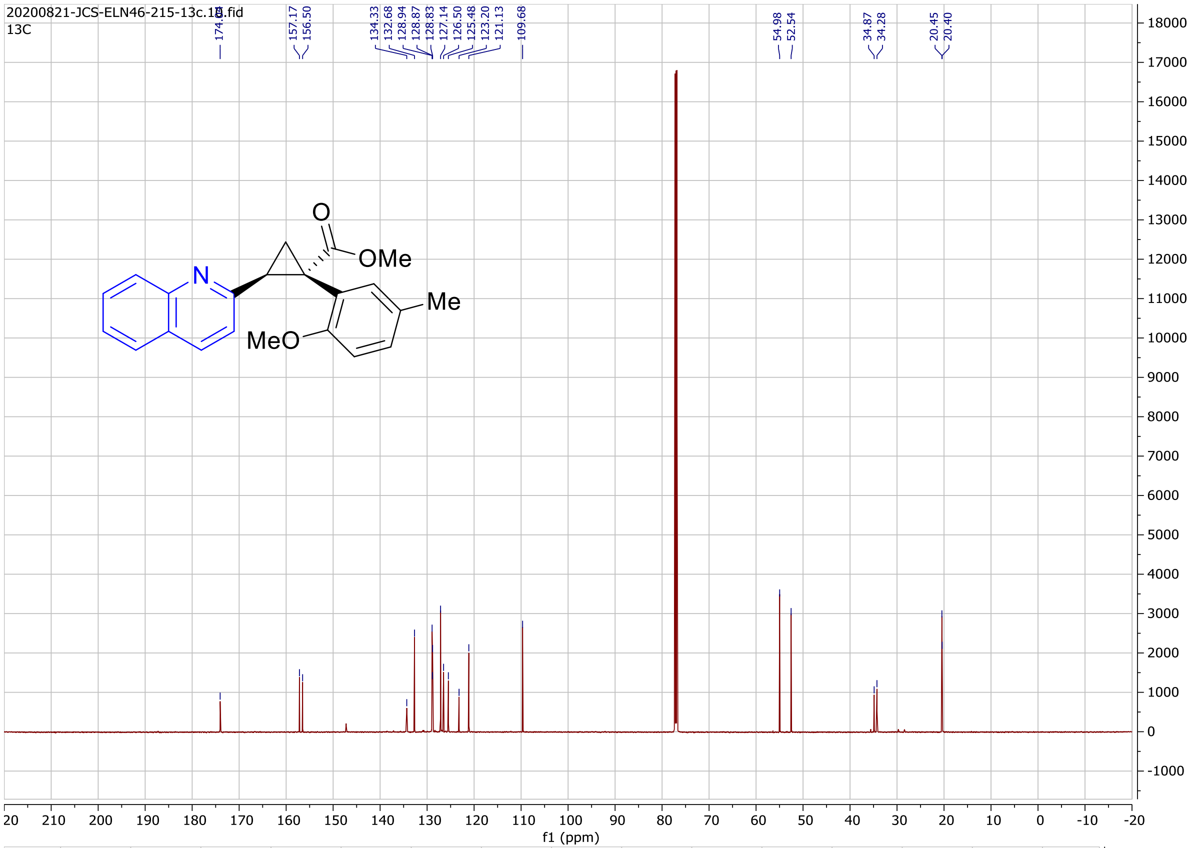
20200821-JCS-ELN46-215-13c.10.fid
13C
174.0
157.17
156.50
134.33
132.68
128.94
128.87
128.83
127.14
126.50
125.48
123.20
121.13
109.68
54.98
52.54
34.87
34.28
20.45
20.40
O
OMe
N
Me
MeO
f1 (ppm)

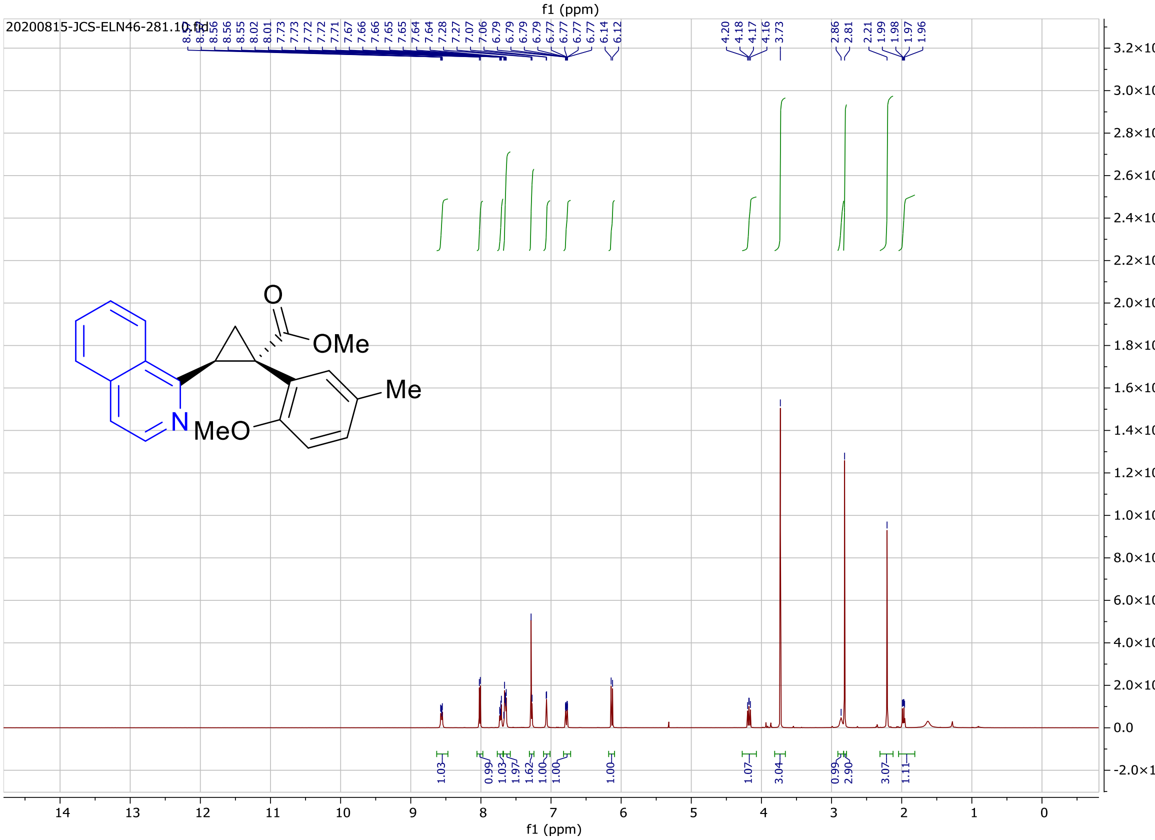
20200815-JCS-ELN46-281.10.fid
4.20
4.18
4.17
4.16
3.73
2.86
2.81
2.21
1.99
1.98
1.97
1.96
O
OMe
N
Me
MeO
f1 (ppm)

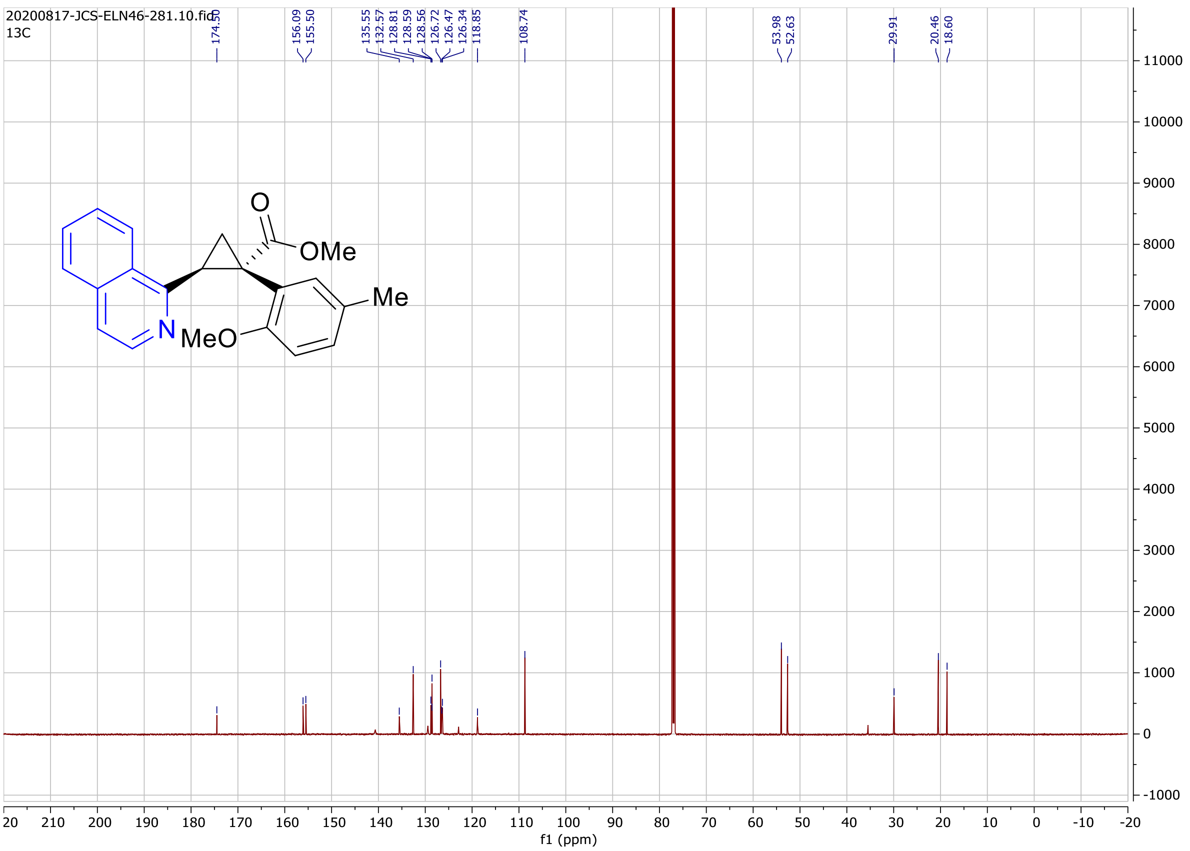
20200817-JCS-ELN46-281.10.fid
13C
174.50
156.09
155.50
135.55
132.57
128.81
128.59
128.56
126.72
126.47
126.34
118.85
108.74
53.98
52.63
29.91
20.46
18.60
O
OMe
Me
N
MeO
f1 (ppm)

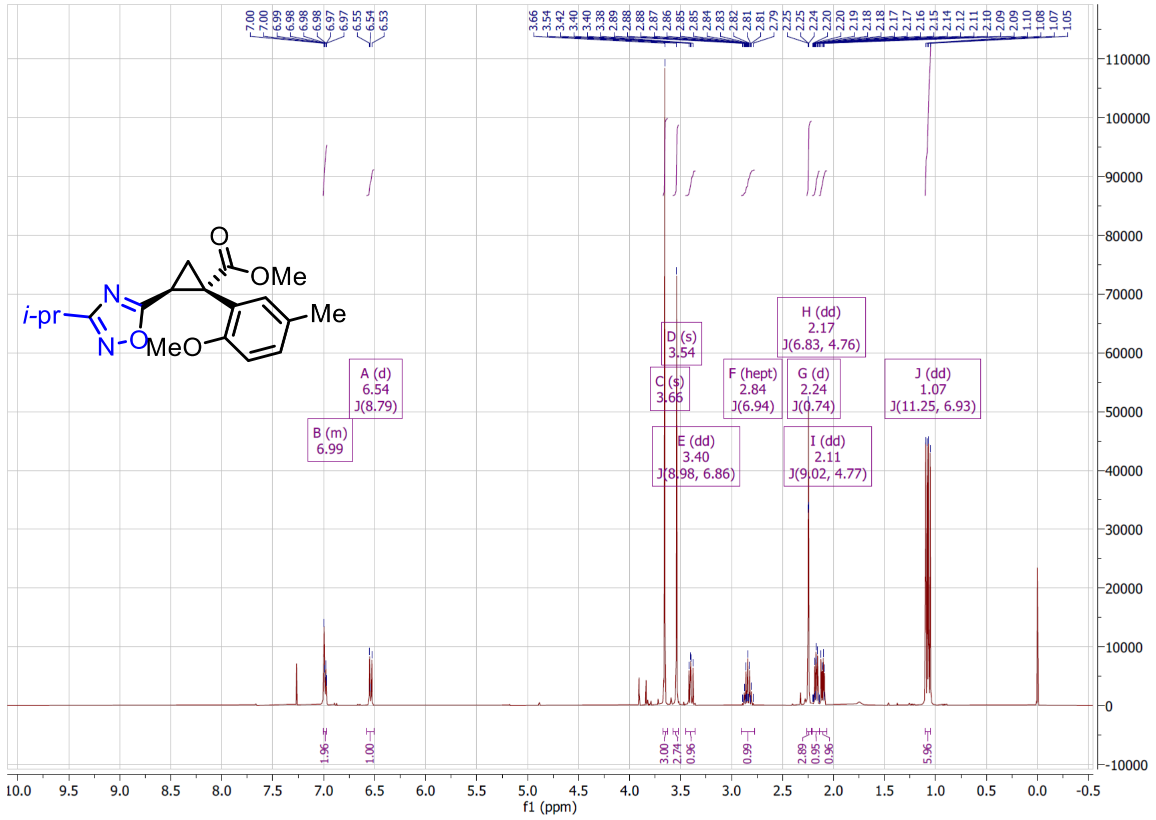
i-pr
N
N-O
MeO
O
OMe
Me
A (d)
6.54
J(8.79)
B (m)
6.99
C (s)
3.66
D (s)
3.54
E (dd)
3.40
J(8.98, 6.86)
F (hept)
2.84
J(6.94)
G (d)
2.24
J(0.74)
H (dd)
2.17
J(6.83, 4.76)
I (dd)
2.11
J(9.02, 4.77)
J (dd)
1.07
J(11.25, 6.93)
f1 (ppm)

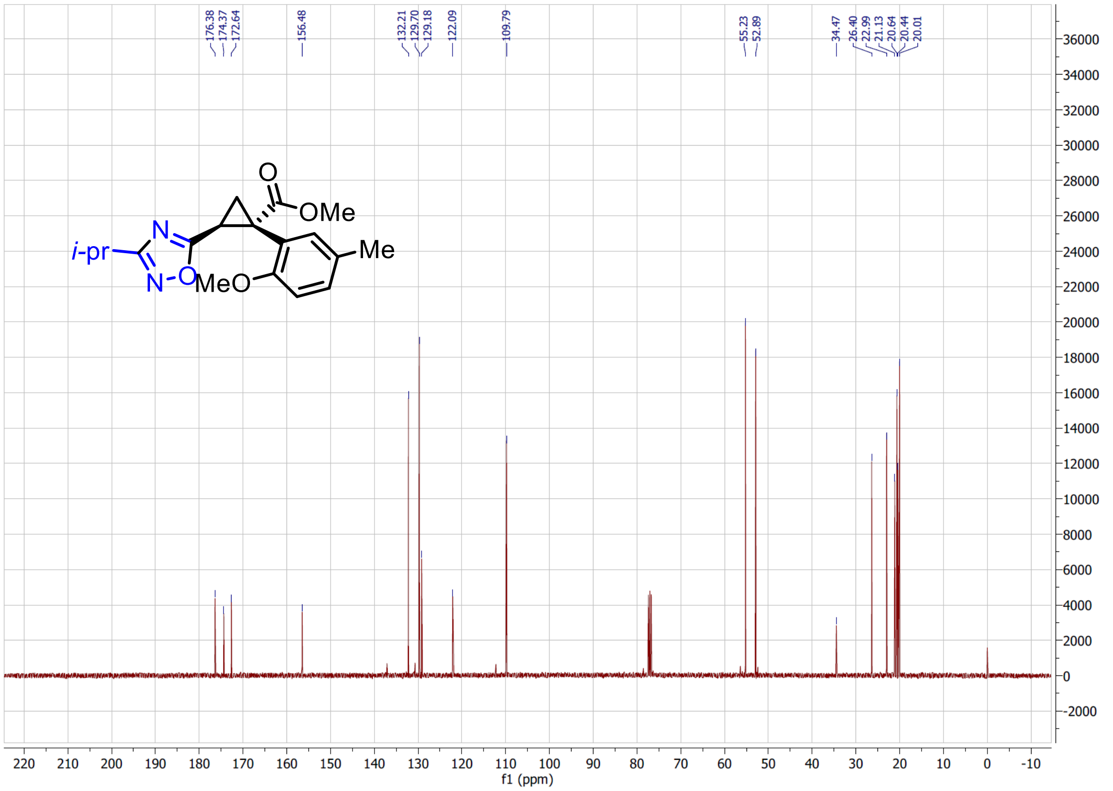
176.38
174.37
172.64
156.48
132.21
129.70
129.18
122.09
109.79
55.23
52.89
34.47
26.40
22.99
21.13
20.64
20.44
20.01
i-pr
N
N-O
O
OMe
Me
MeO
f1 (ppm)

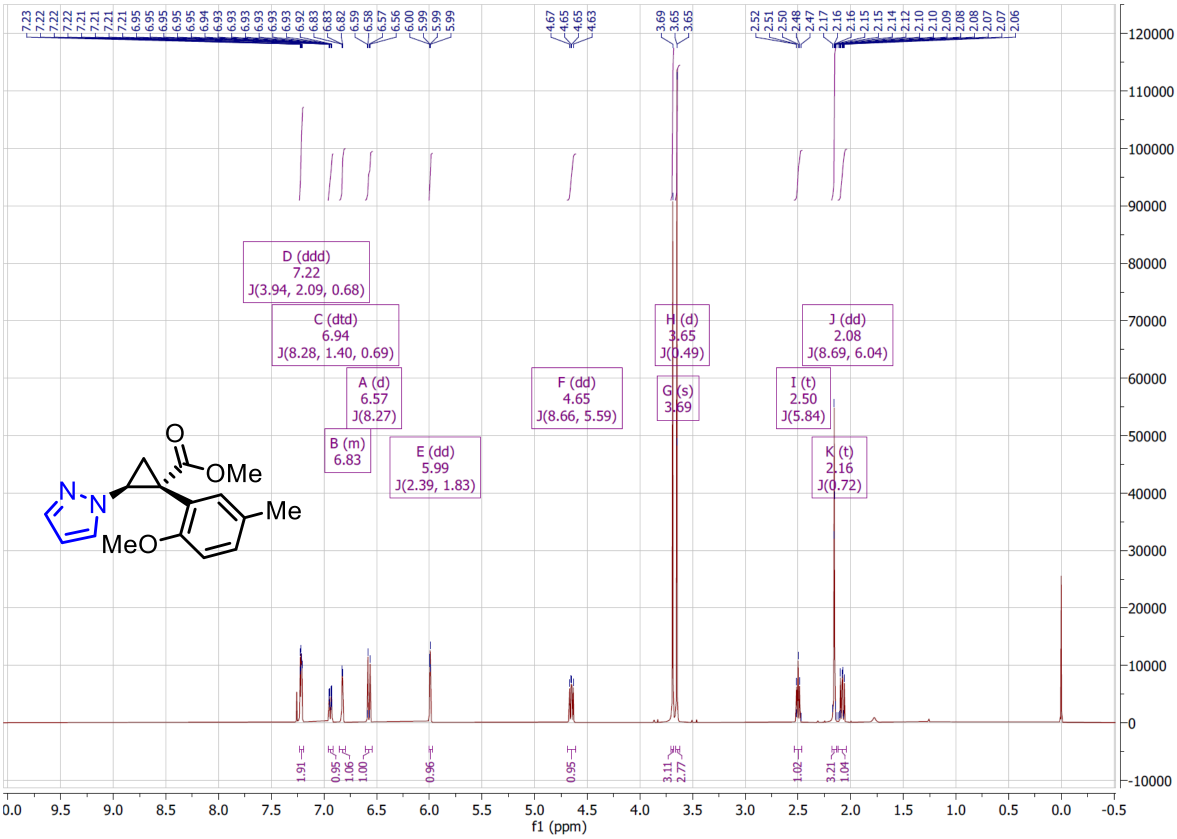
D (ddd)
7.22
J(3.94, 2.09, 0.68)
C (dtd)
6.94
J(8.28, 1.40, 0.69)
A (d)
6.57
J(8.27)
B (m)
6.83
E (dd)
5.99
J(2.39, 1.83)
F (dd)
4.65
J(8.66, 5.59)
H (d)
3.65
J(0.49)
G (s)
3.69
I (t)
2.50
J(5.84)
J (dd)
2.08
J(8.69, 6.04)
K (t)
2.16
J(0.72)
1.91
0.95
1.06
1.00
0.96
0.95
3.11
2.77
1.02
3.21
1.04
N
N
O
OMe
Me
MeO
f1 (ppm)

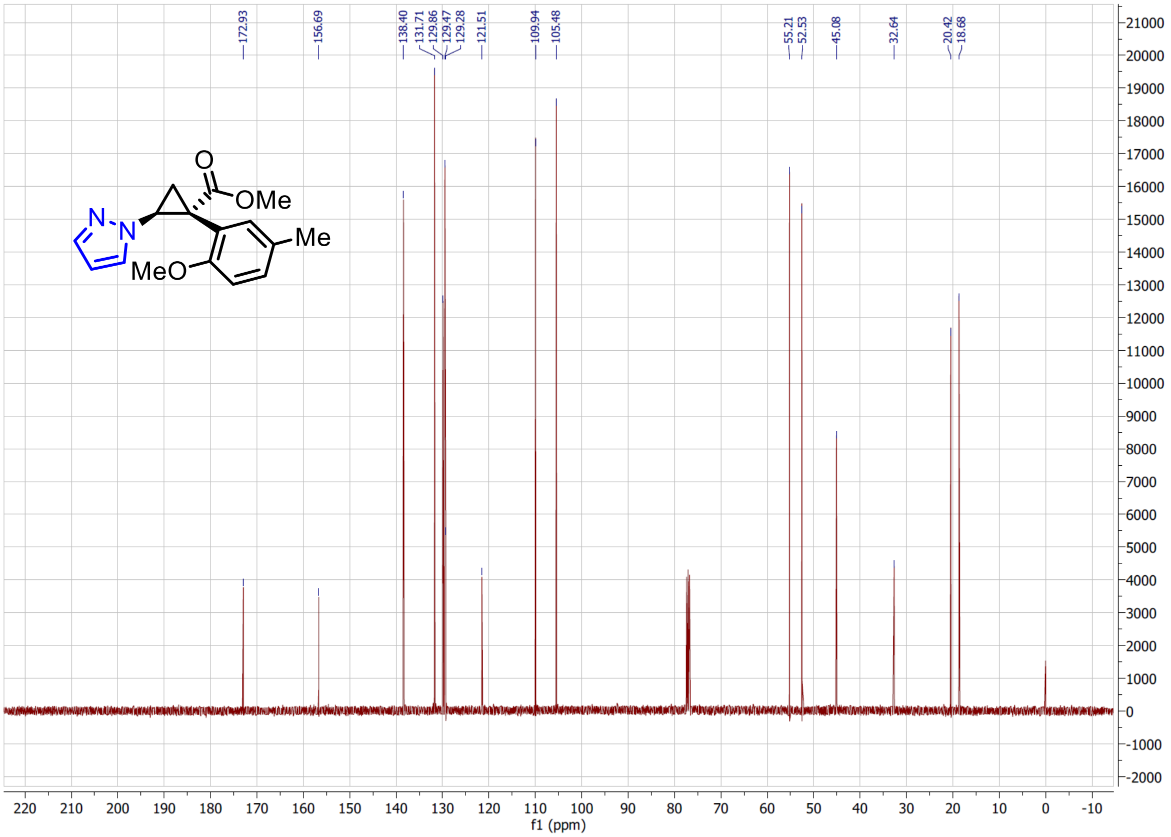
172.93
156.69
138.40
131.71
129.86
129.47
129.28
121.51
109.94
105.48
55.21
52.53
45.08
32.64
20.42
18.68
O
OMe
N
N
Me
MeO
f1 (ppm)

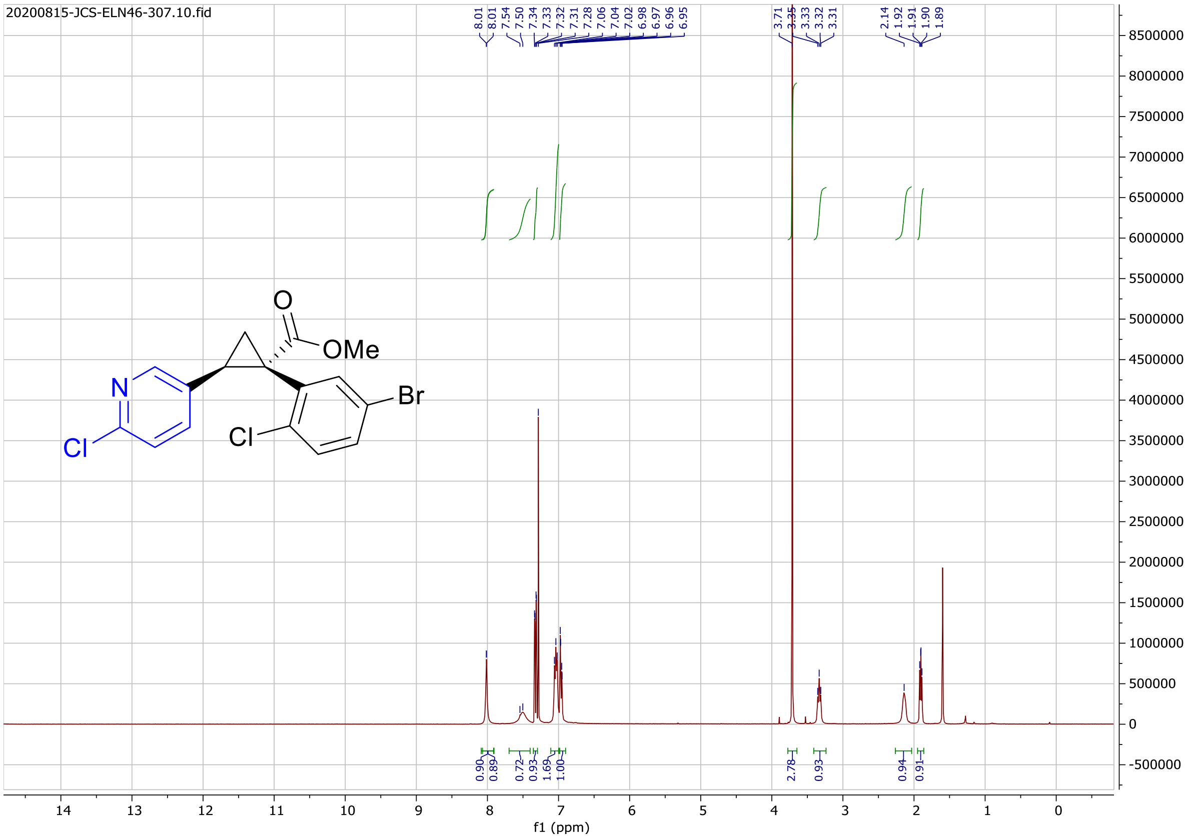
20200815-JCS-ELN46-307.10.fid
8.01
8.01
7.54
7.50
7.34
7.33
7.32
7.31
7.28
7.06
7.04
7.02
6.98
6.97
6.96
6.95
3.71
3.35
3.33
3.32
3.31
2.14
1.92
1.91
1.90
1.89
O
OMe
N
Cl
Br
Cl
0.90
0.89
0.72
0.93
1.69
1.00
2.78
0.93
0.94
0.91
f1 (ppm)

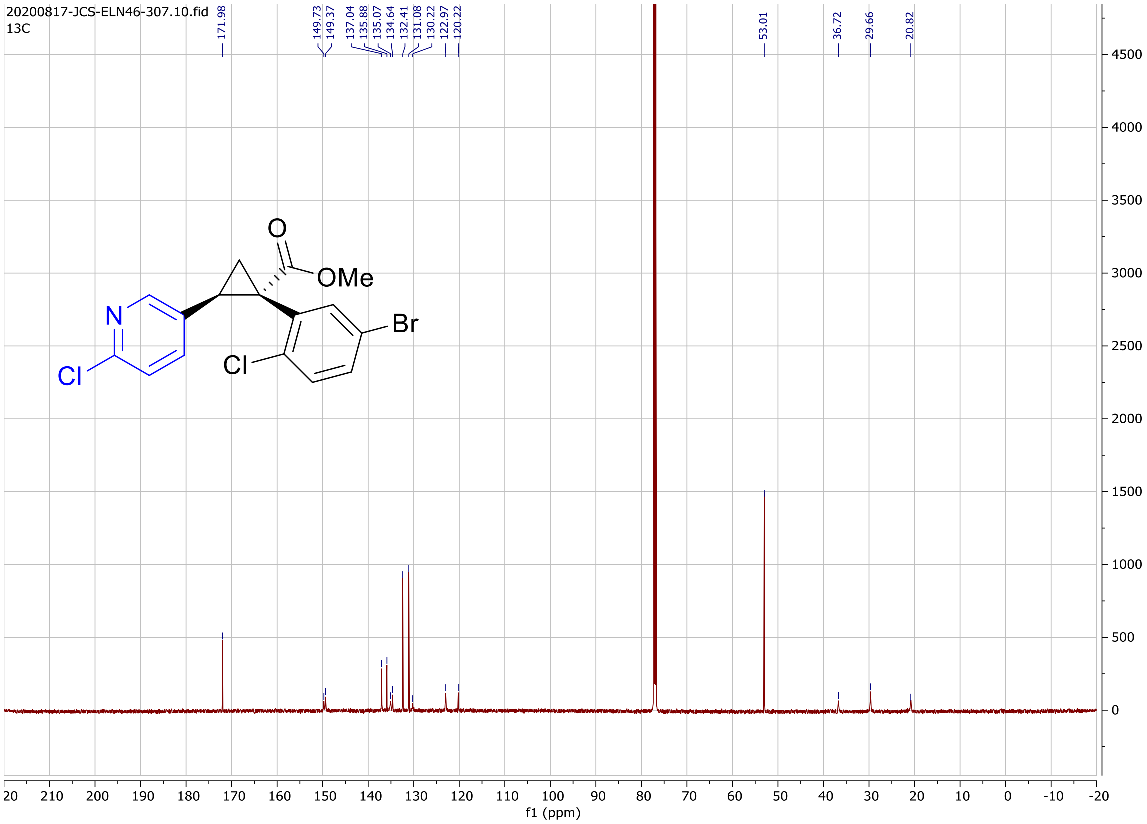

20200817-JCS-ELN46-307.10.fid
13C
171.98
149.73
149.37
137.04
135.88
135.07
134.64
132.41
131.08
130.22
122.97
120.22
53.01
36.72
29.66
20.82
O
OMe
N
Br
Cl
Cl
f1 (ppm)

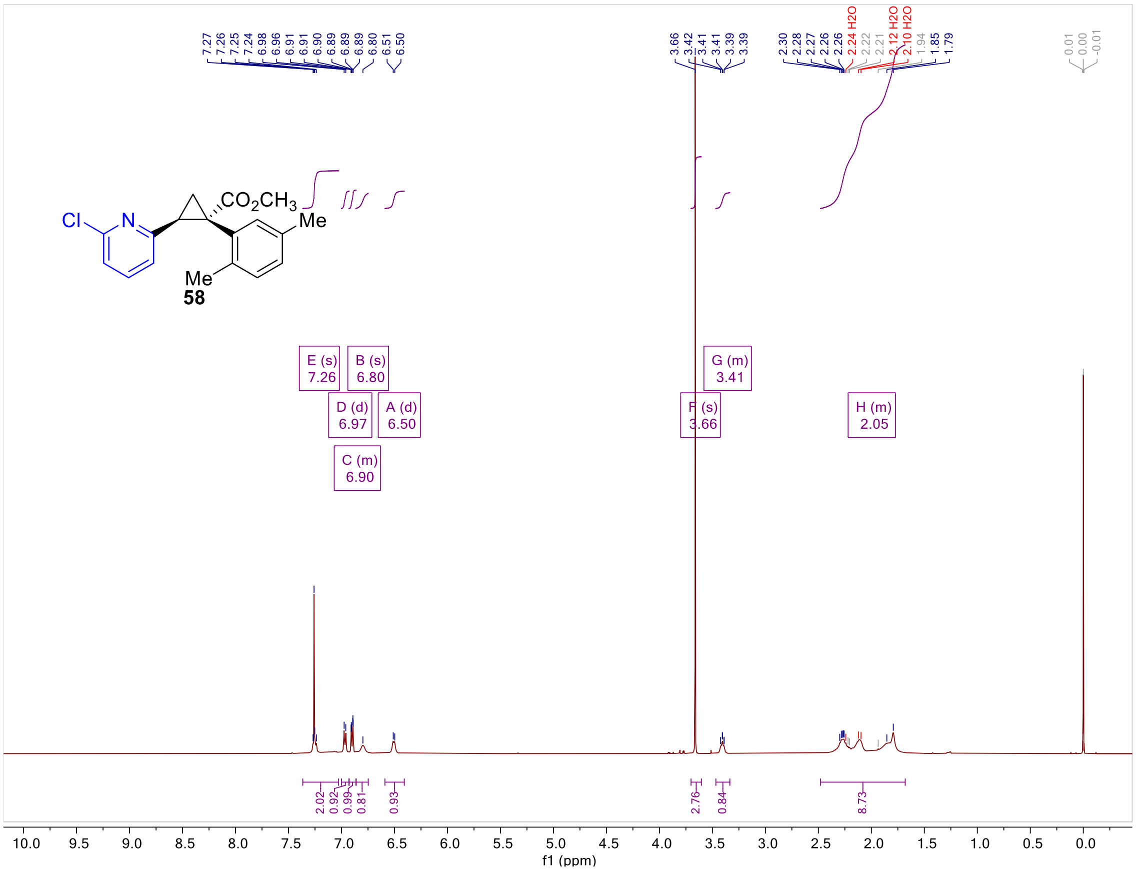

7.27
7.26
7.25
7.24
6.98
6.96
6.91
6.91
6.90
6.89
6.89
6.89
6.80
6.51
6.50
3.66
3.42
3.41
3.41
3.39
3.39
2.30
2.28
2.27
2.26
2.26
2.24 H2O
2.22
2.21
2.12 H2O
2.10 H2O
1.94
1.85
1.79
0.01
0.00
-0.01
Cl
N
CO_2CH_3
Me
Me
58
E (s)
7.26
B (s)
6.80
D (d)
6.97
A (d)
6.50
C (m)
6.90
G (m)
3.41
F (s)
3.66
H (m)
2.05
2.02
0.92
0.99
0.81
0.93
2.76
0.84
8.73
f1 (ppm)

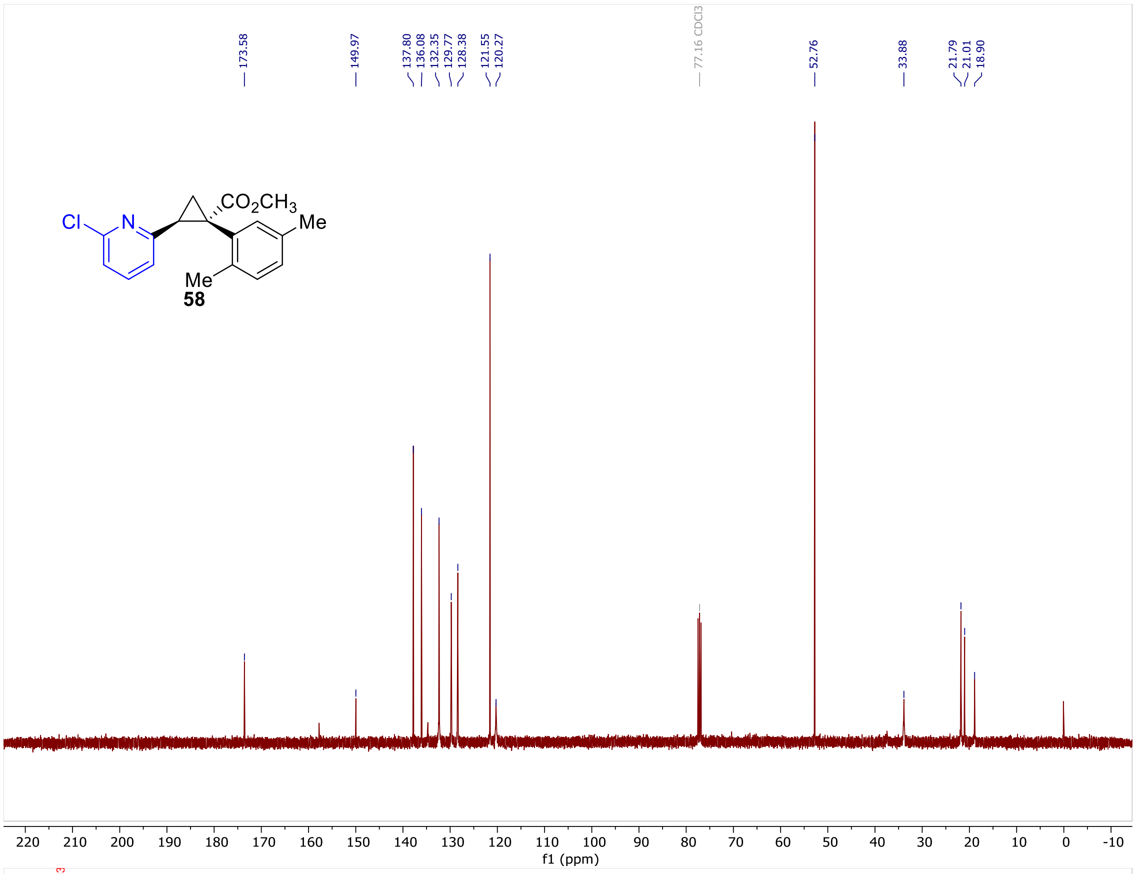

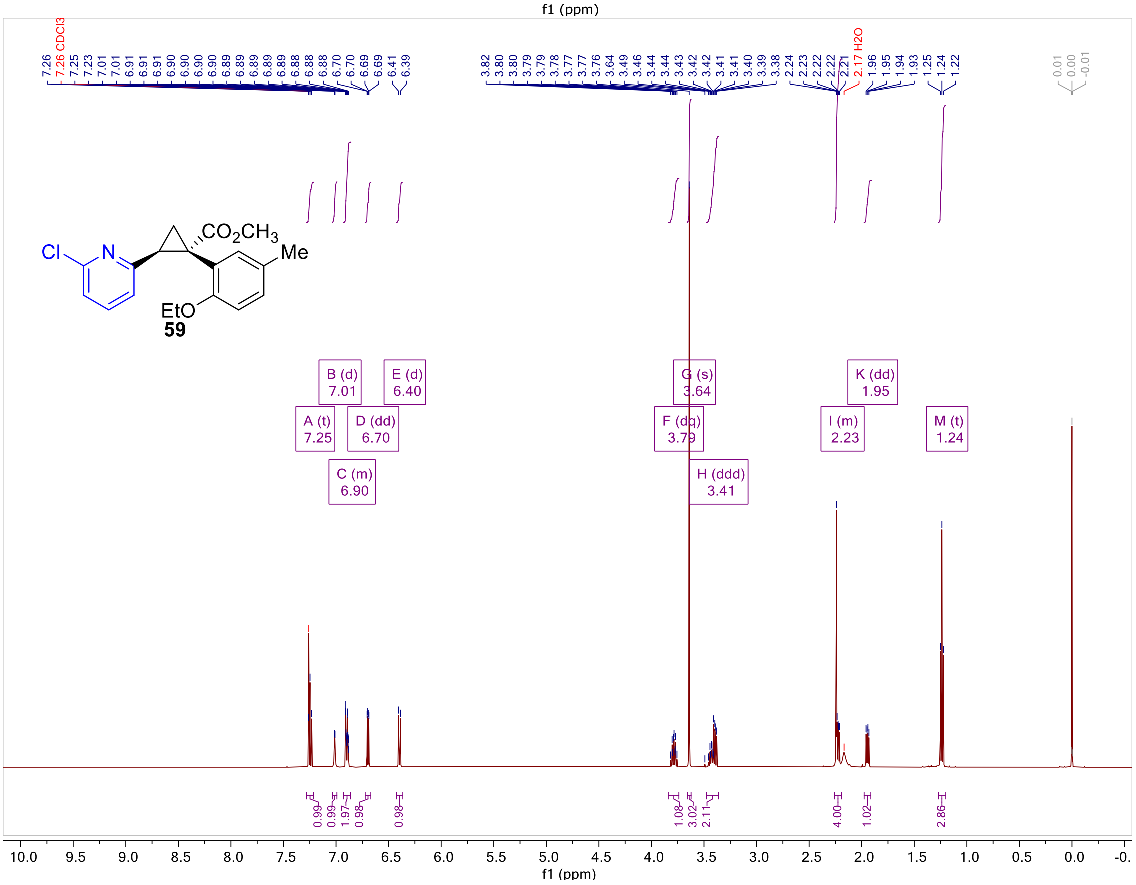

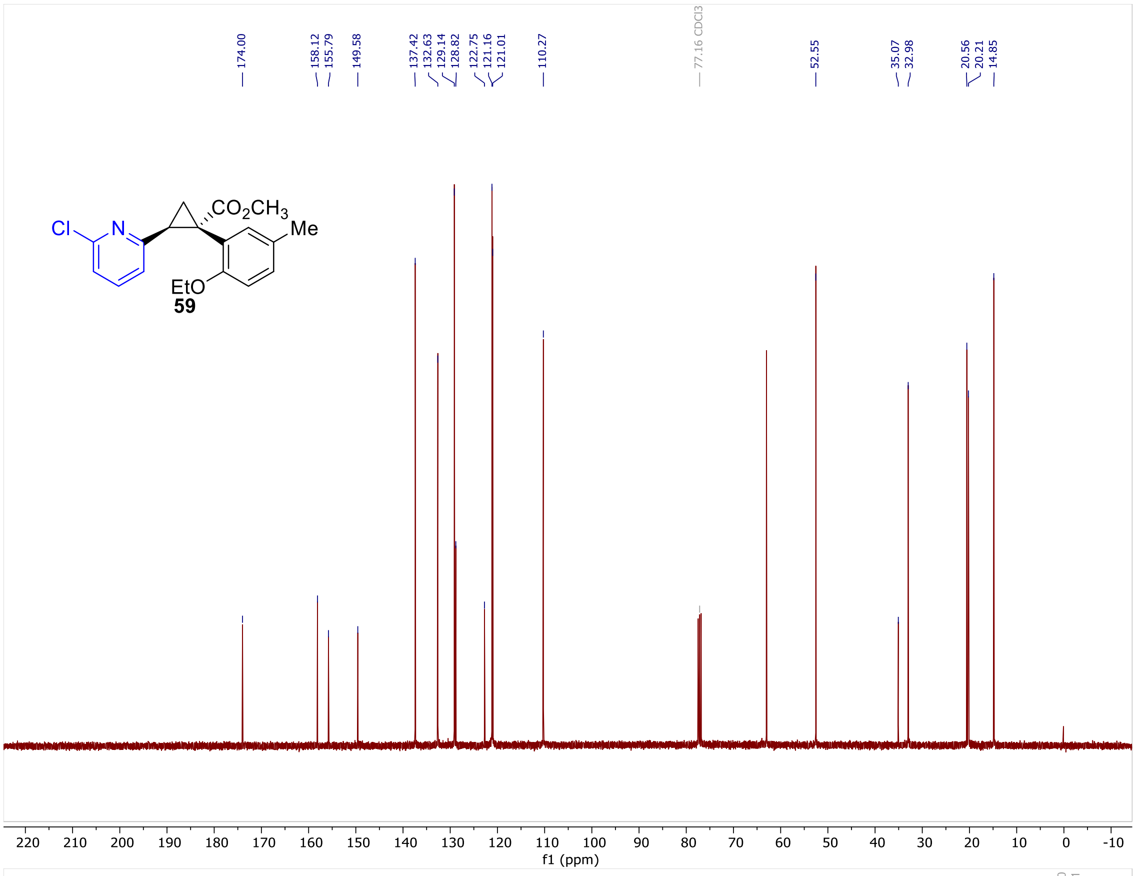
174.00
158.12
155.79
149.58
137.42
132.63
129.14
128.82
122.75
121.16
121.01
110.27
77.16 CDCl3
52.55
35.07
32.98
20.56
20.21
14.85
CO2CH3
Cl
N
Me
EtO
59
220 210 200 190 180 170 160 150 140 130 120 110 100 90 80 70 60 50 40 30 20 10 0 -10
f1 (ppm)

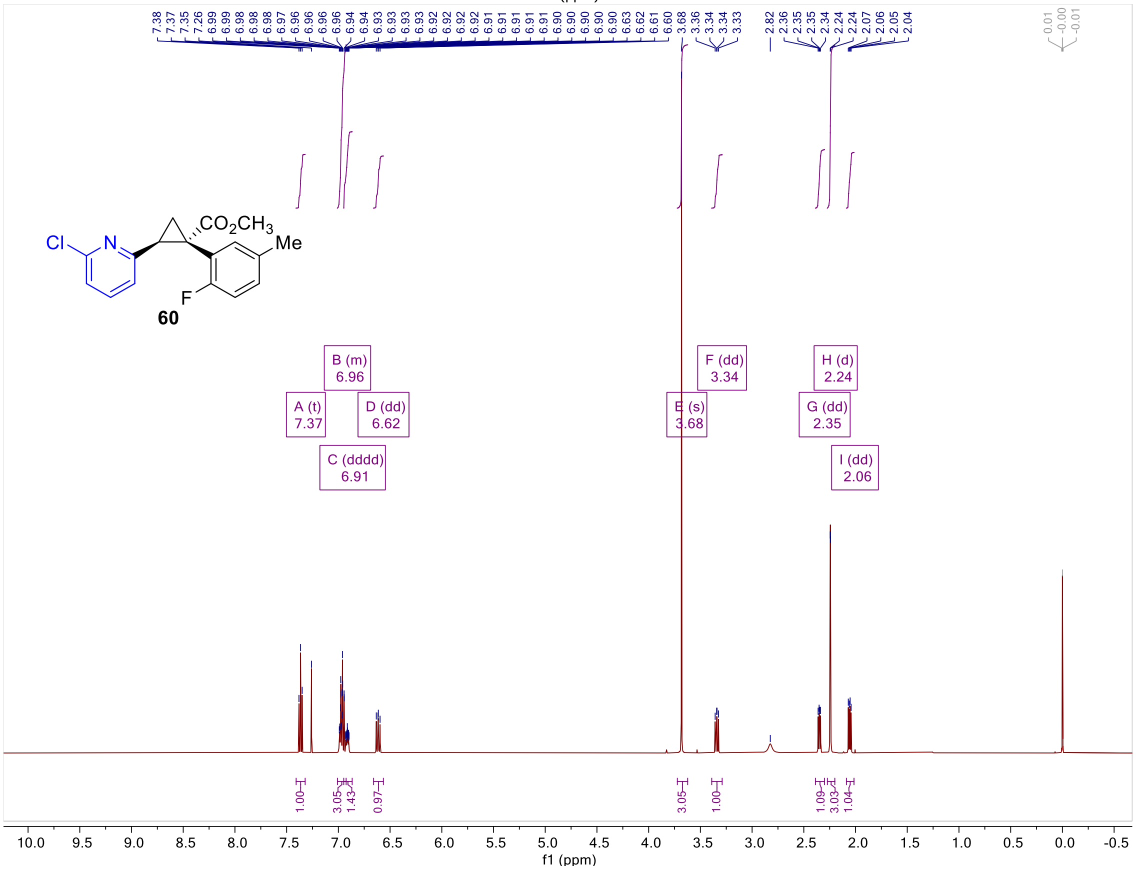
7.38
7.37
7.35
7.26
6.99
6.99
6.98
6.98
6.98
6.97
6.96
6.96
6.96
6.96
6.94
6.94
6.93
6.93
6.93
6.93
6.92
6.92
6.92
6.92
6.91
6.91
6.91
6.91
6.90
6.90
6.90
6.90
6.63
6.62
6.61
6.60
3.68
3.36
3.34
3.34
3.33
2.82
2.36
2.35
2.35
2.34
2.24
2.24
2.07
2.06
2.05
2.04
0.01
-0.00
-0.01
CO2CH3
Cl
N
Me
F
60
B (m)
6.96
A (t)
7.37
D (dd)
6.62
C (dddd)
6.91
E (s)
3.68
F (dd)
3.34
H (d)
2.24
G (dd)
2.35
I (dd)
2.06
1.00
3.05
1.43
0.97
3.05
1.00
1.09
3.03
1.04
10.0 9.5 9.0 8.5 8.0 7.5 7.0 6.5 6.0 5.5 5.0 4.5 4.0 3.5 3.0 2.5 2.0 1.5 1.0 0.5 0.0 -0.5
f1 (ppm)

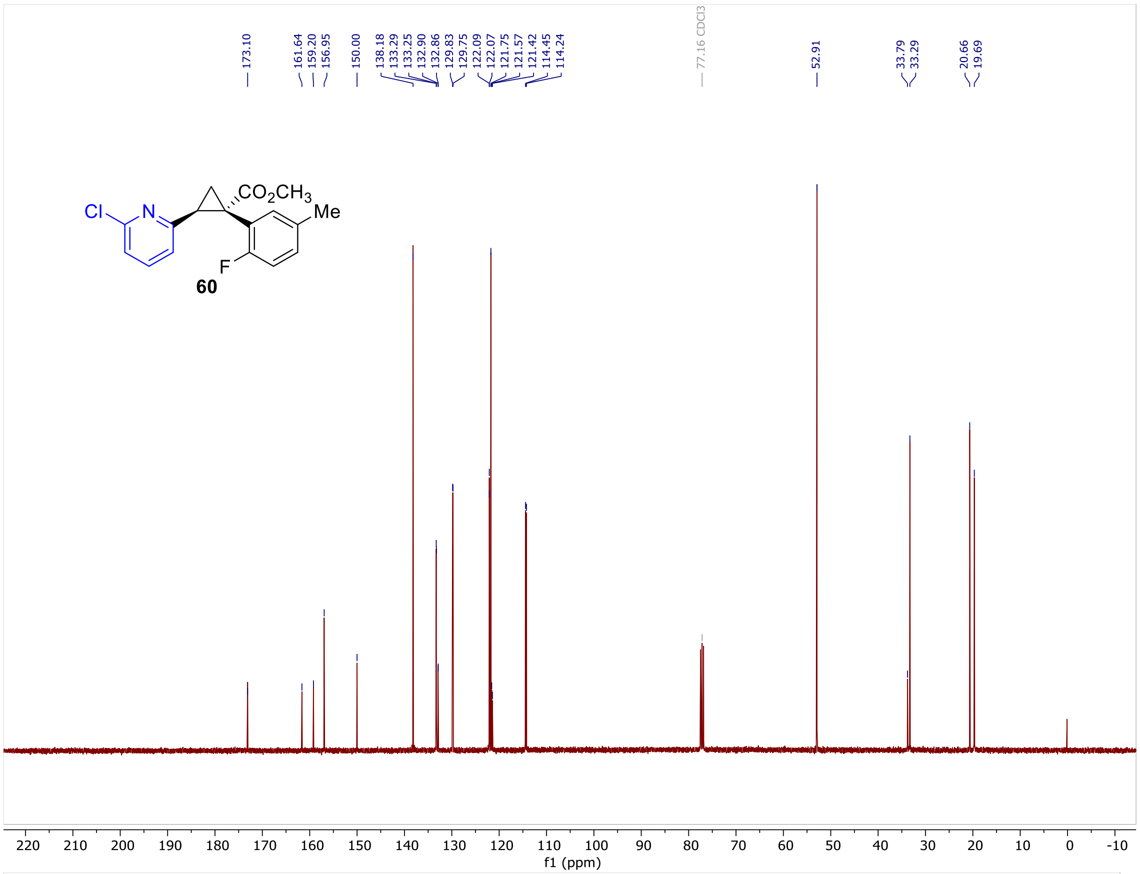

173.10
161.64
159.20
156.95
150.00
138.18
133.29
133.25
132.90
132.86
129.83
129.75
122.09
122.07
121.75
121.57
121.42
114.45
114.24
77.16 CDCl3
52.91
33.79
33.29
20.66
19.69
Cl
N
CO_2CH_3
Me
F
60
220 210 200 190 180 170 160 150 140 130 120 110 100 90 80 70 60 50 40 30 20 10 0 -10
f1 (ppm)

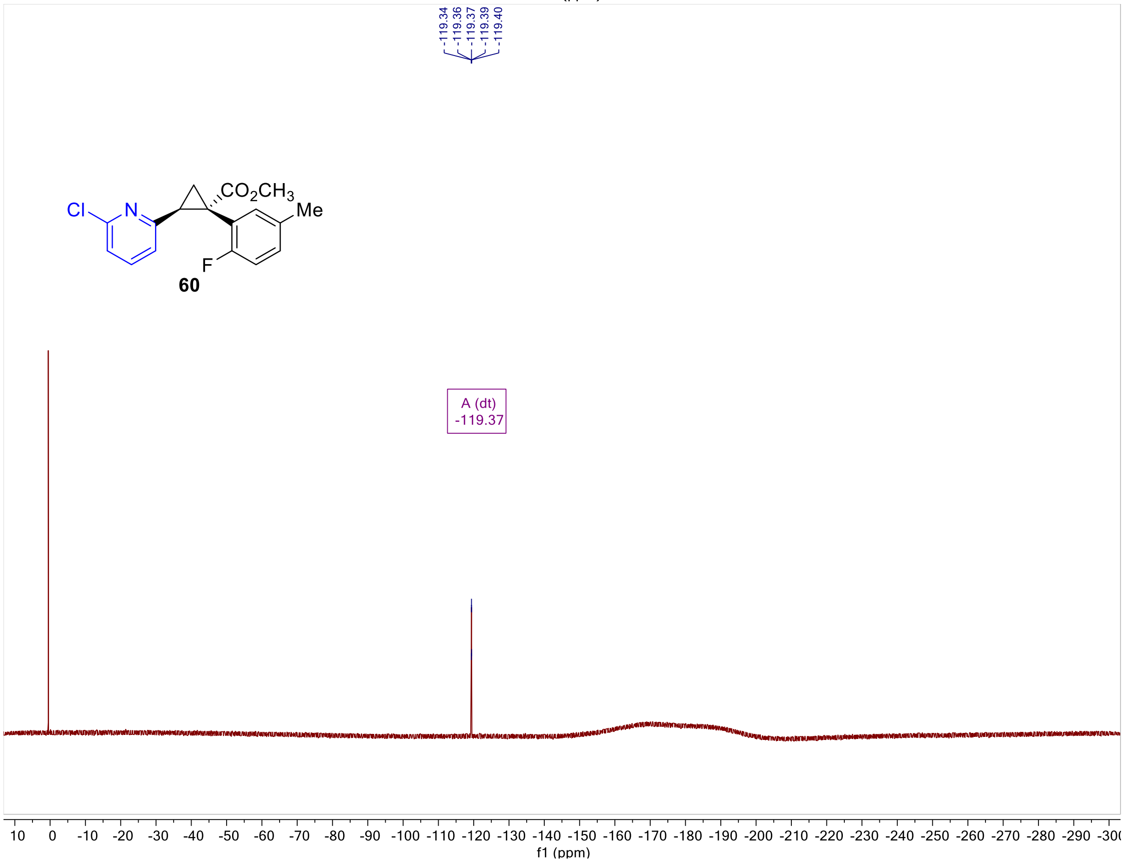

-119.34
-119.36
-119.37
-119.39
-119.40
Cl
N
CO_2CH_3
Me
F
60
A (dt)
-119.37
10 0 -10 -20 -30 -40 -50 -60 -70 -80 -90 -100 -110 -120 -130 -140 -150 -160 -170 -180 -190 -200 -210 -220 -230 -240 -250 -260 -270 -280 -290 -300
f1 (ppm)

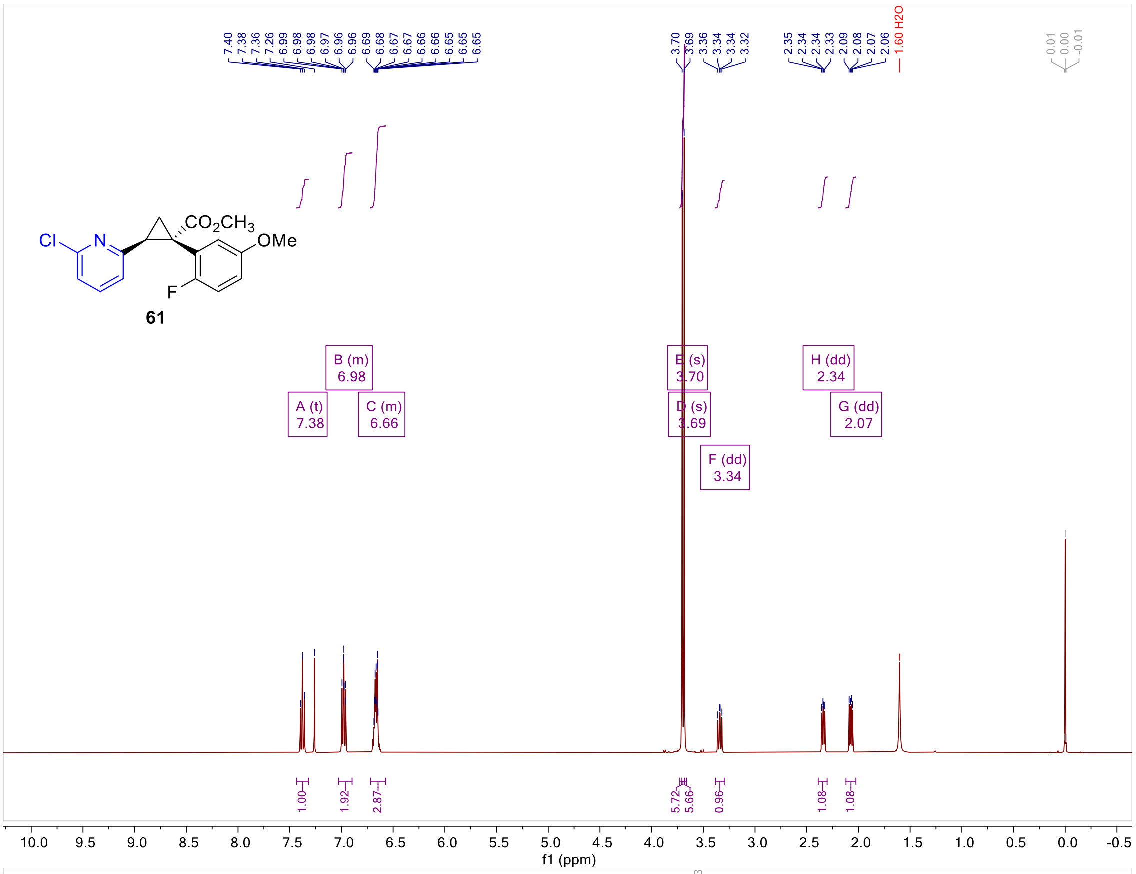

61
f1 (ppm)
A (t) 7.38
B (m) 6.98
C (m) 6.66
D (s) 3.69
E (s) 3.70
F (dd) 3.34
G (dd) 2.07
H (dd) 2.34
1.60 H2O

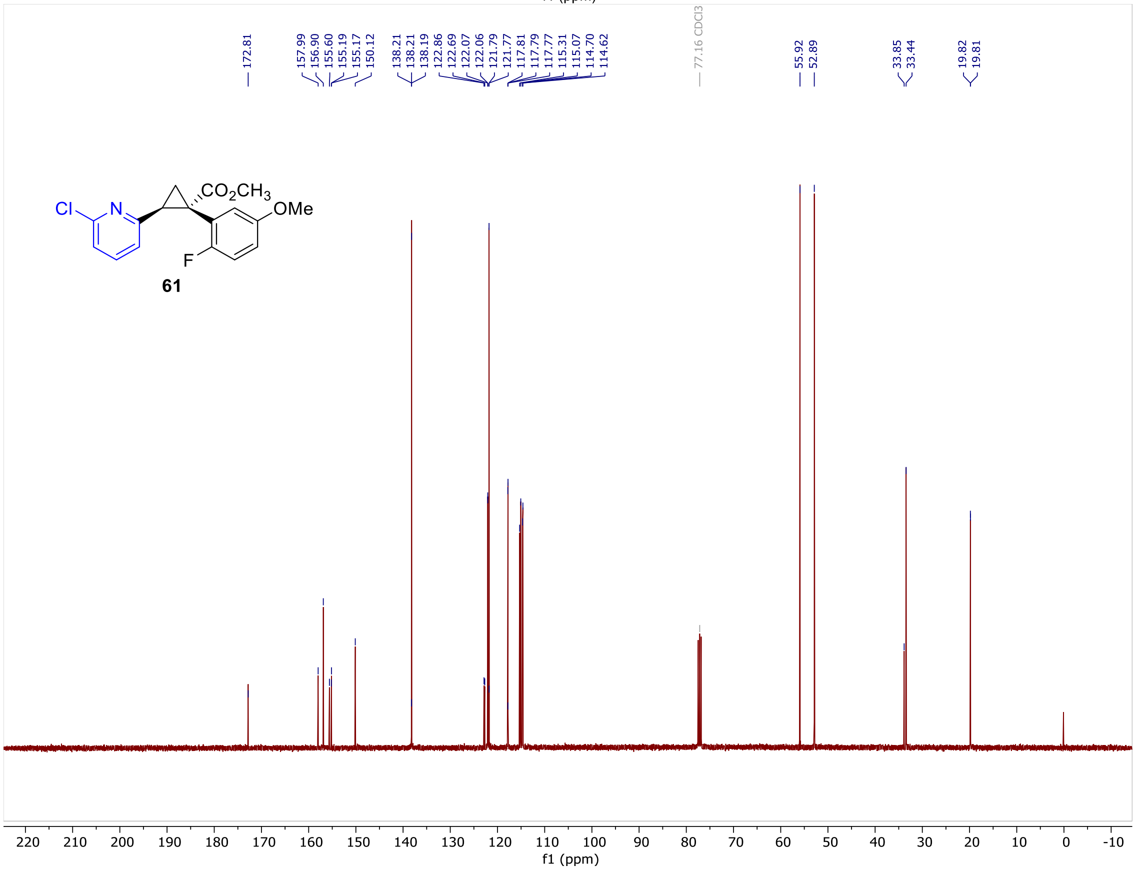

61
f1 (ppm)
77.16 CDCl3

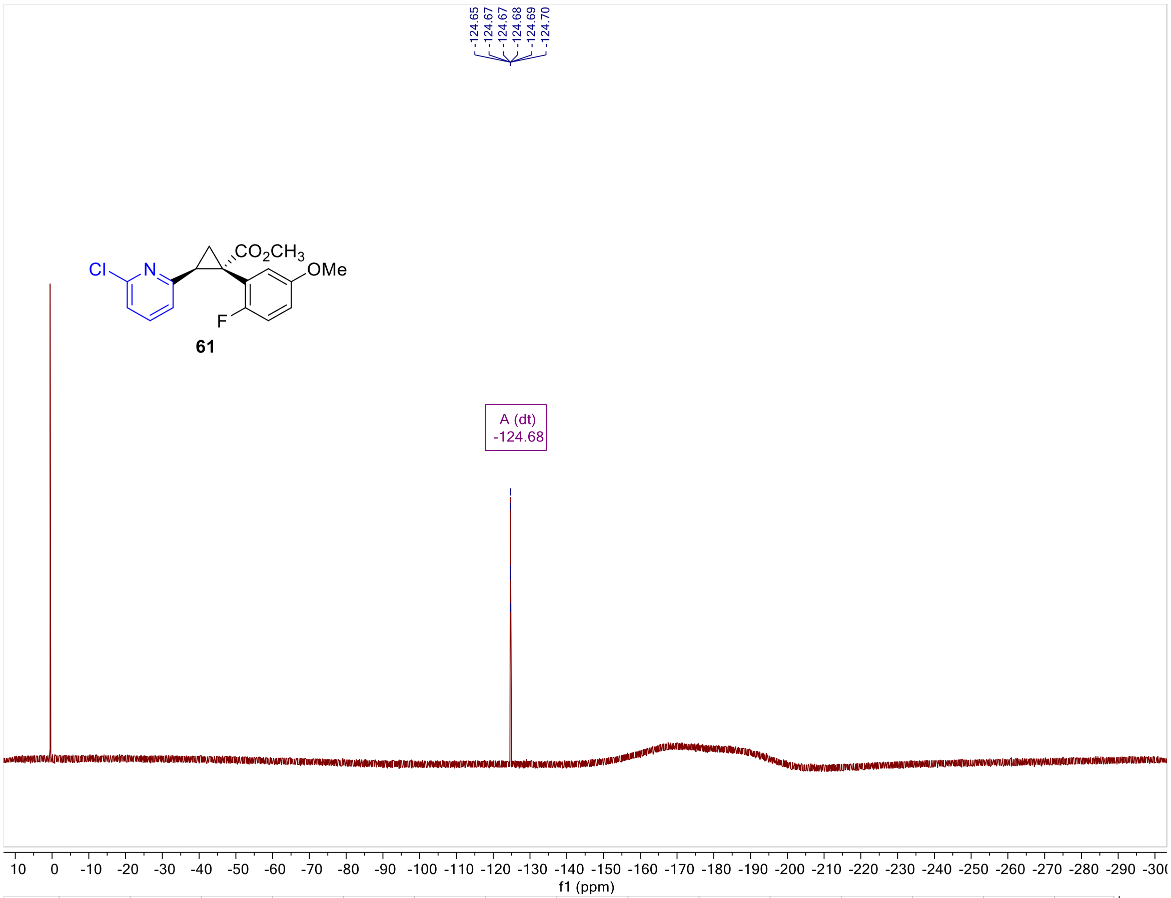
-124.65
-124.67
-124.67
-124.68
-124.69
-124.70
Cl
N
CO_2CH_3
OMe
F
61
A (dt)
-124.68
f1 (ppm)

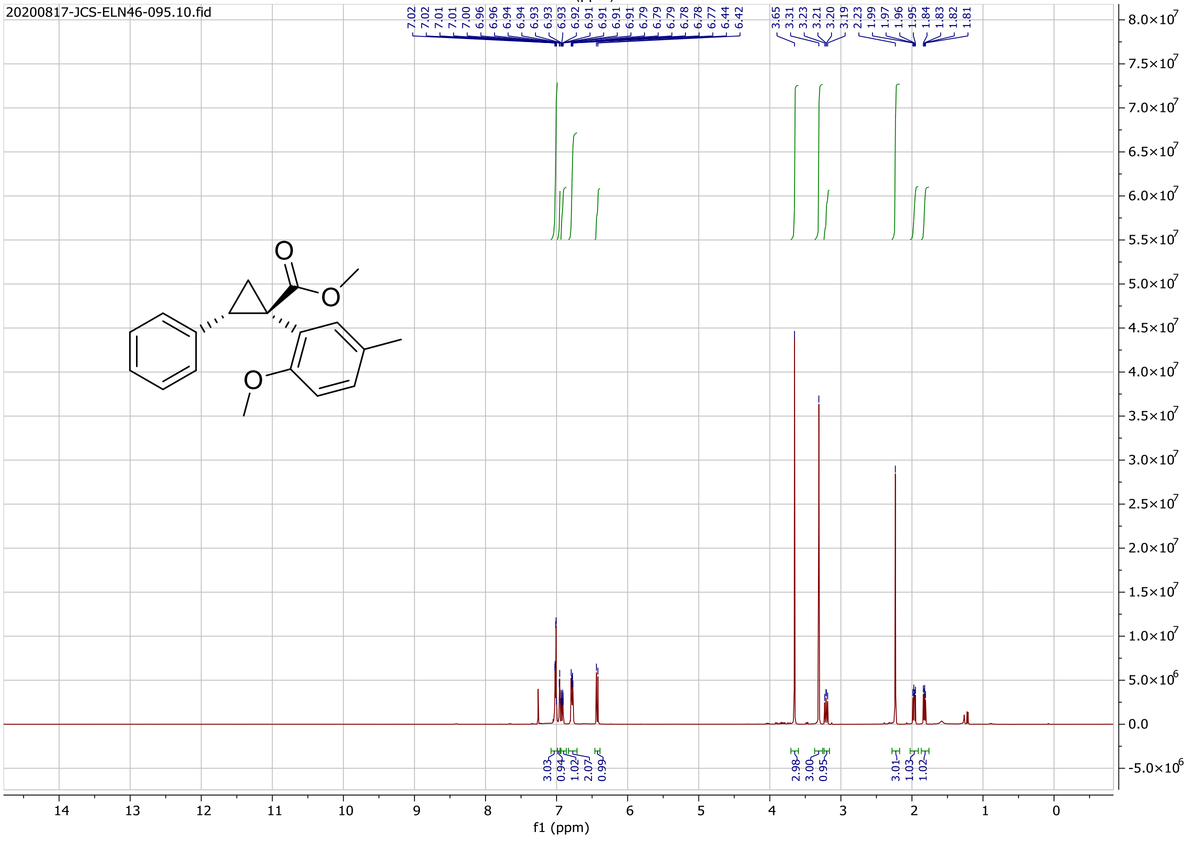
20200817-JCS-ELN46-095.10.fid
f1 (ppm)

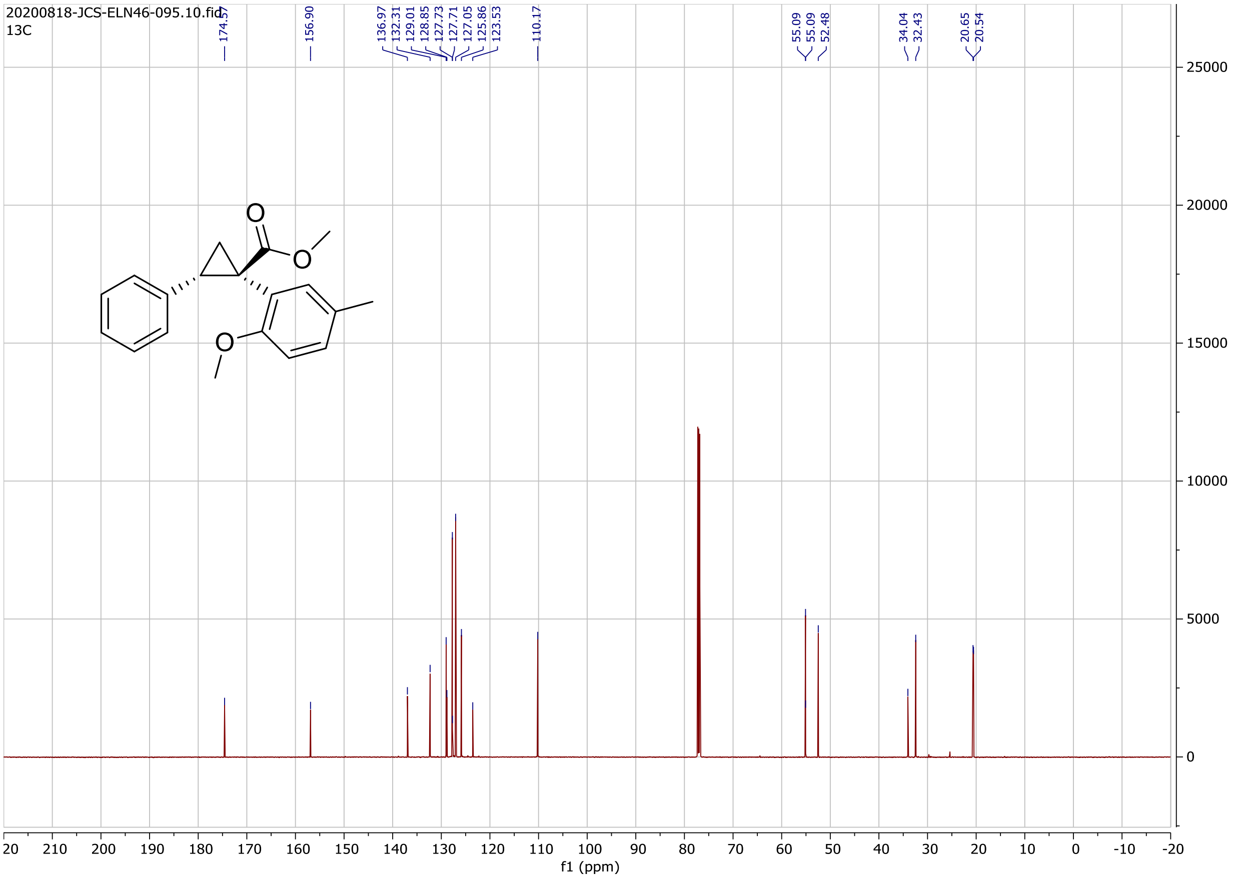
20200818-JCS-ELN46-095.10.fid
13C
174.57
156.90
136.97
132.31
129.01
128.85
127.73
127.71
127.05
125.86
123.53
110.17
55.09
55.09
52.48
34.04
32.43
20.65
20.54
O
O
O
f1 (ppm)

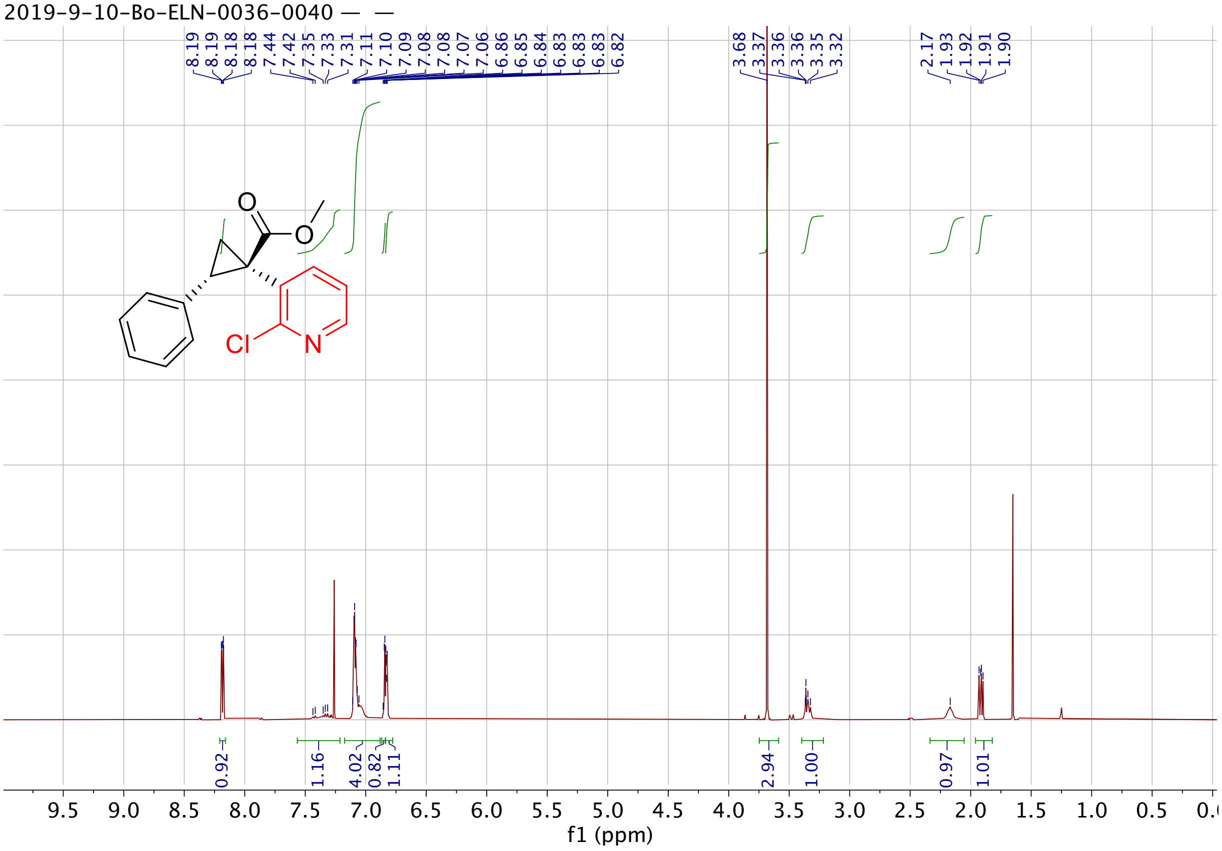
2019-9-10-Bo-ELN-0036-0040
8.19
8.19
8.18
8.18
7.44
7.42
7.35
7.33
7.31
7.11
7.10
7.09
7.08
7.08
7.07
7.06
6.86
6.85
6.84
6.83
6.83
6.83
6.82
3.68
3.37
3.36
3.36
3.35
3.32
2.17
1.93
1.92
1.91
1.90
O
O
Cl
N
0.92
1.16
4.02
0.82
1.11
2.94
1.00
0.97
1.01
f1 (ppm)

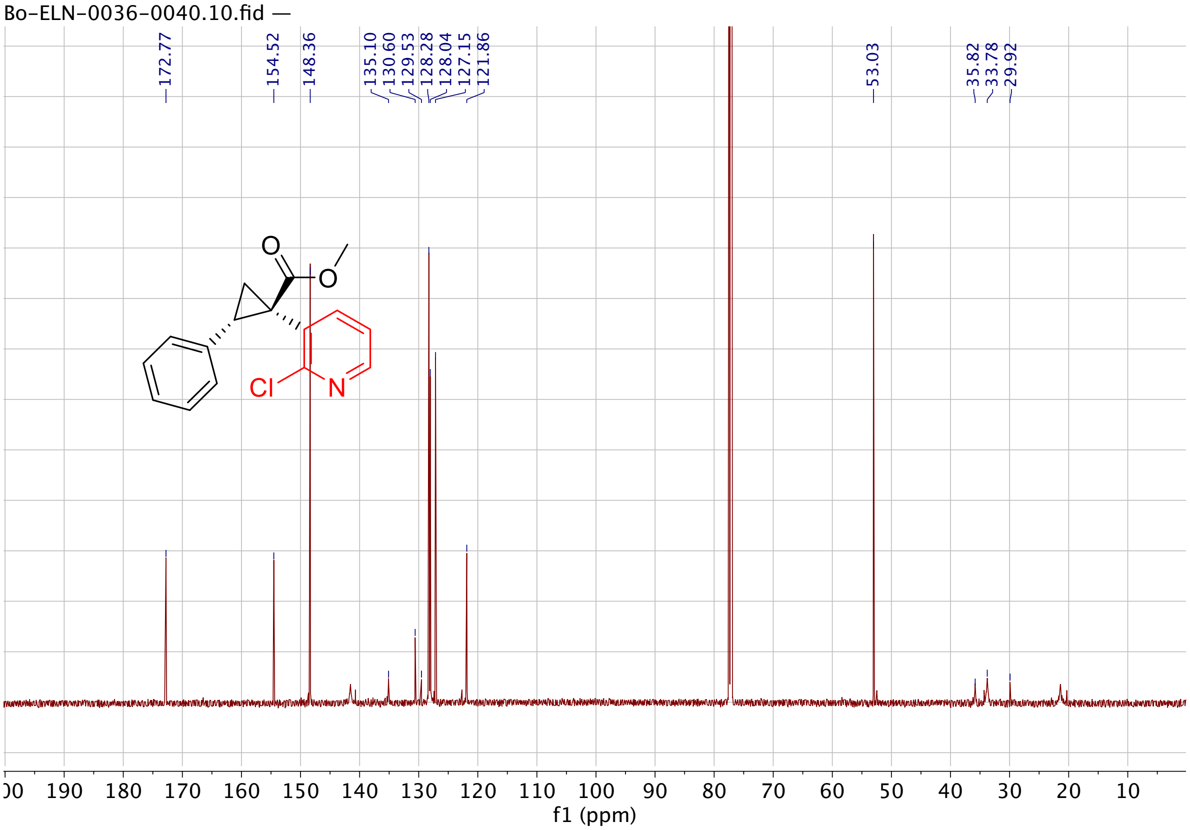
Bo-ELN-0036-0040.10.fid —
172.77
154.52
148.36
135.10
130.60
129.53
128.28
128.04
127.15
121.86
53.03
35.82
33.78
29.92
O
O
Cl
N
f1 (ppm)

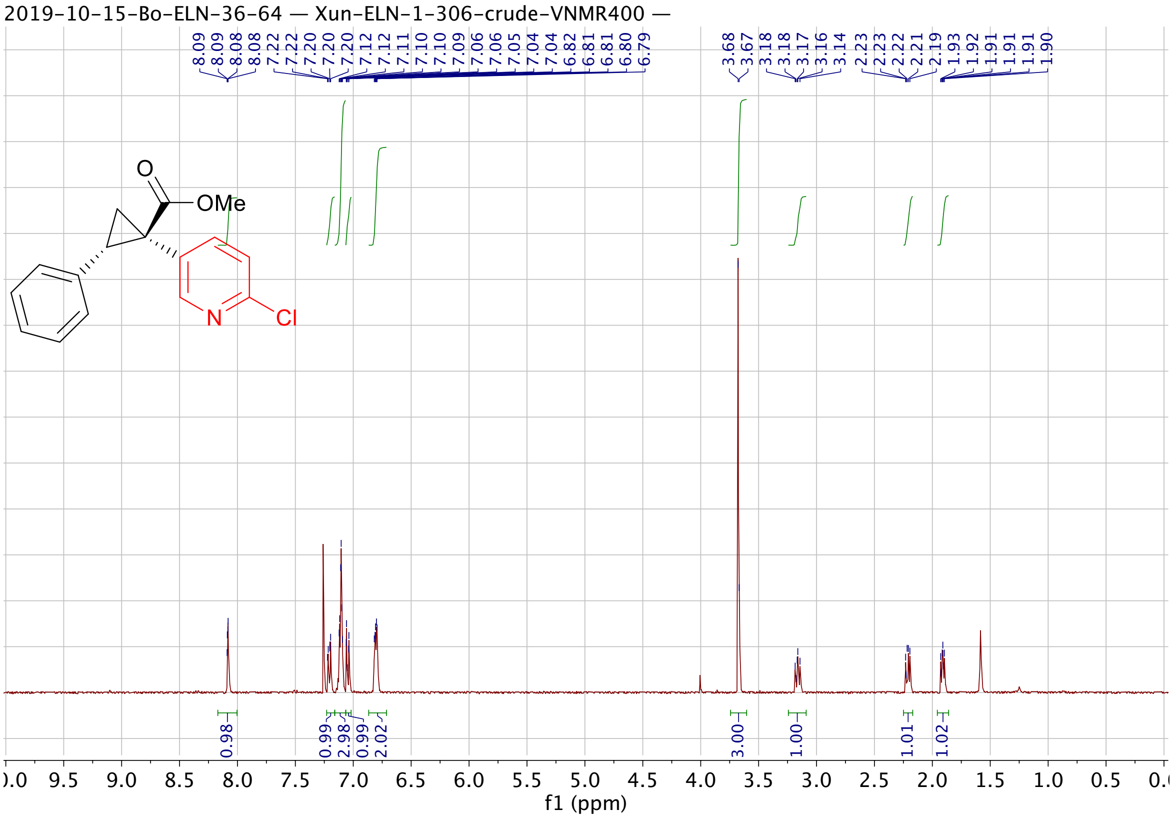
2019-10-15-Bo-ELN-36-64 — Xun-ELN-1-306-crude-VNMR400 —
8.09
8.09
8.08
8.08
7.22
7.22
7.20
7.20
7.20
7.12
7.12
7.11
7.10
7.10
7.09
7.06
7.06
7.05
7.04
7.04
6.82
6.81
6.81
6.80
6.79
3.68
3.67
3.18
3.18
3.17
3.16
3.14
2.23
2.23
2.22
2.21
2.19
1.93
1.92
1.91
1.91
1.91
1.90
O
OMe
N
Cl
0.98
0.99
2.98
0.99
2.02
3.00
1.00
1.01
1.02
f1 (ppm)

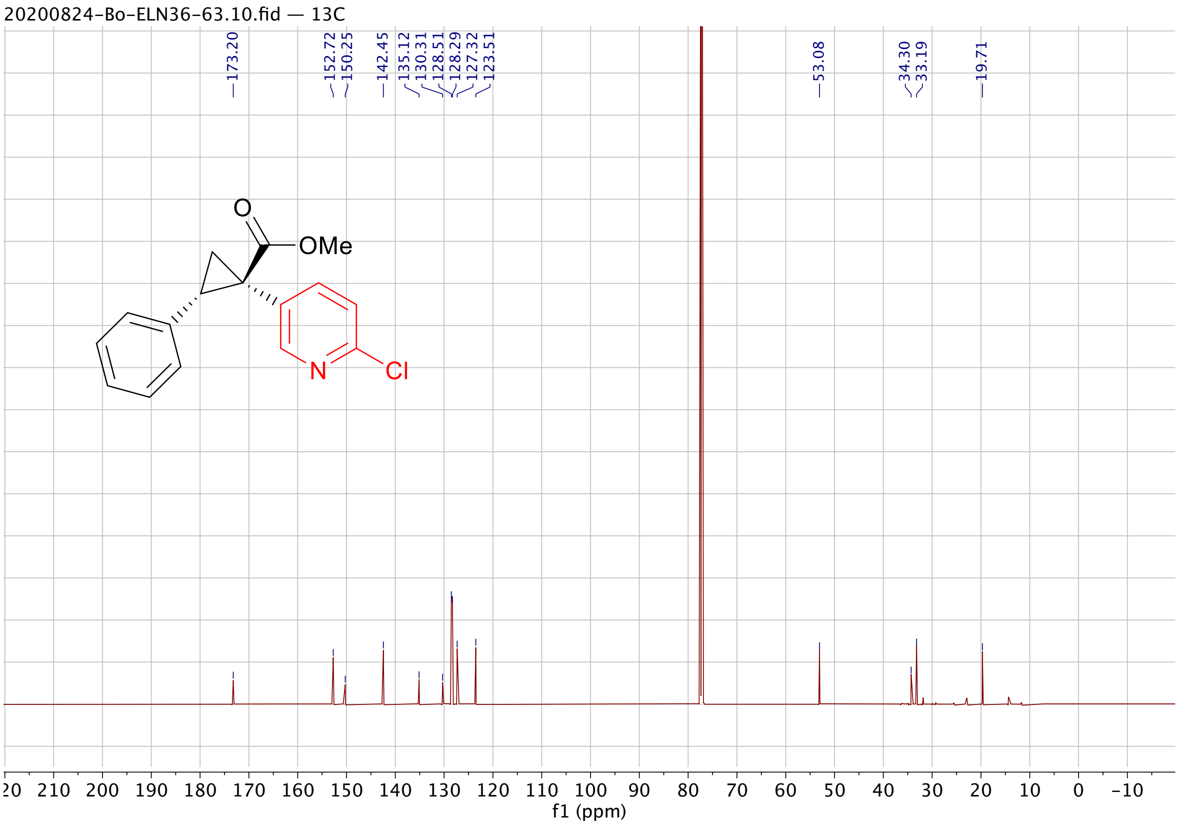
20200824-Bo-ELN36-63.10.fid — 13C
173.20
152.72
150.25
142.45
135.12
130.31
128.51
128.29
127.32
123.51
53.08
34.30
33.19
19.71
O
OMe
N
Cl
f1 (ppm)

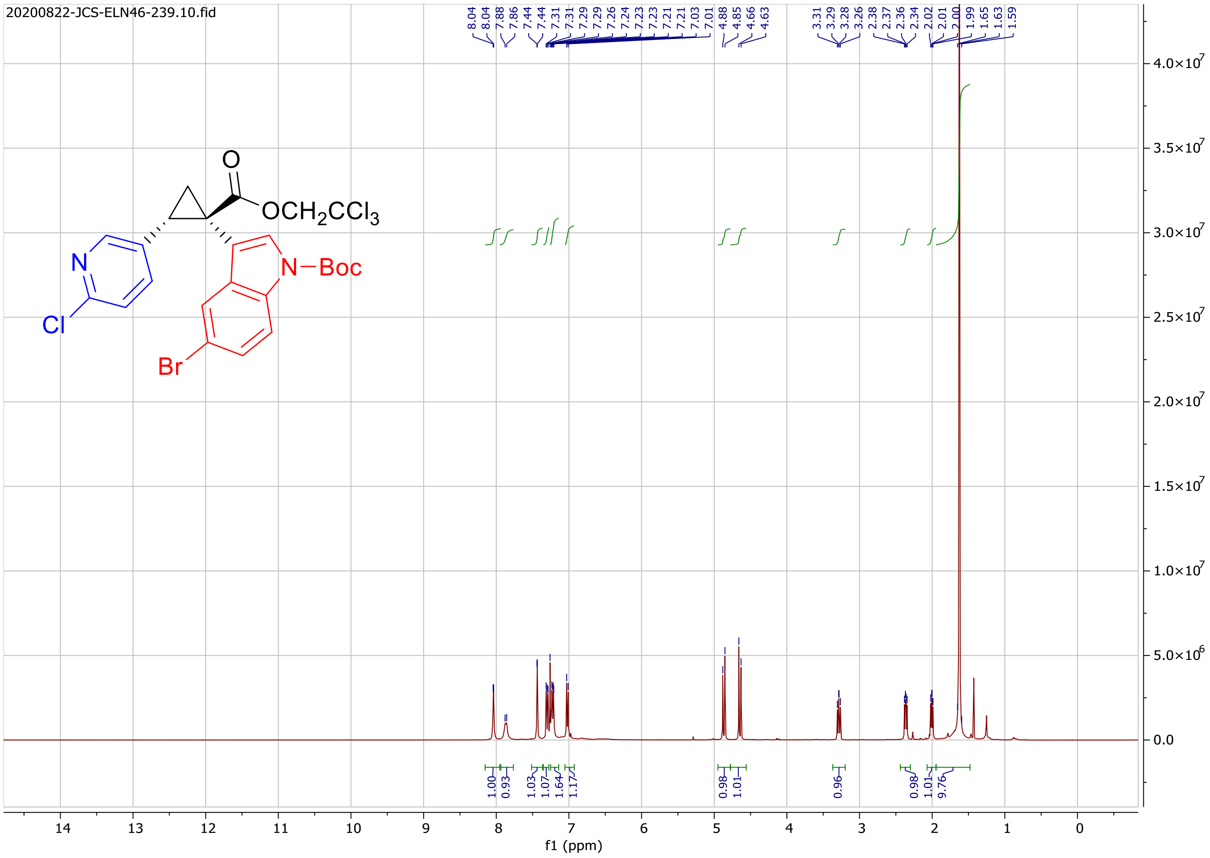
20200822-JCS-ELN46-239.10.fid
O
OCH2CCl3
N–Boc
N
Cl
Br
f1 (ppm)

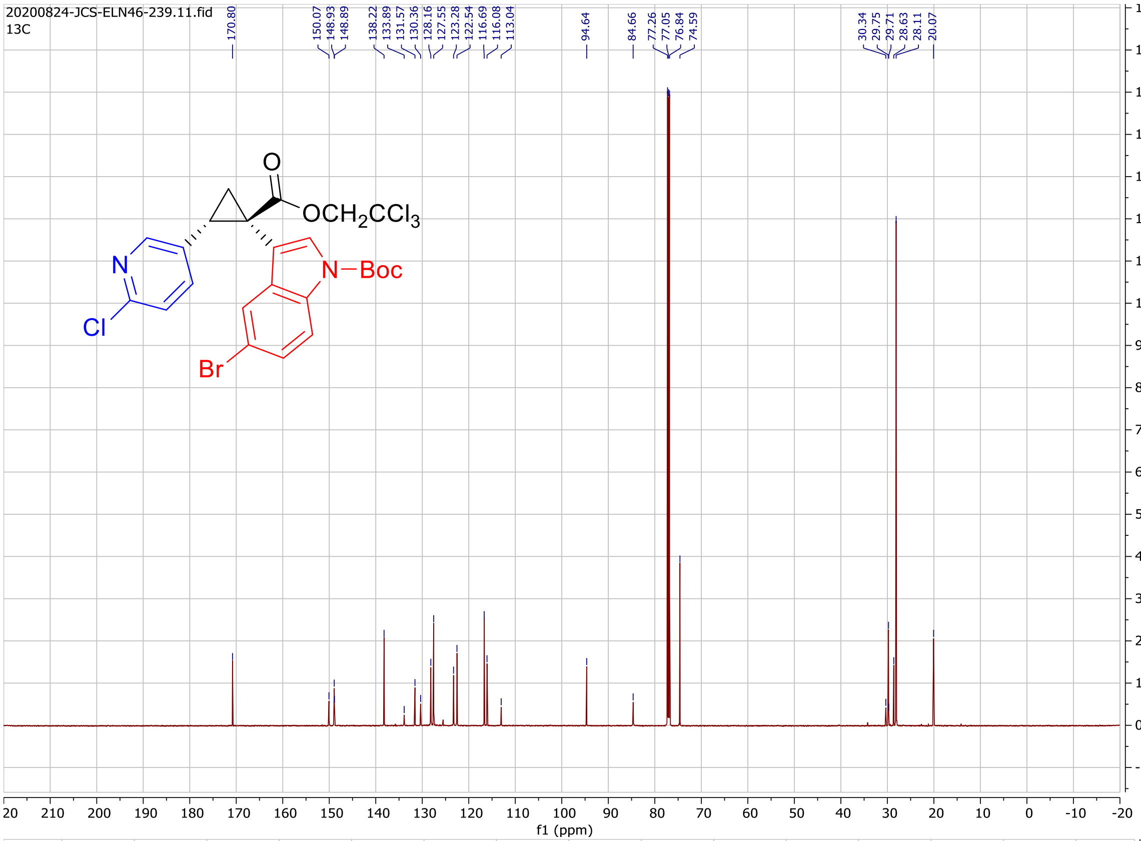
20200824-JCS-ELN46-239.11.fid
13C
170.80
150.07
148.93
148.89
138.22
133.89
131.57
130.36
128.16
127.55
123.28
122.54
116.69
116.08
113.04
94.64
84.66
77.26
77.05
76.84
74.59
30.34
29.75
29.71
28.63
28.11
20.07
O
OCH2CCl3
N
N−Boc
Cl
Br
f1 (ppm)

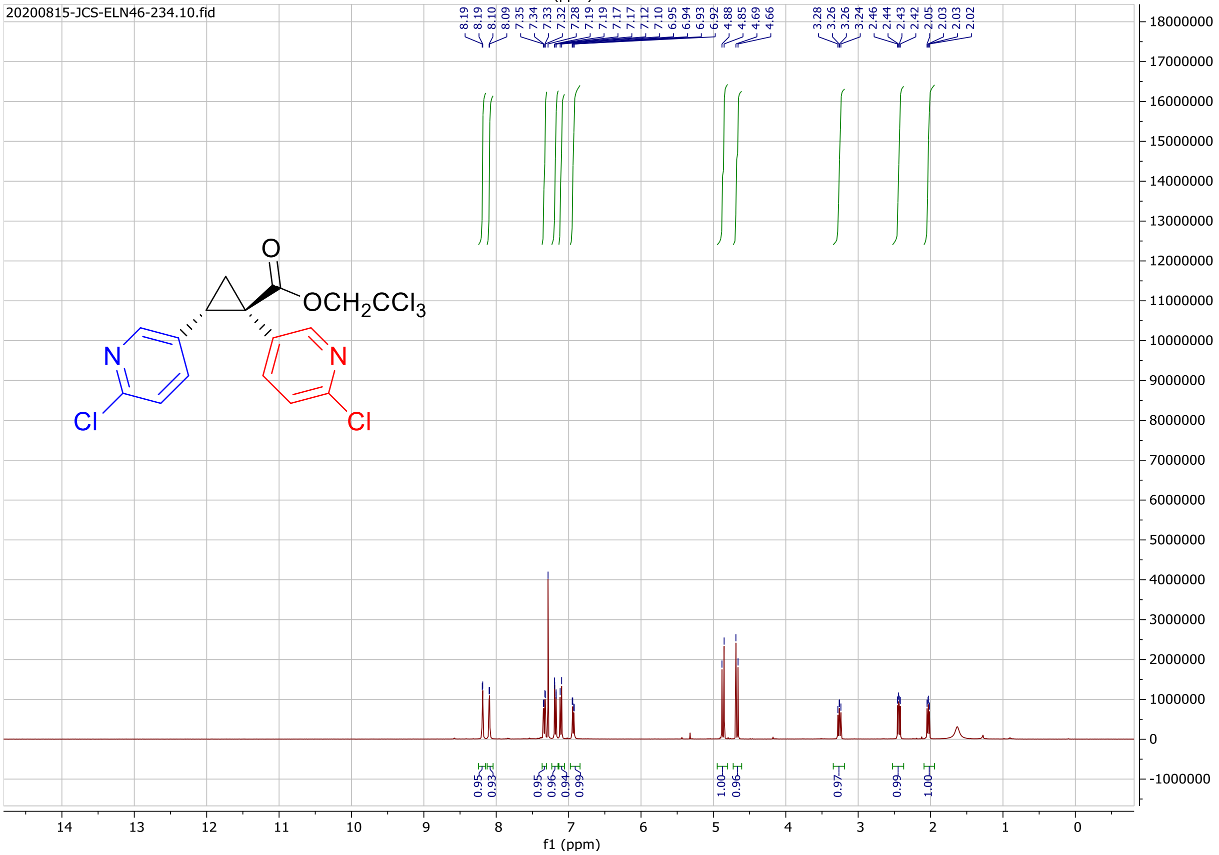
20200815-JCS-ELN46-234.10.fid
8.19
8.19
8.10
8.09
7.35
7.34
7.33
7.32
7.28
7.19
7.19
7.17
7.17
7.12
7.10
6.95
6.94
6.93
6.92
4.88
4.85
4.69
4.66
3.28
3.26
3.26
3.24
2.46
2.44
2.43
2.42
2.05
2.03
2.03
2.02
O
OCH2CCl3
N
N
Cl
Cl
0.95
0.93
0.95
0.96
0.94
0.99
1.00
0.96
0.97
0.99
1.00
f1 (ppm)

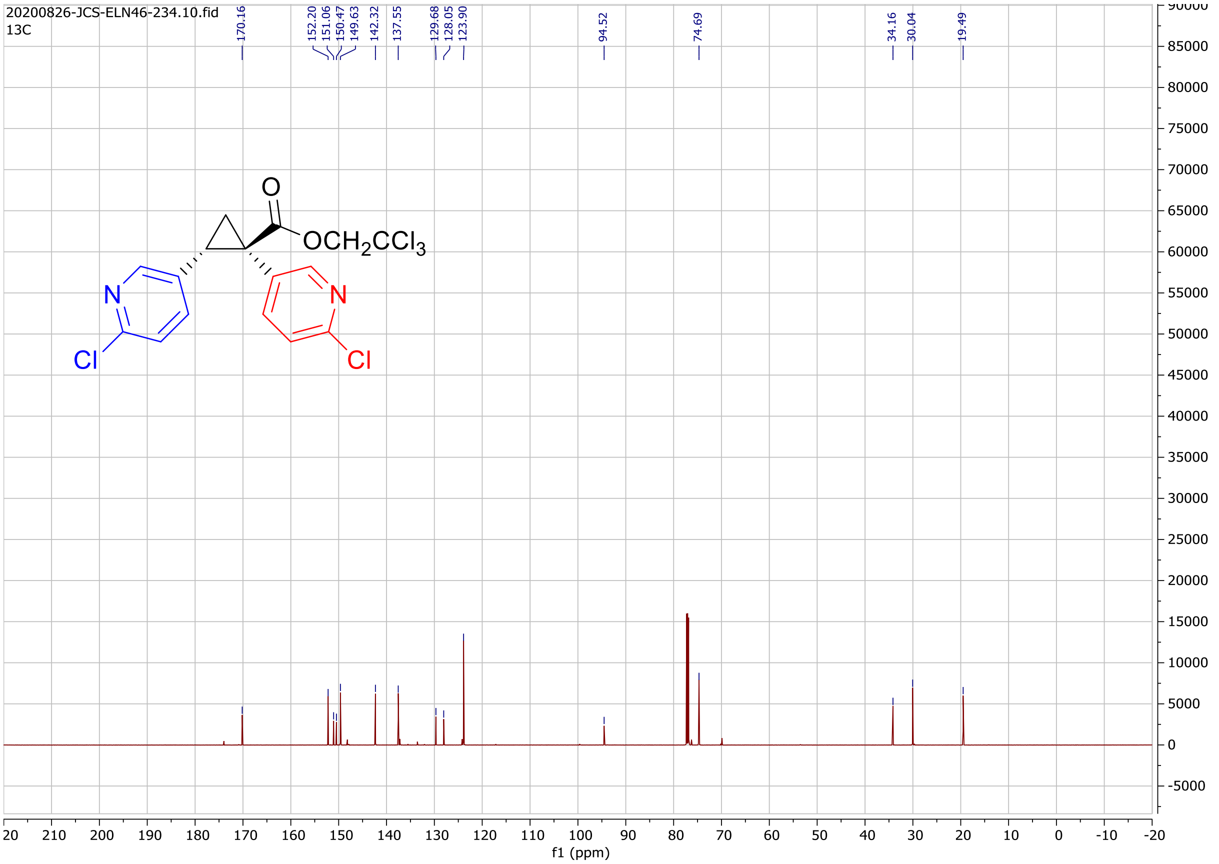

20200826-JCS-ELN46-234.10.fid
13C
170.16
152.20
151.06
150.47
149.63
142.32
137.55
129.68
128.05
123.90
94.52
74.69
34.16
30.04
19.49
O
OCH2CCl3
N
Cl
N
Cl
f1 (ppm)

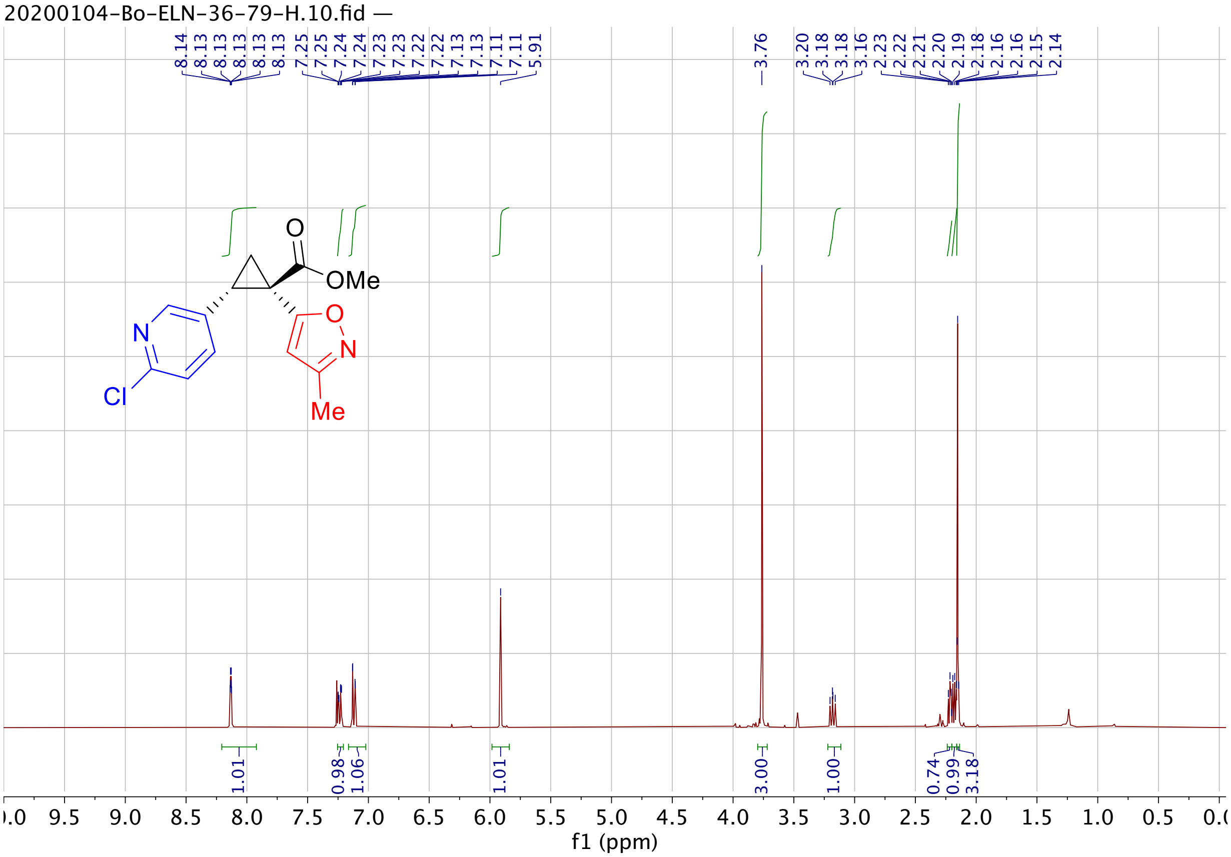

20200104-Bo-ELN-36-79-H.10.fid —
8.14
8.13
8.13
8.13
8.13
8.13
7.25
7.25
7.24
7.24
7.23
7.23
7.22
7.22
7.13
7.13
7.11
7.11
5.91
3.76
3.20
3.18
3.18
3.16
2.23
2.22
2.21
2.20
2.19
2.18
2.16
2.16
2.15
2.14
O
OMe
N
Cl
O
N
Me
1.01
0.98
1.06
1.01
3.00
1.00
0.74
0.99
3.18
f1 (ppm)

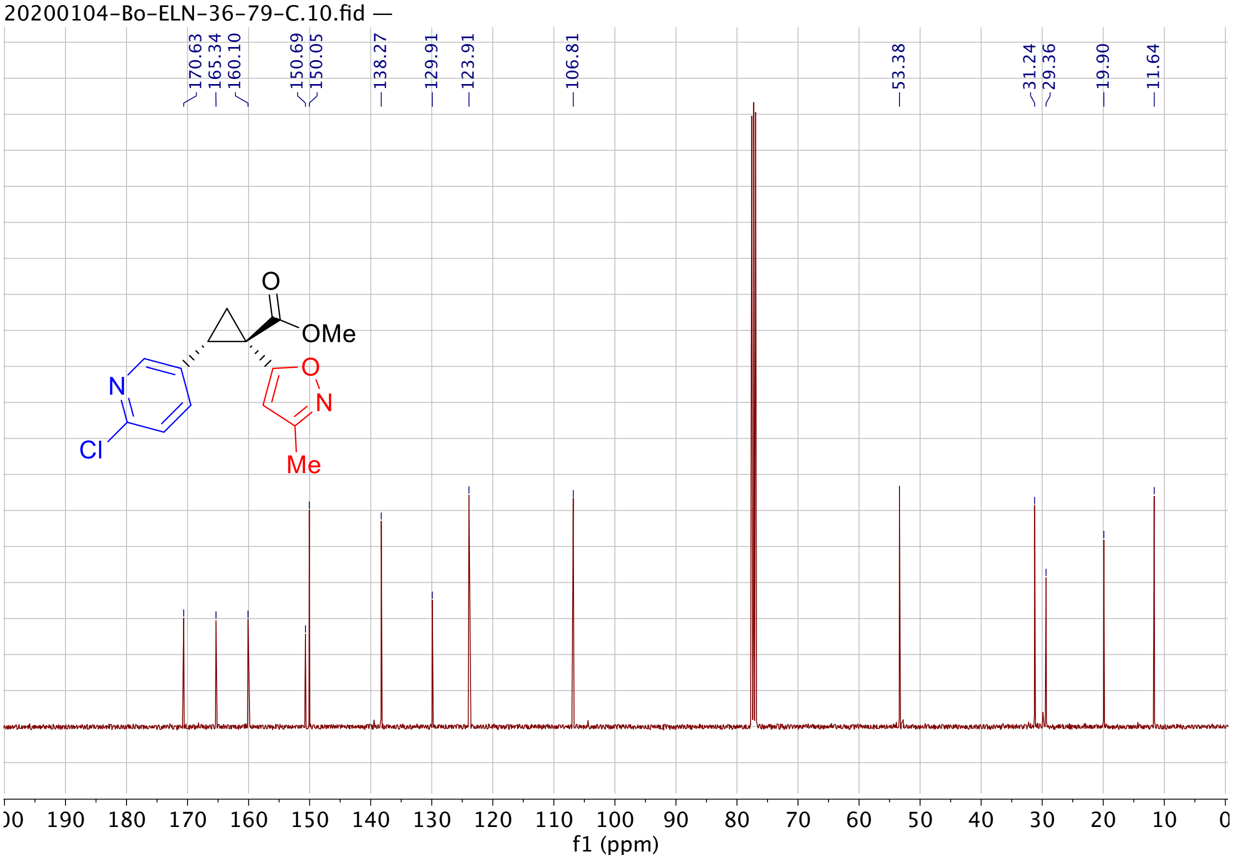
20200104-Bo-ELN-36-79-C.10.fid —
170.63
165.34
160.10
150.69
150.05
138.27
129.91
123.91
106.81
53.38
31.24
29.36
19.90
11.64
O
OMe
N
Cl
O
N
Me
00 190 180 170 160 150 140 130 120 110 100 90 80 70 60 50 40 30 20 10 0
f1 (ppm)

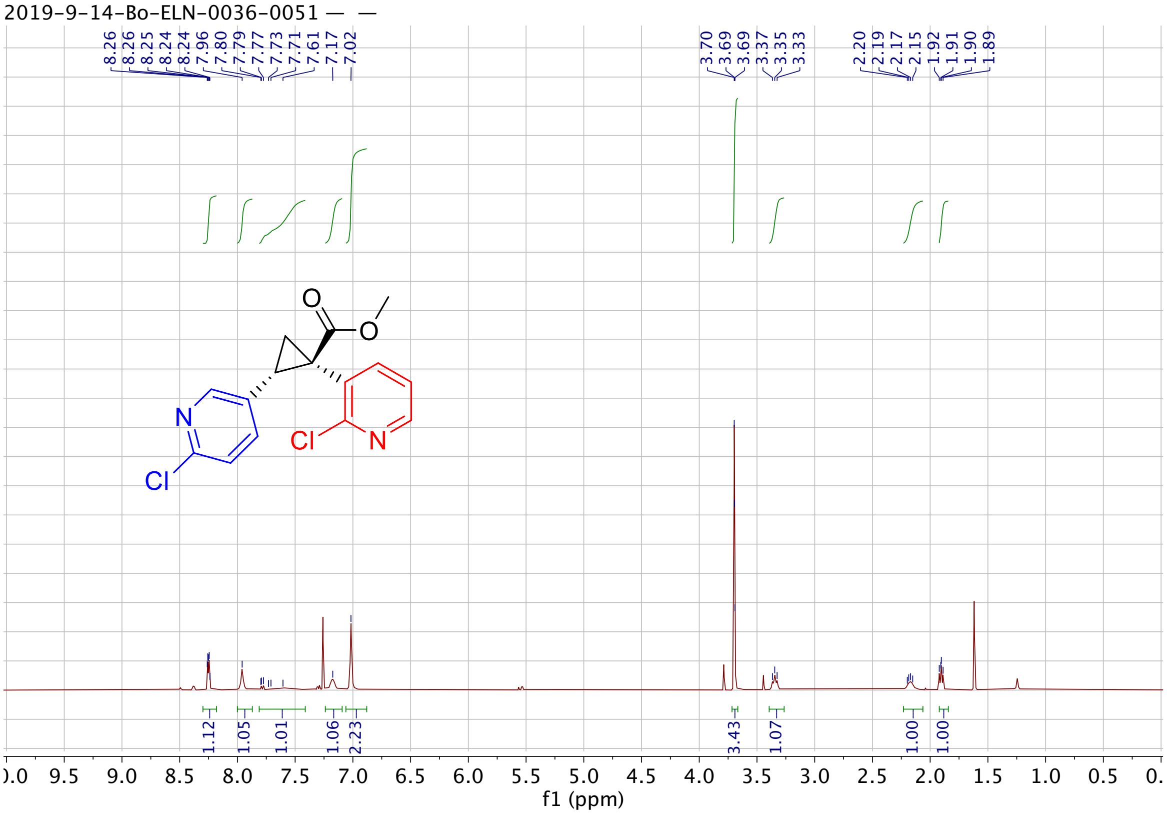
2019-9-14-Bo-ELN-0036-0051 — —
8.26
8.26
8.25
8.24
8.24
7.96
7.80
7.79
7.77
7.73
7.71
7.61
7.17
7.02
3.70
3.69
3.69
3.37
3.35
3.33
2.20
2.19
2.17
2.15
1.92
1.91
1.90
1.89
O
O
N
Cl
Cl
N
1.12
1.05
1.01
1.06
2.23
3.43
1.07
1.00
1.00
0.0 9.5 9.0 8.5 8.0 7.5 7.0 6.5 6.0 5.5 5.0 4.5 4.0 3.5 3.0 2.5 2.0 1.5 1.0 0.5 0.
f1 (ppm)

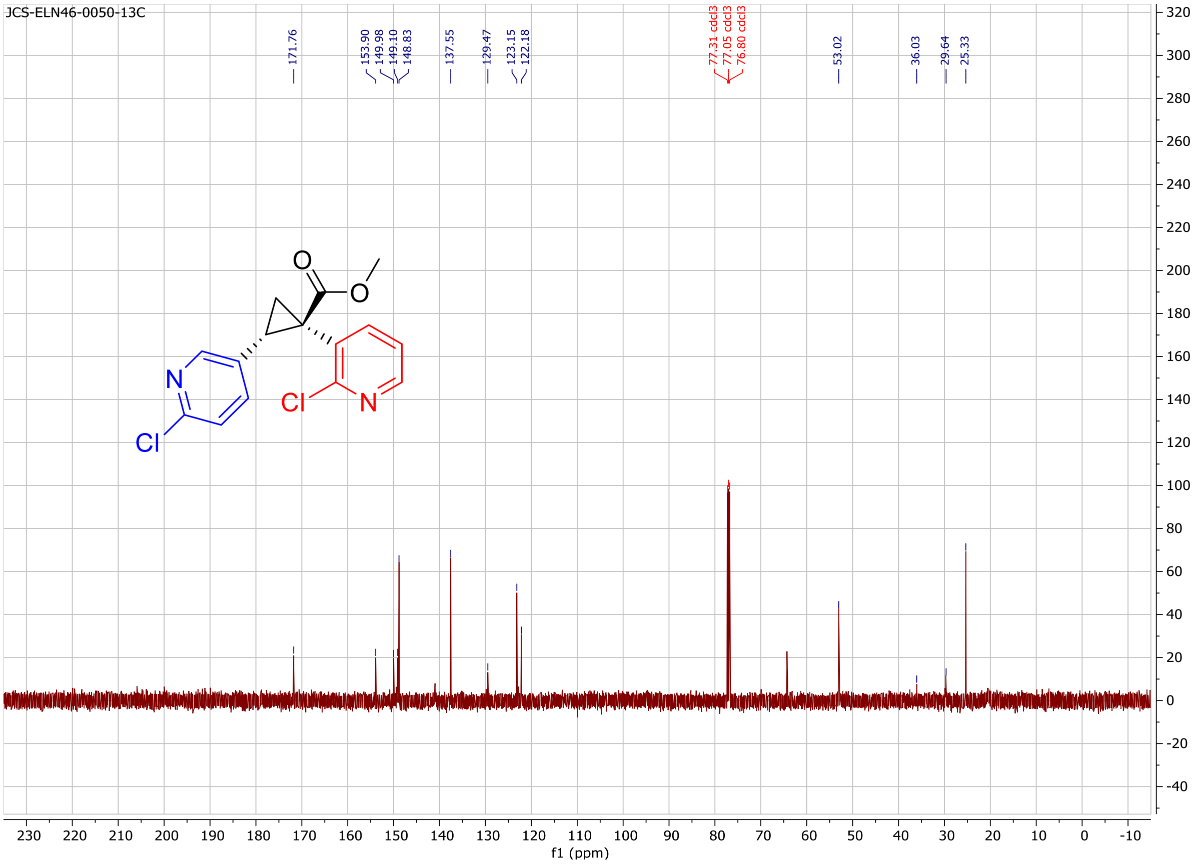
JCS-ELN46-0050-13C
171.76
153.90
149.98
149.10
148.83
137.55
129.47
123.15
122.18
77.31 cdcl3
77.05 cdcl3
76.80 cdcl3
53.02
36.03
29.64
25.33
O
O
N
Cl
Cl
N
f1 (ppm)

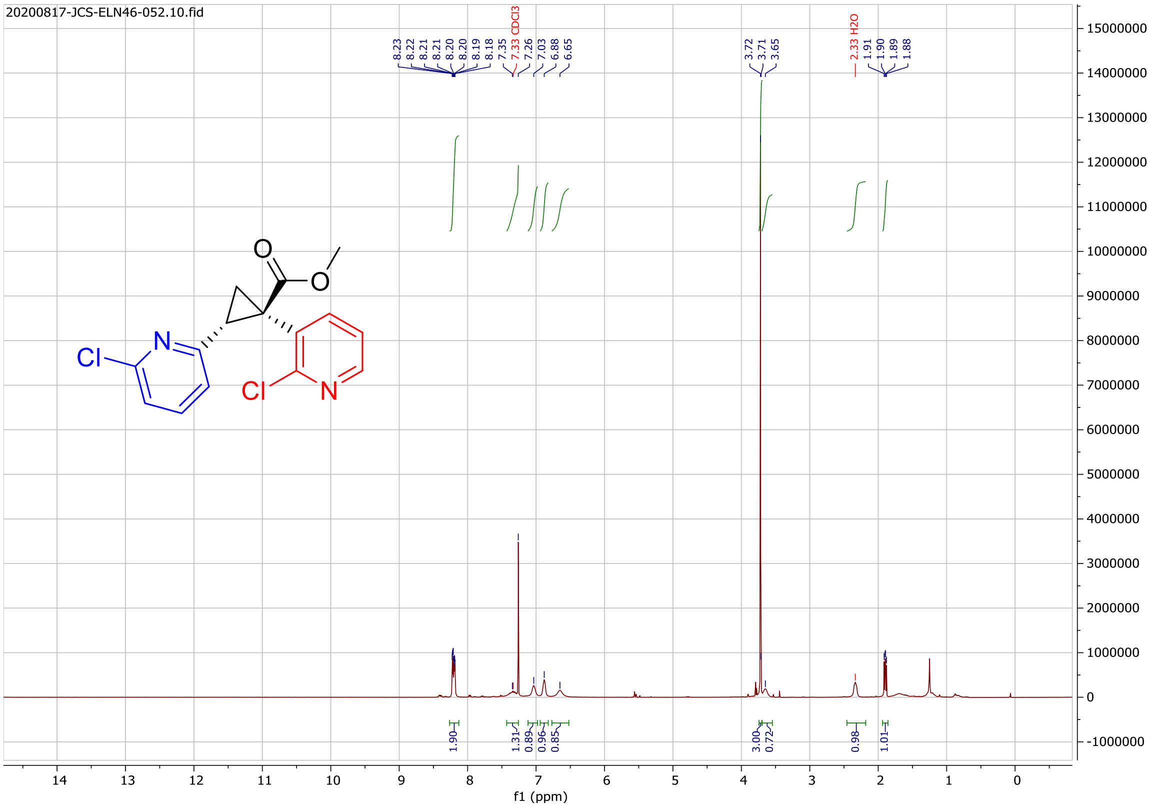
20200817-JCS-ELN46-052.10.fid
8.23
8.22
8.21
8.21
8.20
8.20
8.19
8.18
7.35
7.33 CDCl3
7.26
7.03
6.88
6.65
3.72
3.71
3.65
2.33 H2O
1.91
1.90
1.89
1.88
1.90
1.31
0.89
0.96
0.85
3.00
0.72
0.98
1.01
O
O
Cl
N
Cl
N
f1 (ppm)

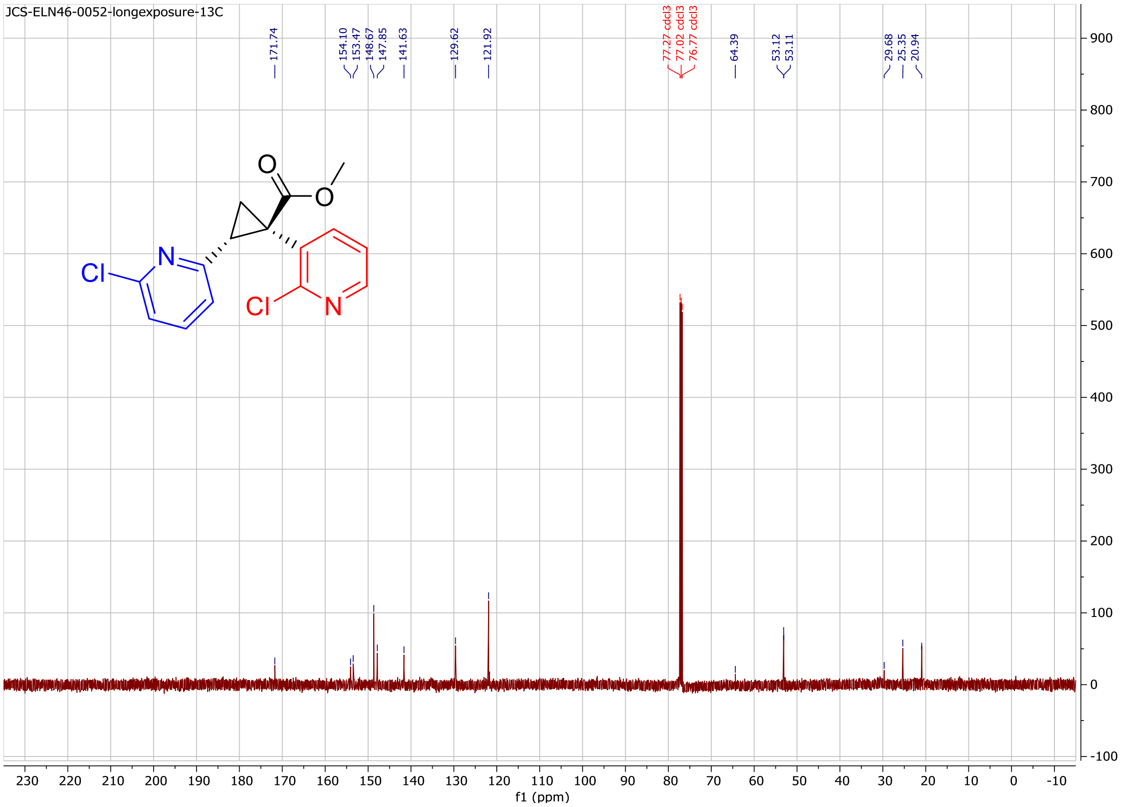
JCS-ELN46-0052-longexposure-13C
171.74
154.10
153.47
148.67
147.85
141.63
129.62
121.92
77.27 cdcl3
77.02 cdcl3
76.77 cdcl3
64.39
53.12
53.11
29.68
25.35
20.94
O
O
Cl
N
Cl
N
f1 (ppm)

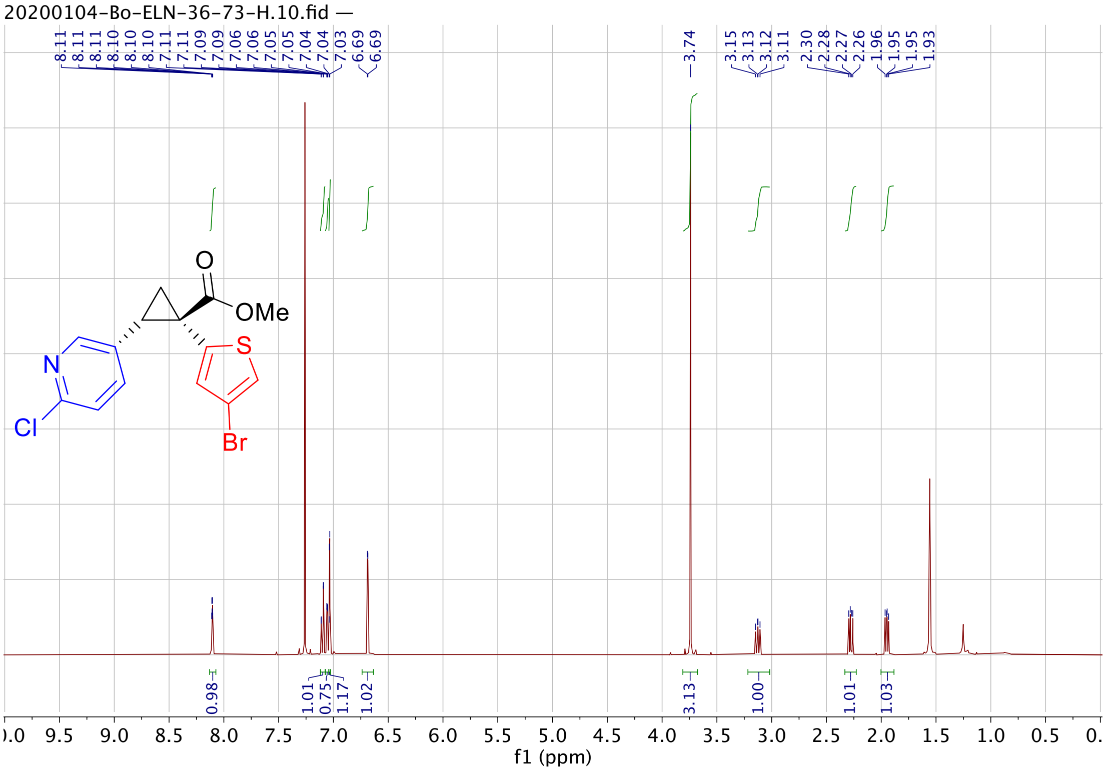
20200104-Bo-ELN-36-73-H.10.fid —
8.11
8.11
8.11
8.10
8.10
8.10
7.11
7.11
7.09
7.06
7.06
7.05
7.04
7.04
7.03
6.69
6.69
3.74
3.15
3.13
3.12
3.11
2.30
2.28
2.27
2.26
1.96
1.95
1.95
1.93
O
OMe
N
Cl
S
Br
0.98
1.01
0.75
1.17
1.02
3.13
1.00
1.01
1.03
f1 (ppm)

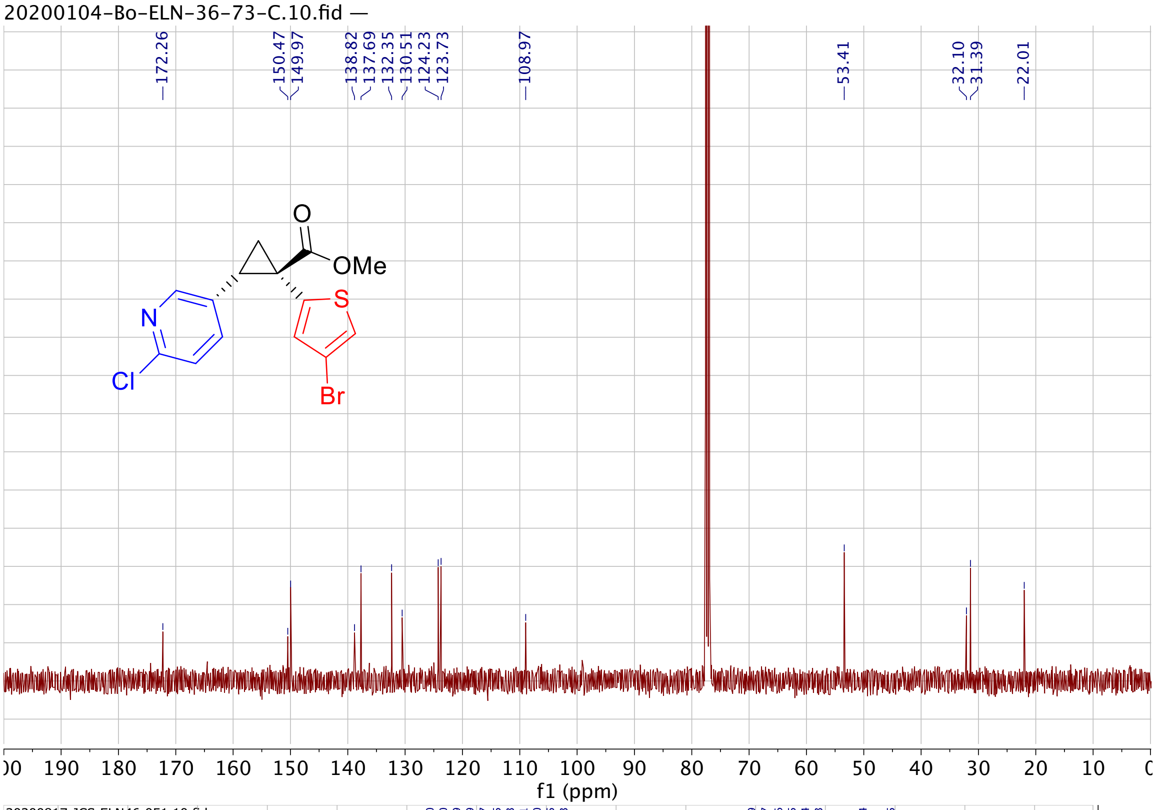
20200104-Bo-ELN-36-73-C.10.fid
172.26
150.47
149.97
138.82
137.69
132.35
130.51
124.23
123.73
108.97
53.41
32.10
31.39
22.01
O
OMe
N
S
Cl
Br
f1 (ppm)

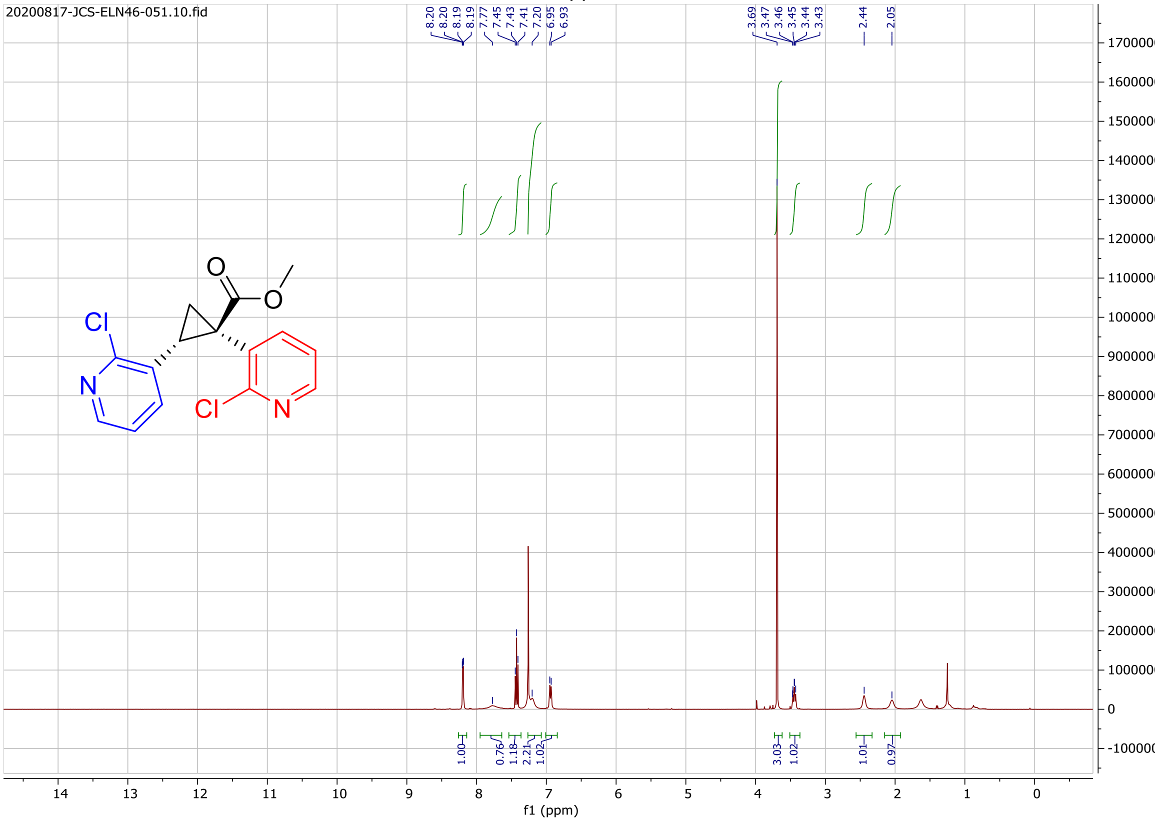
20200817-JCS-ELN46-051.10.fid
8.20
8.20
8.19
8.19
7.77
7.45
7.43
7.41
7.20
6.95
6.93
3.69
3.47
3.46
3.45
3.44
3.43
2.44
2.05
O
O
Cl
N
Cl
N
1.00
0.76
1.18
2.21
1.02
3.03
1.02
1.01
0.97
f1 (ppm)

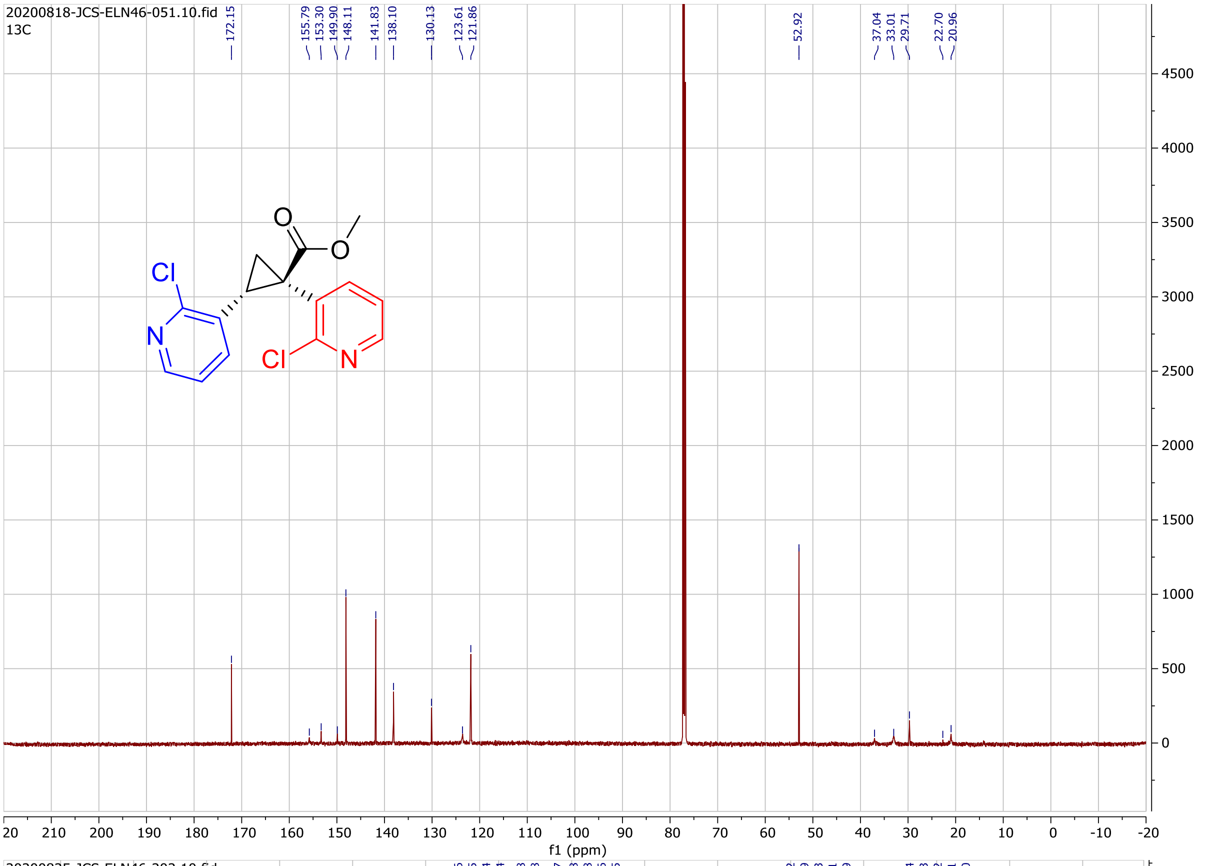
20200818-JCS-ELN46-051.10.fid
13C
172.15
155.79
153.30
149.90
148.11
141.83
138.10
130.13
123.61
121.86
52.92
37.04
33.01
29.71
22.70
20.96
Cl
N
O
O
Cl
N
f1 (ppm)

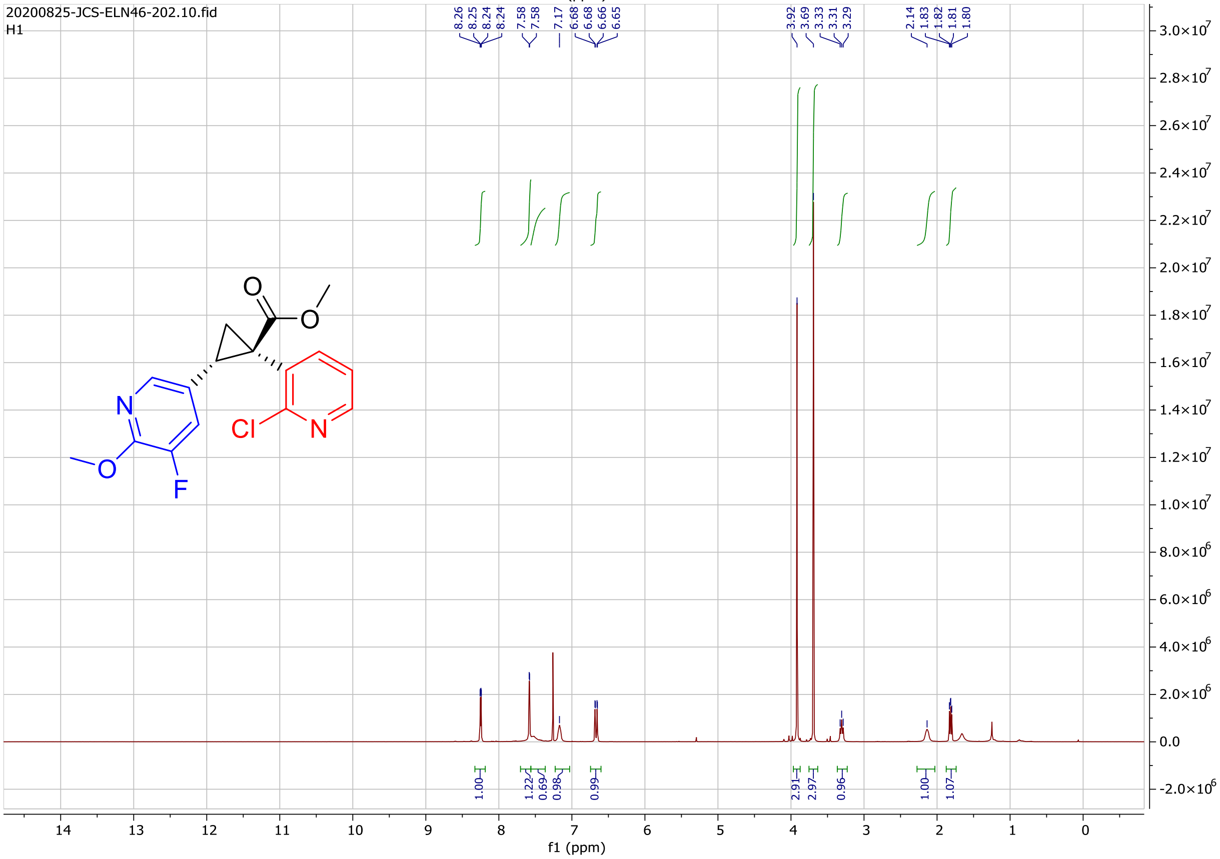
20200825-JCS-ELN46-202.10.fid
H1
8.26
8.25
8.24
8.24
7.58
7.58
7.17
6.68
6.68
6.66
6.65
3.92
3.69
3.33
3.31
3.29
2.14
1.83
1.82
1.81
1.80
O
O
N
O
F
Cl
N
1.00
1.22
0.69
0.98
0.99
2.91
2.97
0.96
1.00
1.07
f1 (ppm)

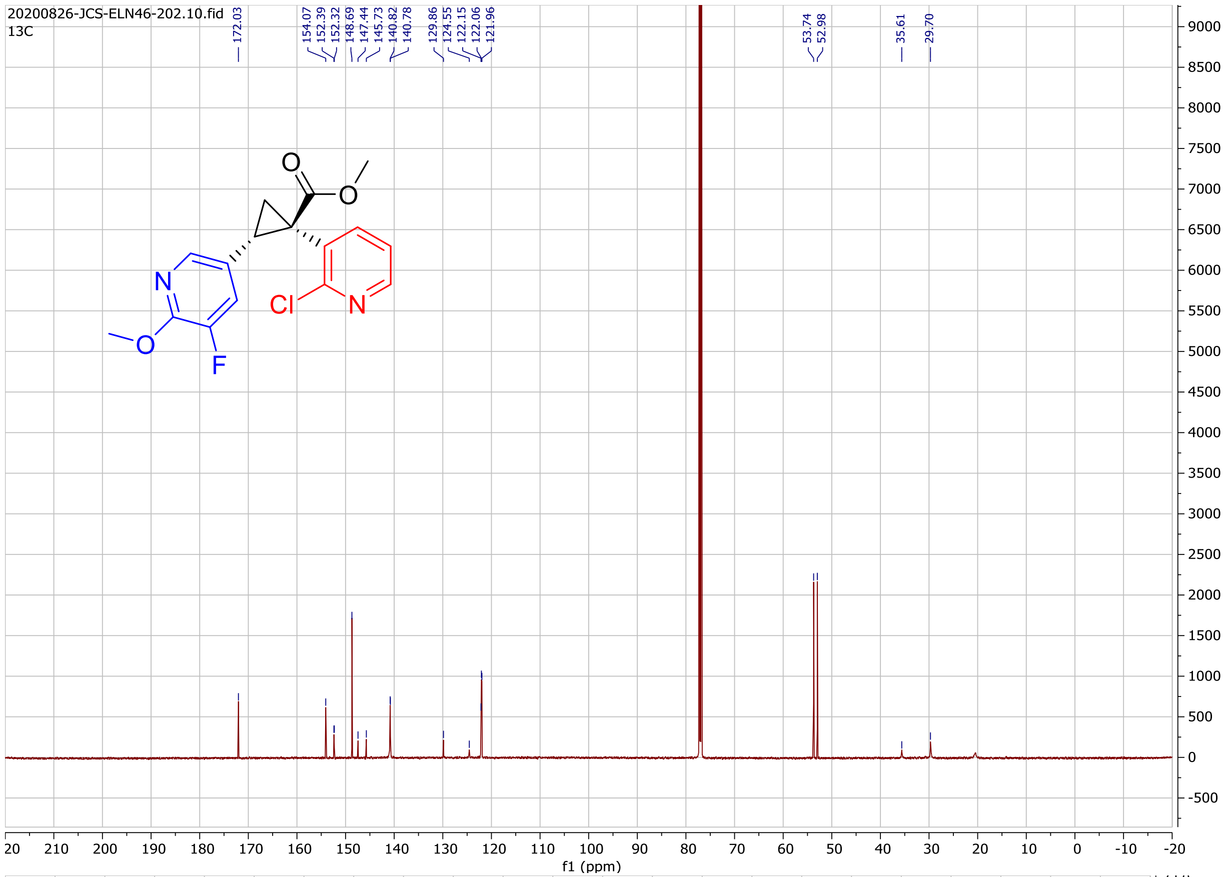
20200826-JCS-ELN46-202.10.fid
13C
172.03
154.07
152.39
152.32
148.69
147.44
145.73
140.82
140.78
129.86
124.55
122.15
122.06
121.96
53.74
52.98
35.61
29.70
O
O
N
Cl
N
O
F
f1 (ppm)

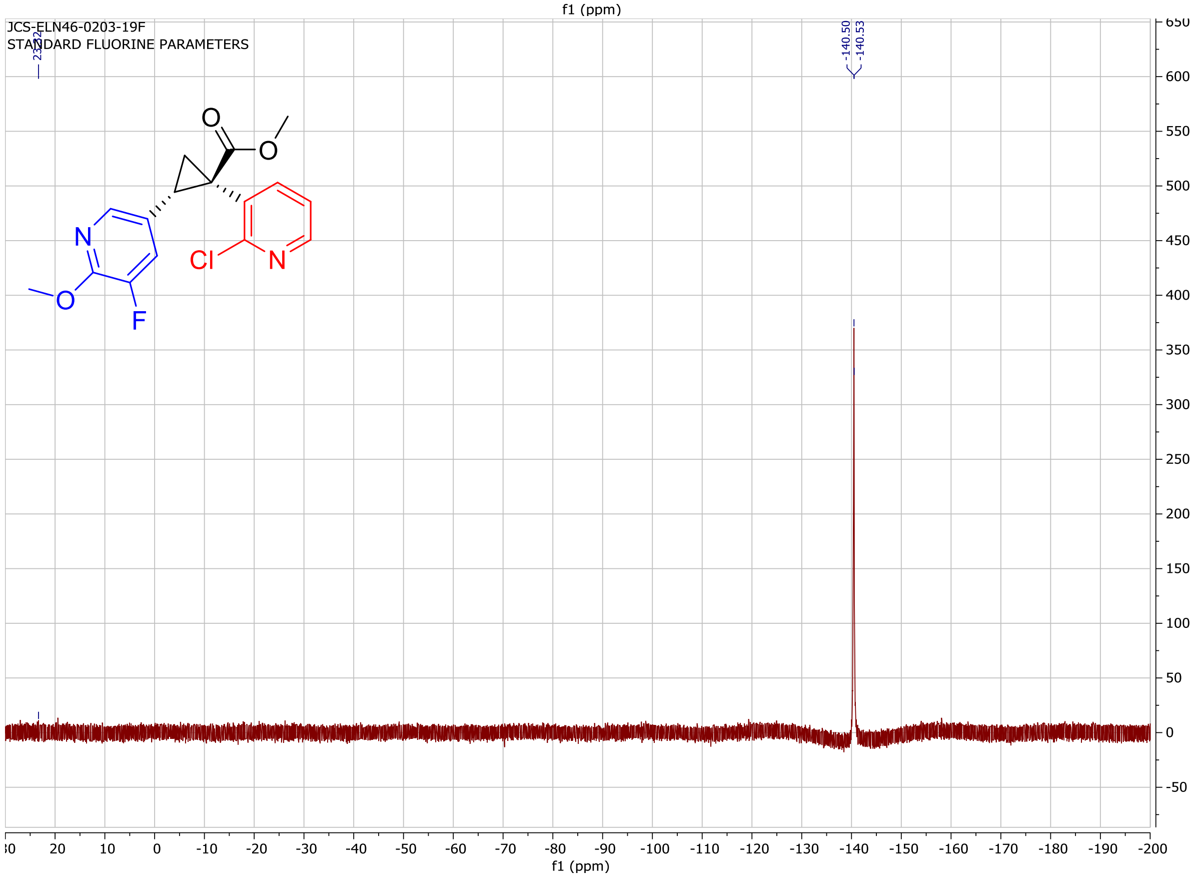
JCS-ELN46-0203-19F
STANDARD FLUORINE PARAMETERS
-140.50
-140.53
O
O
N
Cl
N
O
F
f1 (ppm)

20200104-Bo-ELN-36-55-H.10.fid —

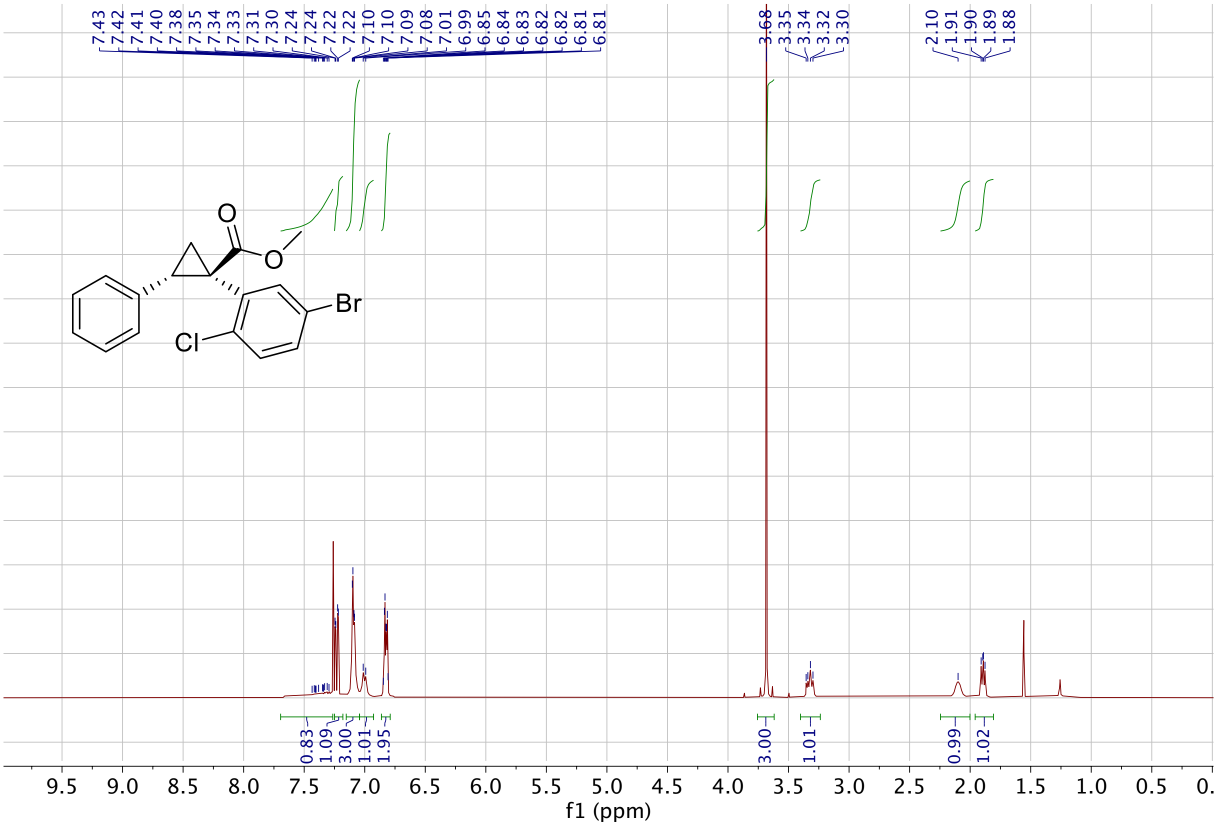

20200104-Bo-ELN-36-55-C.10.fid —

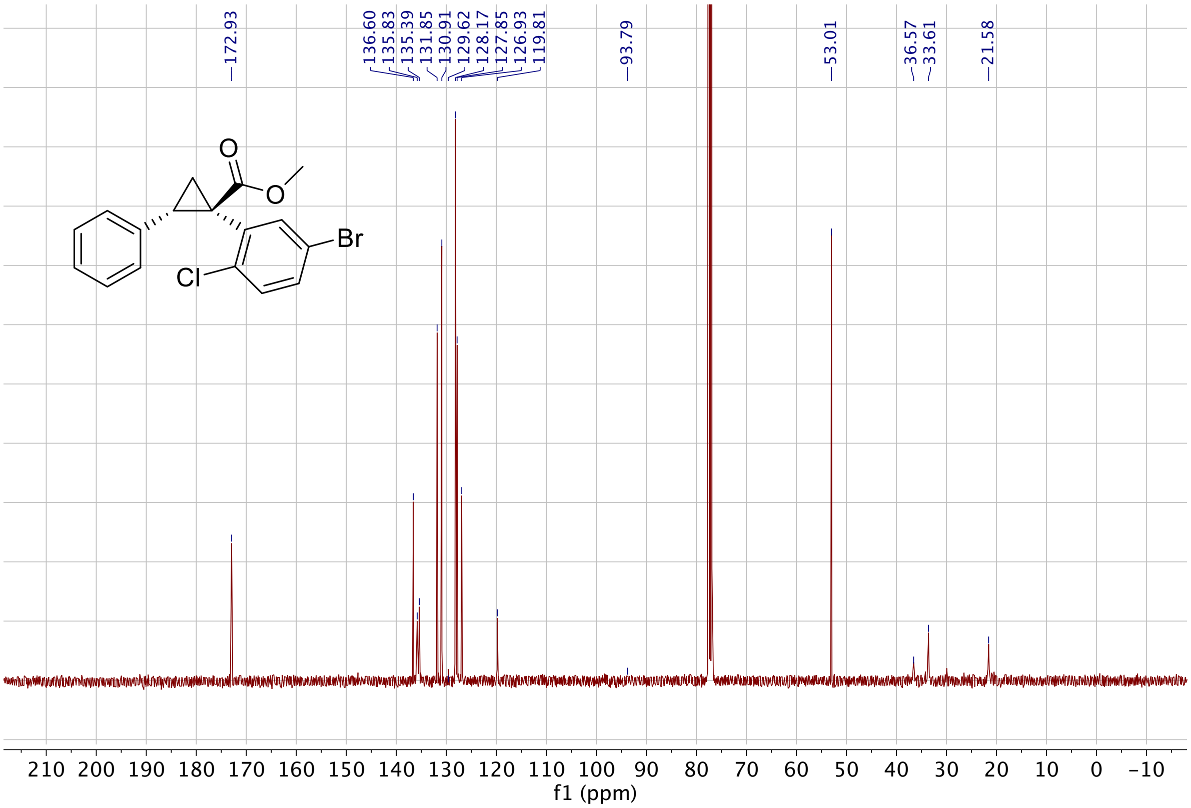

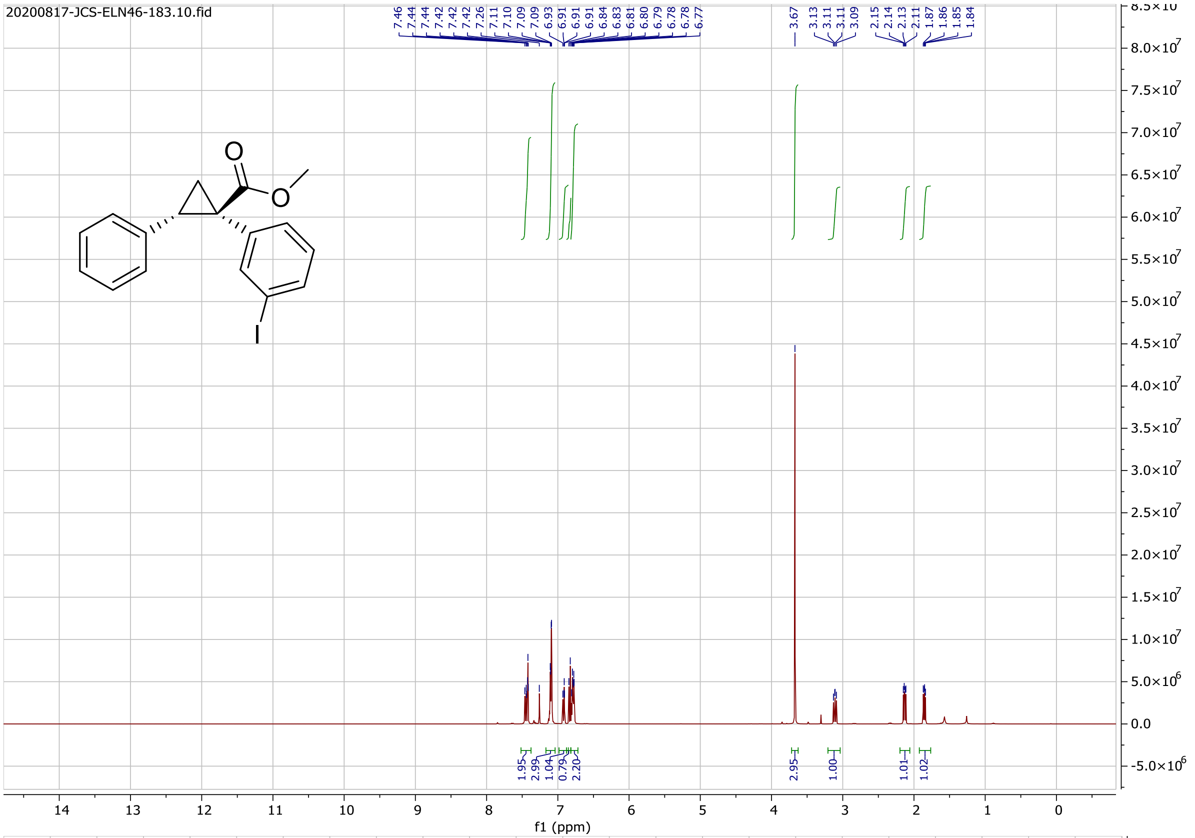
20200817-JCS-ELN46-183.10.fid
7.46
7.44
7.44
7.42
7.42
7.42
7.26
7.11
7.10
7.09
7.09
6.93
6.91
6.91
6.91
6.84
6.83
6.81
6.80
6.79
6.78
6.78
6.77
3.67
3.13
3.11
3.11
3.09
2.15
2.14
2.13
2.11
1.87
1.86
1.85
1.84
1.95
2.99
1.04
0.79
2.20
2.95
1.00
1.01
1.02
f1 (ppm)
O
O
I

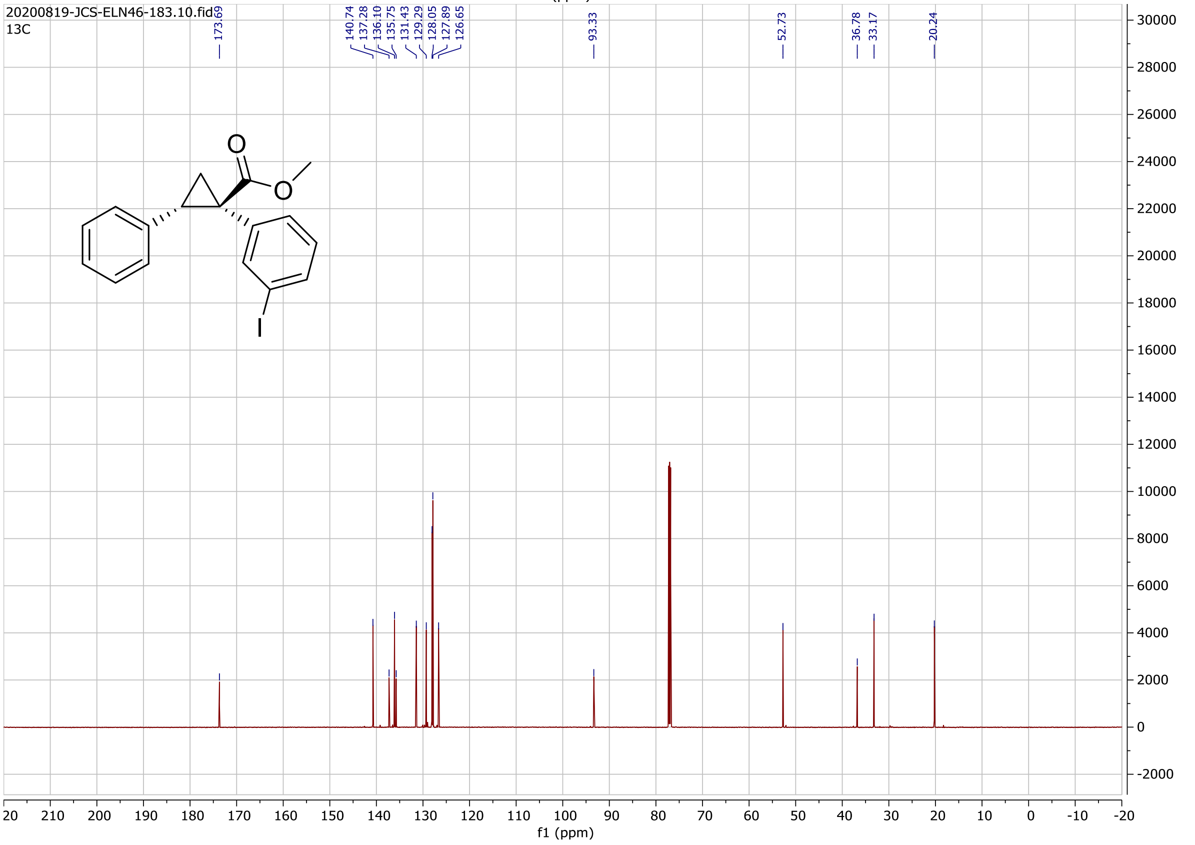
20200819-JCS-ELN46-183.10.fid
13C
173.69
140.74
137.28
136.10
135.75
131.43
129.29
128.05
127.89
126.65
93.33
52.73
36.78
33.17
20.24
f1 (ppm)
O
O
I

7. Crystallographic Data:
7.1: Rh_2(*R*-TPPTTL)$_4$ cocrystalized with 2-Clpyridine from CH_2Cl_2

JCS-Pyridine-TPPTTL-CoXtal-xtal2

Rigaku oxford diffraction

Submitted by: **Jack Sharland**
Emory University
Solved by: **John Bacsa**
Sample ID: **JCS-Pyridine-TPPTTL-CoXtal**

R_1=4.83%

Crystal Data and Experimental

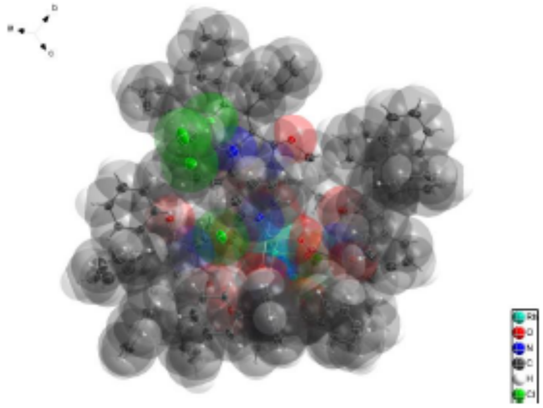

Experimental. Single red prism-shaped crystals of **JCS-Pyridine-TPPTTL-CoXtal-xtal2** were The crystal was chosen from the sample as supplied.. A suitable crystal 0.55×0.41×0.15 mm³ was selected and mounted on a loop with paratone oil on an XtaLAB Synergy, Dualflex, HyPix diffractometer. The crystal was kept at a steady T = 99.8(8) K during data collection. The structure was solved with the ShelXT n/a (Sheldrick, 2015) structure solution program using the Intrinsic Phasing solution method and by using **Olex2** (Dolomanov et al., 2009) as the graphical interface. The model was refined with version 2018/3 of ShelXL 2018/3 (Sheldrick, 2015) using Least Squares minimisation.

Crystal Data. $C_{168}H_{134}Cl_5N_7O_{16}Rh_2$, M_r = 2889.88, orthorhombic, $P2_12_12_1$ (No. 19), a = 20.07057(12) Å, b = 20.40295(11) Å, c = 41.1384(2) Å, $\alpha = \beta = \gamma$ = 90°, V = 16846.11(16) Å³, T = 99.8(8) K, Z = 4, Z' = 1, μ(Cu K_α) = 2.780, 123357 reflections measured, 34540 unique (R_{int} = 0.0491) which were used in all calculations. The final wR_2 was 0.1270 (all data) and R_1 was 0.0483 (I > 2(I)).

**Compound**	**JCS-Pyridine-TPPTTL-CoXtal-xtal2**
Formula	$C_{168}H_{134}Cl_5N_7O_{16}Rh_2$
$D_{calc.}$/ g cm^{-3}	1.139
μ/mm^{-1}	2.780
Formula Weight	2889.88
Colour	red
Shape	prism
Size/mm³	0.55×0.41×0.15
T/K	99.8(8)
Crystal System	orthorhombic
Flack Parameter	0.055(3)
Hooft Parameter	0.0273(15)
Space Group	$P2_12_12_1$
a/Å	20.07057(12)
b/Å	20.40295(11)
c/Å	41.1384(2)
α/°	90
β/°	90
γ/°	90
V/Å³	16846.11(16)
Z	4
Z'	1
Wavelength/Å	1.54184
Radiation type	Cu K_α
Θ_{min}/°	2.449
Θ_{max}/°	77.155
Measured Refl.	123357
Independent Refl.	34540
Reflections with I > 2(I)	33360
R_{int}	0.0491
Parameters	1847
Restraints	2461
Largest Peak	1.126
Deepest Hole	-1.091
GooF	1.028
wR_2 (all data)	0.1270
wR_2	0.1244
R_1 (all data)	0.0503
R_1	Oooops!

Structure Quality Indicators

**Reflections:**	d min (Cu) 0.79	I/σ 23.4	Rint 4.91%	complete 100% (IUCr) 100%	
**Refinement:**	Shift 0.007	Max Peak 1.1	Min Peak -1.1	GooF 1.028	Flack .055(3)

Experimental Extended. A red prism-shaped crystal with dimensions 0.55×0.41×0.15 mm^3 was mounted on a loop with paratone oil. Data were collected using an XtaLAB Synergy, Dualflex, HyPix diffractometer operating at T = 99.8(8) K.

Data were measured using ω scans of 0.5° per frame for 5.0/1.0 s using Cu K_α radiation. The total number of runs and images was based on the strategy calculation from the program CrysAlisPro (Rigaku, V1.171.40.53, 2019) The maximum resolution that was achieved was Θ = 77.155° (0.79 Å).

The diffraction pattern was indexed The total number of runs and images was based on the strategy calculation from the program CrysAlisPro (Rigaku, V1.171.40.53, 2019) and the unit cell was refined using CrysAlisPro (Rigaku, V1.171.40.53, 2019) on 80615 reflections, 65% of the observed reflections.

Data reduction, scaling and absorption corrections were performed using CrysAlisPro (Rigaku, V1.171.40.53, 2019). The final completeness is 99.90 % out to 77.155° in Θ. A gaussian absorption correction was performed using CrysAlisPro 1.171.40.53 (Rigaku Oxford Diffraction, 2019) Numerical absorption correction based on gaussian integration over a multifaceted crystal model Empirical absorption correction using spherical harmonics, implemented in SCALE3 ABSPACK scaling algorithm.. The absorption coefficient μ of this material is 2.780 mm^{-1} at this wavelength (λ = 1.542Å) and the minimum and maximum transmissions are 0.198 and 1.000.

The structure was solved and the space group $P2_12_12_1$ (# 19) determined by the ShelXT n/a (Sheldrick, 2015) structure solution program using Intrinsic Phasing and refined by Least Squares using version 2018/3 of ShelXL 2018/3 (Sheldrick, 2015). All non-hydrogen atoms were refined anisotropically. Hydrogen atom positions were calculated geometrically and refined using the riding model. Hydrogen atom positions were calculated geometrically and refined using the riding model.

_exptl_absorpt_process_details: CrysAlisPro 1.171.40.53 (Rigaku Oxford Diffraction, 2019) Numerical absorption correction based on gaussian integration over a multifaceted crystal model Empirical absorption correction using spherical harmonics, implemented in SCALE3 ABSPACK scaling algorithm.

There is a single molecule in the asymmetric unit, which is represented by the reported sum formula. In other words: Z is 4 and Z' is 1.

The Flack parameter was refined to 0.055(3). Determination of absolute structure using Bayesian statistics on Bijvoet differences using the Olex2 results in 0.0273(15). Note: The Flack parameter is used to determine chirality of the crystal studied, the value should be near 0, a value of 1 means that the stereochemistry is wrong and the model should be inverted. A value of 0.5 means that the crystal consists of a racemic mixture of the two enantiomers.

Table 1: Fractional Atomic Coordinates (×10^4) and Equivalent Isotropic Displacement Parameters (Å^2×10^3) for **JCS-Pyridine-TPPTTL-CoXtal-xtal2**. U_{eq} is defined as 1/3 of the trace of the orthogonalised U_{ij}.

Atom	x	y	z	U_{eq}
Rh1	7401.0(2)	5645.8(2)	7026.8(2)	18.67(8)
Rh2	6867.1(2)	5474.7(2)	6511.8(2)	17.97(8)
O1_1	8262.6(15)	5247.3(16)	6841.6(6)	20.8(7)
O2_1	7748.3(15)	5070.5(16)	6360.8(7)	23.5(7)

Atom	x	y	z	U_{eq}
O3_1	9064(2)	4279.7(17)	5752.0(8)	33.2(9)
O4_1	8140(2)	3654.2(17)	6718.2(8)	39.5(10)
N1_1	8699.8(19)	4095.8(14)	6277.0(7)	26.0(9)
C1_1	8240.9(17)	5047(2)	6554.8(8)	23.9(9)
C2_1	8866.7(16)	4738.4(16)	6404.6(9)	28.5(10)
C3_1	8788(2)	3929.8(17)	5949.9(8)	27.1(10)
C4_1	8510(3)	3260.0(17)	5907.4(8)	31.3(11)
C5_1	8301(3)	3040.4(16)	6211.7(9)	31.8(11)
C6_1	8356(3)	3604.4(18)	6444.4(9)	32.5(12)
C7_1	8096(3)	2397.0(17)	6265.1(8)	39.5(13)
C8_1	8065(3)	1987.1(16)	5986.9(8)	36.2(12)
C9_1	8250(3)	2211.9(19)	5676.6(9)	45.0(15)
C10_1	8474(3)	2868.8(17)	5631.4(8)	35.7(12)
C11_1	8678(2)	3110(2)	5303.8(9)	33.8(12)
C12_1	9157(3)	2797(2)	5123.3(11)	36.8(13)
C13_1	9333(3)	3034(3)	4818.3(13)	48.2(16)
C14_1	9024(4)	3571(3)	4690.4(12)	52.3(17)
C15_1	8536(3)	3880(3)	4861.8(12)	47.1(16)
C16_1	8355(3)	3662(2)	5171.7(11)	38.2(13)
C17_1	8158(3)	1779(2)	5385.5(12)	63.5(18)
C18_1	7675(4)	1914(4)	5154.1(18)	83(2)
C19_1	7580(6)	1492(5)	4887(2)	105(3)
C20_1	7980(6)	961(5)	4853(2)	112(3)
C21_1	8481(5)	831(4)	5072(2)	92(3)
C22_1	8556(4)	1224(4)	5345.8(18)	77(2)
C23_1	7875(2)	1285.2(17)	6027.9(11)	33.6(12)
C24_1	8279(2)	849.6(19)	6198.0(14)	37.7(13)
C25_1	8109(3)	195(2)	6222.7(15)	45.1(14)
C26_1	7548(3)	-37(2)	6078.1(15)	40.4(13)
C27_1	7135(3)	384(2)	5911.7(14)	40.4(13)
C28_1	7292(3)	1052(2)	5884.2(15)	41.1(14)
C29_1	7914(2)	2139(2)	6592.9(9)	38.2(13)
C30_1	8383(3)	2138(3)	6840.5(11)	46.5(15)
C31_1	8255(3)	1842(4)	7135.8(12)	70(2)
C32_1	7646(4)	1539(3)	7180.2(13)	70(2)
C33_1	7171(3)	1538(3)	6944.0(14)	62(2)
C34_1	7309(2)	1838(3)	6646.6(12)	43.2(15)
C35_1	9519.8(17)	4753(2)	6609.7(10)	34.6(12)
C36_1	9690(2)	5467(2)	6688.9(14)	40.9(14)
C37_1	10075(2)	4478(3)	6392.8(14)	47.9(16)
C38_1	9479(3)	4339(3)	6919.3(12)	44.4(15)
O1_2	7096.7(17)	4728.3(15)	7156.8(7)	24.9(7)
O2_2	6550.9(17)	4581.1(15)	6681.4(6)	20.5(6)
O3_2	6149.4(17)	2759.8(19)	6585.5(9)	34.4(9)
O4_2	5288.0(16)	4395.6(15)	7211.2(8)	26.2(7)
N1_2	5872.9(13)	3573.7(15)	6950.1(8)	21.0(8)
C1_2	6741(2)	4409.1(17)	6963.5(8)	20.7(9)
C2_2	6547.6(16)	3706.7(16)	7057.3(8)	22.5(9)
C3_2	5727.0(16)	3089.4(19)	6720.0(10)	24.1(9)
C4_2	4991.9(16)	3085.2(19)	6677.9(9)	21.7(9)
C5_2	4726.1(15)	3538(2)	6895.7(10)	23.1(9)
C6_2	5297.1(16)	3907.8(18)	7043.9(10)	20.5(8)
C7_2	4041.6(15)	3623(2)	6935.9(9)	23.2(9)
C8_2	3626.0(15)	3247(2)	6728.6(9)	23.6(9)
C9_2	3891.2(16)	2802(2)	6502.2(10)	29.5(10)
C10_2	4592.9(16)	2707.8(18)	6475.2(9)	23.9(9)
C11_2	4875(2)	2205.3(17)	6250.4(10)	26.9(10)
C12_2	4730(3)	1551.8(19)	6283.2(12)	35.3(12)
C13_2	5020(3)	1094(2)	6076.8(13)	44.1(14)
C14_2	5461(3)	1283(3)	5842.7(14)	51.7(16)
C15_2	5610(3)	1929(3)	5804.6(13)	44.4(15)
C16_2	5332(3)	2403(2)	6008.9(12)	31.7(11)
C17_2	3438(2)	2444(2)	6273.3(9)	32.7(11)

Atom	x	y	z	U_{eq}
C18_2	3415(3)	2607(3)	5946.5(10)	35.5(12)
C19_2	2962(3)	2294(3)	5736.8(12)	47.4(15)
C20_2	2530(3)	1846(3)	5859.2(14)	53.7(16)
C21_2	2562(3)	1656(3)	6179.8(14)	50.7(16)
C22_2	3002(3)	1967(3)	6390.8(12)	41.3(14)
C23_2	2888.4(16)	3307(3)	6759.6(10)	32.8(11)
C24_2	2561(2)	3143(3)	7046.3(11)	44.9(15)
C25_2	1879(2)	3206(4)	7073.7(14)	58.7(19)
C26_2	1514(2)	3457(4)	6825.0(16)	58.5(19)
C27_2	1826(2)	3641(4)	6541.3(14)	51.1(16)
C28_2	2517(2)	3568(3)	6503.9(12)	37.5(12)
C29_2	3760(2)	4047.4(19)	7197.1(8)	22.4(9)
C30_2	3886(3)	3903(2)	7520.6(9)	34.1(12)
C31_2	3598(3)	4262(3)	7768.0(10)	35.3(12)
C32_2	3196(3)	4787(2)	7687.0(11)	39.1(13)
C33_2	3083(3)	4958(2)	7372.2(11)	39.8(13)
C34_2	3364(3)	4581(2)	7124.8(10)	34.2(12)
C35_2	6699.7(19)	3480.0(18)	7411.7(8)	26.2(10)
C36_2	7454.5(19)	3395(3)	7448.4(12)	34.6(12)
C37_2	6373(3)	2807(2)	7456.2(12)	34.4(12)
C38_2	6427(3)	3946(2)	7671.1(10)	30.8(12)
O1_3	6520.9(14)	6016.7(17)	7187.7(7)	21.2(7)
O2_3	5999.8(15)	5859.7(17)	6708.7(6)	21.7(7)
O3_3	4007.0(17)	5913.1(15)	6940.4(10)	31.1(8)
O4_3	5696.9(14)	7404.3(18)	6870.9(10)	30.7(8)
N1_3	4942.4(14)	6571.8(14)	6969.9(8)	20.1(8)
C1_3	6017.6(16)	6010(2)	7008.3(8)	22.7(9)
C2_3	5334.3(16)	6122.7(17)	7165.7(7)	20.6(9)
C3_3	4285.3(17)	6428.5(17)	6881.0(12)	25.4(10)
C4_3	4021.7(17)	7016.6(17)	6710.9(11)	26.3(10)
C5_3	4536.0(17)	7472.4(17)	6687.5(11)	25.0(10)
C6_3	5140.1(17)	7179.7(18)	6847.1(13)	27.4(10)
C7_3	4437.5(16)	8091.9(16)	6552.8(11)	24.0(10)
C8_3	3785.2(16)	8234.8(17)	6439.4(11)	25.7(10)
C9_3	3260.2(16)	7783.1(17)	6470.9(11)	24.7(10)
C10_3	3377.1(16)	7149.9(16)	6606.7(10)	23.7(9)
C11_3	2825.2(18)	6666.8(18)	6647.3(10)	26.8(10)
C12_3	2335(3)	6751(2)	6875.9(13)	38.7(13)
C13_3	1855(3)	6268(3)	6922.8(14)	44.8(14)
C14_3	1853(3)	5711(2)	6739.1(13)	41.1(12)
C15_3	2331(2)	5621(2)	6508.5(15)	43.4(13)
C16_3	2831(3)	6088(2)	6460.5(13)	38.1(13)
C17_3	2586.1(19)	7929(2)	6331.2(11)	33.9(11)
C18_3	2387(2)	7654(3)	6038.7(11)	39.2(13)
C19_3	1757(3)	7800(3)	5907.1(13)	51.1(16)
C20_3	1337(3)	8198(3)	6073.8(16)	57.2(17)
C21_3	1516(3)	8462(3)	6369.0(16)	56.1(18)
C22_3	2143(2)	8334(3)	6498.3(14)	48.1(16)
C23_3	3661(2)	8871.5(18)	6271.5(10)	29.1(11)
C24_3	3321(3)	9382(2)	6422.1(13)	41.9(14)
C25_3	3270(3)	9989(2)	6275.5(14)	44.9(15)
C26_3	3522(3)	10092(2)	5973.2(14)	47.6(15)
C27_3	3864(4)	9597(3)	5818.7(13)	51.3(16)
C28_3	3940(3)	8982(2)	5965.3(11)	39.0(13)
C29_3	4983.7(18)	8585.1(17)	6521.5(11)	25.9(10)
C30_3	4913(2)	9194.9(18)	6666.6(11)	27.2(11)
C31_3	5378(2)	9683.8(19)	6618.9(12)	29.4(11)
C32_3	5890(3)	9575(2)	6400.7(14)	37.6(13)
C33_3	5966(2)	8988(2)	6248.0(13)	29.0(11)
C34_3	5504(2)	8491(2)	6307.1(12)	25.7(10)
C35_3	5324.7(18)	6280.3(18)	7537.1(8)	22.4(9)
C36_3	5659(3)	5720(2)	7723.2(10)	30.8(11)
C37_3	4593(2)	6306(3)	7643.4(13)	35.7(13)

Atom	x	y	z	U_{eq}
C38_3	5655(3)	6937(2)	7616.8(11)	28.9(11)
O1_4	7664.5(17)	6560.2(15)	6863.8(6)	21.1(6)
O2_4	7183.1(17)	6385.2(15)	6375.8(7)	22.2(7)
O3_4	7225.6(15)	8155.8(18)	5900.4(8)	28.5(8)
O4_4	8757.2(18)	6698.2(17)	6286.6(8)	29.2(8)
N1_4	7946.7(16)	7474.4(16)	6179.6(7)	23.0(8)
C1_4	7486(2)	6732.9(16)	6585.3(8)	19.7(8)
C2_4	7581.4(18)	7451.9(15)	6484.4(7)	21.8(9)
C3_4	7737.3(18)	7837(2)	5909.5(8)	22.2(9)
C4_4	8256.3(18)	7753(2)	5654.5(8)	21.9(9)
C5_4	8731.6(18)	7318(2)	5774.6(8)	20.4(9)
C6_4	8521(2)	7113(2)	6109.8(8)	23.3(9)
C7_4	9279.0(17)	7111(2)	5592.7(8)	22.0(9)
C8_4	9323.7(17)	7358(2)	5271.5(8)	22.3(9)
C9_4	8851.5(17)	7803.2(18)	5148.9(8)	18.6(9)
C10_4	8290.3(18)	7996.8(18)	5339.6(7)	20.1(9)
C11_4	7783(2)	8451.0(18)	5199.0(9)	24.5(10)
C12_4	7668(3)	9063(2)	5325.3(11)	33.1(11)
C13_4	7236(3)	9492(2)	5168.3(12)	37.2(12)
C14_4	6940(3)	9322(2)	4882.1(12)	40.4(13)
C15_4	7049(3)	8719(2)	4751.0(12)	38.4(13)
C16_4	7469(3)	8270(2)	4905.5(10)	29.6(11)
C17_4	8966(2)	8129.3(19)	4827.0(9)	26.5(10)
C18_4	8957(3)	7779(2)	4538.6(10)	32.8(11)
C19_4	9078(3)	8096(3)	4240.3(10)	38.7(13)
C20_4	9191(3)	8751(3)	4236.1(11)	42.3(14)
C21_4	9201(3)	9110(2)	4519.3(12)	39.6(13)
C22_4	9066(3)	8806(2)	4814.7(11)	34.7(12)
C23_4	9918.8(18)	7198(2)	5069.5(9)	27.0(10)
C24_4	10484(2)	7593(2)	5070.5(14)	38.2(13)
C25_4	11021(2)	7453(3)	4872.2(16)	52.0(16)
C26_4	10992(3)	6950(3)	4656.1(15)	56.3(17)
C27_4	10437(3)	6553(3)	4648.4(15)	55.9(17)
C28_4	9894(2)	6669(2)	4854.9(12)	36.7(13)
C29_4	9820.4(19)	6700.3(18)	5735.7(9)	21.7(9)
C30_4	10169(2)	6920(2)	6005.8(12)	34.2(12)
C31_4	10705(2)	6574(3)	6127.7(12)	38.0(12)
C32_4	10862(3)	5976(3)	5990.1(13)	40.9(13)
C33_4	10512(3)	5733(2)	5733.8(14)	42.0(14)
C34_4	9989(2)	6101(2)	5604.5(12)	31.8(11)
C35_4	7861(2)	7927.9(17)	6748.5(9)	27.7(10)
C36_4	7402(3)	7909(2)	7045.5(10)	40.0(14)
C37_4	7830(3)	8621.6(19)	6605.1(12)	36.3(13)
C38_4	8582(2)	7778(3)	6842.7(14)	41.8(14)
Cl1_6	6347.3(16)	4809.5(13)	3951.1(7)	86.1(8)
Cl2_6	6937.2(14)	4803.1(12)	4589.3(6)	74.3(6)
C1_6	6780(5)	5278(4)	4241(2)	61(2)
Cl3_8	6901.3(10)	4252.9(8)	5875.9(4)	49.5(4)
N2_8	6405(2)	5392.1(19)	6009.3(9)	28.1(8)
C39_8	6496(3)	4972(2)	5770.9(11)	36.1(10)
C40_8	6300(3)	5039(3)	5455.1(12)	46.4(13)
C41_8	5979(4)	5617(3)	5377.5(13)	50.8(14)
C42_8	5848(3)	6080(3)	5609.0(12)	37.7(11)
C43_8	6063(3)	5946(2)	5928.3(12)	31.4(9)
Cl3_9	7639.0(11)	6475.5(8)	4654.3(4)	52.7(4)
N2_9	7487(3)	6451(2)	5278.7(10)	47.4(11)
C39_9	7805(3)	6163(3)	5038.1(12)	41.6(11)
C40_9	8246(3)	5656(3)	5057.7(13)	47.1(12)
C41_9	8360(4)	5411(3)	5365.6(14)	54.9(15)
C42_9	8051(4)	5672(3)	5631.5(14)	58.3(15)
C43_9	7605(3)	6195(3)	5579.5(12)	49.1(12)
Cl3_10	7299(3)	5526(5)	7934.6(13)	111(3)
N2_10	8074(6)	5947(9)	7480.2(18)	60.7(15)

Atom	x	y	z	U_{eq}
C39_10	8026(6)	5891(11)	7797.8(18)	63.8(15)
C40_10	8479(6)	6087(10)	8025(2)	61(2)
C41_10	9022(7)	6429(10)	7908(3)	65(3)
C42_10	9135(8)	6487(14)	7582(3)	81(7)
C43_10	8635(7)	6254(11)	7371(2)	65(4)
Cl3_13	7504(2)	6675.2(17)	7722.4(9)	70.4(11)
N2_13	7930(6)	5559(4)	7514.7(16)	60.7(15)
C39_13	7892(7)	5924(5)	7777.3(18)	63.8(15)
C40_13	8157(11)	5794(6)	8076(2)	99(6)
C41_13	8429(10)	5177(6)	8113(2)	89(5)
C42_13	8524(9)	4773(5)	7852(2)	79(4)
C43_13	8243(6)	4971(4)	7554(2)	59(3)

Table 2: Anisotropic Displacement Parameters (×10⁴) JCS-Pyridine-TPPTTL-CoXtal-xtal2. The anisotropic displacement factor exponent takes the form: $-2\pi^2[h^2a^{*2} \times U_{11} + ... + 2hka^* \times b^* \times U_{12}]$

Atom	U_{11}	U_{22}	U_{33}	U_{23}	U_{13}	U_{12}
Rh1	21.98(16)	19.39(16)	14.64(14)	1.71(12)	1.31(12)	-1.71(13)
Rh2	22.74(16)	16.51(15)	14.67(14)	0.78(12)	0.07(13)	-1.57(13)
O1_1	16.8(16)	24.6(17)	20.9(15)	-1.1(12)	-4.4(12)	4.5(13)
O2_1	35.4(19)	17.0(16)	18.0(15)	-6.0(12)	0.7(13)	2.3(14)
O3_1	46(2)	28.9(19)	24.1(17)	10.1(15)	12.4(16)	0.7(17)
O4_1	64(3)	21.7(18)	33.0(19)	6.2(15)	23(2)	-1.9(19)
N1_1	30(2)	17.8(18)	30(2)	7.7(15)	13.7(18)	2.6(16)
C1_1	31(2)	18(2)	23(2)	0.6(17)	0.4(17)	-9.4(18)
C2_1	35(3)	16(2)	34(3)	9.4(18)	-3(2)	2.9(19)
C3_1	28(3)	24(2)	29(2)	8.5(18)	8(2)	6.0(19)
C4_1	37(3)	24(2)	33(2)	6.8(19)	11(2)	4(2)
C5_1	36(3)	25(2)	34(3)	4.7(19)	11(2)	2(2)
C6_1	43(3)	24(2)	30(2)	6.1(19)	9(2)	3(2)
C7_1	56(4)	21(2)	42(3)	8(2)	17(3)	6(2)
C8_1	42(3)	20(2)	47(3)	6(2)	7(3)	0(2)
C9_1	43(4)	41(3)	50(3)	6(2)	14(3)	-13(3)
C10_1	43(3)	31(3)	33(3)	2(2)	7(2)	-4(2)
C11_1	42(3)	29(3)	30(3)	-1(2)	9(2)	-9(2)
C12_1	50(3)	29(3)	32(3)	-2(2)	7(2)	-3(2)
C13_1	59(4)	44(4)	42(3)	3(3)	20(3)	3(3)
C14_1	69(5)	59(4)	29(3)	6(3)	6(3)	3(3)
C15_1	60(4)	54(4)	28(3)	5(3)	4(3)	2(3)
C16_1	52(4)	35(3)	27(3)	-6(2)	8(2)	1(2)
C17_1	67(3)	61(3)	62(3)	-3(2)	4(2)	-9(2)
C18_1	83(3)	84(3)	82(3)	4(2)	-2(2)	-7(3)
C19_1	107(4)	106(4)	103(4)	-3(3)	-1(3)	-4(3)
C20_1	113(4)	112(4)	111(4)	1(3)	-1(3)	-1(3)
C21_1	95(4)	88(4)	91(3)	-4(2)	6(2)	-5(3)
C22_1	79(3)	74(3)	77(3)	-1(2)	6(2)	-2(2)
C23_1	40(3)	21(2)	40(3)	2(2)	14(2)	-3(2)
C24_1	42(3)	24(2)	46(3)	-4(2)	11(3)	-2(2)
C25_1	60(4)	28(3)	47(3)	-1(2)	4(3)	-12(3)
C26_1	48(3)	21(2)	53(3)	4(2)	7(3)	-5(2)
C27_1	45(3)	37(3)	39(3)	2(2)	8(3)	-2(2)
C28_1	43(3)	30(3)	50(4)	2(2)	8(3)	-3(2)
C29_1	62(3)	10(2)	42(3)	2(2)	21(2)	1(2)
C30_1	75(4)	22(3)	43(3)	-1(2)	13(3)	-3(3)
C31_1	121(6)	38(4)	52(4)	12(3)	23(4)	12(4)
C32_1	126(6)	31(3)	52(4)	9(3)	51(4)	10(4)
C33_1	98(5)	23(3)	64(4)	12(3)	59(3)	5(3)
C34_1	59(4)	17(2)	54(3)	10(2)	31(3)	7(2)
C35_1	25(2)	35(3)	44(3)	7(2)	2(2)	6(2)
C36_1	23(3)	49(3)	51(4)	-12(3)	-5(2)	-5(2)
C37_1	33(3)	51(4)	61(4)	-6(3)	-1(3)	20(3)
C38_1	41(3)	48(4)	44(3)	13(3)	-4(3)	9(3)

Atom	U_{11}	U_{22}	U_{33}	U_{23}	U_{13}	U_{12}
O1_2	31.8(18)	18.5(16)	24.4(16)	10.4(13)	-1.1(14)	-1.0(13)
O2_2	27.5(17)	15.4(15)	18.4(14)	1.8(12)	0.1(12)	-3.1(13)
O3_2	34(2)	34(2)	35(2)	-8.7(16)	-2.0(16)	9.6(16)
O4_2	25.0(17)	21.9(17)	31.9(18)	-4.1(14)	1.4(14)	-2.7(14)
N1_2	24.4(19)	16.8(18)	21.8(19)	4.2(14)	6.2(15)	1.7(15)
C1_2	26(2)	21(2)	15.6(18)	4.8(15)	2.4(15)	0.3(17)
C2_2	28(2)	19(2)	21(2)	0.6(17)	3.1(18)	1.8(17)
C3_2	32(2)	13(2)	28(2)	4.9(17)	3.0(19)	5.3(17)
C4_2	29(2)	15(2)	21(2)	3.1(16)	4.6(17)	1.5(17)
C5_2	24(2)	22(2)	24(2)	-5.1(18)	-0.9(17)	2.2(18)
C6_2	22(2)	18.7(19)	21(2)	3.7(16)	1.6(17)	1.0(16)
C7_2	22(2)	18(2)	30(2)	-4.5(18)	-2.9(18)	0.8(17)
C8_2	24(2)	21(2)	25(2)	-3.5(18)	-0.9(18)	-5.8(18)
C9_2	41(3)	23(2)	24(2)	3.7(19)	7(2)	-2.2(19)
C10_2	35(2)	19(2)	17(2)	2.7(17)	1.4(18)	-2.8(18)
C11_2	36(3)	20(2)	25(2)	-6.2(18)	-4.9(19)	0.7(19)
C12_2	47(3)	24(2)	35(3)	-5(2)	-17(2)	4(2)
C13_2	48(3)	30(3)	55(3)	-17(2)	-28(2)	11(2)
C14_2	56(4)	51(3)	49(4)	-23(3)	-16(3)	22(3)
C15_2	36(3)	56(3)	42(3)	-23(3)	-5(3)	17(3)
C16_2	34(3)	31(3)	30(3)	-8(2)	5(2)	5(2)
C17_2	37(3)	27(3)	34(3)	-2(2)	1(2)	3(2)
C18_2	43(3)	31(3)	33(3)	-3(2)	-1(2)	2(2)
C19_2	58(4)	51(4)	34(3)	-20(3)	-10(3)	8(3)
C20_2	44(4)	54(4)	63(4)	-19(3)	-18(3)	2(3)
C21_2	45(4)	38(3)	69(4)	-15(3)	-7(3)	-2(3)
C22_2	50(4)	32(3)	42(3)	2(2)	-1(3)	-4(2)
C23_2	24(2)	38(3)	37(3)	-4(2)	-2(2)	-2(2)
C24_2	28(3)	68(4)	38(3)	-12(3)	3(2)	-15(3)
C25_2	25(3)	98(6)	53(4)	-19(4)	8(3)	-12(3)
C26_2	34(3)	67(5)	74(4)	-6(4)	9(3)	9(3)
C27_2	32(3)	60(4)	62(4)	-8(3)	-1(3)	16(3)
C28_2	31(3)	36(3)	46(3)	0(3)	-5(2)	3(2)
C29_2	21(2)	21(2)	25(2)	-2.5(17)	3.9(18)	-3.5(17)
C30_2	36(3)	42(3)	24(2)	-6(2)	0(2)	-7(2)
C31_2	37(3)	40(3)	29(3)	-10(2)	7(2)	-12(2)
C32_2	50(3)	29(3)	39(3)	-16(2)	13(3)	-10(2)
C33_2	42(3)	33(3)	45(3)	-12(2)	6(3)	3(3)
C34_2	37(3)	31(3)	34(3)	-1(2)	4(2)	4(2)
C35_2	31(2)	27(2)	20(2)	8.2(18)	1.0(19)	5.7(19)
C36_2	24(2)	46(3)	33(3)	18(2)	2(2)	6(2)
C37_2	48(3)	29(3)	26(3)	6(2)	7(2)	-1(2)
C38_2	48(3)	24(3)	20(2)	0.6(19)	3(2)	-3(2)
O1_3	21.7(16)	22.4(17)	19.3(15)	1.2(13)	1.0(12)	-0.1(13)
O2_3	19.8(16)	24.6(17)	20.8(15)	4.2(13)	-2.0(13)	4.4(13)
O3_3	27.7(18)	24.9(17)	41(2)	10.9(15)	-3.7(16)	-0.4(14)
O4_3	24.0(17)	32(2)	36(2)	12.3(16)	-3.7(15)	-5.4(15)
N1_3	20.6(18)	19.5(18)	20.2(19)	6.1(15)	-3.0(15)	0.6(14)
C1_3	28(2)	18(2)	21.9(19)	7.2(17)	-1.8(17)	-3.4(17)
C2_3	29(2)	18(2)	14.6(19)	7.0(16)	-4.7(17)	-1.2(18)
C3_3	22(2)	22(2)	32(3)	-1.1(19)	-2.4(19)	2.1(17)
C4_3	29(2)	30(2)	20(2)	-1.2(18)	-7.5(19)	3.2(19)
C5_3	28(2)	26(2)	22(2)	4.1(18)	-0.4(19)	6.6(18)
C6_3	22(2)	22(2)	38(3)	5(2)	-2(2)	-3.7(17)
C7_3	23(2)	26(2)	23(2)	6.1(18)	-5.9(18)	-7.2(17)
C8_3	27(2)	25(2)	25(2)	12.3(19)	-1.6(19)	1.0(18)
C9_3	22(2)	28(2)	24(2)	4.2(19)	3.0(18)	2.2(17)
C10_3	26(2)	23(2)	23(2)	0.9(17)	-4.2(18)	0.2(17)
C11_3	27(2)	25(2)	28(2)	5.7(19)	-1.3(19)	4.8(18)
C12_3	38(3)	35(3)	44(3)	2(2)	14(3)	2(2)
C13_3	41(3)	53(3)	40(3)	-5(3)	5(3)	-11(3)
C14_3	40(3)	41(3)	42(3)	7(2)	-10(2)	-8(3)
C15_3	33(3)	36(3)	60(4)	1(3)	-9(2)	-4(2)

Atom	U_{11}	U_{22}	U_{33}	U_{23}	U_{13}	U_{12}
C16_3	39(3)	34(3)	42(3)	-7(2)	0(3)	-5(2)
C17_3	30(3)	36(3)	37(3)	11(2)	-8(2)	-1(2)
C18_3	29(3)	53(4)	36(3)	10(2)	-14(2)	-2(3)
C19_3	30(3)	63(4)	60(4)	33(3)	-16(3)	-12(3)
C20_3	28(3)	54(4)	90(5)	35(3)	-15(3)	-2(3)
C21_3	30(3)	60(4)	79(4)	35(3)	0(3)	12(3)
C22_3	35(3)	59(4)	50(4)	14(3)	0(3)	14(3)
C23_3	28(3)	26(2)	33(3)	9(2)	-4(2)	9(2)
C24_3	54(4)	28(3)	44(3)	5(2)	12(3)	15(3)
C25_3	35(3)	33(3)	67(4)	15(3)	0(3)	9(2)
C26_3	55(4)	36(3)	52(3)	17(3)	-14(3)	-8(3)
C27_3	64(4)	42(3)	47(4)	20(3)	-8(3)	-9(3)
C28_3	50(4)	38(3)	29(3)	7(2)	1(2)	-6(3)
C29_3	28(2)	24(2)	26(2)	4.6(19)	7(2)	-5.8(18)
C30_3	41(3)	21(2)	20(2)	7.2(17)	13(2)	1.6(19)
C31_3	41(3)	20(2)	27(2)	4.7(18)	3(2)	1(2)
C32_3	41(3)	23(2)	49(3)	5(2)	10(2)	-4(2)
C33_3	23(2)	25(2)	38(3)	7(2)	8(2)	-1.9(19)
C34_3	27(2)	20(2)	30(3)	-0.4(19)	3.8(19)	-2.1(18)
C35_3	27(2)	21(2)	19(2)	-0.8(17)	1.7(18)	0.4(18)
C36_3	39(3)	31(3)	23(2)	4(2)	-2(2)	13(2)
C37_3	28(3)	35(3)	45(3)	-1(3)	7(2)	0(2)
C38_3	35(3)	26(3)	25(2)	-6(2)	0(2)	-2(2)
O1_4	22.1(16)	21.4(16)	19.8(14)	4.0(12)	3.0(13)	-1.8(13)
O2_4	27.9(18)	23.4(17)	15.4(14)	-1.8(12)	1.9(13)	3.1(13)
O3_4	30.5(19)	29.1(19)	25.8(18)	3.0(14)	0.9(14)	6.8(15)
O4_4	32(2)	27.8(19)	27.4(18)	7.7(14)	-0.2(15)	3.9(15)
N1_4	27(2)	19.7(19)	22.6(19)	2.2(15)	4.6(16)	3.1(15)
C1_4	16(2)	23(2)	20.6(19)	2.5(15)	4.1(15)	2.4(16)
C2_4	29(2)	19(2)	17.3(19)	-0.6(16)	-1.7(18)	-2.0(18)
C3_4	23(2)	21(2)	22(2)	-1.1(17)	-1.4(16)	-1.0(17)
C4_4	19(2)	24(2)	22(2)	2.8(17)	0.2(17)	3.3(17)
C5_4	18(2)	19(2)	24(2)	2.4(17)	-2.4(17)	-0.6(17)
C6_4	25(2)	21(2)	24(2)	1.5(17)	-0.6(18)	2.6(18)
C7_4	20(2)	23(2)	23(2)	5.7(18)	5.0(17)	-2.0(17)
C8_4	17(2)	27(2)	23(2)	7.5(18)	1.2(17)	-1.0(18)
C9_4	17(2)	18(2)	21(2)	6.0(16)	-3.6(16)	-3.3(16)
C10_4	23(2)	14(2)	23(2)	1.9(16)	-4.7(16)	-3.8(16)
C11_4	26(2)	22(2)	25(2)	5.1(18)	-2.0(18)	0.5(18)
C12_4	41(3)	26(2)	32(3)	4(2)	6(2)	7(2)
C13_4	48(3)	20(3)	43(3)	0(2)	2(2)	6(2)
C14_4	43(3)	43(3)	36(3)	10(2)	-1(2)	18(3)
C15_4	39(3)	45(3)	31(3)	4(2)	-6(2)	6(2)
C16_4	33(3)	33(3)	23(2)	4.5(19)	-8(2)	1(2)
C17_4	27(3)	25(2)	27(2)	8.5(18)	3(2)	-3(2)
C18_4	33(3)	33(3)	32(2)	6(2)	-2(2)	-1(2)
C19_4	38(3)	50(3)	27(3)	6(2)	-7(2)	0(3)
C20_4	44(3)	50(3)	33(3)	22(2)	7(3)	-5(3)
C21_4	39(3)	38(3)	41(3)	20(2)	-6(2)	-4(3)
C22_4	35(3)	30(3)	39(3)	15(2)	1(2)	-6(2)
C23_4	27(2)	34(3)	21(2)	12.0(19)	3.2(19)	2.8(19)
C24_4	30(3)	36(3)	49(3)	15(3)	7(2)	-2(2)
C25_4	23(3)	70(4)	63(4)	24(3)	7(3)	8(3)
C26_4	45(3)	78(5)	47(4)	24(3)	22(3)	30(3)
C27_4	53(4)	66(5)	48(4)	0(3)	9(3)	32(3)
C28_4	35(3)	49(3)	26(3)	0(2)	1(2)	12(3)
C29_4	24(2)	21(2)	20(2)	10.8(17)	0.9(17)	-4.0(17)
C30_4	36(3)	28(3)	39(3)	1(2)	-3(2)	-5(2)
C31_4	32(3)	49(3)	33(3)	13(2)	-7(2)	-10(2)
C32_4	33(3)	46(3)	44(3)	20(2)	-1(2)	10(2)
C33_4	46(3)	34(3)	46(3)	7(2)	0(2)	16(3)
C34_4	31(3)	31(3)	33(3)	3(2)	1(2)	6(2)
C35_4	37(3)	24(2)	22(2)	-5.6(18)	-4(2)	-4(2)

Atom	U_{11}	U_{22}	U_{33}	U_{23}	U_{13}	U_{12}
C36_4	69(4)	22(2)	29(3)	-9(2)	10(3)	0(3)
C37_4	48(3)	33(3)	28(3)	-1(2)	-8(2)	-3(2)
C38_4	44(3)	40(3)	42(3)	0(3)	-22(3)	-6(3)
Cl1_6	98.8(19)	69.2(14)	90.5(17)	-34.1(13)	-19.2(15)	15.8(13)
Cl2_6	85.5(16)	60.5(12)	76.8(14)	12.9(10)	1.3(12)	-0.5(12)
C1_6	60(5)	66(5)	58(4)	-14(4)	0(4)	-17(4)
Cl3_8	58.4(9)	40.1(8)	50.0(8)	-18.9(6)	-8.4(8)	2.2(8)
N2_8	25.8(18)	36.3(16)	22.2(14)	-3.6(11)	-3.2(13)	-12.4(13)
C39_8	33(2)	46.6(18)	29.0(14)	-11.8(12)	-3.4(14)	-9.9(16)
C40_8	50(3)	58(2)	30.3(15)	-10.6(15)	-8.7(17)	-9(2)
C41_8	56(3)	64(2)	32.5(18)	-5.1(16)	-6(2)	-3(2)
C42_8	31(3)	55(2)	27.3(16)	2.1(14)	-4.3(15)	-10.0(19)
C43_8	27(2)	40.0(18)	26.9(16)	0.5(13)	-2.5(15)	-10.3(15)
Cl3_9	84.4(13)	37.5(8)	36.3(7)	7.9(6)	-2.8(8)	0.9(8)
N2_9	53(3)	45(2)	44.2(16)	-7.1(14)	0.0(16)	-14.1(19)
C39_9	54(3)	30(2)	40.5(18)	1.3(15)	0.4(14)	-13.3(17)
C40_9	61(3)	35(2)	45(2)	1.4(18)	-1.6(19)	-7(2)
C41_9	67(4)	51(3)	47(2)	6.9(17)	-2.2(18)	-5(2)
C42_9	66(3)	60(3)	49(2)	0(2)	-2(2)	-10(3)
C43_9	54(3)	49(3)	43.9(17)	-5.8(18)	-2.6(18)	-21(2)
Cl3_10	96(4)	195(8)	43(3)	1(4)	-3(3)	-71(5)
N2_10	61(2)	67(2)	54.5(18)	-2.9(15)	-1.1(17)	-6.9(18)
C39_10	69(3)	68(2)	54.5(18)	-3.3(15)	-0.9(16)	-6.6(19)
C40_10	65(3)	63(4)	56(2)	-4.5(19)	-0.8(17)	-2(2)
C41_10	65(3)	67(4)	63(3)	0(2)	-1(2)	-2(2)
C42_10	67(6)	112(17)	63(3)	1(3)	-2(2)	-24(9)
C43_10	60(4)	72(10)	63(3)	4(4)	-3(3)	-7(6)
Cl3_13	91(3)	63(2)	57.0(19)	-10.9(14)	-7.2(18)	1.7(18)
N2_13	61(2)	67(2)	54.5(18)	-2.9(15)	-1.1(17)	-6.9(18)
C39_13	69(3)	68(2)	54.5(18)	-3.3(15)	-0.9(16)	-6.6(19)
C40_13	144(15)	90(6)	63(3)	-13(3)	-25(5)	27(7)
C41_13	120(13)	87(6)	59(4)	-11(3)	-21(5)	18(7)
C42_13	103(10)	77(6)	56(4)	-5(3)	-14(5)	7(6)
C43_13	58(6)	67(3)	51(3)	-1(2)	2(4)	-7(3)

Table 3: Bond Lengths in Å for **JCS-Pyridine-TPPTTL-CoXtal-xtal2.**

Atom	Atom	Length/Å
Rh1	Rh2	2.3998(5)
Rh1	O1_1	2.057(3)
Rh1	O1_2	2.040(3)
Rh1	O1_3	2.032(3)
Rh1	O1_4	2.052(3)
Rh1	N2_10	2.383(9)
Rh1	N2_13	2.278(7)
Rh2	O2_1	2.048(3)
Rh2	O2_2	2.053(3)
Rh2	O2_3	2.074(3)
Rh2	O2_4	2.041(3)
Rh2	N2_8	2.272(4)
O1_1	C1_1	1.250(3)
O2_1	C1_1	1.271(3)
O3_1	C3_1	1.216(4)
O4_1	C6_1	1.211(4)
N1_1	C2_1	1.451(4)
N1_1	C3_1	1.399(4)
N1_1	C6_1	1.399(4)
C1_1	C2_1	1.535(4)
C2_1	C35_1	1.559(4)
C3_1	C4_1	1.487(4)
C4_1	C5_1	1.394(4)
C4_1	C10_1	1.390(4)
C5_1	C6_1	1.501(4)

Atom	Atom	Length/Å
C5_1	C7_1	1.393(4)
C7_1	C8_1	1.419(4)
C7_1	C29_1	1.493(4)
C8_1	C9_1	1.406(4)
C8_1	C23_1	1.492(4)
C9_1	C10_1	1.426(4)
C9_1	C17_1	1.500(4)
C10_1	C11_1	1.492(4)
C11_1	C12_1	1.372(4)
C11_1	C16_1	1.410(4)
C12_1	C13_1	1.390(5)
C13_1	C14_1	1.365(5)
C14_1	C15_1	1.361(5)
C15_1	C16_1	1.398(4)
C17_1	C18_1	1.385(5)
C17_1	C22_1	1.395(5)
C18_1	C19_1	1.408(5)
C19_1	C20_1	1.356(6)
C20_1	C21_1	1.376(6)
C21_1	C22_1	1.392(5)
C23_1	C24_1	1.392(5)
C23_1	C28_1	1.395(5)
C24_1	C25_1	1.382(5)
C25_1	C26_1	1.359(6)
C26_1	C27_1	1.376(6)

Atom	Atom	Length/Å
C27_1	C28_1	1.402(5)
C29_1	C30_1	1.387(4)
C29_1	C34_1	1.380(4)
C30_1	C31_1	1.381(5)
C31_1	C32_1	1.382(5)
C32_1	C33_1	1.360(5)
C33_1	C34_1	1.395(4)
C35_1	C36_1	1.531(4)
C35_1	C37_1	1.534(4)
C35_1	C38_1	1.530(4)
O1_2	C1_2	1.251(3)
O2_2	C1_2	1.271(3)
O3_2	C3_2	1.215(4)
O4_2	C6_2	1.210(3)
N1_2	C2_2	1.450(3)
N1_2	C3_2	1.399(4)
N1_2	C6_2	1.396(3)
C1_2	C2_2	1.534(4)
C2_2	C35_2	1.560(4)
C3_2	C4_2	1.486(4)
C4_2	C5_2	1.393(4)
C4_2	C10_2	1.389(4)
C5_2	C6_2	1.501(4)
C5_2	C7_2	1.395(4)
C7_2	C8_2	1.417(4)
C7_2	C29_2	1.492(4)
C8_2	C9_2	1.406(4)
C8_2	C23_2	1.491(4)
C9_2	C10_2	1.426(4)
C9_2	C17_2	1.499(4)
C10_2	C11_2	1.493(4)
C11_2	C12_2	1.372(4)
C11_2	C16_2	1.410(4)
C12_2	C13_2	1.390(5)
C13_2	C14_2	1.364(5)
C14_2	C15_2	1.361(5)
C15_2	C16_2	1.398(4)
C17_2	C18_2	1.385(5)
C17_2	C22_2	1.395(5)
C18_2	C19_2	1.407(5)
C19_2	C20_2	1.356(6)
C20_2	C21_2	1.376(6)
C21_2	C22_2	1.392(5)
C23_2	C24_2	1.391(5)
C23_2	C28_2	1.395(5)
C24_2	C25_2	1.381(5)
C25_2	C26_2	1.359(6)
C26_2	C27_2	1.377(6)
C27_2	C28_2	1.403(5)
C29_2	C30_2	1.387(4)
C29_2	C34_2	1.380(4)
C30_2	C31_2	1.381(5)
C31_2	C32_2	1.382(5)
C32_2	C33_2	1.360(5)
C33_2	C34_2	1.394(4)
C35_2	C36_2	1.532(4)
C35_2	C37_2	1.533(4)
C35_2	C38_2	1.530(4)
O1_3	C1_3	1.251(3)
O2_3	C1_3	1.271(3)
O3_3	C3_3	1.216(4)
O4_3	C6_3	1.212(4)
N1_3	C2_3	1.452(3)
N1_3	C3_3	1.400(4)
N1_3	C6_3	1.397(3)
C1_3	C2_3	1.534(4)
C2_3	C35_3	1.561(4)
C3_3	C4_3	1.486(4)
C4_3	C5_3	1.393(4)
C4_3	C10_3	1.390(4)
C5_3	C6_3	1.502(4)
C5_3	C7_3	1.394(4)
C7_3	C8_3	1.420(4)
C7_3	C29_3	1.494(4)
C8_3	C9_3	1.406(4)
C8_3	C23_3	1.492(4)
C9_3	C10_3	1.427(4)
C9_3	C17_3	1.500(4)
C10_3	C11_3	1.492(4)
C11_3	C12_3	1.371(4)
C11_3	C16_3	1.410(4)
C12_3	C13_3	1.391(5)
C13_3	C14_3	1.364(5)
C14_3	C15_3	1.361(5)
C15_3	C16_3	1.398(4)
C17_3	C18_3	1.386(5)
C17_3	C22_3	1.395(5)
C18_3	C19_3	1.408(5)
C19_3	C20_3	1.356(6)
C20_3	C21_3	1.376(6)
C21_3	C22_3	1.392(5)
C23_3	C24_3	1.392(4)
C23_3	C28_3	1.396(5)
C24_3	C25_3	1.380(5)
C25_3	C26_3	1.359(6)
C26_3	C27_3	1.377(6)
C27_3	C28_3	1.402(5)
C29_3	C30_3	1.387(4)
C29_3	C34_3	1.380(4)
C30_3	C31_3	1.380(5)
C31_3	C32_3	1.382(5)
C32_3	C33_3	1.361(5)
C33_3	C34_3	1.395(4)
C35_3	C36_3	1.532(4)
C35_3	C37_3	1.534(4)
C35_3	C38_3	1.530(4)
O1_4	C1_4	1.251(3)
O2_4	C1_4	1.271(3)
O3_4	C3_4	1.217(4)
O4_4	C6_4	1.213(4)
N1_4	C2_4	1.453(3)
N1_4	C3_4	1.399(4)
N1_4	C6_4	1.398(3)
C1_4	C2_4	1.537(4)
C2_4	C35_4	1.561(4)
C3_4	C4_4	1.488(4)
C4_4	C5_4	1.394(4)
C4_4	C10_4	1.389(4)
C5_4	C6_4	1.501(4)
C5_4	C7_4	1.395(4)
C7_4	C8_4	1.418(4)
C7_4	C29_4	1.492(4)
C8_4	C9_4	1.406(4)
C8_4	C23_4	1.491(4)
C9_4	C10_4	1.428(4)
C9_4	C17_4	1.500(4)

Atom	Atom	Length/Å
C10_4	C11_4	1.493(4)
C11_4	C12_4	1.372(4)
C11_4	C16_4	1.411(4)
C12_4	C13_4	1.391(5)
C13_4	C14_4	1.364(5)
C14_4	C15_4	1.362(5)
C15_4	C16_4	1.398(4)
C17_4	C18_4	1.385(5)
C17_4	C22_4	1.396(5)
C18_4	C19_4	1.408(5)
C19_4	C20_4	1.356(6)
C20_4	C21_4	1.376(6)
C21_4	C22_4	1.391(5)
C23_4	C24_4	1.391(5)
C23_4	C28_4	1.396(5)
C24_4	C25_4	1.381(5)
C25_4	C26_4	1.359(6)
C26_4	C27_4	1.377(6)
C27_4	C28_4	1.402(5)
C29_4	C30_4	1.387(4)
C29_4	C34_4	1.379(4)
C30_4	C31_4	1.381(5)
C31_4	C32_4	1.381(5)
C32_4	C33_4	1.361(5)
C33_4	C34_4	1.395(4)
C35_4	C36_4	1.531(4)
C35_4	C37_4	1.535(4)
C35_4	C38_4	1.529(4)
Cl1_6	C1_6	1.757(8)

Atom	Atom	Length/Å
Cl2_6	C1_6	1.759(9)
Cl3_8	C39_8	1.733(5)
N2_8	C39_8	1.315(5)
N2_8	C43_8	1.363(6)
C39_8	C40_8	1.364(6)
C40_8	C41_8	1.381(7)
C41_8	C42_8	1.366(7)
C42_8	C43_8	1.409(6)
Cl3_9	C39_9	1.735(5)
N2_9	C39_9	1.316(5)
N2_9	C43_9	1.364(6)
C39_9	C40_9	1.364(6)
C40_9	C41_9	1.381(7)
C41_9	C42_9	1.366(7)
C42_9	C43_9	1.409(6)
Cl3_10	C39_10	1.733(5)
N2_10	C39_10	1.315(6)
N2_10	C43_10	1.364(6)
C39_10	C40_10	1.364(6)
C40_10	C41_10	1.382(7)
C41_10	C42_10	1.367(7)
C42_10	C43_10	1.410(7)
Cl3_13	C39_13	1.733(5)
N2_13	C39_13	1.315(6)
N2_13	C43_13	1.363(6)
C39_13	C40_13	1.364(6)
C40_13	C41_13	1.382(7)
C41_13	C42_13	1.366(7)
C42_13	C43_13	1.410(6)

Table 4: Bond Angles in ° for **JCS-Pyridine-TPPTTL-CoXtal-xtal2.**

Atom	Atom	Atom	Angle/°
O1_1	Rh1	Rh2	89.47(7)
O1_1	Rh1	N2_10	85.2(4)
O1_1	Rh1	N2_13	84.5(2)
O1_2	Rh1	Rh2	87.95(8)
O1_2	Rh1	O1_1	89.20(15)
O1_2	Rh1	O1_4	175.56(12)
O1_2	Rh1	N2_10	101.6(4)
O1_2	Rh1	N2_13	80.6(3)
O1_3	Rh1	Rh2	87.34(8)
O1_3	Rh1	O1_1	176.69(12)
O1_3	Rh1	O1_2	89.78(15)
O1_3	Rh1	O1_4	89.54(14)
O1_3	Rh1	N2_10	98.1(4)
O1_3	Rh1	N2_13	98.5(2)
O1_4	Rh1	Rh2	87.64(8)
O1_4	Rh1	O1_1	91.24(14)
O1_4	Rh1	N2_10	82.8(4)
O1_4	Rh1	N2_13	103.8(3)
N2_10	Rh1	Rh2	168.9(2)
N2_13	Rh1	Rh2	167.11(18)
O2_1	Rh2	Rh1	86.60(8)
O2_1	Rh2	O2_2	90.71(14)
O2_1	Rh2	O2_3	174.62(12)
O2_1	Rh2	N2_8	92.69(13)
O2_2	Rh2	Rh1	88.11(8)
O2_2	Rh2	O2_3	86.80(14)
O2_2	Rh2	N2_8	96.73(13)
O2_3	Rh2	Rh1	88.55(8)
O2_3	Rh2	N2_8	92.33(13)

Atom	Atom	Atom	Angle/°
O2_4	Rh2	Rh1	88.33(8)
O2_4	Rh2	O2_1	90.86(14)
O2_4	Rh2	O2_2	176.02(12)
O2_4	Rh2	O2_3	91.33(14)
O2_4	Rh2	N2_8	86.85(13)
N2_8	Rh2	Rh1	175.12(11)
C1_1	O1_1	Rh1	116.7(2)
C1_1	O2_1	Rh2	119.8(2)
C3_1	N1_1	C2_1	122.5(3)
C6_1	N1_1	C2_1	125.7(3)
C6_1	N1_1	C3_1	111.3(2)
O1_1	C1_1	O2_1	127.4(3)
O1_1	C1_1	C2_1	119.1(3)
O2_1	C1_1	C2_1	113.5(3)
N1_1	C2_1	C1_1	109.1(3)
N1_1	C2_1	C35_1	114.0(3)
C1_1	C2_1	C35_1	117.5(3)
O3_1	C3_1	N1_1	124.0(3)
O3_1	C3_1	C4_1	129.2(3)
N1_1	C3_1	C4_1	106.7(2)
C5_1	C4_1	C3_1	107.6(3)
C10_1	C4_1	C3_1	130.0(3)
C10_1	C4_1	C5_1	122.3(3)
C4_1	C5_1	C6_1	107.7(3)
C7_1	C5_1	C4_1	122.2(3)
C7_1	C5_1	C6_1	130.1(3)
O4_1	C6_1	N1_1	125.1(3)
O4_1	C6_1	C5_1	129.1(3)
N1_1	C6_1	C5_1	105.8(2)

Atom	Atom	Atom	Angle/°
C5_1	C7_1	C8_1	116.2(3)
C5_1	C7_1	C29_1	123.1(3)
C8_1	C7_1	C29_1	120.7(3)
C7_1	C8_1	C23_1	119.1(3)
C9_1	C8_1	C7_1	121.9(3)
C9_1	C8_1	C23_1	118.9(3)
C8_1	C9_1	C10_1	120.5(3)
C8_1	C9_1	C17_1	120.0(3)
C10_1	C9_1	C17_1	119.3(3)
C4_1	C10_1	C9_1	116.7(3)
C4_1	C10_1	C11_1	122.3(3)
C9_1	C10_1	C11_1	120.9(3)
C12_1	C11_1	C10_1	121.8(3)
C12_1	C11_1	C16_1	119.1(3)
C16_1	C11_1	C10_1	119.0(3)
C11_1	C12_1	C13_1	120.3(4)
C14_1	C13_1	C12_1	120.8(4)
C15_1	C14_1	C13_1	119.9(4)
C14_1	C15_1	C16_1	120.8(4)
C15_1	C16_1	C11_1	119.0(3)
C18_1	C17_1	C9_1	121.1(4)
C18_1	C17_1	C22_1	118.8(4)
C22_1	C17_1	C9_1	120.1(4)
C17_1	C18_1	C19_1	120.5(4)
C20_1	C19_1	C18_1	119.4(5)
C19_1	C20_1	C21_1	121.2(4)
C20_1	C21_1	C22_1	119.9(5)
C21_1	C22_1	C17_1	120.0(4)
C24_1	C23_1	C8_1	121.4(3)
C24_1	C23_1	C28_1	118.9(3)
C28_1	C23_1	C8_1	119.7(3)
C25_1	C24_1	C23_1	120.6(4)
C26_1	C25_1	C24_1	120.7(4)
C25_1	C26_1	C27_1	119.9(4)
C26_1	C27_1	C28_1	120.8(4)
C23_1	C28_1	C27_1	119.1(4)
C30_1	C29_1	C7_1	119.9(3)
C34_1	C29_1	C7_1	121.1(3)
C34_1	C29_1	C30_1	118.6(3)
C31_1	C30_1	C29_1	121.4(4)
C30_1	C31_1	C32_1	118.5(4)
C33_1	C32_1	C31_1	121.7(4)
C32_1	C33_1	C34_1	119.2(4)
C29_1	C34_1	C33_1	120.6(4)
C36_1	C35_1	C2_1	108.7(3)
C36_1	C35_1	C37_1	108.0(3)
C36_1	C35_1	C38_1	111.1(3)
C37_1	C35_1	C2_1	106.8(3)
C38_1	C35_1	C2_1	113.3(3)
C38_1	C35_1	C37_1	108.7(3)
C1_2	O1_2	Rh1	118.8(2)
C1_2	O2_2	Rh2	117.6(2)
C3_2	N1_2	C2_2	122.2(3)
C6_2	N1_2	C2_2	126.7(3)
C6_2	N1_2	C3_2	111.0(2)
O1_2	C1_2	O2_2	127.4(3)
O1_2	C1_2	C2_2	118.1(3)
O2_2	C1_2	C2_2	114.3(3)
N1_2	C2_2	C1_2	109.6(3)
N1_2	C2_2	C35_2	114.3(3)
C1_2	C2_2	C35_2	117.6(3)
O3_2	C3_2	N1_2	123.6(3)
O3_2	C3_2	C4_2	129.5(3)

Atom	Atom	Atom	Angle/°
N1_2	C3_2	C4_2	106.9(2)
C5_2	C4_2	C3_2	107.5(3)
C10_2	C4_2	C3_2	130.2(3)
C10_2	C4_2	C5_2	122.2(3)
C4_2	C5_2	C6_2	107.6(2)
C4_2	C5_2	C7_2	122.4(3)
C7_2	C5_2	C6_2	129.9(3)
O4_2	C6_2	N1_2	124.8(3)
O4_2	C6_2	C5_2	129.3(3)
N1_2	C6_2	C5_2	105.9(2)
C5_2	C7_2	C8_2	116.2(3)
C5_2	C7_2	C29_2	122.1(3)
C8_2	C7_2	C29_2	121.6(3)
C7_2	C8_2	C23_2	119.2(3)
C9_2	C8_2	C7_2	121.7(3)
C9_2	C8_2	C23_2	119.1(3)
C8_2	C9_2	C10_2	120.9(3)
C8_2	C9_2	C17_2	120.1(3)
C10_2	C9_2	C17_2	119.0(3)
C4_2	C10_2	C9_2	116.6(3)
C4_2	C10_2	C11_2	122.3(3)
C9_2	C10_2	C11_2	121.1(3)
C12_2	C11_2	C10_2	121.7(3)
C12_2	C11_2	C16_2	119.1(3)
C16_2	C11_2	C10_2	119.1(3)
C11_2	C12_2	C13_2	120.3(4)
C14_2	C13_2	C12_2	120.8(4)
C15_2	C14_2	C13_2	119.9(4)
C14_2	C15_2	C16_2	120.9(4)
C15_2	C16_2	C11_2	119.0(3)
C18_2	C17_2	C9_2	120.9(3)
C18_2	C17_2	C22_2	118.8(3)
C22_2	C17_2	C9_2	120.2(3)
C17_2	C18_2	C19_2	120.5(4)
C20_2	C19_2	C18_2	119.4(4)
C19_2	C20_2	C21_2	121.1(4)
C20_2	C21_2	C22_2	119.9(4)
C21_2	C22_2	C17_2	120.0(4)
C24_2	C23_2	C8_2	121.4(3)
C24_2	C23_2	C28_2	118.6(3)
C28_2	C23_2	C8_2	119.8(3)
C25_2	C24_2	C23_2	121.0(4)
C26_2	C25_2	C24_2	120.6(4)
C25_2	C26_2	C27_2	119.7(4)
C26_2	C27_2	C28_2	120.9(4)
C23_2	C28_2	C27_2	119.1(4)
C30_2	C29_2	C7_2	119.9(3)
C34_2	C29_2	C7_2	121.4(3)
C34_2	C29_2	C30_2	118.7(3)
C31_2	C30_2	C29_2	121.2(3)
C30_2	C31_2	C32_2	118.6(4)
C33_2	C32_2	C31_2	121.7(3)
C32_2	C33_2	C34_2	119.1(4)
C29_2	C34_2	C33_2	120.7(3)
C36_2	C35_2	C2_2	108.6(3)
C36_2	C35_2	C37_2	108.1(3)
C37_2	C35_2	C2_2	107.1(3)
C38_2	C35_2	C2_2	113.4(3)
C38_2	C35_2	C36_2	110.8(3)
C38_2	C35_2	C37_2	108.7(3)
C1_3	O1_3	Rh1	120.4(2)
C1_3	O2_3	Rh2	116.5(2)
C3_3	N1_3	C2_3	121.6(3)

Atom	Atom	Atom	Angle/°
C6_3	N1_3	C2_3	127.4(3)
C6_3	N1_3	C3_3	111.0(2)
O1_3	C1_3	O2_3	126.7(3)
O1_3	C1_3	C2_3	118.1(3)
O2_3	C1_3	C2_3	114.9(3)
N1_3	C2_3	C1_3	110.2(3)
N1_3	C2_3	C35_3	114.0(3)
C1_3	C2_3	C35_3	117.1(3)
O3_3	C3_3	N1_3	124.1(3)
O3_3	C3_3	C4_3	129.0(3)
N1_3	C3_3	C4_3	106.8(2)
C5_3	C4_3	C3_3	107.9(3)
C10_3	C4_3	C3_3	129.4(3)
C10_3	C4_3	C5_3	122.6(3)
C4_3	C5_3	C6_3	107.6(2)
C4_3	C5_3	C7_3	121.8(3)
C7_3	C5_3	C6_3	130.4(3)
O4_3	C6_3	N1_3	124.6(3)
O4_3	C6_3	C5_3	129.0(3)
N1_3	C6_3	C5_3	106.4(2)
C5_3	C7_3	C8_3	116.6(3)
C5_3	C7_3	C29_3	122.7(3)
C8_3	C7_3	C29_3	120.7(3)
C7_3	C8_3	C23_3	119.0(3)
C9_3	C8_3	C7_3	121.8(3)
C9_3	C8_3	C23_3	119.2(3)
C8_3	C9_3	C10_3	120.4(3)
C8_3	C9_3	C17_3	120.7(3)
C10_3	C9_3	C17_3	118.5(3)
C4_3	C10_3	C9_3	116.8(3)
C4_3	C10_3	C11_3	121.8(3)
C9_3	C10_3	C11_3	121.3(3)
C12_3	C11_3	C10_3	121.8(3)
C12_3	C11_3	C16_3	118.9(3)
C16_3	C11_3	C10_3	119.1(3)
C11_3	C12_3	C13_3	120.2(4)
C14_3	C13_3	C12_3	121.0(4)
C15_3	C14_3	C13_3	119.8(4)
C14_3	C15_3	C16_3	120.8(4)
C15_3	C16_3	C11_3	119.2(3)
C18_3	C17_3	C9_3	120.9(3)
C18_3	C17_3	C22_3	118.9(3)
C22_3	C17_3	C9_3	120.2(3)
C17_3	C18_3	C19_3	120.5(4)
C20_3	C19_3	C18_3	119.4(4)
C19_3	C20_3	C21_3	121.2(4)
C20_3	C21_3	C22_3	120.0(4)
C21_3	C22_3	C17_3	120.0(4)
C24_3	C23_3	C8_3	121.8(3)
C24_3	C23_3	C28_3	118.5(3)
C28_3	C23_3	C8_3	119.4(3)
C25_3	C24_3	C23_3	120.8(4)
C26_3	C25_3	C24_3	120.8(4)
C25_3	C26_3	C27_3	119.6(4)
C26_3	C27_3	C28_3	120.9(4)
C23_3	C28_3	C27_3	119.3(4)
C30_3	C29_3	C7_3	119.5(3)
C34_3	C29_3	C7_3	121.1(3)
C34_3	C29_3	C30_3	118.5(3)
C31_3	C30_3	C29_3	121.2(3)
C30_3	C31_3	C32_3	118.6(3)
C33_3	C32_3	C31_3	121.6(3)
C32_3	C33_3	C34_3	119.0(3)
C29_3	C34_3	C33_3	120.9(3)
C36_3	C35_3	C2_3	109.3(3)
C36_3	C35_3	C37_3	107.6(3)
C36_3	C35_3	C38_3	110.9(3)
C37_3	C35_3	C2_3	107.3(3)
C38_3	C35_3	C2_3	112.6(3)
C38_3	C35_3	C37_3	108.9(3)
C1_4	O1_4	Rh1	118.8(2)
C1_4	O2_4	Rh2	118.1(2)
C3_4	N1_4	C2_4	123.4(3)
C6_4	N1_4	C2_4	125.2(3)
C6_4	N1_4	C3_4	111.3(2)
O1_4	C1_4	O2_4	126.9(3)
O1_4	C1_4	C2_4	118.7(3)
O2_4	C1_4	C2_4	114.2(3)
N1_4	C2_4	C1_4	109.0(3)
N1_4	C2_4	C35_4	113.5(3)
C1_4	C2_4	C35_4	116.8(3)
O3_4	C3_4	N1_4	124.1(3)
O3_4	C3_4	C4_4	129.1(3)
N1_4	C3_4	C4_4	106.8(2)
C5_4	C4_4	C3_4	107.6(2)
C10_4	C4_4	C3_4	130.6(3)
C10_4	C4_4	C5_4	121.7(3)
C4_4	C5_4	C6_4	108.1(2)
C4_4	C5_4	C7_4	122.8(3)
C7_4	C5_4	C6_4	129.1(3)
O4_4	C6_4	N1_4	124.5(3)
O4_4	C6_4	C5_4	129.4(3)
N1_4	C6_4	C5_4	105.9(2)
C5_4	C7_4	C8_4	116.2(3)
C5_4	C7_4	C29_4	122.1(3)
C8_4	C7_4	C29_4	121.4(3)
C7_4	C8_4	C23_4	119.5(3)
C9_4	C8_4	C7_4	121.5(3)
C9_4	C8_4	C23_4	118.8(3)
C8_4	C9_4	C10_4	120.9(3)
C8_4	C9_4	C17_4	120.0(3)
C10_4	C9_4	C17_4	118.9(3)
C4_4	C10_4	C9_4	116.9(3)
C4_4	C10_4	C11_4	123.4(3)
C9_4	C10_4	C11_4	119.8(3)
C12_4	C11_4	C10_4	122.2(3)
C12_4	C11_4	C16_4	119.2(3)
C16_4	C11_4	C10_4	118.3(3)
C11_4	C12_4	C13_4	120.1(4)
C14_4	C13_4	C12_4	120.8(4)
C15_4	C14_4	C13_4	120.1(4)
C14_4	C15_4	C16_4	120.6(4)
C15_4	C16_4	C11_4	119.2(3)
C18_4	C17_4	C9_4	121.7(3)
C18_4	C17_4	C22_4	118.8(3)
C22_4	C17_4	C9_4	119.5(3)
C17_4	C18_4	C19_4	120.5(4)
C20_4	C19_4	C18_4	119.5(4)
C19_4	C20_4	C21_4	121.1(4)
C20_4	C21_4	C22_4	120.0(4)
C21_4	C22_4	C17_4	120.1(4)
C24_4	C23_4	C8_4	121.7(3)
C24_4	C23_4	C28_4	118.6(3)
C28_4	C23_4	C8_4	119.6(3)
C25_4	C24_4	C23_4	121.0(4)
C26_4	C25_4	C24_4	120.5(4)

Atom	Atom	Atom	Angle/°
C25_4	C26_4	C27_4	119.6(4)
C26_4	C27_4	C28_4	121.1(4)
C23_4	C28_4	C27_4	119.0(4)
C30_4	C29_4	C7_4	120.1(3)
C34_4	C29_4	C7_4	121.5(3)
C34_4	C29_4	C30_4	118.4(3)
C31_4	C30_4	C29_4	121.3(3)
C30_4	C31_4	C32_4	118.7(4)
C33_4	C32_4	C31_4	121.5(3)
C32_4	C33_4	C34_4	119.1(4)
C29_4	C34_4	C33_4	120.9(3)
C36_4	C35_4	C2_4	108.9(3)
C36_4	C35_4	C37_4	107.8(3)
C37_4	C35_4	C2_4	106.9(3)
C38_4	C35_4	C2_4	113.1(3)
C38_4	C35_4	C36_4	111.2(3)
C38_4	C35_4	C37_4	108.7(3)
Cl1_6	C1_6	Cl2_6	110.0(5)
C39_8	N2_8	Rh2	132.1(3)
C39_8	N2_8	C43_8	115.3(4)
C43_8	N2_8	Rh2	111.5(3)
N2_8	C39_8	Cl3_8	115.5(3)
N2_8	C39_8	C40_8	127.2(5)
C40_8	C39_8	Cl3_8	117.2(4)
C39_8	C40_8	C41_8	116.1(5)
C42_8	C41_8	C40_8	121.2(5)
C41_8	C42_8	C43_8	117.2(5)
N2_8	C43_8	C42_8	122.8(4)

Atom	Atom	Atom	Angle/°
C39_9	N2_9	C43_9	115.3(4)
N2_9	C39_9	Cl3_9	115.3(4)
N2_9	C39_9	C40_9	127.5(5)
C40_9	C39_9	Cl3_9	117.2(4)
C39_9	C40_9	C41_9	115.9(5)
C42_9	C41_9	C40_9	121.2(5)
C41_9	C42_9	C43_9	117.6(5)
N2_9	C43_9	C42_9	122.5(5)
C39_10	N2_10	Rh1	135.6(5)
C39_10	N2_10	C43_10	115.3(5)
C43_10	N2_10	Rh1	109.2(4)
N2_10	C39_10	Cl3_10	114.9(4)
N2_10	C39_10	C40_10	127.5(5)
C40_10	C39_10	Cl3_10	117.6(4)
C39_10	C40_10	C41_10	115.7(5)
C42_10	C41_10	C40_10	121.2(6)
C41_10	C42_10	C43_10	117.2(5)
N2_10	C43_10	C42_10	122.7(5)
C39_13	N2_13	Rh1	130.7(4)
C39_13	N2_13	C43_13	115.4(4)
C43_13	N2_13	Rh1	112.8(4)
N2_13	C39_13	Cl3_13	114.9(4)
N2_13	C39_13	C40_13	127.3(5)
C40_13	C39_13	Cl3_13	117.7(4)
C39_13	C40_13	C41_13	115.6(5)
C42_13	C41_13	C40_13	121.2(6)
C41_13	C42_13	C43_13	117.1(5)
N2_13	C43_13	C42_13	122.7(5)

Table 5: Torsion Angles in ° for **JCS-Pyridine-TPPTTL-CoXtal-xtal2.**

Atom	Atom	Atom	Atom	Angle/°
Rh1	O1_1	C1_1	O2_1	1.3(6)
Rh1	O1_1	C1_1	C2_1	-179.4(3)
Rh1	O1_2	C1_2	O2_2	2.2(7)
Rh1	O1_2	C1_2	C2_2	176.8(3)
Rh1	O1_3	C1_3	O2_3	-8.9(6)
Rh1	O1_3	C1_3	C2_3	164.1(3)
Rh1	O1_4	C1_4	O2_4	-4.6(6)
Rh1	O1_4	C1_4	C2_4	170.1(3)
Rh1	N2_10	C39_10	Cl3_10	-2(3)
Rh1	N2_10	C39_10	C40_10	178.6(18)
Rh1	N2_10	C43_10	C42_10	-180(2)
Rh1	N2_13	C39_13	Cl3_13	-13.8(19)
Rh1	N2_13	C39_13	C40_13	170.5(16)
Rh1	N2_13	C43_13	C42_13	-171.1(13)
Rh2	O2_1	C1_1	O1_1	0.1(6)
Rh2	O2_1	C1_1	C2_1	-179.3(2)
Rh2	O2_2	C1_2	O1_2	1.2(7)
Rh2	O2_2	C1_2	C2_2	-173.7(2)
Rh2	O2_3	C1_3	O1_3	8.3(6)
Rh2	O2_3	C1_3	C2_3	-165.0(2)
Rh2	O2_4	C1_4	O1_4	6.4(6)
Rh2	O2_4	C1_4	C2_4	-168.6(2)
Rh2	N2_8	C39_8	Cl3_8	15.8(7)
Rh2	N2_8	C39_8	C40_8	-165.1(5)
Rh2	N2_8	C43_8	C42_8	166.4(4)
O1_1	C1_1	C2_1	N1_1	126.1(4)
O1_1	C1_1	C2_1	C35_1	-5.7(5)
O2_1	C1_1	C2_1	N1_1	-54.5(5)
O2_1	C1_1	C2_1	C35_1	173.7(3)
O3_1	C3_1	C4_1	C5_1	174.6(6)

Atom	Atom	Atom	Atom	Angle/°
O3_1	C3_1	C4_1	C10_1	-2.0(10)
N1_1	C2_1	C35_1	C36_1	172.2(3)
N1_1	C2_1	C35_1	C37_1	55.9(4)
N1_1	C2_1	C35_1	C38_1	-63.8(4)
N1_1	C3_1	C4_1	C5_1	-3.2(6)
N1_1	C3_1	C4_1	C10_1	-179.7(6)
C1_1	C2_1	C35_1	C36_1	-58.3(4)
C1_1	C2_1	C35_1	C37_1	-174.6(4)
C1_1	C2_1	C35_1	C38_1	65.7(4)
C2_1	N1_1	C3_1	O3_1	6.9(8)
C2_1	N1_1	C3_1	C4_1	-175.2(4)
C2_1	N1_1	C6_1	O4_1	1.3(9)
C2_1	N1_1	C6_1	C5_1	179.5(4)
C3_1	N1_1	C2_1	C1_1	121.3(4)
C3_1	N1_1	C2_1	C35_1	-105.0(5)
C3_1	N1_1	C6_1	O4_1	-170.3(6)
C3_1	N1_1	C6_1	C5_1	7.9(6)
C3_1	C4_1	C5_1	C6_1	7.8(6)
C3_1	C4_1	C5_1	C7_1	-171.3(5)
C3_1	C4_1	C10_1	C9_1	172.5(6)
C3_1	C4_1	C10_1	C11_1	-5.8(9)
C4_1	C5_1	C6_1	O4_1	168.4(6)
C4_1	C5_1	C6_1	N1_1	-9.7(6)
C4_1	C5_1	C7_1	C8_1	-4.4(9)
C4_1	C5_1	C7_1	C29_1	175.7(5)
C4_1	C10_1	C11_1	C12_1	123.1(6)
C4_1	C10_1	C11_1	C16_1	-59.0(8)
C5_1	C4_1	C10_1	C9_1	-3.6(9)
C5_1	C4_1	C10_1	C11_1	178.1(5)
C5_1	C7_1	C8_1	C9_1	1.8(9)
C5_1	C7_1	C8_1	C23_1	177.7(5)
C5_1	C7_1	C29_1	C30_1	-60.8(8)
C5_1	C7_1	C29_1	C34_1	126.1(6)
C6_1	N1_1	C2_1	C1_1	-49.4(5)
C6_1	N1_1	C2_1	C35_1	84.2(5)
C6_1	N1_1	C3_1	O3_1	178.9(5)
C6_1	N1_1	C3_1	C4_1	-3.2(6)
C6_1	C5_1	C7_1	C8_1	176.7(6)
C6_1	C5_1	C7_1	C29_1	-3.2(10)
C7_1	C5_1	C6_1	O4_1	-12.6(11)
C7_1	C5_1	C6_1	N1_1	169.3(6)
C7_1	C8_1	C9_1	C10_1	-0.2(9)
C7_1	C8_1	C9_1	C17_1	-175.1(5)
C7_1	C8_1	C23_1	C24_1	-65.7(7)
C7_1	C8_1	C23_1	C28_1	116.5(6)
C7_1	C29_1	C30_1	C31_1	-173.2(6)
C7_1	C29_1	C34_1	C33_1	173.3(5)
C8_1	C7_1	C29_1	C30_1	119.4(6)
C8_1	C7_1	C29_1	C34_1	-53.7(7)
C8_1	C9_1	C10_1	C4_1	1.0(9)
C8_1	C9_1	C10_1	C11_1	179.3(5)
C8_1	C9_1	C17_1	C18_1	109.4(8)
C8_1	C9_1	C17_1	C22_1	-69.8(9)
C8_1	C23_1	C24_1	C25_1	-177.4(5)
C8_1	C23_1	C28_1	C27_1	177.0(5)
C9_1	C8_1	C23_1	C24_1	110.4(6)
C9_1	C8_1	C23_1	C28_1	-67.4(7)
C9_1	C10_1	C11_1	C12_1	-55.1(8)
C9_1	C10_1	C11_1	C16_1	122.8(6)
C9_1	C17_1	C18_1	C19_1	-177.9(9)
C9_1	C17_1	C22_1	C21_1	-178.4(8)
C10_1	C4_1	C5_1	C6_1	-175.3(5)
C10_1	C4_1	C5_1	C7_1	5.6(9)

Atom	Atom	Atom	Atom	Angle/°
C10_1	C9_1	C17_1	C18_1	-65.5(9)
C10_1	C9_1	C17_1	C22_1	115.2(8)
C10_1	C11_1	C12_1	C13_1	179.8(6)
C10_1	C11_1	C16_1	C15_1	-178.7(6)
C11_1	C12_1	C13_1	C14_1	-1.5(11)
C12_1	C11_1	C16_1	C15_1	-0.7(9)
C12_1	C13_1	C14_1	C15_1	-0.1(12)
C13_1	C14_1	C15_1	C16_1	1.3(12)
C14_1	C15_1	C16_1	C11_1	-0.9(11)
C16_1	C11_1	C12_1	C13_1	1.9(9)
C17_1	C9_1	C10_1	C4_1	176.0(5)
C17_1	C9_1	C10_1	C11_1	-5.7(8)
C17_1	C18_1	C19_1	C20_1	-2(2)
C18_1	C17_1	C22_1	C21_1	2.3(14)
C18_1	C19_1	C20_1	C21_1	0(2)
C19_1	C20_1	C21_1	C22_1	4(2)
C20_1	C21_1	C22_1	C17_1	-5.0(18)
C22_1	C17_1	C18_1	C19_1	1.4(15)
C23_1	C8_1	C9_1	C10_1	-176.1(5)
C23_1	C8_1	C9_1	C17_1	9.0(8)
C23_1	C24_1	C25_1	C26_1	0.8(10)
C24_1	C23_1	C28_1	C27_1	-0.8(9)
C24_1	C25_1	C26_1	C27_1	-1.6(10)
C25_1	C26_1	C27_1	C28_1	1.2(10)
C26_1	C27_1	C28_1	C23_1	0.0(10)
C28_1	C23_1	C24_1	C25_1	0.4(9)
C29_1	C7_1	C8_1	C9_1	-178.4(5)
C29_1	C7_1	C8_1	C23_1	-2.4(8)
C29_1	C30_1	C31_1	C32_1	0.5(11)
C30_1	C29_1	C34_1	C33_1	0.0(8)
C30_1	C31_1	C32_1	C33_1	-1.4(11)
C31_1	C32_1	C33_1	C34_1	1.6(11)
C32_1	C33_1	C34_1	C29_1	-0.9(9)
C34_1	C29_1	C30_1	C31_1	0.1(9)
O1_2	C1_2	C2_2	N1_2	143.2(4)
O1_2	C1_2	C2_2	C35_2	10.5(5)
O2_2	C1_2	C2_2	N1_2	-41.4(5)
O2_2	C1_2	C2_2	C35_2	-174.1(3)
O3_2	C3_2	C4_2	C5_2	178.0(5)
O3_2	C3_2	C4_2	C10_2	-0.1(9)
N1_2	C2_2	C35_2	C36_2	158.1(3)
N1_2	C2_2	C35_2	C37_2	41.6(4)
N1_2	C2_2	C35_2	C38_2	-78.3(4)
N1_2	C3_2	C4_2	C5_2	-3.2(5)
N1_2	C3_2	C4_2	C10_2	178.7(5)
C1_2	C2_2	C35_2	C36_2	-71.4(4)
C1_2	C2_2	C35_2	C37_2	172.1(3)
C1_2	C2_2	C35_2	C38_2	52.2(4)
C2_2	N1_2	C3_2	O3_2	-2.4(7)
C2_2	N1_2	C3_2	C4_2	178.7(3)
C2_2	N1_2	C6_2	O4_2	6.1(7)
C2_2	N1_2	C6_2	C5_2	-173.9(4)
C3_2	N1_2	C2_2	C1_2	117.2(4)
C3_2	N1_2	C2_2	C35_2	-108.5(4)
C3_2	N1_2	C6_2	O4_2	-171.3(5)
C3_2	N1_2	C6_2	C5_2	8.6(5)
C3_2	C4_2	C5_2	C6_2	8.3(5)
C3_2	C4_2	C5_2	C7_2	-175.3(5)
C3_2	C4_2	C10_2	C9_2	177.3(5)
C3_2	C4_2	C10_2	C11_2	0.1(7)
C4_2	C5_2	C6_2	O4_2	169.5(5)
C4_2	C5_2	C6_2	N1_2	-10.5(5)
C4_2	C5_2	C7_2	C8_2	-3.5(7)

Atom	Atom	Atom	Atom	Angle/°
C4_2	C5_2	C7_2	C29_2	172.4(4)
C4_2	C10_2	C11_2	C12_2	116.6(5)
C4_2	C10_2	C11_2	C16_2	-59.9(6)
C5_2	C4_2	C10_2	C9_2	-0.5(7)
C5_2	C4_2	C10_2	C11_2	-177.7(4)
C5_2	C7_2	C8_2	C9_2	1.8(7)
C5_2	C7_2	C8_2	C23_2	179.4(4)
C5_2	C7_2	C29_2	C30_2	-61.0(7)
C5_2	C7_2	C29_2	C34_2	120.1(5)
C6_2	N1_2	C2_2	C1_2	-60.0(5)
C6_2	N1_2	C2_2	C35_2	74.3(5)
C6_2	N1_2	C3_2	O3_2	175.2(5)
C6_2	N1_2	C3_2	C4_2	-3.7(5)
C6_2	C5_2	C7_2	C8_2	172.0(5)
C6_2	C5_2	C7_2	C29_2	-12.1(8)
C7_2	C5_2	C6_2	O4_2	-6.6(9)
C7_2	C5_2	C6_2	N1_2	173.4(5)
C7_2	C8_2	C9_2	C10_2	0.6(7)
C7_2	C8_2	C9_2	C17_2	-176.3(4)
C7_2	C8_2	C23_2	C24_2	-61.4(7)
C7_2	C8_2	C23_2	C28_2	114.9(5)
C7_2	C29_2	C30_2	C31_2	-175.9(5)
C7_2	C29_2	C34_2	C33_2	177.5(5)
C8_2	C7_2	C29_2	C30_2	114.7(5)
C8_2	C7_2	C29_2	C34_2	-64.2(6)
C8_2	C9_2	C10_2	C4_2	-1.2(7)
C8_2	C9_2	C10_2	C11_2	176.1(4)
C8_2	C9_2	C17_2	C18_2	107.2(6)
C8_2	C9_2	C17_2	C22_2	-69.0(7)
C8_2	C23_2	C24_2	C25_2	179.4(6)
C8_2	C23_2	C28_2	C27_2	-177.9(5)
C9_2	C8_2	C23_2	C24_2	116.4(6)
C9_2	C8_2	C23_2	C28_2	-67.3(7)
C9_2	C10_2	C11_2	C12_2	-60.6(7)
C9_2	C10_2	C11_2	C16_2	122.9(5)
C9_2	C17_2	C18_2	C19_2	-176.4(5)
C9_2	C17_2	C22_2	C21_2	177.3(5)
C10_2	C4_2	C5_2	C6_2	-173.5(4)
C10_2	C4_2	C5_2	C7_2	3.0(8)
C10_2	C9_2	C17_2	C18_2	-69.8(6)
C10_2	C9_2	C17_2	C22_2	114.1(6)
C10_2	C11_2	C12_2	C13_2	-177.7(5)
C10_2	C11_2	C16_2	C15_2	178.2(5)
C11_2	C12_2	C13_2	C14_2	1.2(9)
C12_2	C11_2	C16_2	C15_2	1.6(8)
C12_2	C13_2	C14_2	C15_2	-1.5(10)
C13_2	C14_2	C15_2	C16_2	1.9(10)
C14_2	C15_2	C16_2	C11_2	-2.0(9)
C16_2	C11_2	C12_2	C13_2	-1.2(8)
C17_2	C9_2	C10_2	C4_2	175.7(4)
C17_2	C9_2	C10_2	C11_2	-7.0(6)
C17_2	C18_2	C19_2	C20_2	2.2(10)
C18_2	C17_2	C22_2	C21_2	1.0(9)
C18_2	C19_2	C20_2	C21_2	-5.1(11)
C19_2	C20_2	C21_2	C22_2	5.9(11)
C20_2	C21_2	C22_2	C17_2	-3.8(10)
C22_2	C17_2	C18_2	C19_2	-0.2(9)
C23_2	C8_2	C9_2	C10_2	-177.1(4)
C23_2	C8_2	C9_2	C17_2	6.0(6)
C23_2	C24_2	C25_2	C26_2	-2.8(12)
C24_2	C23_2	C28_2	C27_2	-1.5(9)
C24_2	C25_2	C26_2	C27_2	1.0(13)
C25_2	C26_2	C27_2	C28_2	0.5(13)

Atom	Atom	Atom	Atom	Angle/°
C26_2	C27_2	C28_2	C23_2	-0.2(11)
C28_2	C23_2	C24_2	C25_2	3.0(10)
C29_2	C7_2	C8_2	C9_2	-174.1(4)
C29_2	C7_2	C8_2	C23_2	3.5(6)
C29_2	C30_2	C31_2	C32_2	-2.1(9)
C30_2	C29_2	C34_2	C33_2	-1.5(8)
C30_2	C31_2	C32_2	C33_2	-0.5(9)
C31_2	C32_2	C33_2	C34_2	2.0(10)
C32_2	C33_2	C34_2	C29_2	-1.0(9)
C34_2	C29_2	C30_2	C31_2	3.1(8)
O1_3	C1_3	C2_3	N1_3	136.0(4)
O1_3	C1_3	C2_3	C35_3	3.7(5)
O2_3	C1_3	C2_3	N1_3	-50.1(5)
O2_3	C1_3	C2_3	C35_3	177.5(3)
O3_3	C3_3	C4_3	C5_3	-177.2(6)
O3_3	C3_3	C4_3	C10_3	7.2(10)
N1_3	C2_3	C35_3	C36_3	171.2(3)
N1_3	C2_3	C35_3	C37_3	54.8(4)
N1_3	C2_3	C35_3	C38_3	-65.1(4)
N1_3	C3_3	C4_3	C5_3	3.3(6)
N1_3	C3_3	C4_3	C10_3	-172.3(5)
C1_3	C2_3	C35_3	C36_3	-58.2(4)
C1_3	C2_3	C35_3	C37_3	-174.7(3)
C1_3	C2_3	C35_3	C38_3	65.5(4)
C2_3	N1_3	C3_3	O3_3	-4.8(7)
C2_3	N1_3	C3_3	C4_3	174.7(4)
C2_3	N1_3	C6_3	O4_3	5.0(8)
C2_3	N1_3	C6_3	C5_3	-175.1(4)
C3_3	N1_3	C2_3	C1_3	130.6(4)
C3_3	N1_3	C2_3	C35_3	-95.5(4)
C3_3	N1_3	C6_3	O4_3	-175.2(6)
C3_3	N1_3	C6_3	C5_3	4.7(6)
C3_3	C4_3	C5_3	C6_3	-0.5(6)
C3_3	C4_3	C5_3	C7_3	-177.0(5)
C3_3	C4_3	C10_3	C9_3	175.3(5)
C3_3	C4_3	C10_3	C11_3	-2.1(8)
C4_3	C5_3	C6_3	O4_3	177.5(6)
C4_3	C5_3	C6_3	N1_3	-2.5(6)
C4_3	C5_3	C7_3	C8_3	-0.2(7)
C4_3	C5_3	C7_3	C29_3	-179.2(4)
C4_3	C10_3	C11_3	C12_3	106.3(6)
C4_3	C10_3	C11_3	C16_3	-69.5(6)
C5_3	C4_3	C10_3	C9_3	0.3(7)
C5_3	C4_3	C10_3	C11_3	-177.2(4)
C5_3	C7_3	C8_3	C9_3	2.0(7)
C5_3	C7_3	C8_3	C23_3	-175.7(4)
C5_3	C7_3	C29_3	C30_3	-122.2(5)
C5_3	C7_3	C29_3	C34_3	69.0(7)
C6_3	N1_3	C2_3	C1_3	-49.6(5)
C6_3	N1_3	C2_3	C35_3	84.3(5)
C6_3	N1_3	C3_3	O3_3	175.4(5)
C6_3	N1_3	C3_3	C4_3	-5.1(6)
C6_3	C5_3	C7_3	C8_3	-175.7(5)
C6_3	C5_3	C7_3	C29_3	5.2(8)
C7_3	C5_3	C6_3	O4_3	-6.5(10)
C7_3	C5_3	C6_3	N1_3	173.6(5)
C7_3	C8_3	C9_3	C10_3	-2.8(7)
C7_3	C8_3	C9_3	C17_3	-176.2(4)
C7_3	C8_3	C23_3	C24_3	-106.0(6)
C7_3	C8_3	C23_3	C28_3	68.6(6)
C7_3	C29_3	C30_3	C31_3	-174.1(5)
C7_3	C29_3	C34_3	C33_3	171.7(5)
C8_3	C7_3	C29_3	C30_3	58.8(6)

Atom	Atom	Atom	Atom	Angle/°
C8_3	C7_3	C29_3	C34_3	-110.0(5)
C8_3	C9_3	C10_3	C4_3	1.5(7)
C8_3	C9_3	C10_3	C11_3	179.0(4)
C8_3	C9_3	C17_3	C18_3	100.8(6)
C8_3	C9_3	C17_3	C22_3	-80.5(6)
C8_3	C23_3	C24_3	C25_3	173.4(6)
C8_3	C23_3	C28_3	C27_3	-175.4(5)
C9_3	C8_3	C23_3	C24_3	76.2(7)
C9_3	C8_3	C23_3	C28_3	-109.1(6)
C9_3	C10_3	C11_3	C12_3	-71.0(6)
C9_3	C10_3	C11_3	C16_3	113.2(5)
C9_3	C17_3	C18_3	C19_3	-179.2(5)
C9_3	C17_3	C22_3	C21_3	-179.3(5)
C10_3	C4_3	C5_3	C6_3	175.5(5)
C10_3	C4_3	C5_3	C7_3	-1.0(8)
C10_3	C9_3	C17_3	C18_3	-72.8(6)
C10_3	C9_3	C17_3	C22_3	105.9(6)
C10_3	C11_3	C12_3	C13_3	-175.4(5)
C10_3	C11_3	C16_3	C15_3	177.1(5)
C11_3	C12_3	C13_3	C14_3	-1.4(10)
C12_3	C11_3	C16_3	C15_3	1.2(9)
C12_3	C13_3	C14_3	C15_3	0.7(10)
C13_3	C14_3	C15_3	C16_3	0.9(10)
C14_3	C15_3	C16_3	C11_3	-1.9(10)
C16_3	C11_3	C12_3	C13_3	0.4(9)
C17_3	C9_3	C10_3	C4_3	175.2(4)
C17_3	C9_3	C10_3	C11_3	-7.4(6)
C17_3	C18_3	C19_3	C20_3	-1.9(10)
C18_3	C17_3	C22_3	C21_3	-0.6(9)
C18_3	C19_3	C20_3	C21_3	0.1(11)
C19_3	C20_3	C21_3	C22_3	1.4(11)
C20_3	C21_3	C22_3	C17_3	-1.1(11)
C22_3	C17_3	C18_3	C19_3	2.1(9)
C23_3	C8_3	C9_3	C10_3	174.9(4)
C23_3	C8_3	C9_3	C17_3	1.4(6)
C23_3	C24_3	C25_3	C26_3	3.4(11)
C24_3	C23_3	C28_3	C27_3	-0.5(9)
C24_3	C25_3	C26_3	C27_3	-3.6(11)
C25_3	C26_3	C27_3	C28_3	1.8(11)
C26_3	C27_3	C28_3	C23_3	0.3(11)
C28_3	C23_3	C24_3	C25_3	-1.3(10)
C29_3	C7_3	C8_3	C9_3	-178.9(4)
C29_3	C7_3	C8_3	C23_3	3.4(6)
C29_3	C30_3	C31_3	C32_3	5.5(8)
C30_3	C29_3	C34_3	C33_3	2.8(8)
C30_3	C31_3	C32_3	C33_3	-3.9(9)
C31_3	C32_3	C33_3	C34_3	1.8(9)
C32_3	C33_3	C34_3	C29_3	-1.2(9)
C34_3	C29_3	C30_3	C31_3	-4.9(8)
O1_4	C1_4	C2_4	N1_4	126.9(4)
O1_4	C1_4	C2_4	C35_4	-3.5(5)
O2_4	C1_4	C2_4	N1_4	-57.8(4)
O2_4	C1_4	C2_4	C35_4	171.9(3)
O3_4	C3_4	C4_4	C5_4	-177.4(5)
O3_4	C3_4	C4_4	C10_4	-1.9(9)
N1_4	C2_4	C35_4	C36_4	174.8(3)
N1_4	C2_4	C35_4	C37_4	58.6(4)
N1_4	C2_4	C35_4	C38_4	-61.0(4)
N1_4	C3_4	C4_4	C5_4	3.4(5)
N1_4	C3_4	C4_4	C10_4	178.8(5)
C1_4	C2_4	C35_4	C36_4	-56.9(4)
C1_4	C2_4	C35_4	C37_4	-173.2(3)
C1_4	C2_4	C35_4	C38_4	67.2(4)

Atom	Atom	Atom	Atom	Angle/°
C2_4	N1_4	C3_4	O3_4	-1.6(7)
C2_4	N1_4	C3_4	C4_4	177.8(4)
C2_4	N1_4	C6_4	O4_4	6.0(8)
C2_4	N1_4	C6_4	C5_4	-177.8(4)
C3_4	N1_4	C2_4	C1_4	129.5(4)
C3_4	N1_4	C2_4	C35_4	-98.4(4)
C3_4	N1_4	C6_4	O4_4	-170.3(5)
C3_4	N1_4	C6_4	C5_4	5.9(5)
C3_4	C4_4	C5_4	C6_4	0.2(5)
C3_4	C4_4	C5_4	C7_4	177.7(4)
C3_4	C4_4	C10_4	C9_4	-177.6(5)
C3_4	C4_4	C10_4	C11_4	3.6(8)
C4_4	C5_4	C6_4	O4_4	172.4(5)
C4_4	C5_4	C6_4	N1_4	-3.6(5)
C4_4	C5_4	C7_4	C8_4	-1.2(7)
C4_4	C5_4	C7_4	C29_4	173.5(4)
C4_4	C10_4	C11_4	C12_4	62.3(7)
C4_4	C10_4	C11_4	C16_4	-125.0(5)
C5_4	C4_4	C10_4	C9_4	-2.7(7)
C5_4	C4_4	C10_4	C11_4	178.5(4)
C5_4	C7_4	C8_4	C9_4	1.9(7)
C5_4	C7_4	C8_4	C23_4	175.7(4)
C5_4	C7_4	C29_4	C30_4	-57.2(6)
C5_4	C7_4	C29_4	C34_4	123.1(5)
C6_4	N1_4	C2_4	C1_4	-46.4(5)
C6_4	N1_4	C2_4	C35_4	85.7(5)
C6_4	N1_4	C3_4	O3_4	174.8(5)
C6_4	N1_4	C3_4	C4_4	-5.9(5)
C6_4	C5_4	C7_4	C8_4	175.7(5)
C6_4	C5_4	C7_4	C29_4	-9.6(8)
C7_4	C5_4	C6_4	O4_4	-4.9(9)
C7_4	C5_4	C6_4	N1_4	179.1(5)
C7_4	C8_4	C9_4	C10_4	-3.1(7)
C7_4	C8_4	C9_4	C17_4	171.2(4)
C7_4	C8_4	C23_4	C24_4	-89.8(6)
C7_4	C8_4	C23_4	C28_4	95.2(5)
C7_4	C29_4	C30_4	C31_4	-175.0(5)
C7_4	C29_4	C34_4	C33_4	177.8(5)
C8_4	C7_4	C29_4	C30_4	117.3(5)
C8_4	C7_4	C29_4	C34_4	-62.4(6)
C8_4	C9_4	C10_4	C4_4	3.4(6)
C8_4	C9_4	C10_4	C11_4	-177.8(4)
C8_4	C9_4	C17_4	C18_4	67.6(6)
C8_4	C9_4	C17_4	C22_4	-114.1(5)
C8_4	C23_4	C24_4	C25_4	-177.3(5)
C8_4	C23_4	C28_4	C27_4	175.1(5)
C9_4	C8_4	C23_4	C24_4	84.2(6)
C9_4	C8_4	C23_4	C28_4	-90.8(5)
C9_4	C10_4	C11_4	C12_4	-116.5(5)
C9_4	C10_4	C11_4	C16_4	56.2(6)
C9_4	C17_4	C18_4	C19_4	-178.9(5)
C9_4	C17_4	C22_4	C21_4	177.3(5)
C10_4	C4_4	C5_4	C6_4	-175.8(4)
C10_4	C4_4	C5_4	C7_4	1.7(8)
C10_4	C9_4	C17_4	C18_4	-118.0(5)
C10_4	C9_4	C17_4	C22_4	60.3(6)
C10_4	C11_4	C12_4	C13_4	174.0(5)
C10_4	C11_4	C16_4	C15_4	-173.0(5)
C11_4	C12_4	C13_4	C14_4	-2.3(9)
C12_4	C11_4	C16_4	C15_4	-0.1(8)
C12_4	C13_4	C14_4	C15_4	2.0(10)
C13_4	C14_4	C15_4	C16_4	-0.7(10)
C14_4	C15_4	C16_4	C11_4	-0.3(9)

Atom	Atom	Atom	Atom	Angle/°
C16_4	C11_4	C12_4	C13_4	1.4(8)
C17_4	C9_4	C10_4	C4_4	-171.0(4)
C17_4	C9_4	C10_4	C11_4	7.9(6)
C17_4	C18_4	C19_4	C20_4	-1.2(9)
C18_4	C17_4	C22_4	C21_4	-4.3(8)
C18_4	C19_4	C20_4	C21_4	1.2(10)
C19_4	C20_4	C21_4	C22_4	-2.7(10)
C20_4	C21_4	C22_4	C17_4	4.3(9)
C22_4	C17_4	C18_4	C19_4	2.8(8)
C23_4	C8_4	C9_4	C10_4	-176.9(4)
C23_4	C8_4	C9_4	C17_4	-2.6(6)
C23_4	C24_4	C25_4	C26_4	4.3(10)
C24_4	C23_4	C28_4	C27_4	0.0(8)
C24_4	C25_4	C26_4	C27_4	-3.8(11)
C25_4	C26_4	C27_4	C28_4	1.5(11)
C26_4	C27_4	C28_4	C23_4	0.4(10)
C28_4	C23_4	C24_4	C25_4	-2.3(8)
C29_4	C7_4	C8_4	C9_4	-172.8(4)
C29_4	C7_4	C8_4	C23_4	1.0(6)
C29_4	C30_4	C31_4	C32_4	-4.6(9)
C30_4	C29_4	C34_4	C33_4	-1.9(8)
C30_4	C31_4	C32_4	C33_4	1.7(10)
C31_4	C32_4	C33_4	C34_4	1.1(10)
C32_4	C33_4	C34_4	C29_4	-0.9(9)
C34_4	C29_4	C30_4	C31_4	4.7(8)
Cl3_8	C39_8	C40_8	C41_8	-180.0(5)
N2_8	C39_8	C40_8	C41_8	0.9(11)
C39_8	N2_8	C43_8	C42_8	-2.9(8)
C39_8	C40_8	C41_8	C42_8	-2.1(11)
C40_8	C41_8	C42_8	C43_8	0.8(10)
C41_8	C42_8	C43_8	N2_8	1.9(9)
C43_8	N2_8	C39_8	Cl3_8	-177.6(4)
C43_8	N2_8	C39_8	C40_8	1.5(9)
Cl3_9	C39_9	C40_9	C41_9	-179.1(6)
N2_9	C39_9	C40_9	C41_9	1.2(11)
C39_9	N2_9	C43_9	C42_9	1.9(10)
C39_9	C40_9	C41_9	C42_9	-0.7(11)
C40_9	C41_9	C42_9	C43_9	0.9(12)
C41_9	C42_9	C43_9	N2_9	-1.5(11)
C43_9	N2_9	C39_9	Cl3_9	178.5(5)
C43_9	N2_9	C39_9	C40_9	-1.8(10)
Cl3_10	C39_10	C40_10	C41_10	-174.3(18)
N2_10	C39_10	C40_10	C41_10	5(4)
C39_10	N2_10	C43_10	C42_10	1(4)
C39_10	C40_10	C41_10	C42_10	-8(3)
C40_10	C41_10	C42_10	C43_10	7(4)
C41_10	C42_10	C43_10	N2_10	-4(4)
C43_10	N2_10	C39_10	Cl3_10	177.9(17)
C43_10	N2_10	C39_10	C40_10	-2(4)
Cl3_13	C39_13	C40_13	C41_13	176.7(16)
N2_13	C39_13	C40_13	C41_13	-8(3)
C39_13	N2_13	C43_13	C42_13	-2(2)
C39_13	C40_13	C41_13	C42_13	11(3)
C40_13	C41_13	C42_13	C43_13	-9(3)
C41_13	C42_13	C43_13	N2_13	5(3)
C43_13	N2_13	C39_13	Cl3_13	179.1(11)
C43_13	N2_13	C39_13	C40_13	3(3)

Table 6: Hydrogen Fractional Atomic Coordinates (×10⁴) and Equivalent Isotropic Displacement Parameters (Å²×10³) for JCS-Pyridine-TPPTTL-CoXtal-xtal2. U_{eq} is defined as 1/3 of the trace of the orthogonalised U_{ij}.

Atom	x	y	z	U_{eq}

Atom	x	y	z	U_{eq}
H2_1	8965.74	5011.76	6208.73	34
H12_1	9368.47	2416.2	5207.03	44
H13_1	9672.67	2819.53	4697.61	58
H14_1	9148.86	3728.82	4481.96	63
H15_1	8315.89	4247.75	4769.66	57
H16_1	8018.26	3883.63	5291.53	46
H18_1	7407.13	2295.58	5175.41	100
H19_1	7237.62	1577.29	4733.59	127
H20_1	7913.98	674.61	4673.58	135
H21_1	8775.08	473.3	5035.72	110
H22_1	8877.82	1113.99	5505.99	92
H24_1	8675.77	1003.67	6298.15	45
H25_1	8386.51	-95.21	6341.96	54
H26_1	7440.46	-489.97	6091.78	49
H27_1	6738.78	221.29	5814.49	48
H28_1	7005.56	1340.81	5769.15	49
H30_1	8801.35	2344.5	6806.24	56
H31_1	8577.77	1846.19	7304.6	84
H32_1	7556.26	1324.96	7380.71	84
H33_1	6751.69	1336.5	6981.19	74
H34_1	6982.19	1834.25	6479.46	52
H36A_1	10154.89	5495.57	6760.44	61
H36B_1	9396.73	5624.46	6862.55	61
H36C_1	9627.52	5737.16	6494.41	61
H37A_1	9995.45	4608.33	6166.9	72
H37B_1	10079.33	3998.54	6408.13	72
H37C_1	10506.18	4651.09	6464.88	72
H38A_1	9866.58	4431.11	7057.02	67
H38B_1	9475.25	3873.11	6861.34	67
H38C_1	9070.39	4447.71	7037.7	67
H2_2	6834.03	3421.67	6917.33	27
H12_2	4429.95	1411.53	6447.77	42
H13_2	4908.91	643.29	6099.03	53
H14_2	5663.84	964.23	5706.54	62
H15_2	5907.05	2059.97	5636.85	53
H16_2	5448.25	2851.99	5985.54	38
H18_2	3708.34	2931.68	5863.36	43
H19_2	2958.46	2395.31	5511.38	57
H20_2	2200.14	1661	5721.47	64
H21_2	2284.13	1312.57	6256.98	61
H22_2	3005.49	1855.02	6614.9	50
H24_2	2811.34	2984.07	7225.93	54
H25_2	1662.04	3072.52	7268.09	70
H26_2	1045.18	3506.18	6846.75	70
H27_2	1570.2	3820.17	6368.51	61
H28_2	2728.53	3695.15	6307.16	45
H30_2	4175	3549.56	7573.04	41
H31_2	3674.62	4150.32	7989.1	42
H32_2	2993.03	5034.68	7855.57	47
H33_2	2817.19	5328.74	7321.46	48
H34_2	3281.9	4692.28	6904.24	41
H36A_2	7630.44	3159.5	7259.49	52
H36B_2	7549.69	3145.41	7646.44	52
H36C_2	7666.15	3827.31	7462.79	52
H37A_2	5887.37	2853.97	7448	52
H37B_2	6503.34	2623.53	7666.99	52
H37C_2	6519.42	2512.52	7281.97	52
H38A_2	6741.01	4307.41	7703.84	46
H38B_2	6368.51	3706.8	7875.81	46
H38C_2	5996.45	4120.99	7599.79	46
H2_3	5099.99	5692	7145.95	25
H12_3	2323.41	7139.51	7002.66	46
H13_3	1524.11	6326.96	7085.13	54

Atom	x	y	z	U_{eq}
H14_3	1519	5387.87	6772.06	49
H15_3	2324.02	5235.65	6378.74	52
H16_3	3170.71	6015.39	6303.77	46
H18_3	2677.98	7365.67	5926.13	47
H19_3	1626.76	7621.65	5703.5	61
H20_3	911.34	8295.61	5985.11	69
H21_3	1210.7	8731.52	6484.45	67
H22_3	2269.72	8522.64	6700.2	58
H24_3	3120.12	9312.22	6628.37	50
H25_3	3056.44	10337.65	6387.27	54
H26_3	3462.26	10504.02	5869.24	57
H27_3	4050.6	9674.14	5609.98	62
H28_3	4178.74	8643.52	5857.62	47
H30_3	4538.07	9277.54	6801.3	33
H31_3	5347.47	10086.74	6733.66	35
H32_3	6196.27	9918.28	6356.09	45
H33_3	6327.62	8917.46	6103.3	35
H34_3	5547.94	8082.75	6198.32	31
H36A_3	5630.85	5804.56	7957.28	46
H36B_3	6128.42	5689.05	7658.75	46
H36C_3	5433.03	5306.3	7672.77	46
H37A_3	4386.94	5875.57	7610.16	54
H37B_3	4355.76	6634.99	7514.03	54
H37C_3	4567.91	6423.64	7874.07	54
H38A_3	5667.79	6996.9	7853.11	43
H38B_3	5398.8	7293.52	7517.75	43
H38C_3	6110.37	6940.57	7530.73	43
H2_4	7124.92	7618.13	6432.41	26
H12_4	7884.09	9193.77	5520.3	40
H13_4	7146.77	9908.66	5261.65	45
H14_4	6657.54	9625.37	4773.95	49
H15_4	6837.21	8601.76	4552.75	46
H16_4	7543.03	7848.79	4813.98	36
H18_4	8870	7321.28	4542.16	39
H19_4	9080.35	7852.18	4043.51	46
H20_4	9263.81	8965.9	4034.32	51
H21_4	9299.63	9565.18	4513.06	48
H22_4	9042.83	9058.58	5008.28	42
H24_4	10501.22	7963.91	5209.61	46
H25_4	11414.54	7710.67	4887.1	62
H26_4	11352.1	6872.76	4511.21	68
H27_4	10422.59	6195.71	4500.72	67
H28_4	9514.61	6390.58	4848.83	44
H30_4	10035.8	7315.46	6108.82	41
H31_4	10960.18	6744.07	6302.86	46
H32_4	11222.4	5729.72	6076.17	49
H33_4	10621.29	5317.64	5644.18	50
H34_4	9747.19	5937.04	5423.57	38
H36A_4	6937.63	7872.17	6973.72	60
H36B_4	7457.77	8312.18	7171.99	60
H36C_4	7515.84	7530.11	7181.02	60
H37A_4	8067.48	8630.69	6397.08	54
H37B_4	8038.14	8931.21	6756.13	54
H37C_4	7363.33	8745.82	6570.43	54
H38A_4	8634.07	7304.79	6875.31	63
H38B_4	8692.63	8008.75	7044.48	63
H38C_4	8880.62	7923.68	6668.8	63
H1A_6	6512.23	5667.14	4300.53	74
H1B_6	7206.7	5431.83	4147.16	74
H40_8	6380.48	4708.15	5297.66	56
H41_8	5845.41	5693.84	5159.25	61
H42_8	5622.36	6474.97	5556.85	45
H43_8	5965.94	6255.47	6094.2	38

Atom	x	y	z	U_{eq}
H40_9	8460.95	5481.37	4870.97	57
H41_9	8658.85	5054.06	5393	66
H42_9	8134.21	5508.06	5843.76	70
H43_9	7376.69	6375.87	5760.95	59
H40_10	8423.39	5994.55	8250.2	74
H41_10	9323.36	6626.15	8057.1	78
H42_10	9533.19	6677.53	7500.47	97
H43_10	8690.49	6312.07	7143.57	78
H40_13	8153.78	6106.77	8246.81	119
H41_13	8552.86	5030.21	8323.89	106
H42_13	8769.19	4376.27	7870.86	95
H43_13	8272.69	4683.22	7372.92	71

Table 7: Atomic Occupancies for all atoms that are not fully occupied in **JCS-Pyridine-TPPTTL-CoXtal-xtal2.**

Atom	Occupancy	Atom	Occupancy	Atom	Occupancy	Atom	Occupancy
Cl3_10	0.405(5)	H41_10	0.405(5)	N2_13	0.595(5)	C42_13	0.595(5)
N2_10	0.405(5)	C42_10	0.405(5)	C39_13	0.595(5)	H42_13	0.595(5)
C39_10	0.405(5)	H42_10	0.405(5)	C40_13	0.595(5)	C43_13	0.595(5)
C40_10	0.405(5)	C43_10	0.405(5)	H40_13	0.595(5)	H43_13	0.595(5)
H40_10	0.405(5)	H43_10	0.405(5)	C41_13	0.595(5)		
C41_10	0.405(5)	Cl3_13	0.595(5)	H41_13	0.595(5)		

Table 8: Solvent masking (Olex2) information for **JCS-Pyridine-TPPTTL-CoXtal-xtal2.**

No	x	y	z	V	e	Content
1	-0.615	-0.822	-0.761	7692.7	1167.2	?
2	0.037	0.583	0.236	0.0	0.0	?
3	0.046	0.917	0.889	0.0	0.0	?
4	0.046	0.931	0.894	0.0	0.0	?
5	0.065	0.398	0.227	0.0	0.0	?
6	0.074	0.546	0.301	0.0	0.0	?
7	0.083	0.194	0.032	0.0	0.0	?
8	0.083	0.204	0.028	0.0	0.0	?
9	0.093	0.093	0.796	0.0	0.0	?
10	0.093	0.889	0.838	0.0	0.0	?
11	0.102	0.880	0.829	0.0	0.0	?
12	0.111	0.556	0.319	0.0	0.0	?
13	0.111	0.880	0.824	0.0	0.0	?
14	0.120	0.574	0.324	0.0	0.0	?
15	0.130	0.713	0.602	0.0	0.0	?
16	0.130	0.796	0.056	0.0	0.0	?
17	0.130	0.806	0.060	0.0	0.0	?
18	0.130	0.954	0.778	0.0	0.0	?
19	0.139	0.375	0.162	0.0	0.0	?
20	0.139	0.444	0.222	0.0	0.0	?
21	0.148	0.444	0.218	0.0	0.0	?
22	0.148	0.481	0.384	0.0	0.0	?
23	0.148	0.685	0.509	0.0	0.0	?
24	0.157	0.093	0.801	0.0	0.0	?
25	0.157	0.444	0.213	0.0	0.0	?
26	0.157	0.648	0.403	0.0	0.0	?
27	0.157	0.667	0.495	0.0	0.0	?
28	0.157	0.685	0.505	0.0	0.0	?
29	0.167	0.296	0.282	0.0	0.0	?
30	0.176	0.204	0.829	0.0	0.0	?
31	0.176	0.278	0.292	0.0	0.0	?
32	0.176	0.690	0.486	0.0	0.0	?
33	0.176	0.907	0.454	0.0	0.0	?
34	0.185	0.148	0.912	0.0	0.0	?
35	0.185	0.278	0.287	0.0	0.0	?

No	x	y	z	V	e	Content
36	0.185	0.870	0.250	0.0	0.0	?
37	0.185	0.889	0.394	0.0	0.0	?
38	0.194	0.157	0.912	0.0	0.0	?
39	0.194	0.852	0.259	0.0	0.0	?
40	0.204	0.167	0.912	0.0	0.0	?
41	0.204	0.685	0.120	0.0	0.0	?
42	0.204	0.769	0.977	0.0	0.0	?
43	0.222	0.352	0.935	0.0	0.0	?
44	0.213	0.833	0.796	0.0	0.0	?
45	0.213	0.861	0.264	0.0	0.0	?
46	0.222	0.620	0.852	0.0	0.0	?
47	0.222	0.907	0.560	0.0	0.0	?
48	0.222	0.926	0.546	0.0	-0.0	?
49	0.231	0.009	0.394	0.0	0.0	?
50	0.231	0.620	0.856	0.0	0.0	?
51	0.231	0.722	0.139	0.0	0.0	?
52	0.231	0.926	0.551	0.0	-0.0	?
53	0.241	0.796	0.565	0.0	0.0	?
54	0.241	0.824	0.574	0.0	0.0	?
55	0.241	0.954	0.889	0.0	0.0	?
56	0.241	0.963	0.894	0.0	0.0	?
57	0.259	0.037	0.394	0.0	0.0	?
58	0.259	0.046	0.389	0.0	0.0	?
59	0.259	0.176	0.074	0.0	0.0	?
60	0.259	0.204	0.065	0.0	0.0	?
61	0.269	0.074	0.051	0.0	0.0	?
62	0.269	0.278	0.639	0.0	0.0	?
63	0.269	0.380	0.356	0.0	0.0	?
64	0.278	0.648	0.435	0.0	0.0	?
65	0.269	0.991	0.894	0.0	0.0	?
66	0.278	0.074	0.046	0.0	0.0	?
67	0.278	0.093	0.060	0.0	0.0	?
68	0.278	0.380	0.352	0.0	0.0	?
69	0.287	0.139	0.764	0.0	0.0	?
70	0.287	0.167	0.296	0.0	0.0	?
71	0.296	0.231	0.477	0.0	0.0	?
72	0.296	0.315	0.620	0.0	0.0	?
73	0.296	0.833	0.412	0.0	0.0	?
74	0.306	0.148	0.759	0.0	0.0	?
75	0.306	0.843	0.412	0.0	0.0	?
76	0.315	0.111	0.894	0.0	0.0	?
77	0.315	0.130	0.750	0.0	0.0	?
78	0.315	0.722	0.787	0.0	0.0	?
79	0.315	0.852	0.412	0.0	0.0	?
80	0.324	0.093	0.954	0.0	0.0	?
81	0.324	0.310	0.986	0.0	0.0	?
82	0.324	0.722	0.792	0.0	0.0	?
83	0.324	0.796	0.329	0.0	0.0	?
84	0.333	0.704	0.782	0.0	0.0	?
85	0.343	0.315	0.005	0.0	0.0	?
86	0.343	0.333	0.995	0.0	0.0	?
87	0.343	0.352	0.903	0.0	0.0	?
88	0.343	0.556	0.713	0.0	0.0	?
89	0.343	0.907	0.301	0.0	0.0	?
90	0.352	0.315	0.009	0.0	0.0	?
91	0.352	0.519	0.884	0.0	0.0	?
92	0.352	0.556	0.718	0.0	0.0	?
93	0.361	0.556	0.722	0.0	0.0	?
94	0.361	0.625	0.662	0.0	0.0	?
95	0.370	0.046	0.278	0.0	0.0	?
96	0.370	0.194	0.560	0.0	0.0	?
97	0.370	0.204	0.556	0.0	0.0	?
98	0.370	0.287	0.102	0.0	0.0	?

No	x	y	z	V	e	Content
99	0.380	0.426	0.824	0.0	0.0	?
100	0.389	0.120	0.324	0.0	0.0	?
101	0.389	0.444	0.819	0.0	0.0	?
102	0.398	0.120	0.329	0.0	0.0	?
103	0.407	0.111	0.338	0.0	0.0	?
104	0.407	0.907	0.296	0.0	0.0	?
105	0.417	0.796	0.528	0.0	0.0	?
106	0.417	0.806	0.532	0.0	0.0	?
107	0.426	0.454	0.801	0.0	0.0	?
108	0.435	0.602	0.727	0.0	0.0	?
109	0.454	0.069	0.394	0.0	0.0	?
110	0.454	0.083	0.389	0.0	0.0	?
111	0.463	0.417	0.736	0.0	0.0	?
112	0.537	0.917	0.764	0.0	0.0	?
113	0.546	0.569	0.106	0.0	0.0	?
114	0.546	0.583	0.111	0.0	0.0	?
115	0.565	0.102	0.773	0.0	0.0	?
116	0.574	0.954	0.699	0.0	0.0	?
117	0.583	0.296	0.972	0.0	0.0	?
118	0.583	0.306	0.968	0.0	0.0	?
119	0.593	0.407	0.204	0.0	0.0	?
120	0.593	0.611	0.162	0.0	0.0	?
121	0.602	0.620	0.171	0.0	0.0	?
122	0.611	0.620	0.176	0.0	0.0	?
123	0.611	0.944	0.681	0.0	0.0	?
124	0.620	0.926	0.676	0.0	0.0	?
125	0.630	0.546	0.222	0.0	0.0	?
126	0.630	0.694	0.940	0.0	0.0	?
127	0.630	0.704	0.944	0.0	0.0	?
128	0.630	0.787	0.398	0.0	0.0	?
129	0.639	0.056	0.778	0.0	0.0	?
130	0.639	0.125	0.838	0.0	0.0	?
131	0.648	0.019	0.616	0.0	0.0	?
132	0.648	0.056	0.782	0.0	0.0	?
133	0.648	0.815	0.491	0.0	0.0	?
134	0.657	0.056	0.787	0.0	0.0	?
135	0.657	0.407	0.199	0.0	0.0	?
136	0.657	0.815	0.495	0.0	0.0	?
137	0.657	0.833	0.505	0.0	0.0	?
138	0.657	0.852	0.597	0.0	0.0	?
139	0.667	0.204	0.718	0.0	0.0	?
140	0.676	0.222	0.708	0.0	0.0	?
141	0.676	0.296	0.171	0.0	0.0	?
142	0.676	0.593	0.546	0.0	0.0	?
143	0.676	0.810	0.514	0.0	0.0	?
144	0.685	0.222	0.713	0.0	0.0	?
145	0.685	0.352	0.088	0.0	0.0	?
146	0.685	0.611	0.606	0.0	0.0	?
147	0.685	0.630	0.750	0.0	0.0	?
148	0.694	0.343	0.088	0.0	0.0	?
149	0.694	0.648	0.741	0.0	0.0	?
150	0.704	0.333	0.088	0.0	0.0	?
151	0.704	0.731	0.023	0.0	0.0	?
152	0.704	0.815	0.880	0.0	0.0	?
153	0.722	0.148	0.065	0.0	0.0	?
154	0.713	0.639	0.736	0.0	0.0	?
155	0.713	0.667	0.204	0.0	0.0	?
156	0.722	0.574	0.454	0.0	-0.0	?
157	0.722	0.593	0.440	0.0	0.0	?
158	0.722	0.880	0.148	0.0	0.0	?
159	0.731	0.491	0.606	0.0	0.0	?
160	0.731	0.574	0.449	0.0	-0.0	?
161	0.731	0.778	0.861	0.0	0.0	?

No	x	y	z	V	e	Content
162	0.731	0.880	0.144	0.0	0.0	?
163	0.741	0.537	0.106	0.0	0.0	?
164	0.741	0.546	0.111	0.0	0.0	?
165	0.741	0.676	0.426	0.0	0.0	?
166	0.741	0.704	0.435	0.0	0.0	?
167	0.759	0.296	0.935	0.0	0.0	?
168	0.759	0.324	0.926	0.0	0.0	?
169	0.759	0.454	0.611	0.0	0.0	?
170	0.759	0.463	0.606	0.0	0.0	?
171	0.769	0.120	0.644	0.0	0.0	?
172	0.769	0.222	0.361	0.0	0.0	?
173	0.769	0.426	0.949	0.0	0.0	?
174	0.769	0.509	0.106	0.0	0.0	?
175	0.778	0.852	0.565	0.0	0.0	?
176	0.778	0.120	0.648	0.0	0.0	?
177	0.778	0.407	0.940	0.0	0.0	?
178	0.778	0.426	0.954	0.0	0.0	?
179	0.787	0.333	0.704	0.0	0.0	?
180	0.787	0.361	0.236	0.0	0.0	?
181	0.796	0.185	0.380	0.0	0.0	?
182	0.796	0.269	0.523	0.0	0.0	?
183	0.796	0.667	0.588	0.0	0.0	?
184	0.806	0.352	0.241	0.0	0.0	?
185	0.806	0.657	0.588	0.0	0.0	?
186	0.815	0.370	0.250	0.0	0.0	?
187	0.815	0.389	0.106	0.0	0.0	?
188	0.815	0.648	0.588	0.0	0.0	?
189	0.815	0.778	0.213	0.0	0.0	?
190	0.824	0.190	0.014	0.0	0.0	?
191	0.824	0.407	0.046	0.0	0.0	?
192	0.824	0.704	0.671	0.0	0.0	?
193	0.824	0.778	0.208	0.0	0.0	?
194	0.833	0.796	0.218	0.0	0.0	?
195	0.843	0.148	0.097	0.0	0.0	?
196	0.843	0.167	0.005	0.0	0.0	?
197	0.843	0.185	0.995	0.0	0.0	?
198	0.843	0.593	0.699	0.0	0.0	?
199	0.843	0.944	0.287	0.0	0.0	?
200	0.852	0.185	0.991	0.0	0.0	?
201	0.852	0.944	0.282	0.0	0.0	?
202	0.852	0.981	0.116	0.0	0.0	?
203	0.861	0.875	0.338	0.0	0.0	?
204	0.861	0.944	0.278	0.0	0.0	?
205	0.870	0.213	0.898	0.0	0.0	?
206	0.870	0.296	0.444	0.0	0.0	?
207	0.870	0.306	0.440	0.0	0.0	?
208	0.870	0.454	0.722	0.0	0.0	?
209	0.880	0.074	0.176	0.0	0.0	?
210	0.889	0.056	0.181	0.0	0.0	?
211	0.889	0.380	0.676	0.0	0.0	?
212	0.898	0.380	0.671	0.0	0.0	?
213	0.907	0.389	0.662	0.0	0.0	?
214	0.907	0.593	0.704	0.0	0.0	?
215	0.917	0.694	0.468	0.0	0.0	?
216	0.917	0.704	0.472	0.0	0.0	?
217	0.926	0.046	0.199	0.0	0.0	?
218	0.935	0.898	0.273	0.0	0.0	?
219	0.954	0.417	0.611	0.0	0.0	?
220	0.954	0.431	0.606	0.0	0.0	?
221	0.963	0.083	0.264	0.0	0.0	?

Citations

O.V. Dolomanov and L.J. Bourhis and R.J. Gildea and J.A.K. Howard and H. Puschmann, Olex2: A complete structure solution, refinement and analysis program, *J. Appl. Cryst.*, (2009), 42, 339-341.

7.2: 2,2,2-Trichloroethyl (1*S*,2*R*)-1-(4-bromophenyl)-2-(6-chloropyridin-3-yl)cyclopropane-1-carboxylate crystalized from CH_2Cl_2 for determination of absolute stereo-configuration.

JCS-24-8-2020_Mo

Submitted by: **Jack Sharland, Davies Group**

Solved by: **John Bacsa**

EMORY UNIVERSITY | X-ray Crystallography Center

R_1=2.44%

Crystal Data and Experimental

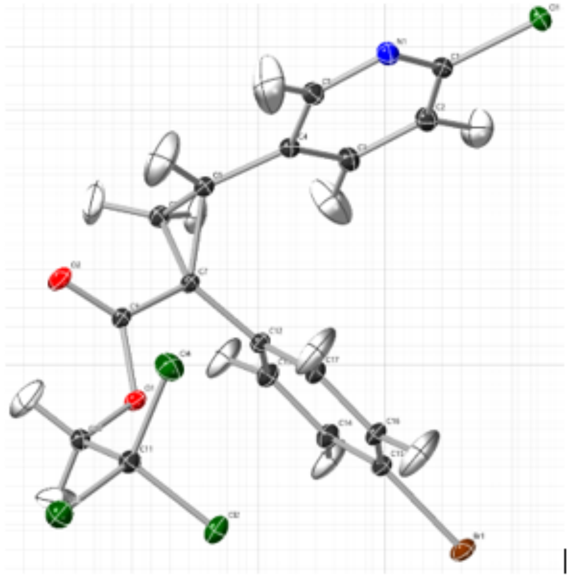

Z' = 1, μ(Mo K_α) = 2.840 mm^{-1}, 29643 reflections measured, 9440 unique (R_{int} = 0.0349) which were used in all calculations. The final wR_2 was 0.0510 (all data) and R_1 was 0.0244 (I≥2 σ(I)).

Experimental. A single colorless prismatic crystal of **JCS-24-8-2020_Mo** with dimensions 0.33 × 0.28 × 0.22 mm³ was chosen from the sample as supplied and mounted on a loop with paratone on a XtaLAB Synergy-S diffractometer. The crystal was kept at a constant T = 100.0(1) K during data collection. The structure was solved with the ShelXT (Sheldrick, 2015) solution program using dual methods and by using Olex2 (Dolomanov et al., 2009) as the graphical interface. The model was refined with olex2.refine 1.3-dev (Bourhis et al., 2015) using full matrix least squares minimisation on F^2.

Crystal Data. $C_{17}H_{12}BrCl_4NO_2$, M_r = 484.004, monoclinic, $P2_1$ (No. 4), a = 5.95245(14) Å, b = 9.2860(2) Å, c = 16.6609(4) Å, β = 96.220(2)°, α = γ = 90°, V = 915.50(4) Å³, T = 100.00(10) K, Z = 2,

**Compound**	**JCS-24-8-2020_Mo**
Formula	$C_{17}H_{12}BrCl_4NO_2$
$D_{calc.}$/ g cm^{-3}	1.756
μ/mm^{-1}	2.840
Formula Weight	484.004
Color	colorless
Shape	prism
Size/mm^3	0.33×0.28×0.22
T/K	100.00(10)
Crystal System	monoclinic
Flack Parameter	0.015(3)
Hooft Parameter	-0.001(2)
Space Group	$P2_1$
a/Å	5.95245(14)
b/Å	9.2860(2)
c/Å	16.6609(4)
α/°	90
β/°	96.220(2)
γ/°	90
V/Å^3	915.50(4)
Z	2
Z'	1
Wavelength/Å	0.71073
Radiation type	Mo K_α
Θ_{min}/°	2.46
Θ_{max}/°	38.07
Measured Refl's.	29643
Indep't Refl's	9440
Refl's I≥2 σ(I)	8716
R_{int}	0.0349
Parameters	347
Restraints	40
Largest Peak	0.5379
Deepest Hole	-0.4013
GooF	0.9915
wR_2 (all data)	0.0510
wR_2	0.0500
R_1 (all data)	0.0288
R_1	0.0244

The Flack parameter was refined to 0.01(1). Determination of absolute structure using Bayesian statistics on Bijvoet differences using the Olex2 results in -0.001(2). Note: The Flack parameter is used to determine chirality of the crystal studied, the value should be near 0, a value of 1 means that the stereochemistry is wrong and the model should be inverted. A value of 0.5 means that the crystal consists of a racemic mixture of the two enantiomers.

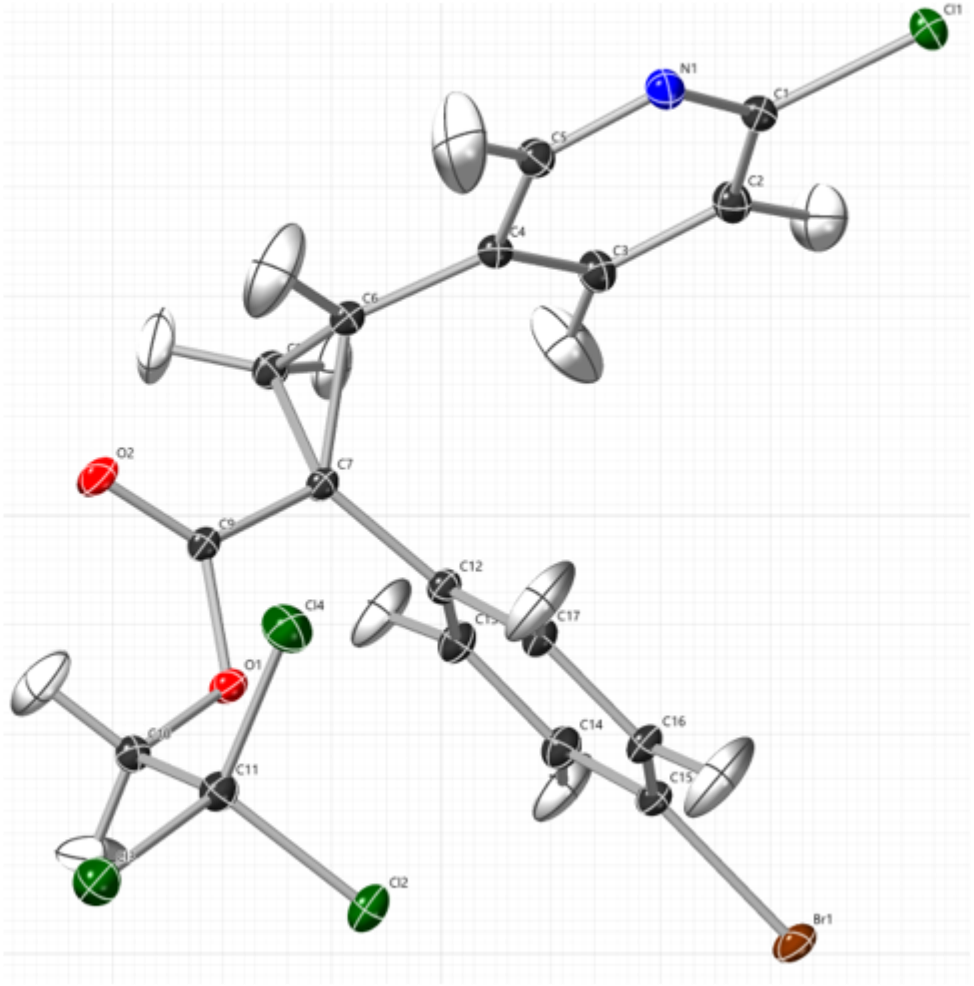

Figure 1: Thermal ellipsoid representation of the asymmetric unit. There is one molecule of the target compound in the asymmetric unit, which is represented by the reported sum formula. In other words: Z is 2 and Z' is 1.

Data Plots: Diffraction Data

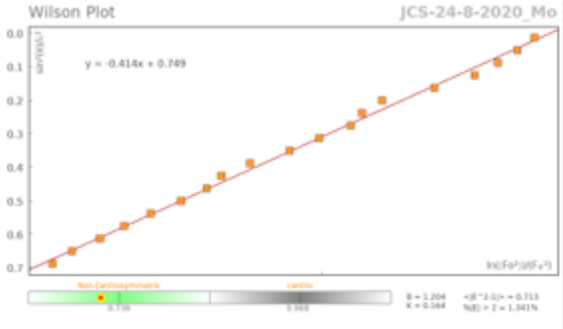

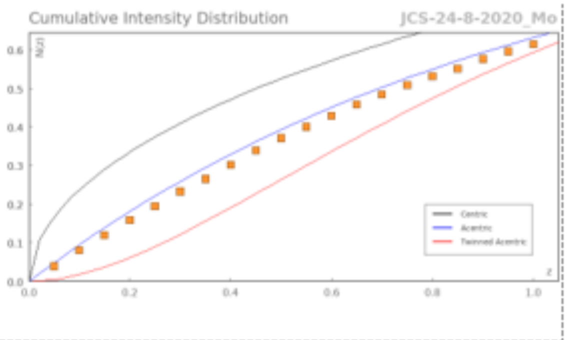

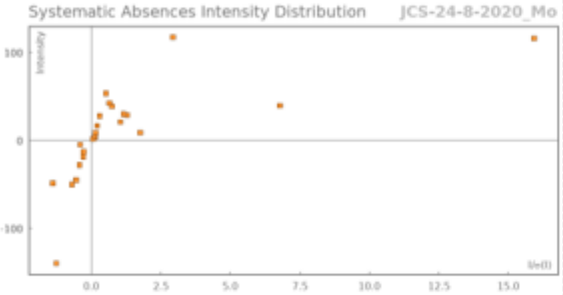

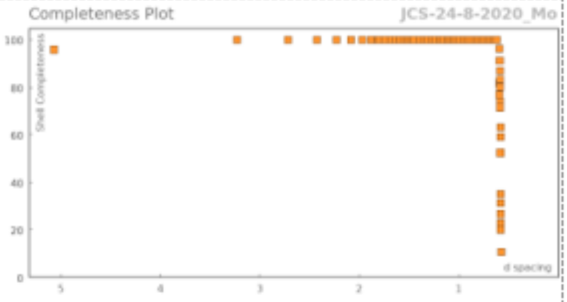

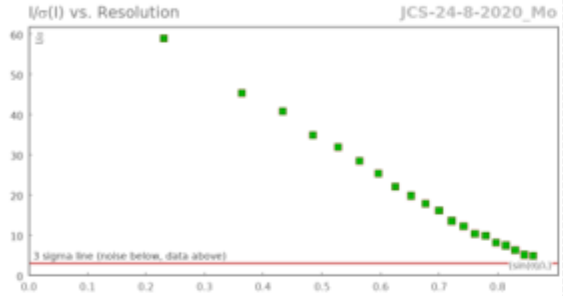

Data Plots: Refinement and Data

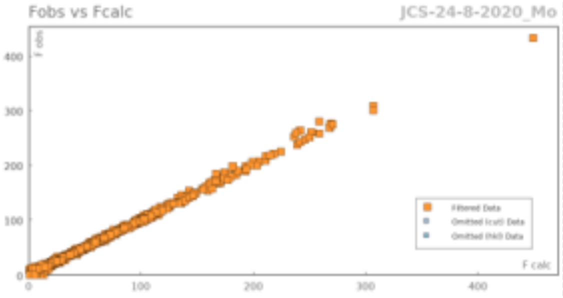

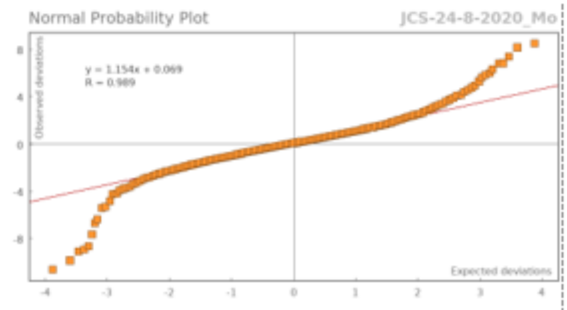

Reflection Statistics

Total reflections (after filtering)	29668	Unique reflections	9440

Table 1: Fractional Atomic Coordinates (×10⁴) and Equivalent Isotropic Displacement Parameters (Å²×10³) for **JCS-24-8-2020_Mo.** U_{eq} is defined as 1/3 of the trace of the orthogonalised U_{ij}.

Atom	x	y	z	U_{eq}
Br1	9489.9(4)	1071.2(3)	2773.30(15)	17.09(8)
Cl1	10893.2(5)	7812.1(4)	-710.80(16)	17.14(5)
Cl3	5844.1(5)	8112.4(4)	5800.00(16)	19.76(6)
Cl2	8033.0(4)	5965.6(4)	4902.20(16)	18.09(5)
Cl4	7375.4(5)	8821.2(4)	4248.00(18)	20.01(6)
O1	4056.1(13)	6478.6(9)	3647.9(5)	12.68(14)
O2	1964.1(16)	8273.1(11)	3042.8(5)	22.16(19)
N1	8424.2(17)	8920.8(11)	329.6(6)	15.78(17)
C3	5994.9(19)	6406.3(12)	530.4(7)	14.08(19)
C2	7616.4(19)	6445.8(12)	-6.2(7)	14.31(18)
C17	7408.4(18)	5337.3(12)	2649.1(6)	12.40(17)
C7	3712.1(17)	6568.7(12)	2229.2(6)	11.41(16)
C12	5167.6(17)	5253.4(12)	2302.6(6)	11.09(17)
C9	3135.2(18)	7227.2(12)	2999.7(6)	12.70(17)
C8	1890.1(18)	6736.7(13)	1529.0(6)	14.46(19)
C4	5596.6(18)	7633.3(12)	982.7(6)	11.77(17)
C1	8780.0(18)	7731.7(13)	-70.1(6)	13.08(17)
C14	5546.3(18)	2654.4(13)	2223.9(6)	14.44(18)
C6	3885.6(18)	7719.5(13)	1563.8(6)	13.11(18)
C5	6860(2)	8858.6(13)	849.2(6)	14.82(18)
C16	8710.7(18)	4099.8(12)	2791.4(6)	13.12(17)
C15	7756.9(18)	2766.5(12)	2580.3(6)	12.85(17)
C13	4268.5(18)	3904.9(13)	2089.0(6)	13.12(17)
C10	3847.7(18)	7058.3(13)	4426.5(6)	12.64(17)
C11	6187.9(19)	7464.3(12)	4824.4(6)	13.38(18)

Table 2: Anisotropic Displacement Parameters (×10⁴) for **JCS-24-8-2020_Mo.** The anisotropic displacement factor exponent takes the form: $-2\pi^2[h^2a^{*2} \times U_{11} + ... + 2hka^* \times b^* \times U_{12}]$

Atom	U_{11}	U_{22}	U_{33}	U_{23}	U_{13}	U_{12}
Br1	21.06(15)	10.90(13)	19.24(14)	6.32(12)	1.92(10)	2.03(12)
Cl1	16.51(12)	20.98(13)	14.81(10)	2.22(10)	5.78(9)	2.08(9)
Cl3	23.66(13)	22.06(14)	13.0(1)	3.31(11)	-0.55(9)	-4.87(9)
Cl2	14.88(10)	17.36(12)	21.36(11)	4.50(11)	-1.09(8)	-1.21(10)
Cl4	22.63(13)	15.39(12)	22.72(12)	-5.67(10)	5.68(10)	-0.10(9)
O1	15.1(3)	11.5(3)	11.2(3)	3.6(3)	0.5(3)	0.4(2)
O2	28.9(5)	22.7(4)	15.5(4)	17.3(4)	5.1(3)	3.8(3)
N1	17.9(4)	12.1(4)	18.3(4)	0.8(3)	6.4(3)	1.6(3)
C3	14.3(4)	14.0(5)	14.4(4)	-0.9(3)	3.3(3)	-1.6(3)
C2	15.4(4)	14.8(4)	12.9(4)	0.5(3)	2.4(3)	-2.1(3)
C17	10.0(4)	11.8(4)	15.1(4)	1.5(3)	-0.0(3)	-0.4(3)
C7	10.6(4)	12.4(4)	11.1(4)	3.0(3)	0.8(3)	1.0(3)
C12	10.2(4)	11.4(4)	11.5(4)	1.6(3)	0.4(3)	0.3(3)
C9	12.6(4)	13.6(4)	12.1(4)	4.4(3)	2.0(3)	1.5(3)
C8	10.4(4)	19.4(5)	13.3(4)	2.8(4)	-0.1(3)	2.4(4)
C4	12.3(4)	12.2(4)	10.8(4)	1.3(3)	1.5(3)	1.2(3)
C1	13.5(4)	14.2(4)	11.7(4)	1.7(3)	2.1(3)	1.2(3)
C14	14.5(5)	10.8(4)	17.9(4)	0.5(3)	1.2(4)	-1.7(3)
C6	13.0(4)	14.1(5)	12.4(4)	3.7(4)	2.1(3)	2.4(3)
C5	18.6(5)	11.0(4)	15.7(4)	1.8(4)	5.6(3)	0.3(3)
C16	11.1(4)	12.8(4)	14.9(4)	3.3(3)	-0.6(3)	0.4(3)

Atom	U_{11}	U_{22}	U_{33}	U_{23}	U_{13}	U_{12}
C15	14.4(4)	11.2(4)	13.1(4)	3.5(3)	2.1(3)	1.1(3)
C13	10.8(4)	12.2(4)	15.7(4)	0.6(3)	-1.1(3)	-1.9(3)
C10	12.8(4)	13.7(4)	11.5(4)	1.8(3)	1.9(3)	0.1(3)
C11	13.8(4)	12.2(4)	13.9(4)	0.3(3)	0.7(3)	-1.4(3)
H8a	13(8)	35(7)	28(8)	4(6)	-2(7)	-12(4)
H2	47(14)	24(7)	31(10)	7(6)	9(8)	-12(4)
H13	16(2)	11(3)	58(9)	1.7(10)	-11.9(17)	-4.6(15)
H10a	38(10)	28(6)	41(12)	21(4)	-6(8)	-6(7)
H17	19(2)	14(3)	78(10)	3.0(10)	-18.4(18)	-6.6(17)
H3	58(13)	29(6)	44(13)	-26(4)	28(10)	-12(6)
H16	19(2)	14(3)	78(10)	3.0(10)	-18.4(18)	-6.6(17)
H8b	19(6)	53(14)	45(13)	17(4)	7(5)	-5(9)
H6	32(11)	25(4)	82(16)	9(4)	-3(11)	-23(3)
H14	16(2)	11(3)	58(9)	1.7(10)	-11.9(17)	-4.6(15)
H10b	21(9)	44(11)	33(10)	-13(5)	-8(6)	25(5)
H5	51(13)	32(7)	74(14)	-5(7)	29(10)	-33(4)

Table 3: Bond Lengths in Å for **JCS-24-8-2020_Mo.**

Atom	Atom	Length/Å
Br1	C15	1.8905(11)
Cl1	C1	1.7373(11)
Cl3	C11	1.7658(11)
Cl2	C11	1.7689(11)
Cl4	C11	1.7767(11)
O1	C9	1.3497(13)
O1	C10	1.4223(13)
O2	C9	1.2022(14)
N1	C1	1.3184(15)
N1	C5	1.3397(14)
C3	C2	1.3853(16)
C3	C4	1.4004(15)
C2	C1	1.3903(16)
C17	C12	1.3970(15)
C17	C16	1.3921(15)
C7	C12	1.4948(15)
C7	C9	1.4951(15)
C7	C8	1.5124(14)
C7	C6	1.5513(15)
C12	C13	1.3928(16)
C8	C6	1.4941(16)
C4	C6	1.4814(15)
C4	C5	1.3949(16)
C14	C15	1.3877(15)
C14	C13	1.3930(16)
C16	C15	1.3913(16)
C10	C11	1.5238(15)

Table 4: Bond Angles in ° for **JCS-24-8-2020_Mo.**

Atom	Atom	Atom	Angle/°
C10	O1	C9	117.83(9)
C5	N1	C1	116.82(10)
C4	C3	C2	119.75(10)
C1	C2	C3	117.38(10)
C16	C17	C12	120.90(10)
C9	C7	C12	116.68(8)
C8	C7	C12	120.55(9)
C8	C7	C9	114.07(9)
C6	C7	C12	122.58(9)
C6	C7	C9	111.79(9)
C6	C7	C8	58.36(7)
C7	C12	C17	120.48(10)
C13	C12	C17	118.62(10)
C13	C12	C7	120.69(9)
O2	C9	O1	123.79(10)
C7	C9	O1	111.42(9)
C7	C9	O2	124.77(10)
C6	C8	C7	62.12(7)
C6	C4	C3	124.34(10)
C5	C4	C3	116.89(10)
C5	C4	C6	118.73(10)
N1	C1	Cl1	116.17(9)
C2	C1	Cl1	118.94(8)
C2	C1	N1	124.89(10)
C13	C14	C15	118.84(10)
C8	C6	C7	59.52(7)
C4	C6	C7	122.24(9)
C4	C6	C8	122.79(10)
C4	C5	N1	124.24(11)
C15	C16	C17	119.15(10)

Atom	Atom	Atom	Angle/°
C14	C15	Br1	119.01(9)
C16	C15	Br1	119.86(8)
C16	C15	C14	121.13(10)
C14	C13	C12	121.35(9)
C11	C10	O1	108.94(8)
Cl2	C11	Cl3	109.49(6)
Cl4	C11	Cl3	110.39(6)
Cl4	C11	Cl2	108.50(6)
C10	C11	Cl3	107.05(7)
C10	C11	Cl2	111.61(8)
C10	C11	Cl4	109.80(8)

Table 5: Torsion Angles in ° for **JCS-24-8-2020_Mo.**

Atom	Atom	Atom	Atom	Angle/°
Br1	C15	C14	C13	179.45(8)
Br1	C15	C16	C17	-179.67(8)
Cl1	C1	N1	C5	-177.51(8)
Cl1	C1	C2	C3	177.89(9)
Cl3	C11	C10	O1	-177.85(7)
Cl2	C11	C10	O1	-58.06(8)
Cl4	C11	C10	O1	62.30(8)
O1	C9	C7	C12	-0.48(11)
O1	C9	C7	C8	147.45(9)
O1	C9	C7	C6	-148.66(9)
O2	C9	C7	C12	-179.07(12)
O2	C9	C7	C8	-31.14(14)
O2	C9	C7	C6	32.74(13)
N1	C1	C2	C3	-1.38(13)
N1	C5	C4	C3	-0.88(13)
N1	C5	C4	C6	-178.81(11)
C3	C4	C6	C7	50.03(12)
C3	C4	C6	C8	-22.09(12)
C17	C12	C7	C9	-66.46(11)
C17	C12	C7	C8	147.79(10)
C17	C12	C7	C6	78.02(11)
C17	C12	C13	C14	0.83(12)
C17	C16	C15	C14	0.70(12)
C7	C12	C13	C14	-173.95(9)
C7	C8	C6	C4	110.93(7)
C7	C6	C4	C5	-132.20(11)
C12	C13	C14	C15	0.14(12)
C8	C6	C4	C5	155.68(10)

Table 6: Hydrogen Fractional Atomic Coordinates (×10⁴) and Equivalent Isotropic Displacement Parameters (Å^2×10^3) for **JCS-24-8-2020_Mo.** U_{eq} is defined as 1/3 of the trace of the orthogonalised U_{ij}.

Atom	x	y	z	U_{eq}
H8a	1790(30)	5870(30)	1081(10)	26(5)
H2	8020(40)	5460(20)	-375(12)	34(5)
H13	2500(30)	3750(20)	1856(12)	30(3)
H10a	2720(30)	7980(20)	4382(12)	36(5)
H17	8310(30)	6360(20)	2844(13)	39(4)
H3	4950(40)	5380(20)	603(13)	42(6)
H16	10380(30)	4170(20)	3016(13)	39(4)
H8b	380(30)	7270(20)	1655(12)	39(6)
H6	3590(30)	8860(20)	1769(14)	47(6)

Atom	x	y	z	U_{eq}
H14	4780(30)	1700(20)	2079(12)	30(3)
H10b	3140(30)	6220(30)	4781(11)	34(5)
H5	6590(30)	9800(20)	1196(13)	51(6)

Citations

CrysAlisPro (ROD), Rigaku Oxford Diffraction, Poland (?).

CrysAlisPro Software System, Rigaku Oxford Diffraction, (2020).

L.J. Bourhis and O.V. Dolomanov and R.J. Gildea and J.A.K. Howard and H. Puschmann, The Anatomy of a Comprehensive Constrained, Restrained, Refinement Program for the Modern Computing Environment - Olex2 Disected, *Acta Cryst. A*, (2015), **A71**, 59-71.

O.V. Dolomanov and L.J. Bourhis and R.J. Gildea and J.A.K. Howard and H. Puschmann, Olex2: A complete structure solution, refinement and analysis program, *J. Appl. Cryst.*, (2009), **42**, 339-341.

, *J. Appl. Cryst.*, (2009), **42**, 339-341.

7.3: Methyl (1*S*,2*S*)-2-(2-chloropyridin-3-yl)-1-(2-methoxy-5-methylphenyl)cyclopropane-1-carboxylate crystalized from IPA for determination of absolute stereo-configuration.

JCS-ELN46-0036

Rigaku oxford diffraction

Submitted by: **Jack Sharland**
Emory University
Solved by: **John Bacsa**
Sample ID: **JCS-ELN46-0036**

R_1=2.51%

Crystal Data and Experimental

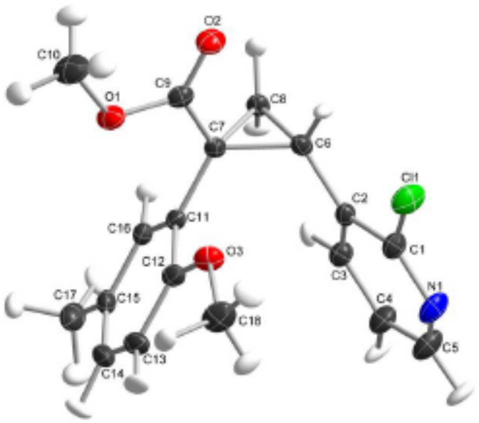

Experimental. Single colourless block-shaped crystals of **JCS-ELN46-0036** were The crystal was chosen from the sample as supplied.. A suitable crystal 0.78×0.51×0.30 mm³ was selected and mounted on a loop with paratone oil on an XtaLAB Synergy, Dualflex, HyPix diffractometer. The crystal was kept at a steady T = 102(3) K during data collection. The structure was solved with the ? structure solution program using the None methods solution method and by using **Olex2** (Dolomanov et al., 2009) as the graphical interface. The model was refined with version of olex2.refine 1.3-alpha (Bourhis et al., 2015) using Levenberg-Marquardt minimisation.

Crystal Data. $C_{18}H_{18}ClNO_3$, M_r = 331.801, orthorhombic, $P2_12_12_1$ (No. 19), a = 8.77283(16) Å, b = 12.4768(2) Å, c = 15.4124(3) Å, $\alpha = \beta = \gamma = 90°$, V = 1686.99(5) Å³, T = 102(3) K, Z = 4, Z' = 1, μ(Mo K$_\alpha$) = 0.240, 42004 reflections measured, 8753 unique (R_{int} = 0.0281) which were used in all calculations. The final wR_2 was 0.0642 (all data) and R_1 was 0.0251 (I≥σ(I)).

Compound	JCS-ELN46-0036
Formula	$C_{18}H_{18}ClNO_3$
$D_{calc.}$/ g cm^{-3}	1.306
μ/mm^{-1}	0.240
Formula Weight	331.801
Colour	colourless
Shape	block
Size/mm³	0.78×0.51×0.30
T/K	102(3)
Crystal System	orthorhombic
Flack Parameter	-0.04(3)
Hooft Parameter	-0.008(9)
Space Group	$P2_12_12_1$
a/Å	8.77283(16)
b/Å	12.4768(2)
c/Å	15.4124(3)
α/°	90
β/°	90
γ/°	90
V/Å³	1686.99(5)
Z	4
Z'	1
Wavelength/Å	0.71073
Radiation type	Mo K$_\alpha$
Θ_{min}/°	2.10
Θ_{max}/°	38.16
Measured Refl.	42004
Independent Refl.	8753
Reflections with I≥σ(I)	8164
R_{int}	0.0281
Parameters	371
Restraints	78
Largest Peak	0.3291
Deepest Hole	-0.2258
GooF	1.0429
wR_2 (all data)	0.0642
wR_2	0.0632
R_1 (all data)	0.0281
R_1	Oooops!

Structure Quality Indicators

**Reflections:**	d min (Mo) 0.58	I/σ 49.8	Rint 2.81%	Complete 100% (IUCr) 100%	
**Refinement:**	Shift 0.003	Max Peak 0.3	Min Peak -0.2	GooF 1.043	Flack -.04(3)

Experimental Extended. A colourless block-shaped crystal with dimensions 0.78×0.51×0.30 mm³ was mounted on a loop with paratone oil. Data were collected using an XtaLAB Synergy, Dualflex, HyPix diffractometer equipped with an Oxford Cryosystems low-temperature device operating at T = 102(3) K.

Data were measured using ω scans of 0.5° per frame for 7.0/9.0 s using Mo K_{α} radiation. The total number of runs and images was based on the strategy calculation from the program CrysAlisPro (Rigaku, V1.171.40.67a, 2019) The maximum resolution that was achieved was Θ = 38.16° (0.58 Å).

The diffraction pattern was indexed The total number of runs and images was based on the strategy calculation from the program CrysAlisPro (Rigaku, V1.171.40.67a, 2019) and the unit cell was refined using CrysAlisPro (Rigaku, V1.171.40.67a, 2019) on 28796 reflections, 69% of the observed reflections.

Data reduction, scaling and absorption corrections were performed using CrysAlisPro (Rigaku, V1.171.40.67a, 2019). The final completeness is 100.00 % out to 38.16° in Θ. A multi-scan absorption correction was performed using CrysAlisPro 1.171.40.67a (Rigaku Oxford Diffraction, 2019) using spherical harmonicsas implemented in SCALE3 ABSPACK.. The absorption coefficient μ of this material is 0.240 mm⁻¹ at this wavelength (λ = 0.711Å) and the minimum and maximum transmissions are 0.657 and 1.000.

The structure was solved and the space group $P2_12_12_1$ (# 19) determined by the ? structure solution program using None methods and refined by Levenberg-Marquardt using version of olex2.refine 1.3-alpha (Bourhis et al., 2015). All non-hydrogen atoms were refined anisotropically. Hydrogen atom positions were calculated geometrically and refined using the riding model.

_refine_special_details: Refinement using NoSpherA2, an implementation of NOn-SPHERical Atom-form-factors in Olex2.Please cite:F. Kleemiss, H. Puschmann, O. Dolomanov, S.Grabowsky - to be publsihed - 2020NoSpherA2 makes use of tailor-made aspherical atomic form factors calculatedon-the-fly from a Hirshfeld-partitioned electron density (ED) - not fromspherical-atom form factors.The ED is calculated from a gaussian basis set single determinant SCFwavefunction - either Hartree-Fock or B3LYP - for a fragment of the crystal embedded inan electrostatic crystal field.The following options were used: SOFTWARE: Tonto METHOD: rks BASIS SET: STO-3G CHARGE: 0 MULTIPLICITY: 1 DATE: 2020-01-03_16-47-16 CLUSTER RADIUS: 0

_exptl_absorpt_process_details: CrysAlisPro 1.171.40.67a (Rigaku Oxford Diffraction, 2019) using spherical harmonicsas implemented in SCALE3 ABSPACK.

Table 1: Fractional Atomic Coordinates (×10⁴) and Equivalent Isotropic Displacement Parameters (Å²×10³) for **JCS-ELN46-0036**. U_{eq} is defined as 1/3 of the trace of the orthogonalised U_{ij}.

Atom	x	y	z	U_{eq}
Cl1	7061.2(2)	2760.02(13)	3319.86(12)	31.82(4)
O1	4349.7(6)	4960.6(4)	6032.7(3)	24.65(9)
O3	3521.2(6)	3493.5(4)	4489.9(3)	23.14(9)
O2	6826.1(6)	4856.6(4)	5724.3(3)	25.58(9)
N1	5528.8(9)	3306.0(5)	1949.0(4)	29.09(12)
C16	2642.1(6)	6306.1(5)	4219.7(3)	16.51(8)
C12	2662.7(6)	4378.1(5)	4341.0(4)	18.35(9)

Atom	x	y	z	U_{eq}
C15	1089.2(6)	6303.2(5)	4009.3(4)	19.83(9)
C9	5536.8(7)	5017.8(4)	5500.6(4)	18.72(9)
C11	3433.8(6)	5364.5(4)	4387.1(3)	14.89(8)
C7	5088.1(6)	5356.5(4)	4602.7(3)	15.11(8)
C13	1110.8(7)	4360.2(5)	4147.1(4)	23.25(11)
C14	346.8(6)	5320.7(6)	3987.5(4)	23.68(11)
C8	6170.1(6)	6166.3(4)	4205.6(4)	17.89(9)
C4	4432.4(8)	5017.5(5)	1668.0(4)	25.20(11)
C6	6251.3(6)	5026.0(4)	3904.9(4)	17.33(8)
C3	4920.4(7)	5378.4(5)	2473.9(4)	20.72(9)
C2	5723.9(6)	4699.8(5)	3030.4(4)	17.50(8)
C17	265.3(8)	7332.0(6)	3805.8(5)	28.40(13)
C5	4751.0(10)	3971.2(6)	1434.1(4)	30.29(14)
C1	5994.1(8)	3670.7(5)	2704.4(4)	22.22(10)
C18	2874.4(10)	2476.0(5)	4312.0(5)	31.48(14)
C10	4684.5(11)	4701.7(8)	6917.2(5)	36.96(17)

Table 2: Anisotropic Displacement Parameters (×10⁴) **JCS-ELN46-0036. The anisotropic displacement factor exponent takes the form:** $-2\pi^2[h^2a^{*2} \times U_{11} + \ldots + 2hka^* \times b^* \times U_{12}]$

Atom	U_{11}	U_{22}	U_{33}	U_{23}	U_{13}	U_{12}
Cl1	42.08(9)	19.44(6)	33.95(8)	9.00(6)	-9.06(7)	-6.84(6)
O1	26.3(2)	30.5(2)	17.16(16)	-4.99(19)	-2.36(15)	3.48(17)
O3	26.8(2)	15.65(17)	27.0(2)	-4.97(15)	-2.79(18)	2.62(16)
O2	26.0(2)	26.6(2)	24.2(2)	5.40(17)	-7.76(17)	0.54(17)
N1	44.2(3)	19.8(2)	23.3(2)	4.7(2)	-2.5(2)	-8.0(2)
C16	17.26(19)	17.4(2)	14.90(19)	0.68(16)	1.02(15)	-1.62(16)
C12	19.9(2)	17.5(2)	17.6(2)	-5.60(17)	1.17(17)	0.64(17)
C15	17.3(2)	26.4(2)	15.8(2)	3.40(19)	1.73(16)	-2.82(19)
C9	23.2(2)	13.93(19)	19.0(2)	-0.61(17)	-3.78(17)	-0.21(17)
C11	15.57(17)	15.15(18)	13.96(18)	-1.94(15)	0.81(14)	-0.09(16)
C7	16.29(18)	12.58(18)	16.44(18)	-1.16(15)	-0.57(15)	-0.43(15)
C13	20.0(2)	27.6(3)	22.1(2)	-9.1(2)	2.04(19)	0.1(2)
C14	15.34(19)	34.4(3)	21.3(2)	-2.9(2)	1.98(17)	-2.8(2)
C8	17.7(2)	14.59(19)	21.4(2)	-3.31(16)	0.70(17)	-1.65(17)
C4	33.7(3)	23.2(2)	18.7(2)	0.8(2)	-1.1(2)	-1.7(2)
C6	15.25(18)	15.51(19)	21.2(2)	-0.87(16)	0.46(16)	-2.74(17)
C3	24.1(2)	18.6(2)	19.4(2)	0.57(19)	0.84(18)	-1.80(18)
C2	17.19(19)	16.21(19)	19.1(2)	-0.62(17)	2.71(16)	-2.70(17)
C17	24.7(3)	32.8(3)	27.7(3)	11.6(2)	-2.7(2)	-5.8(3)
C5	44.8(4)	25.8(3)	20.3(2)	1.5(3)	-2.8(2)	-6.3(2)
C1	26.7(3)	18.2(2)	21.7(2)	1.9(2)	1.1(2)	-4.32(19)
C18	42.2(4)	17.3(2)	34.9(3)	-9.4(3)	0.3(3)	0.4(2)
C10	44.2(4)	48.0(4)	18.7(2)	-10.4(4)	-5.9(3)	9.6(3)
H8a	39(6)	30(5)	40(6)	-11(5)	3(5)	-6(5)
H13	41(6)	38(4)	48(7)	-25(3)	2(5)	2(3)
H8b	39(6)	28(5)	37(6)	-11(4)	-16(5)	7(5)
H6	33(5)	53(6)	29(5)	7(5)	-11(5)	-16(5)
H3	36(6)	29(3)	53(6)	0(2)	18(5)	-12(2)
H16	34(5)	24(3)	40(6)	-8(2)	1(4)	0(3)
H4	81(8)	30(5)	28(5)	17(4)	-21(4)	-11(3)
H18a	58(8)	17(5)	99(11)	9(5)	-7(8)	0(6)
H17a	50(5)	53(6)	45(4)	14(3)	5(2)	-10(2)
H5	91(10)	48(7)	24(4)	-1(7)	-17(3)	-11(3)
H17b	44(4)	37(4)	75(7)	17(2)	9(3)	9(3)
H18b	79(9)	33(6)	43(6)	-1(6)	-8(6)	-12(5)
H18c	50(7)	45(7)	46(7)	-20(6)	11(6)	-14(6)
H14	21(3)	61(7)	59(7)	-7(3)	-10(2)	12(7)
H10a	65(5)	69(5)	39(5)	-21(3)	-15(3)	9(3)
H17c	65(5)	72(7)	59(5)	19(3)	-26(3)	-9(3)
H10b	55(4)	103(8)	24(4)	-12(2)	-3(2)	9(3)
H10c	74(7)	63(4)	42(6)	0(2)	-10(4)	10(2)

Table 3: Bond Lengths in Å for **JCS-ELN46-0036.**

Atom	Atom	Length/Å
Cl1	C1	1.7514(7)
O1	C9	1.3275(8)
O1	C10	1.4315(8)
O3	C12	1.3556(7)
O3	C18	1.4173(8)
O2	C9	1.1994(7)
N1	C5	1.3358(10)
N1	C1	1.3150(8)
C16	C15	1.4004(8)
C16	C11	1.3890(8)
C12	C11	1.4062(7)
C12	C13	1.3941(8)
C15	C14	1.3885(9)
C15	C17	1.5061(9)
C9	C7	1.4995(8)
C11	C7	1.4889(7)
C7	C8	1.5154(7)
C7	C6	1.5389(7)
C13	C14	1.3949(10)
C8	C6	1.4980(7)
C4	C3	1.3887(9)
C4	C5	1.3828(10)
C6	C2	1.4819(8)
C3	C2	1.3963(8)
C2	C1	1.3990(8)

Table 4: Bond Angles in ° for **JCS-ELN46-0036.**

Atom	Atom	Atom	Angle/°
C10	O1	C9	116.07(6)
C18	O3	C12	118.30(5)
C1	N1	C5	118.00(6)
C11	C16	C15	121.82(5)
C11	C12	O3	115.89(5)
C13	C12	O3	124.46(5)
C13	C12	C11	119.65(5)
C14	C15	C16	117.66(6)
C17	C15	C16	120.85(6)
C17	C15	C14	121.49(5)
O2	C9	O1	123.59(5)
C7	C9	O1	112.29(5)
C7	C9	O2	124.06(5)
C12	C11	C16	119.36(5)
C7	C11	C16	122.31(5)
C7	C11	C12	118.32(5)
C11	C7	C9	117.63(4)
C8	C7	C9	113.34(4)
C8	C7	C11	121.06(5)
C6	C7	C9	113.29(4)
C6	C7	C11	119.48(4)
C6	C7	C8	58.74(3)
C14	C13	C12	119.56(6)
C13	C14	C15	121.93(5)
C6	C8	C7	61.42(4)
C5	C4	C3	118.49(6)
C8	C6	C7	59.85(3)
C2	C6	C7	120.15(4)
C2	C6	C8	121.83(5)
C2	C3	C4	120.56(6)
C3	C2	C6	123.35(5)
C1	C2	C6	121.73(5)
C1	C2	C3	114.92(5)
C4	C5	N1	122.34(6)
N1	C1	Cl1	114.89(5)
C2	C1	Cl1	119.44(5)
C2	C1	N1	125.66(6)

Table 5: Torsion Angles in ° for **JCS-ELN46-0036.**

Atom	Atom	Atom	Atom	Angle/°
Cl1	C1	N1	C5	-177.85(6)
Cl1	C1	C2	C6	-3.12(6)
Cl1	C1	C2	C3	177.29(5)
O1	C9	C7	C11	-11.83(6)
O1	C9	C7	C8	137.51(5)
O1	C9	C7	C6	-157.94(5)
O3	C12	C11	C16	178.30(5)
O3	C12	C11	C7	-0.79(6)
O3	C12	C13	C14	-178.65(6)
O2	C9	C7	C11	170.90(6)
O2	C9	C7	C8	-39.76(7)
O2	C9	C7	C6	24.79(7)
N1	C5	C4	C3	-1.07(10)
N1	C1	C2	C6	177.86(6)
N1	C1	C2	C3	-1.72(8)
C16	C15	C14	C13	-1.52(6)
C16	C11	C12	C13	-1.41(6)
C16	C11	C7	C9	113.06(5)

Atom	Atom	Atom	Atom	Angle/°
C16	C11	C7	C8	-33.80(6)
C16	C11	C7	C6	-102.98(5)
C12	C11	C7	C9	-67.88(5)
C12	C11	C7	C8	145.26(5)
C12	C11	C7	C6	76.08(5)
C12	C13	C14	C15	0.47(7)
C9	C7	C8	C6	103.99(6)
C9	C7	C6	C8	-104.08(6)
C9	C7	C6	C2	144.33(4)
C11	C7	C8	C6	-107.84(7)
C11	C7	C6	C8	110.50(7)
C11	C7	C6	C2	-1.10(6)
C7	C8	C6	C2	108.85(4)
C7	C6	C2	C3	61.56(6)
C7	C6	C2	C1	-117.99(5)
C8	C6	C2	C3	-9.59(6)
C8	C6	C2	C1	170.86(5)
C4	C3	C2	C6	-178.77(5)
C4	C3	C2	C1	0.81(7)

Table 6: Hydrogen Fractional Atomic Coordinates (×10⁴) and Equivalent Isotropic Displacement Parameters (Å²×10³) for JCS-ELN46-0036. U_{eq} is defined as 1/3 of the trace of the orthogonalised U_{ij}.

Atom	x	y	z	U_{eq}
H8a	7032(13)	6448(8)	4596(7)	36(2)
H13	571(12)	3642(8)	4098(7)	42(3)
H8b	5687(12)	6735(8)	3799(7)	34(2)
H6	7154(12)	4556(9)	4162(6)	38(3)
H3	4716(12)	6160(8)	2648(7)	40(2)
H16	3233(11)	7027(7)	4232(7)	33(2)
H4	3783(15)	5523(8)	1253(7)	46(3)
H18a	3719(15)	1903(8)	4445(10)	58(4)
H17a	-313(13)	7637(10)	4353(7)	49(3)
H5	4428(16)	3685(10)	821(6)	54(4)
H17b	1066(14)	7930(9)	3632(9)	52(3)
H18b	2492(14)	2441(9)	3667(8)	52(3)
H18c	1859(13)	2311(9)	4718(7)	47(3)
H14	-803(11)	5263(10)	3812(8)	47(3)
H10a	5475(16)	5202(11)	7160(7)	58(3)
H17c	-571(16)	7243(12)	3330(8)	66(3)
H10b	3647(15)	4719(13)	7232(7)	61(3)
H10c	5258(17)	3927(11)	6967(8)	60(3)

Citations

O.V. Dolomanov and L.J. Bourhis and R.J. Gildea and J.A.K. Howard and H. Puschmann, Olex2: A complete structure solution, refinement and analysis program, *J. Appl. Cryst.*, (2009), 42, 339-341.

References

1. S. P. Green, K. M. Wheelhouse, A. D. Payne, J. P. Hallett, P. W. Miller and J. A. Bull, *Org. Process. Res. Dev.*, 2020, **24**, 67-84.
2. D. M. Guptill and H. M. L. Davies, *J. Am. Chem. Soc.*, 2014, **136**, 17718-17721.
3. K. M. Chepiga, C. Qin, J. S. Alford, S. Chennamadhavuni, T. M. Gregg, J. P. Olson and H. M. L. Davies, *Tetrahedron*, 2013, **69**, 5765-5771.
4. L. Fu, J. D. Mighion, E. A. Voight and H. M. L. Davies, *Chem. Eur. J.*, 2017, **23**, 3272-3275.
5. S. Harada, K. Tanikawa, H. Homma, C. Sakai, T. Ito and T. Nemoto, *Chem. Eur. J.*, 2019, **25**, 12058-12062.
6. X. B. Wang, Z. J. Zheng, J. L. Xie, X. W. Gu, Q. C. Mu, G. W. Yin, F. Ye, Z. Xu and L. W. Xu, *Angew. Chem. Int. Ed.*, 2020, **59**, 790-797.
7. L. Fu, K. Hoang, C. Tortoreto, W. Liu and H. M. L. Davies, *Org. Lett.*, 2018, **20**, 2399-2402.
8. H. M. L. Davies and R. J. Townsend, *J. Org. Chem.*, 2001, **66**, 6595-6603.
9. S. M. Nicolle and C. J. Moody, *Chem. Eur. J.*, 2014, **20**, 4420-4425.
10. P.-C. Lv, A.-Q. Jiang, W.-M. Zhang and H.-L. Zhu, *Expert. Opin. Ther. Pat.*, 2018, **28**, 139-145.
11. J. Fu, N. Wurzer, V. Lehner, O. Reiser and H. M. L. Davies, *Org. Lett.*, 2019, **21**, 6102-6106.
12. H. M. L. Davies, P. R. Bruzinski, D. H. Lake, N. Kong and M. J. Fall, *J. Am. Chem. Soc.*, 1996, **118**, 6897-6907.
13. R. P. Reddy, G. H. Lee and H. M. L. Davies, *Org. Lett.*, 2006, **8**, 3437-3440.

14. C. Qin and H. M. L. Davies, *J. Am. Chem. Soc.*, 2014, **136**, 9792-9796.
15. J. Fu, Z. Ren, J. Bacsa, D. G. Musaev and H. M. L. Davies, *Nature*, 2018, **564**, 395-399.
16. B. Wei, J. C. Sharland, P. Lin, S. M. Wilkerson-Hill, F. A. Fullilove, S. McKinnon, D. G. Blackmond and H. M. L. Davies, *ACS Catal.*, 2020, **10**, 1161-1170.
